# LIGHT in combination with IL-13 or IL-17 drives inflammatory transcriptional signatures in human pulmonary fibroblasts relevant for human lung disease

**DOI:** 10.1093/immhor/vlaf042

**Published:** 2025-09-17

**Authors:** Nandita Ghosh, Rinkesh Kumar Gupta, Jeamin Jung, Kai Fung, Michael Croft

**Affiliations:** Center for Autoimmunity and Inflammation, La Jolla Institute for Immunology, La Jolla, CA, United States; Center for Autoimmunity and Inflammation, La Jolla Institute for Immunology, La Jolla, CA, United States; Bioinformatics Core, La Jolla Institute for Immunology, La Jolla, CA, United States; Bioinformatics Core, La Jolla Institute for Immunology, La Jolla, CA, United States; Center for Autoimmunity and Inflammation, La Jolla Institute for Immunology, La Jolla, CA, United States

**Keywords:** asthma, interstitial lung disease pulmonary fibroblasts, TNFSF14 and LIGHT

## Abstract

Fibroblasts are structural cells primarily involved in tissue remodeling, but recent single-cell RNA sequencing (RNA-seq) transcriptomic data have highlighted their potential to display molecules linked to inflammation. The factors that drive such inflammatory transcriptional signatures found in patients are not clear. LIGHT (TNFSF14) is a cytokine that we previously suggested may be central to lung diseases exhibiting fibrosis and inflammation, including asthma and interstitial lung disease. With bulk RNA-seq, we then investigated the transcriptional activity of LIGHT in human pulmonary fibroblasts compared with interleukin (IL)-13 and IL-17, two other cytokines linked to lung disease. While all 3 cytokines individually induced unique and overlapping gene transcripts, when fibroblasts were stimulated with LIGHT and IL-13 they upregulated more inflammatory transcripts including *CCL2*, *CCL26*, *CXCL2*, *CXCL3*, *CXCL5*, *CXCL6*, *IL32*, *CSF2*, *VCAM1*, *ICAM1*, *IL18R1*, *IL1RL1*, *TNFRSF12A*, *TNFRSF4*, *TNFRSF8*, *ITGA2*, *ITGA4*, and *ITGAV*, and when stimulated with LIGHT and IL-17, inflammatory transcripts included *CXCL1*, *CXCL2*, *CXCL3*, *CXCL5*, *CXCL6*, *CXCL8*, *IL32*, *IL33*, *CSF2*, *TSLP*, *IL1A*, *IL6*, *IL18*, *VCAM1*, *ICAM1*, *IL18R1*, *IL1RL1*, *TNFSF4*, *TNFRSF4*, *TNFRSF8*, *ITGA2*, *ITGA4*, and *ITGAV.* Furthermore, multiple cell cycle–related transcripts were induced with these combinations. Providing potential disease significance, portions of the fibroblast transcriptional signatures induced in vitro were found to be present in subsets of fibroblasts defined by single-cell RNA-seq isolated from patients with interstitial lung disease. This study therefore highlights the synergistic activities of LIGHT with other classical cytokines to regulate transcription in pulmonary fibroblasts and infers the involvement of LIGHT in shaping fibroblast phenotypes observed in chronic lung disease.

## Introduction

Fibroblasts are stromal cells of mesenchymal origin, and have a primary potential to produce extracellular matrix in response to external stimuli. Innate molecules such as transforming growth factor β drive fibroblasts to upregulate extracellular matrix proteins that contribute to tissue fibrosis.^1^ Nevertheless, it has become clear from single-cell RNA sequencing (RNA-seq) that fibroblasts in tissues of patients with varying diseases can display multiple phenotypes at the transcriptional level. In particular, subsets of fibroblasts from chronic inflammatory diseases express gene signatures for cytokines, chemokines, cluster of differentiation markers, and other immune-related receptors, and can display antigen-processing and antigen-presenting characteristics.^2^^,^^3^ Moreover, it has been postulated that this alteration in fibroblast characteristics directs changes away from tissue sustaining activities toward greater inflammatory phases, which may contribute to the chronicity of diseases. This suggests that the fibroblast can be far more influential than simply being a cell relevant for tissue structural changes.^2^^,^^3^ Furthermore, some groups have suggested that such varied pathological phenotypes might be shared across tissues from multiple diverse diseases that exhibit both inflammation and fibrosis.^4^ However, which stimuli might promote such pro-inflammatory fibroblast transcriptional signatures seen in diseased tissue is not clear.

TNF superfamily (TNFSF) proteins play important roles in innate and adaptive immune responses and may be central to many immune diseases.^5^ One molecule, LIGHT (homologous to Lymphotoxin, exhibits Inducible expression and competes with HSV Glycoprotein D for binding to Herpesvirus entry mediator, a receptor expressed on T lymphocytes), also known as TNFSF14,^6^ is a cytokine that we have previously found to be involved in inflammation of the lungs in mouse models of asthma and pulmonary fibrosis.^7–10^ Initial data revealed that LIGHT could exhibit inflammatory activity in macrophages, eosinophils, and T cells relevant for lung inflammation,^10^^,^^11^ and more recently we identified activities of LIGHT in bronchial epithelial cells and airway smooth muscle cells that could further contribute to its role in the lungs.^7^^,^^9^^,^^12^ Importantly, the two receptors for LIGHT, HVEM (herpes virus entry mediator) and LTβR (lymphotoxin beta receptor), are constitutively expressed on most fibroblasts, and previously we found that LIGHT could promote cell cycle progression and proliferation of lung fibroblasts and induce select molecules such as ICAM-1 (intercellular adhesion molecule 1), VCAM-1 (vascular cell adhesion molecule 1), interleukin (IL)-6, GM-CSF (granulocyte-macrophage colony-stimulating factor), and CCL5, CXCL5, and CXCL11.^13^ Thus, LIGHT might be one factor relevant for fibroblast differentiation seen in diseases of the lungs. The present study was then designed to investigate the full potential of LIGHT to drive inflammatory transcriptional signatures in human pulmonary fibroblasts (HPFs) that might correlate with signatures found in single-cell RNA-seq analysis of fibroblasts from patients with lung disease. Importantly, given that other molecules are known to have receptors on fibroblasts, we asked how LIGHT might be similar or divergent compared with IL-13 and IL-17, two cytokines that have also been postulated to be involved in driving pathogenesis and inflammation in subsets of patients with diseases of the lung, including asthma, interstitial lung disease (ILD), idiopathic pulmonary fibrosis, and chronic obstructive pulmonary disease. We further tested if LIGHT cooperates with IL-13 and IL-17 to exacerbate inflammatory gene signatures. Last, we asked if these transcriptional changes in vitro correlated with pulmonary fibroblast gene signatures seen in ILD in order to infer whether LIGHT, alone or in combination, might be active in driving some of the transcriptional states that are being described from single-cell RNA-seq studies.

## Materials and methods

### Recombinant cytokines

Recombinant cytokines were purchased from R&D Systems: rLIGHT (664-LI-025/CF), r1L-13 (213-ILB-010), and rIL-17A (7955-IL-025/CF).

### HPF cell culture

Healthy HPF cells were obtained from ScienCell (Cat. #3300, lot #27039). A single cell line was used for all experiments, given that our prior studies did not show any major differences in transcriptional activity induced by LIGHT or other cytokines between multiple cell lines.^13^^,^^14^ Cells were cultured in fibroblast medium containing fetal bovine serum (ScienCell; Cat. #2301) and used at passage 3. HPF were serum starved for 18 h prior to cytokine treatment, culturing in fibroblast basal medium without any added supplements (Lonza; Cat. # CC3131). Cells were seeded at a concentration of 0.3 × 10^6^ cells/mL in 6-well plates.

### Cytokine treatment

HPF were stimulated with LIGHT, IL-13, and IL-17, either alone or in combination for 24 h, or cells were prestimulated with IL-13 or IL-17 for 72 h followed by LIGHT for 24 h. A 24-h time point was chosen, as our prior studies showed that this is the initial time when LIGHT is observed to drive strong transcription in lung fibroblasts.^13^ Cells were 40% to 60% confluent before LIGHT stimulation for 24 h. The cytokines were used at concentrations previously found to induce optimal gene transcription^9^^,^^13^^,^^15–17^: 50 ng/mL LIGHT, 25 ng/mL IL-13, and 100 ng/mL IL-17A for 24 h stimulation, and 50 ng/mL of IL-13 or IL-17A were used for 72 h prestimulation. We observed no detrimental impact on fibroblast health or viability with cytokine treatment, correlating with our previous study in which we found that LIGHT and IL-13 could promote proliferation of lung fibroblasts.^13^

### Transcriptome analysis

RNA was isolated from 3 different replicates using Zymo Direct-zol, and bulk RNA-seq was done with NEB Next Ultra II Directional in the sequencing core of the La Jolla Institute for Immunology. The paired-end reads that passed Illumina filters were filtered for reads aligning to tRNA, rRNA, adapter sequences, and spike-in controls. The reads were then aligned to GRCh38 reference genome and GENCODE v27 annotations using STAR (v2.6.1) (10.1093/bioinformatics/bts635). DUST scores were calculated with PRINSEQ Lite (v0.20.3), and low-complexity reads (DUST > 4) were removed from the BAM files. The alignment results were parsed via the SAMtools to generate SAM files. Read counts to each genomic feature were obtained with the featureCounts (v1.6.5) using the default option along with a minimum quality cutoff (Phred > 10).^18–21^ After removing absent features (zero counts in all samples), the raw counts were then imported to R/Bioconductor package DESeq2 (v1.24.0) to identify differentially expressed genes among samples. *P* values for differential expression were calculated using the Wald test for differences between the base means of two conditions.^22^ These P values were then adjusted for multiple test correction using the Benjamini-Hochberg algorithm.^23^ We considered genes differentially expressed between two groups of samples when the DESeq2 analysis resulted in an adjusted *P* value of <0.10 and the difference in gene expression was 1.5-fold or greater. Principal component analysis was performed using the “prcomp” function in R v4.2.3 (R Foundation for Statistical Computing). A row-scaled heatmap of log-transformed average TPM values was created with the “heatmap.2” function in ggplot2 v3.5.1 library in R.

For pathway analysis, gene set enrichment analysis was done using the “GseaPreranked” method with “classic” scoring scheme. All the Gene Ontology gene sets were downloaded from MSigDB in GMT format. Rank files for each DE comparison of interest were generated by assigning a rank of −log10(pValue) to genes with log2FoldChange >0 and a rank of log10(pValue) to genes with log2FoldChange <0.

### Comparison of bulk RNA-seq data with the single-cell atlas data

Gene transcripts upregulated in HPFs by LIGHT, IL-13, and IL-17, alone or in combination, from bulk RNA sequencing, were compared with previously published single-cell RNA-seq data to examine common transcriptional signatures found in lung fibroblasts from patients with ILD.^4^ We accessed the processed human sequencing data from the BroadSingleCellPortal (SCP738), selected lung fibroblasts for clustering and used lung tissue for cell type integrated annotation.^4^ The transcripts upregulated in HPF in vitro were represented in the latter datasets in dot plot format to show their expression profiles across the 14 clusters (0–13) identified in lung fibroblasts of ILD patients.

## Results

### LIGHT, IL-13, and IL-17 induce divergent as well as overlapping inflammatory gene transcripts in HPFs

**Figure 1.**
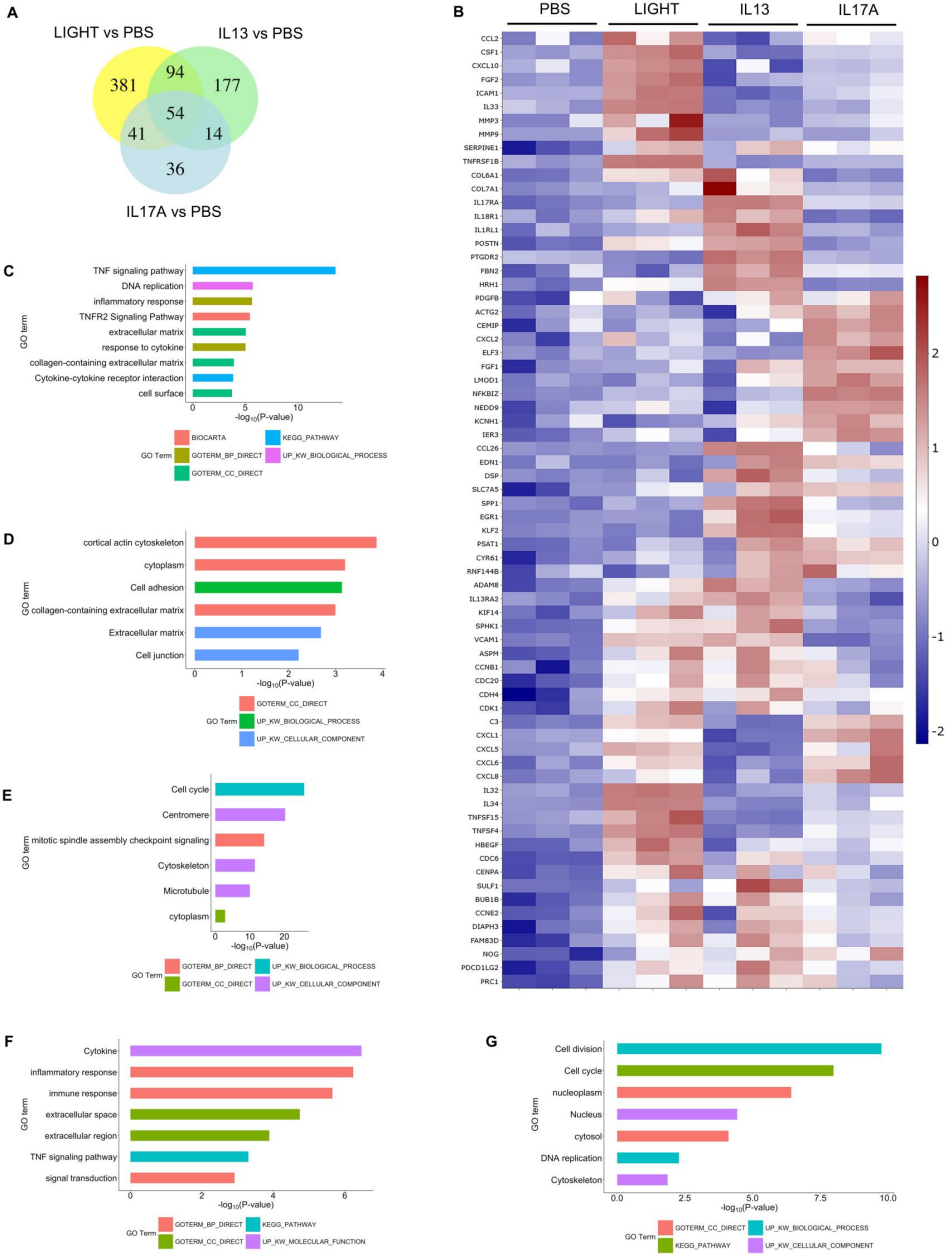
LIGHT, IL-13, and IL-17 induce unique and overlapping transcriptional activity in HPFs. (A, B) RNA-seq of pulmonary fibroblasts with triplicate cell cultures stimulated with human LIGHT, IL-13, or IL-17A for 24 h. (A) Venn diagram of numbers of upregulated transcripts. (B) Heatmap of a select subset of upregulated transcripts. (C–G) Pathway analyses of unique LIGHT targeted gene transcripts (C), unique IL-17 transcripts (D), transcripts induced by both LIGHT and IL-13 (E), transcripts induced by both LIGHT and IL-17 (F), and transcripts common to LIGHT, IL-13, and IL-17 (G). Pathway analysis could not be performed for transcripts induced by IL-17 alone, or both IL-17 and IL-13, due to false discovery rate >0.1. GO, Gene Ontology.

Using healthy HPFs, we compared the transcriptional activity of recombinant LIGHT with that of IL-13 and IL-17 using bulk RNA-seq. Our prior studies with fibroblasts showed elimination of the activity of LIGHT by small interfering RNA knockdown of its receptor, LTβR, demonstrating specificity of the recombinant protein.^13^^,^^14^ LIGHT upregulated transcripts for 570 genes (fold change >1.5; *P* < 0.05, above phosphate-buffered saline [PBS]), compared with IL-13 that upregulated 339 gene transcripts, and IL-17A that induced 145 transcripts, when analyzed after 24 h of stimulation (Fig. 1A; Table S1). A total of 54 transcripts were common to all 3 cytokines, whereas 94 were common between LIGHT and IL-13, 41 between LIGHT and IL-17, and 14 between IL-13 and IL-17. Thus, all cytokines induced unique as well as overlapping gene targets. A sample of genes associated with inflammatory or fibrotic activity and illustrating this specificity or overlap are depicted in the heatmap in Fig. 1B, with transcript expression represented in terms of normalized *z* score. Pathway analysis for each respective group of gene transcripts was performed with the top pathways represented in enrichment plots (Fig. 1C–G; Table S2). Pathway analysis could not be performed on the 14 transcripts shared between IL-13 and IL-17, and the 36 uniquely upregulated by IL-17, due to false discovery rate values >0.1.

LIGHT induced inflammatory gene transcripts such as *CCL2*, *CSF1*, *CXCL10*, *FGF2*, *ICAM1*, *IL32*, *IL33*, and *MMP9*, which were not significantly upregulated by IL-13 or IL-17 (Fig. 1B). The pathway analysis of the 381 unique transcripts upregulated by LIGHT showed many of the genes were associated with TNF family signaling, DNA replication, inflammatory response, extracellular matrix, and cytokine-receptor cell surface interactions (Fig. 1C). IL-13 uniquely induced transcripts that included collagen isoforms (*COL7A1*), cytokine receptors (*IL17RA*, *IL18R1*, *IL1RL1*), inflammatory/fibrotic genes (*POSTN*), and G protein–coupled receptors (*PTGDR2*). Genes associated with the top pathways included those related to actin cytoskeleton, cell adhesion, extracellular matrix, and cell junctions (Fig. 1D). Although pathway analysis could not be performed on the transcripts uniquely induce by IL-17, transcripts of interest included *ACTG2*, *CXCL2*, *ELF3*, and *FGF1* (Fig. 1B). Common genes induced by both LIGHT and IL-13 included *ADAM8*, *IL13RA2*, *KIF14*, *SPHK1*, *VCAM1*, *ASPM*, *CCNB1*, *CDC20*, *CDH4*, and *CDK1* with many associated with cell cycle/cell division (Fig. 1B, E). In contrast, common genes significantly induced by LIGHT and IL-17 above PBS controls included *C3*, *CXCL1*, *CXCL5*, *CXCL6*, *CXCL8*, *IL34*, *TNFSF15*, and *TNFSF4*, with top pathways highlighted corresponding to cytokine and inflammatory responses and extracellular immune-related interactions (Fig. 1B, F). Last, transcripts that were significantly upregulated by all 3 cytokines were also largely related to cell cycle/cell division, such as *CDC6*, *CENPA*, *BUB1B*, *CCNE2*, *PRC1*, and *FAM83D*, although they included transcripts associated with inflammatory activity such as *SULF1*, *NOG*, and *PDCD1LG2* (Fig. 1B, G). Overall, these data show that LIGHT, IL-13, and IL-17 are not identical in their activities in lung fibroblasts, but all are capable of inducing molecules associated with inflammatory processes as well as cell division.

### LIGHT in combination with IL-13 or IL-17 synergizes to upregulate multiple gene transcripts in fibroblasts that are involved in cell division and inflammation

**Figure 2.**
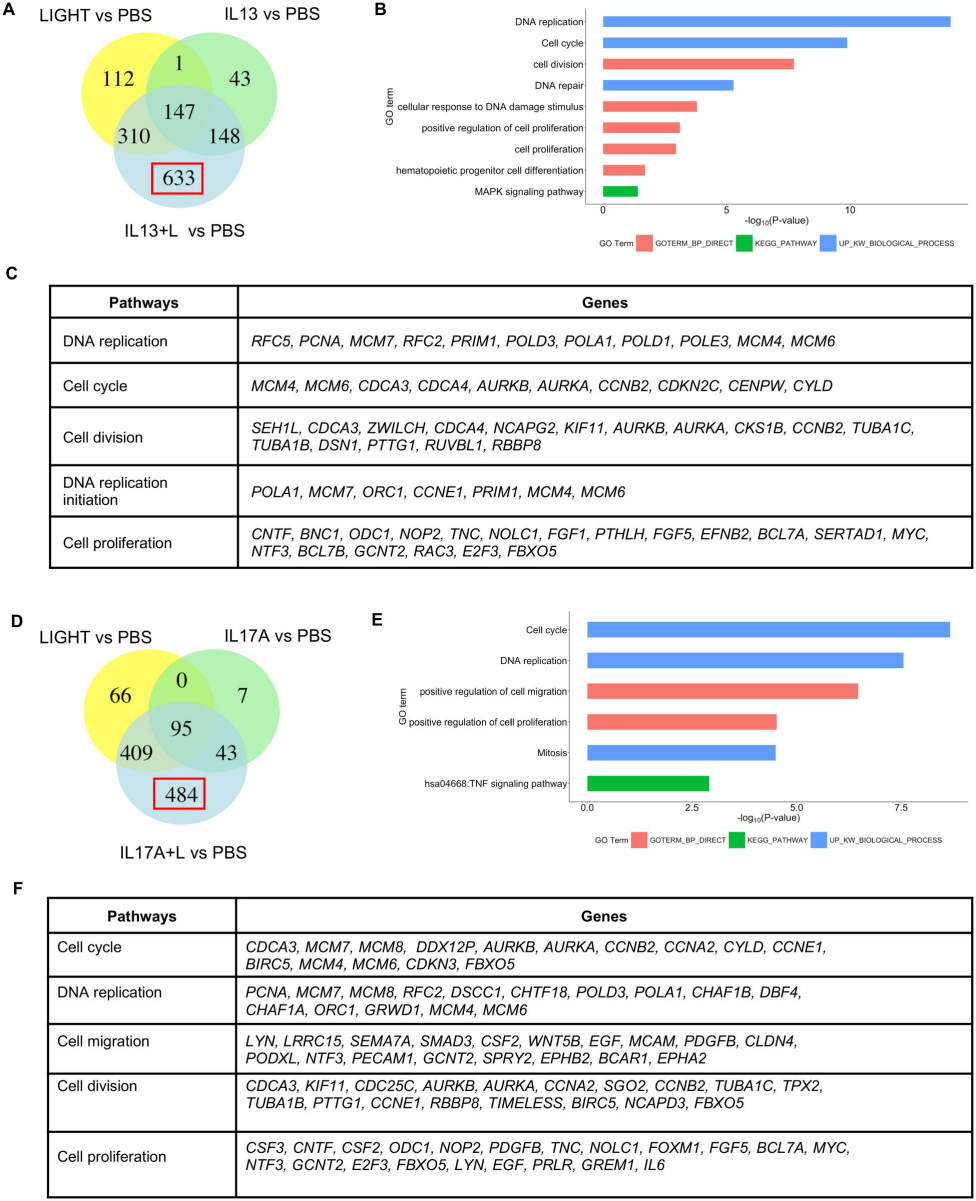
LIGHT in combination with IL-13 or IL-17 uniquely upregulate gene transcripts in pulmonary fibroblasts involved in cell division. (A–F) RNA-seq of pulmonary fibroblasts with triplicate cell cultures stimulated with LIGHT, IL-13, or LIGHT and IL-13 (A–C), or stimulated with LIGHT, IL-17A, or LIGHT and IL-17A (D–F) for 24 h. (A, D) Venn diagram of numbers of upregulated transcripts. (B, E) Pathway analysis of 633 unique LIGHT- and IL-13–induced transcripts (B), and 484 unique LIGHT- and IL-17–induced transcripts (E). (C, F) Shortlist of genes from top pathways highlighted in panels B and E. GO, Gene Ontology.

We then tested if the combination of LIGHT with IL-13 or IL-17 might further enhance or alter transcriptional signatures in lung fibroblasts. LIGHT stimulation together with IL-13 upregulated the expression of transcripts for 1238 genes. Many of these transcripts were also induced by LIGHT or IL-13 alone (310, 147, 148) including those encoding the inflammatory molecules mentioned previously, but 633 were only upregulated significantly (fold change >1.5; *P* < 0.05, above PBS) with the combination of the two cytokines (Fig. 2A, highlighted in red box; Table S3). Interestingly, enrichment analysis of these 633 transcripts revealed the top pathways were related to cell division with many transcripts from genes associated with DNA replication, cell cycle progression, and cell proliferation (Fig. 2B, C; Table S4). Inflammatory transcripts were also found in the 633 induced uniquely by the combination, including for chemokines (*CCL7*, *CXCL2*), cytokines/growth factors (*IL6*, *FGF1*), immune-related cell signaling/cell interaction molecules (*ITGA6*, *MCAM*, *PECAM1*, *TNFRSF12A*), molecules involved in antigen presentation (*CIITA*, *TAP2*), and matrix metalloproteases (*MMP11*, *MMP13*).

Similarly, LIGHT and IL-17 in combination upregulated the transcription of 1031 genes. As with IL-13, many of these transcripts were also significantly induced by LIGHT or IL-17 alone (409, 95, 43), again including those encoding the inflammatory molecules mentioned previously. However, 484 gene transcripts were also uniquely upregulated above the threshold level (fold change >1.5; *P* < 0.05, above PBS) by the combination of LIGHT and IL-17 (Fig. 2D, red highlighting; Table S5). Enrichment analysis once more showed that the top pathways were genes associated with cell cycle, DNA replication, and cell proliferation (Fig. 2E, F; Table S6). In addition, inflammatory-type transcripts were also upregulated including those linked with cell migration (e.g. *SEMA7A*, *SMAD3*, *EGF*, *PDGFB*, *PECAM1*) (Fig. 2F; Table S6) and others such as *CCL20*, *CXCL3*, *CSF2*, *CSF3*, *IL6*, *PDGFB*, *ITGA1*, *MCAM*, *PECAM1*, *TNFRSF12A*, *EPHA2*, *EPHB1*, and *EPHB2*. Thus, one of the effects of signaling from LIGHT in the presence of IL-13 or IL-17 is likely to be to increase the proliferation of fibroblasts as well as enhance inflammatory activity.

**Figure 3.**
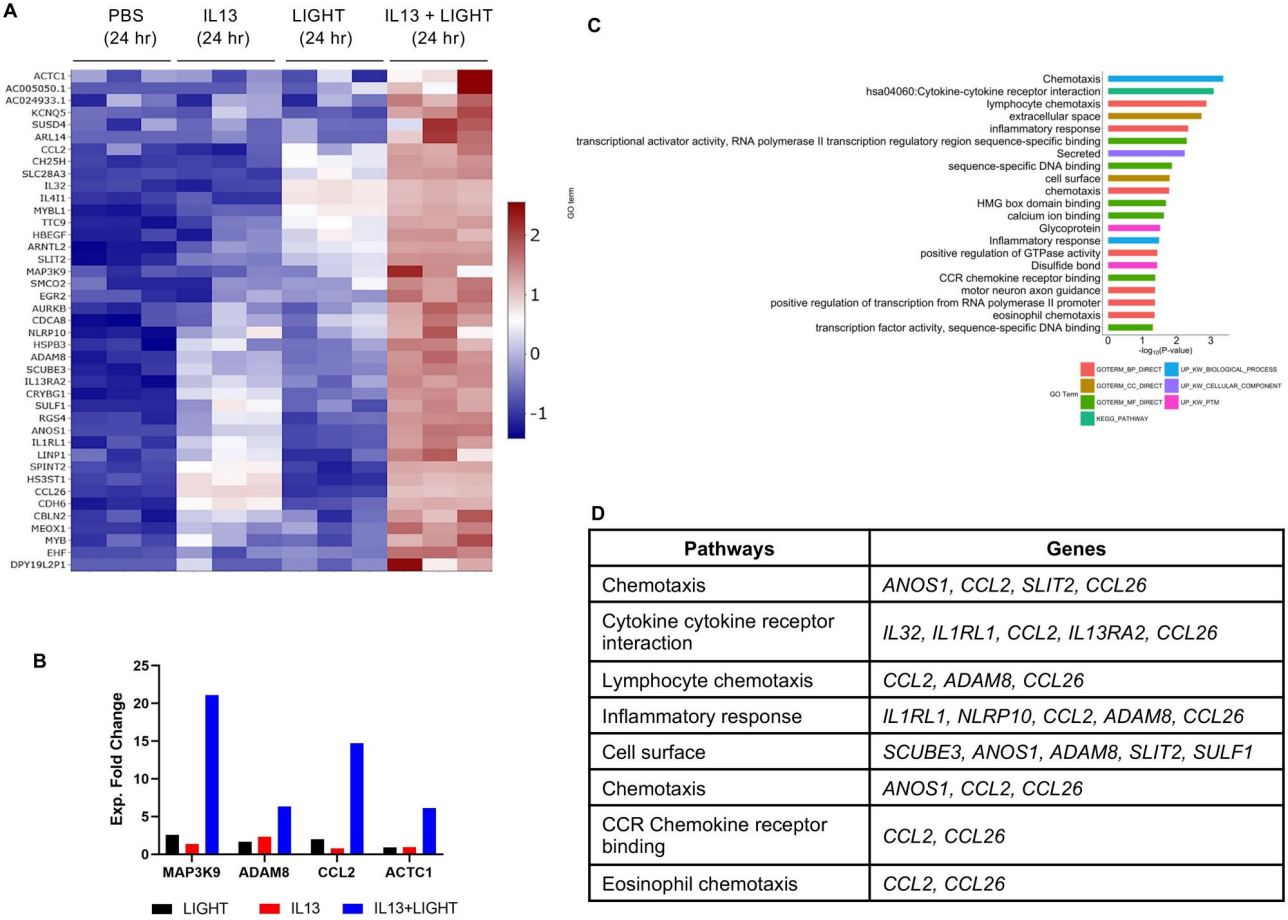
LIGHT in combination with IL-13 synergistically upregulates select inflammatory gene transcripts in pulmonary fibroblasts. RNA-seq of pulmonary fibroblasts stimulated with LIGHT, IL-13, or LIGHT and IL-13 from Fig. 2. (A) Heatmap of transcripts synergistically upregulated by the combination of LIGHT and IL-13, defined as >20% above the additive effect of each cytokine alone. (B) Experimental fold change in expression of select transcripts from panel A. (C, D) Pathway analysis of transcripts synergistically upregulated by LIGHT and IL-13 (C) and shortlist of genes from top pathways (D). GO, Gene Ontology.

In addition to this, we asked whether the combination of cytokines could act in a truly synergistic manner, potentially to enhance specific inflammatory effects. LIGHT when combined with IL-13 upregulated the expression of 41 transcripts in such a manner, defined as >20% above the additive effect of each cytokine alone. The expression profile for all of the synergistically upregulated genes is represented in terms of normalized *z* score in the heatmap in Fig. 3A, and the experimental fold change of several is shown in Fig. 3B to illustrate the synergistic action on those transcripts. Pathway analysis of these genes showed that LIGHT with IL-13 elevated the expression of transcripts related to several inflammatory activities, including chemotaxis of monocytes, T cells, dendritic cells, eosinophils, and basophils (*CCL2*, *CCL26)*; cytokine-cytokine receptor interactions, some of which are also associated with type 2 immunity (*IL32*, *IL13RA2*, *IL1RL1*); and several genes linked to more general inflammatory responses (*NLRP10*, *ADAM8*, *SCUBE3*, *SULF1*) (Fig. 3C, D; Table S7).

**Figure 4.**
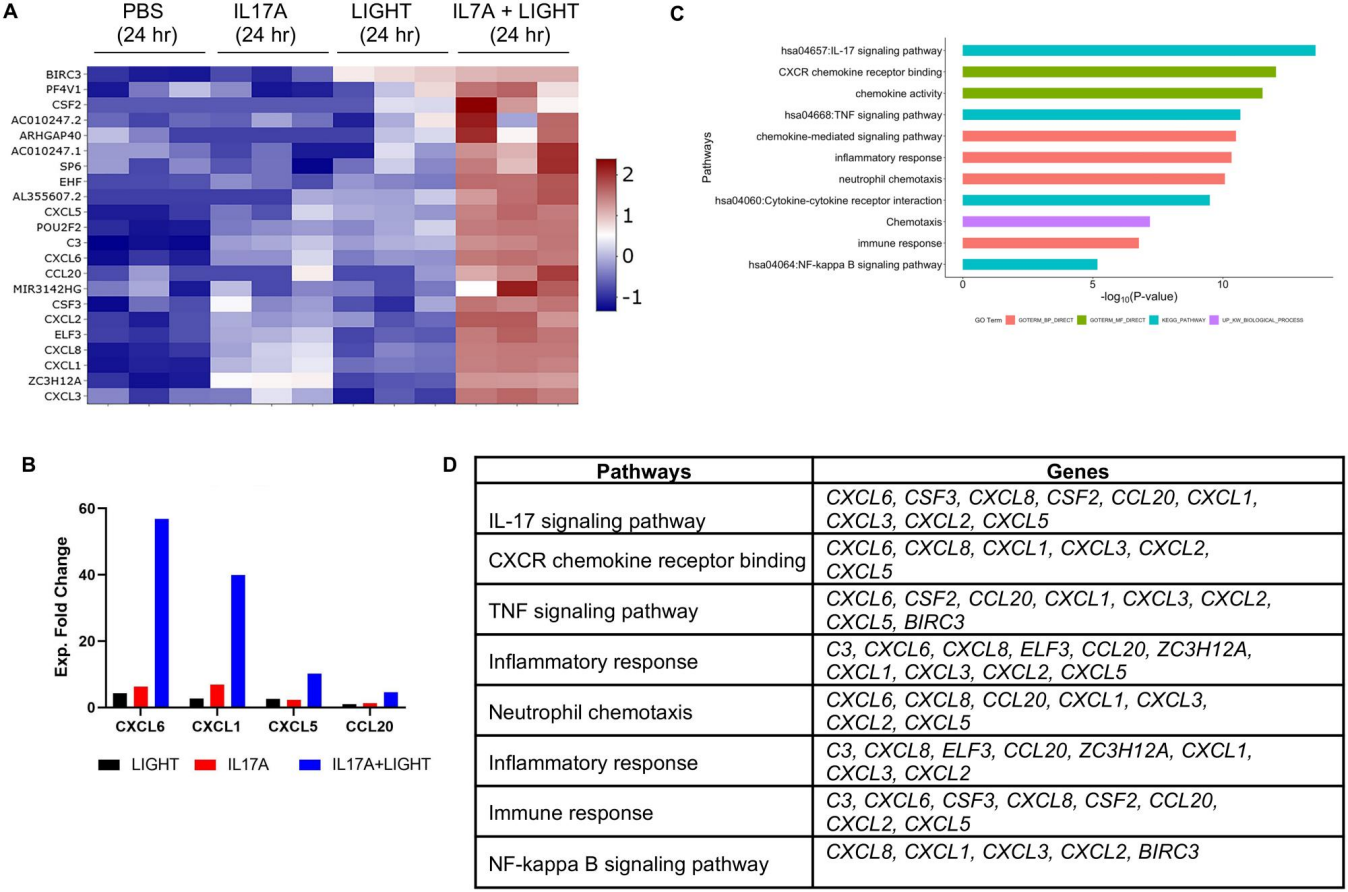
LIGHT in combination with IL-17 synergistically upregulates select inflammatory gene transcripts in pulmonary fibroblasts. RNA-seq of pulmonary fibroblasts stimulated with LIGHT, IL-17, or LIGHT and IL-17 from Fig. 2. (A) Heatmap of transcripts synergistically upregulated by the combination of LIGHT and IL-17, defined as >20% above the additive effect of each cytokine alone. (B) Experimental fold change in expression of select transcripts from A. (C, D) Pathway analysis of transcripts synergistically upregulated by LIGHT and IL-17 (C) and shortlist of genes from top pathways (D).

Similarly, LIGHT and IL-17 together synergistically upregulated the transcriptional expression of 22 genes (Fig. 4). Expression of all of these transcripts normalized with *z* score values is depicted in the heatmap in Fig. 4A, and the experimental fold change of several is shown in Fig. 4B. Pathway analysis indicated many of these were related to inflammatory processes such as chemotaxis, IL-17 and TNF signaling, and others (Fig. 4C, D; Table S8). A major category of these transcripts were for chemokines, namely *CXCL1*, *CXCL2*, *CXCL3*, *CXCL5*, *CXCL6*, *CXCL8*, and *CCL20*, that can act on several cell types, particularly neutrophils, as well as macrophages, monocytes, and T cells. LIGHT and IL-17 in combination also boosted the expression of transcripts for other inflammatory molecules such as the cellular inhibitor of apoptosis gene *BIRC3*, the complement gene *C3*, and the G-CSF and GM-CSF genes *CSF2* and *CSF3*.

### Stimulation of HPFs with IL-13 and IL-17 followed by LIGHT also leads to inflammatory transcriptional phenotypes

We additionally asked whether IL-13 and IL-17 might cooperate with LIGHT in an alternate manner and if prior exposure of pulmonary fibroblasts to these cytokines before LIGHT also drove enhanced inflammatory transcriptional phenotypes. Fibroblasts were cultured with PBS, IL-13, or IL-17 for 72 h, and then stimulated with LIGHT for a further 24 h before RNA-seq analysis (total 96 h).

**Figure 5.**
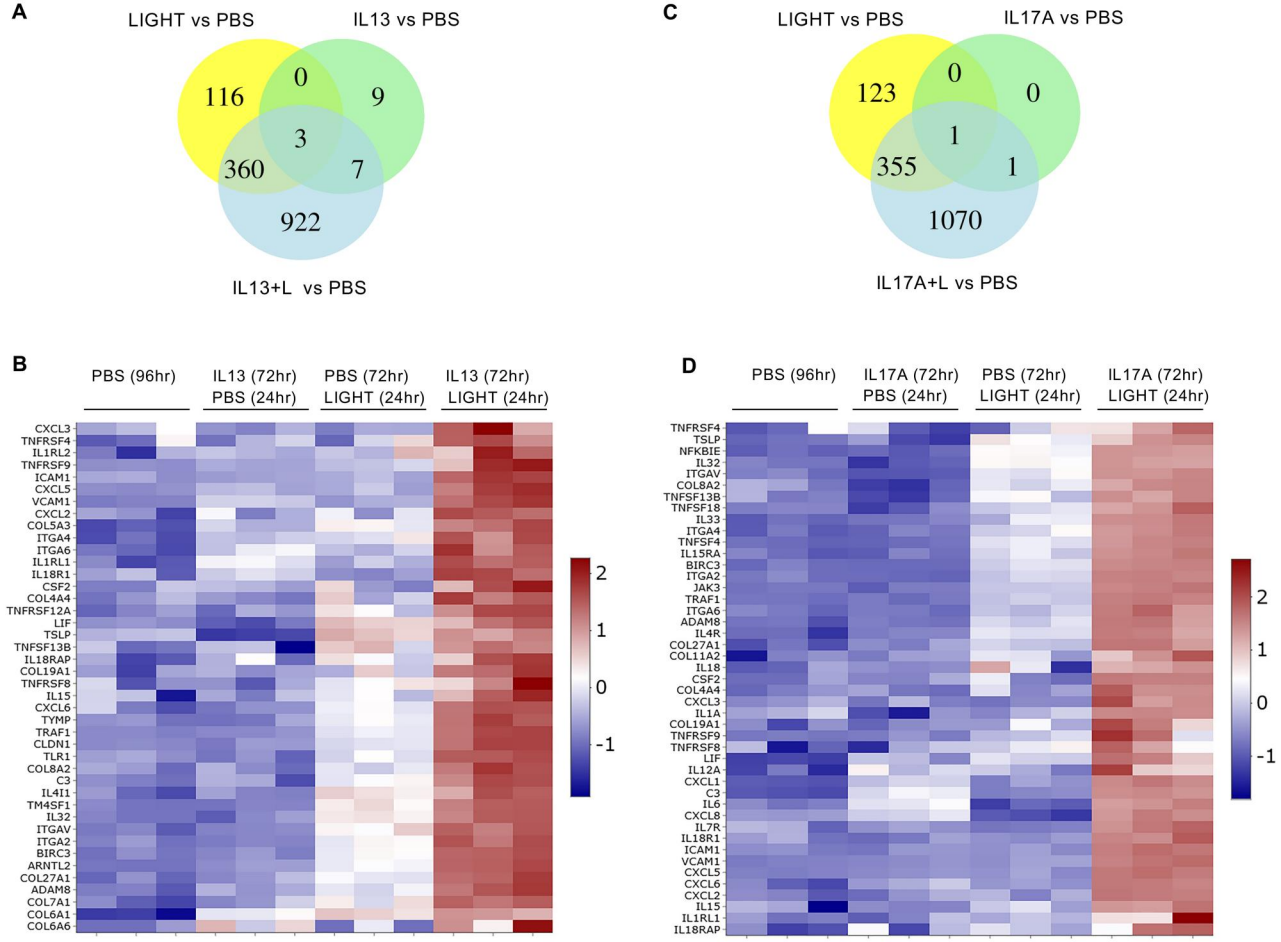
Prior exposure of pulmonary fibroblasts to IL-13 and IL-17 results in inflammatory phenotypes with temporal stimulation from LIGHT. RNA-seq of pulmonary fibroblasts cultured in triplicate with PBS, IL-13, or IL-17 for 72 h and subsequently stimulated with LIGHT for 24 h. (A) Venn diagram of numbers of upregulated transcripts induced in IL-13–pretreated cells. (B) Heatmap of select upregulated transcripts in IL-13–treated cells. (C) Venn diagram of numbers of upregulated transcripts induced in IL-17–pretreated cells. (D) Heatmap of select upregulated transcripts in IL-17–treated cells.

In control cultures (PBS), LIGHT upregulated the expression of 479 transcripts (Fig. 5A, C) similar to its activity without 72 h preculture (see Fig. 1). However, with prior exposure to IL-13 or IL-17, a far greater number of transcripts were upregulated after LIGHT stimulation (1,292 and 1,427, respectively) (Fig. 5A–D; Table S9). This was not primarily due to residual activity of IL-13 or IL-17, as only 19 and 2 transcripts, respectively were elevated at this time point in fibroblasts stimulated with these cytokines in isolation (Fig. 5A, C). Enhanced expression resulted for a number of transcripts that LIGHT, IL-13, or IL-17 alone normally induce, including inflammatory molecules such as *C3*, *CXCL3*, *CXCL6*, *CXCL8*, *ADAM8*, *ICAM1*, *IL33*, *IL32*, and *TNFSF4* (Fig. 5B, D).

Additionally, more transcripts were upregulated over those in control fibroblasts. Of the 1,292 transcripts in IL-13 prestimulated cells, 363 were upregulated after LIGHT stimulation in control cells, whereas 922 were additionally induced (Fig. 5A, B). As well as the molecules mentioned previously, these included transcripts for a number of other inflammatory factors, illustrated in the heatmap in Fig. 5B, including chemokines (*CXCL2*), cytokines (*CSF2*, *IL15*, *LIF*, *TNFSF13B*, *TSLP*), cytokine receptors (*IL18R1*, *IL18RAP*, *IL1RL1*, *IL1RL2*, *TNFRSF12A*), costimulatory molecule receptors (*TNFRSF4*, *TNFRSF8*, *TNFRSF9*), and adhesion molecules (*ITGA2*, *ITGA4*, *ITGA6*, *ITGAV*). Several collagen isoform transcripts were also upregulated in IL-13 pre-exposed cells stimulated with LIGHT (*COL4A4*, *COL5A3*, *COL6A1*, *COL6A6*, *COL7A1*, *COL8A2*, *COL19A1*, *COL27A1*). Similarly, of the 1,427 transcripts upregulated in IL-17 pre-exposed cells stimulated with LIGHT, 1,070 were not induced by LIGHT stimulation in control cells (Fig. 5C, D). The upregulated transcripts again included many of the same inflammatory molecules as in IL-13 prestimulated cells, illustrated in the heatmap in Fig. 5D, including chemokines (*CXCL2*), cytokines (*CSF2*, *LIF*, *TNFSF13B*, *TSLP*, *IL1A*, *IL6*, *IL15*, *IL18*, *IL12A*), cytokine receptors (*IL18R1*, *IL18RAP*, *IL1RL1*, *IL4R*, *IL7R*), costimulatory molecule receptors (*TNFRSF4*, *TNFRSF8*, *TNFRSF9*) and adhesion molecules (*ITGA2*, *ITGA4*, *ITGA6*, *ITGAV*), and a number of collagen isoforms also found in IL-13 pretreated fibroblasts (*COL4A4*, *COL8A2*, *COL11A2*, *COL19A1*, *COL27A1*). Pathway analyses of the total 1,292 and 1,427 transcripts upregulated in LIGHT stimulated IL-13 or IL-17 pre-exposed cells further showed that they included many genes controlling cell cycle and division (e.g. *AURKA*, *AURKB*, *MCM4*, *MCM6*, *CCNB2*, *CCNE1*), similar to fibroblasts stimulated simultaneously with LIGHT and these cytokines (see Fig. 2), as well as some genes linked to antigen processing and presentation or cell migration, and various immune molecules linked to inflammatory pathways (Table S10). Thus, in addition to acting at the same time as LIGHT, IL-13 and IL-17 can act in combination by priming fibroblasts prior to LIGHT signals to also lead to inflammatory fibroblast phenotypes.

### Gene transcripts upregulated by LIGHT, IL-13, and IL-17 are represented in defined fibroblast transcriptional clusters from the lungs of patients with ILD

Several groups have to date studied fibroblast heterogeneity in disease tissue using single-cell RNA-seq analysis. Korsunsky et al.^4^ integrated different single-cell RNA-seq datasets from 4 tissues (salivary gland, lung, intestine, synovium) from 4 human diseases (Sjogren’s syndrome, ILD, ulcerative colitis, and rheumatoid arthritis) into a multitissue atlas to understand if shared phenotypes in fibroblasts exist, and in doing so defined 14 different transcriptional profiles (clusters 0–13) across these tissues and diseases. To then attempt to relate our in vitro findings to human lung disease, we analyzed Korsunsky et al.’s transcriptional clusters defined in lung fibroblasts from patients with ILD.

**Figure 6.**
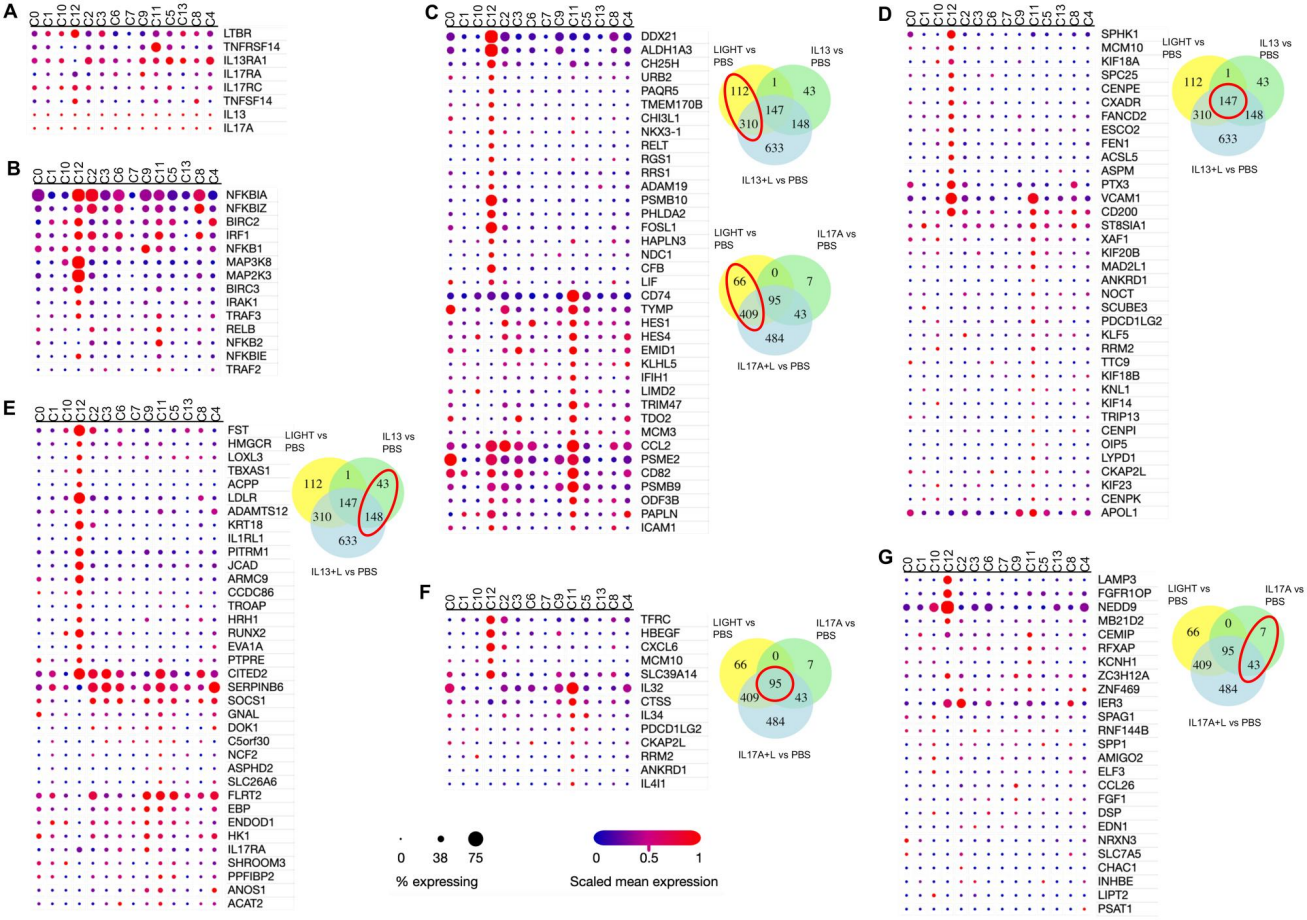
Gene transcripts upregulated by LIGHT, IL-13, and IL-17 are represented in lung fibroblast clusters from ILD. Fourteen different transcriptional profiles (clusters 0–13) from lung ILD fibroblasts, defined by Korsunsky et al.,^4^ were assessed: for gene transcripts of *LIGHT*, *IL13*, or *IL17* or their receptors (A) or for gene transcripts upregulated in HPFs associated with LIGHT signaling (B); or for other gene transcripts upregulated in healthy pulmonary fibroblasts by LIGHT alone (C), LIGHT alone shared with IL-13 alone (D), IL-13 alone (E), LIGHT alone shared with IL-17 alone (F), and IL-17 alone (G), taken from data in Fig. 2 and found in red circles of Venn diagrams. Dot plots show scaled mean expression (blue to red color) integrated with percentage of cells expressing each transcript (size of dots).

We first asked if the receptors for LIGHT, IL-13, or IL-17 were uniformly expressed across fibroblast clusters and if transcripts for any of these cytokines were also present given a prior report suggested that LIGHT might be transcribed in some fibroblasts. Although all fibroblasts are capable of expressing membrane LTβR and HVEM (TNFRSF14), and the receptors for IL-13 and IL-17, differential messenger RNA (mRNA) expression was seen. Interestingly, cluster 12 from Korsunsky et al.’s analysis of lung fibroblasts in ILD^4^ showed the strongest expression of *LTBR* and cluster 11 strongest expression of *TNFRSF14* (Fig. 6A). *IL4R* and *IL17RC* were also strongly represented in cluster 12 compared with most other clusters. Uniform expression of *IL13* and *IL17A* mRNA was seen in a small percentage of all clusters, whereas *TNFSF14* (LIGHT) mRNA was more evident in clusters 12, 11, and 8.

We next asked if the clusters displayed evidence of LIGHT receptor signaling, either alone or in combination with IL-13 or IL-17. Although signaling from LIGHT’s receptors is largely regulated by phosphorylation or ubiquitination events, LIGHT can upregulate the transcription of components of its signaling pathways, particularly molecules involved in NF-κB and MAPK pathways (shown in Fig. 6B; see Tables S1, S3, S5, and S9). Interestingly, a number of these transcripts were strongly expressed in cluster 12 from the analysis of lung fibroblasts in ILD, and to a lesser extent in cluster 11 (Fig. 6B), correlating with *LTBR* and *TNFRSF14* expression. Some of the transcripts were highly expressed in other clusters but without an obvious signature, implying that clusters 12 and 11 might have specifically been influenced by LIGHT with IL-13 or IL-17.

We then asked whether the transcriptional clusters displayed evidence of signatures of other genes that can be induced by LIGHT, IL-13, or IL-17 alone, or the combination of LIGHT with either IL-13 or IL-17, taken from data shown in the Venn diagrams in Fig. 2. LIGHT stimulation alone, or in combination with IL-13 or IL-17 (Fig. 6C, circled genes in Venn diagrams), upregulated the expression of several transcripts that were again represented primarily in clusters 11 and 12 from Korsunsky et al.’s integrated analysis of ILD lung fibroblasts.^4^ These included genes involved in metabolic processes, cytokine signaling pathways, general inflammatory responses, antigen presentation, and chemotaxis (*CHI3L1*, *ALDH1A3*, *ADAM19*, *LIF*, *CFB*, *RELT*, *RGS1*, *NDC1*, *ICAM1*, *CCL2*, *CXCL1*, *CD74*, *CD82*, *PSMB9*, *DDX21*, *PHLDA2*, *CH25H*) (Fig. 6C).

Transcripts that were upregulated by LIGHT alone as well as by IL-13 alone, and their combination (out of 147, circled), also were more strongly represented within clusters 11 and 12 of the Korsunsky et al. analysis.^4^ These included transcripts associated with inflammatory responses, cell cycle, and others (*SPHK1*, *VCAM1*, *CD200*, *MCM10*, *KIF18A*, *KIF20B*, *CENPE*, *CXADR*, *SCUBE3*, and *PDCD1LG2*) (Fig 6D). From transcripts upregulated by IL-13 alone or in combination with LIGHT (circled, Fig. 6E), a number encoding inflammatory regulatory molecules additionally were associated with ILD fibroblast cluster 12 and to an extent cluster 11, such as *FST*, *LOXL3*, *LDLR*, *ADAMTS12*, *IL1RL1*, *CITED2*, *IL17RA*, and *SERPINB6*, although several transcripts were also expressed in other ILD clusters. A few inflammatory transcripts that were upregulated by LIGHT alone, as well as IL-17 alone, and their combination (out of 95, circled), also were largely found within clusters 11 and 12, including *CXCL6*, *MCM10*, *IL32*, *IL34*, and *PDCD1LG2* (Fig. 6F). Furthermore, from transcripts upregulated by IL-17 alone or in combination with LIGHT (circled, Fig. 6G), several were also strongly expressed in ILD fibroblast cluster 12, such as *LAMP3*, *FGFR1OP*, and *NEDD9.*

Of the transcripts uniquely upregulated only by the combinations of LIGHT with IL-13 or IL-17 (633 and 484, respectively) (Fig. 2A, C), those common to both datasets that were associated with DNA replication, cell cycle, and cell proliferation were found in fibroblast clusters 11 and 12 but were also broadly expressed in additional clusters as might be expected for genes involved in cell division (Fig. S1).

**Figure 7.**
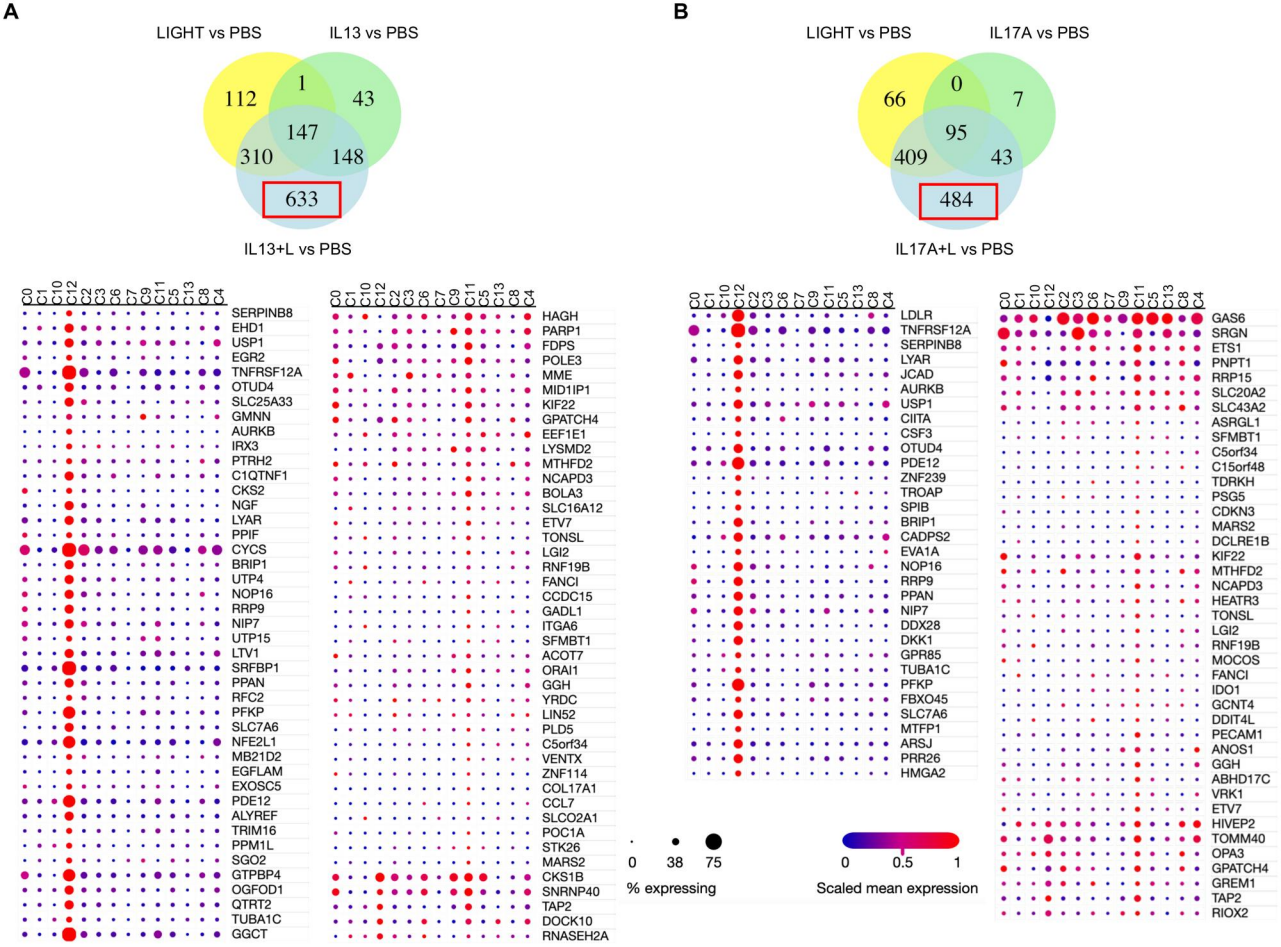
Gene transcripts upregulated by LIGHT in combination with IL-13 or IL-17 represented in lung fibroblast clusters from ILD. Fourteen different transcriptional profiles (clusters 0–13) from lung ILD fibroblasts, defined by Korsunsky et al.,^4^ were assessed for expression of gene transcripts uniquely upregulated in healthy pulmonary fibroblasts by LIGHT in combination with IL-13 (A), and LIGHT in combination with IL-17 (C), taken from data in Fig. 2 and marked in red in Venn diagrams. Dot plots show scaled mean expression (blue to red color) integrated with percentage of cells expressing each transcript (size of dots).

However, other transcripts not associated with cell division, that were only upregulated by the combination of LIGHT and IL-13 or LIGHT and IL-17, showed a more biased association with cluster 12 in particular as well as cluster 11 to a lesser extent (Fig. 7A, 7B). In the transcripts driven by LIGHT with IL-13 (Fig. 7A), these included genes linked to cytokine signaling, cytokine metabolic processes, kinase activity, stress response, antigen presentation, and ribosome biogenesis (*EGR2*, *TNFRSF12A*, *SERPINB8*, *EHD1*, *USP1*, *OTUD4*, *C1QTNF1*, *CKS2*, *NGF*, *BRIP1*, *PPIF*, *CYCS*, *UTP4*, *NOP16*, *RRP9*, *NIP7*, *UTP15*, *LTV1*, *SRFBP1*, *PPAN*, *CCL7*, *ITGA6*, *TAP2*, and others). Of the 41 transcripts that were synergistically upregulated by the combination of LIGHT with IL-13 (Fig. 3), some, such as *CCL2*, *CH25H*, *IL1RL1*, *EGR2*, *RGS4*, and *IL32*, were strongly expressed in ILD fibroblast clusters 11 or 12, whereas others were randomly expressed in alternate clusters (Fig. S2A).

Similarly, genes upregulated only by LIGHT with IL-17 that showed some association with clusters 11 and 12 also were the same genes or related to the same processes, and included *LDLR*, *TNFRSF12A*, *SERPINB8*, *CIITA*, *CSF3*, *DDX28*, *GAS6*, *ETS1*, and *TAP2* (Fig. 7B). Of the 22 transcripts that were synergistically upregulated by the combination of LIGHT with IL-17 (Fig. 4), again some, such as *C3*, *BIRC3*, *CXCL1*, *CXCL6*, and *CSF3*, were strongly expressed in ILD fibroblast clusters 12 or 11, but other transcripts were not associated with these clusters in a more biased manner (Fig. S2B).

In summary, these results show that although the total upregulated transcripts induced by LIGHT, IL-13, IL-17, or their combinations in healthy lung fibroblasts do not associate directly with any one transcriptional phenotype seen in lung fibroblasts from ILD patients, there are signatures of the activities of these cytokines, either alone or in combination, that can be found specifically in several ILD fibroblast subtypes.

## Discussion

Fibroblasts are present in all tissues, with some having specialized phenotypes and activation states related to performing specific functions in maintaining tissue architecture and tissue development, or wound healing. However, the proliferation of studies utilizing single-cell RNA-seq has revealed multiple potential differentiation states of fibroblasts defined by transcriptional signatures, and some of these include genes linked to immune cell and inflammatory activity that could be relevant for immune-mediated diseases.^4^^,^^24–35^ How these transcriptional states are induced is not clear, but they have been hypothesized to be driven in part by inflammatory cytokines. In this study, we show that 3 cytokines, IL-13, IL-17, and the TNF family molecule LIGHT, can integrate their signals to define or exaggerate these inflammatory transcriptional phenotypes, and by analyzing gene expression in lung fibroblasts from patients with ILD we find evidence of the transcriptional signatures of these cytokines alone or together in subsets of fibroblasts.

LIGHT has been linked to a number of different inflammatory diseases based on mouse models with gene-deficient animals or with blocking studies, as well as observations of its elevated expression in human tissue or fluids.^6^ LIGHT can be expressed principally by activated T cells, including T helper 2 cells that produce IL-13, and T helper 17 cells that produce IL-17, as well as other subtypes of CD4 cells and CD8 cells. Thus, it could be made in the tissues of any disease that involves T cells and could be available to stimulate and shape fibroblast activity. Moreover, LIGHT has the potential to be made by dendritic cells, macrophages, natural killer cells, neutrophils, innate lymphoid cells, and natural killer T cells, creating additional sources for its availability. Interestingly, it has also been found at the RNA level to be upregulated in some fibroblast populations in inflammatory bowel disease,^36^ and we also observed this in our current analysis of ILD fibroblasts, potentially creating a situation where a LIGHT feedback loop could be created to drive a specific fibroblast activity state.

We have published a number of studies suggesting LIGHT can be a strong contributor to lung inflammation and fibrosis, implying a potential relevance to fibroblast activity.^7–10^ Indeed, we initially showed with polymerase chain reaction and protein analyses that it could act on human lung fibroblasts in vitro to induce transcription of several inflammatory molecules, including the adhesion molecules *ICAM1* and *VCAM1*, and chemokines *CCL5*, *CXCL5*, *CXCL11*, and *CXCL12*, that are known to orchestrate infiltration and localization of immune cell populations in tissues.^13^ We furthered this concept with human esophagus fibroblasts, demonstrating with RNA-seq that these and additional inflammatory molecules could be induced by LIGHT.^14^^,^^37^ Our current data now extend this, showing again with RNA-seq that alone LIGHT has very similar transcriptional effects on human lung fibroblasts as we reported with fibroblasts from the esophagus. Moreover, we show that some of the gene targets of LIGHT are shared by IL-13 and/or IL-17, whereas some are unique. Most importantly, we find that LIGHT can cooperate and synergize with both IL-13 and IL-17 to enhance and exacerbate transcriptional phenotypes that relate to proliferation of lung fibroblasts as well as their potential to exhibit inflammatory molecules that could be relevant for several lung diseases, including ILD, idiopathic pulmonary fibrosis, chronic obstructive pulmonary disease, and asthma. Elevated expression of LIGHT or its receptors has been found in one or several of these diseases,^38–44^ as has elevated expression of IL-13 or IL-17.^45–50^ The cooperative action of LIGHT together with IL-13 or IL-17 manifested as a synergistic activity in promoting greater levels of transcription of genes such as those encoding CCL and CXCL chemokines, as well as prior exposure to either IL-13 or IL-17 leading to enhanced transcription of additional genes upon stimulation by LIGHT. The latter again included several inflammatory factors including chemokines, cytokines, cytokine receptors, and costimulatory molecules in the TNF receptor family. Moreover, prior exposure to IL-13 or IL-17 followed by LIGHT also upregulated transcription of a number of collagen isoforms. All of these activities in lung fibroblasts, if converted to enhanced protein expression, could then contribute to airway inflammation and inflammation-linked lung fibrosis.

Our analysis has limitations in being centered around three cytokines and their individual or combined transcriptional action on healthy fibroblasts in vitro. However, we provide some evidence of the potential relevance to human lung disease in finding transcriptional signatures of part of the action of all 3 cytokines alone, and in combination, primarily in 2 fibroblast clusters from patients with ILD. Pulmonary fibroblasts have been discussed as being central to pathogenesis of ILD and their crosstalk with immune cells including T cells might be important for disease progression.^1^^,^^51^^,^^52^ Unsurprisingly, all of the gene transcripts upregulated in our in vitro cultures were not represented entirely in any one fibroblast transcriptional phenotype from ILD patients. This is likely because any phenotype found in a tissue in humans is a product of the tissue environment, as well as multiple stimuli and differentiation events that can occur over a long period of time, and simplistic in vitro systems will never reproduce these signals exactly. However, the fact that we found gene signatures in ILD fibroblasts of a number of the transcripts upregulated in the cultures suggests that the in vitro stimulation conditions may mimic some of the signaling activities that shape disease-associated fibroblasts. Furthermore, as these transcriptional signatures were largely confined to 2 out of 14 fibroblast clusters, this suggests that the associations were not purely random and further reinforces the notion that LIGHT, IL-13, or IL-17 alone or in combination could be involved in driving fibroblast transcriptional states seen in patients with lung disease.

In summary, we provide evidence of the potential relevance of LIGHT, IL-13, and IL-17 action in shaping pulmonary fibroblast differentiation events that could occur in inflammatory and fibrosing diseases of the lungs. The ability of diverse cytokines from different protein families to synergize and cooperate together may be important for promoting the crosstalk between immune cells and these tissue resident structural cells that both amplifies and maintains tissue inflammation.

## Supplementary Material

vlaf042_Supplementary_Data

## Data Availability

Sequencing data generated in this study have been deposited in Gene Expression Omnibus and are available with the primary accession code GSE295622 (https://www.ncbi.nlm.nih.gov/geo/query/acc.cgi? acc=GSE295622). All the raw data associated with the manuscript are available in the supplementary file.
