# Supplementary material for: LIGHT in combination with IL-13 or IL-17 drives inflammatory transcriptional signatures in human pulmonary fibroblasts relevant for human lung disease
Source: Immunohorizons. 2025 Sep 17;9(10):vlaf042. doi: 10.1093/immhor/vlaf042 (PMC12448905; doi:10.1093/immhor/vlaf042)
Supplement: vlaf042_Supplementary_Data [file vlaf042_supplementary_data.pdf]

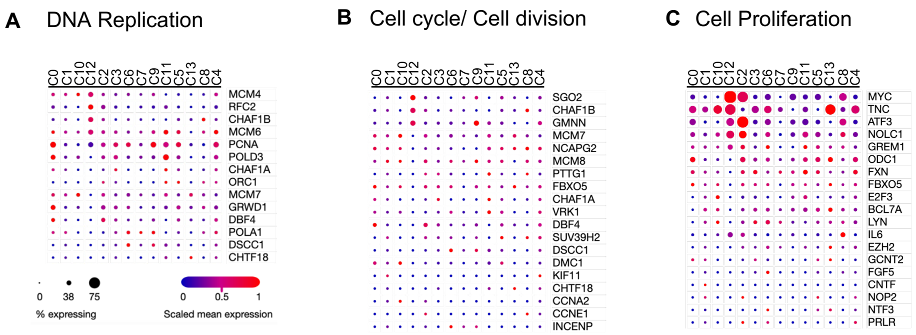

Supplementary Figure 1. Gene transcripts synergistically upregulated by LIGHT in combination with IL-13 or IL-17 represented in lung fibroblast clusters from ILD. 14 different transcriptional profiles (clusters 0-13) from lung ILD fibroblasts, defined by Korsunsky et al, were assessed for expression of overlapping gene transcripts uniquely upregulated in healthy pulmonary fibroblasts by LIGHT in combination with IL-13, and LIGHT in combination with IL-17, defined by pathway analysis as involved in DNA replication, cell cycle, and cell proliferation, taken from data in Fig. 2.

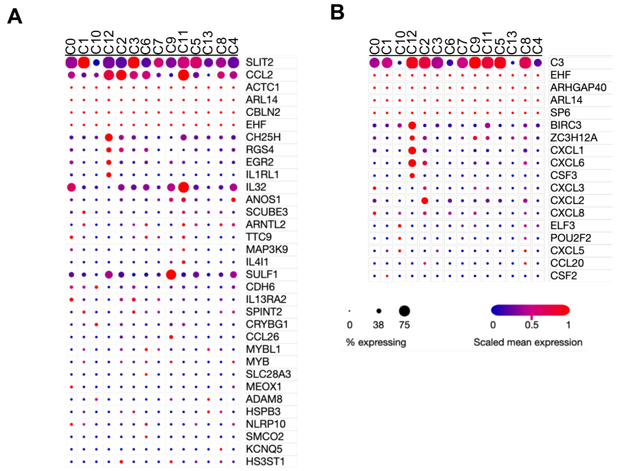

Supplementary Figure 2. Gene transcripts synergistically upregulated by LIGHT in combination with IL-13 or IL-17 represented in lung fibroblast clusters from ILD. 14 different transcriptional profiles (clusters 0-13) from lung ILD fibroblasts, defined by Korsunsky et al, were assessed for expression of gene transcripts synergistically upregulated in healthy pulmonary fibroblasts by: (**A**) LIGHT in combination with IL-13. Note, 8 of the genes were not represented in Korsunsky's dataset; and (**B**) LIGHT in combination with IL-17. Note, 4 of the genes were not represented in Korsunsky's dataset. Gene sets taken from data in Fig. 3 and 4, respectively.

Table S1. Gene transcripts upregulated by LIGHT, IL-13, and IL-17 in human pulmonary fibroblasts, associated with Fig. 1A.

Group	gene	gene_name	LIGHT.Vs.Untreated:log2FoldChangeShrunken	IL13.Vs.Untreated:log2FoldChangeShrunken	IL13_LIGHT.Vs.Untreated:log2FoldChangeShrunken	IL17A.Vs.Untreated:log2FoldChangeShrunken	IL17A_LIGHT.Vs.Untreated:log2FoldChangeShrunken	LIGHT.Vs.Untreated:padj	IL13.Vs.Untreated:padj	IL13_LIGHT.Vs.Untreated:padj	IL17A.Vs.Untreated:padj	IL17A_LIGHT.Vs.Untreated:padj
381	ENSG00000170373.8	CST1	2.96	-0.81	1.42	0.14	2.35	1.00E-53	5.00E-04	7.80E-13	0.75	7.80E-34
381	ENSG00000138135.6	CH25H	2.85	0.36	3.98	1.31	2.15	6.90E-08	0.58	5.60E-14	2	2.00E-05
381	ENSG00000105499.13	PLA2G4C	2.81	-0.74	2.35	0.14	2.83	2.90E-57	0.0065	6.20E-40	0.76	1.50E-58
381	ENSG00000100985.7	MMP9	2.62	0.22	2.78	0.26	1.91	6.90E-06	0.53	1.30E-06	0.56	1.10E-04
381	ENSG00000137033.11	IL33	2.59	-0.89	2.11	0.23	2.77	8.60E-27	0.019	3.10E-18	0.69	3.90E-31
381	ENSG00000133083.14	DCLK1	2.34	-0.08	2.8	0.39	2.35	2.80E-17	0.9	1.40E-25	0.44	1.10E-17
381	ENSG00000104998.3	IL27RA	2.33	-0.12	1.94	0.14	1.93	2.60E-66	0.69	3.20E-46	0.63	6.90E-45
381	ENSG00000006210.6	CX3CL1	2.32	-0.67	1.02	0.36	2.81	6.40E-12	0.21	0.0043	0.58	8.80E-18
381	ENSG00000197506.7	SLC28A3	2.25	-0.07	3.17	0.07	2.47	2.40E-06	0.96	1.00E-11	0.94	1.10E-07
381	ENSG00000120162.9	MOB3B	2.23	-0.58	1.59	-0.04	2.29	2.10E-45	0.012	3.10E-23	0.94	1.40E-48
381	ENSG00000146232.15	NFKBIE	2.23	-0.06	2.44	0.22	2.16	1.10E-133	0.79	1.60E-162	0.14	6.90E-125
381	ENSG00000151790.8	TDO2	2.18	-0.25	2.01	0.03	2.65	1.40E-07	0.76	4.80E-07	0.98	2.80E-11
381	ENSG00000169085.11	C8orf46	2.18	-0.2	2.06	0.09	2.2	2.90E-10	0.79	7.10E-10	0.92	6.90E-11
381	ENSG00000100906.10	NFKBIA	2.14	-0.37	1.61	0.34	1.82	7.30E-130	9.70E-04	5.00E-73	0.0036	6.60E-93
381	ENSG00000050344.8	NFE2L3	2.09	0.38	2.33	0.49	2.09	1.20E-235	3.20E-07	2.00E-298	8.60E-11	1.30E-237
381	ENSG00000152689.17	RASGRP3	2.08	-0.34	2.06	0.46	2.2	3.70E-10	0.58	9.30E-11	0.41	9.80E-12
381	ENSG00000183840.6	GPR39	2.06	0.45	2.39	0.57	2.25	2.90E-57	0.0082	2.40E-79	8.30E-04	2.10E-69
381	ENSG00000064886.13	CHI3L2	2	-0.12	2.2	0.71	2.61	1.00E-07	0.89	9.80E-10	0.19	4.60E-13
381	ENSG00000132481.6	TRIM47	1.96	0	1.88	0.33	1.97	8.30E-176	1	2.60E-162	2.50E-04	2.40E-178
381	ENSG00000104856.13	RELB	1.95	-0.24	1.98	0.45	1.98	3.50E-143	0.035	3.60E-151	4.00E-06	2.20E-149
381	ENSG00000118503.14	TNFAIP3	1.95	-0.55	1.59	0.26	1.87	3.20E-25	0.028	2.00E-17	0.45	2.00E-23
381	ENSG00000165521.15	EML5	1.93	0.42	2.29	0.23	2.49	1.00E-06	0.48	7.90E-10	0.78	3.70E-11
381	ENSG00000049249.8	TNFRSF9	1.86	0.04	2.39	0.47	1.39	9.60E-04	0.9	2.20E-05	0.45	0.0065
381	ENSG00000077150.18	NFKB2	1.86	-0.01	2.04	0.26	2.06	1.00E-161	0.96	2.70E-197	0.0073	9.50E-200
381	ENSG00000112299.7	VNN1	1.83	-0.21	0.97	0.39	2.28	0.0037	0.76	0.04	2	4.80E-04
381	ENSG00000090104.11	RGS1	1.76	0.76	2.13	-0.02	1.65	0.0058	0.2	7.60E-04	0.99	0.0059
381	ENSG00000058085.14	LAMC2	1.73	-0.52	0.71	0.12	1.81	5.60E-26	0.017	4.60E-05	0.76	9.80E-29
381	ENSG00000090339.8	ICAM1	1.68	-0.52	0.97	0.35	1.55	7.20E-45	0.0012	4.50E-15	0.05	5.90E-38
381	ENSG00000025708.13	TYMP	1.64	0.08	1.26	0.34	1.63	1.40E-35	0.78	3.60E-21	0.098	3.20E-35
381	ENSG00000149968.11	MMP3	1.64	-0.18	-0.11	0.44	1.2	0.0095	0.82	0.93	2	0.038
381	ENSG00000166342.18	NETO1	1.64	-0.37	1.5	0.05	1.66	4.90E-04	0.62	8.50E-04	0.96	2.40E-04
381	ENSG00000169436.16	COL22A1	1.64	-0.34	1.38	0.09	1.64	4.30E-19	0.28	3.30E-14	0.85	1.50E-19
381	ENSG00000163545.8	NUAK2	1.63	0.01	1.37	0.09	1.16	4.70E-37	0.96	1.70E-26	0.77	1.10E-18
381	ENSG00000110328.5	GALNT18	1.62	0.18	1.43	-0.23	1.34	9.90E-11	0.7	4.70E-09	0.67	8.10E-08
381	ENSG00000120337.8	TNFSF18	1.6	0.03	1.06	0.59	1.55	7.10E-08	0.96	3.50E-04	0.18	9.10E-08
381	ENSG00000105877.17	DNAH11	1.57	0.12	1.91	0.25	1.87	6.30E-08	0.84	3.20E-12	0.67	1.70E-11
381	ENSG00000119714.10	GPR68	1.57	-0.28	1.32	0.09	2.04	4.00E-11	0.54	1.50E-08	0.88	1.10E-19
381	ENSG00000275993.2	CU639417.1	1.55	0.41	1.07	0.03	1.11	1.70E-04	0.48	0.0075	0.97	0.0068
381	ENSG00000085117.11	CD82	1.54	-0.83	1	0.54	1.85	2.20E-100	2.10E-25	5.80E-42	2.90E-11	2.50E-147
381	ENSG00000129667.12	RHBDF2	1.53	0.23	1.76	0.29	1.59	1.00E-46	0.14	2.70E-64	0.067	3.40E-51
381	ENSG00000133048.12	CHI3L1	1.49	-0.34	0.14	0.06	1.09	0.0031	0.67	0.84	0.96	0.032
381	ENSG00000197859.9	ADAMTSL2	1.49	0.09	0.41	0.72	0.81	0.014	0.82	0.28	2	0.092
381	ENSG00000279118.1	AC093535.2	1.49	0.9	1.21	0.84	1.58	0.0033	0.1	0.0099	0.17	0.0012
381	ENSG00000158315.10	RHBDL2	1.47	0.67	2.1	0.05	1.56	1.40E-04	0.17	2.70E-09	0.95	2.30E-05
381	ENSG00000185101.12	ANO9	1.47	-0.12	1.3	-0.19	1.54	0.0047	0.78	0.0059	0.58	0.0024
381	ENSG00000237499.6	AL357060.2	1.45	0	1.36	0.61	1.17	0.0043	0.99	0.0045	2	0.018
381	ENSG00000131323.14	TRAF3	1.42	0.04	1.5	0.38	1.64	1.70E-110	0.77	9.60E-127	4.50E-07	3.90E-150
381	ENSG00000135636.13	DYSF	1.42	0.41	0.41	0.6	1.16	0.017	0.6	0.48	0.46	0.042
381	ENSG00000170873.18	MTSS1	1.41	-0.54	0.55	0.33	1.52	1.00E-53	3.00E-06	2.70E-08	0.01	2.70E-63
381	ENSG00000137266.14	SLC22A23	1.4	-0.46	-0.21	-0.55	1.39	0.0012	0.5	0.75	0.44	8.30E-04
381	ENSG00000123095.5	BHLHE41	1.39	-0.04	1.16	0.44	1.62	2.30E-10	0.94	6.60E-08	0.18	1.10E-14
381	ENSG00000172901.19	LVRN	1.39	0.02	1.07	0.14	1.48	4.00E-04	0.97	0.0052	2	7.80E-05
381	ENSG00000198759.11	EGFL6	1.38	0.56	1.29	0.72	0.45	0.0056	0.19	0.0045	2	0.22
381	ENSG00000006432.15	MAP3K9	1.37	0.45	2.46	0.7	1.14	0.003	0.49	3.00E-09	2	0.011
381	ENSG00000138448.11	ITGAV	1.37	-0.15	1.29	0.13	1.36	2.30E-88	0.11	8.90E-79	0.22	2.40E-88
381	ENSG00000168685.14	IL7R	1.37	0.1	1.2	0.58	1.85	1.70E-41	0.58	2.10E-32	8.10E-07	7.50E-77
381	ENSG00000240476.1	LINC00973	1.37	0.06	1.82	0.09	1.95	6.40E-06	0.93	1.10E-10	0.91	6.40E-12
381	ENSG00000100767.15	PAPLN	1.34	-0.13	0.96	0.22	1.32	7.30E-15	0.67	2.40E-08	0.48	8.80E-15
381	ENSG00000250657.1	AC097451.1	1.34	-0.58	0.7	0.1	1.55	2.50E-27	1.50E-04	6.30E-08	0.73	3.90E-37
381	ENSG00000067798.14	NAV3	1.33	0.19	1.75	0.54	1.85	8.20E-33	0.3	3.70E-59	9.00E-05	4.10E-65
381	ENSG00000171617.13	ENC1	1.32	-0.03	0.78	0.21	1.26	5.80E-38	0.88	1.70E-13	0.22	9.00E-35
381	ENSG00000128578.9	STRIP2	1.31	0.55	1.59	0.38	1.39	5.50E-05	0.21	1.60E-07	0.5	8.80E-06
381	ENSG00000148488.15	ST8SIA6	1.31	0.72	1.76	0.16	1.07	0.0019	0.17	4.80E-06	2	0.011
381	ENSG00000172817.3	CYP7B1	1.31	0.42	0.36	0.61	0.84	0.0078	0.32	0.25	2	0.039
381	ENSG00000128408.8	RIBC2	1.3	0.4	1.28	0.66	1.32	0.014	0.6	0.0098	2	0.009
381	ENSG00000211448.11	DIO2	1.3	-0.3	0.26	0.26	1.68	8.80E-04	0.66	0.6	0.74	3.60E-06
381	ENSG00000128422.15	KRT17	1.29	-0.43	0.75	0.14	0.88	0.0024	0.53	0.09	0.88	0.043
381	ENSG00000169136.10	ATF5	1.29	-0.05	1.66	0.58	1.61	1.60E-65	0.74	6.70E-112	1.30E-11	2.90E-103
381	ENSG00000056558.10	TRAF1	1.28	-0.03	1.98	0.07	1.58	2.40E-27	0.91	8.90E-68	0.81	1.60E-42
381	ENSG00000115919.14	KYNU	1.28	-0.38	0.9	0	1.47	2.90E-09	0.27	3.30E-05	1	1.50E-12
381	ENSG00000131378.13	RFTN1	1.28	-0.12	1.2	0.35	1.47	8.00E-69	0.29	3.40E-61	1.20E-04	4.30E-91
381	ENSG00000127423.10	AUNIP	1.27	0.49	1.87	0.46	1.39	0.0058	0.43	6.30E-06	0.54	0.0014
381	ENSG00000136367.13	ZFHX2	1.27	-0.35	1.21	0.21	1.28	1.20E-04	0.54	1.30E-04	0.76	5.80E-05
381	ENSG00000169891.17	REPS2	1.27	0.49	1.14	0.18	1.51	1.30E-05	0.22	5.90E-05	0.77	5.30E-08

381	ENSG00000113722.16	CDX1	1.26	0.16	1.68	0.52	1.88	5.00E-04	0.8	3.20E-07	0.34	1.50E-08
381	ENSG00000204385.10	SLC44A4	1.25	-0.12	1.62	-0.18	1.36	0.017	0.9	6.30E-04	2	0.0058
381	ENSG00000160161.9	CILP2	1.24	0.11	0.68	-0.41	0.61	5.90E-06	0.84	0.019	0.37	0.041
381	ENSG00000270607.1	AC009549.1	1.24	-0.89	0.97	0.26	1.02	3.50E-07	0.0059	5.10E-05	0.58	2.90E-05
381	ENSG00000115267.5	IFIH1	1.23	0.22	1.32	0.42	1.4	5.90E-08	0.57	7.50E-10	0.23	1.10E-10
381	ENSG00000141682.11	PMAIP1	1.23	-0.01	1.11	0.36	1.5	1.10E-35	0.95	8.60E-30	0.0061	2.50E-54
381	ENSG00000266010.1	GATA6-AS1	1.23	0.78	1.26	0.65	1.16	0.0011	0.079	3.20E-04	0.21	0.0014
381	ENSG00000185947.14	ZNF267	1.22	-0.17	0.96	-0.18	0.81	1.30E-34	0.31	2.90E-22	0.3	2.90E-15
381	ENSG00000198910.12	L1CAM	1.22	-0.04	0.75	0.2	1.1	1.70E-15	0.91	1.60E-06	0.5	5.80E-13
381	ENSG00000119139.17	TJP2	1.21	0.24	1.43	0.33	1.36	4.90E-66	0.0081	6.90E-94	1.80E-04	1.70E-83
381	ENSG00000134070.4	IRAK2	1.21	0.11	1.32	0.53	1.37	1.10E-22	0.63	2.10E-28	6.90E-04	3.20E-30
381	ENSG00000170745.11	KCNS3	1.21	1.12	1.08	1.08	1.26	0.029	0.061	0.036	2	0.016
381	ENSG00000125355.15	TMEM255A	1.2	-0.05	1.12	0.54	1.64	9.90E-04	0.96	0.0013	0.32	1.10E-06
381	ENSG00000006062.14	MAP3K14	1.19	0.43	1.53	0.01	1.2	7.20E-23	0.0026	3.50E-39	0.99	7.70E-24
381	ENSG00000006118.14	TMEM132A	1.19	0.11	1.44	0.18	1.43	4.50E-40	0.48	1.70E-59	0.2	1.20E-58
381	ENSG00000075702.16	WDR62	1.19	0.52	1.91	0.42	1.41	1.50E-07	0.068	4.20E-20	0.22	7.10E-11
381	ENSG00000080709.14	KCNN2	1.18	0.12	1.03	0.32	1.14	2.60E-04	0.84	9.90E-04	0.58	2.90E-04
381	ENSG00000132003.9	ZSWIM4	1.18	0.18	1.5	0.43	1.37	3.40E-30	0.27	1.30E-50	0.0011	4.10E-41
381	ENSG00000179862.6	CITED4	1.18	0.09	1.37	0.33	1.27	1.60E-15	0.75	2.50E-22	0.14	1.60E-18
381	ENSG00000062282.14	DGAT2	1.17	-0.11	1.25	0.18	1.36	3.00E-12	0.75	4.70E-15	0.6	3.00E-17
381	ENSG00000198075.9	SULT1C4	1.15	-0.63	1.05	-0.11	0.98	7.70E-16	3.80E-04	4.30E-14	0.75	6.70E-12
381	ENSG00000113645.14	WWC1	1.14	0.11	1.92	0.33	2.05	5.10E-11	0.74	2.10E-34	0.22	1.10E-38
381	ENSG00000129173.12	E2F8	1.13	0.78	2.14	0.19	1.43	0.0043	0.086	6.20E-10	0.81	8.80E-05
381	ENSG00000156218.12	ADAMTSL3	1.13	-0.82	-0.92	-0.4	0.79	0.017	0.18	0.067	2	0.1
381	ENSG00000177989.13	ODF3B	1.13	-0.46	0.36	0.39	1.16	0.0046	0.45	0.41	0.56	0.0021
381	ENSG00000007968.6	E2F2	1.1	0.68	2.05	0.85	2.06	0.0043	0.14	9.50E-10	0.073	1.10E-09
381	ENSG00000175592.8	FOSL1	1.1	0.58	1.79	0.37	1.47	1.70E-23	1.80E-06	2.90E-63	0.0094	3.10E-42
381	ENSG00000183049.12	CAMK1D	1.1	0.08	1.22	0.58	1.09	3.80E-09	0.83	4.70E-12	0.017	2.90E-09
381	ENSG00000135678.11	CPM	1.09	-0.62	0.62	-0.09	0.95	3.20E-17	4.70E-05	2.90E-06	0.77	1.80E-13
381	ENSG00000168394.10	TAP1	1.09	0.2	1.13	0.15	1.01	1.70E-25	0.19	2.90E-28	0.43	7.20E-22
381	ENSG00000178226.10	PRSS36	1.09	-0.46	1.35	0.05	1.17	0.0062	0.45	2.00E-04	0.95	0.0018
381	ENSG00000128262.8	POM121L9	1.07	0.28	0.79	-0.07	1.08	4.10E-06	0.44	7.10E-04	0.91	1.50E-06
381	ENSG00000169245.5	CXCL10	1.07	-0.24	-0.21	0.05	0.75	0.025	0.68	0.68	0.96	0.1
381	ENSG00000111863.12	ADTRP	1.06	0.17	0.52	0.65	1.26	0.0044	0.79	0.19	0.19	2.50E-04
381	ENSG00000164690.7	SHH	1.06	-0.11	-0.05	0.06	0.77	0.014	0.89	0.93	2	0.08
381	ENSG00000253616.5	AC107959.3	1.05	-0.68	-0.14	-0.26	1.01	0.026	0.28	0.83	2	0.026
381	ENSG00000085563.14	ABCB1	1.04	-0.05	0.67	0.46	0.58	3.60E-04	0.94	0.023	0.29	0.063
381	ENSG00000162493.16	PDPN	1.04	-0.48	0.2	0.81	1.62	0.017	0.44	0.65	0.13	3.60E-05
381	ENSG00000165891.15	E2F7	1.03	0.46	1.58	0.24	1.22	5.70E-11	0.014	8.20E-27	0.35	5.90E-16
381	ENSG00000184599.13	FAM19A3	1.03	-0.21	0	0.56	0.3	0.029	0.8	0.99	2	0.6
381	ENSG00000147576.15	ADHFE1	1.02	0.28	0.97	0.29	1.04	5.50E-09	0.28	7.30E-09	0.3	9.20E-10
381	ENSG00000165490.12	DDIAS	1.02	0.6	1.39	0.1	1.24	3.00E-04	0.076	3.70E-08	0.87	2.00E-06
381	ENSG00000188290.10	HES4	1.02	0.09	0.54	0.43	0.24	7.60E-05	0.86	0.046	0.26	0.47
381	ENSG00000125347.13	IRF1	1.01	0.51	1.27	0.09	1.01	1.20E-27	7.30E-07	6.60E-45	0.63	3.80E-28
381	ENSG00000135525.18	MAP7	1.01	0.2	0.68	0.42	0.84	0.0086	0.77	0.08	0.49	0.027
381	ENSG00000135919.12	SERPINE2	1.01	-0.05	0.29	0.27	1.14	8.30E-35	0.74	0.0014	0.013	3.20E-45
381	ENSG00000206712.1	RNU6-26P	1.01	0.4	1.35	-0.34	0.94	0.0085	0.47	9.30E-05	0.63	0.011
381	ENSG00000280649.2	AC245100.8	1.01	-0.15	0.45	0.17	0.67	3.90E-04	0.79	0.15	0.77	0.024
381	ENSG00000108691.9	CCL2	1	-0.37	1.88	0.49	1.95	0.024	0.56	2.70E-06	0.43	1.60E-06
381	ENSG00000128284.19	APOL3	1	0.37	1.23	0.33	1.06	1.80E-06	0.19	3.00E-10	0.33	1.40E-07
381	ENSG00000184254.16	ALDH1A3	1	-0.45	0.11	0.16	1.07	3.30E-14	0.0044	0.54	0.5	1.10E-16
381	ENSG00000144583.4	4-Mar	0.99	0.54	1.49	0.36	1.29	6.40E-09	0.0053	6.30E-21	0.14	1.50E-15
381	ENSG00000181938.13	GINS3	0.98	0.53	1.63	0.56	1.38	7.50E-05	0.083	1.60E-13	0.091	1.60E-09
381	ENSG00000188167.8	TMPPE	0.98	0.3	0.54	0.55	0.34	0.0033	0.56	0.13	0.24	0.39
381	ENSG00000015133.18	CCDC88C	0.97	0.26	0.79	0.42	1.08	0.0066	0.65	0.021	0.45	0.0011
381	ENSG00000028137.18	TNFRSF1B	0.97	-0.01	0.87	0.09	1.06	1.10E-52	0.93	3.30E-43	0.43	1.60E-63
381	ENSG00000100055.20	CYTH4	0.97	0.16	1.02	0.34	0.68	0.0044	0.8	0.0013	0.56	0.052
381	ENSG00000122420.9	PTGFR	0.97	-0.34	0.19	-0.23	0.84	3.80E-12	0.08	0.27	0.35	1.20E-09
381	ENSG00000128342.4	LIF	0.97	-0.57	0.25	0.32	1.4	8.00E-38	1.20E-11	0.0031	6.30E-04	2.10E-81
381	ENSG00000130590.13	SAMD10	0.97	0.56	1.23	0.49	1.05	1.40E-07	0.0082	4.10E-13	0.043	2.70E-09
381	ENSG00000110660.14	SLC35F2	0.96	0.29	1.43	0.28	1.11	1.50E-22	0.017	1.00E-52	0.043	5.90E-31
381	ENSG00000257219.5	LINC02407	0.96	0.3	1.44	0.33	0.95	0.0031	0.54	5.60E-07	0.54	0.0021
381	ENSG00000271857.1	AL096865.1	0.96	0.72	0.91	0.09	0.74	0.017	0.12	0.018	0.92	0.064
381	ENSG00000003989.17	SLC7A2	0.95	-0.08	0.73	0.09	1.37	8.50E-49	0.46	4.30E-29	0.44	1.60E-105
381	ENSG00000082293.12	COL19A1	0.95	-0.64	0.42	-0.49	1.14	0.0012	0.11	0.19	0.3	2.80E-05
381	ENSG00000105639.18	JAK3	0.95	-0.46	0.08	0.07	0.62	5.50E-09	0.024	0.72	0.84	2.10E-04
381	ENSG00000141448.8	GATA6	0.95	0.35	1.2	0.52	1.36	1.60E-14	0.026	1.30E-24	5.50E-04	5.10E-31
381	ENSG00000185347.17	C14orf80	0.95	0.5	1.35	0.5	1.31	8.60E-06	0.052	3.10E-12	0.084	3.70E-11
381	ENSG00000204934.10	ATP6V0E2-A	0.95	-0.05	0.78	0.66	1.21	0.0027	0.94	0.011	0.1	3.40E-05
381	ENSG00000079931.14	MOXD1	0.94	-0.51	0.46	0.27	0.98	4.60E-24	1.50E-06	2.40E-06	0.037	1.70E-26
381	ENSG00000100479.12	POLE2	0.94	0.43	1.38	0.15	1.24	1.70E-05	0.13	3.30E-12	0.76	1.50E-09
381	ENSG00000144959.9	NCEH1	0.94	0.37	1.45	0.26	1.17	3.80E-09	0.067	9.10E-23	0.32	1.20E-14
381	ENSG00000189056.13	RELN	0.94	0.31	0.56	0.57	1.47	1.00E-07	0.21	0.002	0.0082	1.20E-19
381	ENSG00000259834.1	AL365361.1	0.94	-0.31	0.62	0.36	1.02	0.00093	0.5	0.031	0.43	1.50E-04
381	ENSG00000281571.2	AC241585.3	0.94	-0.39	0.6	-0.1	0.75	0.012	0.5	0.11	0.91	0.043
381	ENSG00000057019.15	DCBLD2	0.93	0.32	1.72	0.09	1.12	5.00E-68	3.90E-08	2.50E-239	0.34	9.60E-100
381	ENSG00000167034.9	NKX3-1	0.93	-0.21	1.02	0.33	0.85	0.0095	0.75	0.0016	0.59	0.014
381	ENSG00000069399.14	BCL3	0.92	0.03	0.44	0.48	1.11	6.60E-19	0.9	4.70E-05	8.20E-05	2.10E-28
381	ENSG00000127191.17	TRAF2	0.92	-0.06	1.14	0.01	0.93	8.20E-24	0.75	5.00E-39	0.98	5.30E-25
381	ENSG00000184371.13	CSF1	0.92	-0.1	0.9	0.19	1.26	1.70E-29	0.45	4.70E-29	0.12	3.80E-56

381	ENSG00000260822.1	AC004656.	0.92	0.69	1.16	0.57	1.24	0.048	0.21	0.0048	2	0.0028
381	ENSG00000082482.13	KCNK2	0.91	-0.87	0.02	0	0.51	2.70E-14	7.60E-10	0.93	0.99	4.60E-05
381	ENSG00000120539.14	MASTL	0.91	0.5	1.28	0.37	1.19	3.20E-14	2.30E-04	4.60E-30	0.021	3.80E-25
381	ENSG00000148344.10	PTGES	0.91	-0.54	0.28	-0.04	0.81	4.10E-10	0.0019	0.096	0.91	2.10E-08
381	ENSG00000179041.3	RRS1	0.91	0.46	1.59	0.52	1.27	1.20E-12	0.0017	7.70E-40	7.30E-04	3.10E-25
381	ENSG00000225131.2	PSME2P2	0.91	0.17	1.43	0.06	0.19	0.037	0.81	1.50E-04	0.95	0.73
381	ENSG00000248429.5	AC098679.	0.91	-0.31	0.5	0.09	0.58	0.017	0.61	0.22	2	0.15
381	ENSG00000112096.16	SOD2	0.9	-0.28	0.12	0.43	1.69	2.60E-09	0.17	0.57	0.027	2.40E-32
381	ENSG00000162073.13	PAQR4	0.9	0.29	1.41	0.46	1.23	2.10E-11	0.1	1.60E-29	0.0055	4.90E-22
381	ENSG00000227036.6	LINC00511	0.9	-0.56	0.8	-0.05	1.17	2.90E-06	0.022	1.90E-05	0.92	1.10E-10
381	ENSG00000111341.9	MGP	0.89	-1.01	-0.31	0.11	0.64	4.90E-04	0.0016	0.35	0.84	0.013
381	ENSG00000114315.3	HES1	0.89	-0.63	0.51	0.19	0.69	0.028	0.25	0.22	0.81	0.085
381	ENSG00000145860.11	RNF145	0.89	0.06	0.71	0.13	0.86	6.80E-66	0.47	4.50E-43	0.093	1.10E-61
381	ENSG00000258315.5	C17orf49	0.89	0.26	0.67	-0.21	0.5	0.0072	0.63	0.041	0.75	0.16
381	ENSG00000187741.14	FANCA	0.88	0.58	1.77	0.52	1.52	2.80E-07	0.0027	6.90E-30	0.015	1.10E-21
381	ENSG00000144354.13	CDCA7	0.87	0.47	1.44	0.33	1.32	2.50E-08	0.01	4.10E-23	0.14	4.00E-19
381	ENSG00000160013.8	PTGIR	0.87	0.07	0.66	-0.03	0.55	6.40E-09	0.81	9.20E-06	0.93	3.70E-04
381	ENSG00000206341.7	HLA-H	0.87	-0.19	0.69	0.2	0.89	9.10E-12	0.35	4.20E-08	0.37	6.90E-13
381	ENSG00000127533.3	F2RL3	0.86	0.26	0.78	0.53	0.91	0.037	0.68	0.044	0.35	0.017
381	ENSG00000139618.14	BRCA2	0.86	0.55	1.39	0.31	1.22	2.30E-06	0.0078	2.20E-17	0.26	5.60E-13
381	ENSG00000145623.12	OSMR	0.86	0.07	0.47	0.19	0.85	1.20E-40	0.56	1.20E-12	0.035	1.50E-39
381	ENSG00000178860.8	MSC	0.86	-0.53	0.26	0.39	1.03	4.10E-11	8.40E-04	0.089	0.024	3.30E-16
381	ENSG00000182963.9	GJC1	0.86	0.2	1.07	0.19	1.06	1.20E-35	0.023	1.70E-56	0.05	4.00E-55
381	ENSG00000258824.2	AL122035.	0.86	0.76	0.58	0.7	0.86	0.04	0.1	0.17	2	0.03
381	ENSG00000127124.14	HIVEP3	0.85	0.43	1.73	0.54	1.47	7.10E-10	0.0068	2.70E-43	8.00E-04	6.00E-31
381	ENSG00000131351.14	HAUS8	0.85	0.51	1.22	0.23	0.91	6.40E-05	0.043	2.90E-10	0.53	7.70E-06
381	ENSG00000159708.17	LRRC36	0.85	0.37	0.74	0.26	0.78	0.028	0.5	0.045	2	0.036
381	ENSG00000198805.11	PNP	0.85	0.51	1.51	0.26	1.16	1.20E-06	0.013	6.20E-21	0.35	2.80E-12
381	ENSG00000268812.3	AC004264.	0.85	-0.68	-0.16	-0.11	1.14	0.038	0.2	0.77	2	0.0019
381	ENSG00000125845.6	BMP2	0.84	-0.35	0.03	0.11	0.63	9.00E-09	0.062	0.89	0.72	1.90E-05
381	ENSG00000137819.13	PAQR5	0.84	-0.63	-0.4	-0.27	0.36	1.80E-17	2.90E-08	2.60E-04	0.064	8.50E-04
381	ENSG00000197299.10	BLM	0.84	0.35	1.5	0.35	1.16	9.80E-04	0.32	1.70E-11	0.38	7.00E-07
381	ENSG00000240065.7	PSMB9	0.84	0.35	1.35	0.15	0.91	0.0011	0.32	4.40E-09	0.77	2.10E-04
381	ENSG00000241990.5	PRR34-AS1	0.84	0.45	0.55	0.35	0.64	0.037	0.4	0.18	0.59	0.11
381	ENSG00000116117.17	PARD3B	0.83	0.19	0.99	0.29	1.16	2.00E-14	0.25	1.50E-21	0.056	4.70E-29
381	ENSG00000151834.15	GABRA2	0.83	-0.75	-0.35	-0.16	0.56	5.60E-36	5.60E-26	9.30E-07	0.11	1.50E-16
381	ENSG00000166073.10	GPR176	0.83	0.15	0.99	0.43	1.11	1.80E-70	0.01	7.80E-104	1.60E-17	3.00E-128
381	ENSG00000139926.15	FRMD6	0.82	-0.12	0.94	0.3	1.19	9.40E-30	0.3	6.70E-40	7.00E-04	2.10E-63
381	ENSG00000221963.5	APOL6	0.82	-0.04	0.68	0.11	0.93	1.60E-22	0.82	3.40E-16	0.51	8.60E-30
381	ENSG00000271020.1	AC112220.2	0.82	0.31	0.63	0.26	0.38	0.016	0.53	0.058	0.67	0.31
381	ENSG00000091651.8	ORC6	0.81	0.56	1.55	0.33	1.15	4.80E-05	0.013	2.20E-18	0.26	3.70E-10
381	ENSG00000120217.13	CD274	0.81	0.55	1.38	0.47	1.33	1.80E-05	0.0098	5.70E-17	0.056	3.40E-15
381	ENSG00000134917.9	ADAMTS8	0.81	-0.59	-0.37	0.55	0.48	0.0017	0.052	0.2	0.097	0.076
381	ENSG00000136205.16	TNS3	0.81	-0.02	0.67	0.37	1.1	3.90E-49	0.86	3.90E-34	2.20E-09	1.60E-92
381	ENSG00000146670.9	CDCA5	0.81	0.54	1.64	0.38	1.24	2.50E-05	0.012	2.20E-21	0.16	3.20E-12
381	ENSG00000196584.2	XRCC2	0.81	0.28	1.61	0.39	1.18	0.001	0.45	2.80E-14	0.28	1.30E-07
381	ENSG00000012048.20	BRCA1	0.8	0.5	1.47	0.31	1.32	2.90E-05	0.021	7.70E-18	0.28	3.30E-14
381	ENSG00000131153.8	GINS2	0.8	0.46	1.32	0.52	1.22	1.20E-05	0.035	2.30E-15	0.023	5.40E-13
381	ENSG00000133321.10	RARRES3	0.8	0.11	0.63	0.47	1.03	0.0011	0.81	0.0082	0.16	5.00E-06
381	ENSG00000234745.10	HLA-B	0.8	-0.13	0.68	0.15	0.79	1.10E-29	0.23	5.30E-22	0.14	4.50E-29
381	ENSG00000237773.5	AC003075.	0.8	-0.11	0	0.12	0.42	0.046	0.89	1	2	0.34
381	ENSG00000260878.1	AC104072.	0.8	-1.48	-0.55	-0.54	0.22	0.017	9.40E-05	0.13	0.27	0.6
381	ENSG00000133119.12	RFC3	0.79	0.53	1.35	0.5	1.09	8.30E-07	0.0031	9.30E-21	0.01	3.00E-13
381	ENSG00000143179.14	UCK2	0.79	0.32	1.11	0.46	0.99	2.30E-21	8.50E-04	8.10E-44	1.20E-06	9.60E-35
381	ENSG00000164663.14	USP49	0.79	-0.07	0.7	0.05	0.82	1.50E-11	0.76	8.40E-10	0.87	4.80E-13
381	ENSG00000184292.6	TACSTD2	0.79	-0.7	-0.32	-0.08	0.6	0.0029	0.029	0.31	0.9	0.026
381	ENSG00000261061.1	AC092718.	0.79	0.33	0.98	0.12	0.87	0.0014	0.34	1.20E-05	0.82	2.00E-04
381	ENSG00000108797.11	CNTNAP1	0.78	0.09	0.74	0.31	1.03	6.50E-17	0.56	1.30E-15	0.011	7.00E-30
381	ENSG00000113368.11	LMNB1	0.78	0.51	1.46	0.47	1.17	8.70E-05	0.023	5.40E-16	0.068	2.40E-10
381	ENSG00000137767.13	SQOR	0.78	-0.39	0.21	-0.08	0.59	2.30E-08	0.028	0.2	0.79	2.60E-05
381	ENSG00000140937.13	CDH11	0.78	0.42	0.94	0.18	0.64	6.70E-48	8.40E-14	6.20E-72	0.0093	5.30E-33
381	ENSG00000144749.13	LRIG1	0.78	-0.08	0.71	0.24	1.07	1.30E-49	0.33	2.90E-42	1.60E-04	5.20E-96
381	ENSG00000109738.10	GLRB	0.77	0.24	0.77	0.3	1.28	4.40E-06	0.32	1.60E-06	0.25	3.50E-17
381	ENSG00000114529.12	C3orf52	0.77	0.33	1.3	0.12	1.53	0.0028	0.36	3.20E-09	0.83	3.00E-12
381	ENSG00000164045.11	CDC25A	0.77	0.4	1.59	0.21	1.18	5.60E-05	0.092	3.50E-21	0.54	1.80E-11
381	ENSG00000165868.13	HSPA12A	0.77	0.33	1.01	0.32	0.95	1.10E-16	0.003	1.00E-30	0.0079	2.70E-26
381	ENSG00000198355.4	PIM3	0.77	0.21	0.86	0.02	0.61	1.50E-33	0.0085	1.30E-43	0.9	1.40E-21
381	ENSG00000266094.7	RASSF5	0.77	-0.34	0.15	0.28	0.27	6.10E-04	0.28	0.62	0.43	0.32
381	ENSG00000161381.13	PLXDC1	0.76	-0.04	0.98	-0.13	0.9	0.024	0.96	9.40E-04	0.87	0.0035
381	ENSG00000183783.6	KCTD8	0.76	0.08	0.62	0.54	1.13	7.00E-05	0.83	9.20E-04	0.022	1.10E-10
381	ENSG00000243649.8	CFB	0.76	-0.49	0.27	-0.12	0.85	0.019	0.28	0.46	0.87	0.0044
381	ENSG00000279822.1	AC016397.2	0.76	0.45	0.95	0.12	0.79	0.032	0.33	0.0028	0.87	0.019
381	ENSG00000091127.13	PUS7	0.75	0.47	1.19	0.34	0.91	2.80E-10	3.10E-04	6.30E-28	0.034	8.20E-16
381	ENSG00000115946.7	PNO1	0.75	0.42	0.99	0.47	0.87	6.40E-12	6.40E-04	1.80E-21	2.20E-04	2.90E-16
381	ENSG00000125965.8	GDF5	0.75	-0.03	0.68	0.63	1.36	0.016	0.96	0.021	0.098	6.70E-07
381	ENSG00000137975.7	CLCA2	0.75	-0.19	-0.11	0.15	0.65	0.01	0.71	0.78	0.79	0.024
381	ENSG00000073111.13	MCM2	0.74	0.44	1.13	0.41	1.12	1.70E-08	0.0031	5.90E-20	0.011	2.10E-19
381	ENSG00000104689.9	TNFRSF10A	0.74	0.39	1.1	0.17	0.84	1.80E-09	0.0068	6.50E-22	0.43	1.50E-12
381	ENSG00000140526.17	ABHD2	0.74	-0.23	0.34	0.13	0.73	1.70E-42	3.00E-04	1.40E-09	0.096	2.20E-41
381	ENSG00000151789.10	ZNF385D	0.74	0.37	1.13	-0.1	0.6	1.30E-10	0.0064	2.80E-26	0.69	2.10E-07

381	ENSG00000197442.9	MAP3K5	0.74	-0.11	0.44	0.07	0.88	5.20E-32	0.26	5.00E-12	0.55	9.00E-46
381	ENSG00000277161.1	PIGW	0.74	0.37	1.1	0.34	0.9	4.70E-09	0.015	6.30E-21	0.045	7.60E-14
381	ENSG00000105835.11	NAMPT	0.73	0.02	0.45	0.09	0.97	9.50E-23	0.92	2.30E-09	0.54	1.20E-40
381	ENSG00000143942.4	CHAC2	0.73	0.24	1.62	0.52	1.23	0.044	0.66	3.00E-08	0.28	7.10E-05
381	ENSG00000145362.17	ANK2	0.73	0.06	1.57	-0.09	0.66	7.90E-18	0.72	4.80E-86	0.63	6.00E-15
381	ENSG00000156265.15	MAP3K7CL	0.73	0.09	1	0.28	1.05	6.70E-06	0.77	1.40E-11	0.24	2.90E-12
381	ENSG00000171793.13	CTPS1	0.73	0.46	1.13	0.3	1.02	2.50E-17	6.40E-07	2.90E-43	0.0058	8.10E-35
381	ENSG00000179104.8	TMTC2	0.73	-0.51	0.33	-0.04	0.94	5.60E-06	0.012	0.065	0.92	5.50E-10
381	ENSG00000100911.15	PSME2	0.72	0.23	1.12	0.1	0.73	4.00E-22	0.011	3.30E-56	0.45	1.10E-23
381	ENSG00000108932.11	SLC16A6	0.72	-0.37	0.32	-0.23	0.22	6.10E-05	0.12	0.11	0.45	0.31
381	ENSG00000134107.4	BHLHE40	0.72	0.48	1.25	0.37	1.11	1.20E-09	1.90E-04	2.50E-29	0.013	1.00E-22
381	ENSG00000137364.4	TPMT	0.72	0.11	1.01	0	0.81	3.00E-13	0.5	1.70E-27	1	1.50E-17
381	ENSG00000137462.6	TLR2	0.72	0.34	0.79	-0.06	0.7	7.00E-10	0.016	6.00E-13	0.83	6.60E-10
381	ENSG00000168672.3	FAM84B	0.72	0.28	0.93	-0.05	0.47	7.60E-05	0.27	2.50E-08	0.91	0.013
381	ENSG00000173145.11	NOC3L	0.72	0.21	0.6	0.09	0.63	2.50E-12	0.13	3.90E-09	0.69	9.00E-10
381	ENSG00000178974.9	FBXO34	0.72	0.02	0.83	0.24	0.88	1.80E-32	0.9	7.80E-45	0.0027	1.40E-49
381	ENSG00000109320.11	NFKB1	0.71	-0.05	0.72	0.28	0.75	6.40E-23	0.71	9.70E-25	0.0024	3.60E-26
381	ENSG00000148680.15	HTR7	0.71	-0.43	0.15	0.02	0.61	1.90E-04	0.076	0.55	0.97	0.0011
381	ENSG00000156140.9	ADAMTS3	0.71	0.21	0.48	0.29	0.62	0.0027	0.58	0.045	0.44	0.0072
381	ENSG00000156876.9	SASS6	0.71	0.5	0.91	0.05	0.76	0.0064	0.1	9.00E-05	0.94	0.0019
381	ENSG00000161011.19	SQSTM1	0.71	-0.07	0.54	0.21	0.76	1.20E-66	0.31	6.80E-38	1.80E-05	5.00E-75
381	ENSG00000169621.9	APLF	0.71	-0.09	0.45	0.17	0.6	2.80E-05	0.8	0.0097	0.59	3.60E-04
381	ENSG00000184545.10	DUSP8	0.71	0.4	1.21	0.58	1.03	5.90E-04	0.11	2.40E-11	0.019	3.40E-08
381	ENSG00000204923.3	FBXO48	0.71	0.26	0.51	-0.13	0.48	0.02	0.56	0.091	0.84	0.13
381	ENSG00000205220.11	PSMB10	0.71	0.45	0.96	0.33	0.81	3.90E-10	2.90E-04	1.10E-19	0.026	1.20E-13
381	ENSG00000274964.1	AC026356.1	0.71	0.47	0.41	0.18	0.44	0.041	0.27	0.25	0.79	0.23
381	ENSG00000103642.11	LACTB	0.7	-0.07	0.56	0.19	0.83	2.40E-16	0.68	4.30E-11	0.13	1.20E-23
381	ENSG00000105825.11	TFPI2	0.7	-1.47	-0.72	-0.48	0.1	2.90E-04	3.30E-16	9.40E-05	0.044	0.71
381	ENSG00000122861.15	PLAU	0.7	0.32	0.89	0.24	0.9	5.80E-16	9.40E-04	1.10E-26	0.035	6.30E-27
381	ENSG00000135763.9	URB2	0.7	0.48	1.01	0.32	0.96	5.90E-08	7.40E-04	4.50E-17	0.07	7.10E-15
381	ENSG00000138030.12	KHK	0.7	0.52	1.15	0.28	0.77	0.048	0.22	1.20E-04	0.63	0.018
381	ENSG00000140511.11	HAPLN3	0.7	0.29	0.49	0.16	0.52	8.00E-06	0.15	0.0018	0.59	0.001
381	ENSG00000145779.7	TNFAIP8	0.7	-0.11	0.65	0.13	0.77	8.80E-15	0.46	1.70E-13	0.4	2.20E-18
381	ENSG00000150630.3	VEGFC	0.7	0.12	0.74	0.35	1.02	1.20E-28	0.2	3.30E-33	3.00E-06	6.80E-63
381	ENSG00000151320.10	AKAP6	0.7	-0.14	0.33	-0.46	0.24	0.013	0.78	0.29	0.26	0.48
381	ENSG00000160050.14	CCDC28B	0.7	-0.01	0.76	-0.03	0.68	1.40E-09	0.97	6.80E-12	0.91	2.10E-09
381	ENSG00000164171.10	ITGA2	0.7	-0.03	0.36	-0.18	0.51	4.90E-19	0.87	1.10E-05	0.1	1.20E-10
381	ENSG00000172716.16	SLFN11	0.7	0.17	0.78	0.14	0.74	2.30E-29	0.038	5.60E-38	0.14	2.80E-33
381	ENSG00000179361.17	ARID3B	0.7	0.33	0.5	-0.07	0.42	4.90E-06	0.084	0.0011	0.84	0.01
381	ENSG00000198554.11	WDHD1	0.7	0.37	1.13	0.16	0.88	2.40E-07	0.02	9.60E-20	0.49	7.90E-12
381	ENSG00000120833.13	SOCS2	0.69	-0.08	0.72	0.09	0.61	1.40E-15	0.63	7.90E-18	0.59	1.30E-12
381	ENSG00000135002.11	RFK	0.69	0.35	0.79	0.33	0.66	3.30E-10	0.007	2.40E-14	0.018	6.50E-10
381	ENSG00000135074.15	ADAM19	0.69	0.19	0.64	0.27	0.65	8.40E-19	0.055	6.20E-17	0.0076	1.60E-17
381	ENSG00000158286.12	RNF207	0.69	0.05	0.52	-0.2	0.54	0.003	0.91	0.027	0.64	0.021
381	ENSG00000171791.12	BCL2	0.69	-0.25	0.32	0.45	0.71	0.013	0.57	0.3	0.23	0.0068
381	ENSG00000213988.10	ZNF90	0.69	0.41	0.59	-0.21	0.53	0.042	0.35	0.07	0.75	0.12
381	ENSG00000217801.9	AL390719.1	0.69	0.28	0.82	-0.12	0.52	3.90E-10	0.047	6.90E-15	0.58	2.70E-06
381	ENSG00000042062.11	RIPOR3	0.68	-0.46	0.17	0.08	0.65	1.20E-16	3.70E-07	0.075	0.65	1.10E-15
381	ENSG00000055163.19	CYFIP2	0.68	0.02	0.61	0.08	0.6	1.80E-10	0.93	5.90E-09	0.73	1.60E-08
381	ENSG00000077157.21	PPP1R12B	0.68	-0.18	0.09	0.31	1	1.90E-13	0.17	0.47	0.0083	1.00E-29
381	ENSG00000092470.11	WDR76	0.68	0.36	0.99	0.32	0.82	1.20E-05	0.057	5.80E-12	0.14	2.30E-08
381	ENSG00000100297.15	MCM5	0.68	0.26	1.01	0.27	0.9	1.60E-08	0.094	7.40E-19	0.1	6.40E-15
381	ENSG00000109790.16	KLHL5	0.68	-0.23	0.24	0.23	0.63	6.50E-30	0.0011	1.50E-04	0.0022	1.50E-25
381	ENSG00000136867.10	SLC31A2	0.68	0.07	0.52	-0.12	0.7	4.70E-04	0.84	0.0063	0.77	2.00E-04
381	ENSG00000137809.16	ITGA11	0.68	-0.1	-0.04	0.19	0.49	3.10E-10	0.6	0.83	0.26	7.20E-06
381	ENSG00000156802.12	ATAD2	0.68	0.42	1.24	0.25	1.01	3.10E-08	0.0025	2.30E-28	0.15	1.40E-18
381	ENSG00000160606.10	TLCD1	0.68	0.3	0.66	0.21	1	2.50E-06	0.1	2.20E-06	0.36	1.20E-13
381	ENSG00000176890.15	TYMS	0.68	0.2	1.01	0.18	0.81	8.80E-08	0.26	1.80E-17	0.37	3.60E-11
381	ENSG00000180263.13	FGD6	0.68	-0.18	0.68	-0.12	0.8	4.60E-07	0.37	1.70E-07	0.67	4.40E-10
381	ENSG00000184922.13	FMNL1	0.68	0.01	0.65	-0.02	0.62	5.50E-07	0.98	5.30E-07	0.96	2.80E-06
381	ENSG00000184979.9	USP18	0.68	0.05	0.14	0.49	0.65	6.40E-05	0.89	0.53	0.017	7.10E-05
381	ENSG00000246985.7	SOCS2-AS1	0.68	0.3	0.47	0.11	0.73	0.0082	0.41	0.071	0.84	0.0028
381	ENSG00000058804.11	NDC1	0.67	0.45	1.02	0.21	0.72	5.90E-09	3.30E-04	8.50E-22	0.23	7.30E-11
381	ENSG00000069482.6	GAL	0.67	0.08	0.56	-0.22	0.14	2.80E-13	0.6	6.60E-10	0.083	0.2
381	ENSG00000106565.17	TMEM176B	0.67	-0.91	-0.83	0.06	0.35	0.026	0.0067	0.0058	0.93	0.29
381	ENSG00000138685.13	FGF2	0.67	0.02	0.58	0.16	0.78	1.70E-21	0.91	7.70E-17	0.12	1.80E-29
381	ENSG00000150995.18	ITPR1	0.67	0.27	0.8	0.49	0.59	3.30E-04	0.29	2.60E-06	0.033	0.0013
381	ENSG00000164307.12	ERAP1	0.67	0.14	0.68	0.13	0.72	3.40E-33	0.057	1.50E-35	0.099	1.40E-39
381	ENSG00000078269.14	SYNJ2	0.66	0.16	0.86	0.1	0.8	4.70E-40	0.0087	9.00E-71	0.18	3.40E-60
381	ENSG00000136048.13	DRAM1	0.66	-0.15	0.29	0.14	0.6	6.00E-31	0.055	1.90E-06	0.09	8.30E-26
381	ENSG00000162063.12	CCNF	0.66	0.52	1.31	0.33	1.11	1.70E-04	0.0069	1.50E-17	0.17	2.40E-12
381	ENSG00000166801.15	FAM111A	0.66	0.18	0.77	0.15	0.76	8.90E-15	0.11	2.20E-21	0.29	1.90E-20
381	ENSG00000186998.15	EMID1	0.66	-1.46	-1.01	-0.48	0.13	0.029	1.50E-06	3.70E-04	0.24	0.74
381	ENSG00000239672.7	NME1	0.66	0.45	1.06	0.31	0.83	9.50E-07	0.0026	7.60E-18	0.084	9.40E-11
381	ENSG00000108846.15	ABCC3	0.65	0.57	0.73	0.01	0.63	4.30E-06	1.30E-04	5.50E-08	0.98	5.80E-06
381	ENSG00000110218.8	PANX1	0.65	0.19	0.77	0.26	0.77	4.20E-47	4.00E-04	7.80E-69	8.30E-07	5.50E-68
381	ENSG00000111331.12	OAS3	0.65	0.23	1.08	0.28	0.96	0.012	0.54	1.70E-06	0.49	3.70E-05
381	ENSG00000112118.18	MCM3	0.65	0.27	1	0.27	1	3.70E-08	0.075	1.80E-19	0.1	3.90E-19
381	ENSG00000116815.15	CD58	0.65	0	0.92	0.14	0.44	2.60E-04	1	5.90E-09	0.69	0.017
381	ENSG00000128228.4	SDF2L1	0.65	0.09	0.73	0.24	0.91	7.20E-07	0.72	4.20E-09	0.22	1.70E-13

381	ENSG00000136002.18	ARHGEF4	0.65	0.09	0.46	0.33	0.79	0.02	0.87	0.098	0.43	0.0021
381	ENSG00000158201.9	ABHD3	0.65	0.28	0.63	0.15	0.58	9.80E-04	0.3	8.10E-04	0.69	0.0031
381	ENSG00000163918.10	RFC4	0.65	0.38	1.02	0.37	0.93	3.60E-06	0.019	1.10E-15	0.045	1.10E-12
381	ENSG00000006283.17	CACNA1G	0.64	0.22	0.23	-0.35	0.23	0.011	0.56	0.42	0.36	0.44
381	ENSG00000019582.14	CD74	0.64	-0.14	1.12	0.08	0.8	4.60E-04	0.64	8.90E-12	0.84	2.50E-06
381	ENSG00000026103.21	FAS	0.64	-0.08	0.82	0.14	0.63	1.10E-11	0.65	9.90E-20	0.38	8.50E-12
381	ENSG00000110446.10	SLC15A3	0.64	0.29	0.94	0.31	0.79	6.90E-04	0.25	2.60E-08	0.25	7.20E-06
381	ENSG00000112210.11	RAB23	0.64	-0.14	0.17	0.29	0.68	4.70E-20	0.15	0.034	6.20E-04	5.50E-23
381	ENSG00000132329.10	RAMP1	0.64	0.34	1.22	0.24	0.49	9.90E-09	0.009	9.10E-33	0.12	1.00E-05
381	ENSG00000168386.18	FILIP1L	0.64	-0.84	-0.5	0.15	0.52	1.70E-12	3.10E-20	4.90E-08	0.29	9.70E-09
381	ENSG00000169884.13	WNT10B	0.64	0.25	0.96	0.23	0.76	0.0036	0.43	7.80E-07	0.55	2.00E-04
381	ENSG00000170385.9	SLC30A1	0.64	0.23	0.5	0.24	0.6	2.60E-17	0.01	1.70E-11	0.016	5.00E-16
381	ENSG00000184162.14	NR2C2AP	0.64	0.17	0.88	0.19	0.77	0.002	0.6	2.00E-06	0.59	6.40E-05
381	ENSG00000184992.10	BRI3BP	0.64	0.21	1.09	0.36	0.97	0.0035	0.52	8.30E-09	0.23	6.70E-07
381	ENSG00000205269.5	TMEM170B	0.64	0.11	0.58	0.27	0.53	0.0035	0.77	0.0047	0.42	0.015
381	ENSG00000214706.10	IFRD2	0.64	0.31	1	0.28	0.86	3.00E-13	0.0026	3.00E-34	0.014	1.70E-24
381	ENSG00000054967.12	RELT	0.63	0.23	0.89	0.03	0.77	5.20E-04	0.37	3.40E-08	0.94	5.10E-06
381	ENSG00000087085.13	ACHE	0.63	-0.4	0	-0.29	0.11	0.01	0.24	1	0.49	0.75
381	ENSG00000108639.7	SYNGR2	0.63	0.25	0.82	0.1	0.61	3.30E-13	0.018	3.20E-23	0.51	8.30E-13
381	ENSG00000160193.11	WDR4	0.63	0.15	0.6	0.16	0.71	2.00E-06	0.47	2.60E-06	0.48	2.10E-08
381	ENSG00000165732.12	DDX21	0.63	0.33	1.01	0.26	0.86	5.50E-22	3.50E-06	8.10E-59	0.0016	3.00E-42
381	ENSG00000169570.9	DTWD2	0.63	0.23	0.64	0.46	1	0.018	0.57	0.0089	0.18	1.60E-05
381	ENSG00000170485.16	NPAS2	0.63	0.13	0.47	-0.01	1	3.50E-17	0.21	1.30E-10	0.98	2.20E-45
381	ENSG00000064932.15	SBNO2	0.62	0.16	0.66	0.23	0.81	8.40E-20	0.07	8.20E-23	0.0074	5.90E-34
381	ENSG00000080839.11	RBL1	0.62	0.41	1.02	0.21	0.91	5.90E-07	0.0035	9.50E-20	0.26	2.80E-15
381	ENSG00000101680.14	LAMA1	0.62	-0.24	-0.05	0.19	0.42	6.20E-08	0.11	0.77	0.28	3.00E-04
381	ENSG00000106538.9	RARRES2	0.62	-0.33	-0.18	0.18	0.44	2.40E-06	0.04	0.25	0.4	9.90E-04
381	ENSG00000114346.13	ECT2	0.62	0.41	1.1	0.09	0.76	1.10E-05	0.011	2.10E-17	0.75	1.30E-08
381	ENSG00000134352.19	IL6ST	0.62	0.06	0.54	0.09	0.63	6.70E-19	0.64	4.10E-15	0.43	3.30E-20
381	ENSG00000143507.17	DUSP10	0.62	0.18	0.83	0.55	1.15	1.50E-06	0.36	4.20E-12	1.20E-04	1.90E-22
381	ENSG00000151702.16	FLI1	0.62	0.14	0.77	0.22	0.4	0.044	0.79	0.0045	0.69	0.21
381	ENSG00000163002.12	NUP35	0.62	0.17	0.83	0.01	0.51	6.60E-05	0.49	4.60E-09	0.98	0.0011
381	ENSG00000169855.19	ROBO1	0.62	-0.16	0.32	0.09	0.66	2.20E-35	0.011	2.10E-10	0.28	4.90E-41
381	ENSG00000172197.10	MBOAT1	0.62	0.03	0.62	-0.07	0.4	0.012	0.96	0.0074	0.9	0.12
381	ENSG00000173391.8	OLR1	0.62	0.19	0.72	0.62	1.13	0.03	0.67	0.0039	0.059	2.10E-06
381	ENSG00000173402.11	DAG1	0.62	0.32	0.81	0.3	0.72	3.90E-20	1.50E-05	6.40E-36	1.50E-04	2.40E-28
381	ENSG00000182985.17	CADM1	0.62	0.1	0.74	0.44	0.94	1.30E-08	0.57	3.70E-13	4.00E-04	1.90E-20
381	ENSG00000106366.8	SERPINE1	0.61	0.58	1.3	0.47	1.23	3.70E-06	2.30E-05	1.00E-26	0.002	1.80E-23
381	ENSG00000157483.8	MYO1E	0.61	0.33	0.87	0.45	0.83	2.10E-18	1.50E-05	2.00E-39	3.70E-09	1.90E-35
381	ENSG00000160326.13	SLC2A6	0.61	0.48	1.26	0.5	1.2	1.10E-04	0.0051	1.70E-19	0.0071	2.40E-17
381	ENSG00000181649.5	PHLDA2	0.61	0.51	1.04	0.45	0.85	3.90E-10	5.50E-07	5.00E-30	4.00E-05	1.10E-19
381	ENSG00000182667.14	NTM	0.61	-0.18	0.26	0.55	0.89	3.00E-12	0.14	0.0052	3.90E-09	4.20E-27
381	ENSG00000236830.6	CBR3-AS1	0.61	0.22	0.38	0.32	0.38	0.042	0.64	0.22	0.48	0.23
381	ENSG00000014138.8	POLA2	0.6	0.36	0.96	0.27	0.98	3.70E-05	0.034	3.60E-13	0.2	2.20E-13
381	ENSG00000077152.9	UBE2T	0.6	0.29	1.28	0.28	0.84	0.0035	0.28	2.00E-13	0.35	5.80E-06
381	ENSG00000097046.12	CDC7	0.6	0.33	0.78	0.24	0.77	4.60E-04	0.12	6.00E-07	0.37	1.10E-06
381	ENSG00000100629.16	CEP128	0.6	0.48	1.15	0.28	0.86	0.01	0.073	6.10E-09	0.43	4.50E-05
381	ENSG00000124508.16	BTN2A2	0.6	0.03	0.65	-0.04	0.6	1.80E-06	0.9	3.30E-08	0.89	7.30E-07
381	ENSG00000133740.10	E2F5	0.6	0.46	0.91	0.34	0.75	0.0021	0.036	1.40E-07	0.22	3.40E-05
381	ENSG00000136490.8	LIMD2	0.6	-0.02	0.81	0.15	0.7	3.30E-14	0.9	1.30E-26	0.23	9.10E-20
381	ENSG00000138376.10	BARD1	0.6	0.55	1.15	0.41	1.09	0.0013	0.0048	4.40E-13	0.088	1.70E-11
381	ENSG00000154920.14	EME1	0.6	0.46	1.06	0	0.8	0.028	0.14	3.50E-06	1	0.0011
381	ENSG00000162433.14	AK4	0.6	0.19	0.1	0.08	0.53	0.026	0.66	0.78	0.89	0.045
381	ENSG00000163814.7	CDCP1	0.6	0.35	0.76	0.03	0.55	1.80E-15	2.50E-05	3.50E-25	0.85	2.60E-13
381	ENSG00000182326.14	C1S	0.6	-0.22	-0.05	0.25	0.67	9.60E-10	0.083	0.75	0.06	2.90E-12
381	ENSG00000005059.15	MCUB	0.59	0.33	0.85	0.25	0.64	4.00E-05	0.062	1.30E-10	0.24	3.20E-06
381	ENSG00000120334.15	CENPL	0.59	0.25	0.95	0.34	0.81	5.50E-05	0.19	4.90E-13	0.081	3.10E-09
381	ENSG00000137124.7	ALDH1B1	0.59	0.47	0.92	0.38	0.92	1.90E-08	2.90E-05	4.00E-21	0.0027	1.60E-20
381	ENSG00000148798.10	INA	0.59	0.44	1.31	0.3	0.48	7.90E-04	0.027	7.70E-18	0.22	0.0064
381	ENSG00000159403.15	C1R	0.59	-0.37	-0.3	0.2	0.64	3.10E-06	0.011	0.023	0.28	1.30E-07
381	ENSG00000166979.12	EVA1C	0.59	0.37	0.73	-0.01	0.56	0.029	0.27	0.0025	0.99	0.03
381	ENSG00000167767.13	KRT80	0.59	0.56	1.43	0.44	1	7.40E-04	0.002	1.70E-22	0.037	8.00E-11
381	ENSG00000173611.17	SCAI	0.59	0.16	0.68	-0.23	0.38	2.00E-06	0.39	6.90E-09	0.22	0.0025
381	ENSG00000184445.11	KNTC1	0.59	0.43	1.03	0.08	0.83	5.70E-06	0.0027	2.90E-18	0.76	9.10E-12
381	ENSG00000198855.6	FICD	0.59	0.24	0.62	0.24	0.71	1.10E-05	0.19	9.00E-07	0.23	2.30E-08
381	ENSG00000226067.6	LINC00623	0.59	-0.13	0.14	0.06	0.28	0.03	0.79	0.68	0.93	0.36
381	ENSG00000248905.8	FMN1	0.59	0.49	1.22	0.27	0.81	4.20E-08	1.20E-05	6.60E-35	0.06	1.90E-15
177	ENSG00000002587.9	HS3ST1	-1.27	0.0033	3.64	7.40E-40	4.12	2.50E-52	-0.09	0.92	-1.7	8.40E-05
177	ENSG00000058404.19	CAMK2B	0.62	0.083	3.33	2.00E-38	3.61	5.70E-46	0.25	0.68	0.53	0.12
177	ENSG00000011201.11	ANOS1	0.51	0.22	2.99	1.10E-05	4.6	9.40E-10	0.85	2	0.97	0.021
177	ENSG00000114737.15	CISH	0	1	2.66	1.80E-98	2.74	6.80E-106	0.08	0.81	-0.13	0.49
177	ENSG00000115602.16	IL1RL1	0.54	0.27	2.34	2.40E-10	3.47	1.20E-23	0.42	0.55	0.01	0.99
177	ENSG00000183134.4	PTGDR2	-0.65	3.00E-07	2.32	1.60E-101	1.77	4.60E-59	-0.1	0.69	-1.09	6.40E-19
177	ENSG00000167642.12	SPINT2	-0.15	0.55	2.11	1.20E-46	2.84	5.70E-88	0.25	0.35	0.04	0.88
177	ENSG00000079841.18	RIMS1	-0.29	0.61	1.82	1.10E-06	2.33	1.00E-11	-0.16	0.86	-0.39	0.44
177	ENSG00000174564.12	IL20RB	0.52	0.24	1.68	1.20E-06	1.29	1.20E-04	0.64	0.21	0.98	0.0065
177	ENSG00000128510.10	CPA4	0.54	0.013	1.67	7.00E-19	2.45	8.70E-42	0.57	0.022	0.96	8.40E-07
177	ENSG00000157111.12	TMEM171	0.36	0.027	1.64	1.70E-38	2.08	2.20E-64	0.18	0.45	0.41	0.0061
177	ENSG00000110104.11	CCDC86	0.38	1.50E-05	1.57	4.50E-91	2.06	1.40E-160	0.18	0.13	0.54	9.60E-11
177	ENSG00000108551.4	RASD1	0.13	0.77	1.56	3.00E-08	1.86	2.30E-12	0.07	0.92	0.22	0.56

177	ENSG00000141668.9	CBLN2	0.59	0.27	1.56	4.80E-04	2.61	2.70E-11	0.15	2	0.5	0.33
177	ENSG00000171492.14	LRRC8D	0.48	3.80E-07	1.56	3.60E-75	1.83	7.40E-105	0.33	0.0043	0.7	4.90E-15
177	ENSG00000198963.10	RORB	-0.49	0.0065	1.45	3.70E-23	1.23	2.90E-17	-0.34	0.14	-0.94	1.30E-08
177	ENSG00000259518.1	LINC01583	0	1	1.45	1.30E-04	1.62	2.70E-06	0.05	0.96	0.95	0.012
177	ENSG00000135914.5	HTR2B	-0.26	0.33	1.41	5.00E-14	1.34	2.30E-13	0.11	0.8	-0.32	0.18
177	ENSG00000101825.7	MXRA5	-0.53	5.10E-05	1.28	2.50E-27	0.59	1.60E-06	0.17	0.41	-0.4	0.0025
177	ENSG00000106537.7	TSPAN13	-0.26	0.055	1.26	2.70E-35	1.03	5.20E-24	-0.32	0.027	-0.92	1.20E-15
177	ENSG00000118513.18	MYB	0.42	0.47	1.24	0.04	2.52	1.50E-05	0.69	2	1.7	0.0023
177	ENSG00000232759.1	AC002480.	0.39	0.53	1.17	0.027	1.7	2.10E-04	0.76	2	0.71	0.16
177	ENSG00000156515.21	HK1	0.16	0.0025	1.15	1.40E-150	1.18	7.30E-161	0.08	0.27	0.23	2.30E-06
177	ENSG00000225697.12	SLC26A6	-0.38	0.046	1.15	8.90E-13	0.31	0.095	-0.16	0.61	-0.55	0.0012
177	ENSG00000111424.10	VDR	0.47	1.60E-08	1.14	1.60E-51	1.48	1.70E-89	0.44	7.10E-07	0.9	1.70E-31
177	ENSG00000174136.11	RGMB	0.54	9.20E-11	1.14	2.40E-48	1.74	3.40E-114	0.32	0.001	0.93	2.20E-32
177	ENSG00000120129.5	DUSP1	0.1	0.2	1.13	5.50E-90	1.05	4.80E-79	0.58	6.90E-22	0.51	4.00E-18
177	ENSG00000169297.7	NR0B1	0	1	1.13	0.037	0.36	0.54	0.2	2	-0.16	0.81
177	ENSG00000276644.4	DACH1	-0.36	0.013	1.13	4.10E-21	0.7	7.30E-09	-0.2	0.33	-0.82	2.70E-10
177	ENSG00000117152.13	RGS4	0.28	0.11	1.12	6.80E-16	2.22	1.40E-65	0.23	0.32	0.62	2.10E-05
177	ENSG00000235641.4	LINC00484	0.22	2	1.12	2	1.15	0.072	-0.24	2	0.61	0.39
177	ENSG00000168356.11	SCN11A	-0.46	0.16	1.1	2.20E-05	0.49	0.079	-0.71	0.04	-0.7	0.015
177	ENSG00000249700.8	SRD5A3-AS	0.71	0.17	1.09	0.028	0.55	0.27	0.99	2	0.88	0.057
177	ENSG00000123685.8	BATF3	-0.11	0.75	1.08	8.00E-07	1.42	1.00E-12	0.13	0.79	0.33	0.2
177	ENSG00000197147.13	LRRC8B	0.53	5.70E-05	1.08	2.40E-20	1.91	7.40E-68	0.1	0.7	0.6	1.70E-06
177	ENSG00000116701.14	NCF2	0.44	0.46	1.07	0.036	0.54	0.28	0.15	0.88	0.58	0.26
177	ENSG00000101670.11	LIPG	0.24	0.25	1.06	6.90E-11	0.85	9.20E-08	0.34	0.15	0.71	1.70E-05
177	ENSG00000141404.15	GNAL	0.24	0.55	1.06	2.60E-04	0.27	0.44	0.5	0.23	0.69	0.02
177	ENSG00000184113.9	CLDN5	-0.04	0.96	1.06	0.015	0.85	0.036	-0.12	2	-0.38	0.45
177	ENSG00000077684.15	JADE1	0.17	0.036	1.05	5.00E-61	1.28	3.40E-93	0.51	6.20E-13	0.55	2.00E-16
177	ENSG00000111305.18	GSG1	0.19	0.7	1.05	0.0032	1.14	3.70E-04	0.6	0.21	0.32	0.44
177	ENSG00000116991.10	SIPA1L2	-0.48	0.012	1.04	9.20E-10	0.66	1.10E-04	-0.08	0.82	-0.74	1.60E-05
177	ENSG00000139304.12	PTPRQ	0.2	0.74	1.04	0.02	0.68	0.11	0.38	0.6	0.81	0.055
177	ENSG00000145040.3	UCN2	0.42	0.056	1.02	3.10E-08	1.06	1.40E-09	0.19	0.58	0.48	0.017
177	ENSG00000146072.6	TNFRSF21	0.16	0.087	1.02	4.90E-47	0.64	7.50E-19	0.03	0.84	0.1	0.31
177	ENSG00000169174.10	PCSK9	0.19	0.52	1.02	2.20E-06	1.3	4.30E-11	0.48	0.097	0.74	6.20E-04
177	ENSG00000113805.8	CNTN3	0.5	0.0023	1.01	6.30E-12	0.81	1.90E-08	0.24	0.31	0.51	0.001
177	ENSG00000135931.17	ARMC9	-0.15	0.03	1.01	1.30E-81	0.88	9.90E-63	0.13	0.11	-0.03	0.75
177	ENSG00000128016.5	ZFP36	-0.16	0.46	0.98	6.80E-11	0.39	0.017	-0.06	0.86	-0.14	0.48
177	ENSG00000138821.12	SLC39A8	0.48	0.17	0.98	0.0011	1.53	2.00E-09	0.56	0.16	0.8	0.0055
177	ENSG00000114270.17	COL7A1	0.21	0.38	0.97	2.90E-07	0.68	2.70E-04	0.14	0.72	0.38	0.062
177	ENSG00000118762.7	PKD2	0.02	0.83	0.94	1.40E-62	1.11	4.10E-89	0.04	0.76	0.1	0.16
177	ENSG00000111057.10	KRT18	0.22	0.11	0.93	2.10E-17	1.37	7.10E-39	-0.03	0.91	0.14	0.31
177	ENSG00000128052.8	KDR	0.05	0.88	0.93	2.30E-06	0.05	0.87	0.24	0.48	0.12	0.68
177	ENSG00000198768.10	APCDD1L	0.27	0.019	0.93	1.30E-21	1	8.20E-26	-0.03	0.89	0.21	0.063
177	ENSG00000149571.11	KIRREL3	0.07	0.76	0.92	1.40E-10	1.25	7.00E-21	0.14	0.63	0.3	0.067
177	ENSG00000106484.14	MEST	0.14	0.12	0.91	1.70E-36	1.15	2.90E-59	0.27	0.0026	0.3	1.40E-04
177	ENSG00000158715.5	SLC45A3	-0.05	0.84	0.91	1.50E-12	0.88	1.60E-12	0.01	0.99	0.14	0.41
177	ENSG00000160888.6	IER2	0.11	0.12	0.91	3.50E-68	1	2.20E-82	0.31	3.20E-07	0.42	3.00E-14
177	ENSG00000166396.12	SERPINB7	0.2	0.66	0.9	0.0079	1.23	2.00E-05	0.53	0.23	0.85	0.0063
177	ENSG00000269906.1	AL606834.	0.28	0.59	0.9	0.039	0.78	0.047	0.66	2	0.41	0.37
177	ENSG00000145358.6	DDIT4L	0.24	0.6	0.89	0.013	0.76	0.02	0.56	0.23	0.84	0.011
177	ENSG00000186193.8	SAPCD2	0.45	0.13	0.89	8.50E-04	1.28	3.70E-08	0.25	0.59	0.63	0.017
177	ENSG00000196639.6	HRH1	0.48	4.90E-09	0.89	1.20E-31	0.99	1.90E-40	0.03	0.89	0.42	1.90E-07
177	ENSG00000104332.11	SFRP1	0.1	0.42	0.88	8.90E-23	0.91	4.80E-25	0.1	0.55	-0.03	0.82
177	ENSG00000115604.10	IL18R1	0.56	3.80E-04	0.88	3.40E-09	1.35	7.40E-23	-0.14	0.64	0.32	0.056
177	ENSG00000144821.9	MYH15	0.64	0.096	0.88	0.019	1.27	5.30E-05	-0.38	0.53	0.79	0.023
177	ENSG00000074660.15	SCARF1	0.27	0.58	0.87	0.029	0.68	0.064	0.45	0.42	0.49	0.23
177	ENSG00000164442.9	CITED2	0.17	0.13	0.87	3.70E-23	1.18	2.50E-43	0.44	1.20E-05	0.51	1.70E-08
177	ENSG00000207870.1	MIR221	0.32	0.47	0.86	0.017	1.42	1.60E-06	0.43	0.4	1.15	2.30E-04
177	ENSG00000229694.6	LINC00484	-0.34	0.49	0.86	0.033	0.41	0.33	-0.38	2	0	1
177	ENSG00000115318.11	LOXL3	-0.24	0.084	0.85	2.50E-14	0.24	0.061	0.03	0.91	-0.31	0.012
177	ENSG00000183715.13	OPCML	0.31	0.44	0.85	0.0087	0.58	0.063	0.37	0.45	-0.1	0.83
177	ENSG00000118777.10	ABCG2	0.24	0.42	0.84	3.30E-04	1.34	3.00E-11	0.09	0.86	0.43	0.082
177	ENSG00000147573.16	TRIM55	0.47	0.034	0.84	3.30E-05	1.38	1.40E-14	0.4	0.16	0.82	1.90E-05
177	ENSG00000177663.13	IL17RA	0.18	0.062	0.84	5.50E-27	1.27	1.90E-64	0.3	0.0028	0.2	0.027
177	ENSG00000185070.10	FLRT2	0.22	0.087	0.84	3.90E-16	0.6	4.70E-09	0.15	0.42	0.37	8.60E-04
177	ENSG00000231290.5	APCDD1L-A	0.02	0.93	0.84	2.20E-13	0.84	3.10E-14	-0.08	0.75	-0.07	0.7
177	ENSG00000111087.9	GLI1	0.21	0.3	0.83	1.10E-07	0.77	2.90E-07	-0.17	0.55	-0.08	0.71
177	ENSG00000116771.5	AGMAT	0.52	0.11	0.83	0.0053	1.07	3.30E-05	0.4	0.35	0.92	6.80E-04
177	ENSG00000176887.6	SOX11	0.16	0.056	0.83	6.30E-38	1.31	1.20E-100	-0.09	0.47	-0.1	0.24
177	ENSG00000065320.8	NTN1	0.51	0.059	0.82	0.0011	0.89	7.00E-05	-0.2	0.69	0.45	0.088
177	ENSG00000105963.13	ADAP1	-0.23	0.61	0.82	0.014	0.61	0.05	0.23	0.7	0.36	0.33
177	ENSG00000186480.12	INSIG1	0.27	0.016	0.82	2.50E-17	1.34	5.50E-47	0.37	0.0021	0.8	1.10E-16
177	ENSG00000133110.14	POSTN	0.56	3.30E-17	0.81	7.50E-37	1.18	9.00E-79	0.15	0.11	0.63	4.00E-22
177	ENSG00000165030.3	NFIL3	-0.08	0.44	0.81	2.60E-33	0.99	1.10E-50	0.12	0.3	0.06	0.54
177	ENSG00000170558.8	CDH2	-0.55	2.40E-27	0.81	1.90E-66	0.73	3.90E-55	-0.04	0.67	-0.33	1.60E-10
177	ENSG00000189143.9	CLDN4	0.39	0.021	0.81	8.80E-08	1.4	3.10E-24	0.17	0.52	0.69	2.80E-06
177	ENSG00000110427.14	KIAA1549L	0.28	0.42	0.8	0.0043	1.42	1.50E-09	0.22	0.66	0.66	0.013
177	ENSG00000184838.14	PRR16	0.39	1.20E-06	0.8	6.40E-28	1.18	7.60E-63	0.11	0.42	0.32	4.30E-05
177	ENSG00000165757.8	JCAD	0.24	0.025	0.79	4.60E-18	0.88	3.00E-23	0.54	1.10E-07	0.61	3.50E-11
177	ENSG00000181773.6	GPR3	0.46	0.22	0.79	0.026	1.27	1.20E-05	0.69	0.091	1.02	7.50E-04
177	ENSG00000132334.16	PTPRE	0.4	0.053	0.78	2.30E-05	1.29	1.40E-15	-0.09	0.82	0.17	0.46

177	ENSG00000137877.9	SPTBN5	0.04	0.94	0.78	0.025	0.55	0.095	0.64	0.12	0.64	0.049
177	ENSG00000149218.4	ENDOD1	0.02	0.89	0.78	1.10E-15	0.64	2.50E-11	0.29	0.025	0.23	0.041
177	ENSG00000166851.14	PLK1	0.47	0.019	0.78	2.50E-05	1.62	3.70E-23	0.32	0.23	0.81	3.50E-06
177	ENSG00000099337.4	KCNK6	-0.07	0.66	0.77	6.70E-14	0.77	1.70E-14	0.13	0.49	-0.24	0.047
177	ENSG00000112984.11	KIF20A	0.36	0.19	0.77	0.0015	1.3	6.50E-10	0.37	0.27	0.8	3.60E-04
177	ENSG00000113319.12	RASGRF2	0.4	9.70E-07	0.77	2.40E-25	1.01	6.10E-45	0.12	0.32	0.42	5.10E-08
177	ENSG00000114268.11	PFKFB4	-0.07	0.72	0.77	4.80E-11	0.52	7.50E-06	0.26	0.11	0.13	0.39
177	ENSG00000166016.5	ABTB2	-0.21	0.48	0.77	4.20E-04	0.3	0.21	0.37	0.23	-0.14	0.64
177	ENSG00000133107.14	TRPC4	0.13	0.74	0.76	0.0056	1.39	5.20E-10	0.42	0.26	0.42	0.14
177	ENSG00000088325.15	TPX2	0.56	0.0071	0.75	2.40E-04	1.58	3.20E-19	0.38	0.16	1.06	6.70E-09
177	ENSG00000071282.11	LMCD1	-0.26	0.018	0.74	2.30E-15	1.02	1.30E-30	0.49	2.80E-06	-0.05	0.74
177	ENSG00000130164.13	LDLR	0.21	0.027	0.74	1.50E-20	0.74	9.80E-22	0.41	4.60E-06	0.64	4.80E-16
177	ENSG00000157193.15	LRP8	0.56	4.30E-04	0.74	1.40E-06	1.09	4.20E-15	0.37	0.062	0.88	8.00E-10
177	ENSG00000158402.18	CDC25C	0.48	0.18	0.74	0.03	1.65	7.70E-10	0.35	0.47	1.03	3.90E-04
177	ENSG00000133687.15	TMTC1	0.42	1.80E-07	0.73	2.40E-21	1.18	1.20E-56	0.2	0.062	0.5	1.10E-10
177	ENSG00000138119.16	MYOF	0	0.99	0.73	7.60E-59	0.84	2.60E-78	0.29	1.60E-08	0.29	6.30E-10
177	ENSG00000145730.20	PAM	-0.03	0.74	0.73	2.10E-54	0.66	2.70E-45	-0.04	0.69	-0.11	0.056
177	ENSG00000128203.6	ASPHD2	0.55	0.069	0.72	0.015	0.56	0.04	-0.1	0.88	0.45	0.12
177	ENSG00000136158.11	SPRY2	0.26	0.045	0.72	1.90E-10	1.27	1.40E-33	0.28	0.058	0.6	5.70E-08
177	ENSG00000151474.21	FRMD4A	0.17	0.013	0.71	7.20E-33	1	3.10E-67	0.05	0.67	0.16	0.017
177	ENSG00000170379.19	TCAF2	0.05	0.91	0.71	0.015	1	4.40E-05	-0.1	0.87	-0.06	0.87
177	ENSG00000172183.14	ISG20	-0.31	0.29	0.71	0.0039	0.71	0.0014	-0.17	0.72	-0.01	0.99
177	ENSG00000197261.11	C6orf141	-0.53	0.043	0.71	0.0033	0.45	0.059	-0.55	0.07	-0.52	0.037
177	ENSG00000023909.9	GCLM	0.27	0.03	0.7	2.40E-11	0.72	6.80E-13	0.32	0.017	0.54	2.40E-07
177	ENSG00000139318.7	DUSP6	0.18	0.2	0.7	3.10E-10	0.68	1.80E-10	0.33	0.02	0.36	0.0024
177	ENSG00000253276.2	CCDC71L	0.28	0.0018	0.7	1.70E-18	0.89	4.90E-31	0.13	0.31	0.4	1.50E-06
177	ENSG00000135451.12	TROAP	0.55	0.074	0.69	0.025	1.53	4.10E-10	0.41	0.31	0.89	7.90E-04
177	ENSG00000138316.10	ADAMTS14	-0.22	0.38	0.69	3.50E-04	0.31	0.12	0.24	0.43	0.33	0.11
177	ENSG00000142156.14	COL6A1	0.57	1.70E-14	0.69	1.30E-20	0.94	5.40E-39	0.02	0.9	0.46	6.20E-10
177	ENSG00000144891.17	AGTR1	-0.89	5.10E-04	0.68	0.0048	0.14	0.64	-0.16	0.73	-0.73	0.003
177	ENSG00000168477.17	TNXB	0.23	0.3	0.68	2.00E-04	0.16	0.46	-0.26	0.35	-0.19	0.37
177	ENSG00000259863.1	SH3RF3-AS	0.45	0.062	0.68	0.0027	1.03	6.70E-08	0.57	0.026	0.74	2.90E-04
177	ENSG00000076356.6	PLXNA2	0.01	0.96	0.67	1.50E-12	0.6	1.10E-10	0.12	0.47	-0.06	0.68
177	ENSG00000102471.13	NDFIP2	0.18	0.052	0.67	4.20E-19	0.96	3.50E-41	0.19	0.083	0.34	1.90E-05
177	ENSG00000107959.15	PITRM1	0.07	0.27	0.66	7.00E-42	0.82	2.20E-66	0.03	0.77	0.2	1.40E-04
177	ENSG00000109680.10	TBC1D19	-0.06	0.68	0.66	3.20E-13	0.4	1.20E-05	-0.13	0.41	-0.16	0.14
177	ENSG00000161800.12	RACGAP1	0.49	0.0032	0.66	4.50E-05	1.35	1.10E-21	0.25	0.29	0.81	5.00E-08
177	ENSG00000169851.15	PCDH7	0.27	2.90E-04	0.66	1.70E-22	0.75	3.10E-30	0.22	0.012	0.29	3.90E-05
177	ENSG00000180914.10	OXTR	0.28	0.13	0.66	3.60E-05	1.1	3.90E-15	0.58	9.70E-04	0.62	3.90E-05
177	ENSG00000089685.14	BIRC5	0.48	0.031	0.65	0.003	1.44	6.20E-15	0.29	0.35	0.86	9.30E-06
177	ENSG00000100526.19	CDKN3	0.37	0.21	0.65	0.016	1.36	5.10E-10	0.19	0.69	0.74	0.0022
177	ENSG00000112972.14	HMGCS1	0.08	0.6	0.65	1.50E-10	0.74	1.50E-14	0.29	0.024	0.52	2.60E-07
177	ENSG00000167508.11	MVD	0.07	0.75	0.65	9.10E-06	0.71	2.20E-07	0.37	0.04	0.51	4.40E-04
177	ENSG00000014257.15	ACPP	0.19	0.47	0.64	0.0019	-0.15	0.56	0.37	0.17	0.39	0.061
177	ENSG00000115363.13	EVA1A	0.5	0.0018	0.64	3.60E-05	1.21	2.60E-19	0.46	0.012	0.76	9.80E-08
177	ENSG00000138031.14	ADCY3	-0.38	2.40E-06	0.64	4.20E-17	-0.02	0.85	-0.14	0.23	-0.59	1.80E-14
177	ENSG00000188042.7	ARL4C	-0.14	0.2	0.64	1.00E-13	0.58	3.00E-12	-0.08	0.63	-0.23	0.014
177	ENSG00000013619.13	MAMLD1	0.35	0.0044	0.63	2.10E-08	1	2.30E-22	0.58	9.40E-07	0.92	1.90E-18
177	ENSG00000100311.16	PDGFB	0.21	0.56	0.63	0.029	0.56	0.032	0.61	0.061	0.91	2.00E-04
177	ENSG00000111206.12	FOXM1	0.19	0.27	0.63	6.30E-06	1.1	2.70E-18	0.29	0.13	0.62	3.20E-06
177	ENSG00000120437.8	ACAT2	0.11	0.61	0.63	5.90E-05	0.8	3.00E-08	0.37	0.056	0.53	5.00E-04
177	ENSG00000138771.14	SHROOM3	0.06	0.68	0.63	3.00E-10	0.74	6.10E-15	0.27	0.036	0.29	0.0061
177	ENSG00000151012.13	SLC7A11	0.06	0.62	0.63	1.10E-13	-0.08	0.46	0.26	0.014	0.41	1.30E-06
177	ENSG00000151388.10	ADAMTS12	0.06	0.54	0.63	1.00E-21	0.64	1.20E-23	0.09	0.44	0.25	4.20E-04
177	ENSG00000241978.9	AKAP2	0.55	0.035	0.63	0.018	0.93	2.80E-05	0.52	0.1	0.75	0.0014
177	ENSG00000113161.15	HMGCR	0.03	0.83	0.62	2.00E-14	0.57	7.90E-13	0.24	0.021	0.32	1.60E-04
177	ENSG00000115325.13	DOK1	-0.13	0.24	0.62	2.90E-13	0.27	0.0031	-0.02	0.91	-0.16	0.13
177	ENSG00000162909.17	CAPN2	0.12	4.00E-04	0.62	1.30E-90	0.74	4.00E-128	0.11	0.011	0.16	1.60E-06
177	ENSG00000181751.9	C5orf30	0.17	0.43	0.62	1.10E-04	0.49	0.0013	0.25	0.28	0.42	0.01
177	ENSG00000059377.16	TBXAS1	0.11	0.33	0.61	4.50E-13	0.33	1.40E-04	-0.03	0.88	-0.18	0.075
177	ENSG00000134363.11	FST	-0.23	0.042	0.61	6.30E-10	0.11	0.37	0.53	3.30E-07	0.43	9.30E-06
177	ENSG00000167900.11	TK1	0.25	0.14	0.61	3.50E-05	1.05	6.60E-16	0.36	0.049	0.69	4.70E-07
177	ENSG00000188229.5	TUBB4B	0.36	2.00E-04	0.61	7.20E-11	0.94	5.10E-27	0.33	0.0032	0.61	2.00E-11
177	ENSG00000261379.1	AC010735.	0.06	0.9	0.61	0.049	0.18	0.59	-0.11	0.86	0.09	0.82
177	ENSG00000270055.1	AC127502.	0.19	0.46	0.61	0.0022	0.33	0.1	0.37	0.14	0.11	0.66
177	ENSG00000117394.20	SLC2A1	-0.03	0.83	0.6	9.90E-16	0.2	0.016	0.11	0.41	0.06	0.6
177	ENSG00000124570.17	SERPINB6	-0.24	2.70E-07	0.6	8.20E-47	0.36	1.00E-17	-0.07	0.31	-0.3	2.10E-11
177	ENSG00000130038.9	CRACR2A	0.35	0.2	0.6	0.018	0.88	3.50E-05	0.46	0.13	0.85	9.50E-05
177	ENSG00000135549.14	PKIB	0.07	0.68	0.6	5.00E-08	0.9	7.70E-19	0.03	0.91	-0.11	0.42
177	ENSG00000138829.11	FBN2	0	1	0.6	5.80E-09	0.37	3.40E-04	0.22	0.12	-0.01	0.97
177	ENSG00000143816.7	WNT9A	0.17	0.48	0.6	0.0013	0.56	0.0011	0.3	0.23	0.21	0.31
177	ENSG00000161960.14	EIF4A1	0.12	0.57	0.6	1.30E-04	0.79	1.10E-08	0.25	0.26	0.48	0.0015
177	ENSG00000172893.15	DHCR7	0.12	0.32	0.6	1.80E-10	0.67	7.40E-14	0.3	0.0092	0.53	6.10E-09
177	ENSG00000172985.10	SH3RF3	0.38	2.70E-08	0.6	2.00E-20	0.69	3.50E-28	0.26	0.0012	0.59	1.20E-19
177	ENSG00000183496.5	MEX3B	0.07	0.58	0.6	2.00E-14	0.51	5.00E-11	0.04	0.82	-0.13	0.17
177	ENSG00000185338.4	SOCS1	0.07	0.52	0.6	1.70E-16	0.49	8.90E-12	0.01	0.94	-0.09	0.35
177	ENSG00000198826.10	ARHGAP11	0.55	4.60E-04	0.6	2.00E-04	1.36	5.30E-23	0.36	0.072	0.94	4.40E-11
177	ENSG00000272622.1	AC010735.	0.08	0.8	0.6	0.0063	0.01	0.98	0.06	0.91	-0.07	0.8
177	ENSG00000101298.13	SNPH	0.05	0.69	0.59	5.30E-13	0.88	5.50E-31	0.16	0.22	0.21	0.026
177	ENSG00000124813.20	RUNX2	0.51	1.90E-08	0.59	6.80E-11	1.06	5.30E-37	0.07	0.74	0.56	2.80E-10

177	ENSG00000147155.10	EBP	0.11	0.44	0.59	3.00E-08	0.66	3.50E-11	0.2	0.2	0.32	0.0036
177	ENSG00000166387.11	PPFIBP2	-0.02	0.94	0.59	8.60E-04	1.26	1.10E-17	0.05	0.9	0.16	0.44
177	ENSG00000187796.14	CARD9	0.16	0.58	0.59	0.0096	0.14	0.6	0.28	0.4	0.35	0.12
177	ENSG00000262454.3	MIR193BH	-0.23	0.45	0.59	0.015	-0.19	0.49	-0.03	0.95	-0.42	0.095
177	ENSG00000275342.4	PRAG1	0.04	0.81	0.59	1.60E-09	0.61	1.10E-10	0.19	0.17	0.14	0.24
36	ENSG00000163435.15	ELF3	0.9	-0.07	0.84	2.88	4.88	0.085	0.96	0.078	5.80E-09	4.80E-26
36	ENSG00000144802.11	NFKBIZ	0.58	0.36	1.04	2.22	2.58	2.30E-05	0.023	7.50E-17	3.00E-75	3.20E-103
36	ENSG00000163874.10	ZC3H12A	0.49	0.56	1.15	2.1	2.94	7.90E-05	7.60E-06	2.80E-26	3.90E-88	4.50E-180
36	ENSG00000078081.7	LAMP3	0.36	0.42	0.33	1.55	1.74	0.55	0.53	0.49	0.011	8.10E-04
36	ENSG00000021645.18	NRXN3	0.62	0.13	0.48	1.45	1.46	0.18	0.85	0.26	8.30E-04	1.20E-04
36	ENSG00000081041.8	CXCL2	0.68	0.28	0.79	1.18	3.26	0.11	0.64	0.03	0.005	1.20E-22
36	ENSG00000128965.11	CHAC1	0.53	0.5	0.69	0.98	1.2	0.0043	0.012	4.00E-05	3.10E-08	5.20E-14
36	ENSG00000104450.12	SPAG1	0.46	0.45	0.67	0.86	0.91	0.16	0.21	0.014	0.0062	5.70E-04
36	ENSG00000175536.6	LIPT2	0.57	0.53	0.2	0.86	0.67	0.13	0.21	0.65	0.026	0.05
36	ENSG00000113578.17	FGF1	0.32	0.58	0.98	0.85	0.99	0.19	0.0097	1.90E-07	8.80E-05	1.90E-07
36	ENSG00000137331.11	IER3	0.38	0.32	0.81	0.83	1.03	0.0024	0.018	1.30E-13	1.10E-12	1.40E-21
36	ENSG00000111859.16	NEDD9	0.37	0.18	0.46	0.81	0.91	0.011	0.37	5.70E-04	1.90E-09	2.00E-13
36	ENSG00000259426.5	AC027237.	0.28	0.58	0.21	0.81	0.89	0.34	0.027	0.45	0.0017	5.10E-05
36	ENSG00000103888.16	CEMIP	0.16	0.18	0.15	0.8	0.85	0.65	0.68	0.64	0.0055	4.80E-04
36	ENSG00000280143.1	AP000892.	0.32	0.36	0.52	0.79	0.9	0.21	0.19	0.015	7.50E-04	7.50E-06
36	ENSG00000213066.11	FGFR1OP	-0.01	0.13	-0.01	0.78	0.85	0.98	0.56	0.96	1.20E-08	9.70E-12
36	ENSG00000260910.1	LINC00565	0.41	0.13	0.3	0.78	0.78	0.2	0.8	0.33	0.012	0.0028
36	ENSG00000273117.1	AC144652.	0.25	0.49	0.21	0.78	0.27	0.47	0.13	0.5	0.0092	0.39
36	ENSG00000143473.12	KCNH1	-0.15	0.45	0.32	0.76	0.71	0.59	0.04	0.12	1.40E-04	8.00E-05
36	ENSG00000120262.9	CCDC170	-0.45	-0.42	-0.38	0.75	0.09	0.25	0.37	0.32	0.041	0.85
36	ENSG00000147852.15	VLDLR	0.43	0.27	-0.21	0.74	0.62	0.05	0.33	0.39	4.10E-04	0.0011
36	ENSG00000163431.12	LMOD1	0.08	0.29	-0.2	0.73	0.35	0.71	0.1	0.22	5.10E-07	0.016
36	ENSG00000174749.5	C4orf32	0.35	0.25	1.17	0.67	0.91	0.23	0.49	2.80E-08	0.017	4.90E-05
36	ENSG00000139211.6	AMIGO2	0.53	0.25	0.72	0.66	1.11	6.80E-05	0.14	3.40E-09	1.40E-06	2.00E-20
36	ENSG00000170775.2	GPR37	0.14	0.23	0.05	0.64	0.52	0.24	0.048	0.72	8.80E-11	4.50E-08
36	ENSG00000210195.2	MT-TT	-0.41	0.34	0.3	0.63	0.97	0.15	0.28	0.25	0.024	7.00E-06
36	ENSG00000133111.3	RFXAP	-0.26	0.46	0.48	0.62	0.65	0.29	0.035	0.011	0.0043	4.30E-04
36	ENSG00000272695.1	GAS6-AS2	0.42	0.29	0.44	0.62	0.69	0.0091	0.13	0.0034	1.80E-04	1.40E-06
36	ENSG00000114019.14	AMOTL2	0.25	0.47	0.72	0.61	0.72	0.021	1.90E-06	1.20E-15	7.30E-10	1.70E-15
36	ENSG00000130176.7	CNN1	-0.03	0.16	0.24	0.61	0.49	0.93	0.59	0.27	0.0046	0.01
36	ENSG00000225614.2	ZNF469	0.21	0.38	0.4	0.61	0.68	0.032	3.60E-05	1.60E-06	3.80E-12	6.60E-17
36	ENSG00000276107.1	AC037198.	-0.65	0.18	-0.06	0.6	0.26	0.0033	0.56	0.84	0.0074	0.24
36	ENSG00000163017.13	ACTG2	0.02	0.22	0.15	0.59	0.49	0.94	0.33	0.46	8.30E-04	0.0019
36	ENSG00000172379.20	ARNT2	0.28	-0.19	0.18	0.59	0.71	0.12	0.41	0.33	5.10E-04	1.10E-06
36	ENSG00000180611.6	MB21D2	0.41	0.35	0.84	0.59	0.94	0.015	0.064	4.10E-09	6.20E-04	4.50E-11
36	ENSG00000185022.11	MAFF	0.55	0.33	0.87	0.59	0.86	2.10E-07	0.0056	1.10E-18	9.50E-08	6.60E-18
94	ENSG00000005102.12	MEOX1	1.45	1.99	3.88	0.47	0.71	0.0093	0.0016	3.50E-08	0.4	0.11
94	ENSG00000011426.10	ANLN	0.99	0.92	2.01	0.54	1.4	5.00E-07	6.60E-06	3.00E-28	0.03	7.80E-14
94	ENSG00000013810.18	TACC3	0.77	0.77	1.68	0.47	1.21	1.80E-07	2.50E-07	7.80E-36	0.0092	1.50E-18
94	ENSG00000051341.13	POLQ	1.3	0.73	2.07	0.49	1.81	2.40E-07	0.013	2.20E-19	0.19	1.40E-14
94	ENSG00000066279.17	ASPM	0.97	1.04	1.94	0.43	1.27	1.60E-06	3.20E-07	1.20E-25	0.13	4.60E-11
94	ENSG00000071539.13	TRIP13	0.88	0.79	1.6	0.46	1.26	1.30E-06	3.70E-05	6.20E-22	0.055	2.10E-13
94	ENSG00000072571.19	HMMR	0.89	0.69	1.77	0.1	1.13	2.30E-05	0.0027	3.60E-21	0.84	1.30E-08
94	ENSG00000075218.18	GTSE1	1.08	1.03	2.09	0.51	1.51	8.60E-07	4.90E-06	3.80E-26	0.082	1.90E-13
94	ENSG00000076382.16	SPAG5	0.94	0.93	1.87	0.56	1.33	1.50E-04	3.00E-04	4.20E-17	0.082	8.50E-09
94	ENSG00000082438.15	COBLL1	1.42	1.65	2.4	0.87	1.7	1.60E-04	1.00E-05	2.80E-12	0.065	1.70E-06
94	ENSG00000085999.11	RAD54L	1.29	0.83	2.17	0.46	1.49	3.70E-08	0.0015	2.50E-24	0.19	3.10E-11
94	ENSG00000090889.11	KIF4A	0.82	0.83	1.7	0.49	1.32	5.60E-06	8.40E-06	1.50E-25	0.031	3.70E-15
94	ENSG00000092853.13	CLSPN	0.79	0.69	1.66	0.5	1.34	3.90E-06	1.10E-04	1.20E-27	0.017	1.30E-17
94	ENSG00000100078.3	PLA2G3	1.09	1.39	2	0.85	1.37	0.0031	1.10E-04	3.40E-10	0.059	5.50E-05
94	ENSG00000100162.14	CENPM	0.81	0.68	1.36	0.56	0.99	0.0031	0.021	1.30E-08	0.11	9.70E-05
94	ENSG00000100342.20	APOL1	0.96	0.6	1.26	0.11	1.08	5.90E-06	0.014	7.90E-11	0.82	8.80E-08
94	ENSG00000101003.9	GINS1	0.82	0.86	1.76	0.51	1.19	4.80E-05	2.60E-05	2.70E-23	0.048	1.50E-10
94	ENSG00000102384.13	CENPI	0.59	0.79	1.69	0.39	1	0.0075	2.30E-04	1.20E-20	0.19	3.40E-07
94	ENSG00000102468.10	HTR2A	0.88	0.81	1.86	0.27	1.35	4.40E-06	5.20E-05	7.60E-29	0.4	1.30E-14
94	ENSG00000102967.11	DHODH	0.81	0.66	1.08	0.47	1.05	1.60E-05	0.001	2.60E-10	0.056	2.00E-09
94	ENSG00000104147.8	OIP5	0.87	1.06	1.82	0.33	1.43	0.023	0.0053	1.40E-08	0.61	1.90E-05
94	ENSG00000109674.3	NEIL3	1.12	1.19	1.91	0.56	1.63	0.0036	0.0027	2.10E-08	0.31	3.20E-06
94	ENSG00000111247.14	RAD51AP1	1.14	0.89	1.74	0.33	1.48	1.20E-06	4.20E-04	8.40E-16	0.4	3.00E-11
94	ENSG00000111728.10	ST8SIA1	0.84	2.62	3.11	0.43	0.7	1.40E-10	1.10E-116	7.40E-168	0.0092	8.70E-08
94	ENSG00000112297.14	CRYBG1	0.78	1.67	2.66	0.43	1.07	4.70E-17	5.20E-84	1.20E-222	1.00E-04	4.70E-33
94	ENSG00000112742.9	TTK	1.15	0.82	2.09	0.52	1.45	2.10E-07	7.50E-04	4.60E-25	0.085	5.60E-12
94	ENSG00000117399.13	CDC20	0.73	0.84	1.62	0.51	1.18	6.40E-04	1.10E-04	1.20E-17	0.057	1.90E-09
94	ENSG00000117650.12	NEK2	0.89	0.87	2.07	0.61	1.2	0.0033	0.0061	1.50E-15	0.12	1.70E-05
94	ENSG00000117724.12	CENPF	0.68	0.8	1.78	0.38	1.13	3.10E-04	2.40E-05	2.40E-26	0.14	8.10E-11
94	ENSG00000117877.10	CD3EAP	0.83	0.63	1.34	0.52	1.19	1.60E-07	2.60E-04	3.10E-20	0.0082	1.10E-15
94	ENSG00000118193.11	KIF14	0.99	0.98	2.17	0.26	1.44	3.10E-05	5.20E-05	3.00E-25	0.53	4.70E-11
94	ENSG00000119969.14	HELLS	0.81	0.64	1.12	0.26	0.88	4.30E-10	3.10E-06	1.50E-20	0.18	2.60E-12
94	ENSG00000121621.6	KIF18A	1	0.72	1.57	0.39	1.27	1.10E-05	0.0045	3.50E-14	0.26	3.00E-09
94	ENSG00000122952.16	ZWINT	0.89	0.72	1.65	0.44	1.2	1.30E-04	0.0045	4.40E-15	0.17	4.40E-08
94	ENSG00000123219.12	CENPK	0.78	0.61	1.28	0.22	0.98	1.80E-04	0.0078	3.90E-12	0.54	4.30E-07
94	ENSG00000123485.11	HJURP	1.11	1.21	2.12	0.6	1.57	3.70E-05	1.00E-05	5.10E-18	0.09	5.50E-10
94	ENSG00000123496.7	IL13RA2	1.28	1.93	3.37	-0.04	0.55	2.70E-04	5.40E-09	6.10E-29	0.97	0.17
94	ENSG00000126787.12	DLGAP5	0.8	0.93	1.84	0.45	1.11	4.20E-05	1.60E-06	2.80E-26	0.076	9.80E-10
94	ENSG00000129195.15	PIMREG	0.89	0.88	1.85	0.39	1.31	2.90E-04	5.10E-04	1.20E-17	0.28	6.00E-09

94	ENSG00000132530.16	XAF1	0.84	0.65	1.01	0.42	1.07	0.0016	0.025	2.60E-05	0.27	1.20E-05
94	ENSG00000133816.13	MICAL2	0.86	0.86	1.67	0.49	1.27	7.30E-26	9.50E-26	2.30E-100	6.30E-08	1.30E-57
94	ENSG00000133985.2	TTC9	2.72	1.24	3.66	0.54	2.88	8.10E-33	1.40E-06	1.50E-61	0.14	2.30E-37
94	ENSG00000134057.14	CCNB1	0.63	0.76	1.59	0.37	0.84	0.0016	1.30E-04	8.30E-20	0.17	6.10E-06
94	ENSG00000134470.20	IL15RA	2.05	0.66	2.2	0.33	2.37	1.20E-19	0.023	7.30E-24	0.44	3.40E-27
94	ENSG00000134690.10	CDCA8	1.1	0.95	2.3	0.39	1.66	8.00E-07	4.60E-05	2.60E-30	0.25	1.20E-15
94	ENSG00000135373.12	EHF	1.14	1.01	3.72	0.77	3.6	0.012	0.033	1.90E-18	0.17	3.80E-17
94	ENSG00000135476.11	ESPL1	0.94	0.96	1.79	0.39	1.29	3.10E-05	2.70E-05	2.40E-19	0.25	6.00E-10
94	ENSG00000137310.11	TCF19	0.81	0.6	1.38	0.36	1.13	1.50E-07	3.50E-04	2.10E-22	0.085	7.10E-15
94	ENSG00000137804.12	NUSAP1	0.8	0.97	1.84	0.39	1.28	3.60E-05	4.20E-07	1.20E-26	0.14	8.80E-13
94	ENSG00000137807.13	KIF23	0.94	0.78	1.72	0.58	1.35	1.20E-06	1.60E-04	4.10E-22	0.016	1.60E-13
94	ENSG00000137812.19	KNL1	0.94	0.89	1.88	0.55	1.28	3.60E-06	2.20E-05	7.80E-25	0.033	2.40E-11
94	ENSG00000138180.15	CEP55	0.85	0.92	1.96	0.41	1.26	0.0023	0.0014	8.80E-16	0.31	8.80E-07
94	ENSG00000138182.14	KIF20B	0.6	0.61	1.46	0.22	0.97	2.10E-04	2.50E-04	9.40E-25	0.39	5.10E-11
94	ENSG00000138778.11	CENPE	0.85	0.87	1.81	0.42	1.15	9.20E-04	0.001	8.80E-16	0.25	1.20E-06
94	ENSG00000141384.12	TAF4B	1.44	0.75	1.69	0.4	1.5	1.60E-11	0.0028	3.60E-17	0.25	4.20E-13
94	ENSG00000142731.10	PLK4	0.92	0.64	1.78	0.21	1.28	3.20E-06	0.0041	1.90E-23	0.56	4.90E-12
94	ENSG00000142945.12	KIF2C	0.8	0.88	1.96	0.54	1.4	2.80E-05	4.10E-06	1.50E-31	0.02	8.40E-16
94	ENSG00000143228.12	NUF2	1.24	1.41	2.47	0.51	1.6	8.60E-06	4.20E-07	6.70E-23	0.21	1.30E-09
94	ENSG00000144554.10	FANCD2	1	0.89	1.78	0.53	1.5	9.90E-08	3.90E-06	1.50E-25	0.028	1.00E-17
94	ENSG00000146197.8	SCUBE3	1.3	2.09	4.04	0.34	0.92	3.00E-11	6.80E-30	1.10E-117	0.28	4.60E-06
94	ENSG00000148773.13	MKI67	0.94	1.13	2.22	0.55	1.57	2.20E-05	3.70E-07	2.30E-28	0.05	2.60E-14
94	ENSG00000150551.10	LYPD1	0.7	0.89	1.81	0.23	0.79	2.80E-10	8.80E-17	4.00E-75	0.17	2.20E-13
94	ENSG00000151651.15	ADAM8	0.75	1.22	2.71	0.26	1.17	1.10E-06	2.00E-17	3.00E-96	0.27	1.40E-16
94	ENSG00000154639.18	CXADR	1.19	0.91	1.7	0.28	1.07	1.80E-07	2.10E-04	5.70E-16	0.5	2.20E-06
94	ENSG00000160957.12	RECQL4	0.61	0.61	1.06	0.32	0.83	7.30E-06	1.20E-05	4.50E-18	0.08	6.50E-11
94	ENSG00000161888.11	SPC24	0.86	0.7	1.36	0.45	0.91	1.90E-05	0.0012	4.80E-14	0.099	2.50E-06
94	ENSG00000162692.10	VCAM1	1.4	1.48	2.1	-0.03	1.32	4.80E-18	5.30E-20	5.00E-41	0.95	2.90E-16
94	ENSG00000163661.3	PTX3	0.59	0.91	1.2	0.49	0.96	4.20E-10	6.60E-23	2.30E-41	2.50E-06	3.40E-26
94	ENSG00000163808.16	KIF15	0.81	0.64	1.55	0.28	1.02	1.20E-04	0.0053	9.40E-17	0.4	2.60E-07
94	ENSG00000164109.13	MAD2L1	0.9	0.69	1.63	0.37	1.22	4.00E-08	7.30E-05	9.60E-28	0.11	3.00E-15
94	ENSG00000165304.7	MELK	0.64	0.62	1.5	0.39	1.15	4.30E-05	1.20E-04	2.20E-27	0.057	7.10E-16
94	ENSG00000165480.15	SKA3	1.1	0.93	2.17	0.58	1.47	2.40E-05	7.80E-04	1.20E-20	0.099	1.40E-09
94	ENSG00000168496.3	FEN1	1.1	0.69	1.8	0.54	1.44	2.20E-11	1.30E-04	3.20E-31	0.0086	9.80E-20
94	ENSG00000169116.11	PARM1	0.79	0.65	0.3	0.42	0.48	0.0086	0.05	0.37	0.33	0.13
94	ENSG00000169271.2	HSPB3	0.97	1.56	3.01	0.76	1.82	0.023	1.00E-04	7.50E-17	0.15	1.10E-06
94	ENSG00000169679.14	BUB1	0.73	0.8	1.86	0.14	1.18	7.80E-04	2.60E-04	2.70E-23	0.75	2.10E-09
94	ENSG00000170312.15	CDK1	1.2	0.97	2.15	0.64	1.61	6.90E-06	6.50E-04	1.00E-18	0.067	1.50E-10
94	ENSG00000171223.5	JUNB	0.8	0.99	1.24	0.36	1.07	1.70E-06	2.90E-09	2.70E-15	0.12	2.00E-11
94	ENSG00000176170.13	SPHK1	0.8	1.14	2.07	0.2	0.56	1.60E-12	1.10E-25	3.70E-89	0.25	1.30E-06
94	ENSG00000177602.5	HASPIN	0.85	0.66	1.78	0.01	1.34	0.0035	0.043	2.90E-13	0.99	2.00E-07
94	ENSG00000179242.15	CDH4	0.62	0.67	0.79	0.48	0.93	8.50E-06	1.30E-06	8.20E-10	0.0026	7.50E-13
94	ENSG00000179750.15	APOBEC3B	0.64	0.61	1.62	0.29	1.08	0.0037	0.0097	7.50E-18	0.38	5.20E-08
94	ENSG00000183856.10	IQGAP3	0.81	0.95	1.86	0.54	1.18	2.10E-04	1.50E-05	1.10E-21	0.048	7.20E-09
94	ENSG00000184661.13	CDCA2	0.76	1.04	1.99	0.51	1.35	6.70E-04	1.10E-06	1.20E-25	0.07	1.00E-11
94	ENSG00000186185.13	KIF18B	1.24	1.17	2.46	0.6	1.92	1.50E-07	1.10E-06	3.00E-31	0.058	1.50E-18
94	ENSG00000186638.16	KIF24	0.64	0.6	1.14	0.15	1.07	0.022	0.046	1.40E-06	0.78	1.10E-05
94	ENSG00000189057.10	FAM111B	1.66	0.96	2.46	0.52	1.92	4.30E-10	0.0014	1.20E-22	0.19	9.40E-14
94	ENSG00000189410.11	SH2D5	1.32	0.78	2.12	0.58	1.86	2.30E-06	0.017	8.60E-17	0.14	1.20E-12
94	ENSG00000197142.10	ACSL5	0.96	0.69	1.69	0.39	1	4.10E-04	0.024	5.10E-13	0.36	1.10E-04
94	ENSG00000198885.9	ITPRIPL1	0.74	0.7	0.85	0.55	0.85	4.80E-04	0.0016	1.50E-05	0.034	2.20E-05
94	ENSG00000224413.1	AP001476.1	1.01	1.63	1.9	0.49	0.63	0.0081	4.90E-06	8.30E-09	0.39	0.11
94	ENSG00000241644.2	INMT	0.62	0.85	0.86	0.58	0.67	7.20E-04	1.90E-06	2.30E-07	0.0059	1.40E-04
94	ENSG00000248927.1	AC114284.1	0.59	0.76	1.25	-0.07	0.28	0.016	0.0016	7.60E-10	0.9	0.3
94	ENSG00000276043.4	UHRF1	0.9	0.65	1.52	0.5	1.35	7.00E-12	3.00E-06	3.40E-34	0.0018	6.20E-27
41	ENSG00000163739.4	CXCL1	1.41	-0.83	0.28	2.78	5.32	2.30E-11	0.0026	0.28	1.90E-45	3.10E-175
41	ENSG00000124875.9	CXCL6	2.12	-0.07	1.21	2.65	5.83	3.10E-09	0.94	6.40E-04	6.80E-13	2.40E-63
41	ENSG00000125730.16	C3	2.46	0.09	2.66	2.48	4.67	2.40E-15	0.88	1.70E-18	2.50E-14	9.30E-55
41	ENSG00000157368.10	IL34	4.95	-0.31	4.59	1.82	5.04	5.60E-60	0.6	1.80E-52	6.90E-08	1.50E-62
41	ENSG00000169429.10	CXCL8	0.79	0.01	-0.29	1.64	3.36	0.022	0.98	0.48	4.10E-07	4.20E-32
41	ENSG00000166863.11	TAC3	4.54	-0.53	4.65	1.34	4.79	2.50E-47	0.28	3.90E-50	2.70E-04	8.90E-53
41	ENSG00000104951.15	IL4I1	3.27	0.27	3.82	1.31	3.68	3.10E-21	0.65	3.80E-29	0.0017	6.30E-27
41	ENSG00000135604.9	STX11	1.39	0.5	1.68	1.3	1.65	5.80E-04	0.35	7.80E-06	0.0045	1.70E-05
41	ENSG00000163131.10	CTSS	2.87	-0.13	2.93	1.23	3.25	5.20E-36	0.8	4.50E-38	4.60E-06	2.50E-46
41	ENSG00000163347.5	CLDN1	2.29	-0.09	1.42	1.21	3.02	4.30E-57	0.82	1.80E-21	2.30E-13	8.90E-105
41	ENSG00000163735.6	CXCL5	1.38	-0.6	1.11	1.17	3.35	1.80E-06	0.15	8.80E-05	3.60E-04	1.40E-37
41	ENSG00000023445.13	BIRC3	4.34	-0.07	4.61	1.09	4.82	5.90E-45	0.93	6.90E-51	0.005	2.80E-55
41	ENSG00000177272.8	KCNA3	1.72	0.53	1.54	1.06	2.15	3.40E-19	0.038	4.90E-16	1.60E-06	2.70E-31
41	ENSG00000181634.7	TNFSF15	2.86	-1.48	0.51	1.02	2.93	2.10E-25	4.00E-04	0.13	0.0045	6.00E-27
41	ENSG00000099958.14	DERL3	0.9	0.38	1.33	0.94	1.65	0.013	0.46	2.60E-05	0.022	1.50E-07
41	ENSG00000107968.9	MAP3K8	1.16	-0.24	0.66	0.91	1.41	2.20E-04	0.68	0.04	0.015	1.30E-06
41	ENSG00000028277.21	POU2F2	1.13	-0.11	1.22	0.87	2.52	6.30E-15	0.71	2.60E-18	3.70E-08	1.60E-81
41	ENSG00000048052.21	HDAC9	1.98	0.42	2.53	0.86	2.21	1.70E-17	0.24	7.00E-30	0.0041	2.40E-22
41	ENSG00000167080.8	B4GALNT2	0.78	0.51	0.56	0.85	1.22	0.029	0.25	0.12	0.034	9.20E-05
41	ENSG00000166592.11	RRAD	2.21	0.02	2.33	0.8	2.54	8.90E-74	0.94	3.80E-83	2.40E-08	1.40E-99
41	ENSG00000241749.4	RPSAP52	0.63	0.43	1.2	0.79	1.24	0.038	0.24	1.40E-06	0.014	9.30E-07
41	ENSG00000008517.16	IL32	3.36	-0.02	4.02	0.72	3.59	2.70E-55	0.99	8.20E-80	0.015	2.40E-63
41	ENSG00000113070.7	HBEGF	1.58	0.51	2.41	0.71	2.27	5.60E-20	0.021	2.60E-49	0.0012	4.70E-43
41	ENSG00000106688.11	SLC1A1	1.16	0.47	0.95	0.71	1.32	1.00E-08	0.071	1.90E-06	0.0046	1.20E-11
41	ENSG00000117586.10	TNFSF4	1.82	-0.05	1.86	0.7	2.17	6.50E-114	0.78	1.90E-121	7.90E-14	4.20E-167

41	ENSG00000155324.9	GRAMD2B	0.69	0.2	0.98	0.7	1.23	1.30E-05	0.39	1.10E-11	4.20E-05	5.80E-18
41	ENSG00000104415.13	WISP1	1.34	-0.14	0.28	0.69	1.56	2.20E-08	0.79	0.35	0.03	1.00E-11
41	ENSG00000171522.5	PTGER4	0.73	0.48	1.38	0.68	1.16	9.90E-11	9.40E-05	1.50E-40	1.60E-08	3.80E-28
41	ENSG00000101412.12	E2F1	1.14	0.55	1.68	0.67	1.53	1.00E-10	0.0074	3.60E-24	0.0015	8.00E-20
41	ENSG00000151892.14	GFRA1	0.84	-0.15	0.54	0.67	1.58	3.20E-05	0.71	0.0078	0.0048	3.30E-19
41	ENSG00000039139.9	DNAH5	0.81	-0.17	0.07	0.67	1.09	2.40E-13	0.31	0.69	1.90E-08	1.40E-24
41	ENSG00000072274.12	TFRC	0.64	0.34	0.66	0.67	1.16	9.90E-13	0.00093	1.70E-14	6.10E-13	2.90E-43
41	ENSG00000164023.14	SGMS2	0.71	0.54	1.01	0.65	0.88	5.00E-06	0.0013	1.50E-12	1.30E-04	1.80E-09
41	ENSG00000090530.9	P3H2	1.16	0.21	1.25	0.63	1.83	3.40E-23	0.25	1.70E-28	4.90E-06	9.40E-63
41	ENSG00000198796.6	ALPK2	1.12	0.18	1.11	0.63	1.72	2.40E-29	0.21	1.00E-29	1.60E-08	5.50E-72
41	ENSG00000148154.9	UGCG	1.01	0.47	1.33	0.62	1.44	2.80E-25	1.20E-05	2.60E-44	1.30E-08	3.40E-52
41	ENSG00000250899.3	AC125807.2	0.85	0.42	0.85	0.61	1.05	3.60E-05	0.1	1.10E-05	0.015	4.30E-08
41	ENSG00000104635.13	SLC39A14	0.79	0.36	1.01	0.61	1.49	4.10E-36	3.50E-07	3.50E-61	7.90E-20	1.10E-136
41	ENSG00000165507.8	C10orf10	1.05	-0.54	0.22	0.6	1.31	5.20E-10	0.0085	0.29	0.0031	5.30E-16
41	ENSG00000179431.6	FJX1	1.01	0.38	1.22	0.6	1.29	5.40E-21	0.0027	1.50E-31	9.00E-07	9.00E-35
41	ENSG00000115828.15	QPCT	0.78	0.01	0.88	0.59	1.19	1.60E-04	0.99	3.80E-06	0.016	1.40E-10
54	ENSG00000182261.3	NLRP10	2.28	2.83	3.91	2.16	3.03	6.20E-05	3.50E-06	2.90E-10	5.10E-04	2.80E-07
54	ENSG00000183691.4	NOG	0.92	1.22	1.94	1.3	1.55	0.027	0.0026	2.70E-08	0.0025	2.00E-05
54	ENSG00000137573.13	SULF1	1.31	2.63	3.61	1.29	2.29	5.40E-05	8.40E-18	1.30E-33	2.60E-04	3.80E-14
54	ENSG00000128165.8	ADM2	0.79	0.75	0.77	1.14	1.35	0.008	0.017	0.0057	1.80E-04	2.60E-07
54	ENSG00000185697.16	MYBL1	3.51	1.12	4.4	1.1	3.9	4.40E-80	7.10E-08	2.70E-127	5.30E-07	1.40E-99
54	ENSG00000168389.17	MFSD2A	1.49	0.78	2.01	1.06	2.32	2.30E-08	0.013	4.00E-16	7.70E-04	6.50E-21
54	ENSG00000128917.6	DLL4	1.36	1.63	1.74	1.02	2.13	4.70E-05	7.80E-07	1.80E-08	0.011	3.30E-12
54	ENSG00000129810.14	SGO1	1.36	1.12	2.16	1	1.58	1.10E-04	0.0029	1.40E-11	0.018	2.00E-06
54	ENSG00000115163.14	CENPA	1.58	1.11	2.47	0.99	1.71	5.70E-07	0.0013	2.70E-17	0.011	1.90E-08
54	ENSG00000140534.13	TICRR	1.15	1.24	2.25	0.96	2.01	8.10E-05	2.60E-05	3.90E-18	0.0042	2.60E-14
54	ENSG00000154839.9	SKA1	1.42	1.14	2.47	0.92	1.73	7.30E-07	1.70E-04	3.40E-21	0.0086	1.70E-10
54	ENSG00000174371.16	EXO1	1.57	1.12	2.22	0.92	1.98	1.10E-12	2.20E-06	2.10E-26	5.20E-04	1.40E-20
54	ENSG00000186871.6	ERCC6L	1.73	1.31	2.65	0.91	2.15	9.40E-09	4.70E-05	6.20E-21	0.017	1.10E-13
54	ENSG00000148677.6	ANKRD1	1.56	1.06	2.5	0.9	2.12	4.90E-08	6.40E-04	4.30E-21	0.01	4.50E-15
54	ENSG00000171320.14	ESCO2	1.59	1.04	2.44	0.9	2.09	4.70E-06	0.0071	3.10E-14	0.041	2.10E-10
54	ENSG00000175063.16	UBE2C	1.19	1.31	2.25	0.89	1.71	9.90E-07	9.00E-08	7.90E-24	0.0017	8.30E-14
54	ENSG00000151725.11	CENPU	1.51	1.17	2.1	0.88	1.68	1.20E-16	6.20E-10	4.70E-34	3.80E-05	3.90E-21
54	ENSG00000237649.7	KIFC1	1.28	1.26	2.41	0.86	1.85	2.00E-08	5.90E-08	4.60E-30	0.0013	1.40E-17
54	ENSG00000101057.15	MYBL2	1.11	1.13	2.17	0.85	1.84	2.70E-06	2.70E-06	1.50E-23	0.0022	7.20E-17
54	ENSG00000065328.16	MCM10	1.35	1.16	2.36	0.8	2.03	1.30E-08	3.10E-06	3.20E-27	0.0062	7.80E-20
54	ENSG00000121152.9	NCAPH	1.14	1.09	2.18	0.8	1.71	2.30E-06	1.20E-05	3.20E-23	0.0062	3.00E-14
54	ENSG00000260196.1	AC124798.1	0.69	0.79	1.32	0.8	1.49	0.038	0.019	1.50E-06	0.029	5.60E-08
54	ENSG00000145147.19	SLIT2	1.95	1.13	3.44	0.79	2.11	3.00E-92	1.20E-29	3.50E-303	4.00E-13	3.20E-109
54	ENSG00000166741.7	NNMT	0.86	0.93	1.79	0.79	1.93	2.90E-07	2.70E-08	1.90E-34	1.70E-05	1.30E-39
54	ENSG00000113361.12	CDH6	0.93	3.18	3.86	0.78	1.06	3.60E-06	2.30E-73	1.20E-109	5.60E-04	2.60E-08
54	ENSG00000029153.14	ARNTL2	2.29	1.29	3.5	0.77	2.73	3.80E-82	3.20E-25	1.00E-195	3.40E-08	1.60E-117
54	ENSG00000024526.16	DEPDC1	1.59	1.27	2.45	0.77	1.89	1.00E-16	1.70E-10	3.10E-42	0.0013	2.20E-24
54	ENSG00000102554.13	KLF5	0.91	0.59	1.84	0.77	1.87	2.00E-06	0.0069	2.10E-27	4.00E-04	8.90E-28
54	ENSG00000143476.17	DTL	1.32	0.87	2.15	0.76	1.85	8.00E-09	5.10E-04	7.10E-24	0.0065	1.60E-17
54	ENSG00000152253.8	SPC25	0.88	0.92	2.03	0.75	1.4	0.005	0.0045	1.50E-14	0.046	4.90E-07
54	ENSG00000127564.16	PKMYT1	1.45	0.86	2.11	0.75	1.89	9.50E-15	2.70E-05	5.70E-33	0.001	4.20E-26
54	ENSG00000168078.9	PBK	1.03	0.93	2.06	0.74	1.48	2.00E-06	3.50E-05	4.10E-26	0.0041	2.20E-13
54	ENSG00000139514.12	SLC7A1	0.88	0.72	1.37	0.74	1.42	5.70E-18	4.90E-12	4.40E-45	6.40E-12	2.40E-48
54	ENSG00000197646.7	PDCD1LG2	0.98	1.23	2.24	0.72	1.58	4.00E-08	9.40E-13	4.70E-46	5.10E-04	5.50E-22
54	ENSG00000175305.17	CCNE2	1.29	0.88	2	0.72	1.69	2.90E-06	0.0043	3.10E-15	0.044	9.80E-11
54	ENSG00000105011.8	ASF1B	1.28	0.79	2.2	0.72	1.77	4.70E-06	0.014	7.80E-18	0.043	1.80E-11
54	ENSG00000171241.8	SHCBP1	1.2	1.09	2.13	0.7	1.76	3.00E-09	1.50E-07	4.30E-30	0.0047	3.40E-20
54	ENSG00000094804.9	CDC6	1.55	1.05	2.22	0.7	2.06	7.20E-25	3.20E-11	1.60E-53	1.30E-04	2.70E-45
54	ENSG00000093009.9	CDC45	1.1	0.94	1.98	0.7	1.72	1.20E-06	9.80E-05	6.50E-22	0.013	3.30E-16
54	ENSG00000176208.8	ATAD5	1.11	0.82	1.9	0.7	1.33	8.30E-06	0.0026	2.20E-17	0.025	1.60E-08
54	ENSG00000169607.12	CKAP2L	1.14	1.05	2.15	0.69	1.65	1.30E-06	1.40E-05	4.20E-24	0.018	4.30E-14
54	ENSG00000127666.9	TICAM1	0.62	0.6	1.08	0.69	1.05	6.50E-11	3.80E-10	2.60E-35	1.60E-12	6.20E-33
54	ENSG00000091972.18	CD200	0.61	1.29	1.37	0.68	0.87	0.027	4.10E-08	5.90E-10	0.025	3.40E-04
54	ENSG00000131747.14	TOP2A	1.14	1.16	2.2	0.68	1.53	1.80E-07	1.60E-07	3.70E-27	0.011	2.50E-13
54	ENSG00000151014.5	NOCT	0.94	0.76	1.56	0.67	1.82	5.40E-07	1.20E-04	1.90E-20	0.0026	2.00E-27
54	ENSG00000156970.12	BUB1B	1.12	1.15	2.18	0.66	1.54	6.90E-09	2.90E-09	4.10E-35	0.0055	3.20E-17
54	ENSG00000051180.16	RAD51	0.88	0.67	1.55	0.65	1.24	2.30E-06	8.10E-04	1.40E-20	0.0027	8.00E-13
54	ENSG00000080986.12	NDC80	1.23	1.19	2.15	0.64	1.61	3.90E-09	1.90E-08	4.60E-29	0.018	4.80E-16
54	ENSG00000101447.14	FAM83D	0.93	1.09	2.25	0.63	1.61	4.90E-05	1.40E-06	7.60E-30	0.025	4.80E-15
54	ENSG00000165244.6	ZNF367	1.6	0.98	2.19	0.63	1.95	3.80E-13	5.60E-05	5.60E-26	0.036	3.60E-20
54	ENSG00000139734.17	DIAPH3	0.88	0.84	1.48	0.63	1.33	1.30E-05	6.20E-05	1.20E-15	0.0086	2.00E-12
54	ENSG00000109805.9	NCAPG	1.02	0.97	1.94	0.61	1.41	2.80E-05	1.30E-04	2.30E-18	0.049	7.10E-10
54	ENSG00000171848.14	RRM2	0.96	0.82	1.96	0.61	1.51	1.10E-05	4.10E-04	6.30E-23	0.024	9.60E-14
54	ENSG00000198901.13	PRC1	0.85	0.93	1.9	0.6	1.29	1.10E-05	2.00E-06	3.40E-27	0.01	1.20E-12
14	ENSG00000006606.8	CCL26	0.15	6.63	7.12	2.5	1.43	0.8	7.70E-15	2.30E-17	0.0013	0.015
14	ENSG00000118785.13	SPP1	0.9	3.5	3.66	1.51	0.92	0.072	3.30E-14	3.80E-16	0.0039	0.048
14	ENSG00000264230.7	ANXA8L1	0.36	1.11	1.13	1.14	0.99	0.53	0.018	0.007	0.026	0.023
14	ENSG00000078401.6	EDN1	-0.1	1.23	1.13	1.1	0.79	0.85	1.60E-04	2.20E-04	0.0022	0.016
14	ENSG00000137393.9	RNF144B	-0.02	0.93	1.22	1.09	0.85	0.98	0.037	0.0011	0.019	0.038
14	ENSG00000139269.2	INHBE	0.54	2.28	2.63	1.04	1.15	0.14	8.90E-17	2.90E-23	0.0026	8.60E-05
14	ENSG00000142871.16	CYR61	0.47	0.85	1.18	0.97	1.33	0.0083	4.80E-07	3.60E-14	1.50E-08	1.10E-17
14	ENSG00000269929.1	AL158152.1	0.01	0.63	0.78	0.87	0.87	0.98	0.021	7.80E-04	0.0012	2.00E-04
14	ENSG00000103257.8	SLC7A5	0.49	0.77	0.96	0.86	1.16	0.0012	1.10E-07	2.20E-12	9.00E-09	1.60E-17
14	ENSG00000127528.5	KLF2	-0.05	2	2.01	0.8	0.74	0.82	3.70E-62	4.10E-63	7.30E-09	1.20E-08

14	ENSG00000120738.7	EGR1	0.11	1.76	1.96	0.72	0.78	0.72	1.30E-20	3.80E-26	0.002	9.40E-05
14	ENSG00000135069.13	PSAT1	0.23	0.69	0.81	0.71	1	0.21	9.10E-06	1.30E-08	1.20E-05	2.00E-12
14	ENSG00000096696.13	DSP	0.13	1.67	2.07	0.63	0.82	0.71	1.60E-18	2.60E-30	0.016	8.00E-05
14	ENSG00000144655.14	CSRNP1	0.35	0.96	1.45	0.61	0.75	0.037	1.60E-11	1.20E-27	1.90E-04	1.50E-07

Table S2. Pathway enrichment analysis for transcripts upregulated by LIGHT, IL-13, and IL-17 in human pulmonary fibroblasts, associated with Fig. 1C-G.

Group	Category	Term	Count	%	PValue	Genes	List Total	Pop Hits	Pop Total	Fold Enric	Bonferror	Benjamini	FDR
177	UP_SEQ_F	CARBOHYD:N-linked (GlcNAc...) asparagine	62	36.90476	9.11E-07	KCNK6, O	160	4422	20569	1.802462	0.001004	0.001005	0.001003
177	UP_KW_B	KW-0153~Cholesterol metabolism	8	4.761905	1.87E-06	EBP, HMG	96	71	11450	13.43897	1.34E-04	1.34E-04	1.23E-04
177	UP_KW_B	KW-1207~Sterol metabolism	8	4.761905	4.20E-06	EBP, HMG	96	80	11450	11.92708	3.02E-04	1.51E-04	1.39E-04
177	GOTERM_	GO:0006695~cholesterol biosynthetic process	6	3.571429	1.43E-05	EBP, HMG	155	40	19734	19.09742	0.020331	0.020541	0.020526
177	UP_KW_B	KW-0753~Steroid metabolism	8	4.761905	3.24E-05	EBP, HMG	96	109	11450	8.753823	0.00233	7.49E-04	6.87E-04
177	GOTERM_	GO:0005886~plasma membrane	66	39.28571	3.77E-05	KCNK6, O	157	5592	20887	1.570194	0.009499	0.009544	0.009356
177	UP_KW_B	KW-0152~Cholesterol biosynthesis	5	2.97619	4.16E-05	EBP, HMG	96	24	11450	24.84809	0.002991	7.49E-04	6.87E-04
177	UP_KW_B	KW-0756~Sterol biosynthesis	5	2.97619	1.03E-04	EBP, HMG	96	30	11450	19.87847	0.00741	0.001487	0.001364
177	GOTERM_	GO:0030864~cortical actin cytoskeleton	6	3.571429	1.35E-04	CLDN5, SP	157	66	20887	12.09438	0.03356	0.017067	0.01673
177	GOTERM_	GO:0016324~apical plasma membrane	12	7.142857	2.67E-04	CLDN4, O	157	410	20887	3.893801	0.065212	0.022476	0.022031
177	GOTERM_	GO:0005737~cytoplasm	63	37.5	6.28E-04	EIF4A1, PI	157	5754	20887	1.456623	0.146846	0.039691	0.038907
177	UP_KW_B	KW-0752~Steroid biosynthesis	5	2.97619	6.54E-04	EBP, HMG	96	48	11450	12.42405	0.046037	0.007574	0.006942
177	UP_KW_B	KW-0130~Cell adhesion	13	7.738095	7.36E-04	POSTN, TI	96	492	11450	3.151465	0.051653	0.007574	0.006942
177	GOTERM_	GO:0016323~basolateral plasma membrane	9	5.357143	9.59E-04	CDH2, TRI	157	269	20887	4.451093	0.215591	0.042715	0.041871
177	GOTERM_	GO:0062023~collagen-containing extracellular matrix	11	6.547619	0.001013	FBN2, SFR	157	408	20887	3.586815	0.226183	0.042715	0.041871
177	UP_KW_P	KW-0325~Glycoprotein	65	38.69048	0.001092	KCNK6, O	138	4813	14252	1.394743	0.025878	0.027297	0.027297
177	GOTERM_	GO:0005576~extracellular region	29	17.2619	0.001759	FBN2, TN	157	2101	20887	1.83632	0.359444	0.063575	0.062319
177	UP_KW_C	KW-0272~Extracellular matrix	9	5.357143	0.002053	FBN2, PO	147	279	17942	3.937239	0.067504	0.045374	0.04137
177	UP_KW_C	KW-1003~Cell membrane	49	29.16667	0.002669	OXTR, PTI	147	4049	17942	1.477073	0.086862	0.045374	0.04137
177	UP_KW_D	KW-0677~Repeat	59	35.11905	0.003507	PTPRQ, N	125	5064	14597	1.360542	0.061276	0.063123	0.063123
177	UP_KW_C	KW-0965~Cell junction	10	5.952381	0.006153	CLDN5, CL	147	407	17942	2.99888	0.18928	0.069729	0.063576
177	UP_KW_C	KW-0964~Secreted	29	17.2619	0.009313	FBN2, TN	147	2180	17942	1.62366	0.272486	0.07916	0.072175
26	GOTERM_	GO:0071347~cellular response to interleukin-1	3	25	8.72E-04	EDN1, KLF	12	80	19734	61.66875	0.205649	0.23013	0.23013
26	GOTERM_	GO:0071356~cellular response to tumor necrosis factor	3	25	0.00249	EDN1, KLF	12	136	19734	36.27574	0.482152	0.328627	0.328627
26	GOTERM_	GO:0005125~cytokine activity	3	25	0.005259	EDN1, SPI	12	195	19304	24.74872	0.275026	0.320774	0.320774
26	KEGG_PA	hsa04371:Apelin signaling pathway	3	25	0.00655	EGR1, SPP	9	140	8840	21.04762	0.231162	0.262013	0.262013
26	GOTERM_	GO:0045429~positive regulation of nitric oxide biosynth	2	16.66667	0.024261	EDN1, KLF	12	44	19734	74.75	0.998472	1	1
26	GOTERM_	GO:0045944~positive regulation of transcription by RNA	4	33.33333	0.028863	EGR1, CSR	12	1256	19734	5.237261	0.999561	1	1
26	GOTERM_	GO:0003700~DNA-binding transcription factor activity	3	25	0.035853	EGR1, CSR	12	536	19304	9.003731	0.892173	1	1
26	GOTERM_	GO:0070301~cellular response to hydrogen peroxide	2	16.66667	0.037267	EDN1, KLF	12	68	19734	48.36765	0.999956	1	1
14	GOTERM_	GO:0071347~cellular response to interleukin-1	3	25	8.72E-04	EDN1, KLF	12	80	19734	61.66875	0.205649	0.23013	0.23013
14	GOTERM_	GO:0071356~cellular response to tumor necrosis factor	3	25	0.00249	EDN1, KLF	12	136	19734	36.27574	0.482152	0.328627	0.328627
14	GOTERM_	GO:0005125~cytokine activity	3	25	0.005259	EDN1, SPI	12	195	19304	24.74872	0.275026	0.320774	0.320774
14	KEGG_PA	hsa04371:Apelin signaling pathway	3	25	0.00655	EGR1, SPP	9	140	8840	21.04762	0.231162	0.262013	0.262013
14	GOTERM_	GO:0045429~positive regulation of nitric oxide biosynth	2	16.66667	0.024261	EDN1, KLF	12	44	19734	74.75	0.998472	1	1
14	GOTERM_	GO:0045944~positive regulation of transcription by RNA	4	33.33333	0.028863	EGR1, CSR	12	1256	19734	5.237261	0.999561	1	1
14	GOTERM_	GO:0003700~DNA-binding transcription factor activity	3	25	0.035853	EGR1, CSR	12	536	19304	9.003731	0.892173	1	1
14	GOTERM_	GO:0070301~cellular response to hydrogen peroxide	2	16.66667	0.037267	EDN1, KLF	12	68	19734	48.36765	0.999956	1	1
381	KEGG_PA	hsa04668:TNF signaling pathway	22	6.25	2.73E-14	CSF1, RHB	184	119	8840	8.881988	6.99E-12	7.00E-12	6.26E-12
381	KEGG_PA	hsa05169:Epstein-Barr virus infection	21	5.965909	6.10E-09	HLA-B, TA	184	203	8840	4.970015	1.56E-06	7.81E-07	6.99E-07
381	GOTERM_	GO:0006915~apoptotic process	31	8.806818	6.15E-08	CYFIP2, TI	327	593	19734	3.154818	1.42E-04	1.42E-04	1.40E-04
381	GOTERM_	GO:0048711~positive regulation of astrocyte differentia	6	1.704545	5.08E-07	SHH, BMP	327	11	19734	32.91743	0.001168	5.85E-04	5.78E-04
381	UP_KW_B	KW-0235~DNA replication	12	3.409091	2.00E-06	GINS2, FA	224	94	11450	6.525456	1.84E-04	1.84E-04	1.84E-04
381	GOTERM_	GO:0006954~inflammatory response	23	6.534091	2.30E-06	PTGFR, PT	327	422	19734	3.289143	0.005271	0.001762	0.001742
381	BIOCARTA	h_tnfr2Pathway:TNFR2 Signaling Pathway	8	2.272727	3.67E-06	NFKBIA, T	69	18	1623	10.45411	4.55E-04	4.55E-04	4.40E-04
381	KEGG_PA	hsa05200:Pathways in cancer	29	8.238636	4.30E-06	LAMA1, L	184	533	8840	2.613998	0.0011	3.67E-04	3.28E-04
381	UP_SEQ_F	CARBOHYD:N-linked (GlcNAc...) asparagine	107	30.39773	8.63E-06	CD82, SER	336	4422	20569	1.481286	0.015181	0.015297	0.01528
381	GOTERM_	GO:0031012~extracellular matrix	16	4.545455	8.98E-06	LAMA1, R.	338	240	20887	4.119724	0.003489	0.003217	0.003135
381	GOTERM_	GO:0034097~response to cytokine	8	2.272727	9.69E-06	CD274, BC	327	46	19734	10.49541	0.022053	0.005575	0.005512
381	KEGG_PA	hsa04064:NF-kappa B signaling pathway	12	3.409091	1.02E-05	NFKBIA, P	184	105	8840	5.490683	0.002602	6.51E-04	5.83E-04
381	GOTERM_	GO:0006268~DNA unwinding involved in DNA replicatio	6	1.704545	1.95E-05	GINS2, BL	327	21	19734	17.24246	0.043952	0.008333	0.008239
381	GOTERM_	GO:0071162~CMG complex	5	1.420455	2.01E-05	GINS2, GI	338	11	20887	28.08903	0.007784	0.003217	0.003135
381	KEGG_PA	hsa05222:Small cell lung cancer	11	3.125	2.06E-05	NFKBIA, T	184	93	8840	5.682562	0.00527	0.001057	9.45E-04
381	GOTERM_	GO:0005515~protein binding	257	73.01136	2.16E-05	CYFIP2, SE	336	12754	19304	1.157697	0.012929	0.013013	0.01297
381	GOTERM_	GO:0043065~positive regulation of apoptotic process	18	5.113636	2.82E-05	BARD1, W	327	322	19734	3.373526	0.062821	0.008333	0.008239
381	GOTERM_	GO:0005737~cytoplasm	128	36.36364	2.91E-05	CYFIP2, TI	338	5754	20887	1.374673	0.011259	0.003217	0.003135
381	GOTERM_	GO:0038061~non-canonical NF-kappaB signal transducti	6	1.704545	3.14E-05	NFKBIA, T	327	23	19734	15.74312	0.069781	0.008333	0.008239
381	GOTERM_	GO:0030198~extracellular matrix organization	13	3.693182	3.21E-05	EGFL6, CO	327	173	19734	4.534868	0.071168	0.008333	0.008239
381	GOTERM_	GO:0000727~double-strand break repair via break-induc	5	1.420455	3.26E-05	GINS2, M	327	12	19734	25.14526	0.072258	0.008333	0.008239
381	GOTERM_	GO:0005576~extracellular region	59	16.76136	3.31E-05	ACHE, CPI	338	2101	20887	1.735343	0.012787	0.003217	0.003135
381	KEGG_PA	hsa05417:Lipid and atherosclerosis	16	4.545455	3.96E-05	MMP3, IT	184	216	8840	3.558776	0.010078	0.001688	0.00151
381	GOTERM_	GO:0071260~cellular response to mechanical stimulus	9	2.556818	3.98E-05	IL33, ITGA	327	77	19734	7.053735	0.087497	0.009156	0.009053
381	GOTERM_	GO:0007155~cell adhesion	23	6.534091	5.37E-05	ACHE, CN	327	517	19734	2.684755	0.116223	0.011232	0.011105
381	GOTERM_	GO:0098609~cell-cell adhesion	14	3.977273	6.34E-05	CYFIP2, IT	327	215	19734	3.929678	0.135769	0.012159	0.012022
381	GOTERM_	GO:0006270~DNA replication initiation	6	1.704545	7.14E-05	POLA2, OI	327	27	19734	13.41081	0.151589	0.012645	0.012502
381	GOTERM_	GO:0009887~animal organ morphogenesis	11	3.125	7.88E-05	BMP2, CD	327	134	19734	4.953992	0.16586	0.012953	0.012807
381	KEGG_PA	hsa03030:DNA replication	7	1.988636	8.42E-05	POLA2, RI	184	36	8840	9.341787	0.021336	0.003081	0.002756
381	GOTERM_	GO:0005102~signaling receptor binding	19	5.397727	8.62E-05	TNFSF18,	336	369	19304	2.958253	0.050657	0.025992	0.025905
381	GOTERM_	GO:0071356~cellular response to tumor necrosis factor	11	3.125	8.92E-05	NFKBIA, C	327	136	19734	4.881139	0.185652	0.013691	0.013536
381	KEGG_PA	hsa04210:Apoptosis	12	3.409091	1.14E-04	NFKBIA, B	184	136	8840	4.23913	0.028784	0.003651	0.003266
381	GOTERM_	GO:0062023~collagen-containing extracellular matrix	19	5.397727	1.24E-04	SERPINE2,	338	408	20887	2.877748	0.046954	0.009618	0.009371
381	UP_KW_C	KW-0272~Extracellular matrix	16	4.545455	1.40E-04	WNT10B,	318	279	17942	3.235635	0.005601	0.005616	0.005616
381	KEGG_PA	hsa04060:Cytokine-cytokine receptor interaction	18	5.113636	1.43E-04	TNFSF18,	184	298	8840	2.901955	0.035876	0.00373	0.003337
381	KEGG_PA	hsa04657:IL-17 signaling pathway	10	2.840909	1.46E-04	NFKBIA, F	184	95	8840	5.057208	0.03662	0.00373	0.003337
381	UP_KW_B	KW-0053~Apoptosis	25	7.102273	1.75E-04	CYFIP2, TI	224	549	11450	2.32769	0.015963	0.008045	0.008045
381	GOTERM_	GO:0005615~extracellular space	55	15.625	1.76E-04	MOXD1, A	338	2033	20887	1.671801	0.066071	0.010205	0.009943
381	GOTERM_	GO:0009986~cell surface	25	7.102273	1.84E-04	CPM, ACH	338	657	20887	2.351441	0.06895	0.010205	0.009943
381	GOTERM_	GO:0009897~external side of plasma membrane	19	5.397727	2.30E-04	CD274, CD	338	429	20887	2.736879	0.085664	0.011193	0.010906
381	GOTERM_	GO:0007249~canonical NF-kappaB signal transduction	7	1.988636	2.32E-04	NFKBIA, IF	327	53	19734	7.970573	0.413421	0.033337	0.03296
381	GOTERM_	GO:0002376~immune system process	7	1.988636	2.57E-04	IFIH1, SLFI	327	54	19734	7.82297	0.446724	0.034813	0.03442
381	GOTERM_	GO:0043066~negative regulation of apoptotic process	21	5.965909	3.17E-04	TNFSF18,	327	507	19734	2.499647	0.517388	0.040468	0.040011
381	GOTERM_	GO:0002052~positive regulation of neuroblast proliferat	6	1.704545	3.83E-04	SHH, VEGI	327	38	19734	9.52873	0.585646	0.044178	0.043679
381	GOTERM_	GO:0006508~proteolysis	22	6.25	3.84E-04	USP49, C1	327	554	19734	2.396516	0.586765	0.044178	0.043679
381	UP_KW_N	KW-0645~Protease	24	6.818182	4.17E-04	CPM, USP	230	553	11889	2.243384	0.029189	0.029618	0.0292
381	GOTERM_	GO:0017116~single-stranded DNA helicase activity	5	1.420455	4.22E-04	RFC3, RFC	336	21	19304	13.67914	0.224767	0.084846	0.084564
381	UP_KW_D	KW-0732~Signal	101	28.69318	4.78E-04	CD82, SER	250	4410	14597	1.337231	0.012364	0.012438	0.012438
381	SMART	SM00511:ORANGE	4	1.136364	4.91E-04	BHLHE40,	200	9	10692	23.76	0.08373	0.083529	0.082121
381	GOTERM_	GO:0033209~tumor necrosis factor-mediated signaling p	7	1.988636	5.02E-04	TNFSF18,	327	61	19734	6.925252	0.685078	0.055007	0.054385
381	GOTERM_	GO:0006974~DNA damage response	15	4.261364	6.29E-04	BARD1, BI	327	307	19734	2.94863	0.765053	0.065815	0.065072
381	GOTERM_	GO:0001934~positive regulation of protein phosphoryla	12	3.409091	6.62E-04	CD74, BM	327	206	19734	3.515454	0.782252	0.066257	0.065508
381	GOTERM_	GO:0045121~membrane raft	12	3.409091	6.64E-04	SHH, PDPI	338	211	20887	3.514456	0.227746	0.028706	0.027968
381	BIOCARTA	h_hivnefPathway:HIV-I Nef: negative effector of Fas anc	10	2.840909	7.07E-04	NFKBIA, B	69	61	1623	3.856023	0.083955	0.043829	0.042415
381	UP_KW_D	KW-0923~Fanconi anemia	5	1.420455	7.39E-04	UBE2T, XF	99	21	4817	11.5849	0.038428	0.039172	0.039172
381	GOTERM_	GO:0008284~positive regulation of cell population prolif	20	5.681818	8.16E-04	PTGFR, CS	327	506	19734	2.385321	0.847084	0.078213	0.077329
381	KEGG_PA	hsa03460:Fanconi anemia pathway	7	1.988636	9.06E-04	BLM, EME	184	55	8840	6.114625	0.207004	0.021076	0.018853
381	GOTERM_	GO:0006935~chemotaxis	9	2.556818	9.10E-04	FOSL1, CX	327	121	19734	4.488741	0.876903	0.083753	0.082807
381	SMART	SM01372:E2F_TDP	4	1.136364	9.39E-04	E2F2, E2F	200	11	10692	19.44	0.153916	0.083529	0.082121
381	GOTERM_	GO:0010332~response to gamma radiation	5	1.420455	9.50E-04	CXCL10, X	327	27	19734	11.17567	0.887834	0.084105	0.083155
381	GOTERM_	GO:0030336~negative regulation of cell migration	11	3.125	0.001138	CD74, SHH	327	187	19734	3.549919	0.927135	0.09695	0.095855
381	KEGG_PA	hsa04010:MAPK signaling pathway	16	4.545455	0.001383	CSF1, PLA	184	300	8840	2.562319	0.298241	0.029025	0.025964
381	SMART	SM00005:DEATH	5	1.420455	0.001461	FAS, TNFR	200	27	10692	9.9	0.229098	0.086668	0.085207
381	KEGG_PA	hsa03440:Homologous recombination	6	1.704545	0.001474	BARD1, BI	184	41	8840	7.030753	0.314499	0.029025	0.025964
381	GOTERM_	GO:0005654~nucleoplasm	88	25	0.001895	TNFAIP8,	338	4030	20887	1.349388	0.521907	0.073725	0.07183
381	KEGG_PA	hsa05162:Measles	10	2.840909	0.002335	IFIH1, NFK	184	139	8840	3.456365	0.450405	0.042705	0.038201
381	GOTERM_	GO:0005886~plasma membrane	115	32.67045	0.002344	CD82, CSF	338	5592	20887	1.270838	0.598571	0.082878	0.080747
381	KEGG_PA	hsa04380:Osteoclast differentiation	10	2.840909	0.0027	NFKBIA, F	184	142	8840	3.383344	0.499503	0.046081	0.041221
381	BIOCARTA	h_cd40Pathway:CD40L Signaling Pathway	5	1.420455	0.002714	NFKBIA, T	69	15	1623	7.84058	0.286126	0.096243	0.093139
381	KEGG_PA	hsa05164:Influenza A	11	3.125	0.002995	IFIH1, NFK	184	172	8840	3.072548	0.536025	0.047923	0.042869
381	UP_KW_D	KW-1133~Transmembrane helix	123	34.94318	0.003097	CD82, CSF	250	5882	14597	1.220966	0.077472	0.039809	0.039809
381	BIOCARTA	h_keratinocytePathway:Keratinocyte Differentiation	8	2.272727	0.003105	NFKBIA, B	69	48	1623	3.92029	0.319939	0.096243	0.093139
381	KEGG_PA	hsa05205:Proteoglycans in cancer	12	3.409091	0.003399	WNT10B,	184	204	8840	2.826087	0.581684	0.051179	0.045781

Note for group 14: (NONE OF THESE ARE VALID DUE TO HIGH FDR VALUE.)

381	KEGG_PA1	hsa05171:Coronavirus disease - COVID-19	13	3.693182	0.003836	C1S, C1R,	184	238	8840	2.624224	0.626112	0.054551	0.048797
381	KEGG_PA1	hsa05215:Prostate cancer	8	2.272727	0.004123	NFKBIA, P	184	98	8840	3.921917	0.652693	0.055545	0.049687
381	UP_KW_D	KW-0812~Transmembrane	123	34.94318	0.004593	CD82, CSF	250	5941	14597	1.208841	0.112816	0.039809	0.039809
381	KEGG_PA1	hsa04218:Cellular senescence	10	2.840909	0.005252	RBL1, RAS	184	157	8840	3.060094	0.74027	0.066714	0.059677
381	KEGG_PA1	hsa04110:Cell cycle	10	2.840909	0.005473	ORC6, RBL	184	158	8840	3.040726	0.754593	0.066714	0.059677
381	KEGG_PA1	hsa04621:NOD-like receptor signaling pathway	11	3.125	0.005754	PANX1, NI	184	189	8840	2.796181	0.771754	0.066928	0.059869
381	KEGG_PA1	hsa04625:C-type lectin receptor signaling pathway	8	2.272727	0.006013	NFKBIA, IF	184	105	8840	3.660455	0.786474	0.066928	0.059869
381	KEGG_PA1	hsa05161:Hepatitis B	10	2.840909	0.006682	IFIH1, NFK	184	163	8840	2.947453	0.820282	0.071276	0.063759
381	KEGG_PA1	hsa05167:Kaposi sarcoma-associated herpesvirus infecti	11	3.125	0.007488	NFKBIA, T	184	196	8840	2.696318	0.853981	0.076672	0.068586
381	KEGG_PA1	hsa04151:PI3K-Akt signaling pathway	16	4.545455	0.008062	CSF1, LAM	184	362	8840	2.123469	0.874094	0.07784	0.06963
381	KEGG_PA1	hsa01232:Nucleotide metabolism	7	1.988636	0.00821	UCK2, PNI	184	85	8840	3.956522	0.878805	0.07784	0.06963
381	KEGG_PA1	hsa05165:Human papillomavirus infection	15	4.261364	0.009178	WNT10B,	184	333	8840	2.164121	0.905627	0.083916	0.075066
381	KEGG_PA1	hsa04610:Complement and coagulation cascades	7	1.988636	0.009671	SERPINE2,	184	88	8840	3.82164	0.916903	0.084203	0.075322
381	KEGG_PA1	hsa05203:Viral carcinogenesis	11	3.125	0.010088	NFKBIA, R	184	205	8840	2.577943	0.925404	0.084203	0.075322
381	KEGG_PA1	hsa04512:ECM-receptor interaction	7	1.988636	0.010196	RELN, LAM	184	89	8840	3.778701	0.927465	0.084203	0.075322
94	UP_KW_B	KW-0498~Mitosis	28	30.76923	1.01E-26	CDCA2, CD	65	292	11450	16.89146	4.22E-25	3.97E-25	3.97E-25
94	UP_KW_B	KW-0131~Cell cycle	36	39.56044	1.89E-26	CDCA2, UI	65	687	11450	9.230769	7.93E-25	3.97E-25	3.97E-25
94	UP_KW_B	KW-0132~Cell division	28	30.76923	1.56E-22	CDCA2, CD	65	418	11450	11.79978	6.53E-21	2.18E-21	2.18E-21
94	GOTERM_	GO:0051301~cell division	25	27.47253	2.41E-21	CDCA2, KI	88	385	19734	14.56169	1.44E-18	1.44E-18	1.40E-18
94	UP_KW_C	KW-0137~Centromere	19	20.87912	6.31E-21	SPAG5, HJ	85	148	17942	27.09841	1.51E-19	1.51E-19	1.51E-19
94	GOTERM_	GO:0007059~chromosome segregation	17	18.68132	9.10E-21	CDCA2, SP	88	104	19734	36.65625	5.45E-18	2.73E-18	2.64E-18
94	GOTERM_	GO:0000776~kinetochore	18	19.78022	4.35E-19	PLK4, SPA	90	167	20887	25.01437	7.79E-17	7.79E-17	6.79E-17
94	GOTERM_	GO:0000070~mitotic sister chromatid segregation	12	13.18681	2.41E-18	KIF18A, KI	88	35	19734	76.88571	1.44E-15	4.82E-16	4.66E-16
94	UP_KW_C	KW-0158~Chromosome	27	29.67033	3.90E-18	HJURP, CD	85	627	17942	9.089671	9.35E-17	4.68E-17	4.68E-17
94	UP_KW_C	KW-0995~Kinetochore	15	16.48352	4.98E-17	SPAG5, KN	85	105	17942	30.15462	1.20E-15	3.99E-16	3.99E-16
94	GOTERM_	GO:0008017~microtubule binding	18	19.78022	3.81E-15	SPAG5, KI	86	277	19304	14.58618	7.10E-13	7.16E-13	6.97E-13
94	GOTERM_	GO:0007094~mitotic spindle assembly checkpoint signal	10	10.98901	7.70E-15	CDC20, CE	88	31	19734	72.33871	4.59E-12	1.15E-12	1.12E-12
94	UP_SEQ_F	DOMAIN:Kinesin motor	10	10.98901	2.47E-13	CENPE, KI	89	45	20569	51.3583	1.57E-10	1.57E-10	1.57E-10
94	SMART	SM00129:KISc	10	10.98901	2.63E-13	CENPE, KI	49	45	10692	48.4898	1.45E-11	1.45E-11	1.45E-11
94	INTERPRO	IPR001752:Kinesin_motor_dom	10	10.98901	2.99E-13	CENPE, KI	91	45	20607	50.32234	8.87E-11	8.87E-11	8.81E-11
94	UP_KW_C	KW-0206~Cytoskeleton	30	32.96703	3.65E-12	KIF14, CDC	85	1422	17942	4.453214	8.77E-11	2.19E-11	2.19E-11
94	GOTERM_	GO:0003777~microtubule motor activity	10	10.98901	4.23E-12	CENPE, KI	86	59	19304	38.04493	7.95E-10	3.98E-10	3.87E-10
94	GOTERM_	GO:0005654~nucleoplasm	47	51.64835	7.47E-12	EHF, FEN1	90	4030	20887	2.706614	1.34E-09	6.69E-10	5.83E-10
94	INTERPRO	IPR019821:Kinesin_motor_CS	9	9.89011	8.18E-12	CENPE, KI	91	41	20607	49.70866	2.43E-09	1.21E-09	1.21E-09
94	GOTERM_	GO:0030496~midbody	13	14.28571	3.30E-11	SPAG5, KI	90	192	20887	15.7136	5.90E-09	1.97E-09	1.72E-09
94	GOTERM_	GO:0007018~microtubule-based movement	10	10.98901	7.40E-11	CENPE, KI	88	80	19734	28.03125	4.43E-08	8.86E-09	8.58E-09
94	UP_KW_C	KW-0493~Microtubule	15	16.48352	1.05E-10	SPAG5, KI	85	299	17942	10.58942	2.52E-09	5.03E-10	5.03E-10
94	INTERPRO	IPR036961:Kinesin_motor_dom_sf	10	10.98901	1.08E-10	CENPE, KI	91	84	20607	26.9584	3.21E-08	8.67E-09	8.62E-09
94	INTERPRO	IPR027640:Kinesin-like_fam	8	8.791209	1.17E-10	CENPE, KI	91	34	20607	53.28248	3.47E-08	8.67E-09	8.62E-09
94	UP_KW_D	KW-0175~Coiled coil	35	38.46154	2.12E-10	FEN1, KIF1	63	2734	14597	2.966146	3.40E-09	3.40E-09	3.40E-09
94	UP_KW_C	KW-0539~Nucleus	56	61.53846	6.87E-10	EHF, FEN1	85	5858	17942	2.017858	1.65E-08	2.75E-09	2.75E-09
94	GOTERM_	GO:0072686~mitotic spindle	11	12.08791	7.40E-10	CENPE, ES	90	148	20887	17.24902	1.33E-07	3.31E-08	2.89E-08
94	GOTERM_	GO:0005819~spindle	11	12.08791	1.02E-09	CDC20, CE	90	153	20887	16.68533	1.83E-07	3.67E-08	3.20E-08
94	GOTERM_	GO:0005813~centrosome	18	19.78022	2.97E-09	PLK4, KIF2	90	671	20887	6.225633	5.31E-07	8.86E-08	7.72E-08
94	GOTERM_	GO:0000775~chromosome, centromeric region	8	8.791209	7.26E-09	CENPE, HE	90	61	20887	30.43643	1.30E-06	1.86E-07	1.62E-07
94	UP_KW_M	KW-0505~Motor protein	10	10.98901	1.00E-08	CENPE, KI	55	138	11889	15.66403	4.12E-07	4.12E-07	4.12E-07
94	GOTERM_	GO:0007052~mitotic spindle organization	8	8.791209	1.97E-08	PLK4, CEN	88	68	19734	26.38235	1.18E-05	1.97E-06	1.91E-06
94	GOTERM_	GO:0005524~ATP binding	25	27.47253	2.86E-08	KIF14, TTK	86	1541	19304	3.64155	5.39E-06	1.80E-06	1.75E-06
94	GOTERM_	GO:0005634~nucleus	52	57.14286	4.56E-08	EHF, FEN1	90	6175	20887	1.954339	8.16E-06	1.02E-06	8.89E-07
94	GOTERM_	GO:0005871~kinesin complex	7	7.692308	6.09E-08	KIF18A, KI	90	49	20887	33.15397	1.09E-05	1.21E-06	1.06E-06
94	UP_KW_L	KW-0067~ATP-binding	25	27.47253	6.80E-08	KIF14, TTK	41	1443	6947	2.935534	1.09E-06	1.09E-06	1.09E-06
94	GOTERM_	GO:0000922~spindle pole	9	9.89011	2.06E-07	PLK4, CDC	90	146	20887	14.30616	3.69E-05	3.69E-06	3.21E-06
94	KEGG_PA1	hsa04814:Motor proteins	10	10.98901	2.67E-07	CENPE, KI	43	197	8840	10.4356	2.05E-05	2.05E-05	2.03E-05
94	GOTERM_	GO:0000278~mitotic cell cycle	9	9.89011	2.93E-07	ASPM, CEI	88	148	19734	13.63682	1.76E-04	2.51E-05	2.43E-05
94	KEGG_PA1	hsa04110:Cell cycle	9	9.89011	6.15E-07	CDC20, CC	43	158	8840	11.71033	4.73E-05	2.37E-05	2.34E-05
94	GOTERM_	GO:0000281~mitotic cytokinesis	7	7.692308	7.02E-07	ANLN, ESF	88	71	19734	22.10915	4.20E-04	5.25E-05	5.09E-05
94	GOTERM_	GO:0005874~microtubule	11	12.08791	1.28E-06	CENPE, KI	90	326	20887	7.830845	2.29E-04	2.05E-05	1.79E-05
94	GOTERM_	GO:0008574~plus-end-directed microtubule motor activ	5	5.494505	1.29E-06	KIF18A, KI	86	19	19304	59.06977	2.43E-04	6.06E-05	5.90E-05
94	GOTERM_	GO:0005694~chromosome	10	10.98901	1.38E-06	RECQL4, R	90	254	20887	9.13692	2.46E-04	2.05E-05	1.79E-05
94	GOTERM_	GO:0007080~mitotic metaphase chromosome alignmen	6	6.593407	1.41E-06	CENPE, KI	88	44	19734	30.57955	8.42E-04	9.36E-05	9.06E-05
94	INTERPRO	IPR027417:P-loop_NTPase	17	18.68132	1.96E-06	POLQ, HEI	91	915	20607	4.207278	5.83E-04	1.17E-04	1.16E-04
94	GOTERM_	GO:0090307~mitotic spindle assembly	6	6.593407	2.19E-06	CDC20, UH	88	48	19734	28.03125	0.001309	1.31E-04	1.27E-04
94	UP_KW_P	KW-0597~Phosphoprotein	66	72.52747	3.56E-06	FEN1, CRY	79	8417	14252	1.414605	4.28E-05	4.63E-05	4.63E-05
94	GOTERM_	GO:0005515~protein binding	75	82.41758	9.32E-06	FEN1, HJU	86	12754	19304	1.319969	0.00175	3.50E-04	3.41E-04
94	UP_KW_L	KW-0547~Nucleotide-binding	25	27.47253	1.16E-05	KIF14, TTK	41	1879	6947	2.254378	1.86E-04	9.30E-05	9.30E-05
94	GOTERM_	GO:0000940~outer kinetochore	4	4.395604	1.20E-05	CENPF, CC	90	11	20887	84.39192	0.002153	1.66E-04	1.44E-04
94	GOTERM_	GO:0051983~regulation of chromosome segregation	4	4.395604	1.33E-05	CDCA2, KI	88	11	19734	81.54545	0.007942	7.25E-04	7.02E-04
94	GOTERM_	GO:0005829~cytosol	44	48.35165	1.46E-05	CDCA2, PA	90	5649	20887	1.807651	0.002607	1.86E-04	1.62E-04
94	GOTERM_	GO:0016887~ATP hydrolysis activity	11	12.08791	2.07E-05	RECQL4, P	86	434	19304	5.689208	0.00388	6.48E-04	6.31E-04
94	GOTERM_	GO:0051382~kinetochore assembly	4	4.395604	2.29E-05	CENPE, CE	88	13	19734	69	0.013639	0.001144	0.001108
94	GOTERM_	GO:1990023~mitotic spindle midzone	4	4.395604	2.63E-05	CENPE, KI	90	14	20887	66.30794	0.004699	3.14E-04	2.74E-04
94	UP_KW_D	KW-0905~Primary microcephaly	5	5.494505	3.32E-05	ASPM, CEI	26	37	4817	25.03638	5.65E-04	5.65E-04	5.65E-04
94	GOTERM_	GO:0051315~attachment of mitotic spindle microtubule	4	4.395604	3.62E-05	CENPE, NU	88	15	19734	59.8	0.021476	0.00167	0.001617
94	GOTERM_	GO:0007019~microtubule depolymerization	4	4.395604	4.45E-05	KIF18A, KI	88	16	19734	56.0625	0.026283	0.001902	0.001842
94	GOTERM_	GO:0007088~regulation of mitotic nuclear division	4	4.395604	1.20E-04	CDCA2, NI	88	22	19734	40.77273	0.069338	0.00479	0.004638
94	UP_KW_B	KW-0159~Chromosome partition	5	5.494505	1.32E-04	ESPL1, KIF	65	47	11450	18.73977	0.005538	0.001388	0.001388
94	GOTERM_	GO:0051233~spindle midzone	4	4.395604	2.27E-04	CENPE, KI	90	28	20887	33.15397	0.03979	0.002537	0.002211
94	KEGG_PA1	hsa04114:Oocyte meiosis	6	6.593407	4.77E-04	CDC20, CC	43	139	8840	8.874017	0.03604	0.012232	0.012073
94	GOTERM_	GO:0005876~spindle microtubule	4	4.395604	8.16E-04	KIF4A, CDI	90	43	20887	21.58863	0.135929	0.008591	0.007487
94	GOTERM_	GO:0034080~CENP-A containing chromatin assembly	3	3.296703	8.45E-04	CENPI, HJU	88	10	19734	67.275	0.397309	0.031634	0.03063
94	GOTERM_	GO:0045171~intercellular bridge	5	5.494505	9.18E-04	CENPE, CD	90	101	20887	11.489	0.151564	0.009127	0.007954
94	GOTERM_	GO:0006260~DNA replication	5	5.494505	9.29E-04	RECQL4, F	88	98	19734	11.44133	0.426765	0.032465	0.031435
94	GOTERM_	GO:0051256~mitotic spindle midzone assembly	3	3.296703	0.00103	KIF4A, CDC	88	11	19734	61.15909	0.460524	0.032465	0.031435
94	GOTERM_	GO:0034501~protein localization to kinetochore	3	3.296703	0.00103	CDK1, TTK	88	11	19734	61.15909	0.460524	0.032465	0.031435
94	GOTERM_	GO:0006281~DNA repair	7	7.692308	0.001172	RECQL4, R	88	267	19734	5.879213	0.504692	0.035108	0.033995
94	GOTERM_	GO:0045842~positive regulation of mitotic metaphase/a	3	3.296703	0.001232	CDC20, ES	88	12	19734	56.0625	0.522191	0.035147	0.034032
94	GOTERM_	GO:0005737~cytoplasm	39	42.85714	0.001473	FEN1, KIF1	90	5754	20887	1.572998	0.231893	0.013875	0.012092
94	GOTERM_	GO:0051310~metaphase chromosome alignment	3	3.296703	0.001689	CENPE, CE	88	14	19734	48.05357	0.636765	0.045993	0.044534
94	GOTERM_	GO:0015630~microtubule cytoskeleton	6	6.593407	0.001732	CENPE, KI	90	202	20887	6.893399	0.266735	0.015499	0.013508
94	GOTERM_	GO:0006996~organelle organization	3	3.296703	0.001944	KIF18A, KI	88	15	19734	44.85	0.688175	0.050617	0.049011
94	GOTERM_	GO:0008608~attachment of spindle microtubules to kin	3	3.296703	0.002808	NUF2, KNI	88	18	19734	37.375	0.814414	0.070078	0.067855
94	GOTERM_	GO:0000939~inner kinetochore	3	3.296703	0.002929	CENPI, CE	90	19	20887	36.64386	0.408436	0.024963	0.021755
94	GOTERM_	GO:0007051~spindle organization	3	3.296703	0.003129	ASPM, SPA	88	19	19734	35.40789	0.846998	0.074975	0.072596
94	GOTERM_	GO:0016604~nuclear body	7	7.692308	0.003734	KIF18B, SF	90	348	20887	4.668231	0.488129	0.030383	0.026479
94	UP_KW_P	KW-0832~Ubl conjugation	26	28.57143	0.003907	CDCA2, UI	79	2701	14252	1.736591	0.045894	0.025398	0.025398
94	UP_KW_B	KW-0234~DNA repair	8	8.791209	0.004053	RAD51AP1	65	364	11450	3.871513	0.156822	0.034046	0.034046
94	UP_KW_C	KW-0963~Cytoplasm	40	43.95604	0.004866	KIF14, CDC	85	5805	17942	1.454486	0.110482	0.016684	0.016684
94	GOTERM_	GO:0035371~microtubule plus-end	3	3.296703	0.006315	KIF18B, SF	90	28	20887	24.86548	0.678231	0.049145	0.04283
94	GOTERM_	GO:0180019~Knl1/Spc105 complex	2	2.197802	0.008504	KNL1, ZW	90	2	20887	232.0778	0.783192	0.060889	0.053066
94	GOTERM_	GO:0097125~cyclin B1-CDK1 complex	2	2.197802	0.008504	CCNB1, CD	90	2	20887	232.0778	0.783192	0.060889	0.053066
94	GOTERM_	GO:0090543~Flemming body	3	3.296703	0.009218	KIF14, KIF	90	34	20887	20.47745	0.809424	0.063464	0.055309
94	UP_KW_B	KW-0227~DNA damage	8	8.791209	0.010961	RAD51AP1	65	439	11450	3.210093	0.370536	0.076724	0.076724
94	GOTERM_	GO:0033597~mitotic checkpoint complex	2	2.197802	0.016937	CDC20, M	90	4	20887	116.0389	0.953001	0.105792	0.092198
94	GOTERM_	GO:0031262~Ndc80 complex	2	2.197802	0.016937	NUF2, SPC	90	4	20887	116.0389	0.953001	0.105792	0.092198
94	GOTERM_	GO:0097431~mitotic spindle pole	3	3.296703	0.017139	ASPM, SPA	90	47	20887	14.81348	0.954705	0.105792	0.092198
41	UP_KW_M	KW-0202~Cytokine	8	21.05263	3.41E-07	IL32, CXCL	30	196	11889	16.17551	9.90E-06	9.90E-06	9.90E-06
41	GOTERM_	GO:0006954~inflammatory response	9	23.68421	5.81E-07	PTGER4, C	36	422	19734	11.69076	2.46E-04	2.46E-04	2.44E-04
41	INTERPRO	IPR018048:Chemokine_CXC_CS	4	10.52632	1.76E-06	CXCL6, CX	37	14	20607	159.1274	2.88E-04	1.20E-04	1.19E-04
41	INTERPRO	IPR001089:Chemokine_CXC	4	10.52632	1.76E-06	CXCL6, CX	37	14	20607	159.1274	2.88E-04	1.20E-04	1.19E-04
41	INTERPRO	IPR033899:CXC_Chemokine_domain	4	10.52632	2.20E-06	CXCL6, CX	37	15	20607	148.5189	3.60E-04	1.20E-04	1.19E-04
41	GOTERM_	GO:0006955~immune response	9	23.68421	2.23E-06	IL32, PTGE	36	505	19734	9.769307	9.44E-04	4.72E-04	4.69E-04
41	GOTERM_	GO:0045236~CXCR chemokine receptor binding	4	10.52632	2.45E-06	CXCL6, CX	36	15	19304	142.9926	2.99E-04	2.99E-04	2.99E-04
41	GOTERM_	GO:0005615~extracellular space	14	36.84211	1.82E-05	IL32, CXCL	37	2033	20887	3.887452	0.001914	0.001915	0.001897

41	KEGG_PA	hsa04060:Cytokine-cytokine receptor interaction	8	21.05263	2.97E-05	IL32, CXCL	29	298	8840	8.183291	0.003405	0.003411	0.003233
41	UP_KW_C	KW-0964~Secreted	15	39.47368	4.09E-05	IL32, CXCL	37	2180	17942	3.336598	8.18E-04	8.18E-04	8.18E-04
41	INTERPRO	IPR039809:Chemokine_b/g/d	4	10.52632	5.76E-05	CXCL6, CX	37	43	20607	51.80893	0.009401	0.002058	0.002033
41	SMART	SM00199:SCY	4	10.52632	6.02E-05	CXCL6, CX	20	44	10692	48.6	0.001744	0.001745	0.001745
41	INTERPRO	IPR001811:Chemokine_IL8-like_dom	4	10.52632	7.53E-05	CXCL6, CX	37	47	20607	47.39965	0.012275	0.002058	0.002033
41	INTERPRO	IPR036048:Interleukin_8-like_sf	4	10.52632	7.53E-05	CXCL6, CX	37	47	20607	47.39965	0.012275	0.002058	0.002033
41	GOTERM_	GO:0008009~chemokine activity	4	10.52632	1.07E-04	CXCL6, CX	36	51	19304	42.05664	0.012984	0.006534	0.006534
41	GOTERM_	GO:0005576~extracellular region	13	34.21053	1.30E-04	CXCL6, CX	37	2101	20887	3.492944	0.013534	0.006813	0.006748
41	KEGG_PA	hsa04061:Viral protein interaction with cytokine and cyt	5	13.15789	2.56E-04	CXCL6, CX	29	100	8840	15.24138	0.029041	0.014734	0.013965
41	GOTERM_	GO:0070098~chemokine-mediated signaling pathway	4	10.52632	2.58E-04	CXCL6, CX	36	70	19734	31.32381	0.103589	0.036132	0.035876
41	GOTERM_	GO:0071222~cellular response to lipopolysaccharide	5	13.15789	3.52E-04	CXCL6, CX	36	191	19734	14.34991	0.138696	0.036132	0.035876
41	GOTERM_	GO:0005125~cytokine activity	5	13.15789	4.14E-04	IL32, TNFS	36	195	19304	13.74929	0.049231	0.015342	0.015342
41	GOTERM_	GO:0030593~neutrophil chemotaxis	4	10.52632	4.26E-04	CXCL6, CX	36	83	19734	26.41767	0.165311	0.036132	0.035876
41	KEGG_PA	hsa04668:TNF signaling pathway	5	13.15789	4.98E-04	CXCL6, CX	29	119	8840	12.80788	0.055663	0.019086	0.01809
41	GOTERM_	GO:0005102~signaling receptor binding	6	15.78947	5.03E-04	C3, TNFSF	36	369	19304	8.719061	0.059536	0.015342	0.015342
41	GOTERM_	GO:0061844~antimicrobial humoral immune response n	4	10.52632	9.43E-04	CXCL6, CX	36	109	19734	20.11621	0.329784	0.066661	0.066189
41	GOTERM_	GO:0007165~signal transduction	9	23.68421	0.001217	IL32, C3, C	36	1240	19734	3.978629	0.403193	0.067593	0.067115
41	GOTERM_	GO:0006935~chemotaxis	4	10.52632	0.001275	CXCL6, CX	36	121	19734	18.12121	0.417885	0.067593	0.067115
41	KEGG_PA	hsa05133:Pertussis	4	10.52632	0.001847	C3, CXCL6	29	78	8840	15.63218	0.19153	0.053104	0.050333
41	KEGG_PA	hsa05323:Rheumatoid arthritis	4	10.52632	0.003146	CXCL6, CX	29	94	8840	12.97139	0.303991	0.062139	0.058897
41	KEGG_PA	hsa04657:IL-17 signaling pathway	4	10.52632	0.003242	CXCL6, CX	29	95	8840	12.83485	0.311636	0.062139	0.058897
41	UP_KW_B	KW-0395~Inflammatory response	4	10.52632	0.003855	C3, CXCL8	20	195	11450	11.74359	0.092035	0.096364	0.096364
41	UP_KW_P	KW-1015~Disulfide bond	16	42.10526	0.007007	PTGER4, C	31	3906	14252	1.883223	0.087359	0.098099	0.098099
54	GOTERM_	GO:0051301~cell division	14	26.92308	8.96E-12	ERCC6L, U	51	385	19734	14.07059	4.68E-09	4.68E-09	4.64E-09
54	UP_KW_B	KW-0131~Cell cycle	19	36.53846	1.20E-11	ERCC6L, U	44	687	11450	7.19697	4.45E-10	4.45E-10	4.09E-10
54	UP_KW_B	KW-0132~Cell division	15	28.84615	1.79E-10	ERCC6L, U	44	418	11450	9.338299	6.63E-09	3.32E-09	3.05E-09
54	UP_KW_B	KW-0498~Mitosis	13	25	4.57E-10	ERCC6L, U	44	292	11450	11.58546	1.69E-08	5.64E-09	5.18E-09
54	UP_KW_C	KW-0158~Chromosome	15	28.84615	7.65E-10	CENPU, E	50	627	17942	8.584689	1.22E-08	1.22E-08	9.94E-09
54	GOTERM_	GO:0007059~chromosome segregation	8	15.38462	7.63E-09	TOP2A, S	51	104	19734	29.76471	3.98E-06	1.99E-06	1.98E-06
54	KEGG_PA	hsa04110:Cell cycle	9	17.30769	1.04E-08	SGO1, CD	27	158	8840	18.64979	6.01E-07	6.01E-07	6.01E-07
54	UP_KW_C	KW-0137~Centromere	8	15.38462	1.45E-07	SGO1, CE	50	148	17942	19.39676	2.32E-06	1.16E-06	9.42E-07
54	GOTERM_	GO:0005654~nucleoplasm	27	51.92308	3.78E-07	TOP2A, M	52	4030	20887	2.69111	4.46E-05	4.46E-05	4.01E-05
54	UP_KW_C	KW-0995~Kinetochore	7	13.46154	3.97E-07	SGO1, CE	50	105	17942	23.92267	6.34E-06	2.11E-06	1.72E-06
54	GOTERM_	GO:0005634~nucleus	33	63.46154	9.14E-07	TOP2A, E	52	6175	20887	2.146593	1.08E-04	5.39E-05	4.84E-05
54	GOTERM_	GO:0000278~mitotic cell cycle	6	11.53846	3.58E-05	PBK, MYB	51	148	19734	15.6868	0.018512	0.005597	0.005554
54	UP_KW_C	KW-0539~Nucleus	31	59.61538	3.69E-05	TOP2A, N	50	5858	17942	1.898948	5.90E-04	1.48E-04	1.20E-04
54	GOTERM_	GO:0006270~DNA replication initiation	4	7.692308	4.29E-05	CDC45, C	51	27	19734	57.32462	0.022139	0.005597	0.005554
54	GOTERM_	GO:0000776~kinetochore	6	11.53846	5.37E-05	SGO1, ER	52	167	20887	14.43137	0.006316	0.002112	0.001897
54	GOTERM_	GO:0005829~cytosol	28	53.84615	7.79E-05	ERCC6L, N	52	5649	20887	1.990945	0.009152	0.002298	0.002065
54	GOTERM_	GO:0005813~centrosome	9	17.30769	2.04E-04	SGO1, RA	52	671	20887	5.387567	0.023828	0.004823	0.004332
54	GOTERM_	GO:0000940~outer kinetochore	3	5.769231	3.17E-04	BUB1B, N	52	11	20887	109.5472	0.036721	0.006122	0.0055
54	GOTERM_	GO:0000922~spindle pole	5	9.615385	4.44E-04	SGO1, DIA	52	146	20887	13.75593	0.051006	0.006122	0.0055
54	GOTERM_	GO:0000775~chromosome, centromeric region	4	7.692308	4.47E-04	TOP2A, S	52	61	20887	26.33922	0.051354	0.006122	0.0055
54	GOTERM_	GO:0072686~mitotic spindle	5	9.615385	4.67E-04	CKAP2L, K	52	148	20887	13.57004	0.053623	0.006122	0.0055
54	GOTERM_	GO:0051984~positive regulation of chromosome segreg	3	5.769231	5.61E-04	NCAPG, C	51	14	19734	82.91597	0.254115	0.05862	0.058171
54	UP_KW_P	KW-0832~Ubl conjugation	20	38.46154	7.14E-04	TOP2A, C	49	2701	14252	2.1537	0.007117	0.007854	0.007854
54	GOTERM_	GO:0008017~microtubule binding	6	11.53846	7.33E-04	DIAPH3, K	51	277	19304	8.198768	0.089644	0.049338	0.049338
54	GOTERM_	GO:0003688~DNA replication origin binding	3	5.769231	7.71E-04	CDC45, M	51	16	19304	70.97059	0.093998	0.049338	0.049338
54	GOTERM_	GO:0000779~condensed chromosome, centromeric regi	3	5.769231	7.77E-04	SGO1, NC	52	17	20887	70.88348	0.087591	0.009163	0.008231
54	GOTERM_	GO:0008608~attachment of spindle microtubules to kin	3	5.769231	9.38E-04	SGO1, ND	51	18	19734	64.4902	0.387279	0.081603	0.080977
54	GOTERM_	GO:0045171~intercellular bridge	4	7.692308	0.001931	PRC1, CD	52	101	20887	15.90784	0.203957	0.020716	0.01861
54	GOTERM_	GO:0003682~chromatin binding	7	13.46154	0.002222	TOP2A, R	51	523	19304	5.066097	0.24781	0.094817	0.094817
54	GOTERM_	GO:0000793~condensed chromosome	3	5.769231	0.002275	TOP2A, R	52	29	20887	41.55239	0.235688	0.022373	0.020098
54	UP_KW_L	KW-0067~ATP-binding	11	21.15385	0.003436	TOP2A, E	21	1443	6947	2.521764	0.027162	0.02749	0.02749
54	UP_KW_D	KW-0175~Coiled coil	13	25	0.00396	CENPU, N	30	2734	14597	2.313594	0.050272	0.051477	0.051477
54	GOTERM_	GO:0000794~condensed nuclear chromosome	3	5.769231	0.00451	RAD51, N	52	41	20887	29.39071	0.413394	0.040938	0.036775
54	UP_KW_B	KW-0235~DNA replication	4	7.692308	0.005215	CDC45, M	44	94	11450	11.0735	0.175895	0.048238	0.044327
54	GOTERM_	GO:0005819~spindle	4	7.692308	0.006206	PRC1, BU	52	153	20887	10.50126	0.520295	0.052307	0.046988
54	GOTERM_	GO:0031262~Ndc80 complex	2	3.846154	0.009732	NDC80, S	52	4	20887	200.8365	0.684621	0.076557	0.068772
54	GOTERM_	GO:0015630~microtubule cytoskeleton	4	7.692308	0.013203	CKAP2L, P	52	202	20887	7.953922	0.791617	0.097375	0.087473
54	UP_KW_C	KW-0206~Cytoskeleton	10	19.23077	0.013738	SGO1, RA	50	1422	17942	2.523488	0.198555	0.043963	0.03572
54	UP_KW_L	KW-0547~Nucleotide-binding	11	21.15385	0.023912	TOP2A, E	21	1879	6947	1.936618	0.176027	0.095647	0.095647

Table S3. Gene transcripts upregulated by LIGHT, IL-13, and LIGHT with IL-13, in human pulmonary fibroblasts, associated with Fig. 2A.

Group	gene_name	gene	IL13.Vs.Untreated:log2FoldChangeShrunken	IL13.Vs.Untreated:padj	LIGHT.Vs.Untreated:log2FoldChangeShrunken	LIGHT.Vs.Untreated:padj	IL13_LIGHT.Vs.Untreated:log2FoldChangeShrunken	IL13_LIGHT.Vs.Untreated:padj
1	PARM1	ENSG00000169116.11	0.65	0.05	0.79	0.0086	0.3	0.37
112	CACNA1G	ENSG00000006283.17	0.22	0.56	0.64	0.011	0.23	0.42
112	DNAH5	ENSG00000039139.9	-0.17	0.31	0.81	2.40E-13	0.07	0.69
112	RIPOR3	ENSG00000042062.11	-0.46	3.70E-07	0.68	1.20E-16	0.17	0.075
112	BCL3	ENSG00000069399.14	0.03	0.9	0.92	6.60E-19	0.44	4.70E-05
112	GAL	ENSG00000069482.6	0.08	0.6	0.67	2.80E-13	0.56	6.60E-10
112	PPP1R12B	ENSG00000077157.21	-0.18	0.17	0.68	1.90E-13	0.09	0.47
112	MOXD1	ENSG00000079931.14	-0.51	1.50E-06	0.94	4.60E-24	0.46	2.40E-06
112	COL19A1	ENSG00000082293.12	-0.64	0.11	0.95	0.0012	0.42	0.19
112	KCNK2	ENSG00000082482.13	-0.87	7.60E-10	0.91	2.70E-14	0.02	0.93
112	ACHE	ENSG00000087085.13	-0.4	0.24	0.63	0.01	0	1
112	LAMA1	ENSG00000101680.14	-0.24	0.11	0.62	6.20E-08	-0.05	0.77
112	LACTB	ENSG00000103642.11	-0.07	0.68	0.7	2.40E-16	0.56	4.30E-11
112	WISP1	ENSG00000104415.13	-0.14	0.79	1.34	2.20E-08	0.28	0.35
112	JAK3	ENSG00000105639.18	-0.46	0.024	0.95	5.50E-09	0.08	0.72
112	TFPI2	ENSG00000105825.11	-1.47	3.30E-16	0.7	2.90E-04	-0.72	9.40E-05
112	NAMPT	ENSG00000105835.11	0.02	0.92	0.73	9.50E-23	0.45	2.30E-09
112	RARRES2	ENSG00000106538.9	-0.33	0.04	0.62	2.40E-06	-0.18	0.25
112	TMEM176	ENSG00000106565.17	-0.91	0.0067	0.67	0.026	-0.83	0.0058
112	SLC16A6	ENSG00000108932.11	-0.37	0.12	0.72	6.10E-05	0.32	0.11
112	KLHL5	ENSG00000109790.16	-0.23	0.0011	0.68	6.50E-30	0.24	1.50E-04
112	MGP	ENSG00000111341.9	-1.01	0.0016	0.89	4.90E-04	-0.31	0.35
112	ADTRP	ENSG00000111863.12	0.17	0.79	1.06	0.0044	0.52	0.19
112	SOD2	ENSG00000112096.16	-0.28	0.17	0.9	2.60E-09	0.12	0.57
112	RAB23	ENSG00000112210.11	-0.14	0.15	0.64	4.70E-20	0.17	0.034
112	HES1	ENSG00000114315.3	-0.63	0.25	0.89	0.028	0.51	0.22
112	PTGFR	ENSG00000122420.9	-0.34	0.08	0.97	3.80E-12	0.19	0.27
112	BMP2	ENSG00000125845.6	-0.35	0.062	0.84	9.00E-09	0.03	0.89
112	LIF	ENSG00000128342.4	-0.57	1.20E-11	0.97	8.00E-38	0.25	0.0031
112	KRT17	ENSG00000128422.15	-0.43	0.53	1.29	0.0024	0.75	0.09
112	CHI3L1	ENSG00000133048.12	-0.34	0.67	1.49	0.0031	0.14	0.84
112	IL6ST	ENSG00000134352.19	0.06	0.64	0.62	6.70E-19	0.54	4.10E-15
112	ADAMTS8	ENSG00000134917.9	-0.59	0.052	0.81	0.0017	-0.37	0.2
112	MAP7	ENSG00000135525.18	0.2	0.77	1.01	0.0086	0.68	0.08
112	DYSF	ENSG00000135636.13	0.41	0.6	1.42	0.017	0.41	0.48
112	SERPINE2	ENSG00000135919.12	-0.05	0.74	1.01	8.30E-35	0.29	0.0014
112	ARHGEF4	ENSG00000136002.18	0.09	0.87	0.65	0.02	0.46	0.098
112	DRAM1	ENSG00000136048.13	-0.15	0.055	0.66	6.00E-31	0.29	1.90E-06
112	SLC31A2	ENSG00000136867.10	0.07	0.84	0.68	4.70E-04	0.52	0.0063
112	SLC22A23	ENSG00000137266.14	-0.46	0.5	1.4	0.0012	-0.21	0.75
112	SQOR	ENSG00000137767.13	-0.39	0.028	0.78	2.30E-08	0.21	0.2
112	ITGA11	ENSG00000137809.16	-0.1	0.6	0.68	3.10E-10	-0.04	0.83
112	PAQR5	ENSG00000137819.13	-0.63	2.90E-08	0.84	1.80E-17	-0.4	2.60E-04
112	CLCA2	ENSG00000137975.7	-0.19	0.71	0.75	0.01	-0.11	0.78
112	FGF2	ENSG00000138685.13	0.02	0.91	0.67	1.70E-21	0.58	7.70E-17
112	HAPLN3	ENSG00000140511.11	0.29	0.15	0.7	8.00E-06	0.49	0.0018
112	ABHD2	ENSG00000140526.17	-0.23	3.00E-04	0.74	1.70E-42	0.34	1.40E-09
112	OSMR	ENSG00000145623.12	0.07	0.56	0.86	1.20E-40	0.47	1.20E-12
112	PTGES	ENSG00000148344.10	-0.54	0.0019	0.91	4.10E-10	0.28	0.096
112	HTR7	ENSG00000148680.15	-0.43	0.076	0.71	1.90E-04	0.15	0.55
112	MMP3	ENSG00000149968.11	-0.18	0.82	1.64	0.0095	-0.11	0.93
112	AKAP6	ENSG00000151320.10	-0.14	0.78	0.7	0.013	0.33	0.29
112	GABRA2	ENSG00000151834.15	-0.75	5.60E-26	0.83	5.60E-36	-0.35	9.30E-07
112	GFRA1	ENSG00000151892.14	-0.15	0.71	0.84	3.20E-05	0.54	0.0078

112	ADAMTS3	ENSG00000156140.9	0.21	0.58	0.71	0.0027	0.48	0.045
112	ADAMTSL3	ENSG00000156218.12	-0.82	0.18	1.13	0.017	-0.92	0.067
112	RNF207	ENSG00000158286.12	0.05	0.91	0.69	0.003	0.52	0.027
112	C1R	ENSG00000159403.15	-0.37	0.011	0.59	3.10E-06	-0.3	0.023
112	SQSTM1	ENSG00000161011.19	-0.07	0.31	0.71	1.20E-66	0.54	6.80E-38
112	AK4	ENSG00000162433.14	0.19	0.66	0.6	0.026	0.1	0.78
112	PDPN	ENSG00000162493.16	-0.48	0.44	1.04	0.017	0.2	0.65
112	CXCL1	ENSG00000163739.4	-0.83	0.0026	1.41	2.30E-11	0.28	0.28
112	ITGA2	ENSG00000164171.10	-0.03	0.87	0.7	4.90E-19	0.36	1.10E-05
112	SHH	ENSG00000164690.7	-0.11	0.89	1.06	0.014	-0.05	0.93
112	C10orf10	ENSG00000165507.8	-0.54	0.0085	1.05	5.20E-10	0.22	0.29
112	B4GALNT2	ENSG00000167080.8	0.51	0.25	0.78	0.029	0.56	0.12
112	FILIP1L	ENSG00000168386.18	-0.84	3.10E-20	0.64	1.70E-12	-0.5	4.90E-08
112	CXCL10	ENSG00000169245.5	-0.24	0.68	1.07	0.025	-0.21	0.68
112	CXCL8	ENSG00000169429.10	0.01	0.98	0.79	0.022	-0.29	0.48
112	APLF	ENSG00000169621.9	-0.09	0.8	0.71	2.80E-05	0.45	0.0097
112	ROBO1	ENSG00000169855.19	-0.16	0.011	0.62	2.20E-35	0.32	2.10E-10
112	SLC30A1	ENSG00000170385.9	0.23	0.01	0.64	2.60E-17	0.5	1.70E-11
112	NPAS2	ENSG00000170485.16	0.13	0.21	0.63	3.50E-17	0.47	1.30E-10
112	MTSS1	ENSG00000170873.18	-0.54	3.00E-06	1.41	1.00E-53	0.55	2.70E-08
112	BCL2	ENSG00000171791.12	-0.25	0.57	0.69	0.013	0.32	0.3
112	CYP7B1	ENSG00000172817.3	0.42	0.32	1.31	0.0078	0.36	0.25
112	ODF3B	ENSG00000177989.13	-0.46	0.45	1.13	0.0046	0.36	0.41
112	MSC	ENSG00000178860.8	-0.53	8.40E-04	0.86	4.10E-11	0.26	0.089
112	TMTC2	ENSG00000179104.8	-0.51	0.012	0.73	5.60E-06	0.33	0.065
112	ARID3B	ENSG00000179361.17	0.33	0.084	0.7	4.90E-06	0.5	0.0011
112	TNFSF15	ENSG00000181634.7	-1.48	4.00E-04	2.86	2.10E-25	0.51	0.13
112	C1S	ENSG00000182326.14	-0.22	0.083	0.6	9.60E-10	-0.05	0.75
112	NTM	ENSG00000182667.14	-0.18	0.14	0.61	3.00E-12	0.26	0.0052
112	ALDH1A3	ENSG00000184254.16	-0.45	0.0044	1	3.30E-14	0.11	0.54
112	TACSTD2	ENSG00000184292.6	-0.7	0.029	0.79	0.0029	-0.32	0.31
112	FAM19A3	ENSG00000184599.13	-0.21	0.8	1.03	0.029	0	0.99
112	USP18	ENSG00000184979.9	0.05	0.89	0.68	6.40E-05	0.14	0.53
112	EMID1	ENSG00000186998.15	-1.46	1.50E-06	0.66	0.029	-1.01	3.70E-04
112	TMPPE	ENSG00000188167.8	0.3	0.56	0.98	0.0033	0.54	0.13
112	HES4	ENSG00000188290.10	0.09	0.86	1.02	7.60E-05	0.54	0.046
112	RELN	ENSG00000189056.13	0.31	0.21	0.94	1.00E-07	0.56	0.002
112	MAP3K5	ENSG00000197442.9	-0.11	0.26	0.74	5.20E-32	0.44	5.00E-12
112	ADAMTSL2	ENSG00000197859.9	0.09	0.82	1.49	0.014	0.41	0.28
112	FBXO48	ENSG00000204923.3	0.26	0.56	0.71	0.02	0.51	0.091
112	TMEM170	ENSG00000205269.5	0.11	0.77	0.64	0.0035	0.58	0.0047
112	DIO2	ENSG00000211448.11	-0.3	0.66	1.3	8.80E-04	0.26	0.6
112	ZNF90	ENSG00000213988.10	0.41	0.35	0.69	0.042	0.59	0.07
112	LINC0062	ENSG00000226067.6	-0.13	0.79	0.59	0.03	0.14	0.68
112	CBR3-AS1	ENSG00000236830.6	0.22	0.64	0.61	0.042	0.38	0.22
112	AC003075	ENSG00000237773.5	-0.11	0.89	0.8	0.046	0	1
112	PRR34-AS	ENSG00000241990.5	0.45	0.4	0.84	0.037	0.55	0.18
112	CFB	ENSG00000243649.8	-0.49	0.28	0.76	0.019	0.27	0.46
112	SOCS2-AS	ENSG00000246985.7	0.3	0.41	0.68	0.0082	0.47	0.071
112	AC098679	ENSG00000248429.5	-0.31	0.61	0.91	0.017	0.5	0.22
112	AC107959	ENSG00000253616.5	-0.68	0.28	1.05	0.026	-0.14	0.83
112	AL122035	ENSG00000258824.2	0.76	0.1	0.86	0.04	0.58	0.17
112	AC104072	ENSG00000260878.1	-1.48	9.40E-05	0.8	0.017	-0.55	0.13
112	RASSF5	ENSG00000266094.7	-0.34	0.28	0.77	6.10E-04	0.15	0.62
112	AC004264	ENSG00000268812.3	-0.68	0.2	0.85	0.038	-0.16	0.77
112	AC112220	ENSG00000271020.1	0.31	0.53	0.82	0.016	0.63	0.058
112	AC026356	ENSG00000274964.1	0.47	0.27	0.71	0.041	0.41	0.25
112	AC245100	ENSG00000280649.2	-0.15	0.79	1.01	3.90E-04	0.45	0.15
112	AC241585	ENSG00000281571.2	-0.39	0.5	0.94	0.012	0.6	0.11
310	SLC7A2	ENSG00000003989.17	-0.08	0.46	0.95	8.50E-49	0.73	4.30E-29

310	MCUB	ENSG00000005059.15	0.33	0.062	0.59	4.00E-05	0.85	1.30E-10
310	MAP3K14	ENSG00000006062.14	0.43	0.0026	1.19	7.20E-23	1.53	3.50E-39
310	TMEM132	ENSG00000006118.14	0.11	0.48	1.19	4.50E-40	1.44	1.70E-59
310	CX3CL1	ENSG00000006210.6	-0.67	0.21	2.32	6.40E-12	1.02	0.0043
310	MAP3K9	ENSG00000006432.15	0.45	0.49	1.37	0.003	2.46	3.00E-09
310	E2F2	ENSG00000007968.6	0.68	0.14	1.1	0.0043	2.05	9.50E-10
310	IL32	ENSG00000008517.16	-0.02	0.99	3.36	2.70E-55	4.02	8.20E-80
310	BRCA1	ENSG00000012048.20	0.5	0.021	0.8	2.90E-05	1.47	7.70E-18
310	POLA2	ENSG00000014138.8	0.36	0.034	0.6	3.70E-05	0.96	3.60E-13
310	CCDC88C	ENSG00000015133.18	0.26	0.65	0.97	0.0066	0.79	0.021
310	CD74	ENSG00000019582.14	-0.14	0.64	0.64	4.60E-04	1.12	8.90E-12
310	BIRC3	ENSG00000023445.13	-0.07	0.93	4.34	5.90E-45	4.61	6.90E-51
310	TYMP	ENSG00000025708.13	0.08	0.78	1.64	1.40E-35	1.26	3.60E-21
310	FAS	ENSG00000026103.21	-0.08	0.65	0.64	1.10E-11	0.82	9.90E-20
310	TNFRSF1B	ENSG00000028137.18	-0.01	0.93	0.97	1.10E-52	0.87	3.30E-43
310	POU2F2	ENSG00000028277.21	-0.11	0.71	1.13	6.30E-15	1.22	2.60E-18
310	HDAC9	ENSG00000048052.21	0.42	0.24	1.98	1.70E-17	2.53	7.00E-30
310	TNFRSF9	ENSG00000049249.8	0.04	0.9	1.86	9.60E-04	2.39	2.20E-05
310	NFE2L3	ENSG00000050344.8	0.38	3.20E-07	2.09	1.20E-235	2.33	2.00E-298
310	RELT	ENSG00000054967.12	0.23	0.37	0.63	5.20E-04	0.89	3.40E-08
310	CYFIP2	ENSG00000055163.19	0.02	0.93	0.68	1.80E-10	0.61	5.90E-09
310	TRAF1	ENSG00000056558.10	-0.03	0.91	1.28	2.40E-27	1.98	8.90E-68
310	DCBLD2	ENSG00000057019.15	0.32	3.90E-08	0.93	5.00E-68	1.72	2.50E-239
310	LAMC2	ENSG00000058085.14	-0.52	0.017	1.73	5.60E-26	0.71	4.60E-05
310	NDC1	ENSG00000058804.11	0.45	3.30E-04	0.67	5.90E-09	1.02	8.50E-22
310	DGAT2	ENSG00000062282.14	-0.11	0.75	1.17	3.00E-12	1.25	4.70E-15
310	CHI3L2	ENSG00000064886.13	-0.12	0.89	2	1.00E-07	2.2	9.80E-10
310	SBNO2	ENSG00000064932.15	0.16	0.07	0.62	8.40E-20	0.66	8.20E-23
310	NAV3	ENSG00000067798.14	0.19	0.3	1.33	8.20E-33	1.75	3.70E-59
310	TFRC	ENSG00000072274.12	0.34	0.00093	0.64	9.90E-13	0.66	1.70E-14
310	MCM2	ENSG00000073111.13	0.44	0.0031	0.74	1.70E-08	1.13	5.90E-20
310	WDR62	ENSG00000075702.16	0.52	0.068	1.19	1.50E-07	1.91	4.20E-20
310	NFKB2	ENSG00000077150.18	-0.01	0.96	1.86	1.00E-161	2.04	2.70E-197
310	UBE2T	ENSG00000077152.9	0.29	0.28	0.6	0.0035	1.28	2.00E-13
310	SYNJ2	ENSG00000078269.14	0.16	0.0087	0.66	4.70E-40	0.86	9.00E-71
310	KCNN2	ENSG00000080709.14	0.12	0.84	1.18	2.60E-04	1.03	9.90E-04
310	RBL1	ENSG00000080839.11	0.41	0.0035	0.62	5.90E-07	1.02	9.50E-20
310	CD82	ENSG00000085117.11	-0.83	2.10E-25	1.54	2.20E-100	1	5.80E-42
310	ABCB1	ENSG00000085563.14	-0.05	0.94	1.04	3.60E-04	0.67	0.023
310	RGS1	ENSG00000090104.11	0.76	0.2	1.76	0.0058	2.13	7.60E-04
310	ICAM1	ENSG00000090339.8	-0.52	0.0012	1.68	7.20E-45	0.97	4.50E-15
310	P3H2	ENSG00000090530.9	0.21	0.25	1.16	3.40E-23	1.25	1.70E-28
310	PUS7	ENSG00000091127.13	0.47	3.10E-04	0.75	2.80E-10	1.19	6.30E-28
310	ORC6	ENSG00000091651.8	0.56	0.013	0.81	4.80E-05	1.55	2.20E-18
310	WDR76	ENSG00000092470.11	0.36	0.057	0.68	1.20E-05	0.99	5.80E-12
310	CDC7	ENSG00000097046.12	0.33	0.12	0.6	4.60E-04	0.78	6.00E-07
310	DERL3	ENSG00000099958.14	0.38	0.46	0.9	0.013	1.33	2.60E-05
310	CYTH4	ENSG00000100055.20	0.16	0.8	0.97	0.0044	1.02	0.0013
310	MCM5	ENSG00000100297.15	0.26	0.094	0.68	1.60E-08	1.01	7.40E-19
310	POLE2	ENSG00000100479.12	0.43	0.13	0.94	1.70E-05	1.38	3.30E-12
310	CEP128	ENSG00000100629.16	0.48	0.073	0.6	0.01	1.15	6.10E-09
310	PAPLN	ENSG00000100767.15	-0.13	0.67	1.34	7.30E-15	0.96	2.40E-08
310	NFKBIA	ENSG00000100906.10	-0.37	9.70E-04	2.14	7.30E-130	1.61	5.00E-73
310	PSME2	ENSG00000100911.15	0.23	0.011	0.72	4.00E-22	1.12	3.30E-56
310	MMP9	ENSG00000100985.7	0.22	0.53	2.62	6.90E-06	2.78	1.30E-06
310	E2F1	ENSG00000101412.12	0.55	0.0074	1.14	1.00E-10	1.68	3.60E-24
310	SLC39A14	ENSG00000104635.13	0.36	3.50E-07	0.79	4.10E-36	1.01	3.50E-61
310	TNFRSF10A	ENSG00000104689.9	0.39	0.0068	0.74	1.80E-09	1.1	6.50E-22
310	RELB	ENSG00000104856.13	-0.24	0.035	1.95	3.50E-143	1.98	3.60E-151
310	IL4I1	ENSG00000104951.15	0.27	0.65	3.27	3.10E-21	3.82	3.80E-29
310	IL27RA	ENSG00000104998.3	-0.12	0.69	2.33	2.60E-66	1.94	3.20E-46

310	PLA2G4C	ENSG00000105499.13	-0.74	0.0065	2.81	2.90E-57	2.35	6.20E-40
310	DNAH11	ENSG00000105877.17	0.12	0.84	1.57	6.30E-08	1.91	3.20E-12
310	SERPINE1	ENSG00000106366.8	0.58	2.30E-05	0.61	3.70E-06	1.3	1.00E-26
310	SLC1A1	ENSG00000106688.11	0.47	0.071	1.16	1.00E-08	0.95	1.90E-06
310	MAP3K8	ENSG00000107968.9	-0.24	0.68	1.16	2.20E-04	0.66	0.04
310	SYNGR2	ENSG00000108639.7	0.25	0.018	0.63	3.30E-13	0.82	3.20E-23
310	CCL2	ENSG00000108691.9	-0.37	0.56	1	0.024	1.88	2.70E-06
310	CNTNAP1	ENSG00000108797.11	0.09	0.56	0.78	6.50E-17	0.74	1.30E-15
310	ABCC3	ENSG00000108846.15	0.57	1.30E-04	0.65	4.30E-06	0.73	5.50E-08
310	NFKB1	ENSG00000109320.11	-0.05	0.71	0.71	6.40E-23	0.72	9.70E-25
310	GLRB	ENSG00000109738.10	0.24	0.32	0.77	4.40E-06	0.77	1.60E-06
310	PANX1	ENSG00000110218.8	0.19	4.00E-04	0.65	4.20E-47	0.77	7.80E-69
310	GALNT18	ENSG00000110328.5	0.18	0.7	1.62	9.90E-11	1.43	4.70E-09
310	SLC15A3	ENSG00000110446.10	0.29	0.25	0.64	6.90E-04	0.94	2.60E-08
310	SLC35F2	ENSG00000110660.14	0.29	0.017	0.96	1.50E-22	1.43	1.00E-52
310	OAS3	ENSG00000111331.12	0.23	0.54	0.65	0.012	1.08	1.70E-06
310	MCM3	ENSG00000112118.18	0.27	0.075	0.65	3.70E-08	1	1.80E-19
310	VNN1	ENSG00000112299.7	-0.21	0.76	1.83	0.0037	0.97	0.04
310	HBEGF	ENSG00000113070.7	0.51	0.021	1.58	5.60E-20	2.41	2.60E-49
310	LMNB1	ENSG00000113368.11	0.51	0.023	0.78	8.70E-05	1.46	5.40E-16
310	WWC1	ENSG00000113645.14	0.11	0.74	1.14	5.10E-11	1.92	2.10E-34
310	CDX1	ENSG00000113722.16	0.16	0.8	1.26	5.00E-04	1.68	3.20E-07
310	ECT2	ENSG00000114346.13	0.41	0.011	0.62	1.10E-05	1.1	2.10E-17
310	C3orf52	ENSG00000114529.12	0.33	0.36	0.77	0.0028	1.3	3.20E-09
310	IFIH1	ENSG00000115267.5	0.22	0.57	1.23	5.90E-08	1.32	7.50E-10
310	QPCT	ENSG00000115828.15	0.01	0.99	0.78	1.60E-04	0.88	3.80E-06
310	KYNU	ENSG00000115919.14	-0.38	0.27	1.28	2.90E-09	0.9	3.30E-05
310	PNO1	ENSG00000115946.7	0.42	6.40E-04	0.75	6.40E-12	0.99	1.80E-21
310	PARD3B	ENSG00000116117.17	0.19	0.25	0.83	2.00E-14	0.99	1.50E-21
310	CD58	ENSG00000116815.15	0	1	0.65	2.60E-04	0.92	5.90E-09
310	TNFSF4	ENSG00000117586.10	-0.05	0.78	1.82	6.50E-114	1.86	1.90E-121
310	TNFAIP3	ENSG00000118503.14	-0.55	0.028	1.95	3.20E-25	1.59	2.00E-17
310	TJP2	ENSG00000119139.17	0.24	0.0081	1.21	4.90E-66	1.43	6.90E-94
310	GPR68	ENSG00000119714.10	-0.28	0.54	1.57	4.00E-11	1.32	1.50E-08
310	MOB3B	ENSG00000120162.9	-0.58	0.012	2.23	2.10E-45	1.59	3.10E-23
310	CD274	ENSG00000120217.13	0.55	0.0098	0.81	1.80E-05	1.38	5.70E-17
310	CENPL	ENSG00000120334.15	0.25	0.19	0.59	5.50E-05	0.95	4.90E-13
310	TNFSF18	ENSG00000120337.8	0.03	0.96	1.6	7.10E-08	1.06	3.50E-04
310	MASTL	ENSG00000120539.14	0.5	2.30E-04	0.91	3.20E-14	1.28	4.60E-30
310	SOCS2	ENSG00000120833.13	-0.08	0.63	0.69	1.40E-15	0.72	7.90E-18
310	PLAU	ENSG00000122861.15	0.32	9.40E-04	0.7	5.80E-16	0.89	1.10E-26
310	BHLHE41	ENSG00000123095.5	-0.04	0.94	1.39	2.30E-10	1.16	6.60E-08
310	BTN2A2	ENSG00000124508.16	0.03	0.9	0.6	1.80E-06	0.65	3.30E-08
310	CXCL6	ENSG00000124875.9	-0.07	0.94	2.12	3.10E-09	1.21	6.40E-04
310	IRF1	ENSG00000125347.13	0.51	7.30E-07	1.01	1.20E-27	1.27	6.60E-45
310	TMEM255	ENSG00000125355.15	-0.05	0.96	1.2	9.90E-04	1.12	0.0013
310	C3	ENSG00000125730.16	0.09	0.88	2.46	2.40E-15	2.66	1.70E-18
310	GDF5	ENSG00000125965.8	-0.03	0.96	0.75	0.016	0.68	0.021
310	HIVEP3	ENSG00000127124.14	0.43	0.0068	0.85	7.10E-10	1.73	2.70E-43
310	TRAF2	ENSG00000127191.17	-0.06	0.75	0.92	8.20E-24	1.14	5.00E-39
310	AUNIP	ENSG00000127423.10	0.49	0.43	1.27	0.0058	1.87	6.30E-06
310	F2RL3	ENSG00000127533.3	0.26	0.68	0.86	0.037	0.78	0.044
310	SDF2L1	ENSG00000128228.4	0.09	0.72	0.65	7.20E-07	0.73	4.20E-09
310	POM121L	ENSG00000128262.8	0.28	0.44	1.07	4.10E-06	0.79	7.10E-04
310	APOL3	ENSG00000128284.19	0.37	0.19	1	1.80E-06	1.23	3.00E-10
310	RIBC2	ENSG00000128408.8	0.4	0.6	1.3	0.014	1.28	0.0098
310	STRIP2	ENSG00000128578.9	0.55	0.21	1.31	5.50E-05	1.59	1.60E-07
310	E2F8	ENSG00000129173.12	0.78	0.086	1.13	0.0043	2.14	6.20E-10
310	RHBDF2	ENSG00000129667.12	0.23	0.14	1.53	1.00E-46	1.76	2.70E-64
310	SAMD10	ENSG00000130590.13	0.56	0.0082	0.97	1.40E-07	1.23	4.10E-13
310	GINS2	ENSG00000131153.8	0.46	0.035	0.8	1.20E-05	1.32	2.30E-15

310	TRAF3	ENSG00000131323.14	0.04	0.77	1.42	1.70E-110	1.5	9.60E-127
310	HAUS8	ENSG00000131351.14	0.51	0.043	0.85	6.40E-05	1.22	2.90E-10
310	RFTN1	ENSG00000131378.13	-0.12	0.29	1.28	8.00E-69	1.2	3.40E-61
310	ZSWIM4	ENSG00000132003.9	0.18	0.27	1.18	3.40E-30	1.5	1.30E-50
310	RAMP1	ENSG00000132329.10	0.34	0.009	0.64	9.90E-09	1.22	9.10E-33
310	TRIM47	ENSG00000132481.6	0	1	1.96	8.30E-176	1.88	2.60E-162
310	DCLK1	ENSG00000133083.14	-0.08	0.9	2.34	2.80E-17	2.8	1.40E-25
310	RFC3	ENSG00000133119.12	0.53	0.0031	0.79	8.30E-07	1.35	9.30E-21
310	RARRES3	ENSG00000133321.10	0.11	0.81	0.8	0.0011	0.63	0.0082
310	E2F5	ENSG00000133740.10	0.46	0.036	0.6	0.0021	0.91	1.40E-07
310	IRAK2	ENSG00000134070.4	0.11	0.63	1.21	1.10E-22	1.32	2.10E-28
310	BHLHE40	ENSG00000134107.4	0.48	1.90E-04	0.72	1.20E-09	1.25	2.50E-29
310	RFK	ENSG00000135002.11	0.35	0.007	0.69	3.30E-10	0.79	2.40E-14
310	ADAM19	ENSG00000135074.15	0.19	0.055	0.69	8.40E-19	0.64	6.20E-17
310	STX11	ENSG00000135604.9	0.5	0.35	1.39	5.80E-04	1.68	7.80E-06
310	CPM	ENSG00000135678.11	-0.62	4.70E-05	1.09	3.20E-17	0.62	2.90E-06
310	URB2	ENSG00000135763.9	0.48	7.40E-04	0.7	5.90E-08	1.01	4.50E-17
310	TNS3	ENSG00000136205.16	-0.02	0.86	0.81	3.90E-49	0.67	3.90E-34
310	ZFHX2	ENSG00000136367.13	-0.35	0.54	1.27	1.20E-04	1.21	1.30E-04
310	LIMD2	ENSG00000136490.8	-0.02	0.9	0.6	3.30E-14	0.81	1.30E-26
310	IL33	ENSG00000137033.11	-0.89	0.019	2.59	8.60E-27	2.11	3.10E-18
310	ALDH1B1	ENSG00000137124.7	0.47	2.90E-05	0.59	1.90E-08	0.92	4.00E-21
310	TPMT	ENSG00000137364.4	0.11	0.5	0.72	3.00E-13	1.01	1.70E-27
310	TLR2	ENSG00000137462.6	0.34	0.016	0.72	7.00E-10	0.79	6.00E-13
310	KHK	ENSG00000138030.12	0.52	0.22	0.7	0.048	1.15	1.20E-04
310	CH25H	ENSG00000138135.6	0.36	0.58	2.85	6.90E-08	3.98	5.60E-14
310	BARD1	ENSG00000138376.10	0.55	0.0048	0.6	0.0013	1.15	4.40E-13
310	ITGAV	ENSG00000138448.11	-0.15	0.11	1.37	2.30E-88	1.29	8.90E-79
310	BRCA2	ENSG00000139618.14	0.55	0.0078	0.86	2.30E-06	1.39	2.20E-17
310	FRMD6	ENSG00000139926.15	-0.12	0.3	0.82	9.40E-30	0.94	6.70E-40
310	CDH11	ENSG00000140937.13	0.42	8.40E-14	0.78	6.70E-48	0.94	6.20E-72
310	GATA6	ENSG00000141448.8	0.35	0.026	0.95	1.60E-14	1.2	1.30E-24
310	PMAIP1	ENSG00000141682.11	-0.01	0.95	1.23	1.10E-35	1.11	8.60E-30
310	UCK2	ENSG00000143179.14	0.32	8.50E-04	0.79	2.30E-21	1.11	8.10E-44
310	DUSP10	ENSG00000143507.17	0.18	0.36	0.62	1.50E-06	0.83	4.20E-12
310	CHAC2	ENSG00000143942.4	0.24	0.66	0.73	0.044	1.62	3.00E-08
310	CDCA7	ENSG00000144354.13	0.47	0.01	0.87	2.50E-08	1.44	4.10E-23
310	4-Mar	ENSG00000144583.4	0.54	0.0053	0.99	6.40E-09	1.49	6.30E-21
310	LRIG1	ENSG00000144749.13	-0.08	0.33	0.78	1.30E-49	0.71	2.90E-42
310	NCEH1	ENSG00000144959.9	0.37	0.067	0.94	3.80E-09	1.45	9.10E-23
310	ANK2	ENSG00000145362.17	0.06	0.72	0.73	7.90E-18	1.57	4.80E-86
310	TNFAIP8	ENSG00000145779.7	-0.11	0.46	0.7	8.80E-15	0.65	1.70E-13
310	RNF145	ENSG00000145860.11	0.06	0.47	0.89	6.80E-66	0.71	4.50E-43
310	NFKBIE	ENSG00000146232.15	-0.06	0.79	2.23	1.10E-133	2.44	1.60E-162
310	CDCA5	ENSG00000146670.9	0.54	0.012	0.81	2.50E-05	1.64	2.20E-21
310	ADHFE1	ENSG00000147576.15	0.28	0.28	1.02	5.50E-09	0.97	7.30E-09
310	UGCG	ENSG00000148154.9	0.47	1.20E-05	1.01	2.80E-25	1.33	2.60E-44
310	ST8SIA6	ENSG00000148488.15	0.72	0.17	1.31	0.0019	1.76	4.80E-06
310	INA	ENSG00000148798.10	0.44	0.027	0.59	7.90E-04	1.31	7.70E-18
310	VEGFC	ENSG00000150630.3	0.12	0.2	0.7	1.20E-28	0.74	3.30E-33
310	ITPR1	ENSG00000150995.18	0.27	0.29	0.67	3.30E-04	0.8	2.60E-06
310	FLI1	ENSG00000151702.16	0.14	0.79	0.62	0.044	0.77	0.0045
310	ZNF385D	ENSG00000151789.10	0.37	0.0064	0.74	1.30E-10	1.13	2.80E-26
310	TDO2	ENSG00000151790.8	-0.25	0.76	2.18	1.40E-07	2.01	4.80E-07
310	RASGRP3	ENSG00000152689.17	-0.34	0.58	2.08	3.70E-10	2.06	9.30E-11
310	EME1	ENSG00000154920.14	0.46	0.14	0.6	0.028	1.06	3.50E-06
310	GRAMD2B	ENSG00000155324.9	0.2	0.39	0.69	1.30E-05	0.98	1.10E-11
310	MAP3K7CL	ENSG00000156265.15	0.09	0.77	0.73	6.70E-06	1	1.40E-11
310	ATAD2	ENSG00000156802.12	0.42	0.0025	0.68	3.10E-08	1.24	2.30E-28
310	SASS6	ENSG00000156876.9	0.5	0.1	0.71	0.0064	0.91	9.00E-05
310	IL34	ENSG00000157368.10	-0.31	0.6	4.95	5.60E-60	4.59	1.80E-52

310	MYO1E	ENSG00000157483.8	0.33	1.50E-05	0.61	2.10E-18	0.87	2.00E-39
310	ABHD3	ENSG00000158201.9	0.28	0.3	0.65	9.80E-04	0.63	8.10E-04
310	RHBDL2	ENSG00000158315.10	0.67	0.17	1.47	1.40E-04	2.1	2.70E-09
310	LRRC36	ENSG00000159708.17	0.37	0.5	0.85	0.028	0.74	0.045
310	PTGIR	ENSG00000160013.8	0.07	0.81	0.87	6.40E-09	0.66	9.20E-06
310	CCDC28B	ENSG00000160050.14	-0.01	0.97	0.7	1.40E-09	0.76	6.80E-12
310	CILP2	ENSG00000160161.9	0.11	0.84	1.24	5.90E-06	0.68	0.019
310	WDR4	ENSG00000160193.11	0.15	0.47	0.63	2.00E-06	0.6	2.60E-06
310	SLC2A6	ENSG00000160326.13	0.48	0.0051	0.61	1.10E-04	1.26	1.70E-19
310	TLCD1	ENSG00000160606.10	0.3	0.1	0.68	2.50E-06	0.66	2.20E-06
310	PLXDC1	ENSG00000161381.13	-0.04	0.96	0.76	0.024	0.98	9.40E-04
310	CCNF	ENSG00000162063.12	0.52	0.0069	0.66	1.70E-04	1.31	1.50E-17
310	PAQR4	ENSG00000162073.13	0.29	0.1	0.9	2.10E-11	1.41	1.60E-29
310	NUP35	ENSG00000163002.12	0.17	0.49	0.62	6.60E-05	0.83	4.60E-09
310	CTSS	ENSG00000163131.10	-0.13	0.8	2.87	5.20E-36	2.93	4.50E-38
310	CLDN1	ENSG00000163347.5	-0.09	0.82	2.29	4.30E-57	1.42	1.80E-21
310	NUAK2	ENSG00000163545.8	0.01	0.96	1.63	4.70E-37	1.37	1.70E-26
310	CXCL5	ENSG00000163735.6	-0.6	0.15	1.38	1.80E-06	1.11	8.80E-05
310	CDCP1	ENSG00000163814.7	0.35	2.50E-05	0.6	1.80E-15	0.76	3.50E-25
310	RFC4	ENSG00000163918.10	0.38	0.019	0.65	3.60E-06	1.02	1.10E-15
310	SGMS2	ENSG00000164023.14	0.54	0.0013	0.71	5.00E-06	1.01	1.50E-12
310	CDC25A	ENSG00000164045.11	0.4	0.092	0.77	5.60E-05	1.59	3.50E-21
310	ERAP1	ENSG00000164307.12	0.14	0.057	0.67	3.40E-33	0.68	1.50E-35
310	USP49	ENSG00000164663.14	-0.07	0.76	0.79	1.50E-11	0.7	8.40E-10
310	DDIAS	ENSG00000165490.12	0.6	0.076	1.02	3.00E-04	1.39	3.70E-08
310	EML5	ENSG00000165521.15	0.42	0.48	1.93	1.00E-06	2.29	7.90E-10
310	DDX21	ENSG00000165732.12	0.33	3.50E-06	0.63	5.50E-22	1.01	8.10E-59
310	HSPA12A	ENSG00000165868.13	0.33	0.003	0.77	1.10E-16	1.01	1.00E-30
310	E2F7	ENSG00000165891.15	0.46	0.014	1.03	5.70E-11	1.58	8.20E-27
310	GPR176	ENSG00000166073.10	0.15	0.01	0.83	1.80E-70	0.99	7.80E-104
310	NETO1	ENSG00000166342.18	-0.37	0.62	1.64	4.90E-04	1.5	8.50E-04
310	RRAD	ENSG00000166592.11	0.02	0.94	2.21	8.90E-74	2.33	3.80E-83
310	FAM111A	ENSG00000166801.15	0.18	0.11	0.66	8.90E-15	0.77	2.20E-21
310	TAC3	ENSG00000166863.11	-0.53	0.28	4.54	2.50E-47	4.65	3.90E-50
310	EVA1C	ENSG00000166979.12	0.37	0.27	0.59	0.029	0.73	0.0025
310	NKX3-1	ENSG00000167034.9	-0.21	0.75	0.93	0.0095	1.02	0.0016
310	KRT80	ENSG00000167767.13	0.56	0.002	0.59	7.40E-04	1.43	1.70E-22
310	TAP1	ENSG00000168394.10	0.2	0.19	1.09	1.70E-25	1.13	2.90E-28
310	FAM84B	ENSG00000168672.3	0.28	0.27	0.72	7.60E-05	0.93	2.50E-08
310	IL7R	ENSG00000168685.14	0.1	0.58	1.37	1.70E-41	1.2	2.10E-32
310	C8orf46	ENSG00000169085.11	-0.2	0.79	2.18	2.90E-10	2.06	7.10E-10
310	ATF5	ENSG00000169136.10	-0.05	0.74	1.29	1.60E-65	1.66	6.70E-112
310	COL22A1	ENSG00000169436.16	-0.34	0.28	1.64	4.30E-19	1.38	3.30E-14
310	DTWD2	ENSG00000169570.9	0.23	0.57	0.63	0.018	0.64	0.0089
310	WNT10B	ENSG00000169884.13	0.25	0.43	0.64	0.0036	0.96	7.80E-07
310	REPS2	ENSG00000169891.17	0.49	0.22	1.27	1.30E-05	1.14	5.90E-05
310	CST1	ENSG00000170373.8	-0.81	5.00E-04	2.96	1.00E-53	1.42	7.80E-13
310	KCNS3	ENSG00000170745.11	1.12	0.061	1.21	0.029	1.08	0.036
310	PTGER4	ENSG00000171522.5	0.48	9.40E-05	0.73	9.90E-11	1.38	1.50E-40
310	ENC1	ENSG00000171617.13	-0.03	0.88	1.32	5.80E-38	0.78	1.70E-13
310	CTPS1	ENSG00000171793.13	0.46	6.40E-07	0.73	2.50E-17	1.13	2.90E-43
310	MBOAT1	ENSG00000172197.10	0.03	0.96	0.62	0.012	0.62	0.0074
310	SLFN11	ENSG00000172716.16	0.17	0.038	0.7	2.30E-29	0.78	5.60E-38
310	LVRN	ENSG00000172901.19	0.02	0.97	1.39	4.00E-04	1.07	0.0052
310	NOC3L	ENSG00000173145.11	0.21	0.13	0.72	2.50E-12	0.6	3.90E-09
310	OLR1	ENSG00000173391.8	0.19	0.67	0.62	0.03	0.72	0.0039
310	DAG1	ENSG00000173402.11	0.32	1.50E-05	0.62	3.90E-20	0.81	6.40E-36
310	SCAI	ENSG00000173611.17	0.16	0.39	0.59	2.00E-06	0.68	6.90E-09
310	FOSL1	ENSG00000175592.8	0.58	1.80E-06	1.1	1.70E-23	1.79	2.90E-63
310	TYMS	ENSG00000176890.15	0.2	0.26	0.68	8.80E-08	1.01	1.80E-17
310	KCNA3	ENSG00000177272.8	0.53	0.038	1.72	3.40E-19	1.54	4.90E-16

310	PRSS36	ENSG00000178226.10	-0.46	0.45	1.09	0.0062	1.35	2.00E-04
310	FBXO34	ENSG00000178974.9	0.02	0.9	0.72	1.80E-32	0.83	7.80E-45
310	RRS1	ENSG00000179041.3	0.46	0.0017	0.91	1.20E-12	1.59	7.70E-40
310	FJX1	ENSG00000179431.6	0.38	0.0027	1.01	5.40E-21	1.22	1.50E-31
310	CITED4	ENSG00000179862.6	0.09	0.75	1.18	1.60E-15	1.37	2.50E-22
310	FGD6	ENSG00000180263.13	-0.18	0.37	0.68	4.60E-07	0.68	1.70E-07
310	PHLDA2	ENSG00000181649.5	0.51	5.50E-07	0.61	3.90E-10	1.04	5.00E-30
310	GINS3	ENSG00000181938.13	0.53	0.083	0.98	7.50E-05	1.63	1.60E-13
310	GJC1	ENSG00000182963.9	0.2	0.023	0.86	1.20E-35	1.07	1.70E-56
310	CADM1	ENSG00000182985.17	0.1	0.57	0.62	1.30E-08	0.74	3.70E-13
310	CAMK1D	ENSG00000183049.12	0.08	0.83	1.1	3.80E-09	1.22	4.70E-12
310	KCTD8	ENSG00000183783.6	0.08	0.83	0.76	7.00E-05	0.62	9.20E-04
310	GPR39	ENSG00000183840.6	0.45	0.0082	2.06	2.90E-57	2.39	2.40E-79
310	NR2C2AP	ENSG00000184162.14	0.17	0.6	0.64	0.002	0.88	2.00E-06
310	CSF1	ENSG00000184371.13	-0.1	0.45	0.92	1.70E-29	0.9	4.70E-29
310	KNTC1	ENSG00000184445.11	0.43	0.0027	0.59	5.70E-06	1.03	2.90E-18
310	DUSP8	ENSG00000184545.10	0.4	0.11	0.71	5.90E-04	1.21	2.40E-11
310	FMNL1	ENSG00000184922.13	0.01	0.98	0.68	5.50E-07	0.65	5.30E-07
310	BRI3BP	ENSG00000184992.10	0.21	0.52	0.64	0.0035	1.09	8.30E-09
310	ANO9	ENSG00000185101.12	-0.12	0.78	1.47	0.0047	1.3	0.0059
310	C14orf80	ENSG00000185347.17	0.5	0.052	0.95	8.60E-06	1.35	3.10E-12
310	ZNF267	ENSG00000185947.14	-0.17	0.31	1.22	1.30E-34	0.96	2.90E-22
310	FANCA	ENSG00000187741.14	0.58	0.0027	0.88	2.80E-07	1.77	6.90E-30
310	XRCC2	ENSG00000196584.2	0.28	0.45	0.81	0.001	1.61	2.80E-14
310	BLM	ENSG00000197299.10	0.35	0.32	0.84	9.80E-04	1.5	1.70E-11
310	SLC28A3	ENSG00000197506.7	-0.07	0.96	2.25	2.40E-06	3.17	1.00E-11
310	SULT1C4	ENSG00000198075.9	-0.63	3.80E-04	1.15	7.70E-16	1.05	4.30E-14
310	PIM3	ENSG00000198355.4	0.21	0.0085	0.77	1.50E-33	0.86	1.30E-43
310	WDHD1	ENSG00000198554.11	0.37	0.02	0.7	2.40E-07	1.13	9.60E-20
310	EGFL6	ENSG00000198759.11	0.56	0.19	1.38	0.0056	1.29	0.0045
310	ALPK2	ENSG00000198796.6	0.18	0.21	1.12	2.40E-29	1.11	1.00E-29
310	PNP	ENSG00000198805.11	0.51	0.013	0.85	1.20E-06	1.51	6.20E-21
310	FICD	ENSG00000198855.6	0.24	0.19	0.59	1.10E-05	0.62	9.00E-07
310	L1CAM	ENSG00000198910.12	-0.04	0.91	1.22	1.70E-15	0.75	1.60E-06
310	SLC44A4	ENSG00000204385.10	-0.12	0.9	1.25	0.017	1.62	6.30E-04
310	ATP6V0E2	ENSG00000204934.10	-0.05	0.94	0.95	0.0027	0.78	0.011
310	PSMB10	ENSG00000205220.11	0.45	2.90E-04	0.71	3.90E-10	0.96	1.10E-19
310	HLA-H	ENSG00000206341.7	-0.19	0.35	0.87	9.10E-12	0.69	4.20E-08
310	RNU6-26P	ENSG00000206712.1	0.4	0.47	1.01	0.0085	1.35	9.30E-05
310	IFRD2	ENSG00000214706.10	0.31	0.0026	0.64	3.00E-13	1	3.00E-34
310	AL390719	ENSG00000217801.9	0.28	0.047	0.69	3.90E-10	0.82	6.90E-15
310	APOL6	ENSG00000221963.5	-0.04	0.82	0.82	1.60E-22	0.68	3.40E-16
310	PSME2P2	ENSG00000225131.2	0.17	0.81	0.91	0.037	1.43	1.50E-04
310	LINC0051	ENSG00000227036.6	-0.56	0.022	0.9	2.90E-06	0.8	1.90E-05
310	HLA-B	ENSG00000234745.10	-0.13	0.23	0.8	1.10E-29	0.68	5.30E-22
310	AL357060	ENSG00000237499.6	0	0.99	1.45	0.0043	1.36	0.0045
310	NME1	ENSG00000239672.7	0.45	0.0026	0.66	9.50E-07	1.06	7.60E-18
310	PSMB9	ENSG00000240065.7	0.35	0.32	0.84	0.0011	1.35	4.40E-09
310	LINC0097	ENSG00000240476.1	0.06	0.93	1.37	6.40E-06	1.82	1.10E-10
310	RPSAP52	ENSG00000241749.4	0.43	0.24	0.63	0.038	1.2	1.40E-06
310	FMN1	ENSG00000248905.8	0.49	1.20E-05	0.59	4.20E-08	1.22	6.60E-35
310	AC097451	ENSG00000250657.1	-0.58	1.50E-04	1.34	2.50E-27	0.7	6.30E-08
310	AC125807	ENSG00000250899.3	0.42	0.1	0.85	3.60E-05	0.85	1.10E-05
310	LINC0240	ENSG00000257219.5	0.3	0.54	0.96	0.0031	1.44	5.60E-07
310	C17orf49	ENSG00000258315.5	0.26	0.63	0.89	0.0072	0.67	0.041
310	AL365361	ENSG00000259834.1	-0.31	0.5	0.94	0.00093	0.62	0.031
310	AC004656	ENSG00000260822.1	0.69	0.21	0.92	0.048	1.16	0.0048
310	AC092718	ENSG00000261061.1	0.33	0.34	0.79	0.0014	0.98	1.20E-05
310	GATA6-AS	ENSG00000266010.1	0.78	0.079	1.23	0.0011	1.26	3.20E-04
310	AC009549	ENSG00000270607.1	-0.89	0.0059	1.24	3.50E-07	0.97	5.10E-05

310	AL096865	ENSG00000271857.1	0.72	0.12	0.96	0.017	0.91	0.018
310	CU639417	ENSG00000275993.2	0.41	0.48	1.55	1.70E-04	1.07	0.0075
310	PIGW	ENSG00000277161.1	0.37	0.015	0.74	4.70E-09	1.1	6.30E-21
310	AC093535	ENSG00000279118.1	0.9	0.1	1.49	0.0033	1.21	0.0099
310	AC016397	ENSG00000279822.1	0.45	0.33	0.76	0.032	0.95	0.0028
43	ACPP	ENSG00000014257.15	0.64	0.0019	0.19	0.47	-0.15	0.56
43	TBXAS1	ENSG00000059377.16	0.61	4.50E-13	0.11	0.33	0.33	1.40E-04
43	SCARF1	ENSG00000074660.15	0.87	0.029	0.27	0.58	0.68	0.064
43	PDGFB	ENSG00000100311.16	0.63	0.029	0.21	0.56	0.56	0.032
43	TBC1D19	ENSG00000109680.10	0.66	3.20E-13	-0.06	0.68	0.4	1.20E-05
43	HMGCR	ENSG00000113161.15	0.62	2.00E-14	0.03	0.83	0.57	7.90E-13
43	PFKFB4	ENSG00000114268.11	0.77	4.80E-11	-0.07	0.72	0.52	7.50E-06
43	LOXL3	ENSG00000115318.11	0.85	2.50E-14	-0.24	0.084	0.24	0.061
43	DOK1	ENSG00000115325.13	0.62	2.90E-13	-0.13	0.24	0.27	0.0031
43	NCF2	ENSG00000116701.14	1.07	0.036	0.44	0.46	0.54	0.28
43	SLC2A1	ENSG00000117394.20	0.6	9.90E-16	-0.03	0.83	0.2	0.016
43	SERPINB6	ENSG00000124570.17	0.6	8.20E-47	-0.24	2.70E-07	0.36	1.00E-17
43	ZFP36	ENSG00000128016.5	0.98	6.80E-11	-0.16	0.46	0.39	0.017
43	KDR	ENSG00000128052.8	0.93	2.30E-06	0.05	0.88	0.05	0.87
43	ASPHD2	ENSG00000128203.6	0.72	0.015	0.55	0.069	0.56	0.04
43	FST	ENSG00000134363.11	0.61	6.30E-10	-0.23	0.042	0.11	0.37
43	SPTBN5	ENSG00000137877.9	0.78	0.025	0.04	0.94	0.55	0.095
43	ADCY3	ENSG00000138031.14	0.64	4.20E-17	-0.38	2.40E-06	-0.02	0.85
43	ADAMTS14	ENSG00000138316.10	0.69	3.50E-04	-0.22	0.38	0.31	0.12
43	FBN2	ENSG00000138829.11	0.6	5.80E-09	0	1	0.37	3.40E-04
43	PTPRQ	ENSG00000139304.12	1.04	0.02	0.2	0.74	0.68	0.11
43	GNAL	ENSG00000141404.15	1.06	2.60E-04	0.24	0.55	0.27	0.44
43	WNT9A	ENSG00000143816.7	0.6	0.0013	0.17	0.48	0.56	0.0011
43	AGTR1	ENSG00000144891.17	0.68	0.0048	-0.89	5.10E-04	0.14	0.64
43	SLC7A11	ENSG00000151012.13	0.63	1.10E-13	0.06	0.62	-0.08	0.46
43	ABTB2	ENSG00000166016.5	0.77	4.20E-04	-0.21	0.48	0.3	0.21
43	SCN11A	ENSG00000168356.11	1.1	2.20E-05	-0.46	0.16	0.49	0.079
43	TNXB	ENSG00000168477.17	0.68	2.00E-04	0.23	0.3	0.16	0.46
43	NR0B1	ENSG00000169297.7	1.13	0.037	0	1	0.36	0.54
43	C5orf30	ENSG00000181751.9	0.62	1.10E-04	0.17	0.43	0.49	0.0013
43	MEX3B	ENSG00000183496.5	0.6	2.00E-14	0.07	0.58	0.51	5.00E-11
43	OPCML	ENSG00000183715.13	0.85	0.0087	0.31	0.44	0.58	0.063
43	SOCS1	ENSG00000185338.4	0.6	1.70E-16	0.07	0.52	0.49	8.90E-12
43	CARD9	ENSG00000187796.14	0.59	0.0096	0.16	0.58	0.14	0.6
43	ARL4C	ENSG00000188042.7	0.64	1.00E-13	-0.14	0.2	0.58	3.00E-12
43	C6orf141	ENSG00000197261.11	0.71	0.0033	-0.53	0.043	0.45	0.059
43	SLC26A6	ENSG00000225697.12	1.15	8.90E-13	-0.38	0.046	0.31	0.095
43	LINC0048	ENSG00000229694.6	0.86	0.033	-0.34	0.49	0.41	0.33
43	LINC0048	ENSG00000235641.4	1.12	2	0.22	2	1.15	0.072
43	SRD5A3-A	ENSG00000249700.8	1.09	0.028	0.71	0.17	0.55	0.27
43	AC010735	ENSG00000261379.1	0.61	0.049	0.06	0.9	0.18	0.59
43	MIR193BI	ENSG00000262454.3	0.59	0.015	-0.23	0.45	-0.19	0.49
43	AC127502	ENSG00000270055.1	0.61	0.0022	0.19	0.46	0.33	0.1
43	AC010735	ENSG00000272622.1	0.6	0.0063	0.08	0.8	0.01	0.98
633	ARL14	ENSG00000179674.3	0.17	2	0.39	2	2.67	1.40E-04
633	AC004917	ENSG00000243797.6	2.35	2	1.09	2	2.5	3.10E-05
633	COLCA1	ENSG00000196167.9	0.13	2	2.93	2	2.47	0.0012
633	LINP1	ENSG00000223784.1	1.43	2	-0.14	2	2.44	0.0024
633	KCNQ5	ENSG00000185760.15	0.7	0.16	-0.25	0.66	2.37	2.90E-11
633	PTGDR	ENSG00000168229.3	0.9	2	1.42	2	2.32	1.50E-04
633	CLCA1	ENSG00000016490.15	2.91	2	-0.67	2	2.29	0.0014
633	SMCO2	ENSG00000165935.9	0.44	2	1.09	2	2.17	0.0013
633	COL17A1	ENSG00000065618.18	1.43	2	-0.17	2	2.09	0.0089
633	GADL1	ENSG00000144644.14	1.81	2	0.63	2	2.02	0.0024
633	HS6ST3	ENSG00000185352.8	1.39	2	0.13	2	2.02	0.0063

633	ST14	ENSG00000149418.10	1.67	2	0.7	2	2.01	0.0023
633	SYT9	ENSG00000170743.16	-0.18	2	1.31	2	1.97	0.0032
633	DPY19L2F	ENSG00000189212.12	0.45	2	0.36	2	1.97	7.70E-04
633	NIPAL4	ENSG00000172548.14	0.86	0.14	0.95	0.073	1.87	1.10E-04
633	AC107308	ENSG00000277945.1	1.48	2	0.81	2	1.86	1.60E-04
633	AURKB	ENSG00000178999.12	0.56	0.014	0.53	0.016	1.82	6.30E-25
633	LGI2	ENSG00000153012.11	0	2	1.23	2	1.78	8.40E-04
633	EGR2	ENSG00000122877.15	0.31	0.45	0.47	0.14	1.77	6.60E-13
633	AC023906	ENSG00000259712.1	-0.36	2	2.03	2	1.76	0.0023
633	AP000777	ENSG00000255959.1	2.17	2	1.14	2	1.74	0.013
633	CIITA	ENSG00000179583.18	0.81	2	0.74	2	1.72	0.0017
633	AC005050	ENSG00000267052.1	0.15	2	0.51	2	1.72	0.0039
633	AC078850	ENSG00000248187.1	1.51	2	0.77	2	1.71	0.0023
633	UOX	ENSG00000240520.6	1.49	2	0.48	2	1.7	0.0017
633	AL157702	ENSG00000227482.1	0.53	2	0.85	2	1.69	0.016
633	CCL7	ENSG00000108688.11	0.62	2	0.89	2	1.67	0.0017
633	PRSS22	ENSG00000005001.9	0.62	2	0.51	2	1.66	0.0011
633	LINC0205	ENSG00000226859.1	-0.66	2	1.58	2	1.65	0.0056
633	DEPDC1B	ENSG00000035499.12	0.57	0.05	0.44	0.13	1.64	1.30E-14
633	COLCA2	ENSG00000214290.8	-0.74	2	2.45	2	1.63	0.02
633	ETV7	ENSG00000010030.13	0.25	0.73	1.02	0.057	1.58	0.0017
633	VENTX	ENSG00000151650.7	1.52	2	-0.19	2	1.57	0.027
633	AC099568	ENSG00000272931.1	1.28	2	-0.72	2	1.57	0.033
633	KY	ENSG00000174611.11	-0.06	2	0.67	2	1.54	0.0061
633	DEPDC1-A	ENSG00000234264.1	0.15	2	0.51	2	1.54	0.006
633	ZNF114	ENSG00000178150.9	0.86	0.14	0.71	0.19	1.52	6.10E-04
633	AC116345	ENSG00000249743.5	0.51	2	0.45	2	1.51	0.0051
633	AC024933	ENSG00000272656.1	0.09	2	0.01	2	1.51	0.05
633	STAC	ENSG00000144681.10	1.04	2	0.95	2	1.49	0.018
633	CST2	ENSG00000170369.3	0.25	2	1.48	2	1.49	0.02
633	OVCH1	ENSG00000187950.8	1	0.074	0.94	0.072	1.47	0.0012
633	IL6	ENSG00000136244.11	0.7	0.16	0.66	0.16	1.46	2.00E-04
633	IRX3	ENSG00000177508.11	1.76	2	-0.04	2	1.46	0.0099
633	CDCA3	ENSG00000111665.11	0.48	0.085	0.54	0.033	1.45	2.00E-12
633	AC007611	ENSG00000260086.2	0.87	2	1.3	2	1.45	0.006
633	AL355607	ENSG00000260454.1	0	2	1.01	2	1.45	0.0031
633	KCNAB1	ENSG00000169282.17	1.41	2	1.1	2	1.44	0.0088
633	MMP13	ENSG00000137745.11	0	1	0.35	0.42	1.43	0.0022
633	AC073130	ENSG00000237870.6	0.47	2	0.74	2	1.43	0.0056
633	SLC22A16	ENSG00000004809.13	0.98	2	-0.04	2	1.41	0.0068
633	CST4	ENSG00000101441.4	0.71	2	1.61	2	1.41	0.0061
633	AC069499	ENSG00000241634.1	0.84	0.14	0.95	0.066	1.41	0.0017
633	AC090197	ENSG00000253837.1	0.71	0.24	0.92	0.075	1.41	0.0016
633	AC016205	ENSG00000267374.1	0.4	0.47	0.76	0.071	1.41	4.40E-05
633	SUSD4	ENSG00000143502.14	0.09	2	0.17	2	1.4	0.031
633	FBXO43	ENSG00000156509.13	0.69	0.31	0.77	0.2	1.4	0.0065
633	EVI2B	ENSG00000185862.6	0.41	2	0.34	2	1.4	0.04
633	DRD1	ENSG00000184845.3	0.18	2	1.23	2	1.39	0.021
633	ZNF724	ENSG00000196081.9	-0.11	0.91	0.65	0.23	1.39	0.0014
633	AC003092	ENSG00000236453.5	0.37	0.64	1.02	0.08	1.39	0.0076
633	AMZ1	ENSG00000174945.13	0.19	0.79	0.87	0.066	1.38	7.80E-04
633	PECAM1	ENSG00000261371.5	0.65	2	1.19	2	1.38	0.015
633	ASB2	ENSG00000100628.11	0.45	2	0.99	2	1.37	0.025
633	EVI2A	ENSG00000126860.11	0.63	2	0.28	2	1.37	0.05
633	FAM201B	ENSG00000230992.3	-0.06	2	0.94	2	1.37	0.037
633	AURKA	ENSG00000087586.17	0.58	2.70E-04	0.5	0.0015	1.36	5.10E-24
633	MIR573	ENSG00000207697.1	0.07	2	0.84	2	1.36	0.04
633	AL356414	ENSG00000205300.3	0.84	0.071	0	1	1.35	0.004
633	PRKCQ-AS	ENSG00000237943.6	0.49	0.42	0.27	0.66	1.33	8.80E-04
633	PTHLH	ENSG00000087494.15	0.26	0.65	-0.15	0.79	1.32	3.40E-05

633	C1QTNF1-	ENSG00000265096.1	0.4	2	0.11	2	1.3	0.05
633	RNF19B	ENSG00000116514.16	0.46	0.0024	0.45	0.0023	1.29	3.00E-25
633	STIL	ENSG00000123473.15	0.53	0.0044	0.57	0.0012	1.29	9.10E-18
633	SERPINB2	ENSG00000197632.8	0.39	0.0079	0.41	0.0029	1.29	1.30E-28
633	KIF11	ENSG00000138160.5	0.51	0.0064	0.45	0.013	1.28	4.60E-17
633	MTFR2	ENSG00000146410.11	0.54	0.23	0.63	0.11	1.28	5.90E-05
633	ACTC1	ENSG00000159251.6	-0.07	0.95	-0.11	0.84	1.28	0.0017
633	MICALCL	ENSG00000133808.4	0.62	0.18	0.56	0.21	1.27	2.20E-04
633	LINC0225	ENSG00000238042.5	0.7	2	0.76	2	1.27	0.026
633	SNTG1	ENSG00000147481.14	0.67	2	0.7	2	1.26	0.028
633	CCNB2	ENSG00000157456.7	0.53	0.014	0.36	0.11	1.26	9.00E-13
633	LINC0177	ENSG00000226053.1	-0.24	2	0.43	2	1.26	0.042
633	GAS2L3	ENSG00000139354.10	0.56	8.30E-04	0.38	0.034	1.23	2.90E-18
633	EPHB1	ENSG00000154928.16	0.38	0.21	0.26	0.39	1.23	2.90E-09
633	PPM1L	ENSG00000163590.13	0.14	0.82	0.32	0.47	1.22	6.20E-05
633	NCALD	ENSG00000104490.17	-0.49	0.51	1.11	0.054	1.21	0.022
633	GCNT4	ENSG00000176928.5	0.92	0.085	0.32	0.61	1.21	0.0071
633	PSG1	ENSG00000231924.9	0.57	0.37	0.62	0.25	1.21	0.0061
633	ST3GAL6-	ENSG00000239445.5	0.77	2	0.71	2	1.21	0.046
633	RASGRF1	ENSG00000058335.15	0.83	2	0.82	2	1.2	0.048
633	SEMA7A	ENSG00000138623.9	0.55	1.30E-11	0.55	1.70E-11	1.2	1.00E-57
633	FGF5	ENSG00000138675.16	0.51	7.50E-05	0.43	9.00E-04	1.19	6.90E-26
633	ABCA3	ENSG00000167972.13	0.96	0.089	0.75	0.17	1.19	0.011
633	SNORD46	ENSG00000200913.1	1.33	2	1.21	2	1.19	0.042
633	KRT81	ENSG00000205426.10	0.76	0.25	-0.09	0.91	1.19	0.019
633	CCNA2	ENSG00000145386.9	0.51	0.011	0.53	0.0053	1.18	5.60E-13
633	ANKRD61	ENSG00000157999.5	0.73	2	0.86	2	1.18	0.05
633	TNFRSF12	ENSG00000006327.13	0.53	5.00E-06	0.54	2.20E-06	1.17	8.30E-29
633	DBF4	ENSG00000006634.7	0.48	0.0094	0.47	0.0073	1.17	2.60E-15
633	NR1I2	ENSG00000144852.17	1.47	2	1.19	2	1.17	0.021
633	C4orf32	ENSG00000174749.5	0.25	0.49	0.35	0.23	1.17	2.80E-08
633	C1QTNF1	ENSG00000173918.14	0.4	5.90E-04	0.21	0.095	1.16	3.10E-32
633	CNTF	ENSG00000242689.2	0.24	0.76	0.65	0.25	1.16	0.014
633	ZC3H12A	ENSG00000163874.10	0.56	7.60E-06	0.49	7.90E-05	1.15	2.80E-26
633	AC011498	ENSG00000280239.1	0.72	0.093	0.47	0.29	1.14	6.90E-04
633	ORC1	ENSG00000085840.12	0.4	0.13	0.45	0.06	1.12	5.60E-09
633	RTL3	ENSG00000179300.3	0.62	2	0.37	2	1.12	0.031
633	MIRLET7D	ENSG00000199133.3	0.88	0.11	0.31	0.62	1.12	0.014
633	TAP2	ENSG00000204267.13	0.48	3.50E-04	0.58	6.80E-06	1.12	2.40E-23
633	AC112777	ENSG00000256663.1	0.04	0.96	0.23	0.73	1.12	0.013
633	PTGIS	ENSG00000124212.5	0.76	0.13	0	1	1.11	0.0049
633	SLCO2A1	ENSG00000174640.12	0.56	0.011	-0.01	0.97	1.11	1.20E-09
633	NFAM1	ENSG00000235568.6	-0.27	0.76	0.81	0.18	1.11	0.043
633	SGO2	ENSG00000163535.17	0.51	0.0075	0.34	0.09	1.1	2.80E-12
633	POC1A	ENSG00000164087.7	0.33	0.22	0.45	0.049	1.1	2.20E-09
633	ZNF726	ENSG00000213967.10	0.57	0.3	0.68	0.15	1.1	0.0053
633	NOP16	ENSG00000048162.20	0.34	0.042	0.58	3.10E-05	1.09	3.10E-18
633	CKS2	ENSG00000123975.4	0.35	0.1	0.34	0.082	1.09	3.40E-12
633	XRCC3	ENSG00000126215.13	0.28	0.25	0.52	0.0079	1.09	5.40E-11
633	INCENP	ENSG00000149503.12	0.58	2.00E-05	0.44	0.0016	1.09	4.50E-20
633	PTTG1	ENSG00000164611.12	0.43	0.019	0.36	0.05	1.09	2.30E-13
633	ABCC9	ENSG00000069431.11	-0.28	0.74	0.54	0.37	1.08	0.027
633	DOCK2	ENSG00000134516.15	0.53	0.0032	0.42	0.023	1.08	1.30E-13
633	PLD5	ENSG00000180287.16	0.3	0.68	0.64	0.22	1.08	0.012
633	FANCB	ENSG00000181544.13	0.33	0.47	0.37	0.33	1.08	1.40E-04
633	NCR3LG1	ENSG00000188211.8	0.31	0.07	0.56	5.60E-05	1.08	7.50E-19
633	VPS9D1-A	ENSG00000261373.1	0.5	0.063	0.38	0.15	1.08	1.10E-07
633	RGS3	ENSG00000138835.22	0.52	1.60E-13	-0.1	0.29	1.07	4.30E-61
633	FANCI	ENSG00000140525.17	0.54	4.40E-04	0.48	0.0015	1.07	6.10E-16
633	EPHA2	ENSG00000142627.12	0.49	6.70E-09	0.55	1.50E-11	1.07	8.10E-44
633	TUBA1C	ENSG00000167553.15	0.44	8.90E-05	0.46	1.60E-05	1.07	8.40E-28

633	CHAF1A	ENSG00000167670.15	0.47	4.20E-05	0.45	6.10E-05	1.07	1.10E-26
633	KPNA2	ENSG00000182481.8	0.33	0.012	0.44	2.30E-04	1.07	6.30E-24
633	AP001148	ENSG00000278989.1	0.62	0.25	0.68	0.15	1.07	0.0071
633	CENPN	ENSG00000166451.13	0.38	0.011	0.48	4.70E-04	1.06	1.10E-19
633	NTF3	ENSG00000185652.11	0.45	0.49	0.76	0.15	1.06	0.018
633	DDX12P	ENSG00000214826.5	0.57	0.25	0.61	0.16	1.06	0.0035
633	GEMIN8P	ENSG00000228175.3	0.86	2	-0.24	2	1.06	0.027
633	NGF	ENSG00000134259.3	0.71	0.15	0.11	0.87	1.05	0.007
633	PKN3	ENSG00000160447.6	0.55	0.017	0.54	0.013	1.05	1.40E-08
633	H2AFX	ENSG00000188486.3	0.39	0.0023	0.54	2.60E-06	1.05	2.40E-23
633	TUBA1B	ENSG00000123416.15	0.57	4.30E-06	0.35	0.008	1.04	2.50E-20
633	NFKBIZ	ENSG00000144802.11	0.36	0.023	0.58	2.30E-05	1.04	7.50E-17
633	KIAA1524	ENSG00000163507.13	0.44	0.014	0.47	0.0051	1.04	3.80E-13
633	CDCA4	ENSG00000170779.10	0.45	0.0085	0.4	0.016	1.04	7.50E-14
633	ARRDC3-A	ENSG00000281357.2	0.66	0.3	0.78	0.16	1.04	0.03
633	NIPA1	ENSG00000170113.15	0.58	2.20E-06	0.34	0.012	1.03	3.70E-22
633	NECTIN3	ENSG00000177707.10	0.34	4.60E-07	0.4	5.20E-10	1.03	1.10E-67
633	LINC0045	ENSG00000229373.8	0.82	0.16	0.59	0.3	1.03	0.029
633	AC013451	ENSG00000258425.1	1.25	2	0.79	2	1.03	0.035
633	AC103923	ENSG00000235545.1	0.57	0.18	0.27	0.56	1.02	0.0013
633	NCAPG2	ENSG00000146918.19	0.4	0.0071	0.51	1.70E-04	1.01	8.60E-17
633	CHAF1B	ENSG00000159259.7	0.56	7.50E-04	0.43	0.012	1.01	1.50E-12
633	HHIP	ENSG00000164161.9	0.53	0.16	0.46	0.19	1.01	3.20E-04
633	RBBP8	ENSG00000101773.18	0.4	0.0013	0.44	2.00E-04	1	1.70E-22
633	GPR1	ENSG00000183671.12	0.47	0.43	0.28	0.64	1	0.017
633	MICB	ENSG00000204516.9	0.57	1.30E-04	0.58	4.90E-05	1	5.20E-15
633	MYC	ENSG00000136997.17	0.43	1.50E-05	0.32	0.0019	0.99	7.30E-29
633	GPC5	ENSG00000179399.14	0.93	0.1	0.19	0.79	0.99	0.039
633	DDO	ENSG00000203797.9	0.22	0.68	0.12	0.81	0.99	9.80E-04
633	MTFP1	ENSG00000242114.5	0.4	0.46	0.47	0.31	0.99	0.0059
633	LINC0160	ENSG00000253161.5	0.3	0.35	0.29	0.31	0.99	9.90E-07
633	AL158206	ENSG00000260912.1	0.04	0.96	0.68	0.13	0.99	0.009
633	AC080188	ENSG00000279384.1	0.44	2	0.03	2	0.99	0.038
633	CCNE1	ENSG00000105173.13	0.33	0.29	0.34	0.22	0.98	2.50E-06
633	FGF1	ENSG00000113578.17	0.58	0.0097	0.32	0.19	0.98	1.90E-07
633	EFNB2	ENSG00000125266.6	0.49	1.80E-07	0.26	0.012	0.98	5.80E-31
633	SLC25A19	ENSG00000125454.11	0.15	0.74	0.57	0.042	0.98	3.10E-05
633	AP003356	ENSG00000253669.3	0.55	0.24	0.42	0.34	0.98	0.0042
633	GADD45A	ENSG00000116717.11	0.34	0.0072	0.54	1.30E-06	0.97	1.10E-20
633	ETS1	ENSG00000134954.14	0.23	0.0014	0.42	2.50E-11	0.97	2.00E-64
633	GGH	ENSG00000137563.11	0.43	0.0038	0.5	3.30E-04	0.97	1.90E-15
633	AL138724	ENSG00000272269.1	-0.18	0.6	0.5	0.018	0.97	2.90E-08
633	MGLL	ENSG00000074416.13	0.46	8.90E-06	0.43	2.30E-05	0.96	1.40E-25
633	MND1	ENSG00000121211.7	0.26	0.56	0.38	0.27	0.96	2.40E-04
633	SRGN	ENSG00000122862.4	0.55	0.004	0.31	0.14	0.96	2.90E-09
633	C9orf40	ENSG00000135045.6	0.53	0.0013	0.26	0.18	0.96	4.80E-12
633	CCDC81	ENSG00000149201.9	0.84	0.1	0.86	0.07	0.96	0.024
633	HPDL	ENSG00000186603.5	0.14	0.8	0.54	0.12	0.96	7.80E-04
633	YRDC	ENSG00000196449.3	0.4	0.014	0.5	7.90E-04	0.96	3.40E-13
633	AMD1	ENSG00000123505.15	0.47	2.00E-08	0.32	2.00E-04	0.95	1.70E-35
633	LRRC4	ENSG00000128594.7	0.06	0.94	0.11	0.84	0.95	0.0061
633	CKAP2	ENSG00000136108.14	0.36	0.011	0.34	0.014	0.95	7.30E-17
633	DUSP5	ENSG00000138166.5	0.42	3.10E-06	0.51	3.00E-09	0.95	2.60E-32
633	WDR89	ENSG00000140006.11	0.26	0.15	0.58	3.70E-05	0.95	1.90E-14
633	REL	ENSG00000162924.13	-0.03	0.94	0.44	0.07	0.95	1.00E-06
633	LYPD6	ENSG00000187123.14	0.7	0.14	-0.13	0.83	0.95	0.013
633	AFAP1	ENSG00000196526.10	0.31	5.60E-05	0.48	2.80E-11	0.95	7.10E-45
633	KCND3	ENSG00000171385.9	0.52	2	0.9	2	0.94	0.028
633	PIK3CD	ENSG00000171608.15	0.47	2.30E-06	0.38	2.00E-04	0.94	3.40E-26
633	SLC22A4	ENSG00000197208.5	0.5	2.00E-04	0.49	1.80E-04	0.94	1.50E-16

633	EZR	ENSG00000092820.17	0.45	2.70E-05	0.33	0.0024	0.93	1.30E-22
633	POLA1	ENSG00000101868.10	0.27	0.17	0.41	0.01	0.93	5.20E-12
633	WNT5B	ENSG00000111186.12	0.58	3.20E-12	0.24	0.012	0.93	3.90E-33
633	TMEM106	ENSG00000134291.11	0.35	0.02	0.43	0.0018	0.93	1.10E-14
633	ATP6V0E2	ENSG00000171130.17	0.37	0.0047	0.4	0.0012	0.93	1.10E-18
633	C6orf223	ENSG00000181577.15	0.57	0.24	0.31	0.54	0.93	0.01
633	VRK1	ENSG00000100749.7	0.29	0.16	0.45	0.0097	0.92	2.20E-10
633	USP31	ENSG00000103404.14	0.09	0.66	0.58	5.10E-07	0.92	1.30E-18
633	C17orf53	ENSG00000125319.14	0.41	0.061	0.42	0.04	0.92	2.30E-08
633	TBC1D4	ENSG00000136111.12	0.54	9.60E-12	-0.02	0.88	0.92	2.50E-36
633	SYBU	ENSG00000147642.16	0.48	0.4	-0.19	0.74	0.92	0.018
633	FBXO45	ENSG00000174013.7	0.51	2.00E-04	0.41	0.0037	0.92	5.80E-15
633	PUS1	ENSG00000177192.13	0.56	1.50E-06	0.52	7.60E-06	0.92	1.10E-18
633	AL157394	ENSG00000261438.1	-0.3	0.63	0.52	0.24	0.92	0.01
633	MET	ENSG00000105976.14	0.42	0.0012	0.15	0.31	0.91	4.00E-17
633	NOP2	ENSG00000111641.11	0.43	1.80E-06	0.49	1.00E-08	0.91	5.20E-31
633	ID3	ENSG00000117318.8	0.26	0.11	0.42	0.0022	0.91	1.20E-14
633	LYAR	ENSG00000145220.13	0.35	0.027	0.5	2.60E-04	0.91	6.40E-14
633	ATF3	ENSG00000162772.16	0.34	0.14	0.34	0.11	0.91	4.90E-08
633	MB21D1	ENSG00000164430.15	0.24	0.31	0.41	0.022	0.91	7.00E-10
633	HACD1	ENSG00000165996.13	0.33	0.076	0.37	0.034	0.91	6.10E-11
633	MME	ENSG00000196549.10	-0.03	0.86	0.51	1.50E-07	0.91	6.60E-24
633	ATP10A	ENSG00000206190.11	0.43	0.037	0.51	0.0062	0.91	7.40E-09
633	AC005831	ENSG00000280202.1	0.28	0.68	0.4	0.47	0.91	0.029
633	MYEF2	ENSG00000104177.17	0.23	0.23	0.47	0.0014	0.9	1.00E-12
633	EZH2	ENSG00000106462.10	0.25	0.14	0.36	0.013	0.9	8.90E-14
633	PPIF	ENSG00000108179.13	0.26	5.60E-04	0.57	5.80E-18	0.9	8.90E-47
633	GCNT2	ENSG00000111846.16	0.19	0.65	0.53	0.062	0.9	1.20E-04
633	FBXO5	ENSG00000112029.9	0.12	0.59	0.45	0.0015	0.9	2.50E-13
633	MCM8	ENSG00000125885.13	0.08	0.77	0.48	9.20E-04	0.9	1.40E-12
633	SUV39H2	ENSG00000152455.15	0.32	0.15	0.49	0.0074	0.9	1.00E-08
633	EGFLAM	ENSG00000164318.17	0.39	0.23	0.56	0.037	0.9	8.10E-05
633	PGP	ENSG00000184207.8	0.5	1.10E-04	0.55	7.30E-06	0.9	1.50E-15
633	AC020916	ENSG00000267519.5	0.39	0.012	0.18	0.31	0.9	4.30E-13
633	MCM6	ENSG00000076003.4	0.29	0.017	0.53	4.20E-07	0.89	7.60E-20
633	TRIM14	ENSG00000106785.14	0.41	1.20E-04	0.47	5.80E-06	0.89	1.10E-21
633	NCAPD3	ENSG00000151503.12	0.36	0.019	0.55	6.60E-05	0.89	1.10E-12
633	FXN	ENSG00000165060.11	0.16	0.49	0.52	6.40E-04	0.89	2.80E-11
633	MZT1	ENSG00000204899.5	0.34	0.048	0.45	0.0028	0.89	4.40E-12
633	AC078785	ENSG00000240057.5	-0.54	0.39	0.65	0.18	0.89	0.036
633	HIVEP2	ENSG00000010818.9	0.44	1.10E-05	0.52	4.20E-08	0.88	2.60E-24
633	PTGS2	ENSG00000073756.11	0.42	0.48	0.87	0.064	0.88	0.037
633	SIPA1L3	ENSG00000105738.10	0.42	5.40E-07	0.35	3.80E-05	0.88	4.50E-32
633	TIMELESS	ENSG00000111602.11	0.28	0.013	0.46	3.30E-06	0.88	7.70E-23
633	GINS4	ENSG00000147536.11	0.41	0.0085	0.17	0.34	0.88	7.00E-12
633	PRR22	ENSG00000212123.3	0.17	0.82	0.47	0.34	0.88	0.027
633	HMSD	ENSG00000221887.5	0.41	0.43	0.22	0.67	0.88	0.014
633	TOMM34	ENSG00000025772.7	0.2	0.027	0.36	5.50E-06	0.87	3.50E-34
633	PODXL	ENSG00000128567.16	0.52	0.01	0.57	0.0027	0.87	2.70E-07
633	STK26	ENSG00000134602.15	0.45	0.11	0.24	0.44	0.87	3.90E-05
633	GMPR	ENSG00000137198.9	0.04	0.94	0.28	0.41	0.87	1.90E-04
633	ZNF710	ENSG00000140548.9	0.49	4.00E-07	0.14	0.25	0.87	1.70E-23
633	LMNB2	ENSG00000176619.12	0.38	3.40E-05	0.4	4.40E-06	0.87	4.10E-27
633	WSCD1	ENSG00000179314.13	0.79	0.062	0.25	0.63	0.87	0.015
633	MAFF	ENSG00000185022.11	0.33	0.0056	0.55	2.10E-07	0.87	1.10E-18
633	MARS2	ENSG00000247626.4	0.23	0.44	0.52	0.016	0.87	2.40E-06
633	AC018690	ENSG00000273306.1	0.35	0.59	0.59	0.24	0.87	0.04
633	MCM4	ENSG00000104738.16	0.21	0.18	0.41	0.0012	0.86	7.10E-15
633	AC009533	ENSG00000111788.10	-0.09	0.88	0.27	0.51	0.86	0.0036
633	GMNN	ENSG00000112312.9	0.36	0.035	0.56	1.30E-04	0.86	7.80E-11
633	ARHGEF39	ENSG00000137135.17	0.32	0.58	0.24	0.64	0.86	0.015

633	GPAT3	ENSG00000138678.10	0.44	0.098	0.33	0.2	0.86	1.80E-05
633	PARP1	ENSG00000143799.12	0.39	3.90E-08	0.28	1.40E-04	0.86	2.10E-40
633	LRR1	ENSG00000165501.16	0.19	0.4	0.47	0.0036	0.86	1.20E-09
633	WNT7B	ENSG00000188064.9	-0.61	0.23	0.37	0.43	0.86	0.019
633	AC100861	ENSG00000246582.2	0.14	0.69	0.46	0.053	0.86	1.00E-05
633	AC130371	ENSG00000274370.1	0.42	0.46	0.54	0.25	0.86	0.026
633	DOCK9	ENSG00000088387.18	0.48	8.40E-04	-0.12	0.55	0.85	3.30E-12
633	DMC1	ENSG00000100206.9	0.31	0.58	0.45	0.32	0.85	0.016
633	TANGO6	ENSG00000103047.7	0.44	0.0011	0.37	0.0064	0.85	8.50E-14
633	RRP9	ENSG00000114767.6	0.39	0.011	0.5	3.60E-04	0.85	1.20E-11
633	BRIP1	ENSG00000136492.8	0.39	0.065	0.45	0.019	0.85	2.10E-07
633	MLKL	ENSG00000168404.12	0.49	0.0034	0.53	9.20E-04	0.85	1.50E-09
633	KIF22	ENSG00000079616.12	0.36	0.034	0.42	0.0068	0.84	2.30E-10
633	GPATCH4	ENSG00000160818.16	0.37	0.0023	0.46	4.80E-05	0.84	2.10E-16
633	MB21D2	ENSG00000180611.6	0.35	0.064	0.41	0.015	0.84	4.10E-09
633	ACOT7	ENSG00000097021.19	0.24	0.031	0.34	5.50E-04	0.83	3.50E-22
633	POLR3G	ENSG00000113356.11	0.13	0.72	0.22	0.42	0.83	7.30E-06
633	HEATR3	ENSG00000155393.12	0.38	0.0046	0.43	6.80E-04	0.83	8.80E-14
633	UTP15	ENSG00000164338.9	0.24	0.069	0.52	1.00E-06	0.83	5.40E-18
633	SHMT1	ENSG00000176974.19	0.42	0.11	0.54	0.019	0.83	3.60E-05
633	JPT2	ENSG00000206053.12	0.3	1.20E-05	0.53	1.60E-17	0.83	4.90E-45
633	LYN	ENSG00000254087.7	0.38	0.1	0.22	0.36	0.83	1.30E-06
633	MTHFD1	ENSG00000100714.15	0.3	0.0019	0.45	4.00E-07	0.82	8.30E-24
633	ARHGEF26	ENSG00000114790.12	0.33	0.3	-0.05	0.89	0.82	1.50E-04
633	UAP1	ENSG00000117143.13	0.42	5.90E-08	0.34	8.50E-06	0.82	3.90E-32
633	BORA	ENSG00000136122.15	0.12	0.75	0.21	0.47	0.82	3.50E-05
633	KIF21A	ENSG00000139116.18	0.29	0.53	0.41	0.24	0.82	0.0032
633	MMS22L	ENSG00000146263.11	0.21	0.3	0.47	0.0016	0.82	4.40E-10
633	DUSP2	ENSG00000158050.4	0.5	0.22	0.4	0.29	0.82	0.007
633	NOLC1	ENSG00000166197.16	0.24	0.0031	0.49	2.70E-12	0.82	3.50E-35
633	CYCS	ENSG00000172115.8	0.23	0.064	0.51	5.50E-07	0.82	2.40E-18
633	ZDHHC14	ENSG00000175048.16	0.33	0.22	0.48	0.032	0.82	1.10E-05
633	TNC	ENSG00000041982.15	0.44	2.30E-06	0.56	2.90E-10	0.81	1.10E-21
633	EXOSC5	ENSG00000077348.8	0.26	0.14	0.41	0.0059	0.81	1.40E-10
633	UBE2S	ENSG00000108106.13	0.38	0.0036	0.34	0.0086	0.81	2.50E-13
633	EHD1	ENSG00000110047.17	0.52	3.00E-15	0.21	0.0039	0.81	2.00E-38
633	RHPN2	ENSG00000131941.7	0.36	0.48	0.47	0.26	0.81	0.016
633	IER3	ENSG00000137331.11	0.32	0.018	0.38	0.0024	0.81	1.30E-13
633	CCDC15	ENSG00000149548.14	0	1	0.06	0.89	0.81	0.0011
633	HMGB2	ENSG00000164104.11	0.3	0.074	0.22	0.19	0.81	1.20E-10
633	SERPINB8	ENSG00000166401.14	0.23	0.041	0.48	2.50E-07	0.81	1.40E-21
633	C5orf34	ENSG00000172244.8	0.28	0.52	0.58	0.069	0.81	0.0033
633	PLA2G16	ENSG00000176485.11	0.46	0.011	0.56	8.60E-04	0.81	6.10E-08
633	RFLNB	ENSG00000183688.4	0.11	0.5	0.31	0.0064	0.81	9.60E-17
633	SCFD2	ENSG00000184178.15	0.51	7.50E-05	0.33	0.018	0.81	1.00E-12
633	KIAA1671	ENSG00000197077.13	0.06	0.95	0.63	0.16	0.81	0.039
633	FARSB	ENSG00000116120.9	0.28	0.0052	0.49	3.00E-08	0.8	2.10E-22
633	MCM7	ENSG00000166508.17	0.18	0.12	0.48	3.60E-08	0.8	1.00E-22
633	GREM1	ENSG00000166923.10	0.04	0.84	0.36	8.20E-04	0.8	1.80E-17
633	SNHG15	ENSG00000232956.8	0.3	0.023	0.39	0.0014	0.8	7.50E-15
633	SNHG3	ENSG00000242125.3	0.37	1.90E-05	0.37	1.40E-05	0.8	1.80E-26
633	AC132872	ENSG00000280407.2	0.18	0.75	0.5	0.2	0.8	0.013
633	CXCL2	ENSG00000081041.8	0.28	0.64	0.68	0.11	0.79	0.03
633	DNMT1	ENSG00000130816.14	0.2	0.022	0.47	4.00E-10	0.79	2.60E-29
633	ABCE1	ENSG00000164163.10	0.24	0.01	0.49	3.70E-10	0.79	1.10E-26
633	MIR222H	ENSG00000270069.1	0.26	0.041	0.49	7.50E-06	0.79	9.30E-16
633	RFC2	ENSG00000049541.10	0.43	8.10E-05	0.58	1.50E-08	0.78	7.50E-16
633	HIVEP1	ENSG00000095951.16	0.41	6.60E-07	0.52	3.40E-11	0.78	8.00E-26
633	SUV39H1	ENSG00000101945.16	0.09	0.74	0.36	0.035	0.78	2.70E-08
633	STEAP1B	ENSG00000105889.14	0.42	0.26	0.05	0.91	0.78	0.0042
633	NIP7	ENSG00000132603.13	0.26	0.018	0.44	3.30E-06	0.78	4.60E-20

633	C11orf70	ENSG00000137691.12	0.26	0.34	-0.05	0.88	0.78	1.30E-05
633	PPRC1	ENSG00000148840.10	0.35	2.00E-05	0.42	6.80E-08	0.78	9.80E-27
633	DSN1	ENSG00000149636.15	0.18	0.35	0.4	0.0046	0.78	6.60E-11
633	SLC16A12	ENSG00000152779.13	0.5	0.11	0.19	0.6	0.78	0.0019
633	MAMDC2	ENSG00000165072.9	0.03	0.9	0.56	2.00E-06	0.78	4.20E-13
633	SMCO4	ENSG00000166002.6	0.3	0.29	0.44	0.057	0.78	4.60E-05
633	BNC1	ENSG00000169594.13	0.24	0.52	0.25	0.42	0.78	4.80E-04
633	AKAP5	ENSG00000179841.8	0.5	0.27	0.64	0.095	0.78	0.02
633	ZNF257	ENSG00000197134.11	0.07	0.93	-0.03	0.97	0.78	0.02
633	PLAUR	ENSG00000011422.11	0.28	0.0098	0.33	7.60E-04	0.77	9.30E-19
633	KITLG	ENSG00000049130.14	0.2	0.023	0.37	3.90E-07	0.77	1.60E-29
633	L2HGDH	ENSG00000087299.11	0.35	0.16	0.44	0.048	0.77	2.40E-05
633	SH2D4A	ENSG00000104611.11	0.49	0.0016	0.41	0.0071	0.77	7.00E-09
633	PPAT	ENSG00000128059.8	0.2	0.17	0.39	8.60E-04	0.77	6.50E-14
633	KRT7	ENSG00000135480.15	0.31	0.062	0.32	0.038	0.77	2.20E-09
633	DOPEY2	ENSG00000142197.12	0.22	0.055	0.48	2.70E-07	0.77	1.10E-19
633	C16orf59	ENSG00000162062.14	0.51	0.026	0.42	0.067	0.77	6.00E-05
633	WDR43	ENSG00000163811.11	0.3	1.20E-04	0.39	6.90E-08	0.77	2.00E-30
633	LRRC8C	ENSG00000171488.14	0.52	8.70E-10	0.32	4.00E-04	0.77	5.80E-22
633	AEN	ENSG00000181026.14	0.26	0.0011	0.53	8.20E-14	0.77	1.80E-30
633	LIN9	ENSG00000183814.15	0.38	0.14	0.22	0.42	0.77	8.00E-05
633	HYLS1	ENSG00000198331.10	0.28	0.16	0.28	0.13	0.77	7.60E-08
633	CENPW	ENSG00000203760.8	0.36	0.21	0.32	0.23	0.77	2.40E-04
633	ZNF826P	ENSG00000231205.11	0.51	0.078	0.35	0.24	0.77	8.90E-04
633	RPL36A	ENSG00000241343.9	0.34	0.055	0.42	0.0072	0.77	8.70E-09
633	KCNN4	ENSG00000104783.11	0.37	0.024	0.31	0.055	0.76	9.50E-09
633	PRLR	ENSG00000113494.16	0.51	0.29	0.55	0.2	0.76	0.036
633	KNSTRN	ENSG00000128944.13	0.27	0.15	0.24	0.19	0.76	2.00E-08
633	PCNA	ENSG00000132646.10	0.02	0.92	0.54	3.50E-06	0.76	3.80E-12
633	DSCC1	ENSG00000136982.5	0.14	0.71	0.34	0.2	0.76	3.20E-04
633	ZGRF1	ENSG00000138658.15	0.04	0.93	0.38	0.061	0.76	5.60E-06
633	TMEM87B	ENSG00000153214.9	0.37	3.50E-07	0.32	8.70E-06	0.76	6.40E-32
633	USP1	ENSG00000162607.12	0.3	0.0043	0.43	8.50E-06	0.76	3.90E-18
633	HSPB7	ENSG00000173641.17	0.54	0.0067	0.07	0.8	0.76	7.00E-06
633	ANXA2	ENSG00000182718.16	0.26	0.0017	0.34	1.50E-05	0.76	1.50E-26
633	MAP2K3	ENSG00000034152.18	0.24	0.0014	0.4	2.90E-09	0.75	1.30E-32
633	SPDL1	ENSG00000040275.16	0.2	0.32	0.35	0.026	0.75	8.60E-09
633	TARBP1	ENSG00000059588.9	0.23	0.22	0.31	0.05	0.75	9.50E-09
633	SGK3	ENSG00000104205.12	0.07	0.87	0.45	0.05	0.75	1.10E-04
633	E2F3	ENSG00000112242.14	0.43	2.60E-07	0.39	3.60E-06	0.75	1.30E-23
633	CIT	ENSG00000122966.15	0.51	0.0041	0.02	0.94	0.75	6.70E-07
633	PPAN	ENSG00000130810.19	0.31	0.29	0.42	0.083	0.75	1.70E-04
633	PSMC3IP	ENSG00000131470.14	0.25	0.29	0.37	0.051	0.75	1.90E-06
633	EXOC6	ENSG00000138190.16	0.19	0.29	0.53	4.30E-05	0.75	1.90E-10
633	LYSMD2	ENSG00000140280.13	0.03	0.95	0.32	0.17	0.75	3.80E-05
633	FAM86C1	ENSG00000158483.15	0.13	0.6	0.43	0.0068	0.75	3.00E-08
633	RAB3B	ENSG00000169213.6	0.36	2.40E-04	0.52	4.40E-09	0.75	5.90E-19
633	THOP1	ENSG00000172009.14	0.37	1.60E-06	0.39	2.30E-07	0.75	1.00E-27
633	PFAS	ENSG00000178921.13	0.33	7.70E-04	0.5	1.00E-08	0.75	6.60E-20
633	PLD6	ENSG00000179598.5	0.31	0.28	0.2	0.47	0.75	1.10E-04
633	ZDHHC23	ENSG00000184307.14	0.37	0.45	0.42	0.31	0.75	0.023
633	S100A3	ENSG00000188015.9	0.16	0.68	0.52	0.032	0.75	3.50E-04
633	HACD2	ENSG00000206527.9	0.34	1.60E-04	0.53	1.00E-10	0.75	1.70E-22
633	EHD4	ENSG00000103966.10	0.39	5.60E-04	0.31	0.0063	0.74	4.40E-14
633	CAP2	ENSG00000112186.11	0.34	0.14	0.3	0.17	0.74	1.30E-05
633	POPDC3	ENSG00000132429.9	0.32	0.022	0.51	3.10E-05	0.74	3.70E-11
633	ALPK3	ENSG00000136383.6	0.46	0.32	0.04	0.95	0.74	0.03
633	AGPAT5	ENSG00000155189.11	0.36	9.60E-04	0.36	8.20E-04	0.74	1.20E-15
633	ATP5G1	ENSG00000159199.13	0.19	0.21	0.41	7.20E-04	0.74	6.80E-12
633	RFWD3	ENSG00000168411.13	0.19	0.14	0.45	6.90E-06	0.74	6.30E-16
633	KBTBD11	ENSG00000176595.3	0.6	0.2	0.57	0.18	0.74	0.047

633	MYO1D	ENSG00000176658.16	0.58	0.036	0.2	0.56	0.74	0.0015
633	AC008560	ENSG00000253251.2	0.41	0.42	0.04	0.95	0.74	0.035
633	PFKP	ENSG00000067057.16	0.48	3.40E-10	0.32	6.50E-05	0.73	1.00E-24
633	PPP2R3A	ENSG00000073711.10	0.25	0.0016	0.17	0.037	0.73	1.60E-28
633	POP1	ENSG00000104356.10	0.33	0.0069	0.41	2.80E-04	0.73	2.70E-13
633	GALNT7	ENSG00000109586.11	0.39	7.50E-07	0.15	0.1	0.73	6.10E-25
633	EIF5A	ENSG00000132507.17	0.21	0.0017	0.39	1.10E-11	0.73	4.40E-40
633	PLCL2	ENSG00000154822.17	0.15	0.51	0.56	7.30E-05	0.73	1.20E-08
633	LZTS1	ENSG00000061337.15	0.31	0.48	0.06	0.9	0.72	0.014
633	CYLD	ENSG00000083799.17	-0.04	0.75	0.53	4.10E-14	0.72	1.10E-26
633	PYCR3	ENSG00000104524.13	0.32	0.043	0.41	0.0035	0.72	3.10E-09
633	NSD2	ENSG00000109685.17	0.26	0.014	0.28	0.0045	0.72	3.20E-17
633	AMOTL2	ENSG00000114019.14	0.47	1.90E-06	0.25	0.021	0.72	1.20E-15
633	DKC1	ENSG00000130826.17	0.21	0.027	0.38	1.90E-06	0.72	1.30E-23
633	AMIGO2	ENSG00000139211.6	0.25	0.14	0.53	6.80E-05	0.72	3.40E-09
633	AFF3	ENSG00000144218.18	0.2	0.04	0.46	5.50E-09	0.72	2.20E-23
633	VPS37C	ENSG00000167987.10	0.18	0.039	0.56	4.90E-16	0.72	1.80E-27
633	MTHFD2	ENSG00000065911.11	0.46	3.50E-04	0.31	0.019	0.71	4.70E-10
633	POLD3	ENSG00000077514.8	0.19	0.21	0.43	3.10E-04	0.71	1.80E-11
633	XYLT1	ENSG00000103489.11	0.21	0.12	0.56	3.10E-08	0.71	7.00E-14
633	MORC4	ENSG00000133131.14	0.24	0.01	0.39	1.60E-06	0.71	3.60E-21
633	IP6K3	ENSG00000161896.11	-0.21	0.51	0.52	0.014	0.71	1.30E-04
633	SFMBT1	ENSG00000163935.13	0.2	0.68	0.34	0.32	0.71	0.0082
633	PPA1	ENSG00000180817.11	0.48	5.90E-08	0.22	0.023	0.71	2.40E-18
633	GJD3	ENSG00000183153.6	0.51	0.071	0.53	0.044	0.71	0.0018
633	TRAIP	ENSG00000183763.8	0.2	0.59	0.37	0.18	0.71	0.002
633	ENO1	ENSG00000074800.13	0.28	1.10E-04	0.34	5.30E-07	0.7	1.80E-28
633	ITGA6	ENSG00000091409.14	0.25	0.037	0.44	1.10E-05	0.7	9.70E-14
633	SEC23B	ENSG00000101310.14	0.21	0.0061	0.53	2.60E-17	0.7	2.60E-31
633	PEX5L	ENSG00000114757.18	-0.27	0.56	0.3	0.41	0.7	0.011
633	PSMD14	ENSG00000115233.11	0.2	0.05	0.44	2.10E-07	0.7	5.30E-19
633	FAM110A	ENSG00000125898.12	0.12	0.77	0.46	0.055	0.7	6.90E-04
633	SLC25A4	ENSG00000151729.10	0.4	0.0062	0.45	9.80E-04	0.7	1.00E-08
633	TONSL	ENSG00000160949.16	0.34	0.013	0.41	8.40E-04	0.7	2.50E-10
633	RAC3	ENSG00000169750.8	0.54	4.20E-05	0.15	0.39	0.7	5.30E-09
633	3-Mar	ENSG00000173926.5	0.1	0.81	0.18	0.58	0.7	0.0017
633	STAP2	ENSG00000178078.11	-0.13	0.58	0.52	2.30E-04	0.7	9.10E-08
633	NPB	ENSG00000183979.7	0.4	0.39	0.44	0.27	0.7	0.037
633	VEPH1	ENSG00000197415.11	0.54	2.50E-04	0.19	0.29	0.7	1.20E-07
633	HMBS	ENSG00000256269.7	0.31	0.041	0.33	0.018	0.7	3.00E-09
633	ORAI1	ENSG00000276045.2	0.23	0.065	0.47	4.50E-06	0.7	9.70E-14
633	POLD1	ENSG00000062822.12	0.2	0.18	0.4	9.10E-04	0.69	1.20E-10
633	RANGAP1	ENSG00000100401.19	0.19	0.1	0.54	2.20E-09	0.69	1.20E-15
633	BRIX1	ENSG00000113460.12	0.23	0.089	0.3	0.01	0.69	6.80E-12
633	SRM	ENSG00000116649.9	0.35	4.90E-06	0.4	5.50E-08	0.69	1.70E-24
633	CHAC1	ENSG00000128965.11	0.5	0.012	0.53	0.0043	0.69	4.00E-05
633	TOMM40	ENSG00000130204.12	0.17	0.12	0.43	5.90E-07	0.69	5.50E-18
633	GCSH	ENSG00000140905.10	0.31	0.55	0.24	0.61	0.69	0.04
633	MYO10	ENSG00000145555.14	0.47	8.50E-12	0.13	0.12	0.69	7.00E-26
633	ROBO4	ENSG00000154133.14	0.42	0.16	0.38	0.18	0.69	0.0025
633	JPT1	ENSG00000189159.15	0.3	0.0014	0.21	0.026	0.69	4.30E-18
633	LIN52	ENSG00000205659.10	0.11	0.71	0.15	0.48	0.69	2.60E-06
633	ITPRIPL2	ENSG00000205730.6	0.45	5.00E-14	0.3	1.60E-06	0.69	8.70E-34
633	PGAM5	ENSG00000247077.6	0.2	0.15	0.47	7.80E-06	0.69	1.80E-12
633	TDP1	ENSG00000042088.13	0.18	0.14	0.26	0.012	0.68	5.30E-15
633	PRR11	ENSG00000068489.12	0.3	0.026	-0.03	0.86	0.68	2.60E-10
633	ME2	ENSG00000082212.12	0.17	0.11	0.48	4.00E-09	0.68	7.20E-19
633	PSMA3	ENSG00000100567.12	0.24	0.0093	0.35	2.60E-05	0.68	3.90E-19
633	C1GALT1	ENSG00000106392.10	0.2	0.16	0.33	0.0042	0.68	1.70E-11
633	PLXNA1	ENSG00000114554.11	0.18	0.02	0.57	4.30E-19	0.68	2.00E-28
633	FAM98A	ENSG00000119812.18	0.27	1.10E-04	0.35	1.70E-07	0.68	1.50E-28

633	TRIM25	ENSG00000121060.16	0.36	8.50E-12	0.45	6.60E-19	0.68	2.10E-44
633	TEX2	ENSG00000136478.7	0.52	4.40E-14	0.13	0.13	0.68	1.10E-24
633	PNPT1	ENSG00000138035.14	0.21	0.11	0.32	0.0039	0.68	1.20E-12
633	SLC16A3	ENSG00000141526.16	0.27	9.60E-05	0.55	9.80E-19	0.68	1.80E-29
633	DDAH1	ENSG00000153904.18	0.23	0.077	0.54	1.40E-07	0.68	1.70E-12
633	SNRPD1	ENSG00000167088.10	0.21	0.12	0.36	0.0012	0.68	5.00E-12
633	SYNM	ENSG00000182253.14	0.52	1.50E-04	0.02	0.91	0.68	3.30E-08
633	ALYREF	ENSG00000183684.7	0.29	9.80E-04	0.37	6.90E-06	0.68	5.40E-20
633	PSG5	ENSG00000204941.13	-0.06	0.93	0.26	0.54	0.68	0.035
633	KLHL23	ENSG00000213160.9	0.12	0.75	0.35	0.18	0.68	0.001
633	BID	ENSG00000015475.18	0.08	0.58	0.48	3.50E-08	0.67	2.20E-16
633	MRTO4	ENSG00000053372.4	0.19	0.13	0.3	0.0036	0.67	5.80E-14
633	MXD1	ENSG00000059728.10	0.51	9.70E-04	0.3	0.079	0.67	8.30E-07
633	TM7SF3	ENSG00000064115.10	0.15	0.15	0.49	4.50E-10	0.67	4.60E-20
633	PKM	ENSG00000067225.17	0.16	0.017	0.5	2.50E-19	0.67	5.00E-36
633	MCAM	ENSG00000076706.16	0.5	1.10E-05	0.37	0.0018	0.67	1.10E-10
633	SLC7A6	ENSG00000103064.13	0.34	1.20E-04	0.35	3.60E-05	0.67	2.60E-18
633	SPAG1	ENSG00000104450.12	0.45	0.21	0.46	0.16	0.67	0.014
633	RNASEH2A	ENSG00000104889.5	0.15	0.44	0.26	0.091	0.67	5.60E-08
633	LRAT	ENSG00000121207.11	0.5	0.012	0	0.99	0.67	8.00E-05
633	SDC4	ENSG00000124145.6	0.18	0.011	0.55	2.60E-21	0.67	2.00E-32
633	EEF1E1	ENSG00000124802.11	0.21	0.4	0.41	0.033	0.67	3.50E-05
633	ARHGAP22	ENSG00000128805.14	0.09	0.48	0.56	2.20E-11	0.67	6.40E-17
633	TUBB2A	ENSG00000137267.5	0.48	2.60E-05	0.15	0.28	0.67	8.30E-11
633	BEND6	ENSG00000151917.17	0.55	3.50E-08	0.19	0.12	0.67	5.70E-13
633	BOLA3	ENSG00000163170.11	0.22	0.38	0.27	0.19	0.67	2.60E-05
633	DUSP7	ENSG00000164086.9	0.38	6.70E-08	0.25	7.40E-04	0.67	3.90E-26
633	SLC25A33	ENSG00000171612.6	0.16	0.46	0.22	0.21	0.67	3.10E-07
633	PDE12	ENSG00000174840.8	0.23	0.019	0.45	6.90E-08	0.67	9.20E-19
633	RUVBL1	ENSG00000175792.11	0.26	3.20E-04	0.18	0.012	0.67	1.70E-28
633	SLC25A22	ENSG00000177542.10	0.44	1.30E-07	0.33	1.60E-04	0.67	1.70E-18
633	RFX8	ENSG00000196460.12	0.35	0.19	0.52	0.018	0.67	7.50E-04
633	NUP62	ENSG00000213024.11	0.05	0.64	0.53	3.20E-21	0.67	2.60E-35
633	C10orf55	ENSG00000222047.8	0.18	0.72	0.4	0.24	0.67	0.015
633	AC116347	ENSG00000238000.1	-0.39	0.44	0.16	0.73	0.67	0.036
633	ZNF280B	ENSG00000275004.3	0.26	0.58	0.34	0.36	0.67	0.022
633	NRIP2	ENSG00000053702.14	0.09	0.84	0.15	0.68	0.66	0.0051
633	RRP15	ENSG00000067533.5	0.23	0.079	0.41	2.10E-04	0.66	3.80E-11
633	TIGAR	ENSG00000078237.6	0.02	0.91	0.54	1.80E-07	0.66	1.20E-11
633	NUP188	ENSG00000095319.14	0.28	0.001	0.44	8.50E-09	0.66	1.50E-19
633	SAMHD1	ENSG00000101347.8	0.24	0.047	0.39	2.70E-04	0.66	3.20E-12
633	DCLRE1B	ENSG00000118655.4	0.07	0.79	0.36	0.012	0.66	5.10E-08
633	UCHL3	ENSG00000118939.17	0.18	0.59	0.37	0.12	0.66	7.40E-04
633	UTP20	ENSG00000120800.4	0.31	1.40E-04	0.48	6.90E-11	0.66	2.40E-21
633	FIGNL1	ENSG00000132436.11	0.23	0.17	0.46	5.50E-04	0.66	2.10E-08
633	LDHA	ENSG00000134333.13	0.25	3.20E-04	0.41	2.20E-11	0.66	5.90E-29
633	TTLL4	ENSG00000135912.10	0.07	0.67	0.5	1.50E-08	0.66	3.60E-15
633	HERC6	ENSG00000138642.14	0.3	0.46	0.16	0.69	0.66	0.018
633	COL6A2	ENSG00000142173.14	0.5	6.00E-11	0.53	3.60E-12	0.66	9.60E-20
633	CNOT9	ENSG00000144580.13	0.23	6.80E-04	0.4	1.10E-11	0.66	2.60E-32
633	SCLT1	ENSG00000151466.11	0.2	0.11	0.29	0.0071	0.66	3.90E-13
633	KBTBD8	ENSG00000163376.11	0.62	0.066	0.18	0.66	0.66	0.021
633	DNAJC9	ENSG00000213551.4	0.23	0.079	0.22	0.078	0.66	3.40E-11
633	LIG1	ENSG00000105486.13	0.08	0.68	0.35	0.0042	0.65	2.00E-09
633	ODC1	ENSG00000115758.12	0.25	0.053	0.2	0.1	0.65	5.30E-11
633	DHCR24	ENSG00000116133.11	0.57	3.90E-08	0.14	0.28	0.65	2.30E-11
633	DOCK10	ENSG00000135905.18	0.24	0.0028	0.46	2.40E-11	0.65	4.70E-24
633	LMO7	ENSG00000136153.19	0.27	0.038	0.25	0.039	0.65	1.20E-10
633	TMEM14B	ENSG00000137210.13	0	0.99	0.39	1.70E-05	0.65	3.40E-15
633	TAPBPL	ENSG00000139192.11	0.19	0.27	0.53	4.50E-05	0.65	4.40E-08
633	SLC9B2	ENSG00000164038.14	0.29	0.03	0.47	3.80E-05	0.65	3.30E-10

633	OTUD4	ENSG00000164164.15	0.15	0.093	0.43	2.70E-10	0.65	2.60E-24
633	CDT1	ENSG00000167513.8	0.27	0.047	0.32	0.011	0.65	7.50E-10
633	RILP	ENSG00000167705.11	0.24	0.3	0.25	0.21	0.65	2.20E-05
633	DTYMK	ENSG00000168393.12	0.26	0.056	0.16	0.25	0.65	9.00E-10
633	DCLK2	ENSG00000170390.15	0	1	0.24	0.14	0.65	3.10E-07
633	NEGR1	ENSG00000172260.14	0.37	6.90E-04	0.47	2.40E-06	0.65	2.30E-12
633	AC098864	ENSG00000177822.7	0.53	0.051	0.04	0.92	0.65	0.0048
633	EIF4EBP1	ENSG00000187840.4	0.27	0.099	0.1	0.59	0.65	2.00E-07
633	HNRNPAB	ENSG00000197451.11	0.19	0.013	0.34	4.10E-07	0.65	1.40E-25
633	GPX1P1	ENSG00000197582.5	0.11	0.58	0.34	0.0077	0.65	3.80E-09
633	TRIM16	ENSG00000221926.11	0.26	0.0056	0.56	1.00E-12	0.65	1.50E-18
633	SSH1	ENSG00000084112.14	0.37	9.60E-10	0.3	6.10E-07	0.64	2.80E-30
633	MMP11	ENSG00000099953.9	0.21	0.27	0.25	0.13	0.64	2.00E-06
633	PPFIBP1	ENSG00000110841.13	0.13	0.077	0.24	7.50E-05	0.64	3.40E-32
633	PAK1IP1	ENSG00000111845.4	0.1	0.59	0.45	5.20E-05	0.64	3.80E-10
633	HABP4	ENSG00000130956.13	0.27	9.20E-04	0.33	1.00E-05	0.64	9.00E-21
633	NXT1	ENSG00000132661.3	0.11	0.59	0.49	1.90E-04	0.64	9.10E-08
633	LTV1	ENSG00000135521.8	0.12	0.31	0.39	4.90E-06	0.64	1.50E-16
633	SERTAD1	ENSG00000197019.4	0.15	0.39	0.4	8.20E-04	0.64	2.50E-09
633	ASH1L-AS	ENSG00000235919.4	0.26	0.55	0.45	0.15	0.64	0.016
633	METTL1	ENSG00000037897.16	0.36	0.013	0.47	3.00E-04	0.63	8.40E-08
633	NFKBIB	ENSG00000104825.16	0.1	0.61	0.46	7.50E-05	0.63	5.70E-09
633	GRWD1	ENSG00000105447.12	0.26	0.018	0.46	9.50E-07	0.63	1.00E-12
633	BCL7A	ENSG00000110987.8	-0.16	0.35	0.44	2.10E-04	0.63	1.60E-09
633	ICMT	ENSG00000116237.15	0.3	1.50E-04	0.43	5.90E-09	0.63	7.60E-20
633	FABP3	ENSG00000121769.7	0.56	0.013	-0.15	0.58	0.63	0.0017
633	GTF3A	ENSG00000122034.14	0.26	0.0058	0.33	1.50E-04	0.63	4.90E-16
633	INHBA	ENSG00000122641.10	0.33	4.30E-04	0.54	1.30E-10	0.63	2.50E-15
633	SNRPA1	ENSG00000131876.16	0.3	6.40E-04	0.36	1.20E-05	0.63	6.10E-18
633	RRAS2	ENSG00000133818.13	0.13	0.31	0.4	7.80E-06	0.63	3.00E-14
633	NUDT15	ENSG00000136159.3	0.09	0.62	0.28	0.029	0.63	1.70E-09
633	ZNF365	ENSG00000138311.15	0.07	0.89	0.28	0.42	0.63	0.02
633	UTP4	ENSG00000141076.17	0.19	0.017	0.4	2.90E-09	0.63	4.20E-24
633	SRFBP1	ENSG00000151304.5	0.26	0.15	0.39	0.013	0.63	2.60E-06
633	KLF10	ENSG00000155090.14	0.04	0.83	0.45	2.80E-07	0.63	3.20E-14
633	AZIN1	ENSG00000155096.13	0.21	0.0056	0.42	1.30E-10	0.63	9.10E-25
633	IRAK1	ENSG00000184216.13	0.27	2.10E-04	0.29	2.20E-05	0.63	8.80E-24
633	TAF13	ENSG00000197780.9	0.22	0.079	0.53	1.70E-07	0.63	2.40E-11
633	SELENOT	ENSG00000198843.12	0.16	0.058	0.44	1.80E-10	0.63	1.10E-21
633	C4orf46	ENSG00000205208.4	0.06	0.77	0.28	0.03	0.63	4.80E-09
633	DHFR	ENSG00000228716.6	0.22	0.19	0.22	0.15	0.63	1.60E-07
633	TMPO-AS	ENSG00000257167.2	-0.19	0.56	0.09	0.78	0.63	9.50E-04
633	AC010168	ENSG00000261324.2	0.35	0.41	0	1	0.63	0.037
633	MRPL12	ENSG00000262814.7	0.13	0.52	0.2	0.2	0.63	1.40E-07
633	TTLL12	ENSG00000100304.12	0.21	0.014	0.28	3.10E-04	0.62	1.80E-19
633	LOX	ENSG00000113083.13	0.31	1.50E-05	0.26	2.20E-04	0.62	6.20E-23
633	CHTF18	ENSG00000127586.16	0.46	0.0072	0.44	0.0073	0.62	2.80E-05
633	LRP12	ENSG00000147650.11	0.44	4.60E-06	0.03	0.86	0.62	4.60E-13
633	NUP205	ENSG00000155561.14	0.26	0.0011	0.28	3.30E-04	0.62	1.00E-19
633	FDPS	ENSG00000160752.14	0.48	4.90E-06	0.01	0.94	0.62	2.40E-10
633	ZMYND19	ENSG00000165724.5	0.28	0.014	0.37	2.90E-04	0.62	1.30E-11
633	CKS1B	ENSG00000173207.12	0.19	0.34	0.14	0.44	0.62	2.30E-06
633	MAK16	ENSG00000198042.10	0.2	0.17	0.38	0.0011	0.62	2.90E-09
633	SLC5A3	ENSG00000198743.6	0.19	0.2	0.24	0.057	0.62	1.60E-09
633	RAI14	ENSG00000039560.13	0.2	0.0073	0.39	3.10E-09	0.61	1.70E-22
633	AIFM2	ENSG00000042286.14	0.48	9.10E-07	0.3	0.0043	0.61	1.30E-11
633	USP13	ENSG00000058056.8	0.28	0.016	0.33	0.002	0.61	8.90E-11
633	HAGH	ENSG00000063854.12	0.05	0.81	0.42	1.50E-05	0.61	3.00E-12
633	UHRF1BP1	ENSG00000065060.16	0.17	0.31	0.58	1.30E-06	0.61	6.20E-08
633	ATP8B1	ENSG00000081923.11	0.29	0.035	0.19	0.19	0.61	3.80E-08
633	NOP56	ENSG00000101361.16	0.24	0.002	0.34	1.20E-06	0.61	4.20E-21

633	CSTF2	ENSG00000101811.13	0.32	0.0082	0.3	0.013	0.61	1.70E-09
633	ERCC2	ENSG00000104884.14	0.28	0.0064	0.27	0.0048	0.61	3.90E-13
633	GTPBP4	ENSG00000107937.18	0.25	0.0016	0.37	1.30E-07	0.61	1.00E-20
633	PARP2	ENSG00000129484.13	0.19	0.22	0.18	0.22	0.61	1.60E-08
633	RAN	ENSG00000132341.11	0.16	0.11	0.31	6.60E-05	0.61	1.30E-17
633	MFHAS1	ENSG00000147324.10	-0.08	0.54	0.37	2.00E-05	0.61	5.30E-15
633	POLE3	ENSG00000148229.12	0.16	0.25	0.34	8.60E-04	0.61	1.90E-11
633	CCT5	ENSG00000150753.11	0.22	0.0025	0.31	2.40E-06	0.61	3.40E-24
633	WDR66	ENSG00000158023.9	0.36	0.031	0.34	0.035	0.61	4.90E-06
633	IER5	ENSG00000162783.10	0.38	5.80E-06	0.2	0.024	0.61	9.80E-16
633	FASN	ENSG00000169710.8	0.57	2.70E-09	0.13	0.28	0.61	2.60E-11
633	TUBB6	ENSG00000176014.12	0.42	1.50E-05	0.22	0.042	0.61	5.50E-12
633	POLE	ENSG00000177084.16	0.25	0.041	0.25	0.029	0.61	1.30E-10
633	EMC3-AS	ENSG00000180385.8	0.4	0.19	0.39	0.16	0.61	0.0094
633	PRIM1	ENSG00000198056.13	0.04	0.91	0.26	0.28	0.61	0.001
633	ACACA	ENSG00000278540.4	0.4	7.70E-12	0.15	0.03	0.61	1.00E-28
633	GGCT	ENSG00000006625.17	0.17	0.4	0.38	0.011	0.6	3.60E-06
633	SNRNP40	ENSG00000060688.12	0.23	0.0076	0.29	1.70E-04	0.6	9.90E-19
633	POLR1A	ENSG00000068654.15	0.28	4.00E-05	0.36	3.00E-08	0.6	2.40E-23
633	PAG1	ENSG00000076641.4	-0.15	0.5	0.46	0.0015	0.6	5.10E-06
633	NFE2L1	ENSG00000082641.15	0.29	4.30E-07	0.34	7.80E-10	0.6	5.00E-30
633	BBC3	ENSG00000105327.17	0.18	0.089	0.41	1.50E-06	0.6	2.60E-14
633	TWNK	ENSG00000107815.7	0.27	0.047	0.41	3.70E-04	0.6	7.40E-09
633	CORO1C	ENSG00000110880.10	0.34	1.30E-06	0.26	2.80E-04	0.6	2.40E-21
633	RFC5	ENSG00000111445.13	0.11	0.66	0.24	0.17	0.6	1.20E-05
633	NUDCD1	ENSG00000120526.10	0	1	0.32	0.011	0.6	1.60E-08
633	CDKN2C	ENSG00000123080.10	0.21	0.35	-0.04	0.89	0.6	7.30E-05
633	CSE1L	ENSG00000124207.16	0.13	0.29	0.26	0.0041	0.6	1.00E-14
633	SNRPB	ENSG00000125835.18	0.19	0.077	0.32	4.30E-04	0.6	3.70E-13
633	YWHAH	ENSG00000128245.14	0.3	2.40E-04	0.21	0.01	0.6	3.30E-17
633	TLNRD1	ENSG00000140406.3	0.38	9.60E-04	0.32	0.004	0.6	1.00E-09
633	SRPRB	ENSG00000144867.11	0.21	0.028	0.35	2.40E-05	0.6	1.60E-15
633	HAUS6	ENSG00000147874.10	0.27	0.023	0.26	0.022	0.6	1.70E-10
633	QTRT2	ENSG00000151576.10	0.22	0.053	0.37	1.10E-04	0.6	1.70E-12
633	NRGN	ENSG00000154146.12	0.35	0.11	0.22	0.33	0.6	4.60E-04
633	NAA15	ENSG00000164134.12	0.17	0.014	0.35	1.50E-09	0.6	4.60E-29
633	MID1IP1	ENSG00000165175.15	0.2	0.0012	0.08	0.23	0.6	1.40E-32
633	SLC19A1	ENSG00000173638.18	0.2	0.19	0.35	0.0037	0.6	1.30E-08
633	ZWILCH	ENSG00000174442.11	0.35	0.0056	0.28	0.03	0.6	1.80E-08
633	FOXC2	ENSG00000176692.5	0.35	1.80E-06	0.07	0.5	0.6	3.70E-20
633	GCNT1	ENSG00000187210.12	0.47	1.20E-04	-0.11	0.5	0.6	4.90E-08
633	HSPA14	ENSG00000187522.14	0.28	0.078	0.31	0.035	0.6	6.00E-07
633	ANKRD34A	ENSG00000272031.2	0.5	0.077	0.19	0.56	0.6	0.01
633	DPF1	ENSG00000011332.19	0.31	0.3	-0.21	0.5	0.59	0.0074
633	DDX20	ENSG00000064703.11	0.18	0.14	0.25	0.017	0.59	4.90E-12
633	RAD18	ENSG00000070950.9	0.05	0.83	0.11	0.5	0.59	3.20E-08
633	PUM3	ENSG00000080608.9	0.19	0.049	0.3	2.80E-04	0.59	3.80E-16
633	CADPS2	ENSG00000081803.15	0	1	0.51	0.0012	0.59	4.00E-05
633	SEH1L	ENSG00000085415.15	0.12	0.27	0.31	1.70E-04	0.59	5.40E-16
633	OGFOD1	ENSG00000087263.16	0.23	0.075	0.28	0.012	0.59	1.50E-09
633	BCL7B	ENSG00000106635.7	0.33	7.60E-05	0.24	0.0042	0.59	3.60E-16
633	SMURF2	ENSG00000108854.15	0.26	0.016	0.33	6.30E-04	0.59	6.80E-12
633	MVK	ENSG00000110921.13	0.49	5.60E-04	0.07	0.74	0.59	3.90E-06
633	GPN3	ENSG00000111231.8	0.28	0.096	0.43	0.0021	0.59	2.70E-06
633	BYSL	ENSG00000112578.9	0.19	0.14	0.33	0.0015	0.59	8.30E-11
633	ZMIZ2	ENSG00000122515.14	0.07	0.68	0.55	5.50E-08	0.59	1.00E-09
633	LRRFIP1	ENSG00000124831.18	0.44	4.10E-21	0.12	0.026	0.59	2.10E-40
633	PAICS	ENSG00000128050.8	0.21	0.0013	0.32	3.80E-08	0.59	2.10E-27
633	CEP85	ENSG00000130695.14	0.26	0.17	0.06	0.79	0.59	1.10E-05
633	PDLIM4	ENSG00000131435.12	-0.04	0.77	0.47	1.00E-10	0.59	2.40E-17
633	TUBG1	ENSG00000131462.7	0.23	0.041	0.32	0.001	0.59	1.00E-11

633	HSD17B7	ENSG00000132196.13	0.36	0.0084	0.16	0.33	0.59	3.00E-07
633	SLC43A3	ENSG00000134802.17	0.01	0.95	0.52	7.40E-07	0.59	2.70E-09
633	TMEM2	ENSG00000135048.13	0.2	0.063	0.4	6.20E-06	0.59	9.90E-13
633	PPIL1	ENSG00000137168.7	0.15	0.36	0.41	4.40E-04	0.59	1.80E-08
633	PTRH2	ENSG00000141378.14	0.25	0.052	0.33	0.0033	0.59	1.50E-09
633	PSEN2	ENSG00000143801.16	0.15	0.2	0.38	1.80E-05	0.59	2.90E-13
633	VOPP1	ENSG00000154978.12	0.1	0.16	0.3	8.00E-09	0.59	1.60E-36
633	COA7	ENSG00000162377.5	0.22	0.05	0.45	7.30E-07	0.59	4.40E-12
633	GNL3	ENSG00000163938.16	0.26	2.10E-05	0.24	1.20E-04	0.59	7.70E-28
633	CEP83	ENSG00000173588.14	0.11	0.68	0.29	0.1	0.59	3.30E-05
633	ERN1	ENSG00000178607.15	0.05	0.85	0.57	1.40E-06	0.59	1.60E-07
633	DCC	ENSG00000187323.11	0.41	0.15	0.57	0.019	0.59	0.009
633	ATG7	ENSG00000197548.12	0.07	0.55	0.48	2.60E-13	0.59	2.20E-21
633	ATAD3A	ENSG00000197785.13	0.31	7.10E-05	0.34	4.90E-06	0.59	3.20E-18
147	MEOX1	ENSG00000005102.12	1.99	0.0016	1.45	0.0093	3.88	3.50E-08
147	ANLN	ENSG00000011426.10	0.92	6.60E-06	0.99	5.00E-07	2.01	3.00E-28
147	TACC3	ENSG00000013810.18	0.77	2.50E-07	0.77	1.80E-07	1.68	7.80E-36
147	DEPDC1	ENSG00000024526.16	1.27	1.70E-10	1.59	1.00E-16	2.45	3.10E-42
147	ARNTL2	ENSG00000029153.14	1.29	3.20E-25	2.29	3.80E-82	3.5	1.00E-195
147	RAD51	ENSG00000051180.16	0.67	8.10E-04	0.88	2.30E-06	1.55	1.40E-20
147	POLQ	ENSG00000051341.13	0.73	0.013	1.3	2.40E-07	2.07	2.20E-19
147	MCM10	ENSG00000065328.16	1.16	3.10E-06	1.35	1.30E-08	2.36	3.20E-27
147	ASPM	ENSG00000066279.17	1.04	3.20E-07	0.97	1.60E-06	1.94	1.20E-25
147	TRIP13	ENSG00000071539.13	0.79	3.70E-05	0.88	1.30E-06	1.6	6.20E-22
147	HMMR	ENSG00000072571.19	0.69	0.0027	0.89	2.30E-05	1.77	3.60E-21
147	GTSE1	ENSG00000075218.18	1.03	4.90E-06	1.08	8.60E-07	2.09	3.80E-26
147	SPAG5	ENSG00000076382.16	0.93	3.00E-04	0.94	1.50E-04	1.87	4.20E-17
147	NDC80	ENSG00000080986.12	1.19	1.90E-08	1.23	3.90E-09	2.15	4.60E-29
147	COBLL1	ENSG00000082438.15	1.65	1.00E-05	1.42	1.60E-04	2.4	2.80E-12
147	RAD54L	ENSG00000085999.11	0.83	0.0015	1.29	3.70E-08	2.17	2.50E-24
147	KIF4A	ENSG00000090889.11	0.83	8.40E-06	0.82	5.60E-06	1.7	1.50E-25
147	CD200	ENSG00000091972.18	1.29	4.10E-08	0.61	0.027	1.37	5.90E-10
147	CLSPN	ENSG00000092853.13	0.69	1.10E-04	0.79	3.90E-06	1.66	1.20E-27
147	CDC45	ENSG00000093009.9	0.94	9.80E-05	1.1	1.20E-06	1.98	6.50E-22
147	CDC6	ENSG00000094804.9	1.05	3.20E-11	1.55	7.20E-25	2.22	1.60E-53
147	PLA2G3	ENSG00000100078.3	1.39	1.10E-04	1.09	0.0031	2	3.40E-10
147	CENPM	ENSG00000100162.14	0.68	0.021	0.81	0.0031	1.36	1.30E-08
147	APOL1	ENSG00000100342.20	0.6	0.014	0.96	5.90E-06	1.26	7.90E-11
147	GINS1	ENSG00000101003.9	0.86	2.60E-05	0.82	4.80E-05	1.76	2.70E-23
147	MYBL2	ENSG00000101057.15	1.13	2.70E-06	1.11	2.70E-06	2.17	1.50E-23
147	FAM83D	ENSG00000101447.14	1.09	1.40E-06	0.93	4.90E-05	2.25	7.60E-30
147	CENPI	ENSG00000102384.13	0.79	2.30E-04	0.59	0.0075	1.69	1.20E-20
147	HTR2A	ENSG00000102468.10	0.81	5.20E-05	0.88	4.40E-06	1.86	7.60E-29
147	KLF5	ENSG00000102554.13	0.59	0.0069	0.91	2.00E-06	1.84	2.10E-27
147	DHODH	ENSG00000102967.11	0.66	0.001	0.81	1.60E-05	1.08	2.60E-10
147	OIP5	ENSG00000104147.8	1.06	0.0053	0.87	0.023	1.82	1.40E-08
147	ASF1B	ENSG00000105011.8	0.79	0.014	1.28	4.70E-06	2.2	7.80E-18
147	NEIL3	ENSG00000109674.3	1.19	0.0027	1.12	0.0036	1.91	2.10E-08
147	NCAPG	ENSG00000109805.9	0.97	1.30E-04	1.02	2.80E-05	1.94	2.30E-18
147	RAD51AP1	ENSG00000111247.14	0.89	4.20E-04	1.14	1.20E-06	1.74	8.40E-16
147	ST8SIA1	ENSG00000111728.10	2.62	1.10E-116	0.84	1.40E-10	3.11	7.40E-168
147	CRYBG1	ENSG00000112297.14	1.67	5.20E-84	0.78	4.70E-17	2.66	1.20E-222
147	TTK	ENSG00000112742.9	0.82	7.50E-04	1.15	2.10E-07	2.09	4.60E-25
147	CDH6	ENSG00000113361.12	3.18	2.30E-73	0.93	3.60E-06	3.86	1.20E-109
147	CENPA	ENSG00000115163.14	1.11	0.0013	1.58	5.70E-07	2.47	2.70E-17
147	CDC20	ENSG00000117399.13	0.84	1.10E-04	0.73	6.40E-04	1.62	1.20E-17
147	NEK2	ENSG00000117650.12	0.87	0.0061	0.89	0.0033	2.07	1.50E-15
147	CENPF	ENSG00000117724.12	0.8	2.40E-05	0.68	3.10E-04	1.78	2.40E-26
147	CD3EAP	ENSG00000117877.10	0.63	2.60E-04	0.83	1.60E-07	1.34	3.10E-20
147	KIF14	ENSG00000118193.11	0.98	5.20E-05	0.99	3.10E-05	2.17	3.00E-25
147	HELLS	ENSG00000119969.14	0.64	3.10E-06	0.81	4.30E-10	1.12	1.50E-20

147	NCAPH	ENSG00000121152.9	1.09	1.20E-05	1.14	2.30E-06	2.18	3.20E-23
147	KIF18A	ENSG00000121621.6	0.72	0.0045	1	1.10E-05	1.57	3.50E-14
147	ZWINT	ENSG00000122952.16	0.72	0.0045	0.89	1.30E-04	1.65	4.40E-15
147	CENPK	ENSG00000123219.12	0.61	0.0078	0.78	1.80E-04	1.28	3.90E-12
147	HJURP	ENSG00000123485.11	1.21	1.00E-05	1.11	3.70E-05	2.12	5.10E-18
147	IL13RA2	ENSG00000123496.7	1.93	5.40E-09	1.28	2.70E-04	3.37	6.10E-29
147	DLGAP5	ENSG00000126787.12	0.93	1.60E-06	0.8	4.20E-05	1.84	2.80E-26
147	PKMYT1	ENSG00000127564.16	0.86	2.70E-05	1.45	9.50E-15	2.11	5.70E-33
147	TICAM1	ENSG00000127666.9	0.6	3.80E-10	0.62	6.50E-11	1.08	2.60E-35
147	ADM2	ENSG00000128165.8	0.75	0.017	0.79	0.008	0.77	0.0057
147	DLL4	ENSG00000128917.6	1.63	7.80E-07	1.36	4.70E-05	1.74	1.80E-08
147	PIMREG	ENSG00000129195.15	0.88	5.10E-04	0.89	2.90E-04	1.85	1.20E-17
147	SGO1	ENSG00000129810.14	1.12	0.0029	1.36	1.10E-04	2.16	1.40E-11
147	TOP2A	ENSG00000131747.14	1.16	1.60E-07	1.14	1.80E-07	2.2	3.70E-27
147	XAF1	ENSG00000132530.16	0.65	0.025	0.84	0.0016	1.01	2.60E-05
147	MICAL2	ENSG00000133816.13	0.86	9.50E-26	0.86	7.30E-26	1.67	2.30E-100
147	TTC9	ENSG00000133985.2	1.24	1.40E-06	2.72	8.10E-33	3.66	1.50E-61
147	CCNB1	ENSG00000134057.14	0.76	1.30E-04	0.63	0.0016	1.59	8.30E-20
147	IL15RA	ENSG00000134470.20	0.66	0.023	2.05	1.20E-19	2.2	7.30E-24
147	CDCA8	ENSG00000134690.10	0.95	4.60E-05	1.1	8.00E-07	2.3	2.60E-30
147	EHF	ENSG00000135373.12	1.01	0.033	1.14	0.012	3.72	1.90E-18
147	ESPL1	ENSG00000135476.11	0.96	2.70E-05	0.94	3.10E-05	1.79	2.40E-19
147	TCF19	ENSG00000137310.11	0.6	3.50E-04	0.81	1.50E-07	1.38	2.10E-22
147	SULF1	ENSG00000137573.13	2.63	8.40E-18	1.31	5.40E-05	3.61	1.30E-33
147	NUSAP1	ENSG00000137804.12	0.97	4.20E-07	0.8	3.60E-05	1.84	1.20E-26
147	KIF23	ENSG00000137807.13	0.78	1.60E-04	0.94	1.20E-06	1.72	4.10E-22
147	KNL1	ENSG00000137812.19	0.89	2.20E-05	0.94	3.60E-06	1.88	7.80E-25
147	CEP55	ENSG00000138180.15	0.92	0.0014	0.85	0.0023	1.96	8.80E-16
147	KIF20B	ENSG00000138182.14	0.61	2.50E-04	0.6	2.10E-04	1.46	9.40E-25
147	CENPE	ENSG00000138778.11	0.87	0.001	0.85	9.20E-04	1.81	8.80E-16
147	SLC7A1	ENSG00000139514.12	0.72	4.90E-12	0.88	5.70E-18	1.37	4.40E-45
147	DIAPH3	ENSG00000139734.17	0.84	6.20E-05	0.88	1.30E-05	1.48	1.20E-15
147	TICRR	ENSG00000140534.13	1.24	2.60E-05	1.15	8.10E-05	2.25	3.90E-18
147	TAF4B	ENSG00000141384.12	0.75	0.0028	1.44	1.60E-11	1.69	3.60E-17
147	PLK4	ENSG00000142731.10	0.64	0.0041	0.92	3.20E-06	1.78	1.90E-23
147	KIF2C	ENSG00000142945.12	0.88	4.10E-06	0.8	2.80E-05	1.96	1.50E-31
147	NUF2	ENSG00000143228.12	1.41	4.20E-07	1.24	8.60E-06	2.47	6.70E-23
147	DTL	ENSG00000143476.17	0.87	5.10E-04	1.32	8.00E-09	2.15	7.10E-24
147	FANCD2	ENSG00000144554.10	0.89	3.90E-06	1	9.90E-08	1.78	1.50E-25
147	SLIT2	ENSG00000145147.19	1.13	1.20E-29	1.95	3.00E-92	3.44	3.50E-303
147	SCUBE3	ENSG00000146197.8	2.09	6.80E-30	1.3	3.00E-11	4.04	1.10E-117
147	ANKRD1	ENSG00000148677.6	1.06	6.40E-04	1.56	4.90E-08	2.5	4.30E-21
147	MKI67	ENSG00000148773.13	1.13	3.70E-07	0.94	2.20E-05	2.22	2.30E-28
147	LYPD1	ENSG00000150551.10	0.89	8.80E-17	0.7	2.80E-10	1.81	4.00E-75
147	NOCT	ENSG00000151014.5	0.76	1.20E-04	0.94	5.40E-07	1.56	1.90E-20
147	ADAM8	ENSG00000151651.15	1.22	2.00E-17	0.75	1.10E-06	2.71	3.00E-96
147	CENPU	ENSG00000151725.11	1.17	6.20E-10	1.51	1.20E-16	2.1	4.70E-34
147	SPC25	ENSG00000152253.8	0.92	0.0045	0.88	0.005	2.03	1.50E-14
147	CXADR	ENSG00000154639.18	0.91	2.10E-04	1.19	1.80E-07	1.7	5.70E-16
147	SKA1	ENSG00000154839.9	1.14	1.70E-04	1.42	7.30E-07	2.47	3.40E-21
147	BUB1B	ENSG00000156970.12	1.15	2.90E-09	1.12	6.90E-09	2.18	4.10E-35
147	RECQL4	ENSG00000160957.12	0.61	1.20E-05	0.61	7.30E-06	1.06	4.50E-18
147	SPC24	ENSG00000161888.11	0.7	0.0012	0.86	1.90E-05	1.36	4.80E-14
147	VCAM1	ENSG00000162692.10	1.48	5.30E-20	1.4	4.80E-18	2.1	5.00E-41
147	PTX3	ENSG00000163661.3	0.91	6.60E-23	0.59	4.20E-10	1.2	2.30E-41
147	KIF15	ENSG00000163808.16	0.64	0.0053	0.81	1.20E-04	1.55	9.40E-17
147	MAD2L1	ENSG00000164109.13	0.69	7.30E-05	0.9	4.00E-08	1.63	9.60E-28
147	ZNF367	ENSG00000165244.6	0.98	5.60E-05	1.6	3.80E-13	2.19	5.60E-26
147	MELK	ENSG00000165304.7	0.62	1.20E-04	0.64	4.30E-05	1.5	2.20E-27
147	SKA3	ENSG00000165480.15	0.93	7.80E-04	1.1	2.40E-05	2.17	1.20E-20
147	NNMT	ENSG00000166741.7	0.93	2.70E-08	0.86	2.90E-07	1.79	1.90E-34

147	PBK	ENSG00000168078.9	0.93	3.50E-05	1.03	2.00E-06	2.06	4.10E-26
147	MFSD2A	ENSG00000168389.17	0.78	0.013	1.49	2.30E-08	2.01	4.00E-16
147	FEN1	ENSG00000168496.3	0.69	1.30E-04	1.1	2.20E-11	1.8	3.20E-31
147	HSPB3	ENSG00000169271.2	1.56	1.00E-04	0.97	0.023	3.01	7.50E-17
147	CKAP2L	ENSG00000169607.12	1.05	1.40E-05	1.14	1.30E-06	2.15	4.20E-24
147	BUB1	ENSG00000169679.14	0.8	2.60E-04	0.73	7.80E-04	1.86	2.70E-23
147	CDK1	ENSG00000170312.15	0.97	6.50E-04	1.2	6.90E-06	2.15	1.00E-18
147	JUNB	ENSG00000171223.5	0.99	2.90E-09	0.8	1.70E-06	1.24	2.70E-15
147	SHCBP1	ENSG00000171241.8	1.09	1.50E-07	1.2	3.00E-09	2.13	4.30E-30
147	ESCO2	ENSG00000171320.14	1.04	0.0071	1.59	4.70E-06	2.44	3.10E-14
147	RRM2	ENSG00000171848.14	0.82	4.10E-04	0.96	1.10E-05	1.96	6.30E-23
147	EXO1	ENSG00000174371.16	1.12	2.20E-06	1.57	1.10E-12	2.22	2.10E-26
147	UBE2C	ENSG00000175063.16	1.31	9.00E-08	1.19	9.90E-07	2.25	7.90E-24
147	CCNE2	ENSG00000175305.17	0.88	0.0043	1.29	2.90E-06	2	3.10E-15
147	SPHK1	ENSG00000176170.13	1.14	1.10E-25	0.8	1.60E-12	2.07	3.70E-89
147	ATAD5	ENSG00000176208.8	0.82	0.0026	1.11	8.30E-06	1.9	2.20E-17
147	HASPIN	ENSG00000177602.5	0.66	0.043	0.85	0.0035	1.78	2.90E-13
147	CDH4	ENSG00000179242.15	0.67	1.30E-06	0.62	8.50E-06	0.79	8.20E-10
147	APOBEC3B	ENSG00000179750.15	0.61	0.0097	0.64	0.0037	1.62	7.50E-18
147	NLRP10	ENSG00000182261.3	2.83	3.50E-06	2.28	6.20E-05	3.91	2.90E-10
147	NOG	ENSG00000183691.4	1.22	0.0026	0.92	0.027	1.94	2.70E-08
147	IQGAP3	ENSG00000183856.10	0.95	1.50E-05	0.81	2.10E-04	1.86	1.10E-21
147	CDCA2	ENSG00000184661.13	1.04	1.10E-06	0.76	6.70E-04	1.99	1.20E-25
147	MYBL1	ENSG00000185697.16	1.12	7.10E-08	3.51	4.40E-80	4.4	2.70E-127
147	KIF18B	ENSG00000186185.13	1.17	1.10E-06	1.24	1.50E-07	2.46	3.00E-31
147	KIF24	ENSG00000186638.16	0.6	0.046	0.64	0.022	1.14	1.40E-06
147	ERCC6L	ENSG00000186871.6	1.31	4.70E-05	1.73	9.40E-09	2.65	6.20E-21
147	FAM111B	ENSG00000189057.10	0.96	0.0014	1.66	4.30E-10	2.46	1.20E-22
147	SH2D5	ENSG00000189410.11	0.78	0.017	1.32	2.30E-06	2.12	8.60E-17
147	ACSL5	ENSG00000197142.10	0.69	0.024	0.96	4.10E-04	1.69	5.10E-13
147	PDCD1LG2	ENSG00000197646.7	1.23	9.40E-13	0.98	4.00E-08	2.24	4.70E-46
147	ITPRIPL1	ENSG00000198885.9	0.7	0.0016	0.74	4.80E-04	0.85	1.50E-05
147	PRC1	ENSG00000198901.13	0.93	2.00E-06	0.85	1.10E-05	1.9	3.40E-27
147	AP001476	ENSG00000224413.1	1.63	4.90E-06	1.01	0.0081	1.9	8.30E-09
147	KIFC1	ENSG00000237649.7	1.26	5.90E-08	1.28	2.00E-08	2.41	4.60E-30
147	INMT	ENSG00000241644.2	0.85	1.90E-06	0.62	7.20E-04	0.86	2.30E-07
147	AC114284	ENSG00000248927.1	0.76	0.0016	0.59	0.016	1.25	7.60E-10
147	AC124798	ENSG00000260196.1	0.79	0.019	0.69	0.038	1.32	1.50E-06
147	UHRF1	ENSG00000276043.4	0.65	3.00E-06	0.9	7.00E-12	1.52	3.40E-34
148	HS3ST1	ENSG00000002587.9	3.64	7.40E-40	-1.27	0.0033	4.12	2.50E-52
148	CCL26	ENSG00000006606.8	6.63	7.70E-15	0.15	0.8	7.12	2.30E-17
148	ANOS1	ENSG00000011201.11	2.99	1.10E-05	0.51	0.22	4.6	9.40E-10
148	MAMLD1	ENSG00000013619.13	0.63	2.10E-08	0.35	0.0044	1	2.30E-22
148	GCLM	ENSG00000023909.9	0.7	2.40E-11	0.27	0.03	0.72	6.80E-13
148	CAMK2B	ENSG00000058404.19	3.33	2.00E-38	0.62	0.083	3.61	5.70E-46
148	NTN1	ENSG00000065320.8	0.82	0.0011	0.51	0.059	0.89	7.00E-05
148	LMCD1	ENSG00000071282.11	0.74	2.30E-15	-0.26	0.018	1.02	1.30E-30
148	PLXNA2	ENSG00000076356.6	0.67	1.50E-12	0.01	0.96	0.6	1.10E-10
148	JADE1	ENSG00000077684.15	1.05	5.00E-61	0.17	0.036	1.28	3.40E-93
148	EDN1	ENSG00000078401.6	1.23	1.60E-04	-0.1	0.85	1.13	2.20E-04
148	RIMS1	ENSG00000079841.18	1.82	1.10E-06	-0.29	0.61	2.33	1.00E-11
148	TPX2	ENSG00000088325.15	0.75	2.40E-04	0.56	0.0071	1.58	3.20E-19
148	BIRC5	ENSG00000089685.14	0.65	0.003	0.48	0.031	1.44	6.20E-15
148	DSP	ENSG00000096696.13	1.67	1.60E-18	0.13	0.71	2.07	2.60E-30
148	KCNK6	ENSG00000099337.4	0.77	6.70E-14	-0.07	0.66	0.77	1.70E-14
148	CDKN3	ENSG00000100526.19	0.65	0.016	0.37	0.21	1.36	5.10E-10
148	SNPH	ENSG00000101298.13	0.59	5.30E-13	0.05	0.69	0.88	5.50E-31
148	LIPG	ENSG00000101670.11	1.06	6.90E-11	0.24	0.25	0.85	9.20E-08
148	MXRA5	ENSG00000101825.7	1.28	2.50E-27	-0.53	5.10E-05	0.59	1.60E-06
148	NDFIP2	ENSG00000102471.13	0.67	4.20E-19	0.18	0.052	0.96	3.50E-41
148	SLC7A5	ENSG00000103257.8	0.77	1.10E-07	0.49	0.0012	0.96	2.20E-12

148	SFRP1	ENSG00000104332.11	0.88	8.90E-23	0.1	0.42	0.91	4.80E-25
148	ADAP1	ENSG00000105963.13	0.82	0.014	-0.23	0.61	0.61	0.05
148	MEST	ENSG00000106484.14	0.91	1.70E-36	0.14	0.12	1.15	2.90E-59
148	TSPAN13	ENSG00000106537.7	1.26	2.70E-35	-0.26	0.055	1.03	5.20E-24
148	PITRM1	ENSG00000107959.15	0.66	7.00E-42	0.07	0.27	0.82	2.20E-66
148	RASD1	ENSG00000108551.4	1.56	3.00E-08	0.13	0.77	1.86	2.30E-12
148	CCDC86	ENSG00000110104.11	1.57	4.50E-91	0.38	1.50E-05	2.06	1.40E-160
148	KIAA1549L	ENSG00000110427.14	0.8	0.0043	0.28	0.42	1.42	1.50E-09
148	KRT18	ENSG00000111057.10	0.93	2.10E-17	0.22	0.11	1.37	7.10E-39
148	GLI1	ENSG00000111087.9	0.83	1.10E-07	0.21	0.3	0.77	2.90E-07
148	FOXM1	ENSG00000111206.12	0.63	6.30E-06	0.19	0.27	1.1	2.70E-18
148	GSG1	ENSG00000111305.18	1.05	0.0032	0.19	0.7	1.14	3.70E-04
148	VDR	ENSG00000111424.10	1.14	1.60E-51	0.47	1.60E-08	1.48	1.70E-89
148	HMGCS1	ENSG00000112972.14	0.65	1.50E-10	0.08	0.6	0.74	1.50E-14
148	KIF20A	ENSG00000112984.11	0.77	0.0015	0.36	0.19	1.3	6.50E-10
148	RASGRF2	ENSG00000113319.12	0.77	2.40E-25	0.4	9.70E-07	1.01	6.10E-45
148	CNTN3	ENSG00000113805.8	1.01	6.30E-12	0.5	0.0023	0.81	1.90E-08
148	COL7A1	ENSG00000114270.17	0.97	2.90E-07	0.21	0.38	0.68	2.70E-04
148	CISH	ENSG00000114737.15	2.66	1.80E-98	0	1	2.74	6.80E-106
148	EVA1A	ENSG00000115363.13	0.64	3.60E-05	0.5	0.0018	1.21	2.60E-19
148	IL1RL1	ENSG00000115602.16	2.34	2.40E-10	0.54	0.27	3.47	1.20E-23
148	IL18R1	ENSG00000115604.10	0.88	3.40E-09	0.56	3.80E-04	1.35	7.40E-23
148	AGMAT	ENSG00000116771.5	0.83	0.0053	0.52	0.11	1.07	3.30E-05
148	SIPA1L2	ENSG00000116991.10	1.04	9.20E-10	-0.48	0.012	0.66	1.10E-04
148	RGS4	ENSG00000117152.13	1.12	6.80E-16	0.28	0.11	2.22	1.40E-65
148	MYB	ENSG00000118513.18	1.24	0.04	0.42	0.47	2.52	1.50E-05
148	PKD2	ENSG00000118762.7	0.94	1.40E-62	0.02	0.83	1.11	4.10E-89
148	ABCG2	ENSG00000118777.10	0.84	3.30E-04	0.24	0.42	1.34	3.00E-11
148	SPP1	ENSG00000118785.13	3.5	3.30E-14	0.9	0.072	3.66	3.80E-16
148	DUSP1	ENSG00000120129.5	1.13	5.50E-90	0.1	0.2	1.05	4.80E-79
148	ACAT2	ENSG00000120437.8	0.63	5.90E-05	0.11	0.61	0.8	3.00E-08
148	EGR1	ENSG00000120738.7	1.76	1.30E-20	0.11	0.72	1.96	3.80E-26
148	BATF3	ENSG00000123685.8	1.08	8.00E-07	-0.11	0.75	1.42	1.00E-12
148	RUNX2	ENSG00000124813.20	0.59	6.80E-11	0.51	1.90E-08	1.06	5.30E-37
148	KLF2	ENSG00000127528.5	2	3.70E-62	-0.05	0.82	2.01	4.10E-63
148	CPA4	ENSG00000128510.10	1.67	7.00E-19	0.54	0.013	2.45	8.70E-42
148	CRACR2A	ENSG00000130038.9	0.6	0.018	0.35	0.2	0.88	3.50E-05
148	LDLR	ENSG00000130164.13	0.74	1.50E-20	0.21	0.027	0.74	9.80E-22
148	PTPRE	ENSG00000132334.16	0.78	2.30E-05	0.4	0.053	1.29	1.40E-15
148	TRPC4	ENSG00000133107.14	0.76	0.0056	0.13	0.74	1.39	5.20E-10
148	POSTN	ENSG00000133110.14	0.81	7.50E-37	0.56	3.30E-17	1.18	9.00E-79
148	TMTC1	ENSG00000133687.15	0.73	2.40E-21	0.42	1.80E-07	1.18	1.20E-56
148	PSAT1	ENSG00000135069.13	0.69	9.10E-06	0.23	0.21	0.81	1.30E-08
148	TROAP	ENSG00000135451.12	0.69	0.025	0.55	0.074	1.53	4.10E-10
148	PKIB	ENSG00000135549.14	0.6	5.00E-08	0.07	0.68	0.9	7.70E-19
148	HTR2B	ENSG00000135914.5	1.41	5.00E-14	-0.26	0.33	1.34	2.30E-13
148	ARMC9	ENSG00000135931.17	1.01	1.30E-81	-0.15	0.03	0.88	9.90E-63
148	SPRY2	ENSG00000136158.11	0.72	1.90E-10	0.26	0.045	1.27	1.40E-33
148	RNF144B	ENSG00000137393.9	0.93	0.037	-0.02	0.98	1.22	0.0011
148	MYOF	ENSG00000138119.16	0.73	7.60E-59	0	0.99	0.84	2.60E-78
148	SHROOM3	ENSG00000138771.14	0.63	3.00E-10	0.06	0.68	0.74	6.10E-15
148	SLC39A8	ENSG00000138821.12	0.98	0.0011	0.48	0.17	1.53	2.00E-09
148	INHBE	ENSG00000139269.2	2.28	8.90E-17	0.54	0.14	2.63	2.90E-23
148	DUSP6	ENSG00000139318.7	0.7	3.10E-10	0.18	0.2	0.68	1.80E-10
148	CBLN2	ENSG00000141668.9	1.56	4.80E-04	0.59	0.27	2.61	2.70E-11
148	COL6A1	ENSG00000142156.14	0.69	1.30E-20	0.57	1.70E-14	0.94	5.40E-39
148	CYR61	ENSG00000142871.16	0.85	4.80E-07	0.47	0.0083	1.18	3.60E-14
148	CSRNP1	ENSG00000144655.14	0.96	1.60E-11	0.35	0.037	1.45	1.20E-27
148	MYH15	ENSG00000144821.9	0.88	0.019	0.64	0.096	1.27	5.30E-05
148	UCN2	ENSG00000145040.3	1.02	3.10E-08	0.42	0.056	1.06	1.40E-09
148	DDIT4L	ENSG00000145358.6	0.89	0.013	0.24	0.6	0.76	0.02

148	PAM	ENSG00000145730.20	0.73	2.10E-54	-0.03	0.74	0.66	2.70E-45
148	TNFRSF21	ENSG00000146072.6	1.02	4.90E-47	0.16	0.087	0.64	7.50E-19
148	EBP	ENSG00000147155.10	0.59	3.00E-08	0.11	0.44	0.66	3.50E-11
148	TRIM55	ENSG00000147573.16	0.84	3.30E-05	0.47	0.034	1.38	1.40E-14
148	ENDOD1	ENSG00000149218.4	0.78	1.10E-15	0.02	0.89	0.64	2.50E-11
148	KIRREL3	ENSG00000149571.11	0.92	1.40E-10	0.07	0.76	1.25	7.00E-21
148	ADAMTS12	ENSG00000151388.10	0.63	1.00E-21	0.06	0.54	0.64	1.20E-23
148	FRMD4A	ENSG00000151474.21	0.71	7.20E-33	0.17	0.013	1	3.10E-67
148	HK1	ENSG00000156515.21	1.15	1.40E-150	0.16	0.0025	1.18	7.30E-161
148	TMEM171	ENSG00000157111.12	1.64	1.70E-38	0.36	0.027	2.08	2.20E-64
148	LRP8	ENSG00000157193.15	0.74	1.40E-06	0.56	4.30E-04	1.09	4.20E-15
148	CDC25C	ENSG00000158402.18	0.74	0.03	0.48	0.18	1.65	7.70E-10
148	SLC45A3	ENSG00000158715.5	0.91	1.50E-12	-0.05	0.84	0.88	1.60E-12
148	IER2	ENSG00000160888.6	0.91	3.50E-68	0.11	0.12	1	2.20E-82
148	RACGAP1	ENSG00000161800.12	0.66	4.50E-05	0.49	0.0032	1.35	1.10E-21
148	EIF4A1	ENSG00000161960.14	0.6	1.30E-04	0.12	0.57	0.79	1.10E-08
148	CAPN2	ENSG00000162909.17	0.62	1.30E-90	0.12	4.00E-04	0.74	4.00E-128
148	CITED2	ENSG00000164442.9	0.87	3.70E-23	0.17	0.13	1.18	2.50E-43
148	NFIL3	ENSG00000165030.3	0.81	2.60E-33	-0.08	0.44	0.99	1.10E-50
148	JCAD	ENSG00000165757.8	0.79	4.60E-18	0.24	0.025	0.88	3.00E-23
148	PPFIBP2	ENSG00000166387.11	0.59	8.60E-04	-0.02	0.94	1.26	1.10E-17
148	SERPINB7	ENSG00000166396.12	0.9	0.0079	0.2	0.66	1.23	2.00E-05
148	PLK1	ENSG00000166851.14	0.78	2.50E-05	0.47	0.019	1.62	3.70E-23
148	MVD	ENSG00000167508.11	0.65	9.10E-06	0.07	0.75	0.71	2.20E-07
148	SPINT2	ENSG00000167642.12	2.11	1.20E-46	-0.15	0.55	2.84	5.70E-88
148	TK1	ENSG00000167900.11	0.61	3.50E-05	0.25	0.14	1.05	6.60E-16
148	PCSK9	ENSG00000169174.10	1.02	2.20E-06	0.19	0.52	1.3	4.30E-11
148	PCDH7	ENSG00000169851.15	0.66	1.70E-22	0.27	2.90E-04	0.75	3.10E-30
148	TCAF2	ENSG00000170379.19	0.71	0.015	0.05	0.91	1	4.40E-05
148	CDH2	ENSG00000170558.8	0.81	1.90E-66	-0.55	2.40E-27	0.73	3.90E-55
148	LRRC8D	ENSG00000171492.14	1.56	3.60E-75	0.48	3.80E-07	1.83	7.40E-105
148	ISG20	ENSG00000172183.14	0.71	0.0039	-0.31	0.29	0.71	0.0014
148	DHCR7	ENSG00000172893.15	0.6	1.80E-10	0.12	0.32	0.67	7.40E-14
148	SH3RF3	ENSG00000172985.10	0.6	2.00E-20	0.38	2.70E-08	0.69	3.50E-28
148	RGMB	ENSG00000174136.11	1.14	2.40E-48	0.54	9.20E-11	1.74	3.40E-114
148	IL20RB	ENSG00000174564.12	1.68	1.20E-06	0.52	0.24	1.29	1.20E-04
148	SOX11	ENSG00000176887.6	0.83	6.30E-38	0.16	0.056	1.31	1.20E-100
148	IL17RA	ENSG00000177663.13	0.84	5.50E-27	0.18	0.062	1.27	1.90E-64
148	OXTR	ENSG00000180914.10	0.66	3.60E-05	0.28	0.13	1.1	3.90E-15
148	GPR3	ENSG00000181773.6	0.79	0.026	0.46	0.22	1.27	1.20E-05
148	PTGDR2	ENSG00000183134.4	2.32	1.60E-101	-0.65	3.00E-07	1.77	4.60E-59
148	CLDN5	ENSG00000184113.9	1.06	0.015	-0.04	0.96	0.85	0.036
148	PRR16	ENSG00000184838.14	0.8	6.40E-28	0.39	1.20E-06	1.18	7.60E-63
148	FLRT2	ENSG00000185070.10	0.84	3.90E-16	0.22	0.087	0.6	4.70E-09
148	SAPCD2	ENSG00000186193.8	0.89	8.50E-04	0.45	0.13	1.28	3.70E-08
148	INSIG1	ENSG00000186480.12	0.82	2.50E-17	0.27	0.016	1.34	5.50E-47
148	TUBB4B	ENSG00000188229.5	0.61	7.20E-11	0.36	2.00E-04	0.94	5.10E-27
148	CLDN4	ENSG00000189143.9	0.81	8.80E-08	0.39	0.021	1.4	3.10E-24
148	HRH1	ENSG00000196639.6	0.89	1.20E-31	0.48	4.90E-09	0.99	1.90E-40
148	LRRC8B	ENSG00000197147.13	1.08	2.40E-20	0.53	5.70E-05	1.91	7.40E-68
148	APCDD1L	ENSG00000198768.10	0.93	1.30E-21	0.27	0.019	1	8.20E-26
148	ARHGAP11	ENSG00000198826.10	0.6	2.00E-04	0.55	4.60E-04	1.36	5.30E-23
148	RORB	ENSG00000198963.10	1.45	3.70E-23	-0.49	0.0065	1.23	2.90E-17
148	MIR221	ENSG00000207870.1	0.86	0.017	0.32	0.47	1.42	1.60E-06
148	APCDD1L-A	ENSG00000231290.5	0.84	2.20E-13	0.02	0.93	0.84	3.10E-14
148	AC002480.	ENSG00000232759.1	1.17	0.027	0.39	0.53	1.7	2.10E-04
148	AKAP2	ENSG00000241978.9	0.63	0.018	0.55	0.035	0.93	2.80E-05
148	CCDC71L	ENSG00000253276.2	0.7	1.70E-18	0.28	0.0018	0.89	4.90E-31
148	LINC01583	ENSG00000259518.1	1.45	1.30E-04	0	1	1.62	2.70E-06
148	SH3RF3-AS	ENSG00000259863.1	0.68	0.0027	0.45	0.062	1.03	6.70E-08
148	ANXA8L1	ENSG00000264230.7	1.11	0.018	0.36	0.53	1.13	0.007

148	AL606834.	ENSG00000269906.1	0.9	0.039	0.28	0.59	0.78	0.047
148	AL158152.	ENSG00000269929.1	0.63	0.021	0.01	0.98	0.78	7.80E-04
148	PRAG1	ENSG00000275342.4	0.59	1.60E-09	0.04	0.81	0.61	1.10E-10
148	DACH1	ENSG00000276644.4	1.13	4.10E-21	-0.36	0.013	0.7	7.30E-09

Table S4. Pathway analysis for 633 unique transcripts upregulated by LIGHT with IL-13 in human pulmonary fibroblasts, associated with Fig. 2B-C.

Category	Term	Count	%	PValue	Genes	List Total	Pop Hits	Pop Total	Fold Enrichment	Bonferroni	Benjamini	FDR
UP_KW_BIOLOGICAL_PROCESS	KW-0235~DNA replication	24	4.18118	8.57E-15	RFC5, CDT1, PCNA, LIG1, MCM7, MCM8, RFC2, DSCC1, PRIM1, GINS4, TWNK, CHTF18, SAMHD1, POLD3, POLA1, CHAF1B, DBF4, CHAF1A, ORC1, POLD1, GRWD1, MCM4, MCM6, POLE	350	96	11376	8.12571429	9.75E-13	9.77E-13	8.92E-13
UP_KW_BIOLOGICAL_PROCESS	KW-0131~Cell cycle	55	9.58188	1.30E-10	SUV39H2, MCM7, ZWILCH, MCM8, SUV39H1, DSCC1, DDX12P, NCAPG2, GMNN, KIF11, CHTF18, CKS1B, CHAF1B, CHAF1A, PTTG1, RUVBL1, FBXO5, SPDL1, LZTS1, BORA, LIG1, CKAP2, HAUS6, VRK1, KNSTRN, CIT, CCNA2, SGO2, DBF4, CCNE1, INCENP, DMC1, CKS2, TIMELESS, MCM4, MCM6, SEH1L, CDCA3, CDCA4, LIN9, AURKB, AURKA, CCNB2, DSN1, RBBP8, E2F3, FANCI, CDT1, CDKN2C, CENPW, GADD45A, CYLD, UBE2S, NCAPD3, RAN	350	689	11376	2.5945677	1.48E-08	7.39E-09	6.74E-09
UP_KW_PTM	KW-0007~Acetylation	167	29.0941	4.30E-10	OTUD4, RAB3B, JPT1, TLNRD1, NUP188, CSE1L, JPT2, GMNN, PPAN, GPATCH4, ENO1, FGF1, ETS1, RRP9, DOCK10, PTTG1, MYC, PPAT, RUVBL1, TRIM25, KIF21A, KPNA2, MAP2K3, ACOT7, LIG1, METTL1, KRT7, AFAP1, LMO7, ATAD3A, GTPBP4, MTHFD1, MTHFD2, PPA1, DKC1, HMBS, PPIF, SRFBP1, S100A3, EZR, RAI14, NUP205, DNMT1, RNASEH2A, SHMT1, NOP2, NOLC1, FBXO45, MRPL12, HACD2, ACACA, CORO1C, BRIP1, LDHA, ORC1, FIGNL1, EIF4EBP1, BRIX1, ATG7, SLC19A1, FDPS, STIL, EGR2, PNPT1, NOP16, ALYREF, XRCC3, FANCB, PYCR3, PAICS, PUM3, SSH1, EHD1, MFHAS1, POLA1, PPFIBP1, EHD4, UBE2S, STK26, FASN, REL, CYCS, CNOT9, RAD18, NXT1, MCM7, SUV39H1, HMGB2, KIF11, SIPA1L3, LMNB2, NRGN, CKS1B, TUBA1C, CHAF1B, TUBA1B, ZNF280B, NUP62, RAC3, ME2, SPDL1, LRRFIP1, COA7, RRP15, YWHAH, RFC5, UTP15, EIF5A, ANXA2, PARP1, PARP2, RFC2, RRAS2, RANGAP1, CIT, GNL3, CCNA2, PSMA3, TUBB2A, PKM, DDAH1, TBC1D4, CKS2, MCM4, PGAM5, SNRPA1, L2HGDH, MCM6, HAGH, MTFR2, DOCK2, EEF1E1, SLC25A4, THOP1, DTYMK, PCNA, PRIM1, MAK16, LIN9, SAMHD1, PTGS2, AURKB, SRM, POLD3, BID, SEC23B, CCT5, CAP2, NOP56, TIGAR, MYO10, MID1IP1, GINS4, PLCL2, LYSMD2, SCLT1, MND1, HNRNPAB, MZT1, TARBP1, MYO1D, FABP3, ACTC1, POLE3, NAA15, RAN, PFKP	441	3500	14201	1.53648656	1.20E-08	1.25E-08	1.20E-08
KEGG_PATHWAY	hsa03030:DNA replication	14	2.43902	4.62E-11	RFC5, RNASEH2A, PCNA, LIG1, MCM7, RFC2, PRIM1, POLD3, POLA1, POLD1, POLE3, MCM4, MCM6, POLE	283	36	8586	11.7985866	1.29E-08	1.29E-08	1.27E-08
GOTERM_BP_DIRECT	GO:0006260~DNA replication	22	3.83275	9.97E-12	RFC5, RNASEH2A, PCNA, LIG1, MCM7, RFC2, DSCC1, TWNK, CHTF18, SAMHD1, POLD3, POLA1, CHAF1B, DBF4, CHAF1A, ORC1, POLD1, GRWD1, POLE3, MCM4, MCM6, POLE	520	122	19462	6.74911728	2.67E-08	2.67E-08	2.65E-08
UP_KW_PTM	KW-0597~Phosphoprotein	311	54.1812	3.48E-07	MYC, ZC3H12A, HABP4, TRIM25, KIF21A, FBXO5, EPHB1, IER3, TANGO6, METTL1, SFMBT1, KRT7, AFAP1, KNSTRN, GTPBP4, DEPDC1B, MTHFD1, PPA1, PDE12, GRWD1, HMBS, ORAI1, KCNQ5, ALPK3, SRFBP1, TRIM16, CCDC81, EZR, PRR11, ATF3, EPHA2, NUP205, TMEM87B, HACD1, SEH1L, CDCA3, CSTF2, NOLC1, SLC5A3, BRIP1, DSN1, LDHA, FIGNL1, HIVEP1, HIVEP2, KCNN4, ATG7, LYAR, PAG1, SLC19A1, LYN, CDT1, GADD45A, MCAM, DCLK2, PAICS, EHD1, CYLD, POLA1, PPFIBP1, PEX5L, EHD4, FASN, TDP1, REL, CEP83, CEP85, RAD18, EZH2, SUV39H2, ROBO4, SYNM, SUV39H1, PAK1IP1, NCAPG2, KIF11, SIPA1L3, PFAS, WDR43, BBC3, TUBA1C, CHAF1B, TUBA1B, CHAF1A, NUDCD1, ZNF280B, RRP15, LIN52, ZNF365, PARP1, MME, ANXA2, KCND3, PARP2, NFAM1, RRAS2, KIF22, PPRC1, TUBG1, UCHL3, ZDHHC14, GNL3, CIT, PSMA3, SLC7A6, TUBB2A, MMP13, INCENP, PGAM5, SGK3, MGLL, FOXC2, PCNA, PKN3, SLC43A3, ODC1, AKAP5, CST4, PSMC3IP, EXOSC5, NSD2, USP1, HEATR3, GPAT3, BID, CCT5, CAP2, MYEF2, UTP4, C1GALT1, GINS4, RFLNB, BYSL, TARBP1, FABP3, TTLL4, RFWD3, HYLS1, CENPN, NCAPD3, RAB3B, OTUD4, NUP188, ZWILCH, CSE1L, GMNN, TNC, FGF1, ETS1, DOCK10, TUBB6, RGS3, PTTG1, KPNA2, MB21D2, SLC16A3, LZTS1, SYBU, MAP2K3, CADPS2, GAS2L3, BORA, LIG1, ZGRF1, PUS1, TOMM34, VRK1, LMO7, TEX2, ATAD3A, SLC9B2, ERN1, SGO2, DKC1, CCNE1, MRTO4, TIMELESS, RAI14, COL17A1, DNMT1, RNASEH2A, ARHGEF26, PSMD14, NOP2, PSEN2, FBXO43, ACACA, ARHGAP22, ITPRIPL2, EFNB2, CCNB2, IRAK1, ORC1, PODXL, EIF4EBP1, BRIX1, UTP20, STAP2, RHPN2, FANCI, ANKRD34A, STIL, PNPT1, MLKL, NOP16, ATP8B1, ALYREF, SYT9, SSH1, GGCT, YRDC, IL6, UBE2S, STK26, SRPRB, CYCS, AMOTL2, NFE2L1, NFKBIB, CIITA, MCM7,	441	8392	14201	1.19337037	9.74E-06	5.04E-06	4.87E-06
GOTERM_BP_DIRECT	GO:0051301~cell division	33	5.74913	1.90E-08	SEH1L, CDCA3, ZWILCH, CDCA4, NCAPG2, KIF11, AURKB, AURKA, CKS1B, CCNB2, TUBA1C, TUBA1B, DSN1, PTTG1, RUVBL1, RBBP8, FBXO5, SPDL1, CDT1, CENPW, BORA, LIG1, HAUS6, VRK1, KNSTRN, CCNA2, SGO2, CCNE1, UBE2S, CKS2, TIMELESS, NCAPD3, RAN	520	389	19462	3.17503461	5.08E-05	2.54E-05	2.53E-05
UP_KW_BIOLOGICAL_PROCESS	KW-0132~Cell division	33	5.74913	2.01E-06	SEH1L, CDCA3, ZWILCH, CDCA4, NCAPG2, KIF11, AURKB, AURKA, CKS1B, CCNB2, DSN1, PTTG1, RUVBL1, RBBP8, FBXO5, SPDL1, CDT1, CENPW, BORA, LIG1, HAUS6, VRK1, KNSTRN, CIT, CCNA2, SGO2, CCNE1, UBE2S, INCENP, CKS2, TIMELESS, NCAPD3, RAN	350	421	11376	2.5477299	2.29E-04	7.64E-05	6.97E-05
UP_KW_BIOLOGICAL_PROCESS	KW-0498~Mitosis	26	4.52962	3.11E-06	SEH1L, CDCA3, ZWILCH, NCAPG2, KIF11, AURKB, AURKA, CCNB2, DSN1, PTTG1, RUVBL1, RBBP8, FBXO5, SPDL1, CDT1, CENPW, BORA, HAUS6, VRK1, KNSTRN, CIT, CCNA2, INCENP, TIMELESS, NCAPD3, RAN	350	290	11376	2.91404926	3.55E-04	8.87E-05	8.09E-05
UP_KW_PTM	KW-0832~Ubl conjugation	120	20.9059	9.40E-06	DCLRE1B, GPATCH4, ENO1, ETS1, RRP9, RGS3, PTTG1, SNRPD1, MYC, ZMIZ2, DPF1, ZC3H12A, RUVBL1, HABP4, TRIM25, FBXO5, EPHB1, TOMM34, KRT7, VRK1, LMO7, GTPBP4, TRAIP, MTHFD2, CCNE1, DKC1, TRIM14, ORAI1, SRFBP1, ZNF710, TRIM16, PRR11, ATF3, EPHA2, DNMT1, SEH1L, CDCA3, CSTF2, NOP2, NOLC1, FBXO43, LDHA, DSN1, IRAK1, FIGNL1, EIF4EBP1, BRIX1, LYAR, FANCI, LYN, CDT1, STIL, EGR2, NOP16, SMURF2, AZIN1, CYLD, MFHAS1, PPFIBP1, UBE2S, FASN, MXD1, RAD18, NFE2L1, EZH2, SUV39H2, MCM7, SUV39H1, RASGRF1, KIF11, LMNB2, WDR43, TUBA1B, CHAF1A, BCL7A, ZNF280B, RAC3, SPDL1, LRRFIP1, RRP15, KLF10, UTP15, ANXA2, PARP1, DCC, KIF22, RANGAP1, GNL3, CCNA2, TUBB2A, PKM, PTRH2, MCM4, SNRPA1, MET, USP13, KRT81, FOXC2, PCNA, MAK16, LIN9, SAMHD1, AURKB, AURKA, POLD3, SERTAD1, POLD1, RBBP8, USP1, BID, CCT5, NOP56, MYEF2, UTP4, HNRNPAB, SNRNP40, ERCC2, ABCE1, ASB2, RAN	441	2662	14201	1.45162197	2.63E-04	9.09E-05	8.78E-05

UP_KW_BIOLOGICAL_PROCESS	KW-0234~DNA repair	29	5.05226	5.24E-06	DCLRE1B, PCNA, PSMD14, MCM8, TONSL, SAMHD1, POLD3, CHAF1B, BRIP1, CHAF1A, PTTG1, POLD1, RUVBL1, USP1, RBBP8, POLE, FANCI, LIG1, PARP1, PARP2, XRCC3, FANCB, TRAIP, MMS22L, TDP1, RFWD3, ERCC2, TIMELESS, RAD18	350	358	11376	2.63291301	5.97E-04	1.19E-04	1.09E-04
UP_KW_BIOLOGICAL_PROCESS	KW-0227~DNA damage	32	5.57491	8.08E-06	DCLRE1B, PCNA, PSMD14, MCM8, TONSL, SAMHD1, POLD3, CHAF1B, BRIP1, CHAF1A, PTTG1, POLD1, ZC3H12A, RUVBL1, USP1, RBBP8, POLE, FANCI, LIG1, PARP1, GADD45A, PARP2, XRCC3, AEN, FANCB, TRAIP, MMS22L, TDP1, RFWD3, ERCC2, TIMELESS, RAD18	350	429	11376	2.42445554	9.21E-04	1.54E-04	1.40E-04
UP_KW_BIOLOGICAL_PROCESS	KW-0690~Ribosome biogenesis	14	2.43902	9.74E-06	NOP56, UTP15, UTP4, PAK1IP1, NIP7, NOP2, GTF3A, GTPBP4, WDR43, BYSL, DKC1, MRTO4, HEATR3, BRIX1	350	99	11376	4.59636364	0.00111012	1.59E-04	1.45E-04
KEGG_PATHWAY	hsa03410:Base excision repair	11	1.91638	1.15E-06	RFC5, POLD3, PCNA, LIG1, PARP1, PARP2, RFC2, POLD1, TDP1, POLE3, POLE	283	44	8586	7.58480565	3.22E-04	1.61E-04	1.57E-04
UP_SEQ_FEATURE	CROSSLNK:Glycyl lysine isopeptide (Lys-Gly) (interchain with G-Cter in SUMO2)	65	11.324	2.32E-07	DCLRE1B, MCM7, GPATCH4, KIF11, ETS1, LMNB2, RRP9, BCL7A, SNRPD1, MYC, ZMIZ2, ZNF280B, DPF1, RUVBL1, LRRFIP1, RRP15, UTP15, PARP1, TOMM34, KRT7, VRK1, LMO7, KIF22, RANGAP1, GTPBP4, TRAIP, GNL3, PKM, DKC1, MCM4, SRFBP1, SNRPA1, ZNF710, ATF3, USP13, FOXC2, DNMT1, SEH1L, PCNA, MAK16, CSTF2, NOP2, NOLC1, LIN9, SAMHD1, AURKA, POLD3, DSN1, POLD1, FIGNL1, RBBP8, BRIX1, LYAR, CCT5, NOP56, MYEF2, NOP16, UTP4, HNRNPAB, PPFIBP1, SNRNP40, FASN, RAN, RAD18, EZH2	531	1275	20583	1.97613825	5.43E-04	5.43E-04	5.43E-04
KEGG_PATHWAY	hsa04110:Cell cycle	18	3.13589	1.44E-05	CDT1, CDKN2C, PCNA, MCM7, GADD45A, AURKB, CCNA2, CCNB2, DBF4, ORC1, PTTG1, CCNE1, MYC, MCM4, E2F3, MCM6, FBXO5, YWHAH	283	157	8586	3.47838221	0.00403623	0.00134812	0.00132
UP_KW_PTM	KW-1017~Isopeptide bond	85	14.8084	1.96E-04	DCLRE1B, MCM7, GPATCH4, ENO1, KIF11, ETS1, LMNB2, WDR43, RRP9, TUBA1B, TUBB6, CHAF1A, BCL7A, SNRPD1, MYC, ZMIZ2, ZNF280B, DPF1, RUVBL1, HABP4, TRIM25, RAC3, LRRFIP1, RRP15, UTP15, PARP1, ANXA2, TOMM34, KRT7, VRK1, LMO7, KIF22, RANGAP1, GTPBP4, TRAIP, GNL3, PKM, TUBB2A, MTHFD2, DKC1, PTRH2, MCM4, SRFBP1, SNRPA1, ZNF710, ATF3, USP13, KRT81, FOXC2, DNMT1, SEH1L, PCNA, MAK16, CSTF2, NOP2, NOLC1, LIN9, SAMHD1, AURKA, POLD3, LDHA, DSN1, IRAK1, POLD1, FIGNL1, RBBP8, EIF4EBP1, BRIX1, LYAR, CCT5, NOP56, FANCI, MYEF2, NOP16, UTP4, SMURF2, HNRNPAB, PPFIBP1, SNRNP40, ACTC1, FASN, ABCE1, RAN, RAD18, EZH2	441	1858	14201	1.47317233	0.00547976	0.00142263	0.00137
GOTERM_BP_DIRECT	GO:0000278~mitotic cell cycle	17	2.96167	4.08E-06	CDT1, CENPW, NOLC1, KIF11, KIF22, TUBG1, AURKB, AURKA, CIT, TUBA1C, TUBA1B, TUBB6, TUBB2A, INCENP, NUDT15, POLE, RAN	520	155	19462	4.10488834	0.01087481	0.00364478	0.00362
GOTERM_BP_DIRECT	GO:0006281~DNA repair	24	4.18118	6.97E-06	RFC5, FANCI, LIG1, PARP1, GADD45A, PARP2, RFC2, XRCC3, KIF22, SAMHD1, POLA1, CHAF1B, BRIP1, CHAF1A, PTTG1, POLD1, TDP1, DMC1, RUVBL1, USP1, RBBP8, TIMELESS, POLE, RAD18	520	302	19462	2.97432501	0.01849043	0.00466586	0.00464
BIOCARTA	h_mcmPathway:CDK Regulation of DNA Replication	7	1.21951	5.41E-05	CDT1, KITLG, MCM7, ORC1, CCNE1, MCM4, MCM6	69	18	1623	9.147343	0.00652748	0.0065487	0.00649
GOTERM_BP_DIRECT	GO:1900264~positive regulation of DNA-directed DNA polymerase activity	6	1.0453	1.43E-05	RFC5, PCNA, RFC2, DSCC1, GINS4, CHTF18	520	13	19462	17.2739645	0.03746983	0.00763792	0.00759
GOTERM_BP_DIRECT	GO:0006364~rRNA processing	15	2.61324	1.98E-05	NOP56, UTP15, SUV39H1, PPAN, NOLC1, WDR43, BYSL, RRP9, EXOSC5, DKC1, MRTO4, BRIX1, UTP20, LYAR, RRP15	520	138	19462	4.06814381	0.05154267	0.00881966	0.00876
GOTERM_BP_DIRECT	GO:0006272~leading strand elongation	4	0.69686	7.39E-05	POLA1, PCNA, POLE3, POLE	520	4	19462	37.4269231	0.17962616	0.02529098	0.02513
GOTERM_BP_DIRECT	GO:0006270~DNA replication initiation	7	1.21951	7.99E-05	POLA1, MCM7, ORC1, CCNE1, PRIM1, MCM4, MCM6	520	28	19462	9.35673077	0.19253556	0.02529098	0.02513
GOTERM_BP_DIRECT	GO:0000731~DNA synthesis involved in DNA repair	6	1.0453	8.50E-05	RFC5, POLD3, POLA1, RFC2, POLD1, POLE	520	18	19462	12.475641	0.20357992	0.02529098	0.02513
UP_KW_BIOLOGICAL_PROCESS	KW-0620~Polyamine biosynthesis	4	0.69686	0.0021	AMD1, ODC1, AZIN1, SRM	350	9	11376	14.4457143	0.21267367	0.02814897	0.02568
UP_KW_BIOLOGICAL_PROCESS	KW-0444~Lipid biosynthesis	14	2.43902	0.00222	AGPAT5, FDPS, HACD1, PTGIS, MVK, MID1IP1, DHCR24, PTGS2, HSD17B7, HACD2, ACACA, FASN, GPAT3, MGLL	350	170	11376	2.67670588	0.22401544	0.02814897	0.02568
UP_KW_BIOLOGICAL_PROCESS	KW-0159~Chromosome partition	7	1.21951	0.00265	SGO2, DSN1, SEH1L, PTTG1, INCENP, ERCC2, CEP85	350	46	11376	4.94608696	0.26139303	0.03025871	0.0276
UP_KW_BIOLOGICAL_PROCESS	KW-0698~rRNA processing	10	1.74216	0.0038	UTP15, EXOSC5, UTP4, DKC1, SUV39H1, NOP2, UTP20, LYAR, WDR43, RRP9	350	101	11376	3.21810467	0.35182758	0.03934318	0.03589
KEGG_PATHWAY	hsa04218:Cellular senescence	15	2.61324	5.96E-04	MAP2K3, GADD45A, PIK3CD, RRAS2, LIN9, ETS1, CCNA2, CCNB2, IL6, CCNE1, MYC, EIF4EBP1, E2F3, SLC25A4, LIN52	283	156	8586	2.91723294	0.15383546	0.04174791	0.04085
KEGG_PATHWAY	hsa03430:Mismatch repair	6	1.0453	7.63E-04	RFC5, POLD3, PCNA, LIG1, RFC2, POLD1	283	23	8586	7.91457981	0.19238994	0.04271889	0.0418
GOTERM_BP_DIRECT	GO:0006974~cellular response to DNA damage stimulus	21	3.65854	1.57E-04	LYN, TIGAR, PARP1, MCM7, MCM8, PARP2, SUV39H1, XRCC3, FBXO45, VRK1, SAMHD1, BBC3, TRAIP, MYC, RFWD3, ZC3H12A, POLE3, TIMELESS, FBXO5, RAD18, IER3	520	298	19462	2.63746773	0.34385278	0.04213369	0.04187
GOTERM_BP_DIRECT	GO:0032508~DNA duplex unwinding	9	1.56794	1.83E-04	RFC5, BRIP1, MCM8, RFC2, DSCC1, ERCC2, DDX12P, RUVBL1, CHTF18	520	60	19462	5.61403846	0.38793423	0.04462459	0.04434
BIOCARTA	h_ranMSPathway:Role of Ran in mitotic spindle regulation	5	0.87108	7.46E-04	RANGAP1, KPNA2, RAN, SNHG3, AURKA	69	11	1623	10.6916996	0.08628848	0.04510337	0.04473
KEGG_PATHWAY	hsa03420:Nucleotide excision repair	9	1.56794	9.89E-04	RFC5, POLD3, PCNA, LIG1, RFC2, POLD1, ERCC2, POLE3, POLE	283	63	8586	4.33417466	0.24198118	0.04615167	0.04516
UP_KW_BIOLOGICAL_PROCESS	KW-0275~Fatty acid biosynthesis	7	1.21951	0.00544	HACD1, PTGIS, FASN, PTGS2, HACD2, ACACA, MGLL	350	53	11376	4.29283019	0.46298731	0.0516701	0.04714
UP_SEQ_FEATURE	CROSSLNK:Glycyl lysine isopeptide (Lys-Gly) (interchain with G-Cter in SUMO2); alternate	24	4.18118	4.07E-05	DNMT1, PCNA, PARP1, ANXA2, NOLC1, ENO1, RANGAP1, ETS1, GTPBP4, LMNB2, WDR43, POLD3, LDHA, PKM, CHAF1A, DKC1, MTHFD2, MYC, RUVBL1, RBBP8, HABP4, SNRPA1, RRP15, RAN	531	349	20583	2.66563062	0.09089105	0.0476442	0.04764
GOTERM_BP_DIRECT	GO:0006275~regulation of DNA replication	8	1.39373	2.30E-04	CCNA2, PCNA, DSCC1, POLE3, GMNN, RUVBL1, ID3, FBXO5	520	47	19462	6.3705401	0.45962229	0.05127758	0.05095
GOTERM_BP_DIRECT	GO:0042273~ribosomal large subunit biogenesis	7	1.21951	2.49E-04	NOP16, PAK1IP1, NIP7, MRTO4, GTF3A, HEATR3, GTPBP4	520	34	19462	7.70554299	0.48659566	0.05127758	0.05095
UP_KW_BIOLOGICAL_PROCESS	KW-0658~Purine biosynthesis	4	0.69686	0.00651	MTHFD1, PPAT, PFAS, PAICS	350	13	11376	10.0008791	0.52515115	0.05710241	0.05209

BIOCARTA	h_npcPathway:Mechanism of Protein Import into the Nucleus	5	0.87108	0.00152	NUP62, RANGAP1, KPNA2, RAN, SNHG3	69	13	1623	9.04682274	0.16764866	0.06112052	0.06062
UP_KW_PTM	KW-0702~S-nitrosylation	7	1.21951	0.01128	DDAH1, FASN, ODC1, PPIF, EZR, PTGS2, SLC25A4	441	61	14201	3.69529014	0.27207063	0.06540688	0.06315
GOTERM_BP_DIRECT	GO:0006261~DNA-dependent DNA replication	7	1.21951	3.45E-04	RFC5, POLD3, RFC2, POLD1, GINS4, POLE3, POLE	520	36	19462	7.27745727	0.60268094	0.06591833	0.0655
GOTERM_BP_DIRECT	GO:0030174~regulation of DNA-dependent DNA replication initiation	5	0.87108	4.04E-04	CDT1, MCM7, GMNN, MCM4, MCM6	520	14	19462	13.3667582	0.66154767	0.0722102	0.07175
GOTERM_BP_DIRECT	GO:0044772~mitotic cell cycle phase transition	6	1.0453	4.52E-04	CCNA2, CCNB2, CCNE1, CKS2, LZTS1, CKS1B	520	25	19462	8.98246154	0.70179476	0.07229981	0.07184
GOTERM_BP_DIRECT	GO:0000070~mitotic sister chromatid segregation	7	1.21951	4.67E-04	SGO2, DSN1, INCENP, NCAPG2, TUBG1, KNSTRN, RAN	520	38	19462	6.8944332	0.71368611	0.07229981	0.07184
GOTERM_BP_DIRECT	GO:0000082~G1/S transition of mitotic cell cycle	9	1.56794	4.86E-04	CCNA2, DBF4, CCNE1, MYC, EIF4EBP1, RBBP8, E2F3, POLE, EZH2	520	69	19462	4.88177258	0.72793462	0.07229981	0.07184
GOTERM_BP_DIRECT	GO:0008284~positive regulation of cell proliferation	29	5.05226	7.70E-04	CNTF, BNC1, ODC1, NOP2, TNC, NOLC1, FGF1, PTHLH, FGF5, EFNB2, BCL7A, SERTAD1, MYC, NTF3, BCL7B, GCNT2, RAC3, E2F3, FBXO5, LYN, PRLR, GREM1, KITLG, IL6, FAM98A, FXN, SLC25A33, ATF3, EZH2	520	545	19462	1.99152435	0.87282408	0.10849422	0.10781
GOTERM_BP_DIRECT	GO:0007052~mitotic spindle organization	8	1.39373	8.54E-04	FAM110A, STIL, INCENP, KIF11, TUBG1, AURKB, RAN, AURKA	520	58	19462	5.16233422	0.89851119	0.11434149	0.11362
GOTERM_BP_DIRECT	GO:0006271~DNA strand elongation involved in DNA replication	4	0.69686	9.55E-04	POLD3, POLA1, MCM7, MCM4	520	8	19462	18.7134615	0.92267391	0.12183338	0.12106
KEGG_PATHWAY	hsa05222:Small cell lung cancer	10	1.74216	0.00313	CCNE1, GADD45A, MYC, CKS2, PIK3CD, CYCS, ITGA6, E2F3, PTGS2, CKS1B	283	92	8586	3.29774159	0.58420943	0.1251714	0.12249
GOTERM_BP_DIRECT	GO:0008283~cell proliferation	14	2.43902	0.00111	USP13, MCM7, PAK1IP1, RASGRF1, ODC1, AURKB, BYSL, CKS1B, GNL3, MYC, OGFOD1, ERCC2, CKS2, POLR3G	520	180	19462	2.91098291	0.94955126	0.13568784	0.13483
UP_KW_BIOLOGICAL_PROCESS	KW-0554~One-carbon metabolism	4	0.69686	0.02216	DHFR, MTHFD1, MTHFD2, SHMT1	350	20	11376	6.50057143	0.92224037	0.18040951	0.16458
KEGG_PATHWAY	hsa04210:Apoptosis	12	2.09059	0.00506	ERN1, TUBA1C, TUBA1B, PARP1, PARP2, GADD45A, PIK3CD, CYCS, NGF, BID, LMNB2, BBC3	283	136	8586	2.67699023	0.75839845	0.17710855	0.17331
GOTERM_BP_DIRECT	GO:0071168~protein localization to chromatin	5	0.87108	0.00172	MMS22L, PARP1, MCM8, TONSL, EZH2	520	20	19462	9.35673077	0.99016174	0.20076049	0.19949
KEGG_PATHWAY	hsa05169:Epstein-Barr virus infection	15	2.61324	0.00666	LYN, MAP2K3, PSMD14, GADD45A, TAP2, PIK3CD, CCNA2, IL6, IRAK1, CCNE1, MYC, CYCS, E2F3, BID, NFKBIB	283	202	8586	2.25291257	0.84620311	0.20731975	0.20288
KEGG_PATHWAY	hsa05230:Central carbon metabolism in cancer	8	1.39373	0.00784	LDHA, TIGAR, PKM, MYC, PIK3CD, SLC16A3, MET, PFKP	283	70	8586	3.46733973	0.88948329	0.2193948	0.21469
GOTERM_BP_DIRECT	GO:0071897~DNA biosynthetic process	6	1.0453	0.00194	POLD3, LIG1, LOX, POLD1, POLE3, LIN9	520	34	19462	6.60475113	0.99451869	0.21672309	0.21535
KEGG_PATHWAY	hsa03013:Nucleocytoplasmic transport	10	1.74216	0.00895	NUP205, NXT1, SEH1L, NUP188, CSE1L, ALYREF, NUP62, RANGAP1, KPNA2, RAN	283	108	8586	2.80918728	0.91936166	0.22786317	0.22298
GOTERM_BP_DIRECT	GO:0007049~cell cycle	21	3.65854	0.00235	FANCI, SUV39H2, CDT1, CDKN2C, BORA, CDCA3, MCM7, MCM8, SUV39H1, LIN9, KIF11, CHTF18, AURKA, CKS1B, CYLD, CHAF1B, CHAF1A, RUVBL1, MCM4, MCM6, LZTS1	520	370	19462	2.12423077	0.99818439	0.24984838	0.24826
GOTERM_BP_DIRECT	GO:0006268~DNA unwinding involved in DNA replication	5	0.87108	0.0025	MCM7, GINS4, TWNK, MCM4, MCM6	520	22	19462	8.50611888	0.99875922	0.24984838	0.24826
GOTERM_BP_DIRECT	GO:0097421~liver regeneration	6	1.0453	0.00252	IL6, PCNA, PNPT1, MYC, EZH2, AURKA	520	36	19462	6.23782051	0.99883428	0.24984838	0.24826
GOTERM_BP_DIRECT	GO:0006913~nucleocytoplasmic transport	7	1.21951	0.00277	NUP205, NXT1, SEH1L, NUP188, NUP62, RANGAP1, RAN	520	53	19462	4.94317852	0.99941155	0.26527516	0.26359
BIOCARTA	h_btg2Pathway:BTG family proteins and cell cycle regulation	4	0.69686	0.00912	CHAF1B, CHAF1A, NGF, FGF1	69	11	1623	8.55335968	0.66990305	0.275827	0.27355
GOTERM_BP_DIRECT	GO:0000079~regulation of cyclin-dependent protein serine/threonine kinase activity	7	1.21951	0.00305	CCNA2, CCNB2, CDKN2C, SERTAD1, CCNE1, GADD45A, GTPBP4	520	54	19462	4.85163818	0.99972096	0.27737254	0.27561
GOTERM_BP_DIRECT	GO:0071456~cellular response to hypoxia	11	1.91638	0.00319	CCNA2, SUV39H2, BRIP1, TIGAR, PTGIS, IRAK1, MYC, SUV39H1, CLCA1, PTGS2, BBC3	520	134	19462	3.07235936	0.99980691	0.27737254	0.27561
GOTERM_BP_DIRECT	GO:0031297~replication fork processing	6	1.0453	0.00321	MMS22L, PCNA, RFWD3, ALYREF, TONSL, TRAIP	520	38	19462	5.90951417	0.99981816	0.27737254	0.27561
GOTERM_BP_DIRECT	GO:0000727~double-strand break repair via break-induced replication	4	0.69686	0.00347	MCM7, GINS4, MCM4, MCM6	520	12	19462	12.475641	0.99990838	0.2900533	0.28821
KEGG_PATHWAY	hsa01523:Antifolate resistance	5	0.87108	0.01593	DHFR, IL6, SHMT1, GGH, SLC19A1	283	30	8586	5.0565371	0.98885006	0.32886952	0.32182
KEGG_PATHWAY	hsa04360:Axon guidance	13	2.26481	0.01668	SEMA7A, WNT5B, DCC, LRRC4, PIK3CD, SSH1, EFNB2, RGS3, PLXNA1, RAC3, MET, EPHB1, EPHA2	283	182	8586	2.16708733	0.99099849	0.32886952	0.32182
KEGG_PATHWAY	hsa05132:Salmonella infection	16	2.78746	0.01711	MAP2K3, ARHGEF26, MLKL, ANXA2, CSE1L, PIK3CD, TUBA1C, TUBA1B, TUBB6, IL6, TUBB2A, IRAK1, PODXL, MYC, CYCS, RILP	283	249	8586	1.94950828	0.99202693	0.32886952	0.32182
KEGG_PATHWAY	hsa05161:Hepatitis B	12	2.09059	0.01762	MAP2K3, CCNA2, IL6, EGR2, PCNA, IRAK1, CCNE1, MYC, PIK3CD, CYCS, E2F3, BID	283	162	8586	2.24734982	0.99310539	0.32886952	0.32182
GOTERM_BP_DIRECT	GO:0007057~spindle assembly involved in female meiosis I	3	0.52265	0.00411	CCNB2, FBXO5, AURKA	520	4	19462	28.0701923	0.99998376	0.32541283	0.32335
GOTERM_BP_DIRECT	GO:0007059~chromosome segregation	9	1.56794	0.00413	CDT1, SGO2, DSN1, CENPW, INCENP, ERCC2, CENPN, CEP85, KNSTRN	520	96	19462	3.50877404	0.99998469	0.32541283	0.32335

GOTERM_BP_DIRECT	GO:0030335~positive regulation of cell migration	16	2.78746	0.00557	LYN, SEMA7A, WNT5B, MCAM, ARHGEF39, PIK3CD, RRAS2, FGF1, CCL7, PODXL, NTF3, PECAM1, GCNT2, ITGA6, DRD1, EPHA2	520	267	19462	2.24281187	0.99999968	0.41446732	0.41184
GOTERM_BP_DIRECT	GO:0000226~microtubule cytoskeleton organization	11	1.91638	0.00557	TUBA1C, GAS2L3, TUBA1B, TUBB6, TUBB2A, TTLL4, FIGNL1, DCLK2, HAUS6, TUBG1, KNSTRN	520	145	19462	2.83928382	0.99999968	0.41446732	0.41184
KEGG_PATHWAY	hsa00670:One carbon pool by folate	4	0.69686	0.02642	DHFR, MTHFD1, MTHFD2, SHMT1	283	20	8586	6.06784452	0.99944563	0.44668413	0.43711
KEGG_PATHWAY	hsa04151:PI3K-Akt signaling pathway	20	3.48432	0.02712	PKN3, TNC, PIK3CD, PPP2R3A, NGF, FGF1, PRLR, FGF5, IL6, KITLG, CCNE1, MYC, COL6A2, NTF3, EIF4EBP1, ITGA6, SGK3, MET, YWHAH, EPHA2	283	359	8586	1.69020739	0.99954649	0.44668413	0.43711
GOTERM_BP_DIRECT	GO:0006297~nucleotide-excision repair, DNA gap filling	3	0.52265	0.00673	POLD3, POLD1, POLE	520	5	19462	22.4561539	0.99999999	0.46380285	0.46086
GOTERM_BP_DIRECT	GO:0006595~polyamine metabolic process	3	0.52265	0.00673	AMD1, ODC1, SRM	520	5	19462	22.4561539	0.99999999	0.46380285	0.46086
GOTERM_BP_DIRECT	GO:0006287~base-excision repair, gap-filling	4	0.69686	0.00675	PCNA, LIG1, POLD1, POLE	520	15	19462	9.98051282	0.99999999	0.46380285	0.46086
KEGG_PATHWAY	hsa05166:Human T-cell leukemia virus 1 infection	14	2.43902	0.03113	FDPS, EGR2, CDKN2C, PIK3CD, ETS1, CCNA2, CCNB2, IL6, PTTG1, CCNE1, MYC, E2F3, SLC25A4, RAN	283	222	8586	1.91328431	0.99985735	0.48425153	0.47387
KEGG_PATHWAY	hsa04115:p53 signaling pathway	7	1.21951	0.03422	CCNB2, CCNE1, AIFM2, GADD45A, CYCS, BID, BBC3	283	74	8586	2.86992646	0.99994171	0.50432693	0.49352
GOTERM_BP_DIRECT	GO:0006606~protein import into nucleus	9	1.56794	0.00747	NXT1, NUP188, CSE1L, NUP62, HEATR3, E2F3, DRD1, KPNA2, RAN	520	106	19462	3.17775762	1	0.50019196	0.49702
GOTERM_BP_DIRECT	GO:0016579~protein deubiquitination	10	1.74216	0.00814	OTUD4, USP13, CYLD, PSMA3, PSMD14, USP31, SHMT1, ZC3H12A, USP1, UCHL3	520	130	19462	2.87899408	1	0.50760314	0.50438
GOTERM_BP_DIRECT	GO:0046655~folic acid metabolic process	4	0.69686	0.00815	DHFR, MTHFD2, SHMT1, SLC19A1	520	16	19462	9.35673077	1	0.50760314	0.50438
GOTERM_BP_DIRECT	GO:0070266~necroptotic process	4	0.69686	0.00815	CYLD, MLKL, PPIF, PGAM5	520	16	19462	9.35673077	1	0.50760314	0.50438
GOTERM_BP_DIRECT	GO:0007346~regulation of mitotic cell cycle	7	1.21951	0.00883	CYLD, MYC, GMNN, CKS2, TTLL12, FBXO5, CKS1B	520	67	19462	3.91027555	1	0.53746152	0.53405
KEGG_PATHWAY	hsa04010:MAPK signaling pathway	17	2.96167	0.03926	MAP2K3, DUSP5, DUSP2, GADD45A, RASGRF1, RRAS2, NGF, FGF1, DUSP7, FGF5, KITLG, IRAK1, MYC, NTF3, RAC3, MET, EPHA2	283	301	8586	1.71351091	0.99998652	0.54963568	0.53786
BIOCARTA	h_ranPathway:Cycling of Ran in nucleocytoplasmic transport	3	0.52265	0.02327	RANGAP1, RAN, SNHG3	69	6	1623	11.7608696	0.94206615	0.56303748	0.55838
GOTERM_BP_DIRECT	GO:0006189~'de novo' IMP biosynthetic process	3	0.52265	0.00992	PPAT, PFAS, PAICS	520	6	19462	18.7134615	1	0.56494867	0.56136
GOTERM_BP_DIRECT	GO:0007100~mitotic centrosome separation	3	0.52265	0.00992	NUP62, KIF11, AURKA	520	6	19462	18.7134615	1	0.56494867	0.56136
GOTERM_BP_DIRECT	GO:0009113~purine nucleobase biosynthetic process	3	0.52265	0.00992	SHMT1, PPAT, PAICS	520	6	19462	18.7134615	1	0.56494867	0.56136
KEGG_PATHWAY	hsa03440:Homologous recombination	5	0.87108	0.04455	POLD3, BRIP1, POLD1, XRCC3, RBBP8	283	41	8586	3.6999052	0.99999713	0.58162764	0.56916
KEGG_PATHWAY	hsa05017:Spinocerebellar ataxia	10	1.74216	0.0457	NOP56, ERN1, PSMA3, PSMD14, KCND3, TWNK, PPIF, PIK3CD, CYCS, SLC25A4	283	143	8586	2.12162396	0.99999795	0.58162764	0.56916
GOTERM_BP_DIRECT	GO:0043388~positive regulation of DNA binding	5	0.87108	0.01109	MYC, ERCC2, PLAUR, HMGB2, NGF	520	33	19462	5.67074592	1	0.61860674	0.61468
GOTERM_BP_DIRECT	GO:0008637~apoptotic mitochondrial changes	4	0.69686	0.01142	AIFM2, PPIF, BID, SLC25A4	520	18	19462	8.31709402	1	0.62398155	0.62002
GOTERM_BP_DIRECT	GO:0034605~cellular response to heat	6	1.0453	0.01227	LYN, IRAK1, STAC, FGF1, PTGS2, IER5	520	52	19462	4.31849112	1	0.65739795	0.65322
GOTERM_BP_DIRECT	GO:0007051~spindle organization	4	0.69686	0.01329	KIF11, KNSTRN, AURKB, AURKA	520	19	19462	7.87935223	1	0.69804949	0.69362
GOTERM_BP_DIRECT	GO:0097294~'de novo' XMP biosynthetic process	3	0.52265	0.01364	PPAT, PFAS, PAICS	520	7	19462	16.0401099	1	0.70232862	0.69787
GOTERM_BP_DIRECT	GO:0042274~ribosomal small subunit biogenesis	7	1.21951	0.01404	NOP56, LTV1, UTP15, UTP4, UTP20, WDR43, RRP9	520	74	19462	3.54038462	1	0.70945898	0.70496
GOTERM_BP_DIRECT	GO:2000045~regulation of G1/S transition of mitotic cell cycle	6	1.0453	0.01431	CDKN2C, BCL7A, DPF1, BCL7B, BID, TMEM14B	520	54	19462	4.15854701	1	0.70945898	0.70496
GOTERM_BP_DIRECT	GO:0030282~bone mineralization	6	1.0453	0.0154	KLF10, MMP13, LOX, ERCC2, PTGS2, PTHLH	520	55	19462	4.08293706	1	0.74990686	0.74515
GOTERM_BP_DIRECT	GO:0044208~'de novo' AMP biosynthetic process	3	0.52265	0.01786	PPAT, PFAS, PAICS	520	8	19462	14.0350962	1	0.85432054	0.8489
UP_SEQ_FEATURE	DOMAIN:MCM	4	0.69686	0.00127	MCM7, MCM8, MCM4, MCM6	531	9	20583	17.2278719	0.94895362	0.85784302	0.85784
UP_SEQ_FEATURE	MOTIF:Nuclear localization signal	24	4.18118	0.00179	DUSP5, DNMT1, PARP1, BNC1, PARP2, NOLC1, AEN, FGF1, PUM3, PTHLH, LMNB2, RRP9, BRIP1, OGFOD1, POLD1, NFKBIZ, ERCC2, REL, UTP20, SGK3, HIVEP2, MXD1, KPNA2, RAI14	531	456	20583	2.04014273	0.98478336	0.85784302	0.85784
UP_SEQ_FEATURE	DOMAIN:DNA-directed DNA polymerase family B exonuclease	3	0.52265	0.00195	POLA1, POLD1, POLE	531	3	20583	38.7627119	0.98967185	0.85784302	0.85784
UP_SEQ_FEATURE	DOMAIN:Tubulin/FtsZ 2-layer sandwich	5	0.87108	0.0022	TUBA1C, TUBA1B, TUBB6, TUBB2A, TUBG1	531	22	20583	8.80970724	0.99421638	0.85784302	0.85784

GOTERM_BP_DIRECT	GO:0000724~double-strand break repair via homologous recombination	8	1.39373	0.01846	MMS22L, PSMD14, MCM8, RFWD3, XRCC3, TONSL, RBBP8, SAMHD1	520	101	19462	2.96450876	1	0.8673453	0.86184
GOTERM_BP_DIRECT	GO:1905168~positive regulation of double-strand break repair via homologous recombination	5	0.87108	0.01968	PARP1, RUVBL1, TIMELESS, RBBP8, FANCB	520	39	19462	4.79832347	1	0.89089436	0.88524
GOTERM_BP_DIRECT	GO:0036297~interstrand cross-link repair	5	0.87108	0.01968	DCLRE1B, FANCI, RFWD3, XRCC3, FANCB	520	39	19462	4.79832347	1	0.89089436	0.88524
GOTERM_BP_DIRECT	GO:0002244~hematopoietic progenitor cell differentiation	7	1.21951	0.01996	LYN, AGPAT5, KITLG, GPATCH4, INHBA, SIPA1L3, HERC6	520	80	19462	3.27485577	1	0.89089436	0.88524
UP_SEQ_FEATURE	DOMAIN:Tubulin/FtsZ GTPase	5	0.87108	0.00358	TUBA1C, TUBA1B, TUBB6, TUBB2A, TUBG1	531	25	20583	7.75254237	0.99977279	0.8980903	0.89809
UP_SEQ_FEATURE	DOMAIN:DNA-directed DNA polymerase family B multifunctional	3	0.52265	0.00384	POLA1, POLD1, POLE	531	4	20583	29.0720339	0.99987637	0.8980903	0.89809
UP_SEQ_FEATURE	ZN_FING:CysA-type	3	0.52265	0.00384	POLA1, POLD1, POLE	531	4	20583	29.0720339	0.99987637	0.8980903	0.89809
UP_SEQ_FEATURE	MOTIF:CysB motif	3	0.52265	0.00384	POLA1, POLD1, POLE	531	4	20583	29.0720339	0.99987637	0.8980903	0.89809
GOTERM_BP_DIRECT	GO:0001649~osteoblast differentiation	9	1.56794	0.02146	SEMA7A, LOX, FASN, FIGNL1, ALYREF, TNC, RRAS2, GTPBP4, EPHA2	520	128	19462	2.63158053	1	0.91570063	0.90989
GOTERM_BP_DIRECT	GO:0090630~activation of GTPase activity	9	1.56794	0.02237	ARHGAP22, ARHGEF26, TBC1D4, RASGRF1, NTF3, RANGAP1, SIPA1L3, EPHA2, CORO1C	520	129	19462	2.61118068	1	0.91570063	0.90989
GOTERM_BP_DIRECT	GO:1901214~regulation of neuron death	4	0.69686	0.02246	DCC, RRAS2, DHCR24, EPHB1	520	23	19462	6.5090301	1	0.91570063	0.90989
GOTERM_BP_DIRECT	GO:0000027~ribosomal large subunit assembly	4	0.69686	0.02246	MRTO4, NOP2, PPAN, BRIX1	520	23	19462	6.5090301	1	0.91570063	0.90989
GOTERM_BP_DIRECT	GO:0006596~polyamine biosynthetic process	3	0.52265	0.02257	ODC1, AZIN1, SRM	520	9	19462	12.475641	1	0.91570063	0.90989
GOTERM_BP_DIRECT	GO:0006177~GMP biosynthetic process	3	0.52265	0.02257	PPAT, PFAS, PAICS	520	9	19462	12.475641	1	0.91570063	0.90989
GOTERM_BP_DIRECT	GO:0034644~cellular response to UV	6	1.0453	0.02316	PCNA, PARP1, POLD1, MYC, PTGS2, AURKB	520	61	19462	3.6813367	1	0.92559343	0.91972
GOTERM_BP_DIRECT	GO:0046653~tetrahydrofolate metabolic process	3	0.52265	0.02772	DHFR, MTHFD2, SHMT1	520	10	19462	11.2280769	1	1	0.99402
GOTERM_BP_DIRECT	GO:0045943~positive regulation of transcription from RNA polymerase I promoter	4	0.69686	0.02806	UTP15, BNC1, LYAR, WDR43	520	25	19462	5.98830769	1	1	0.99402
GOTERM_BP_DIRECT	GO:0043069~negative regulation of programmed cell death	4	0.69686	0.02806	TIGAR, NUP62, AMIGO2, TM7SF3	520	25	19462	5.98830769	1	1	0.99402
GOTERM_BP_DIRECT	GO:0042908~xenobiotic transport	4	0.69686	0.02806	SLC22A4, SLC43A3, ABCA3, NR1I2	520	25	19462	5.98830769	1	1	0.99402
GOTERM_BP_DIRECT	GO:0030334~regulation of cell migration	8	1.39373	0.03305	DOCK10, FGF5, ROBO4, TNC, PLXNA1, AMOTL2, SGK3, FGF1	520	114	19462	2.62645074	1	1	0.99402
GOTERM_BP_DIRECT	GO:0006390~transcription from mitochondrial promoter	3	0.52265	0.03329	TWNK, MRPL12, SLC25A33	520	11	19462	10.2073427	1	1	0.99402
GOTERM_BP_DIRECT	GO:0035999~tetrahydrofolate interconversion	3	0.52265	0.03329	MTHFD1, MTHFD2, SHMT1	520	11	19462	10.2073427	1	1	0.99402
GOTERM_BP_DIRECT	GO:0090200~positive regulation of release of cytochrome c from mitochondria	4	0.69686	0.03432	PLAUR, PPIF, BID, BBC3	520	27	19462	5.54472935	1	1	0.99402
GOTERM_BP_DIRECT	GO:0000281~mitotic cytokinesis	6	1.0453	0.03495	INCENP, CKAP2, EXOC6, AURKB, CIT, ZNF365	520	68	19462	3.30237557	1	1	0.99402
GOTERM_BP_DIRECT	GO:0006096~glycolytic process	5	0.87108	0.03618	LDHA, TIGAR, PKM, ENO1, IER3	520	47	19462	3.98158756	1	1	0.99402
GOTERM_BP_DIRECT	GO:0070301~cellular response to hydrogen peroxide	6	1.0453	0.03688	ERN1, IL6, PCNA, PPIF, FXN, EZH2	520	69	19462	3.25451505	1	1	0.99402
GOTERM_BP_DIRECT	GO:0034501~protein localization to kinetochore	3	0.52265	0.03925	ZWILCH, SPDL1, AURKB	520	12	19462	9.35673077	1	1	0.99402
GOTERM_BP_DIRECT	GO:0090267~positive regulation of mitotic cell cycle spindle assembly checkpoint	3	0.52265	0.03925	INCENP, XRCC3, AURKB	520	12	19462	9.35673077	1	1	0.99402
GOTERM_BP_DIRECT	GO:0000462~maturation of SSU-rRNA from tricistronic rRNA transcript (SSU-rRNA, 5.8S rRNA, LSU-rRNA)	4	0.69686	0.04124	UTP4, ERCC2, WDR43, BYSL	520	29	19462	5.16233422	1	1	0.99402
GOTERM_BP_DIRECT	GO:0007422~peripheral nervous system development	4	0.69686	0.04124	EGR2, NTF3, NGF, SLC5A3	520	29	19462	5.16233422	1	1	0.99402
GOTERM_BP_DIRECT	GO:0007129~synapsis	4	0.69686	0.04495	PSMC3IP, CCNE1, DMC1, MND1	520	30	19462	4.99025641	1	1	0.99402

GOTERM_BP_DIRECT	GO:0006511~ubiquitin-dependent protein catabolic process	14	2.43902	0.04812	USP13, PSMD14, USP31, SMURF2, RNF19B, FBXO45, UCHL3, CYLD, PSMA3, UBE2S, USP1, TRIM25, ASB2, HERC6	520	290	19462	1.80681698	1	1	0.99402
GOTERM_BP_DIRECT	GO:0070536~protein K63-linked deubiquitination	4	0.69686	0.04881	OTUD4, USP13, CYLD, PSMD14	520	31	19462	4.8292804	1	1	0.99402
GOTERM_BP_DIRECT	GO:0006633~fatty acid biosynthetic process	5	0.87108	0.04957	HACD1, FASN, HACD2, ACACA, MGLL	520	52	19462	3.5987426	1	1	0.99402
GOTERM_BP_DIRECT	GO:0006302~double-strand break repair	6	1.0453	0.04988	BRIP1, PARP1, ZGRF1, PARP2, TDP1, NSD2	520	75	19462	2.99415385	1	1	0.99402
UP_SEQ_FEATURE	MOTIF:Arginine finger	3	0.52265	0.00629	MCM7, MCM4, MCM6	531	5	20583	23.2576271	0.99999961	1	1
UP_SEQ_FEATURE	DOMAIN:AAA+ ATPase	6	1.0453	0.01246	RFC5, RFC2, FIGNL1, RUVBL1, CHTF18, ATAD3A	531	54	20583	4.30696799	1	1	1
UP_SEQ_FEATURE	COMPBIAS:Basic and acidic residues	169	29.4425	0.01899	DCLRE1B, OTUD4, LTV1, POP1, JPT2, GMNN, PPAN, TONSL, XYLT1, GPATCH4, RRP9, HS6ST3, DOCK10, RGS3, MYC, OGFOD1, DPF1, HABP4, TRIM25, SMCO2, KIF21A, SMCO4, KPNA2, LZTS1, EPHB1, SYBU, GAS2L3, TANGO6, LIG1, SFMBT1, ZGRF1, PUS1, TOMM34, VRK1, LMO7, TEX2, KNSTRN, ATAD3A, PRLR, GTPBP4, SGO2, DKC1, GRWD1, TIMELESS, SRFBP1, ALPK3, TRIM16, TTLL12, EZR, RAI14, COL17A1, DNMT1, CDCA3, SDC4, NOP2, NOLC1, FBXO43, BRIP1, DSN1, ORC1, FIGNL1, HIVEP1, EIF4EBP1, BRIX1, HIVEP2, LYAR, PAG1, FANCI, ANKRD34A, STIL, ATP8B1, ALYREF, GGH, DCLK2, PUM3, SSH1, POLA1, PPFIBP1, PEX5L, UBE2S, TDP1, CEP83, AMOTL2, LRP12, RAD18, NFE2L1, EZH2, NFKBIB, SYNM, CIITA, USP31, SH2D4A, MCM8, DOCK9, PAK1IP1, DDX12P, HMGB2, KIAA1671, AFF3, CHTF18, SIPA1L3, LMNB2, WDR43, CHAF1B, CHAF1A, C1QTNF1, BCL7A, ZNF280B, NFKBIZ, SPDL1, LRRFIP1, RRP15, ZNF365, PARP1, PARP2, TWNK, CKAP2, AEN, HAUS6, KIF22, PPRC1, CIT, GNL3, DBF4, POLR1A, INCENP, COL6A2, PECAM1, TMEM106C, SGK3, DNAJC9, HAGH, FOXC2, SLC43A3, PKN3, MAK16, RPL36A, ATP10A, AKAP5, LIN9, SAMHD1, AURKA, POLD3, NRIP2, POLD1, NCR3LG1, NSD2, LRRC8C, RBBP8, USP1, GPC5, BEND6, NOP56, GALNT7, MYEF2, MYO10, UTP4, IRX3, PLCL2, LYSMD2, C9ORF40, BYSL, TTLL4, POLE3, SPAG1, POLR3G, NCAPD3, FAM98A, EVI2B	531	5678	20583	1.15373341	1	1	1
UP_SEQ_FEATURE	CROSSLNK:Glycyl lysine isopeptide (Lys-Gly) (interchain with G-Cter in SUMO1); alternate	11	1.91638	0.01979	PKM, CHAF1A, PARP1, ANXA2, DKC1, RUVBL1, HABP4, NOLC1, RANGAP1, WDR43, RRP15	531	182	20583	2.34280127	1	1	1
UP_SEQ_FEATURE	BINDING:in other chain	5	0.87108	0.02248	ODC1, GMPR, SAMHD1, NGF, PFKP	531	42	20583	4.61460856	1	1	1
UP_SEQ_FEATURE	REPEAT:WD 3	14	2.43902	0.02256	UTP15, SEH1L, UTP4, PAK1IP1, WDR89, WDR43, RRP9, CORO1C, SNRNP40, CHAF1B, RFWD3, GRWD1, POC1A, KIF21A	531	269	20583	2.01739021	1	1	1
UP_SEQ_FEATURE	MUTAGEN:C->A: Loss of activity.	3	0.52265	0.02597	DNMT1, GGH, LRAT	531	10	20583	11.6288136	1	1	1
UP_SEQ_FEATURE	REPEAT:WD 1	14	2.43902	0.02778	UTP15, SEH1L, UTP4, PAK1IP1, WDR89, WDR43, RRP9, CORO1C, SNRNP40, CHAF1B, RFWD3, GRWD1, POC1A, KIF21A	531	277	20583	1.95912623	1	1	1
UP_SEQ_FEATURE	REPEAT:WD 2	14	2.43902	0.02778	UTP15, SEH1L, UTP4, PAK1IP1, WDR89, WDR43, RRP9, CORO1C, SNRNP40, CHAF1B, RFWD3, GRWD1, POC1A, KIF21A	531	277	20583	1.95912623	1	1	1
UP_SEQ_FEATURE	DOMAIN:C2 DOCK-type	3	0.52265	0.03121	DOCK10, DOCK9, DOCK2	531	11	20583	10.5716487	1	1	1
UP_SEQ_FEATURE	DOMAIN:DOCKER	3	0.52265	0.03121	DOCK10, DOCK9, DOCK2	531	11	20583	10.5716487	1	1	1
UP_SEQ_FEATURE	REPEAT:WD 6	13	2.26481	0.03476	UTP15, SEH1L, UTP4, PAK1IP1, WDR89, WDR43, RRP9, CORO1C, SNRNP40, CHAF1B, GRWD1, POC1A, KIF21A	531	257	20583	1.96075974	1	1	1
UP_SEQ_FEATURE	ACT_SITE:Nucleophile	17	2.96167	0.03863	OTUD4, USP13, USP31, PUS1, NOP2, GCNT1, GGH, UCHL3, PFAS, CYLD, DDAH1, DKC1, PPAT, TDP1, USP1, PGP, MGLL	531	381	20583	1.72956982	1	1	1
UP_SEQ_FEATURE	COMPBIAS:Acidic residues	36	6.27178	0.03938	LTV1, MAK16, NOP2, GMNN, HMGB2, PPAN, TONSL, WDR43, HS6ST3, TUBB6, RGS3, CHAF1A, HIVEP1, UTP20, KIF21A, HIVEP2, LRRFIP1, POLE, RRP15, CADPS2, SFMBT1, ATP8B1, TAF13, IRX3, ABCC9, RANGAP1, GNL3, POLA1, TUBB2A, POLR1A, GRWD1, MRTO4, POLE3, TIMELESS, POLR3G, EZH2	531	995	20583	1.40246998	1	1	1
UP_SEQ_FEATURE	REPEAT:WD 5	13	2.26481	0.04164	UTP15, SEH1L, UTP4, PAK1IP1, WDR89, WDR43, RRP9, CORO1C, SNRNP40, CHAF1B, GRWD1, POC1A, KIF21A	531	264	20583	1.9087699	1	1	1
UP_SEQ_FEATURE	REPEAT:WD 4	13	2.26481	0.04432	UTP15, SEH1L, UTP4, PAK1IP1, WDR89, WDR43, RRP9, CORO1C, SNRNP40, CHAF1B, GRWD1, POC1A, KIF21A	531	267	20583	1.88732305	1	1	1

Table S5. Gene transcripts upregulated by LIGHT, IL-17, and LIGHT with IL-17, in human pulmonary fibroblasts, associated with Fig. 2D.

Group	IL17A.Vs.Untreated	gene	IL17A.Vs.Untreated:log2FoldChangeShrunken	IL17A.Vs.Untreated:padj	LIGHT.Vs.Untreated:log2FoldChangeShrunken	LIGHT.Vs.Untreated:padj	IL17A_LIGHT.Vs.Untreated:log2FoldChangeShrunken	IL17A_LIGHT.Vs.Untreated:padj
7	CCDC170	ENSG00000120262.9	0.75	0.041	-0.45	0.25	0.09	0.85
7	CNN1	ENSG00000130176.7	0.61	0.0046	-0.03	0.93	0.49	0.01
7	ACTG2	ENSG00000163017.13	0.59	8.30E-04	0.02	0.94	0.49	0.0019
7	LMOD1	ENSG00000163431.12	0.73	5.10E-07	0.08	0.71	0.35	0.016
7	GPR37	ENSG00000170775.2	0.64	8.80E-11	0.14	0.24	0.52	4.50E-08
7	AC144652.	ENSG00000273117.1	0.78	0.0092	0.25	0.47	0.27	0.39
7	AC037198.	ENSG00000276107.1	0.6	0.0074	-0.65	0.0033	0.26	0.24
484	CXCL3	ENSG00000163734.4	0.73	0.074	-0.51	0.23	2.45	5.30E-19
484	COLCA2	ENSG00000214290.8	-0.15	2	2.45	2	2.41	7.60E-04
484	AL355607	ENSG00000260454.1	0.33	2	1.01	2	2.27	1.30E-04
484	CCL20	ENSG00000115009.11	0.38	0.61	0.03	0.99	2.2	0.0012
484	AC00518(	ENSG00000267405.1	0.9	2	1.27	2	2.1	5.40E-04
484	NIPAL4	ENSG00000172548.14	0.8	2	0.95	0.073	2.07	2.60E-05
484	CSF3	ENSG00000108342.12	0.94	0.14	0.33	0.59	2.06	1.00E-04
484	AC107308	ENSG00000277945.1	1.48	2	0.81	2	2.04	4.10E-05
484	ASB2	ENSG00000100628.11	1.35	2	0.99	2	1.92	0.0026
484	CST2	ENSG00000170369.3	0.38	2	1.48	2	1.88	0.004
484	LINC0205	ENSG00000226859.1	-0.14	2	1.58	2	1.8	0.0029
484	CSF2	ENSG00000164400.5	0	1	0.63	0.12	1.79	5.70E-04
484	SMCO2	ENSG00000165935.9	0.49	2	1.09	2	1.79	0.0062
484	AL161891	ENSG00000276672.1	1.04	2	0.91	0.1	1.76	4.20E-04
484	AC010247	ENSG00000259436.1	0.29	2	0.66	2	1.75	0.0067
484	TRGV7	ENSG00000249978.1	0.28	2	0.81	2	1.72	0.0097
484	IL6	ENSG00000136244.11	0.92	0.075	0.66	0.16	1.71	1.60E-05
484	MYB	ENSG00000118513.18	0.69	2	0.42	0.47	1.7	0.0023
484	CIITA	ENSG00000179583.18	1.06	2	0.74	2	1.69	0.0023
484	AC023906	ENSG00000259712.1	0.87	2	2.03	2	1.65	0.0049
484	ARHGAP4(	ENSG00000124143.10	-0.84	2	0.31	2	1.63	0.019
484	LINC0201	ENSG00000231574.5	0.67	2	1.18	2	1.63	0.0013
484	AC003092	ENSG00000236453.5	0.65	2	1.02	0.08	1.57	0.003
484	AC010247	ENSG00000254887.1	-0.58	2	0.02	2	1.56	0.018
484	PTGS2	ENSG00000073756.11	0.46	0.49	0.87	0.064	1.55	2.10E-04
484	MIR3142H	ENSG00000253522.5	0.2	2	0.03	0.98	1.54	0.0094
484	AC022509	ENSG00000256894.1	0.97	2	0.73	2	1.54	0.0099
484	PTGDR	ENSG00000168229.3	0.27	2	1.42	2	1.53	0.013
484	LINC0045	ENSG00000229373.8	1.19	2	0.59	0.3	1.53	0.001
484	NFAM1	ENSG00000235568.6	-0.47	2	0.81	0.18	1.52	0.0057
484	NPAS4	ENSG00000174576.9	1.31	2	0.13	2	1.49	0.0084
484	AC022532	ENSG00000280401.1	0.67	2	0.9	2	1.48	0.024
484	PF4V1	ENSG00000109272.3	-0.32	2	0.53	2	1.47	0.022
484	NPY4R	ENSG00000204174.7	0.37	2	0.4	2	1.46	0.017
484	PSG1	ENSG00000231924.9	1.01	2	0.62	0.25	1.46	7.90E-04
484	TRIML2	ENSG00000179046.8	-0.04	2	0.66	2	1.45	0.037
484	PECAM1	ENSG00000261371.5	0.31	2	1.19	2	1.45	0.011
484	SP6	ENSG00000189120.4	-0.38	2	0.22	0.72	1.41	4.20E-04
484	RNPS1P1	ENSG00000250896.1	0.83	2	0.93	2	1.39	0.016
484	SYT9	ENSG00000170743.16	-0.29	2	1.31	2	1.36	0.044
484	AC079305	ENSG00000222043.2	0.65	2	0.64	2	1.34	0.011
484	EGF	ENSG00000138798.11	0.76	2	1.03	2	1.33	0.027
484	XIRP2	ENSG00000163092.19	0.66	2	1.11	2	1.33	0.029
484	KCND3	ENSG00000171385.9	0.54	2	0.9	2	1.33	0.0069
484	GAPLINC	ENSG00000266835.5	0.71	2	1.03	2	1.33	0.033
484	ZP4	ENSG00000116996.9	0.35	2	0.77	2	1.32	0.013
484	GREM1	ENSG00000166923.10	0.56	1.30E-07	0.36	8.20E-04	1.32	3.40E-46
484	KLHDC7B	ENSG00000130487.5	0.54	2	0.33	0.43	1.31	0.0036
484	CNTF	ENSG00000242689.2	0.79	2	0.65	0.25	1.31	0.0054
484	ETV7	ENSG00000010030.13	0.49	0.52	1.02	0.057	1.3	0.011
484	AL121987	ENSG00000227741.1	1.15	2	0.37	2	1.29	0.02
484	CALB2	ENSG00000172137.18	0.83	0.12	0.46	0.38	1.28	0.0012
484	MIRLET7D	ENSG00000199133.3	0.94	2	0.31	0.62	1.28	0.0046

484	CACNB2	ENSG00000165995.18	0.06	2	0.58	2	1.25	0.035
484	AC073130	ENSG00000237870.6	0.81	2	0.74	2	1.25	0.019
484	AP001350	ENSG00000280010.1	0.62	2	0.66	2	1.24	0.03
484	INHBA	ENSG00000122641.10	0.55	3.60E-10	0.54	1.30E-10	1.23	1.20E-58
484	ST6GAL2	ENSG00000144057.15	0.94	0.16	0.92	0.1	1.21	0.026
484	SLC8A1-A	ENSG00000227028.6	0.22	2	0.72	2	1.21	0.037
484	DEPDC1B	ENSG00000035499.12	0.13	0.81	0.44	0.13	1.2	1.20E-07
484	RHEBL1	ENSG00000167550.10	0.24	2	0.79	0.17	1.2	0.022
484	C15orf48	ENSG00000166920.12	0.52	0.42	0.23	0.69	1.19	0.015
484	SYT2	ENSG00000143858.11	0.51	2	0.8	0.13	1.17	0.013
484	ASRGL1	ENSG00000162174.12	0.5	2	0.71	0.21	1.16	0.017
484	GPR1	ENSG00000183671.12	0.04	0.96	0.28	0.64	1.16	0.0066
484	AC069499	ENSG00000241634.1	0.67	2	0.95	0.066	1.16	0.013
484	MIR221	ENSG00000207870.1	0.43	0.4	0.32	0.47	1.15	2.30E-04
484	DDX12P	ENSG00000214826.5	0.56	0.32	0.61	0.16	1.15	0.0018
484	AC011498	ENSG00000280239.1	0.67	2	0.47	0.29	1.15	7.70E-04
484	FBXO43	ENSG00000156509.13	0.57	2	0.77	0.2	1.14	0.032
484	NCR3LG1	ENSG00000188211.8	0.4	0.018	0.56	5.60E-05	1.14	1.70E-20
484	ZNF726	ENSG00000213967.10	0.35	2	0.68	0.15	1.13	0.0048
484	AURKB	ENSG00000178999.12	0.3	0.33	0.53	0.016	1.12	2.30E-09
484	CLDN16	ENSG00000113946.3	0.64	2	1.15	2	1.11	0.042
484	AC093503	ENSG00000269292.1	0.66	2	0.81	0.13	1.11	0.02
484	AC004917	ENSG00000243797.6	0.9	2	1.09	2	1.1	0.05
484	IDO1	ENSG00000131203.12	-0.02	1	0.73	0.17	1.09	0.043
484	ELN	ENSG00000049540.16	0.37	0.094	-0.11	0.66	1.08	1.10E-12
484	PRR26	ENSG00000180525.11	0.51	0.42	0.82	0.069	1.08	0.0077
484	ARRDC3-A	ENSG00000281357.2	-0.03	2	0.78	0.16	1.08	0.027
484	AC006978	ENSG00000235859.5	0.67	2	0.44	0.48	1.07	0.032
484	TPX2	ENSG00000088325.15	0.38	0.16	0.56	0.0071	1.06	6.70E-09
484	AC016026	ENSG00000093100.13	0.72	2	0.4	2	1.06	0.028
484	TDRKH	ENSG00000182134.15	0.43	0.47	0.08	0.9	1.06	0.0032
484	AL138724	ENSG00000272269.1	-0.09	0.83	0.5	0.018	1.05	2.80E-09
484	XRCC3	ENSG00000126215.13	0.28	0.32	0.52	0.0079	1.04	8.20E-10
484	ACTC1	ENSG00000159251.6	-0.1	0.92	-0.11	0.84	1.04	0.015
484	FANCB	ENSG00000181544.13	0.62	0.12	0.37	0.33	1.04	3.40E-04
484	DMC1	ENSG00000100206.9	0.28	0.69	0.45	0.32	1.03	0.0033
484	CDC25C	ENSG00000158402.18	0.35	0.47	0.48	0.18	1.03	3.90E-04
484	GPR3	ENSG00000181773.6	0.69	0.091	0.46	0.22	1.02	7.50E-04
484	LRRC15	ENSG00000172061.8	0.47	0.29	0.63	0.067	1.01	4.90E-04
484	3-Mar	ENSG00000173926.5	0.55	0.063	0.18	0.58	1.01	3.20E-06
484	GCNT4	ENSG00000176928.5	0.43	0.56	0.32	0.61	1.01	0.032
484	SERPINB2	ENSG00000197632.8	0.35	0.032	0.41	0.0029	1.01	3.50E-17
484	PLS1	ENSG00000120756.12	0.7	2	0.6	0.31	1	0.048
484	AKAP5	ENSG00000179841.8	0.28	0.66	0.64	0.095	1	0.0024
484	MIR600H	ENSG00000236901.6	1.15	2	0.55	2	1	0.047
484	AC078785	ENSG00000240057.5	0.14	2	0.65	0.18	1	0.018
484	CDCA3	ENSG00000111665.11	0.08	0.88	0.54	0.033	0.99	5.10E-06
484	PRLR	ENSG00000113494.16	-0.16	0.84	0.55	0.2	0.99	0.0048
484	KIAA1671	ENSG00000197077.13	0.18	2	0.63	0.16	0.99	0.01
484	IL20RB	ENSG00000174564.12	0.64	0.21	0.52	0.24	0.98	0.0065
484	MTMR7	ENSG00000003987.13	-0.26	2	0.62	0.21	0.97	0.022
484	ANOS1	ENSG00000011201.11	0.85	2	0.51	0.22	0.97	0.021
484	LINC0096	ENSG00000246430.6	0.78	0.054	0.58	0.12	0.97	0.0025
484	AC080188	ENSG00000279384.1	-0.07	2	0.03	2	0.97	0.046
484	CPA4	ENSG00000128510.10	0.57	0.022	0.54	0.013	0.96	8.40E-07
484	MAMDC2	ENSG00000165072.9	0.17	0.36	0.56	2.00E-06	0.96	2.90E-19
484	HIVEP1	ENSG00000095951.16	0.52	2.60E-10	0.52	3.40E-11	0.95	2.40E-38
484	TMC7	ENSG00000170537.12	0.87	2	0.56	0.24	0.95	0.02
484	LINC0158	ENSG00000259518.1	0.05	0.96	0	1	0.95	0.012
484	AC132872	ENSG00000280407.2	0.43	0.4	0.5	0.2	0.95	0.0029
484	TNFRSF12	ENSG00000006327.13	0.54	8.80E-06	0.54	2.20E-06	0.94	1.60E-18
484	ORC1	ENSG00000085840.12	0.09	0.84	0.45	0.06	0.94	2.50E-06
484	MTFR2	ENSG00000146410.11	0.19	0.79	0.63	0.11	0.94	0.0062
484	PKN3	ENSG00000160447.6	0.36	0.21	0.54	0.013	0.94	8.90E-07
484	SERPINB8	ENSG00000166401.14	0.46	3.70E-06	0.48	2.50E-07	0.94	2.00E-28

484	ARHGAP11	ENSG00000198826.10	0.36	0.072	0.55	4.60E-04	0.94	4.40E-11
484	HIVEP2	ENSG00000010818.9	0.32	0.0065	0.52	4.20E-08	0.93	3.00E-26
484	IP6K3	ENSG00000161896.11	0.18	0.63	0.52	0.014	0.93	3.10E-07
484	PPM1L	ENSG00000163590.13	0.39	0.47	0.32	0.47	0.93	0.0043
484	RGMB	ENSG00000174136.11	0.32	0.001	0.54	9.20E-11	0.93	2.20E-32
484	NTF3	ENSG00000185652.11	0.55	2	0.76	0.15	0.93	0.047
484	CLN3	ENSG00000188603.18	0.28	2	0.63	0.24	0.93	0.045
484	HMSD	ENSG00000221887.5	0.29	0.67	0.22	0.67	0.93	0.01
484	MAMLD1	ENSG00000013619.13	0.58	9.40E-07	0.35	0.0044	0.92	1.90E-18
484	GCNT2	ENSG00000111846.16	0.31	0.45	0.53	0.062	0.92	1.20E-04
484	AGMAT	ENSG00000116771.5	0.4	0.35	0.52	0.11	0.92	6.80E-04
484	MICALCL	ENSG00000133808.4	0.57	0.29	0.56	0.21	0.92	0.013
484	HHIP	ENSG00000164161.9	0.59	0.13	0.46	0.19	0.92	0.0015
484	FDCSP	ENSG00000181617.5	0	1	0	1	0.92	0.02
484	AC096921	ENSG00000261468.1	0.45	0.5	0.36	0.51	0.92	0.028
484	SPIB	ENSG00000269404.6	0	2	0	2	0.92	0.049
484	PDGFB	ENSG00000100311.16	0.61	0.061	0.21	0.56	0.91	2.00E-04
484	STIL	ENSG00000123473.15	0.19	0.53	0.57	0.0012	0.91	9.60E-09
484	SLC20A2	ENSG00000168575.9	0.52	4.00E-05	0.56	1.10E-06	0.91	5.90E-17
484	LYN	ENSG00000254087.7	0.48	0.039	0.22	0.36	0.91	1.50E-07
484	VDR	ENSG00000111424.10	0.44	7.10E-07	0.47	1.60E-08	0.9	1.70E-31
484	RERG	ENSG00000134533.6	0.21	0.66	0	0.99	0.9	1.10E-04
484	FGF5	ENSG00000138675.16	0.4	0.0072	0.43	9.00E-04	0.9	5.10E-15
484	LGI2	ENSG00000153012.11	0.59	2	1.23	2	0.9	0.035
484	RAB3B	ENSG00000169213.6	0.36	4.20E-04	0.52	4.40E-09	0.9	8.10E-27
484	GADD45A	ENSG00000116717.11	0.34	0.014	0.54	1.30E-06	0.89	2.80E-17
484	TROAP	ENSG00000135451.12	0.41	0.31	0.55	0.074	0.89	7.90E-04
484	BRIP1	ENSG00000136492.8	0.18	0.58	0.45	0.019	0.89	9.30E-08
484	CHAF1B	ENSG00000159259.7	0.43	0.027	0.43	0.012	0.89	1.20E-09
484	MGLL	ENSG00000074416.13	0.45	3.30E-05	0.43	2.30E-05	0.88	4.40E-21
484	LRP8	ENSG00000157193.15	0.37	0.062	0.56	4.30E-04	0.88	8.00E-10
484	CHAF1A	ENSG00000167670.15	0.31	0.027	0.45	6.10E-05	0.88	6.60E-18
484	C6orf223	ENSG00000181577.15	0.54	0.32	0.31	0.54	0.88	0.019
484	PSG5	ENSG00000204941.13	0.37	0.47	0.26	0.54	0.88	0.0049
484	AP003356	ENSG00000253669.3	0.68	0.15	0.42	0.34	0.88	0.013
484	SLC11A2	ENSG00000110911.14	0.35	1.10E-04	0.36	1.20E-05	0.87	2.10E-31
484	FAM53A	ENSG00000174137.12	0.83	0.052	0.35	0.44	0.87	0.013
484	BIRC5	ENSG00000089685.14	0.29	0.35	0.48	0.031	0.86	9.30E-06
484	NALCN	ENSG00000102452.15	0.19	0.63	0.58	0.0095	0.86	1.50E-05
484	TRIM14	ENSG00000106785.14	0.46	4.10E-05	0.47	5.80E-06	0.86	1.10E-19
484	OGFRL1	ENSG00000119900.7	0.57	3.60E-14	0.27	5.40E-04	0.86	1.60E-36
484	FANCI	ENSG00000140525.17	0.24	0.27	0.48	0.0015	0.86	3.30E-10
484	PALM2-AI	ENSG00000157654.17	0.22	2	0.41	0.34	0.86	0.012
484	SHMT1	ENSG00000176974.19	0.18	0.65	0.54	0.019	0.86	2.30E-05
484	SLC22A4	ENSG00000197208.5	0.03	0.91	0.49	1.80E-04	0.86	1.80E-13
484	AC016205	ENSG00000267374.1	0.13	0.88	0.76	0.071	0.86	0.025
484	TRIO	ENSG00000038382.18	0.43	5.80E-12	0.5	2.40E-17	0.85	2.40E-50
484	MCM6	ENSG00000076003.4	0.29	0.033	0.53	4.20E-07	0.85	6.40E-18
484	PAG1	ENSG00000076641.4	0.16	0.51	0.46	0.0015	0.85	3.60E-11
484	RBBP8	ENSG00000101773.18	0.24	0.14	0.44	2.00E-04	0.85	9.40E-16
484	CRACR2A	ENSG00000130038.9	0.46	0.13	0.35	0.2	0.85	9.50E-05
484	CHRNB3	ENSG00000147432.6	0.32	2	1.4	2	0.85	0.043
484	SERPINB7	ENSG00000166396.12	0.53	0.23	0.2	0.66	0.85	0.0063
484	ZNF239	ENSG00000196793.13	0.43	0.067	0.35	0.095	0.85	4.00E-07
484	TAP2	ENSG00000204267.13	0.06	0.84	0.58	6.80E-06	0.85	3.90E-13
484	DDIT4L	ENSG00000145358.6	0.56	0.23	0.24	0.6	0.84	0.011
484	PLCL2	ENSG00000154822.17	0.32	0.081	0.56	7.30E-05	0.84	3.90E-11
484	GJD3	ENSG00000183153.6	0.43	0.2	0.53	0.044	0.84	2.00E-04
484	AFAP1	ENSG00000196526.10	0.41	9.50E-08	0.48	2.80E-11	0.84	1.40E-34
484	SUGT1P1	ENSG00000226823.1	0.56	2	0.53	0.27	0.84	0.037
484	AC130371	ENSG00000274370.1	0.77	0.13	0.54	0.25	0.84	0.033
484	USP31	ENSG00000103404.14	0.15	0.43	0.58	5.10E-07	0.83	1.70E-14
484	SLC43A3	ENSG00000134802.17	0.12	0.51	0.52	7.40E-07	0.83	1.10E-17
484	KIF11	ENSG00000138160.5	0.09	0.79	0.45	0.013	0.83	1.80E-07
484	TEAD4	ENSG00000197905.8	0.22	0.37	0.37	0.03	0.83	1.30E-08
484	AC010168	ENSG00000261324.2	0.5	0.24	0	1	0.83	0.0046

484	SRGN	ENSG00000122862.4	0.3	0.25	0.31	0.14	0.82	9.80E-07
484	TRIM55	ENSG00000147573.16	0.4	0.16	0.47	0.034	0.82	1.90E-05
484	TIMELESS	ENSG00000111602.11	0.22	0.11	0.46	3.30E-06	0.81	1.50E-18
484	EPHA2	ENSG00000142627.12	0.32	9.40E-04	0.55	1.50E-11	0.81	7.10E-25
484	RACGAP1	ENSG00000161800.12	0.25	0.29	0.49	0.0032	0.81	5.00E-08
484	POC1A	ENSG00000164087.7	0.41	0.13	0.45	0.049	0.81	2.80E-05
484	PLK1	ENSG00000166851.14	0.32	0.23	0.47	0.019	0.81	3.50E-06
484	RPL36A	ENSG00000241343.9	0.46	0.0074	0.42	0.0072	0.81	2.70E-09
484	AL158206	ENSG00000260912.1	0.52	2	0.68	0.13	0.81	0.043
484	KIF20A	ENSG00000112984.11	0.37	0.27	0.36	0.19	0.8	3.60E-04
484	SLC39A8	ENSG00000138821.12	0.56	0.16	0.48	0.17	0.8	0.0055
484	SLC43A2	ENSG00000167703.14	0.27	0.26	0.29	0.14	0.8	1.60E-07
484	PUS1	ENSG00000177192.13	0.34	0.016	0.52	7.60E-06	0.8	1.10E-13
484	PCED1B	ENSG00000179715.12	0.23	0.64	0.17	0.67	0.8	0.002
484	INSIG1	ENSG00000186480.12	0.37	0.0021	0.27	0.016	0.8	1.10E-16
484	MICB	ENSG00000204516.9	0.37	0.042	0.58	4.90E-05	0.8	1.60E-09
484	RANGAP1	ENSG00000100401.19	0.45	6.10E-06	0.54	2.20E-09	0.79	1.40E-19
484	PPIF	ENSG00000108179.13	0.2	0.025	0.57	5.80E-18	0.79	2.00E-35
484	MYH15	ENSG00000144821.9	-0.38	0.53	0.64	0.096	0.79	0.023
484	HMGA2	ENSG00000149948.13	0.24	0.016	0.56	2.90E-13	0.79	6.30E-27
484	DDAH1	ENSG00000153904.18	0.44	1.10E-04	0.54	1.40E-07	0.79	4.20E-16
484	NEGR1	ENSG00000172260.14	0.28	0.027	0.47	2.40E-06	0.79	5.80E-18
484	CADPS2	ENSG00000081803.15	0.41	0.03	0.51	0.0012	0.78	3.80E-08
484	XYLT1	ENSG00000103489.11	0.21	0.14	0.56	3.10E-08	0.78	3.70E-16
484	SEMA7A	ENSG00000138623.9	0.32	0.0012	0.55	1.70E-11	0.78	1.10E-23
484	WDR89	ENSG00000140006.11	0.13	0.62	0.58	3.70E-05	0.78	1.50E-09
484	LYSMD2	ENSG00000140280.13	0.25	0.43	0.32	0.17	0.78	2.90E-05
484	NCAPG2	ENSG00000146918.19	0.34	0.048	0.51	1.70E-04	0.78	4.40E-10
484	VPS37C	ENSG00000167987.10	0.22	0.017	0.56	4.90E-16	0.78	9.10E-32
484	WNT7B	ENSG00000188064.9	-0.62	0.28	0.37	0.43	0.78	0.04
484	VPS9D1-A	ENSG00000261373.1	0.15	0.74	0.38	0.15	0.78	3.30E-04
484	TNC	ENSG00000041982.15	0.48	7.00E-07	0.56	2.90E-10	0.77	4.50E-19
484	MTHFD2	ENSG00000065911.11	0.5	1.80E-04	0.31	0.019	0.77	1.90E-11
484	PODXL	ENSG00000128567.16	0.29	0.29	0.57	0.0027	0.77	9.20E-06
484	GGH	ENSG00000137563.11	0.32	0.073	0.5	3.30E-04	0.77	1.30E-09
484	TUBA1C	ENSG00000167553.15	0.3	0.021	0.46	1.60E-05	0.77	2.10E-14
484	HACD2	ENSG00000206527.9	0.31	0.0021	0.53	1.00E-10	0.77	3.70E-23
484	VRK1	ENSG00000100749.7	0.25	0.29	0.45	0.0097	0.76	4.30E-07
484	EVA1A	ENSG00000115363.13	0.46	0.012	0.5	0.0018	0.76	9.80E-08
484	ARHGEF39	ENSG00000137135.17	-0.09	0.92	0.24	0.64	0.76	0.041
484	TPM1	ENSG00000140416.19	0.43	3.20E-06	0.41	2.00E-06	0.76	3.70E-21
484	C16orf59	ENSG00000162062.14	0.08	0.86	0.42	0.067	0.76	1.00E-04
484	REL	ENSG00000162924.13	0.05	0.93	0.44	0.07	0.76	2.40E-04
484	KIAA1524	ENSG00000163507.13	0.21	0.42	0.47	0.0051	0.76	3.60E-07
484	LINC0047	ENSG00000233237.6	0.46	0.14	-0.02	0.96	0.76	6.70E-04
484	MTFP1	ENSG00000242114.5	-0.12	0.89	0.47	0.31	0.76	0.05
484	TANGO6	ENSG00000103047.7	0.32	0.047	0.37	0.0064	0.75	1.90E-10
484	SDC4	ENSG00000124145.6	0.36	1.50E-08	0.55	2.60E-21	0.75	1.50E-40
484	MCM8	ENSG00000125885.13	0.06	0.84	0.48	9.20E-04	0.75	1.10E-08
484	DOCK2	ENSG00000134516.15	0.52	0.0082	0.42	0.023	0.75	1.80E-06
484	PPRC1	ENSG00000148840.10	0.35	6.40E-05	0.42	6.80E-08	0.75	2.50E-24
484	IL1RAP	ENSG00000196083.9	0.24	0.5	0.41	0.096	0.75	2.30E-04
484	YRDC	ENSG00000196449.3	0.47	0.0067	0.5	7.90E-04	0.75	5.60E-08
484	AKAP2	ENSG00000241978.9	0.52	0.1	0.55	0.035	0.75	0.0014
484	EXOSC5	ENSG00000077348.8	0.13	0.63	0.41	0.0059	0.74	1.10E-08
484	CDKN3	ENSG00000100526.19	0.19	0.69	0.37	0.21	0.74	0.0022
484	MCM4	ENSG00000104738.16	0.2	0.27	0.41	0.0012	0.74	5.90E-11
484	BCL7A	ENSG00000110987.8	0.13	0.51	0.44	2.10E-04	0.74	2.50E-12
484	RNF19B	ENSG00000116514.16	0.29	0.12	0.45	0.0023	0.74	1.70E-08
484	ETS1	ENSG00000134954.14	0.33	1.10E-06	0.42	2.50E-11	0.74	4.90E-37
484	TTLL4	ENSG00000135912.10	-0.16	0.23	0.5	1.50E-08	0.74	1.00E-18
484	PCSK9	ENSG00000169174.10	0.48	0.097	0.19	0.52	0.74	6.20E-04
484	CDCA4	ENSG00000170779.10	0.35	0.074	0.4	0.016	0.74	2.60E-07
484	CYCS	ENSG00000172115.8	0.23	0.09	0.51	5.50E-07	0.74	1.00E-14
484	GAS6	ENSG00000183087.14	0.57	8.10E-05	0.32	0.033	0.74	4.50E-09
484	SH3RF3-A	ENSG00000259863.1	0.57	0.026	0.45	0.062	0.74	2.90E-04

484	MOCOS	ENSG00000075643.5	0.47	0.045	0.19	0.43	0.73	4.50E-05
484	MYEF2	ENSG00000104177.17	0.01	0.97	0.47	0.0014	0.73	3.10E-08
484	GMNN	ENSG00000112312.9	0.15	0.57	0.56	1.30E-04	0.73	1.10E-07
484	PLXNA1	ENSG00000114554.11	0.21	0.0092	0.57	4.30E-19	0.73	8.50E-32
484	TUBA1B	ENSG00000123416.15	0.42	0.0037	0.35	0.008	0.73	2.90E-10
484	TMEM2	ENSG00000135048.13	0.3	0.0037	0.4	6.20E-06	0.73	8.50E-19
484	DOCK10	ENSG00000135905.18	0.16	0.1	0.46	2.40E-11	0.73	2.00E-29
484	EXOC6	ENSG00000138190.16	0.24	0.19	0.53	4.30E-05	0.73	9.20E-10
484	HYOU1	ENSG00000149428.18	0.22	0.002	0.56	1.40E-22	0.73	5.90E-38
484	ATP6V0E2	ENSG00000171130.17	0.32	0.032	0.4	0.0012	0.73	2.80E-11
484	C3orf70	ENSG00000187068.2	0.46	0.29	0.24	0.55	0.73	0.015
484	C10orf55	ENSG00000222047.8	0.42	0.31	0.4	0.24	0.73	0.008
484	NOP16	ENSG00000048162.20	0.14	0.57	0.58	3.10E-05	0.72	4.40E-08
484	SLC16A3	ENSG00000141526.16	0.32	7.10E-06	0.55	9.80E-19	0.72	3.60E-32
484	INCENP	ENSG00000149503.12	0.27	0.15	0.44	0.0016	0.72	1.10E-08
484	NCAPD3	ENSG00000151503.12	0.13	0.59	0.55	6.60E-05	0.72	2.30E-08
484	CCNB2	ENSG00000157456.7	0.23	0.48	0.36	0.11	0.72	1.40E-04
484	RFX8	ENSG00000196460.12	-0.1	0.82	0.52	0.018	0.72	3.30E-04
484	WFDC21P	ENSG00000261040.6	0.57	0.064	-0.12	0.74	0.72	0.0025
484	DBF4	ENSG00000006634.7	0.24	0.35	0.47	0.0073	0.71	7.00E-06
484	TARBP1	ENSG00000059588.9	0.15	0.53	0.31	0.05	0.71	7.60E-08
484	UHRF1BP1	ENSG00000065060.16	0.32	0.04	0.58	1.30E-06	0.71	3.50E-10
484	LIPG	ENSG00000101670.11	0.34	0.15	0.24	0.25	0.71	1.70E-05
484	SH2D4A	ENSG00000104611.11	0.52	0.0014	0.41	0.0071	0.71	2.10E-07
484	GLIS3	ENSG00000107249.21	0.5	1.20E-04	0.29	0.026	0.71	8.60E-11
484	ZGRF1	ENSG00000138658.15	0.03	0.95	0.38	0.061	0.71	3.80E-05
484	SLFN13	ENSG00000154760.13	0.31	0.41	0.28	0.36	0.71	0.0024
484	NOLC1	ENSG00000166197.16	0.23	0.0089	0.49	2.70E-12	0.71	8.10E-26
484	H2AFX	ENSG00000188486.3	0.3	0.043	0.54	2.60E-06	0.71	1.20E-10
484	CD47	ENSG00000196776.14	0.14	0.15	0.57	1.90E-18	0.71	7.70E-29
484	ITGA1	ENSG00000213949.8	0.43	1.40E-06	0.27	0.0032	0.71	1.50E-19
484	RFC2	ENSG00000049541.10	0.31	0.016	0.58	1.50E-08	0.7	1.10E-12
484	CCNE1	ENSG00000105173.13	0.34	0.32	0.34	0.22	0.7	0.0016
484	NOP2	ENSG00000111641.11	0.22	0.05	0.49	1.00E-08	0.7	3.90E-18
484	ATP1B1	ENSG00000143153.12	0.24	0.16	0.41	0.0015	0.7	7.80E-10
484	AFF3	ENSG00000144218.18	0.27	0.0041	0.46	5.50E-09	0.7	2.30E-21
484	WDR66	ENSG00000158023.9	0.48	0.0034	0.34	0.035	0.7	1.60E-07
484	ABCE1	ENSG00000164163.10	0.15	0.2	0.49	3.70E-10	0.7	1.30E-20
484	CENPN	ENSG00000166451.13	0.15	0.53	0.48	4.70E-04	0.7	1.60E-08
484	MARS	ENSG00000166986.14	0.36	2.20E-06	0.36	3.80E-07	0.7	3.10E-26
484	LRRC8D	ENSG00000171492.14	0.33	0.0043	0.48	3.80E-07	0.7	4.90E-15
484	KPNA2	ENSG00000182481.8	0.25	0.12	0.44	2.30E-04	0.7	1.30E-10
484	SAMD4A	ENSG00000020577.13	0.33	5.80E-09	0.27	1.10E-06	0.69	5.40E-45
484	MTHFD1	ENSG00000100714.15	0.16	0.22	0.45	4.00E-07	0.69	1.50E-16
484	POLA1	ENSG00000101868.10	0.17	0.5	0.41	0.01	0.69	1.00E-06
484	PYCR3	ENSG00000104524.13	0.41	0.0083	0.41	0.0035	0.69	3.10E-08
484	CAP2	ENSG00000112186.11	0.49	0.026	0.3	0.17	0.69	8.60E-05
484	CYP1B1	ENSG00000138061.11	0.35	2.50E-09	0.33	1.80E-09	0.69	1.50E-40
484	GNAL	ENSG00000141404.15	0.5	0.23	0.24	0.55	0.69	0.02
484	GPATCH4	ENSG00000160818.16	0.25	0.093	0.46	4.80E-05	0.69	4.20E-11
484	APBB2	ENSG00000163697.16	0.15	0.11	0.39	1.10E-08	0.69	6.50E-28
484	PTTG1	ENSG00000164611.12	0.31	0.17	0.36	0.05	0.69	1.30E-05
484	TK1	ENSG00000167900.11	0.36	0.049	0.25	0.14	0.69	4.70E-07
484	AEN	ENSG00000181026.14	0.26	0.003	0.53	8.20E-14	0.69	2.60E-24
484	CLDN4	ENSG00000189143.9	0.17	0.52	0.39	0.021	0.69	2.80E-06
484	MARS2	ENSG00000247626.4	0.22	0.52	0.52	0.016	0.69	3.90E-04
484	MCAM	ENSG00000076706.16	0.41	0.0013	0.37	0.0018	0.68	9.10E-11
484	SEC23B	ENSG00000101310.14	0.25	0.0011	0.53	2.60E-17	0.68	4.20E-29
484	POP1	ENSG00000104356.10	0.2	0.21	0.41	2.80E-04	0.68	2.20E-11
484	FBXO5	ENSG00000112029.9	0.11	0.69	0.45	0.0015	0.68	1.10E-07
484	PNPT1	ENSG00000138035.14	0.24	0.084	0.32	0.0039	0.68	3.20E-12
484	CTSK	ENSG00000143387.12	0.03	0.77	0.56	2.80E-27	0.68	3.00E-41
484	CCNA2	ENSG00000145386.9	0.26	0.36	0.53	0.0053	0.68	1.10E-04
484	FXN	ENSG00000165060.11	0.25	0.26	0.52	6.40E-04	0.68	1.20E-06
484	LIMS1	ENSG00000169756.16	0.19	0.056	0.51	2.90E-12	0.68	2.20E-22
484	FAM46C	ENSG00000183508.4	0.19	0.66	0.56	0.025	0.68	0.0033

484	PGP	ENSG00000184207.8	0.4	0.0058	0.55	7.30E-06	0.68	4.30E-09
484	ATP10A	ENSG00000206190.11	0.37	0.12	0.51	0.0062	0.68	4.80E-05
484	DCLRE1B	ENSG00000118655.4	0.16	0.46	0.36	0.012	0.67	5.90E-08
484	PPAN	ENSG00000130810.19	0.22	0.55	0.42	0.083	0.67	0.0013
484	SUV39H2	ENSG00000152455.15	0.11	0.75	0.49	0.0074	0.67	5.80E-05
484	NAV2	ENSG00000166833.19	0.06	0.76	0.52	1.50E-08	0.67	4.80E-14
484	PIK3CD	ENSG00000171608.15	0.35	0.0025	0.38	2.00E-04	0.67	2.10E-13
484	ZDHHC14	ENSG00000175048.16	0.21	0.55	0.48	0.032	0.67	6.60E-04
484	SBSN	ENSG00000189001.10	0.14	0.82	-0.11	0.81	0.67	0.022
484	IARS	ENSG00000196305.17	0.32	7.00E-05	0.29	9.90E-05	0.67	8.20E-23
484	KIF22	ENSG00000079616.12	0.22	0.33	0.42	0.0068	0.66	2.10E-06
484	NFE2L1	ENSG00000082641.15	0.17	0.014	0.34	7.80E-10	0.66	2.70E-36
484	KIAA1549L	ENSG00000110427.14	0.22	0.66	0.28	0.42	0.66	0.013
484	E2F3	ENSG00000112242.14	0.28	0.005	0.39	3.60E-06	0.66	1.30E-17
484	B4GALT6	ENSG00000118276.11	0.35	0.51	0.21	0.64	0.66	0.05
484	DNMT1	ENSG00000130816.14	0.14	0.21	0.47	4.00E-10	0.66	3.50E-20
484	PDLIM4	ENSG00000131435.12	0.03	0.86	0.47	1.00E-10	0.66	2.70E-21
484	RRAS2	ENSG00000133818.13	0.24	0.031	0.4	7.80E-06	0.66	3.40E-15
484	DSCC1	ENSG00000136982.5	0.08	0.87	0.34	0.2	0.66	0.0027
484	WARS	ENSG00000140105.17	0.31	0.0019	0.53	5.30E-10	0.66	6.20E-16
484	DOPEY2	ENSG00000142197.12	0.33	0.0027	0.48	2.70E-07	0.66	2.90E-14
484	TTC13	ENSG00000143643.12	0.05	0.87	0.58	2.80E-05	0.66	4.00E-07
484	SRFBP1	ENSG00000151304.5	0.08	0.79	0.39	0.013	0.66	9.10E-07
484	MARVELD2	ENSG00000152939.14	0.38	0.42	0.4	0.27	0.66	0.032
484	USP1	ENSG00000162607.12	0.25	0.035	0.43	8.50E-06	0.66	1.90E-13
484	DCBLD1	ENSG00000164465.18	0.31	0.001	0.37	1.40E-05	0.66	1.90E-17
484	MCM7	ENSG00000166508.17	0.13	0.38	0.48	3.60E-08	0.66	3.10E-15
484	GPRC5B	ENSG00000167191.11	0.41	5.40E-06	0.37	1.30E-05	0.66	5.00E-17
484	MLKL	ENSG00000168404.12	0.17	0.54	0.53	9.20E-04	0.66	7.80E-06
484	NECTIN3	ENSG00000177707.10	0.2	0.014	0.4	5.20E-10	0.66	1.60E-27
484	RAI14	ENSG00000039560.13	0.33	6.40E-06	0.39	3.10E-09	0.65	4.30E-25
484	PHLPP2	ENSG00000040199.18	0.23	0.13	0.47	2.70E-05	0.65	5.90E-10
484	RRP15	ENSG00000067533.5	0.25	0.084	0.41	2.10E-04	0.65	1.30E-10
484	ERCC2	ENSG00000104884.14	0.3	0.0058	0.27	0.0048	0.65	1.70E-14
484	TUBD1	ENSG00000108423.14	0.4	0.32	0.37	0.25	0.65	0.017
484	SLC38A1	ENSG00000111371.15	0.35	1.20E-06	0.42	6.60E-10	0.65	2.60E-24
484	ZMIZ2	ENSG00000122515.14	0.02	0.95	0.55	5.50E-08	0.65	2.10E-11
484	KIF21A	ENSG00000139116.18	0.49	0.24	0.41	0.24	0.65	0.032
484	USP53	ENSG00000145390.11	0.25	0.088	0.53	1.20E-06	0.65	3.90E-10
484	ADAM12	ENSG00000148848.14	0.35	0.005	0.08	0.58	0.65	4.60E-11
484	EPHB1	ENSG00000154928.16	0	1	0.26	0.39	0.65	0.0047
484	FAM86C1	ENSG00000158483.15	0.4	0.028	0.43	0.0068	0.65	4.20E-06
484	LRR1	ENSG00000165501.16	0.32	0.13	0.47	0.0036	0.65	1.00E-05
484	C5orf34	ENSG00000172244.8	0.02	0.98	0.58	0.069	0.65	0.027
484	LMNB2	ENSG00000176619.12	0.31	0.0022	0.4	4.40E-06	0.65	3.80E-15
484	NUP62	ENSG00000213024.11	0.18	0.018	0.53	3.20E-21	0.65	2.20E-32
484	PFKP	ENSG00000067057.16	0.4	1.30E-06	0.32	6.50E-05	0.64	2.30E-18
484	AURKA	ENSG00000087586.17	0.23	0.32	0.5	0.0015	0.64	1.50E-05
484	FARSB	ENSG00000116120.9	0.19	0.12	0.49	3.00E-08	0.64	3.90E-14
484	LDLR	ENSG00000130164.13	0.41	4.60E-06	0.21	0.027	0.64	4.80E-16
484	NIP7	ENSG00000132603.13	0.16	0.25	0.44	3.30E-06	0.64	5.70E-13
484	PCNA	ENSG00000132646.10	0.13	0.54	0.54	3.50E-06	0.64	1.10E-08
484	ABHD17C	ENSG00000136379.11	0.33	0.023	0.48	3.30E-05	0.64	4.50E-09
484	SPTBN5	ENSG00000137877.9	0.64	0.12	0.04	0.94	0.64	0.049
484	TAPBPL	ENSG00000139192.11	0.22	0.24	0.53	4.50E-05	0.64	1.60E-07
484	TMEM132	ENSG00000139364.10	-0.16	0.76	0.49	0.079	0.64	0.0096
484	KLF10	ENSG00000155090.14	0.18	0.14	0.45	2.80E-07	0.64	1.90E-14
484	SMAD3	ENSG00000166949.15	0.24	2.70E-07	0.58	1.20E-45	0.64	2.10E-55
484	RFWD3	ENSG00000168411.13	0.16	0.3	0.45	6.90E-06	0.64	8.20E-12
484	PFAS	ENSG00000178921.13	0.18	0.17	0.5	1.00E-08	0.64	3.70E-14
484	AC144831	ENSG00000261888.1	0.52	0.11	0.36	0.22	0.64	0.011
484	USP13	ENSG00000058056.8	0.18	0.21	0.33	0.002	0.63	3.20E-11
484	CYLD	ENSG00000083799.17	0.08	0.55	0.53	4.10E-14	0.63	9.80E-20
484	FAM234B	ENSG00000084444.13	0.34	0.11	0.32	0.082	0.63	3.00E-05
484	EZR	ENSG00000092820.17	0.35	0.0037	0.33	0.0024	0.63	1.30E-10
484	PIM2	ENSG00000102096.9	0.09	0.83	0.32	0.15	0.63	4.60E-04

484	IGDCC4	ENSG00000103742.11	-0.05	0.82	0.47	4.90E-06	0.63	1.40E-10
484	GRWD1	ENSG00000105447.12	0.24	0.046	0.46	9.50E-07	0.63	1.60E-12
484	DKK1	ENSG00000107984.9	0.34	0.0057	0.42	7.70E-05	0.63	1.80E-10
484	WNT5B	ENSG00000111186.12	0.32	0.0015	0.24	0.012	0.63	6.30E-15
484	RRP9	ENSG00000114767.6	0.29	0.11	0.5	3.60E-04	0.63	1.20E-06
484	FIGNL1	ENSG00000132436.11	0.08	0.77	0.46	5.50E-04	0.63	1.70E-07
484	POSTN	ENSG00000133110.14	0.15	0.11	0.56	3.30E-17	0.63	4.00E-22
484	LRRK1	ENSG00000154237.12	0.11	0.45	0.44	5.90E-07	0.63	1.60E-14
484	HEATR3	ENSG00000155393.12	0.16	0.42	0.43	6.80E-04	0.63	4.40E-08
484	ATF3	ENSG00000162772.16	0.21	0.48	0.34	0.11	0.63	4.40E-04
484	SFMBT1	ENSG00000163935.13	0.31	0.51	0.34	0.32	0.63	0.024
484	MICALL2	ENSG00000164877.18	-0.02	0.95	0.57	4.70E-08	0.63	6.90E-10
484	SAPCD2	ENSG00000186193.8	0.25	0.59	0.45	0.13	0.63	0.017
484	MZT1	ENSG00000204899.5	0.38	0.033	0.45	0.0028	0.63	4.80E-06
484	JPT2	ENSG00000206053.12	0.2	0.011	0.53	1.60E-17	0.63	4.10E-25
484	SMIM13	ENSG00000224531.5	0.21	0.11	0.45	1.70E-06	0.63	1.10E-12
484	POLR1A	ENSG00000068654.15	0.22	0.0056	0.36	3.00E-08	0.62	3.00E-24
484	SSH1	ENSG00000084112.14	0.35	2.80E-08	0.3	6.10E-07	0.62	3.60E-28
484	FOXM1	ENSG00000111206.12	0.29	0.13	0.19	0.27	0.62	3.20E-06
484	RGS4	ENSG00000117152.13	0.23	0.32	0.28	0.11	0.62	2.10E-05
484	EPHB2	ENSG00000133216.16	0.14	0.14	0.4	6.50E-10	0.62	3.80E-24
484	KRT7	ENSG00000135480.15	0.33	0.071	0.32	0.038	0.62	4.40E-06
484	FBXO45	ENSG00000174013.7	0.34	0.042	0.41	0.0037	0.62	9.20E-07
484	BDNF	ENSG00000176697.18	0.46	5.50E-04	0.28	0.033	0.62	3.60E-08
484	OXTR	ENSG00000180914.10	0.58	9.70E-04	0.28	0.13	0.62	3.90E-05
484	SYNM	ENSG00000182253.14	0.42	0.0077	0.02	0.91	0.62	7.80E-07
484	ATG7	ENSG00000197548.12	0.08	0.49	0.48	2.60E-13	0.62	6.60E-23
484	AC015712	ENSG00000259583.2	0.13	0.7	0.23	0.25	0.62	8.40E-05
484	AL136164	ENSG00000279312.1	-0.26	0.62	0.1	0.81	0.62	0.027
484	SPEN	ENSG00000065526.10	0.17	0.14	0.26	0.0036	0.61	2.90E-15
484	POLD3	ENSG00000077514.8	0.22	0.19	0.43	3.10E-04	0.61	2.70E-08
484	FAT1	ENSG00000083857.13	0.29	0.0035	0.32	2.10E-04	0.61	1.80E-14
484	NUP188	ENSG00000095319.14	0.18	0.08	0.44	8.50E-09	0.61	1.20E-16
484	SLC1A4	ENSG00000115902.10	0.36	0.14	0	0.99	0.61	4.90E-04
484	CHTF18	ENSG00000127586.16	0.24	0.32	0.44	0.0073	0.61	4.90E-05
484	RNF128	ENSG00000133135.13	0.03	0.95	0.43	0.054	0.61	0.0019
484	MYC	ENSG00000136997.17	0.06	0.75	0.32	0.0019	0.61	4.10E-11
484	SORL1	ENSG00000137642.12	0.12	0.79	0.41	0.098	0.61	0.0056
484	C9orf72	ENSG00000147894.14	0.23	0.39	0.51	0.0031	0.61	1.30E-04
484	CYP2U1	ENSG00000155016.17	0.25	0.17	0.49	1.40E-04	0.61	4.10E-07
484	SGO2	ENSG00000163535.17	0.23	0.41	0.34	0.09	0.61	4.00E-04
484	WDR43	ENSG00000163811.11	0.18	0.057	0.39	6.90E-08	0.61	8.80E-19
484	OSGIN2	ENSG00000164823.9	-0.03	0.91	0.23	0.072	0.61	3.70E-09
484	JCAD	ENSG00000165757.8	0.54	1.10E-07	0.24	0.025	0.61	3.50E-11
484	NIPA1	ENSG00000170113.15	0.17	0.38	0.34	0.012	0.61	1.10E-07
484	RIOX2	ENSG00000170854.17	0.16	0.1	0.56	1.10E-15	0.61	3.30E-19
484	SUSD5	ENSG00000173705.8	0.42	0.074	0.43	0.029	0.61	5.00E-04
484	DDX28	ENSG00000182810.6	0.36	0.0077	0.38	0.001	0.61	6.20E-09
484	SOCS3	ENSG00000184557.4	0.12	0.7	0.39	0.022	0.61	8.60E-05
484	TUBB4B	ENSG00000188229.5	0.33	0.0032	0.36	2.00E-04	0.61	2.00E-11
484	LIN52	ENSG00000205659.10	0.25	0.31	0.15	0.48	0.61	7.10E-05
484	HGH1	ENSG00000235173.6	0.28	0.027	0.38	3.00E-04	0.61	2.90E-10
484	ATP8B1	ENSG00000081923.11	0.37	0.01	0.19	0.19	0.6	9.70E-08
484	KIAA0391	ENSG00000100890.15	0.12	0.73	0.36	0.059	0.6	3.00E-04
484	SLC7A6	ENSG00000103064.13	0.13	0.32	0.35	3.60E-05	0.6	1.00E-14
484	NFKBIB	ENSG00000104825.16	0.17	0.34	0.46	7.50E-05	0.6	4.70E-08
484	EZH2	ENSG00000106462.10	0.24	0.22	0.36	0.013	0.6	2.40E-06
484	DPYSL3	ENSG00000113657.12	0.26	3.60E-06	0.28	6.80E-08	0.6	4.90E-37
484	SRM	ENSG00000116649.9	0.29	6.20E-04	0.4	5.50E-08	0.6	2.60E-18
484	PRDX6	ENSG00000117592.8	0.15	0.15	0.47	4.50E-10	0.6	5.30E-17
484	UTP20	ENSG00000120800.4	0.18	0.092	0.48	6.90E-11	0.6	5.60E-17
484	SPRYD7	ENSG00000123178.14	0.29	0.037	0.24	0.056	0.6	1.00E-08
484	C17orf53	ENSG00000125319.14	0.09	0.82	0.42	0.04	0.6	0.001
484	OPA3	ENSG00000125741.4	0.27	0.0031	0.42	3.10E-08	0.6	1.90E-17
484	SCLY	ENSG00000132330.16	-0.01	0.99	0.36	0.29	0.6	0.037
484	POPDC3	ENSG00000132429.9	0.13	0.54	0.51	3.10E-05	0.6	2.10E-07

484	YARS	ENSG00000134684.10	0.31	1.00E-04	0.25	0.001	0.6	3.30E-19
484	SPRY2	ENSG00000136158.11	0.28	0.058	0.26	0.045	0.6	5.70E-08
484	LYAR	ENSG00000145220.13	0.1	0.7	0.5	2.60E-04	0.6	3.20E-06
484	TONSL	ENSG00000160949.16	0.16	0.41	0.41	8.40E-04	0.6	1.40E-07
484	GPR85	ENSG00000164604.12	0.42	0.24	0.54	0.049	0.6	0.018
484	MSRB3	ENSG00000174099.10	0.44	6.00E-04	0.22	0.096	0.6	1.90E-08
484	PAWR	ENSG00000177425.10	0.19	0.19	0.31	0.0028	0.6	7.40E-11
484	GSAP	ENSG00000186088.15	0.13	0.78	0.53	0.03	0.6	0.0081
484	LRRC8B	ENSG00000197147.13	0.1	0.7	0.53	5.70E-05	0.6	1.70E-06
484	AC016831	ENSG00000226380.9	0.18	0.54	0.46	0.01	0.6	2.90E-04
484	AC105942	ENSG00000235501.5	0.22	0.51	0.33	0.14	0.6	0.0016
484	BCAR1	ENSG00000050820.16	0.28	4.90E-04	0.42	8.20E-10	0.59	2.40E-19
484	TM7SF3	ENSG00000064115.10	0.16	0.15	0.49	4.50E-10	0.59	4.10E-15
484	ISOC1	ENSG00000066583.11	0.06	0.77	0.5	1.60E-07	0.59	8.80E-11
484	PKM	ENSG00000067225.17	0.1	0.25	0.5	2.50E-19	0.59	4.10E-27
484	IFI35	ENSG00000068079.7	0.25	0.18	0.27	0.078	0.59	4.40E-06
484	DNAJC10	ENSG00000077232.17	0.16	0.0032	0.54	3.50E-39	0.59	1.00E-48
484	ODC1	ENSG00000115758.12	0.37	0.0031	0.2	0.1	0.59	4.80E-09
484	ARHGAP22	ENSG00000128805.14	0.15	0.25	0.56	2.20E-11	0.59	2.60E-13
484	TOMM40	ENSG00000130204.12	0.21	0.062	0.43	5.90E-07	0.59	9.80E-13
484	TMEM106	ENSG00000134291.11	0.26	0.15	0.43	0.0018	0.59	3.40E-06
484	LMO7	ENSG00000136153.19	0.3	0.027	0.25	0.039	0.59	1.00E-08
484	CRIM1	ENSG00000150938.9	0.44	1.20E-06	0.22	0.02	0.59	2.20E-13
484	SLC9B2	ENSG00000164038.14	0.23	0.14	0.47	3.80E-05	0.59	2.30E-08
484	OTUD4	ENSG00000164164.15	0.13	0.2	0.43	2.70E-10	0.59	2.30E-19
484	SMCO4	ENSG00000166002.6	0.22	0.54	0.44	0.057	0.59	0.0041
484	SH3RF3	ENSG00000172985.10	0.26	0.0012	0.38	2.70E-08	0.59	1.20E-19
484	PDE12	ENSG00000174840.8	0.16	0.17	0.45	6.90E-08	0.59	5.60E-14
484	RPP25	ENSG00000178718.6	0.21	0.43	0.42	0.019	0.59	2.10E-04
484	ARSJ	ENSG00000180801.13	0.37	7.30E-04	0.36	2.60E-04	0.59	9.60E-11
484	ARHGEF28	ENSG00000214944.9	0.22	0.21	0.37	0.0041	0.59	3.50E-07
484	SNHG3	ENSG00000242125.3	0.31	0.0012	0.37	1.40E-05	0.59	6.00E-14
484	PGAM5	ENSG00000247077.6	0.16	0.33	0.47	7.80E-06	0.59	4.40E-09
484	EBLN2	ENSG00000255423.1	0.66	0.061	-0.08	0.86	0.59	0.044
484	HMBS	ENSG00000256269.7	0.21	0.27	0.33	0.018	0.59	1.70E-06
484	AC243964	ENSG00000279095.1	0.42	0.044	0.36	0.054	0.59	2.30E-04
66	MEOX1	ENSG00000005102.12	0.47	0.4	1.45	0.0093	0.71	0.11
66	CACNA1G	ENSG00000006283.17	-0.35	0.36	0.64	0.011	0.23	0.44
66	GAL	ENSG00000069482.6	-0.22	0.083	0.67	2.80E-13	0.14	0.2
66	KCNK2	ENSG00000082482.13	0	0.99	0.91	2.70E-14	0.51	4.60E-05
66	ABCB1	ENSG00000085563.14	0.46	0.29	1.04	3.60E-04	0.58	0.063
66	ACHE	ENSG00000087085.13	-0.29	0.49	0.63	0.01	0.11	0.75
66	CYTH4	ENSG00000100055.20	0.34	0.56	0.97	0.0044	0.68	0.052
66	LAMA1	ENSG00000101680.14	0.19	0.28	0.62	6.20E-08	0.42	3.00E-04
66	TFPI2	ENSG00000105825.11	-0.48	0.044	0.7	2.90E-04	0.1	0.71
66	RARRES2	ENSG00000106538.9	0.18	0.4	0.62	2.40E-06	0.44	9.90E-04
66	TMEM176	ENSG00000106565.17	0.06	0.93	0.67	0.026	0.35	0.29
66	SLC16A6	ENSG00000108932.11	-0.23	0.45	0.72	6.10E-05	0.22	0.31
66	HES1	ENSG00000114315.3	0.19	0.81	0.89	0.028	0.69	0.085
66	CD58	ENSG00000116815.15	0.14	0.69	0.65	2.60E-04	0.44	0.017
66	IL13RA2	ENSG00000123496.7	-0.04	0.97	1.28	2.70E-04	0.55	0.17
66	RAMP1	ENSG00000132329.10	0.24	0.12	0.64	9.90E-09	0.49	1.00E-05
66	ADAMTS8	ENSG00000134917.9	0.55	0.097	0.81	0.0017	0.48	0.076
66	ITGA11	ENSG00000137809.16	0.19	0.26	0.68	3.10E-10	0.49	7.20E-06
66	PAQR5	ENSG00000137819.13	-0.27	0.064	0.84	1.80E-17	0.36	8.50E-04
66	HAPLN3	ENSG00000140511.11	0.16	0.59	0.7	8.00E-06	0.52	0.001
66	INA	ENSG00000148798.10	0.3	0.22	0.59	7.90E-04	0.48	0.0064
66	AKAP6	ENSG00000151320.10	-0.46	0.26	0.7	0.013	0.24	0.48
66	FLI1	ENSG00000151702.16	0.22	0.69	0.62	0.044	0.4	0.21
66	GABRA2	ENSG00000151834.15	-0.16	0.11	0.83	5.60E-36	0.56	1.50E-16
66	ADAMTSL3	ENSG00000156218.12	-0.4	2	1.13	0.017	0.79	0.1
66	ABHD3	ENSG00000158201.9	0.15	0.69	0.65	9.80E-04	0.58	0.0031
66	RNF207	ENSG00000158286.12	-0.2	0.64	0.69	0.003	0.54	0.021
66	PTGIR	ENSG00000160013.8	-0.03	0.93	0.87	6.40E-09	0.55	3.70E-04
66	AK4	ENSG00000162433.14	0.08	0.89	0.6	0.026	0.53	0.045
66	NUP35	ENSG00000163002.12	0.01	0.98	0.62	6.60E-05	0.51	0.0011

66	CDCP1	ENSG00000163814.7	0.03	0.85	0.6	1.80E-15	0.55	2.60E-13
66	ITGA2	ENSG00000164171.10	-0.18	0.1	0.7	4.90E-19	0.51	1.20E-10
66	SHH	ENSG00000164690.7	0.06	2	1.06	0.014	0.77	0.08
66	EVA1C	ENSG00000166979.12	-0.01	0.99	0.59	0.029	0.56	0.03
66	FILIP1L	ENSG00000168386.18	0.15	0.29	0.64	1.70E-12	0.52	9.70E-09
66	FAM84B	ENSG00000168672.3	-0.05	0.91	0.72	7.60E-05	0.47	0.013
66	PARM1	ENSG00000169116.11	0.42	0.33	0.79	0.0086	0.48	0.13
66	CXCL10	ENSG00000169245.5	0.05	0.96	1.07	0.025	0.75	0.1
66	MBOAT1	ENSG00000172197.10	-0.07	0.9	0.62	0.012	0.4	0.12
66	SCAI	ENSG00000173611.17	-0.23	0.22	0.59	2.00E-06	0.38	0.0025
66	SPHK1	ENSG00000176170.13	0.2	0.25	0.8	1.60E-12	0.56	1.30E-06
66	ARID3B	ENSG00000179361.17	-0.07	0.84	0.7	4.90E-06	0.42	0.01
66	FAM19A3	ENSG00000184599.13	0.56	2	1.03	0.029	0.3	0.6
66	EMID1	ENSG00000186998.15	-0.48	0.24	0.66	0.029	0.13	0.74
66	TMPPE	ENSG00000188167.8	0.55	0.24	0.98	0.0033	0.34	0.39
66	HES4	ENSG00000188290.10	0.43	0.26	1.02	7.60E-05	0.24	0.47
66	ADAMTSL2	ENSG00000197859.9	0.72	2	1.49	0.014	0.81	0.092
66	EGFL6	ENSG00000198759.11	0.72	2	1.38	0.0056	0.45	0.22
66	FBXO48	ENSG00000204923.3	-0.13	0.84	0.71	0.02	0.48	0.13
66	TMEM17	ENSG00000205269.5	0.27	0.42	0.64	0.0035	0.53	0.015
66	ZNF90	ENSG00000213988.10	-0.21	0.75	0.69	0.042	0.53	0.12
66	AL390719	ENSG00000217801.9	-0.12	0.58	0.69	3.90E-10	0.52	2.70E-06
66	AP001476	ENSG00000224413.1	0.49	0.39	1.01	0.0081	0.63	0.11
66	PSME2P2	ENSG00000225131.2	0.06	0.95	0.91	0.037	0.19	0.73
66	LINC0062	ENSG00000226067.6	0.06	0.93	0.59	0.03	0.28	0.36
66	CBR3-AS1	ENSG00000236830.6	0.32	0.48	0.61	0.042	0.38	0.23
66	AC003075	ENSG00000237773.5	0.12	2	0.8	0.046	0.42	0.34
66	PRR34-AS	ENSG00000241990.5	0.35	0.59	0.84	0.037	0.64	0.11
66	AC098679	ENSG00000248429.5	0.09	2	0.91	0.017	0.58	0.15
66	AC114284	ENSG00000248927.1	-0.07	0.9	0.59	0.016	0.28	0.3
66	C17orf49	ENSG00000258315.5	-0.21	0.75	0.89	0.0072	0.5	0.16
66	AC104072	ENSG00000260878.1	-0.54	0.27	0.8	0.017	0.22	0.6
66	RASSF5	ENSG00000266094.7	0.28	0.43	0.77	6.10E-04	0.27	0.32
66	AC112220	ENSG00000271020.1	0.26	0.67	0.82	0.016	0.38	0.31
66	AL096865	ENSG00000271857.1	0.09	0.92	0.96	0.017	0.74	0.064
66	AC026356	ENSG00000274964.1	0.18	0.79	0.71	0.041	0.44	0.23
409	SLC7A2	ENSG00000003989.17	0.09	0.44	0.95	8.50E-49	1.37	1.60E-105
409	MCUB	ENSG00000005059.15	0.25	0.24	0.59	4.00E-05	0.64	3.20E-06
409	MAP3K14	ENSG00000006062.14	0.01	0.99	1.19	7.20E-23	1.2	7.70E-24
409	TMEM132	ENSG00000006118.14	0.18	0.2	1.19	4.50E-40	1.43	1.20E-58
409	CX3CL1	ENSG00000006210.6	0.36	0.58	2.32	6.40E-12	2.81	8.80E-18
409	MAP3K9	ENSG00000006432.15	0.7	2	1.37	0.003	1.14	0.011
409	E2F2	ENSG00000007968.6	0.85	0.073	1.1	0.0043	2.06	1.10E-09
409	ANLN	ENSG00000011426.10	0.54	0.03	0.99	5.00E-07	1.4	7.80E-14
409	BRCA1	ENSG00000012048.20	0.31	0.28	0.8	2.90E-05	1.32	3.30E-14
409	TACC3	ENSG00000013810.18	0.47	0.0092	0.77	1.80E-07	1.21	1.50E-18
409	POLA2	ENSG00000014138.8	0.27	0.2	0.6	3.70E-05	0.98	2.20E-13
409	CCDC88C	ENSG00000015133.18	0.42	0.45	0.97	0.0066	1.08	0.0011
409	CD74	ENSG00000019582.14	0.08	0.84	0.64	4.60E-04	0.8	2.50E-06
409	TYMP	ENSG00000025708.13	0.34	0.098	1.64	1.40E-35	1.63	3.20E-35
409	FAS	ENSG00000026103.21	0.14	0.38	0.64	1.10E-11	0.63	8.50E-12
409	TNFRSF1B	ENSG00000028137.18	0.09	0.43	0.97	1.10E-52	1.06	1.60E-63
409	RIPOR3	ENSG00000042062.11	0.08	0.65	0.68	1.20E-16	0.65	1.10E-15
409	TNFRSF9	ENSG00000049249.8	0.47	0.45	1.86	9.60E-04	1.39	0.0065
409	NFE2L3	ENSG00000050344.8	0.49	8.60E-11	2.09	1.20E-235	2.09	1.30E-237
409	POLQ	ENSG00000051341.13	0.49	0.19	1.3	2.40E-07	1.81	1.40E-14
409	RELT	ENSG00000054967.12	0.03	0.94	0.63	5.20E-04	0.77	5.10E-06
409	CYFIP2	ENSG00000055163.19	0.08	0.73	0.68	1.80E-10	0.6	1.60E-08
409	TRAF1	ENSG00000056558.10	0.07	0.81	1.28	2.40E-27	1.58	1.60E-42
409	DCBLD2	ENSG00000057019.15	0.09	0.34	0.93	5.00E-68	1.12	9.60E-100
409	LAMC2	ENSG00000058085.14	0.12	0.76	1.73	5.60E-26	1.81	9.80E-29
409	NDC1	ENSG00000058804.11	0.21	0.23	0.67	5.90E-09	0.72	7.30E-11
409	DGAT2	ENSG00000062282.14	0.18	0.6	1.17	3.00E-12	1.36	3.00E-17
409	CHI3L2	ENSG00000064886.13	0.71	0.19	2	1.00E-07	2.61	4.60E-13
409	SBNO2	ENSG00000064932.15	0.23	0.0074	0.62	8.40E-20	0.81	5.90E-34

409	ASPM	ENSG00000066279.17	0.43	0.13	0.97	1.60E-06	1.27	4.60E-11
409	NAV3	ENSG00000067798.14	0.54	9.00E-05	1.33	8.20E-33	1.85	4.10E-65
409	BCL3	ENSG00000069399.14	0.48	8.20E-05	0.92	6.60E-19	1.11	2.10E-28
409	TRIP13	ENSG00000071539.13	0.46	0.055	0.88	1.30E-06	1.26	2.10E-13
409	HMMR	ENSG00000072571.19	0.1	0.84	0.89	2.30E-05	1.13	1.30E-08
409	MCM2	ENSG00000073111.13	0.41	0.011	0.74	1.70E-08	1.12	2.10E-19
409	GTSE1	ENSG00000075218.18	0.51	0.082	1.08	8.60E-07	1.51	1.90E-13
409	WDR62	ENSG00000075702.16	0.42	0.22	1.19	1.50E-07	1.41	7.10E-11
409	SPAG5	ENSG00000076382.16	0.56	0.082	0.94	1.50E-04	1.33	8.50E-09
409	NFKB2	ENSG00000077150.18	0.26	0.0073	1.86	1.00E-161	2.06	9.50E-200
409	UBE2T	ENSG00000077152.9	0.28	0.35	0.6	0.0035	0.84	5.80E-06
409	PPP1R12B	ENSG00000077157.21	0.31	0.0083	0.68	1.90E-13	1	1.00E-29
409	SYNJ2	ENSG00000078269.14	0.1	0.18	0.66	4.70E-40	0.8	3.40E-60
409	MOXD1	ENSG00000079931.14	0.27	0.037	0.94	4.60E-24	0.98	1.70E-26
409	KCNN2	ENSG00000080709.14	0.32	0.58	1.18	2.60E-04	1.14	2.90E-04
409	RBL1	ENSG00000080839.11	0.21	0.26	0.62	5.90E-07	0.91	2.80E-15
409	COL19A1	ENSG00000082293.12	-0.49	0.3	0.95	0.0012	1.14	2.80E-05
409	COBLL1	ENSG00000082438.15	0.87	0.065	1.42	1.60E-04	1.7	1.70E-06
409	CD82	ENSG00000085117.11	0.54	2.90E-11	1.54	2.20E-100	1.85	2.50E-147
409	RAD54L	ENSG00000085999.11	0.46	0.19	1.29	3.70E-08	1.49	3.10E-11
409	RGS1	ENSG00000090104.11	-0.02	0.99	1.76	0.0058	1.65	0.0059
409	ICAM1	ENSG00000090339.8	0.35	0.05	1.68	7.20E-45	1.55	5.90E-38
409	KIF4A	ENSG00000090889.11	0.49	0.031	0.82	5.60E-06	1.32	3.70E-15
409	PUS7	ENSG00000091127.13	0.34	0.034	0.75	2.80E-10	0.91	8.20E-16
409	ORC6	ENSG00000091651.8	0.33	0.26	0.81	4.80E-05	1.15	3.70E-10
409	WDR76	ENSG00000092470.11	0.32	0.14	0.68	1.20E-05	0.82	2.30E-08
409	CLSPN	ENSG00000092853.13	0.5	0.017	0.79	3.90E-06	1.34	1.30E-17
409	CDC7	ENSG00000097046.12	0.24	0.37	0.6	4.60E-04	0.77	1.10E-06
409	PLA2G3	ENSG00000100078.3	0.85	0.059	1.09	0.0031	1.37	5.50E-05
409	CENPM	ENSG00000100162.14	0.56	0.11	0.81	0.0031	0.99	9.70E-05
409	MCM5	ENSG00000100297.15	0.27	0.1	0.68	1.60E-08	0.9	6.40E-15
409	APOL1	ENSG00000100342.20	0.11	0.82	0.96	5.90E-06	1.08	8.80E-08
409	POLE2	ENSG00000100479.12	0.15	0.76	0.94	1.70E-05	1.24	1.50E-09
409	CEP128	ENSG00000100629.16	0.28	0.43	0.6	0.01	0.86	4.50E-05
409	PAPLN	ENSG00000100767.15	0.22	0.48	1.34	7.30E-15	1.32	8.80E-15
409	NFKBIA	ENSG00000100906.10	0.34	0.0036	2.14	7.30E-130	1.82	6.60E-93
409	PSME2	ENSG00000100911.15	0.1	0.45	0.72	4.00E-22	0.73	1.10E-23
409	MMP9	ENSG00000100985.7	0.26	0.56	2.62	6.90E-06	1.91	1.10E-04
409	GINS1	ENSG00000101003.9	0.51	0.048	0.82	4.80E-05	1.19	1.50E-10
409	CENPI	ENSG00000102384.13	0.39	0.19	0.59	0.0075	1	3.40E-07
409	HTR2A	ENSG00000102468.10	0.27	0.4	0.88	4.40E-06	1.35	1.30E-14
409	DHODH	ENSG00000102967.11	0.47	0.056	0.81	1.60E-05	1.05	2.00E-09
409	LACTB	ENSG00000103642.11	0.19	0.13	0.7	2.40E-16	0.83	1.20E-23
409	OIP5	ENSG00000104147.8	0.33	0.61	0.87	0.023	1.43	1.90E-05
409	TNFRSF10A	ENSG00000104689.9	0.17	0.43	0.74	1.80E-09	0.84	1.50E-12
409	RELB	ENSG00000104856.13	0.45	4.00E-06	1.95	3.50E-143	1.98	2.20E-149
409	IL27RA	ENSG00000104998.3	0.14	0.63	2.33	2.60E-66	1.93	6.90E-45
409	PLA2G4C	ENSG00000105499.13	0.14	0.76	2.81	2.90E-57	2.83	1.50E-58
409	JAK3	ENSG00000105639.18	0.07	0.84	0.95	5.50E-09	0.62	2.10E-04
409	NAMPT	ENSG00000105835.11	0.09	0.54	0.73	9.50E-23	0.97	1.20E-40
409	DNAH11	ENSG00000105877.17	0.25	0.67	1.57	6.30E-08	1.87	1.70E-11
409	SERPINE1	ENSG00000106366.8	0.47	0.002	0.61	3.70E-06	1.23	1.80E-23
409	SYNGR2	ENSG00000108639.7	0.1	0.51	0.63	3.30E-13	0.61	8.30E-13
409	CCL2	ENSG00000108691.9	0.49	0.43	1	0.024	1.95	1.60E-06
409	CNTNAP1	ENSG00000108797.11	0.31	0.011	0.78	6.50E-17	1.03	7.00E-30
409	ABCC3	ENSG00000108846.15	0.01	0.98	0.65	4.30E-06	0.63	5.80E-06
409	NFKB1	ENSG00000109320.11	0.28	0.0024	0.71	6.40E-23	0.75	3.60E-26
409	NEIL3	ENSG00000109674.3	0.56	0.31	1.12	0.0036	1.63	3.20E-06
409	GLRB	ENSG00000109738.10	0.3	0.25	0.77	4.40E-06	1.28	3.50E-17
409	KLHL5	ENSG00000109790.16	0.23	0.0022	0.68	6.50E-30	0.63	1.50E-25
409	PANX1	ENSG00000110218.8	0.26	8.30E-07	0.65	4.20E-47	0.77	5.50E-68
409	GALNT18	ENSG00000110328.5	-0.23	0.67	1.62	9.90E-11	1.34	8.10E-08
409	SLC15A3	ENSG00000110446.10	0.31	0.25	0.64	6.90E-04	0.79	7.20E-06
409	SLC35F2	ENSG00000110660.14	0.28	0.043	0.96	1.50E-22	1.11	5.90E-31
409	RAD51AP1	ENSG00000111247.14	0.33	0.4	1.14	1.20E-06	1.48	3.00E-11
409	OAS3	ENSG00000111331.12	0.28	0.49	0.65	0.012	0.96	3.70E-05

409	MGP	ENSG00000111341.9	0.11	0.84	0.89	4.90E-04	0.64	0.013
409	ST8SIA1	ENSG00000111728.10	0.43	0.0092	0.84	1.40E-10	0.7	8.70E-08
409	ADTRP	ENSG00000111863.12	0.65	0.19	1.06	0.0044	1.26	2.50E-04
409	SOD2	ENSG00000112096.16	0.43	0.027	0.9	2.60E-09	1.69	2.40E-32
409	MCM3	ENSG00000112118.18	0.27	0.1	0.65	3.70E-08	1	3.90E-19
409	RAB23	ENSG00000112210.11	0.29	6.20E-04	0.64	4.70E-20	0.68	5.50E-23
409	CRYBG1	ENSG00000112297.14	0.43	1.00E-04	0.78	4.70E-17	1.07	4.70E-33
409	VNN1	ENSG00000112299.7	0.39	2	1.83	0.0037	2.28	4.80E-04
409	TTK	ENSG00000112742.9	0.52	0.085	1.15	2.10E-07	1.45	5.60E-12
409	LMNB1	ENSG00000113368.11	0.47	0.068	0.78	8.70E-05	1.17	2.40E-10
409	WWC1	ENSG00000113645.14	0.33	0.22	1.14	5.10E-11	2.05	1.10E-38
409	CDX1	ENSG00000113722.16	0.52	0.34	1.26	5.00E-04	1.88	1.50E-08
409	ECT2	ENSG00000114346.13	0.09	0.75	0.62	1.10E-05	0.76	1.30E-08
409	C3orf52	ENSG00000114529.12	0.12	0.83	0.77	0.0028	1.53	3.00E-12
409	IFIH1	ENSG00000115267.5	0.42	0.23	1.23	5.90E-08	1.4	1.10E-10
409	KYNU	ENSG00000115919.14	0	1	1.28	2.90E-09	1.47	1.50E-12
409	PNO1	ENSG00000115946.7	0.47	2.20E-04	0.75	6.40E-12	0.87	2.90E-16
409	PARD3B	ENSG00000116117.17	0.29	0.056	0.83	2.00E-14	1.16	4.70E-29
409	CDC20	ENSG00000117399.13	0.51	0.057	0.73	6.40E-04	1.18	1.90E-09
409	NEK2	ENSG00000117650.12	0.61	0.12	0.89	0.0033	1.2	1.70E-05
409	CENPF	ENSG00000117724.12	0.38	0.14	0.68	3.10E-04	1.13	8.10E-11
409	CD3EAP	ENSG00000117877.10	0.52	0.0082	0.83	1.60E-07	1.19	1.10E-15
409	KIF14	ENSG00000118193.11	0.26	0.53	0.99	3.10E-05	1.44	4.70E-11
409	TNFAIP3	ENSG00000118503.14	0.26	0.45	1.95	3.20E-25	1.87	2.00E-23
409	TJP2	ENSG00000119139.17	0.33	1.80E-04	1.21	4.90E-66	1.36	1.70E-83
409	GPR68	ENSG00000119714.10	0.09	0.88	1.57	4.00E-11	2.04	1.10E-19
409	HELLS	ENSG00000119969.14	0.26	0.18	0.81	4.30E-10	0.88	2.60E-12
409	MOB3B	ENSG00000120162.9	-0.04	0.94	2.23	2.10E-45	2.29	1.40E-48
409	CD274	ENSG00000120217.13	0.47	0.056	0.81	1.80E-05	1.33	3.40E-15
409	CENPL	ENSG00000120334.15	0.34	0.081	0.59	5.50E-05	0.81	3.10E-09
409	TNFSF18	ENSG00000120337.8	0.59	0.18	1.6	7.10E-08	1.55	9.10E-08
409	MASTL	ENSG00000120539.14	0.37	0.021	0.91	3.20E-14	1.19	3.80E-25
409	SOCS2	ENSG00000120833.13	0.09	0.59	0.69	1.40E-15	0.61	1.30E-12
409	KIF18A	ENSG00000121621.6	0.39	0.26	1	1.10E-05	1.27	3.00E-09
409	PTGFR	ENSG00000122420.9	-0.23	0.35	0.97	3.80E-12	0.84	1.20E-09
409	PLAU	ENSG00000122861.15	0.24	0.035	0.7	5.80E-16	0.9	6.30E-27
409	ZWINT	ENSG00000122952.16	0.44	0.17	0.89	1.30E-04	1.2	4.40E-08
409	BHLHE41	ENSG00000123095.5	0.44	0.18	1.39	2.30E-10	1.62	1.10E-14
409	CENPK	ENSG00000123219.12	0.22	0.54	0.78	1.80E-04	0.98	4.30E-07
409	HJURP	ENSG00000123485.11	0.6	0.09	1.11	3.70E-05	1.57	5.50E-10
409	BTN2A2	ENSG00000124508.16	-0.04	0.89	0.6	1.80E-06	0.6	7.30E-07
409	IRF1	ENSG00000125347.13	0.09	0.63	1.01	1.20E-27	1.01	3.80E-28
409	TMEM255/	ENSG00000125355.15	0.54	0.32	1.2	9.90E-04	1.64	1.10E-06
409	BMP2	ENSG00000125845.6	0.11	0.72	0.84	9.00E-09	0.63	1.90E-05
409	GDF5	ENSG00000125965.8	0.63	0.098	0.75	0.016	1.36	6.70E-07
409	DLGAP5	ENSG00000126787.12	0.45	0.076	0.8	4.20E-05	1.11	9.80E-10
409	HIVEP3	ENSG00000127124.14	0.54	8.00E-04	0.85	7.10E-10	1.47	6.00E-31
409	TRAF2	ENSG00000127191.17	0.01	0.98	0.92	8.20E-24	0.93	5.30E-25
409	AUNIP	ENSG00000127423.10	0.46	0.54	1.27	0.0058	1.39	0.0014
409	F2RL3	ENSG00000127533.3	0.53	0.35	0.86	0.037	0.91	0.017
409	SDF2L1	ENSG00000128228.4	0.24	0.22	0.65	7.20E-07	0.91	1.70E-13
409	POM121L	ENSG00000128262.8	-0.07	0.91	1.07	4.10E-06	1.08	1.50E-06
409	APOL3	ENSG00000128284.19	0.33	0.33	1	1.80E-06	1.06	1.40E-07
409	LIF	ENSG00000128342.4	0.32	6.30E-04	0.97	8.00E-38	1.4	2.10E-81
409	RIBC2	ENSG00000128408.8	0.66	2	1.3	0.014	1.32	0.009
409	KRT17	ENSG00000128422.15	0.14	0.88	1.29	0.0024	0.88	0.043
409	STRIP2	ENSG00000128578.9	0.38	0.5	1.31	5.50E-05	1.39	8.80E-06
409	E2F8	ENSG00000129173.12	0.19	0.81	1.13	0.0043	1.43	8.80E-05
409	PIMREG	ENSG00000129195.15	0.39	0.28	0.89	2.90E-04	1.31	6.00E-09
409	RHBDF2	ENSG00000129667.12	0.29	0.067	1.53	1.00E-46	1.59	3.40E-51
409	SAMD10	ENSG00000130590.13	0.49	0.043	0.97	1.40E-07	1.05	2.70E-09
409	GINS2	ENSG00000131153.8	0.52	0.023	0.8	1.20E-05	1.22	5.40E-13
409	TRAF3	ENSG00000131323.14	0.38	4.50E-07	1.42	1.70E-110	1.64	3.90E-150
409	HAUS8	ENSG00000131351.14	0.23	0.53	0.85	6.40E-05	0.91	7.70E-06
409	RFTN1	ENSG00000131378.13	0.35	1.20E-04	1.28	8.00E-69	1.47	4.30E-91
409	ZSWIM4	ENSG00000132003.9	0.43	0.0011	1.18	3.40E-30	1.37	4.10E-41

409	TRIM47	ENSG00000132481.6	0.33	2.50E-04	1.96	8.30E-176	1.97	2.40E-178
409	XAF1	ENSG00000132530.16	0.42	0.27	0.84	0.0016	1.07	1.20E-05
409	CHI3L1	ENSG00000133048.12	0.06	0.96	1.49	0.0031	1.09	0.032
409	DCLK1	ENSG00000133083.14	0.39	0.44	2.34	2.80E-17	2.35	1.10E-17
409	RFC3	ENSG00000133119.12	0.5	0.01	0.79	8.30E-07	1.09	3.00E-13
409	RARRES3	ENSG00000133321.10	0.47	0.16	0.8	0.0011	1.03	5.00E-06
409	E2F5	ENSG00000133740.10	0.34	0.22	0.6	0.0021	0.75	3.40E-05
409	MICAL2	ENSG00000133816.13	0.49	6.30E-08	0.86	7.30E-26	1.27	1.30E-57
409	TTC9	ENSG00000133985.2	0.54	0.14	2.72	8.10E-33	2.88	2.30E-37
409	CCNB1	ENSG00000134057.14	0.37	0.17	0.63	0.0016	0.84	6.10E-06
409	IRAK2	ENSG00000134070.4	0.53	6.90E-04	1.21	1.10E-22	1.37	3.20E-30
409	BHLHE40	ENSG00000134107.4	0.37	0.013	0.72	1.20E-09	1.11	1.00E-22
409	IL6ST	ENSG00000134352.19	0.09	0.43	0.62	6.70E-19	0.63	3.30E-20
409	IL15RA	ENSG00000134470.20	0.33	0.44	2.05	1.20E-19	2.37	3.40E-27
409	CDCA8	ENSG00000134690.10	0.39	0.25	1.1	8.00E-07	1.66	1.20E-15
409	RFK	ENSG00000135002.11	0.33	0.018	0.69	3.30E-10	0.66	6.50E-10
409	ADAM19	ENSG00000135074.15	0.27	0.0076	0.69	8.40E-19	0.65	1.60E-17
409	EHF	ENSG00000135373.12	0.77	0.17	1.14	0.012	3.6	3.80E-17
409	ESPL1	ENSG00000135476.11	0.39	0.25	0.94	3.10E-05	1.29	6.00E-10
409	MAP7	ENSG00000135525.18	0.42	0.49	1.01	0.0086	0.84	0.027
409	DYSF	ENSG00000135636.13	0.6	0.46	1.42	0.017	1.16	0.042
409	CPM	ENSG00000135678.11	-0.09	0.77	1.09	3.20E-17	0.95	1.80E-13
409	URB2	ENSG00000135763.9	0.32	0.07	0.7	5.90E-08	0.96	7.10E-15
409	SERPINE2	ENSG00000135919.12	0.27	0.013	1.01	8.30E-35	1.14	3.20E-45
409	ARHGEF4	ENSG00000136002.18	0.33	0.43	0.65	0.02	0.79	0.0021
409	DRAM1	ENSG00000136048.13	0.14	0.09	0.66	6.00E-31	0.6	8.30E-26
409	TNS3	ENSG00000136205.16	0.37	2.20E-09	0.81	3.90E-49	1.1	1.60E-92
409	ZFHX2	ENSG00000136367.13	0.21	0.76	1.27	1.20E-04	1.28	5.80E-05
409	LIMD2	ENSG00000136490.8	0.15	0.23	0.6	3.30E-14	0.7	9.10E-20
409	SLC31A2	ENSG00000136867.10	-0.12	0.77	0.68	4.70E-04	0.7	2.00E-04
409	IL33	ENSG00000137033.11	0.23	0.69	2.59	8.60E-27	2.77	3.90E-31
409	ALDH1B1	ENSG00000137124.7	0.38	0.0027	0.59	1.90E-08	0.92	1.60E-20
409	SLC22A23	ENSG00000137266.14	-0.55	0.44	1.4	0.0012	1.39	8.30E-04
409	TCF19	ENSG00000137310.11	0.36	0.085	0.81	1.50E-07	1.13	7.10E-15
409	TPMT	ENSG00000137364.4	0	1	0.72	3.00E-13	0.81	1.50E-17
409	TLR2	ENSG00000137462.6	-0.06	0.83	0.72	7.00E-10	0.7	6.60E-10
409	SQOR	ENSG00000137767.13	-0.08	0.79	0.78	2.30E-08	0.59	2.60E-05
409	NUSAP1	ENSG00000137804.12	0.39	0.14	0.8	3.60E-05	1.28	8.80E-13
409	KIF23	ENSG00000137807.13	0.58	0.016	0.94	1.20E-06	1.35	1.60E-13
409	KNL1	ENSG00000137812.19	0.55	0.033	0.94	3.60E-06	1.28	2.40E-11
409	CLCA2	ENSG00000137975.7	0.15	0.79	0.75	0.01	0.65	0.024
409	KHK	ENSG00000138030.12	0.28	0.63	0.7	0.048	0.77	0.018
409	CH25H	ENSG00000138135.6	1.31	2	2.85	6.90E-08	2.15	2.00E-05
409	CEP55	ENSG00000138180.15	0.41	0.31	0.85	0.0023	1.26	8.80E-07
409	KIF20B	ENSG00000138182.14	0.22	0.39	0.6	2.10E-04	0.97	5.10E-11
409	BARD1	ENSG00000138376.10	0.41	0.088	0.6	0.0013	1.09	1.70E-11
409	ITGAV	ENSG00000138448.11	0.13	0.22	1.37	2.30E-88	1.36	2.40E-88
409	FGF2	ENSG00000138685.13	0.16	0.12	0.67	1.70E-21	0.78	1.80E-29
409	CENPE	ENSG00000138778.11	0.42	0.25	0.85	9.20E-04	1.15	1.20E-06
409	BRCA2	ENSG00000139618.14	0.31	0.26	0.86	2.30E-06	1.22	5.60E-13
409	FRMD6	ENSG00000139926.15	0.3	7.00E-04	0.82	9.40E-30	1.19	2.10E-63
409	ABHD2	ENSG00000140526.17	0.13	0.096	0.74	1.70E-42	0.73	2.20E-41
409	CDH11	ENSG00000140937.13	0.18	0.0093	0.78	6.70E-48	0.64	5.30E-33
409	TAF4B	ENSG00000141384.12	0.4	0.25	1.44	1.60E-11	1.5	4.20E-13
409	GATA6	ENSG00000141448.8	0.52	5.50E-04	0.95	1.60E-14	1.36	5.10E-31
409	PMAIP1	ENSG00000141682.11	0.36	0.0061	1.23	1.10E-35	1.5	2.50E-54
409	PLK4	ENSG00000142731.10	0.21	0.56	0.92	3.20E-06	1.28	4.90E-12
409	KIF2C	ENSG00000142945.12	0.54	0.02	0.8	2.80E-05	1.4	8.40E-16
409	UCK2	ENSG00000143179.14	0.46	1.20E-06	0.79	2.30E-21	0.99	9.60E-35
409	NUF2	ENSG00000143228.12	0.51	0.21	1.24	8.60E-06	1.6	1.30E-09
409	DUSP10	ENSG00000143507.17	0.55	1.20E-04	0.62	1.50E-06	1.15	1.90E-22
409	CHAC2	ENSG00000143942.4	0.52	0.28	0.73	0.044	1.23	7.10E-05
409	CDCA7	ENSG00000144354.13	0.33	0.14	0.87	2.50E-08	1.32	4.00E-19
409	FANCD2	ENSG00000144554.10	0.53	0.028	1	9.90E-08	1.5	1.00E-17
409	4-Mar	ENSG00000144583.4	0.36	0.14	0.99	6.40E-09	1.29	1.50E-15
409	LRIG1	ENSG00000144749.13	0.24	1.60E-04	0.78	1.30E-49	1.07	5.20E-96

409	NCEH1	ENSG00000144959.9	0.26	0.32	0.94	3.80E-09	1.17	1.20E-14
409	ANK2	ENSG00000145362.17	-0.09	0.63	0.73	7.90E-18	0.66	6.00E-15
409	OSMR	ENSG00000145623.12	0.19	0.035	0.86	1.20E-40	0.85	1.50E-39
409	TNFAIP8	ENSG00000145779.7	0.13	0.4	0.7	8.80E-15	0.77	2.20E-18
409	RNF145	ENSG00000145860.11	0.13	0.093	0.89	6.80E-66	0.86	1.10E-61
409	SCUBE3	ENSG00000146197.8	0.34	0.28	1.3	3.00E-11	0.92	4.60E-06
409	NFKBIE	ENSG00000146232.15	0.22	0.14	2.23	1.10E-133	2.16	6.90E-125
409	CDCA5	ENSG00000146670.9	0.38	0.16	0.81	2.50E-05	1.24	3.20E-12
409	ADHFE1	ENSG00000147576.15	0.29	0.3	1.02	5.50E-09	1.04	9.20E-10
409	PTGES	ENSG00000148344.10	-0.04	0.91	0.91	4.10E-10	0.81	2.10E-08
409	ST8SIA6	ENSG00000148488.15	0.16	2	1.31	0.0019	1.07	0.011
409	HTR7	ENSG00000148680.15	0.02	0.97	0.71	1.90E-04	0.61	0.0011
409	MKI67	ENSG00000148773.13	0.55	0.05	0.94	2.20E-05	1.57	2.60E-14
409	MMP3	ENSG00000149968.11	0.44	2	1.64	0.0095	1.2	0.038
409	LYPD1	ENSG00000150551.10	0.23	0.17	0.7	2.80E-10	0.79	2.20E-13
409	VEGFC	ENSG00000150630.3	0.35	3.00E-06	0.7	1.20E-28	1.02	6.80E-63
409	ITPR1	ENSG00000150995.18	0.49	0.033	0.67	3.30E-04	0.59	0.0013
409	ADAM8	ENSG00000151651.15	0.26	0.27	0.75	1.10E-06	1.17	1.40E-16
409	ZNF385D	ENSG00000151789.10	-0.1	0.69	0.74	1.30E-10	0.6	2.10E-07
409	TDO2	ENSG00000151790.8	0.03	0.98	2.18	1.40E-07	2.65	2.80E-11
409	RASGRP3	ENSG00000152689.17	0.46	0.41	2.08	3.70E-10	2.2	9.80E-12
409	CXADR	ENSG00000154639.18	0.28	0.5	1.19	1.80E-07	1.07	2.20E-06
409	EME1	ENSG00000154920.14	0	1	0.6	0.028	0.8	0.0011
409	ADAMTS3	ENSG00000156140.9	0.29	0.44	0.71	0.0027	0.62	0.0072
409	MAP3K7CL	ENSG00000156265.15	0.28	0.24	0.73	6.70E-06	1.05	2.90E-12
409	ATAD2	ENSG00000156802.12	0.25	0.15	0.68	3.10E-08	1.01	1.40E-18
409	SASS6	ENSG00000156876.9	0.05	0.94	0.71	0.0064	0.76	0.0019
409	MYO1E	ENSG00000157483.8	0.45	3.70E-09	0.61	2.10E-18	0.83	1.90E-35
409	RHBDL2	ENSG00000158315.10	0.05	0.95	1.47	1.40E-04	1.56	2.30E-05
409	C1R	ENSG00000159403.15	0.2	0.28	0.59	3.10E-06	0.64	1.30E-07
409	LRRC36	ENSG00000159708.17	0.26	2	0.85	0.028	0.78	0.036
409	CCDC28B	ENSG00000160050.14	-0.03	0.91	0.7	1.40E-09	0.68	2.10E-09
409	CILP2	ENSG00000160161.9	-0.41	0.37	1.24	5.90E-06	0.61	0.041
409	WDR4	ENSG00000160193.11	0.16	0.48	0.63	2.00E-06	0.71	2.10E-08
409	SLC2A6	ENSG00000160326.13	0.5	0.0071	0.61	1.10E-04	1.2	2.40E-17
409	TLCD1	ENSG00000160606.10	0.21	0.36	0.68	2.50E-06	1	1.20E-13
409	RECQL4	ENSG00000160957.12	0.32	0.08	0.61	7.30E-06	0.83	6.50E-11
409	SQSTM1	ENSG00000161011.19	0.21	1.80E-05	0.71	1.20E-66	0.76	5.00E-75
409	PLXDC1	ENSG00000161381.13	-0.13	0.87	0.76	0.024	0.9	0.0035
409	SPC24	ENSG00000161888.11	0.45	0.099	0.86	1.90E-05	0.91	2.50E-06
409	CCNF	ENSG00000162063.12	0.33	0.17	0.66	1.70E-04	1.11	2.40E-12
409	PAQR4	ENSG00000162073.13	0.46	0.0055	0.9	2.10E-11	1.23	4.90E-22
409	PDPN	ENSG00000162493.16	0.81	0.13	1.04	0.017	1.62	3.60E-05
409	VCAM1	ENSG00000162692.10	-0.03	0.95	1.4	4.80E-18	1.32	2.90E-16
409	NUAK2	ENSG00000163545.8	0.09	0.77	1.63	4.70E-37	1.16	1.10E-18
409	PTX3	ENSG00000163661.3	0.49	2.50E-06	0.59	4.20E-10	0.96	3.40E-26
409	KIF15	ENSG00000163808.16	0.28	0.4	0.81	1.20E-04	1.02	2.60E-07
409	RFC4	ENSG00000163918.10	0.37	0.045	0.65	3.60E-06	0.93	1.10E-12
409	CDC25A	ENSG00000164045.11	0.21	0.54	0.77	5.60E-05	1.18	1.80E-11
409	MAD2L1	ENSG00000164109.13	0.37	0.11	0.9	4.00E-08	1.22	3.00E-15
409	ERAP1	ENSG00000164307.12	0.13	0.099	0.67	3.40E-33	0.72	1.40E-39
409	USP49	ENSG00000164663.14	0.05	0.87	0.79	1.50E-11	0.82	4.80E-13
409	MELK	ENSG00000165304.7	0.39	0.057	0.64	4.30E-05	1.15	7.10E-16
409	SKA3	ENSG00000165480.15	0.58	0.099	1.1	2.40E-05	1.47	1.40E-09
409	DDIAS	ENSG00000165490.12	0.1	0.87	1.02	3.00E-04	1.24	2.00E-06
409	EML5	ENSG00000165521.15	0.23	0.78	1.93	1.00E-06	2.49	3.70E-11
409	DDX21	ENSG00000165732.12	0.26	0.0016	0.63	5.50E-22	0.86	3.00E-42
409	HSPA12A	ENSG00000165868.13	0.32	0.0079	0.77	1.10E-16	0.95	2.70E-26
409	E2F7	ENSG00000165891.15	0.24	0.35	1.03	5.70E-11	1.22	5.90E-16
409	GPR176	ENSG00000166073.10	0.43	1.60E-17	0.83	1.80E-70	1.11	3.00E-128
409	NETO1	ENSG00000166342.18	0.05	0.96	1.64	4.90E-04	1.66	2.40E-04
409	FAM111A	ENSG00000166801.15	0.15	0.29	0.66	8.90E-15	0.76	1.90E-20
409	NKX3-1	ENSG00000167034.9	0.33	0.59	0.93	0.0095	0.85	0.014
409	KRT80	ENSG00000167767.13	0.44	0.037	0.59	7.40E-04	1	8.00E-11
409	TAP1	ENSG00000168394.10	0.15	0.43	1.09	1.70E-25	1.01	7.20E-22
409	FEN1	ENSG00000168496.3	0.54	0.0086	1.1	2.20E-11	1.44	9.80E-20

409	IL7R	ENSG00000168685.14	0.58	8.10E-07	1.37	1.70E-41	1.85	7.50E-77
409	C8orf46	ENSG00000169085.11	0.09	0.92	2.18	2.90E-10	2.2	6.90E-11
409	ATF5	ENSG00000169136.10	0.58	1.30E-11	1.29	1.60E-65	1.61	2.90E-103
409	HSPB3	ENSG00000169271.2	0.76	0.15	0.97	0.023	1.82	1.10E-06
409	COL22A1	ENSG00000169436.16	0.09	0.85	1.64	4.30E-19	1.64	1.50E-19
409	DTWD2	ENSG00000169570.9	0.46	0.18	0.63	0.018	1	1.60E-05
409	APLF	ENSG00000169621.9	0.17	0.59	0.71	2.80E-05	0.6	3.60E-04
409	BUB1	ENSG00000169679.14	0.14	0.75	0.73	7.80E-04	1.18	2.10E-09
409	ROBO1	ENSG00000169855.19	0.09	0.28	0.62	2.20E-35	0.66	4.90E-41
409	WNT10B	ENSG00000169884.13	0.23	0.55	0.64	0.0036	0.76	2.00E-04
409	REPS2	ENSG00000169891.17	0.18	0.77	1.27	1.30E-05	1.51	5.30E-08
409	CDK1	ENSG00000170312.15	0.64	0.067	1.2	6.90E-06	1.61	1.50E-10
409	CST1	ENSG00000170373.8	0.14	0.75	2.96	1.00E-53	2.35	7.80E-34
409	SLC30A1	ENSG00000170385.9	0.24	0.016	0.64	2.60E-17	0.6	5.00E-16
409	NPAS2	ENSG00000170485.16	-0.01	0.98	0.63	3.50E-17	1	2.20E-45
409	KCNS3	ENSG00000170745.11	1.08	2	1.21	0.029	1.26	0.016
409	MTSS1	ENSG00000170873.18	0.33	0.01	1.41	1.00E-53	1.52	2.70E-63
409	JUNB	ENSG00000171223.5	0.36	0.12	0.8	1.70E-06	1.07	2.00E-11
409	ENC1	ENSG00000171617.13	0.21	0.22	1.32	5.80E-38	1.26	9.00E-35
409	BCL2	ENSG00000171791.12	0.45	0.23	0.69	0.013	0.71	0.0068
409	CTPS1	ENSG00000171793.13	0.3	0.0058	0.73	2.50E-17	1.02	8.10E-35
409	SLFN11	ENSG00000172716.16	0.14	0.14	0.7	2.30E-29	0.74	2.80E-33
409	CYP7B1	ENSG00000172817.3	0.61	2	1.31	0.0078	0.84	0.039
409	LVRN	ENSG00000172901.19	0.14	2	1.39	4.00E-04	1.48	7.80E-05
409	NOC3L	ENSG00000173145.11	0.09	0.69	0.72	2.50E-12	0.63	9.00E-10
409	OLR1	ENSG00000173391.8	0.62	0.059	0.62	0.03	1.13	2.10E-06
409	DAG1	ENSG00000173402.11	0.3	1.50E-04	0.62	3.90E-20	0.72	2.40E-28
409	FOSL1	ENSG00000175592.8	0.37	0.0094	1.1	1.70E-23	1.47	3.10E-42
409	TYMS	ENSG00000176890.15	0.18	0.37	0.68	8.80E-08	0.81	3.60E-11
409	HASPIN	ENSG00000177602.5	0.01	0.99	0.85	0.0035	1.34	2.00E-07
409	ODF3B	ENSG00000177989.13	0.39	0.56	1.13	0.0046	1.16	0.0021
409	PRSS36	ENSG00000178226.10	0.05	0.95	1.09	0.0062	1.17	0.0018
409	MSC	ENSG00000178860.8	0.39	0.024	0.86	4.10E-11	1.03	3.30E-16
409	FBXO34	ENSG00000178974.9	0.24	0.0027	0.72	1.80E-32	0.88	1.40E-49
409	RRS1	ENSG00000179041.3	0.52	7.30E-04	0.91	1.20E-12	1.27	3.10E-25
409	TMTC2	ENSG00000179104.8	-0.04	0.92	0.73	5.60E-06	0.94	5.50E-10
409	CDH4	ENSG00000179242.15	0.48	0.0026	0.62	8.50E-06	0.93	7.50E-13
409	APOBEC3B	ENSG00000179750.15	0.29	0.38	0.64	0.0037	1.08	5.20E-08
409	CITED4	ENSG00000179862.6	0.33	0.14	1.18	1.60E-15	1.27	1.60E-18
409	FGD6	ENSG00000180263.13	-0.12	0.67	0.68	4.60E-07	0.8	4.40E-10
409	PHLDA2	ENSG00000181649.5	0.45	4.00E-05	0.61	3.90E-10	0.85	1.10E-19
409	GINS3	ENSG00000181938.13	0.56	0.091	0.98	7.50E-05	1.38	1.60E-09
409	C1S	ENSG00000182326.14	0.25	0.06	0.6	9.60E-10	0.67	2.90E-12
409	NTM	ENSG00000182667.14	0.55	3.90E-09	0.61	3.00E-12	0.89	4.20E-27
409	GJC1	ENSG00000182963.9	0.19	0.05	0.86	1.20E-35	1.06	4.00E-55
409	CADM1	ENSG00000182985.17	0.44	4.00E-04	0.62	1.30E-08	0.94	1.90E-20
409	CAMK1D	ENSG00000183049.12	0.58	0.017	1.1	3.80E-09	1.09	2.90E-09
409	KCTD8	ENSG00000183783.6	0.54	0.022	0.76	7.00E-05	1.13	1.10E-10
409	GPR39	ENSG00000183840.6	0.57	8.30E-04	2.06	2.90E-57	2.25	2.10E-69
409	IQGAP3	ENSG00000183856.10	0.54	0.048	0.81	2.10E-04	1.18	7.20E-09
409	NR2C2AP	ENSG00000184162.14	0.19	0.59	0.64	0.002	0.77	6.40E-05
409	ALDH1A3	ENSG00000184254.16	0.16	0.5	1	3.30E-14	1.07	1.10E-16
409	TACSTD2	ENSG00000184292.6	-0.08	0.9	0.79	0.0029	0.6	0.026
409	CSF1	ENSG00000184371.13	0.19	0.12	0.92	1.70E-29	1.26	3.80E-56
409	KNTC1	ENSG00000184445.11	0.08	0.76	0.59	5.70E-06	0.83	9.10E-12
409	DUSP8	ENSG00000184545.10	0.58	0.019	0.71	5.90E-04	1.03	3.40E-08
409	CDCA2	ENSG00000184661.13	0.51	0.07	0.76	6.70E-04	1.35	1.00E-11
409	FMNL1	ENSG00000184922.13	-0.02	0.96	0.68	5.50E-07	0.62	2.80E-06
409	USP18	ENSG00000184979.9	0.49	0.017	0.68	6.40E-05	0.65	7.10E-05
409	BRI3BP	ENSG00000184992.10	0.36	0.23	0.64	0.0035	0.97	6.70E-07
409	ANO9	ENSG00000185101.12	-0.19	0.58	1.47	0.0047	1.54	0.0024
409	C14orf80	ENSG00000185347.17	0.5	0.084	0.95	8.60E-06	1.31	3.70E-11
409	ZNF267	ENSG00000185947.14	-0.18	0.3	1.22	1.30E-34	0.81	2.90E-15
409	KIF18B	ENSG00000186185.13	0.6	0.058	1.24	1.50E-07	1.92	1.50E-18
409	KIF24	ENSG00000186638.16	0.15	0.78	0.64	0.022	1.07	1.10E-05
409	FANCA	ENSG00000187741.14	0.52	0.015	0.88	2.80E-07	1.52	1.10E-21

409	RELN	ENSG00000189056.13	0.57	0.0082	0.94	1.00E-07	1.47	1.20E-19
409	FAM111B	ENSG00000189057.10	0.52	0.19	1.66	4.30E-10	1.92	9.40E-14
409	SH2D5	ENSG00000189410.11	0.58	0.14	1.32	2.30E-06	1.86	1.20E-12
409	XRCC2	ENSG00000196584.2	0.39	0.28	0.81	0.001	1.18	1.30E-07
409	ACSL5	ENSG00000197142.10	0.39	0.36	0.96	4.10E-04	1	1.10E-04
409	BLM	ENSG00000197299.10	0.35	0.38	0.84	9.80E-04	1.16	7.00E-07
409	MAP3K5	ENSG00000197442.9	0.07	0.55	0.74	5.20E-32	0.88	9.00E-46
409	SLC28A3	ENSG00000197506.7	0.07	0.94	2.25	2.40E-06	2.47	1.10E-07
409	SULT1C4	ENSG00000198075.9	-0.11	0.75	1.15	7.70E-16	0.98	6.70E-12
409	PIM3	ENSG00000198355.4	0.02	0.9	0.77	1.50E-33	0.61	1.40E-21
409	WDHD1	ENSG00000198554.11	0.16	0.49	0.7	2.40E-07	0.88	7.90E-12
409	PNP	ENSG00000198805.11	0.26	0.35	0.85	1.20E-06	1.16	2.80E-12
409	FICD	ENSG00000198855.6	0.24	0.23	0.59	1.10E-05	0.71	2.30E-08
409	ITPRIPL1	ENSG00000198885.9	0.55	0.034	0.74	4.80E-04	0.85	2.20E-05
409	L1CAM	ENSG00000198910.12	0.2	0.5	1.22	1.70E-15	1.1	5.80E-13
409	SLC44A4	ENSG00000204385.10	-0.18	2	1.25	0.017	1.36	0.0058
409	ATP6V0E2	ENSG00000204934.10	0.66	0.1	0.95	0.0027	1.21	3.40E-05
409	PSMB10	ENSG00000205220.11	0.33	0.026	0.71	3.90E-10	0.81	1.20E-13
409	HLA-H	ENSG00000206341.7	0.2	0.37	0.87	9.10E-12	0.89	6.90E-13
409	RNU6-26F	ENSG00000206712.1	-0.34	0.63	1.01	0.0085	0.94	0.011
409	DIO2	ENSG00000211448.11	0.26	0.74	1.3	8.80E-04	1.68	3.60E-06
409	IFRD2	ENSG00000214706.10	0.28	0.014	0.64	3.00E-13	0.86	1.70E-24
409	APOL6	ENSG00000221963.5	0.11	0.51	0.82	1.60E-22	0.93	8.60E-30
409	LINC0051	ENSG00000227036.6	-0.05	0.92	0.9	2.90E-06	1.17	1.10E-10
409	HLA-B	ENSG00000234745.10	0.15	0.14	0.8	1.10E-29	0.79	4.50E-29
409	AL357060	ENSG00000237499.6	0.61	2	1.45	0.0043	1.17	0.018
409	NME1	ENSG00000239672.7	0.31	0.084	0.66	9.50E-07	0.83	9.40E-11
409	PSMB9	ENSG00000240065.7	0.15	0.77	0.84	0.0011	0.91	2.10E-04
409	LINC0097	ENSG00000240476.1	0.09	0.91	1.37	6.40E-06	1.95	6.40E-12
409	INMT	ENSG00000241644.2	0.58	0.0059	0.62	7.20E-04	0.67	1.40E-04
409	CFB	ENSG00000243649.8	-0.12	0.87	0.76	0.019	0.85	0.0044
409	SOCS2-AS	ENSG00000246985.7	0.11	0.84	0.68	0.0082	0.73	0.0028
409	FMN1	ENSG00000248905.8	0.27	0.06	0.59	4.20E-08	0.81	1.90E-15
409	AC097451	ENSG00000250657.1	0.1	0.73	1.34	2.50E-27	1.55	3.90E-37
409	AC107959	ENSG00000253616.5	-0.26	2	1.05	0.026	1.01	0.026
409	LINC0240	ENSG00000257219.5	0.33	0.54	0.96	0.0031	0.95	0.0021
409	AL122035	ENSG00000258824.2	0.7	2	0.86	0.04	0.86	0.03
409	AL365361	ENSG00000259834.1	0.36	0.43	0.94	0.00093	1.02	1.50E-04
409	AC004656	ENSG00000260822.1	0.57	2	0.92	0.048	1.24	0.0028
409	AC092718	ENSG00000261061.1	0.12	0.82	0.79	0.0014	0.87	2.00E-04
409	GATA6-AS	ENSG00000266010.1	0.65	0.21	1.23	0.0011	1.16	0.0014
409	AC004264	ENSG00000268812.3	-0.11	2	0.85	0.038	1.14	0.0019
409	AC009549	ENSG00000270607.1	0.26	0.58	1.24	3.50E-07	1.02	2.90E-05
409	CU639417	ENSG00000275993.2	0.03	0.97	1.55	1.70E-04	1.11	0.0068
409	UHRF1	ENSG00000276043.4	0.5	0.0018	0.9	7.00E-12	1.35	6.20E-27
409	PIGW	ENSG00000277161.1	0.34	0.045	0.74	4.70E-09	0.9	7.60E-14
409	AC093535	ENSG00000279118.1	0.84	0.17	1.49	0.0033	1.58	0.0012
409	AC016397	ENSG00000279822.1	0.12	0.87	0.76	0.032	0.79	0.019
409	AC245100	ENSG00000280649.2	0.17	0.77	1.01	3.90E-04	0.67	0.024
409	AC241585	ENSG00000281571.2	-0.1	0.91	0.94	0.012	0.75	0.043
95	IL32	ENSG00000008517.16	0.72	0.015	3.36	2.70E-55	3.59	2.40E-63
95	BIRC3	ENSG00000023445.13	1.09	0.005	4.34	5.90E-45	4.82	2.80E-55
95	DEPDC1	ENSG00000024526.16	0.77	0.0013	1.59	1.00E-16	1.89	2.20E-24
95	POU2F2	ENSG00000028277.21	0.87	3.70E-08	1.13	6.30E-15	2.52	1.60E-81
95	ARNTL2	ENSG00000029153.14	0.77	3.40E-08	2.29	3.80E-82	2.73	1.60E-117
95	DNAH5	ENSG00000039139.9	0.67	1.90E-08	0.81	2.40E-13	1.09	1.40E-24
95	HDAC9	ENSG00000048052.21	0.86	0.0041	1.98	1.70E-17	2.21	2.40E-22
95	RAD51	ENSG00000051180.16	0.65	0.0027	0.88	2.30E-06	1.24	8.00E-13
95	MCM10	ENSG00000065328.16	0.8	0.0062	1.35	1.30E-08	2.03	7.80E-20
95	TFRC	ENSG00000072274.12	0.67	6.10E-13	0.64	9.90E-13	1.16	2.90E-43
95	NDC80	ENSG00000080986.12	0.64	0.018	1.23	3.90E-09	1.61	4.80E-16
95	P3H2	ENSG00000090530.9	0.63	4.90E-06	1.16	3.40E-23	1.83	9.40E-63
95	CD200	ENSG00000091972.18	0.68	0.025	0.61	0.027	0.87	3.40E-04
95	CDC45	ENSG00000093009.9	0.7	0.013	1.1	1.20E-06	1.72	3.30E-16
95	CDC6	ENSG00000094804.9	0.7	1.30E-04	1.55	7.20E-25	2.06	2.70E-45

95	DERL3	ENSG00000099958.14	0.94	0.022	0.9	0.013	1.65	1.50E-07
95	MYBL2	ENSG00000101057.15	0.85	0.0022	1.11	2.70E-06	1.84	7.20E-17
95	E2F1	ENSG00000101412.12	0.67	0.0015	1.14	1.00E-10	1.53	8.00E-20
95	FAM83D	ENSG00000101447.14	0.63	0.025	0.93	4.90E-05	1.61	4.80E-15
95	KLF5	ENSG00000102554.13	0.77	4.00E-04	0.91	2.00E-06	1.87	8.90E-28
95	WISP1	ENSG00000104415.13	0.69	0.03	1.34	2.20E-08	1.56	1.00E-11
95	SLC39A14	ENSG00000104635.13	0.61	7.90E-20	0.79	4.10E-36	1.49	1.10E-136
95	IL4I1	ENSG00000104951.15	1.31	0.0017	3.27	3.10E-21	3.68	6.30E-27
95	ASF1B	ENSG00000105011.8	0.72	0.043	1.28	4.70E-06	1.77	1.80E-11
95	SLC1A1	ENSG00000106688.11	0.71	0.0046	1.16	1.00E-08	1.32	1.20E-11
95	MAP3K8	ENSG00000107968.9	0.91	0.015	1.16	2.20E-04	1.41	1.30E-06
95	NCAPG	ENSG00000109805.9	0.61	0.049	1.02	2.80E-05	1.41	7.10E-10
95	HBEGF	ENSG00000113070.7	0.71	0.0012	1.58	5.60E-20	2.27	4.70E-43
95	CDH6	ENSG00000113361.12	0.78	5.60E-04	0.93	3.60E-06	1.06	2.60E-08
95	CENPA	ENSG00000115163.14	0.99	0.011	1.58	5.70E-07	1.71	1.90E-08
95	QPCT	ENSG00000115828.15	0.59	0.016	0.78	1.60E-04	1.19	1.40E-10
95	TNFSF4	ENSG00000117586.10	0.7	7.90E-14	1.82	6.50E-114	2.17	4.20E-167
95	NCAPH	ENSG00000121152.9	0.8	0.0062	1.14	2.30E-06	1.71	3.00E-14
95	CXCL6	ENSG00000124875.9	2.65	6.80E-13	2.12	3.10E-09	5.83	2.40E-63
95	C3	ENSG00000125730.16	2.48	2.50E-14	2.46	2.40E-15	4.67	9.30E-55
95	PKMYT1	ENSG00000127564.16	0.75	0.001	1.45	9.50E-15	1.89	4.20E-26
95	TICAM1	ENSG00000127666.9	0.69	1.60E-12	0.62	6.50E-11	1.05	6.20E-33
95	ADM2	ENSG00000128165.8	1.14	1.80E-04	0.79	0.008	1.35	2.60E-07
95	DLL4	ENSG00000128917.6	1.02	0.011	1.36	4.70E-05	2.13	3.30E-12
95	SGO1	ENSG00000129810.14	1	0.018	1.36	1.10E-04	1.58	2.00E-06
95	TOP2A	ENSG00000131747.14	0.68	0.011	1.14	1.80E-07	1.53	2.50E-13
95	STX11	ENSG00000135604.9	1.3	0.0045	1.39	5.80E-04	1.65	1.70E-05
95	SULF1	ENSG00000137573.13	1.29	2.60E-04	1.31	5.40E-05	2.29	3.80E-14
95	SLC7A1	ENSG00000139514.12	0.74	6.40E-12	0.88	5.70E-18	1.42	2.40E-48
95	DIAPH3	ENSG00000139734.17	0.63	0.0086	0.88	1.30E-05	1.33	2.00E-12
95	TICRR	ENSG00000140534.13	0.96	0.0042	1.15	8.10E-05	2.01	2.60E-14
95	DTL	ENSG00000143476.17	0.76	0.0065	1.32	8.00E-09	1.85	1.60E-17
95	SLIT2	ENSG00000145147.19	0.79	4.00E-13	1.95	3.00E-92	2.11	3.20E-109
95	UGCG	ENSG00000148154.9	0.62	1.30E-08	1.01	2.80E-25	1.44	3.40E-52
95	ANKRD1	ENSG00000148677.6	0.9	0.01	1.56	4.90E-08	2.12	4.50E-15
95	NOCT	ENSG00000151014.5	0.67	0.0026	0.94	5.40E-07	1.82	2.00E-27
95	CENPU	ENSG00000151725.11	0.88	3.80E-05	1.51	1.20E-16	1.68	3.90E-21
95	GFRA1	ENSG00000151892.14	0.67	0.0048	0.84	3.20E-05	1.58	3.30E-19
95	SPC25	ENSG00000152253.8	0.75	0.046	0.88	0.005	1.4	4.90E-07
95	SKA1	ENSG00000154839.9	0.92	0.0086	1.42	7.30E-07	1.73	1.70E-10
95	GRAMD2B	ENSG00000155324.9	0.7	4.20E-05	0.69	1.30E-05	1.23	5.80E-18
95	BUB1B	ENSG00000156970.12	0.66	0.0055	1.12	6.90E-09	1.54	3.20E-17
95	IL34	ENSG00000157368.10	1.82	6.90E-08	4.95	5.60E-60	5.04	1.50E-62
95	CTSS	ENSG00000163131.10	1.23	4.60E-06	2.87	5.20E-36	3.25	2.50E-46
95	CLDN1	ENSG00000163347.5	1.21	2.30E-13	2.29	4.30E-57	3.02	8.90E-105
95	CXCL5	ENSG00000163735.6	1.17	3.60E-04	1.38	1.80E-06	3.35	1.40E-37
95	CXCL1	ENSG00000163739.4	2.78	1.90E-45	1.41	2.30E-11	5.32	3.10E-175
95	SGMS2	ENSG00000164023.14	0.65	1.30E-04	0.71	5.00E-06	0.88	1.80E-09
95	ZNF367	ENSG00000165244.6	0.63	0.036	1.6	3.80E-13	1.95	3.60E-20
95	C10orf10	ENSG00000165507.8	0.6	0.0031	1.05	5.20E-10	1.31	5.30E-16
95	RRAD	ENSG00000166592.11	0.8	2.40E-08	2.21	8.90E-74	2.54	1.40E-99
95	NNMT	ENSG00000166741.7	0.79	1.70E-05	0.86	2.90E-07	1.93	1.30E-39
95	TAC3	ENSG00000166863.11	1.34	2.70E-04	4.54	2.50E-47	4.79	8.90E-53
95	B4GALNT2	ENSG00000167080.8	0.85	0.034	0.78	0.029	1.22	9.20E-05
95	PBK	ENSG00000168078.9	0.74	0.0041	1.03	2.00E-06	1.48	2.20E-13
95	MFSD2A	ENSG00000168389.17	1.06	7.70E-04	1.49	2.30E-08	2.32	6.50E-21
95	CXCL8	ENSG00000169429.10	1.64	4.10E-07	0.79	0.022	3.36	4.20E-32
95	CKAP2L	ENSG00000169607.12	0.69	0.018	1.14	1.30E-06	1.65	4.30E-14
95	SHCBP1	ENSG00000171241.8	0.7	0.0047	1.2	3.00E-09	1.76	3.40E-20
95	ESCO2	ENSG00000171320.14	0.9	0.041	1.59	4.70E-06	2.09	2.10E-10
95	PTGER4	ENSG00000171522.5	0.68	1.60E-08	0.73	9.90E-11	1.16	3.80E-28
95	RRM2	ENSG00000171848.14	0.61	0.024	0.96	1.10E-05	1.51	9.60E-14
95	EXO1	ENSG00000174371.16	0.92	5.20E-04	1.57	1.10E-12	1.98	1.40E-20
95	UBE2C	ENSG00000175063.16	0.89	0.0017	1.19	9.90E-07	1.71	8.30E-14
95	CCNE2	ENSG00000175305.17	0.72	0.044	1.29	2.90E-06	1.69	9.80E-11
95	ATAD5	ENSG00000176208.8	0.7	0.025	1.11	8.30E-06	1.33	1.60E-08

95	KCNA3	ENSG00000177272.8	1.06	1.60E-06	1.72	3.40E-19	2.15	2.70E-31
95	FJX1	ENSG00000179431.6	0.6	9.00E-07	1.01	5.40E-21	1.29	9.00E-35
95	TNFSF15	ENSG00000181634.7	1.02	0.0045	2.86	2.10E-25	2.93	6.00E-27
95	NLRP10	ENSG00000182261.3	2.16	5.10E-04	2.28	6.20E-05	3.03	2.80E-07
95	NOG	ENSG00000183691.4	1.3	0.0025	0.92	0.027	1.55	2.00E-05
95	MYBL1	ENSG00000185697.16	1.1	5.30E-07	3.51	4.40E-80	3.9	1.40E-99
95	ERCC6L	ENSG00000186871.6	0.91	0.017	1.73	9.40E-09	2.15	1.10E-13
95	PDCD1LG2	ENSG00000197646.7	0.72	5.10E-04	0.98	4.00E-08	1.58	5.50E-22
95	ALPK2	ENSG00000198796.6	0.63	1.60E-08	1.12	2.40E-29	1.72	5.50E-72
95	PRC1	ENSG00000198901.13	0.6	0.01	0.85	1.10E-05	1.29	1.20E-12
95	KIFC1	ENSG00000237649.7	0.86	0.0013	1.28	2.00E-08	1.85	1.40E-17
95	RPSAP52	ENSG00000241749.4	0.79	0.014	0.63	0.038	1.24	9.30E-07
95	AC125807	ENSG00000250899.3	0.61	0.015	0.85	3.60E-05	1.05	4.30E-08
95	AC124798	ENSG00000260196.1	0.8	0.029	0.69	0.038	1.49	5.60E-08
43	CCL26	ENSG00000006606.8	2.5	0.0013	0.15	0.8	1.43	0.015
43	NRXN3	ENSG00000021645.18	1.45	8.30E-04	0.62	0.18	1.46	1.20E-04
43	LAMP3	ENSG00000078081.7	1.55	0.011	0.36	0.55	1.74	8.10E-04
43	EDN1	ENSG00000078401.6	1.1	0.0022	-0.1	0.85	0.79	0.016
43	CXCL2	ENSG00000081041.8	1.18	0.005	0.68	0.11	3.26	1.20E-22
43	DSP	ENSG00000096696.13	0.63	0.016	0.13	0.71	0.82	8.00E-05
43	SLC7A5	ENSG00000103257.8	0.86	9.00E-09	0.49	0.0012	1.16	1.60E-17
43	CEMIP	ENSG00000103888.16	0.8	0.0055	0.16	0.65	0.85	4.80E-04
43	SPAG1	ENSG00000104450.12	0.86	0.0062	0.46	0.16	0.91	5.70E-04
43	NEDD9	ENSG00000111859.16	0.81	1.90E-09	0.37	0.011	0.91	2.00E-13
43	FGF1	ENSG00000113578.17	0.85	8.80E-05	0.32	0.19	0.99	1.90E-07
43	AMOTL2	ENSG00000114019.14	0.61	7.30E-10	0.25	0.021	0.72	1.70E-15
43	SPP1	ENSG00000118785.13	1.51	0.0039	0.9	0.072	0.92	0.048
43	EGR1	ENSG00000120738.7	0.72	0.002	0.11	0.72	0.78	9.40E-05
43	KLF2	ENSG00000127528.5	0.8	7.30E-09	-0.05	0.82	0.74	1.20E-08
43	CHAC1	ENSG00000128965.11	0.98	3.10E-08	0.53	0.0043	1.2	5.20E-14
43	RFXAP	ENSG00000133111.3	0.62	0.0043	-0.26	0.29	0.65	4.30E-04
43	PSAT1	ENSG00000135069.13	0.71	1.20E-05	0.23	0.21	1	2.00E-12
43	IER3	ENSG00000137331.11	0.83	1.10E-12	0.38	0.0024	1.03	1.40E-21
43	RNF144B	ENSG00000137393.9	1.09	0.019	-0.02	0.98	0.85	0.038
43	AMIGO2	ENSG00000139211.6	0.66	1.40E-06	0.53	6.80E-05	1.11	2.00E-20
43	INHBE	ENSG00000139269.2	1.04	0.0026	0.54	0.14	1.15	8.60E-05
43	CYR61	ENSG00000142871.16	0.97	1.50E-08	0.47	0.0083	1.33	1.10E-17
43	KCNH1	ENSG00000143473.12	0.76	1.40E-04	-0.15	0.59	0.71	8.00E-05
43	CSRNP1	ENSG00000144655.14	0.61	1.90E-04	0.35	0.037	0.75	1.50E-07
43	NFKBIZ	ENSG00000144802.11	2.22	3.00E-75	0.58	2.30E-05	2.58	3.20E-103
43	VLDLR	ENSG00000147852.15	0.74	4.10E-04	0.43	0.05	0.62	0.0011
43	ELF3	ENSG00000163435.15	2.88	5.80E-09	0.9	0.085	4.88	4.80E-26
43	ZC3H12A	ENSG00000163874.10	2.1	3.90E-88	0.49	7.90E-05	2.94	4.50E-180
43	ARNT2	ENSG00000172379.20	0.59	5.10E-04	0.28	0.12	0.71	1.10E-06
43	C4orf32	ENSG00000174749.5	0.67	0.017	0.35	0.23	0.91	4.90E-05
43	LIPT2	ENSG00000175536.6	0.86	0.026	0.57	0.13	0.67	0.05
43	MB21D2	ENSG00000180611.6	0.59	6.20E-04	0.41	0.015	0.94	4.50E-11
43	MAFF	ENSG00000185022.11	0.59	9.50E-08	0.55	2.10E-07	0.86	6.60E-18
43	MT-TT	ENSG00000210195.2	0.63	0.024	-0.41	0.15	0.97	7.00E-06
43	FGFR1OP	ENSG00000213066.11	0.78	1.20E-08	-0.01	0.98	0.85	9.70E-12
43	ZNF469	ENSG00000225614.2	0.61	3.80E-12	0.21	0.032	0.68	6.60E-17
43	AC027237	ENSG00000259426.5	0.81	0.0017	0.28	0.34	0.89	5.10E-05
43	LINC0056	ENSG00000260910.1	0.78	0.012	0.41	0.2	0.78	0.0028
43	ANXA8L1	ENSG00000264230.7	1.14	0.026	0.36	0.53	0.99	0.023
43	AL158152	ENSG00000269929.1	0.87	0.0012	0.01	0.98	0.87	2.00E-04
43	GAS6-AS2	ENSG00000272695.1	0.62	1.80E-04	0.42	0.0091	0.69	1.40E-06
43	AP000892	ENSG00000280143.1	0.79	7.50E-04	0.32	0.21	0.9	7.50E-06

Table S6. Pathway analysis for 484 unique transcripts upregulated by LIGHT with IL-17 in human pulmonary fibroblasts, associated with Fig. 2E-F.

Category	Term	Count	%	PValue	Genes	List Total	Pop Hits	Pop Total	Fold Enric	Bonferron	Benjamini	FDR
UP_KW_BIOLOGICAL_PROCESS	KW-0131~Cell cycle	43	10.11765	2.17E-09	SUV39H2, CDCA3, MCM7, MCM8, DSCC1, DDX12P, NCAPG2, GMNN, KIF11, FOXM1, CHTF18, AURKB, AURKA, CCNB2, CHAF1B, CHAF1A, RACGAP1, PTTG1, RBBP8, E2F3, FBXO5, PIM2, FANCI, GADD45A, PLK1, HMGA2, VRK1, CDC25C, CCNA2, CYLD, SGO2, TPX2, DBF4, SAPCD2, CCNE1, INCENP, DMC1, TIMELESS, BIRC5, MCM4, NCAPD3, MCM6, CDKN3	257	684	11371	2.781492	2.30E-07	2.32E-07	2.26E-07
UP_KW_BIOLOGICAL_PROCESS	KW-0235~DNA replication	15	3.529412	2.78E-08	PCNA, MCM7, MCM8, RFC2, DSCC1, CHTF18, POLD3, POLA1, CHAF1B, DBF4, CHAF1A, ORC1, GRWD1, MCM4, MCM6	257	96	11371	6.913303	2.95E-06	1.49E-06	1.45E-06
KEGG_PATHWAY	hsa04110:Cell cycle	18	4.235294	1.05E-07	PCNA, SMAD3, MCM7, GADD45A, PLK1, CDC25C, AURKB, CCNA2, CCNB2, DBF4, ORC1, PTTG1, CCNE1, MYC, MCM4, E2F3, MCM6, FBXO5	200	157	8644	4.955159	2.82E-05	2.82E-05	2.77E-05
GOTERM_BP_DIRECT	GO:0030335~positive regulation of cell migration	21	4.941176	3.42E-07	LYN, LRRC15, SEMA7A, SMAD3, CSF2, WNT5B, EGF, MCAM, ARHGEF39, PDGFB, PIK3CD, RRAS2, CLDN4, PODXL, NTF3, PECAM1, GCNT2, SPRY2, EPHB2, BCAR1, EPHA2	382	267	19453	4.005265	8.57E-04	8.58E-04	8.54E-04
GOTERM_BP_DIRECT	GO:0006260~DNA replication	14	3.294118	8.14E-07	PCNA, MCM7, RFC2, DSCC1, CHTF18, POLD3, POLA1, CHAF1B, DBF4, CHAF1A, ORC1, GRWD1, MCM4, MCM6	382	122	19453	5.843747	0.002039	0.00102	0.001016
GOTERM_BP_DIRECT	GO:0000278~mitotic cell cycle	14	3.294118	1.10E-05	PLK1, NOLC1, TUBD1, KIF11, KIF22, TUBB4B, AURKB, AURKA, TUBA1C, TPX2, TUBA1B, INCENP, MYB, BIRC5	382	154	19453	4.629462	0.027274	0.009217	0.009177
GOTERM_BP_DIRECT	GO:0051301~cell division	22	5.176471	2.41E-05	CDCA3, NCAPG2, HMGA2, VRK1, KIF11, CDC25C, AURKB, AURKA, CCNA2, SGO2, CCNB2, TUBA1C, TPX2, TUBA1B, PTTG1, CCNE1, RBBP8, TIMELESS, BIRC5, NCAPD3, FBXO5, BCAR1	382	383	19453	2.925143	0.058567	0.01504	0.014974
GOTERM_BP_DIRECT	GO:0008284~positive regulation of cell proliferation	27	6.352941	3.00E-05	CSF3, CNTF, CSF2, ODC1, NOP2, PDGFB, TNC, NOLC1, FOXM1, FGF5, BCL7A, MYC, NTF3, GCNT2, E2F3, FBXO5, LYN, EGF, PRLR, GREM1, IL6, SAPCD2, BIRC5, CD47, FXN, ATF3, EZH2	382	545	19453	2.522845	0.072445	0.01504	0.014974
UP_KW_BIOLOGICAL_PROCESS	KW-0498~Mitosis	20	4.705882	3.17E-05	CDCA3, PLK1, NCAPG2, HMGA2, VRK1, KIF11, CDC25C, AURKB, AURKA, CCNA2, CCNB2, TPX2, SAPCD2, PTTG1, INCENP, RBBP8, TIMELESS, BIRC5, NCAPD3, FBXO5	257	291	11371	3.040903	0.003349	0.001129	0.001097
GOTERM_BP_DIRECT	GO:0000082~G1/S transition of mitotic cell cycle	9	2.117647	5.93E-05	CCNA2, DBF4, CCNE1, MYC, RBBP8, E2F3, PIM2, EZH2, CDKN3	382	69	19453	6.642272	0.138193	0.024787	0.024678
GOTERM_BP_DIRECT	GO:0010628~positive regulation of gene expression	25	5.882353	9.78E-05	CNTF, DNMT1, CSF2, PDGFB, TNC, PIK3CD, ETS1, FGF5, MYC, EPHB2, LDLR, SMAD3, EGF, VDR, PAWR, HMGA2, INHBA, DKK1, IL6, ACTC1, SPRY2, EZR, GAS6, ATF3, LIMS1	382	519	19453	2.452991	0.217419	0.035021	0.034867
KEGG_PATHWAY	hsa03030:DNA replication	7	1.647059	1.51E-04	POLD3, POLA1, PCNA, MCM7, RFC2, MCM4, MCM6	200	36	8644	8.403889	0.03984	0.020326	0.020024
GOTERM_BP_DIRECT	GO:0032508~DNA duplex unwinding	8	1.882353	1.65E-04	BRIP1, MCM8, RFC2, DSCC1, ERCC2, DDX12P, NAV2, CHTF18	382	60	19453	6.789878	0.339499	0.05184	0.051613
UP_KW_BIOLOGICAL_PROCESS	KW-0132~Cell division	23	5.411765	1.72E-04	CDCA3, PLK1, NCAPG2, HMGA2, VRK1, KIF11, CDC25C, AURKB, AURKA, CCNA2, SGO2, CCNB2, TPX2, RACGAP1, SAPCD2, PTTG1, CCNE1, INCENP, RBBP8, TIMELESS, BIRC5, NCAPD3, FBXO5	257	415	11371	2.45214	0.018098	0.004609	0.004479
GOTERM_BP_DIRECT	GO:0006270~DNA replication initiation	6	1.411765	1.90E-04	POLA1, MCM7, ORC1, CCNE1, MCM4, MCM6	382	28	19453	10.9123	0.379463	0.053014	0.052781
KEGG_PATHWAY	hsa04218:Cellular senescence	13	3.058824	2.46E-04	SMAD3, GADD45A, PIK3CD, RRAS2, FOXM1, ETS1, CCNA2, CCNB2, IL6, CCNE1, MYC, E2F3, LIN52	200	156	8644	3.601667	0.063989	0.02204	0.021712
BIOCARTA	h_ranMSPathway:Role of Ran in mitotic spindle regulation	5	1.176471	2.62E-04	TPX2, RANGAP1, KPNA2, SNHG3, AURKA	53	11	1623	13.91938	0.028695	0.02911	0.02911
GOTERM_BP_DIRECT	GO:0006271~DNA strand elongation involved in DNA replication	4	0.941176	3.88E-04	POLD3, POLA1, MCM7, MCM4	382	8	19453	25.46204	0.622007	0.097269	0.096842
KEGG_PATHWAY	hsa04668:TNF signaling pathway	10	2.352941	0.001222	CYLD, SOCS3, IL6, CSF2, MLKL, CCL20, PIK3CD, PGAM5, CXCL3, PTGS2	200	114	8644	3.791228	0.280244	0.08216	0.080939
UP_KW_BIOLOGICAL_PROCESS	KW-0234~DNA repair	18	4.235294	0.003051	FANCI, DCLRE1B, PCNA, MCM8, XRCC3, TONSL, FANCB, FOXM1, POLD3, CHAF1B, BRIP1, CHAF1A, PTTG1, RFWD3, ERCC2, USP1, RBBP8, TIMELESS	257	358	11371	2.224616	0.276651	0.065284	0.063454
UP_KW_BIOLOGICAL_PROCESS	KW-0227~DNA damage	20	4.705882	0.003838	FANCI, DCLRE1B, PCNA, MCM8, GADD45A, XRCC3, TONSL, AEN, FANCB, FOXM1, POLD3, CHAF1B, BRIP1, CHAF1A, PTTG1, RFWD3, ERCC2, USP1, RBBP8, TIMELESS	257	429	11371	2.06271	0.334775	0.068448	0.066528

Table S7. Pathway analysis for synergistically upregulated transcripts induced by LIGHT with IL-13 in human pulmonary fibroblasts, associated with Fig. 3C-D.

Category	Term	Count	%	PValue	Genes	List Total
UP_KW_BIOLOGICAL_PROCESS	KW-0145~Chemotaxis	4	11.42857	4.35E-04	ANOS1, CCL2, SLIT2, CCL26	18
KEGG_PATHWAY	hsa04060:Cytokine-cytokine receptor interaction	5	14.28571	8.26E-04	IL32, IL1RL1, CCL2, IL13RA2, CCL26	14
GOTERM_BP_DIRECT	GO:0048247~lymphocyte chemotaxis	3	8.571429	0.001341	CCL2, ADAM8, CCL26	32
GOTERM_CC_DIRECT	GO:0005615~extracellular space	10	28.57143	0.001872	IL32, SCUBE3, ACTC1, ANOS1, CCL2, SLIT2, SULF1, IL13RA2, CBLN2, CCL26	32
GOTERM_BP_DIRECT	GO:0006954~inflammatory response	5	14.28571	0.004575	IL1RL1, NLRP10, CCL2, ADAM8, CCL26	32
GOTERM_MF_DIRECT	GO:0001228~transcriptional activator activity, RNA polymerase II transcription regulatory region sequence-specific binding	5	14.28571	0.005031	EGR2, EHF, MYB, MYBL1, MEOX1	29
UP_KW_CELLULAR_COMPONENT	KW-0964~Secreted	10	28.57143	0.005784	IL32, SCUBE3, IL1RL1, IL4I1, ANOS1, CCL2, SLIT2, SULF1, CBLN2, CCL26	30
GOTERM_MF_DIRECT	GO:0043565~sequence-specific DNA binding	4	11.42857	0.013679	EGR2, EHF, MYBL1, MEOX1	29
GOTERM_CC_DIRECT	GO:0009986~cell surface	5	14.28571	0.01598	SCUBE3, ANOS1, ADAM8, SLIT2, SULF1	32
GOTERM_BP_DIRECT	GO:0006935~chemotaxis	3	8.571429	0.016422	ANOS1, CCL2, CCL26	32
GOTERM_MF_DIRECT	GO:0071837~HMG box domain binding	2	5.714286	0.020501	EGR2, MEOX1	29
GOTERM_MF_DIRECT	GO:0005509~calcium ion binding	5	14.28571	0.023434	CDH6, SCUBE3, ADAM8, SLIT2, SULF1	29
UP_KW_PTM	KW-0325~Glycoprotein	14	40	0.030193	SPINT2, SULF1, HS3ST1, CDH6, SCUBE3, IL4I1, IL1RL1, CH25H, ANOS1, CCL2, ADAM8, SLIT2, IL13RA2, CBLN2	25
UP_KW_BIOLOGICAL_PROCESS	KW-0395~Inflammatory response	3	8.571429	0.032294	NLRP10, CCL2, CCL26	18
GOTERM_BP_DIRECT	GO:0043547~positive regulation of GTPase activity	3	8.571429	0.036443	RGS4, CCL2, CCL26	32
UP_KW_PTM	KW-1015~Disulfide bond	12	34.28571	0.037152	SCUBE3, IL1RL1, IL4I1, ANOS1, CCL2, ADAM8, SLIT2, SPINT2, IL13RA2, HS3ST1, CBLN2, CCL26	25
GOTERM_MF_DIRECT	GO:0048020~CCR chemokine receptor binding	2	5.714286	0.042016	CCL2, CCL26	29
GOTERM_BP_DIRECT	GO:0008045~motor neuron axon guidance	2	5.714286	0.042258	EGR2, SLIT2	32
GOTERM_BP_DIRECT	GO:0045944~positive regulation of transcription from RNA polymerase II promoter	6	17.14286	0.042533	IL4I1, EGR2, EHF, MYB, MYBL1, MEOX1	32
GOTERM_BP_DIRECT	GO:0048245~eosinophil chemotaxis	2	5.714286	0.043789	CCL2, CCL26	32
GOTERM_MF_DIRECT	GO:0003700~transcription factor activity, sequence-specific DNA binding	4	11.42857	0.049341	EGR2, EHF, MYBL1, MEOX1	29
UP_KW_MOLECULAR_FUNCTION	KW-0202~Cytokine	3	8.571429	0.051406	IL32, CCL2, CCL26	23
GOTERM_BP_DIRECT	GO:0007155~cell adhesion	4	11.42857	0.059854	IL32, CDH6, ANOS1, CCL2	32
GOTERM_BP_DIRECT	GO:0030513~positive regulation of BMP signaling pathway	2	5.714286	0.064982	SCUBE3, SULF1	32
GOTERM_BP_DIRECT	GO:0002548~monocyte chemotaxis	2	5.714286	0.069464	CCL2, CCL26	32
GOTERM_MF_DIRECT	GO:0008009~chemokine activity	2	5.714286	0.071373	CCL2, CCL26	29
GOTERM_MF_DIRECT	GO:0004896~cytokine receptor activity	2	5.714286	0.082327	IL1RL1, IL13RA2	29
GOTERM_BP_DIRECT	GO:0060348~bone development	2	5.714286	0.087184	TTC9, SULF1	32
GOTERM_MF_DIRECT	GO:0003924~GTPase activity	3	8.571429	0.09762	RGS4, ARL14, NLRP10	29

Table S8. Pathway analysis for synergistically upregulated transcripts induced by LIGHT with IL-17 in human pulmonary fibroblasts, associated with Fig. 4C-D.

Category	Term	Count	%	PValue	Genes	List Total
KEGG_PATHWAY	hsa04657:IL-17 signaling pathway	9	47.36842	2.74E-14	CXCL6, CSF3, CXCL8, CSF2, CCL20, CXCL1, CXCL3, CXCL2, CXCL5	12
INTERPRO	IPR001089:CXC chemokine	6	31.57895	3.70E-13	CXCL6, CXCL8, CXCL1, CXCL3, CXCL2, CXCL5	18
INTERPRO	IPR018048:CXC chemokine, conserved site	6	31.57895	3.70E-13	CXCL6, CXCL8, CXCL1, CXCL3, CXCL2, CXCL5	18
GOTERM_MF_DIRECT	GO:0045236~CXCR chemokine receptor binding	6	31.57895	9.09E-13	CXCL6, CXCL8, CXCL1, CXCL3, CXCL2, CXCL5	18
INTERPRO	IPR001811:Chemokine interleukin-8-like domain	7	36.84211	1.66E-12	CXCL6, CXCL8, CCL20, CXCL1, CXCL3, CXCL2, CXCL5	18
GOTERM_BP_DIRECT	lipopolysaccharide	9	47.36842	2.92E-12	CXCL6, CSF3, CXCL8, CSF2, ZC3H12A, CXCL1, CXCL3, CXCL2, CXCL5	19
GOTERM_MF_DIRECT	GO:0008009~chemokine activity	7	36.84211	3.00E-12	CXCL6, CXCL8, CCL20, CXCL1, CXCL3, CXCL2, CXCL5	18
KEGG_PATHWAY	hsa05323:Rheumatoid arthritis	8	42.10526	4.89E-12	CXCL6, CXCL8, CSF2, CCL20, CXCL1, CXCL3, CXCL2, CXCL5	12
SMART	SM00199:SCY	7	36.84211	1.71E-11	CXCL6, CXCL8, CCL20, CXCL1, CXCL3, CXCL2, CXCL5	16
KEGG_PATHWAY	hsa04668:TNF signaling pathway	8	42.10526	2.11E-11	CXCL6, CSF2, CCL20, CXCL1, CXCL3, CXCL2, CXCL5, BIRC3	12
UP_KW_MOLECULAR_FUNCTION	KW-0202~Cytokine	9	47.36842	3.03E-11	CXCL6, CSF3, CXCL8, CSF2, CCL20, CXCL1, CXCL3, CXCL2, CXCL5	16
GOTERM_BP_DIRECT	GO:0070098~chemokine-mediated signaling pathway	7	36.84211	3.17E-11	CXCL6, CXCL8, CCL20, CXCL1, CXCL3, CXCL2, CXCL5	19
GOTERM_BP_DIRECT	GO:0006954~inflammatory response	10	52.63158	4.61E-11	C3, CXCL6, CXCL8, ELF3, CCL20, ZC3H12A, CXCL1, CXCL3, CXCL2, CXCL5	19
GOTERM_BP_DIRECT	GO:0030593~neutrophil chemotaxis	7	36.84211	8.40E-11	CXCL6, CXCL8, CCL20, CXCL1, CXCL3, CXCL2, CXCL5	19
KEGG_PATHWAY	hsa04060:Cytokine-cytokine receptor interaction	9	47.36842	3.15E-10	CXCL6, CSF3, CXCL8, CSF2, CCL20, CXCL1, CXCL3, CXCL2, CXCL5	12
GOTERM_BP_DIRECT	GO:0061844~antimicrobial humoral immune response mediated by antimicrobial peptide	7	36.84211	4.79E-10	CXCL6, CXCL8, CCL20, CXCL1, CXCL3, CXCL2, CXCL5	19
UP_KW_BIOLOGICAL_PROCESS	KW-0395~Inflammatory response	8	42.10526	5.29E-10	C3, CXCL8, ELF3, CCL20, ZC3H12A, CXCL1, CXCL3, CXCL2	14
KEGG_PATHWAY	hsa04061:Viral protein interaction with cytokine and cytokine receptor	7	36.84211	1.03E-09	CXCL6, CXCL8, CCL20, CXCL1, CXCL3, CXCL2, CXCL5	12
KEGG_PATHWAY	hsa04062:Chemokine signaling pathway	7	36.84211	5.30E-08	CXCL6, CXCL8, CCL20, CXCL1, CXCL3, CXCL2, CXCL5	12
UP_KW_BIOLOGICAL_PROCESS	KW-0145~Chemotaxis	6	31.57895	6.38E-08	CXCL6, CXCL8, CCL20, CXCL3, CXCL2, CXCL5	14
GOTERM_BP_DIRECT	GO:0006935~chemotaxis	6	31.57895	7.54E-08	CXCL6, CXCL8, CCL20, CXCL1, CXCL2, CXCL5	19
GOTERM_BP_DIRECT	GO:0006955~immune response	8	42.10526	1.72E-07	C3, CXCL6, CSF3, CXCL8, CSF2, CCL20, CXCL2, CXCL5	19
UP_SEQ_FEATURE	DOMAIN:Chemokine interleukin-8-like	4	21.05263	5.74E-07	CXCL6, CXCL8, CXCL2, CXCL5	15
KEGG_PATHWAY	hsa05134:Legionellosis	5	26.31579	5.89E-07	C3, CXCL8, CXCL1, CXCL3, CXCL2	12
GOTERM_BP_DIRECT	GO:0031640~killing of cells of other organism	5	26.31579	1.31E-06	CXCL8, CCL20, CXCL1, CXCL3, CXCL2	19
KEGG_PATHWAY	hsa05167:Kaposi sarcoma-associated herpesvirus infection	6	31.57895	2.48E-06	C3, CXCL8, CSF2, CXCL1, CXCL3, CXCL2	12
KEGG_PATHWAY	hsa05146:Amoebiasis	5	26.31579	6.15E-06	CXCL8, CSF2, CXCL1, CXCL3, CXCL2	12
KEGG_PATHWAY	hsa04064:NF-kappa B signaling pathway	5	26.31579	6.64E-06	CXCL8, CXCL1, CXCL3, CXCL2, BIRC3	12
GOTERM_CC_DIRECT	GO:0005615~extracellular space	10	52.63158	1.42E-05	C3, CXCL6, CSF3, CXCL8, CSF2, CCL20, CXCL1, CXCL3, CXCL2, CXCL5	19
KEGG_PATHWAY	hsa04936:Alcoholic liver disease	5	26.31579	2.29E-05	C3, CXCL8, CXCL1, CXCL3, CXCL2	12
UP_KW_CELLULAR_COMPONENT	KW-0964~Secreted	10	52.63158	2.87E-05	C3, CXCL6, CSF3, CXCL8, CSF2, CCL20, CXCL1, CXCL3, CXCL2, CXCL5	17
GOTERM_CC_DIRECT	GO:0005576~extracellular region	10	52.63158	3.28E-05	C3, CXCL6, CSF3, CXCL8, CSF2, CCL20, CXCL1, CXCL3, CXCL2, CXCL5	19
KEGG_PATHWAY	hsa04621:NOD-like receptor signaling pathway	5	26.31579	6.60E-05	CXCL8, CXCL1, CXCL3, CXCL2, BIRC3	12
KEGG_PATHWAY	hsa05120:Epithelial cell signaling in Helicobacter pylori infection	4	21.05263	8.52E-05	CXCL8, CXCL1, CXCL3, CXCL2	12
KEGG_PATHWAY	hsa05133:Pertussis	4	21.05263	1.09E-04	C3, CXCL6, CXCL8, CXCL5	12
KEGG_PATHWAY	hsa05417:Lipid and atherosclerosis	5	26.31579	1.16E-04	CXCL8, CXCL1, CXCL3, POU2F2, CXCL2	12
GOTERM_BP_DIRECT	GO:0007186~G-protein coupled receptor signaling pathway	7	36.84211	1.61E-04	C3, CXCL6, CXCL8, CCL20, CXCL1, CXCL2, CXCL5	19
BIOCARTA	h_stemPathway:Regulation of hematopoiesis by cytokines	3	15.78947	4.74E-04	CSF3, CXCL8, CSF2	5
UP_KW_PTM	KW-1015~Disulfide bond	10	52.63158	7.35E-04	C3, CXCL6, CSF3, CXCL8, CSF2, CCL20, CXCL1, CXCL3, CXCL2, CXCL5	13
GOTERM_BP_DIRECT	GO:0007165~signal transduction	7	36.84211	7.66E-04	C3, CXCL6, CXCL8, ARHGAP40, CCL20, CXCL1, CXCL5	19
BIOCARTA	h_inflamPathway:Cytokines and Inflammatory Response	3	15.78947	0.00181	CSF3, CXCL8, CSF2	5
GOTERM_BP_DIRECT	GO:0006952~defense response	3	15.78947	0.002519	CXCL6, CXCL2, CXCL5	19
GOTERM_BP_DIRECT	GO:0071347~cellular response to interleukin-1	3	15.78947	0.002706	CXCL8, CCL20, ZC3H12A	19
KEGG_PATHWAY	hsa05171:Coronavirus disease - COVID-19	4	21.05263	0.002849	C3, CSF3, CXCL8, CSF2	12
UP_KW_DOMAIN	KW-0732~Signal	10	52.63158	0.004099	C3, CXCL6, CSF3, CXCL8, CSF2, CCL20, CXCL1, CXCL3, CXCL2, CXCL5	14
GOTERM_BP_DIRECT	GO:0071356~cellular response to tumor necrosis factor	3	15.78947	0.007024	CXCL8, CCL20, ZC3H12A	19
UP_SEQ_FEATURE	DOMAIN:PNT	2	10.52632	0.007473	EHF, ELF3	15
GOTERM_MF_DIRECT	GO:0008083~growth factor activity	3	15.78947	0.00963	CSF3, CSF2, CXCL1	18
INTERPRO	IPR003118:Pointed domain	2	10.52632	0.009727	EHF, ELF3	18
GOTERM_BP_DIRECT	GO:0045766~positive regulation of angiogenesis	3	15.78947	0.00993	C3, CXCL8, ZC3H12A	19
GOTERM_BP_DIRECT	GO:0002237~response to molecule of bacterial origin	2	10.52632	0.010154	CXCL8, CXCL2	19
UP_KW_MOLECULAR_FUNCTION	KW-0339~Growth factor	3	15.78947	0.012127	CSF3, CSF2, CXCL1	16
GOTERM_BP_DIRECT	GO:0010884~positive regulation of lipid storage	2	10.52632	0.012907	C3, ZC3H12A	19
GOTERM_BP_DIRECT	GO:0042119~neutrophil activation	2	10.52632	0.015652	CXCL6, CXCL8	19
SMART	SM00251:SAM_PNT	2	10.52632	0.015792	EHF, ELF3	16
GOTERM_BP_DIRECT	GO:0007267~cell-cell signaling	3	15.78947	0.018872	CXCL6, CCL20, CXCL5	19
UP_SEQ_FEATURE	DNA_BIND:ETS	2	10.52632	0.019589	EHF, ELF3	15
GOTERM_BP_DIRECT	GO:0045944~positive regulation of transcription from RNA polymerase II promoter	5	26.31579	0.023502	CSF3, EHF, ELF3, ZC3H12A, POU2F2	19
KEGG_PATHWAY	hsa05202:Transcriptional misregulation in cancer	3	15.78947	0.024843	CXCL8, CSF2, BIRC3	12
INTERPRO	IPR000418:Ets domain	2	10.52632	0.027186	EHF, ELF3	18

UP_SEQ_FEATURE	MOTIF:9aaTAD	2	10.52632	0.028254	ELF3, SP6	15
BIOCARTA	h_granulocytesPathway:Adhesion and Diapedesis of Granulocytes	2	10.52632	0.036492	CSF3, CXCL8	5
BIOCARTA	h_il17Pathway:IL 17 Signaling Pathway	2	10.52632	0.036492	CSF3, CXCL8	5
BIOCARTA	h_erythPathway:Erythrocyte Differentiation Pathway	2	10.52632	0.036492	CSF3, CSF2	5
GOTERM_MF_DIRECT	GO:0043565~sequence-specific DNA binding	3	15.78947	0.036962	EHF, ELF3, POU2F2	18
KEGG_PATHWAY	hsa05131:Shigellosis	3	15.78947	0.039206	C3, CXCL8, CSF2	12
BIOCARTA	h_LairPathway:Cells and Molecules involved in local acute inflammatory response	2	10.52632	0.041282	C3, CXCL8	5
SMART	SM00413:ETS	2	10.52632	0.04391	EHF, ELF3	16
INTERPRO	IPR009079:Four-helical cytokine-like, core	2	10.52632	0.049449	CSF3, CSF2	18
BBID	109.Chemokine_families	3	15.78947	0.058838	CXCL6, CXCL8, CCL20	7
GOTERM_CC_DIRECT	GO:0000785~chromatin	4	21.05263	0.061084	EHF, ELF3, SP6, POU2F2	19
KEGG_PATHWAY	hsa05144:Malaria	2	10.52632	0.063124	CSF3, CXCL8	12
GOTERM_BP_DIRECT	GO:0098586~cellular response to virus	2	10.52632	0.080271	ZC3H12A, POU2F2	19
GOTERM_BP_DIRECT	GO:0010628~positive regulation of gene expression	3	15.78947	0.080438	CXCL8, CSF2, ZC3H12A	19
GOTERM_BP_DIRECT	GO:0008284~positive regulation of cell proliferation	3	15.78947	0.088422	CSF3, CSF2, CXCL5	19
GOTERM_MF_DIRECT	GO:0003700~transcription factor activity, sequence-specific DNA binding	3	15.78947	0.089378	EHF, ELF3, POU2F2	18
GOTERM_BP_DIRECT	GO:0030855~epithelial cell differentiation	2	10.52632	0.089653	EHF, ELF3	19
GOTERM_MF_DIRECT	GO:0000978~RNA polymerase II core promoter proximal region sequence-specific DNA binding	4	21.05263	0.091687	EHF, ELF3, SP6, POU2F2	18

Table S9. Gene transcripts upregulated after LIGHT stimulation in PBS, IL-13, or IL-17 pre-exposed human pulmonary fibroblasts, associated with Fig. 5.

Cytokines preexposed	IL13 pre-exposed Group	IL13_LIGHT.Vs.Untreated_gene_names	gene	IL13.Vs.Untreated:log2FoldChangeShrunken	IL13.Vs.Untreated:padj	LIGHT.Vs.Untreated:log2FoldChangeShrunken	LIGHT.Vs.Untreated:padj	IL13_LIGHT.Vs.Untreated:log2FoldChangeShrunken	IL13_LIGHT.Vs.Untreated:padj
IL13	922	ANOS1	ENSG00000011201.11	4.4	1.4E-24	0.23	0.92	4.71	3E-28
IL13	922	ST8SIA6	ENSG00000148488.15	3.56	7.1E-13	-0.02	0.99	2.67	0.00000014
IL13	922	BATF3	ENSG00000123685.8	3.55	8.5E-80	0.37	0.49	3.99	1.1E-101
IL13	922	MYB	ENSG00000118513.18	2.71	1E-71	-0.23	0.67	2.81	2.1E-77
IL13	922	EHF	ENSG00000135373.12	2.41	2.4E-16	1.7	1.1E-07	4.15	4.5E-49
IL13	922	GPAT3	ENSG00000138678.10	2.1	2.4E-23	-0.27	0.72	1.92	1.6E-19
IL13	922	DSP	ENSG00000096696.13	2.08	8.7E-28	0.67	0.01	1.75	1.2E-19
IL13	922	LRRC8B	ENSG00000197147.13	1.93	4.8E-17	0.63	0.08	2.39	1.6E-26
IL13	922	ARL4C	ENSG00000188042.7	1.88	8.8E-86	0.21	0.29	1.86	4.3E-84
IL13	922	CCDC86	ENSG00000110104.11	1.71	3.9E-50	-0.02	0.98	1.86	4.2E-60
IL13	922	IL1RL1	ENSG00000115602.16	1.66	0.000021	0.38	0.79	3.24	1.7E-21
IL13	922	MMP8	ENSG00000118113.11	1.61	0.034	0.07	2	1.96	0.0032
IL13	922	C1QTNF1	ENSG00000173918.14	1.52	2.9E-36	0.67	1.1E-06	2.21	1.2E-77
IL13	922	KIRREL3	ENSG00000149571.11	1.44	2.2E-23	0.11	0.89	1.72	3.2E-34
IL13	922	PPFIBP2	ENSG00000166387.11	1.41	1.8E-13	-0.52	0.084	1.56	7E-17
IL13	922	SYT9	ENSG00000170743.16	1.39	0.1	1.53	2	1.83	0.013
IL13	922	HAS2	ENSG00000170961.6	1.35	9.8E-06	0.81	0.043	2.07	1E-13
IL13	922	VCAM1	ENSG00000162692.10	1.33	1.6E-13	0.93	2.9E-06	2.01	1E-31
IL13	922	COL6A6	ENSG00000206384.10	1.31	0.052	0.76	0.48	2.32	0.0000098
IL13	922	OLFML2B	ENSG00000162745.10	1.3	3.2E-07	-0.41	0.48	0.96	0.0003
IL13	922	TMEM56	ENSG00000152078.9	1.27	0.061	0.15	0.96	0.9	0.2
IL13	922	ABCG2	ENSG00000118777.10	1.23	0.0066	0.02	0.99	0.66	0.25
IL13	922	COL6A1	ENSG00000142156.14	1.12	4.3E-13	0.27	0.43	1.36	1.4E-19
IL13	922	SMCO2	ENSG00000165935.9	1.1	0.052	1.37	0.0073	2.03	0.0000013
IL13	922	LRRC8C	ENSG00000171488.14	1.06	1.6E-07	0.34	0.42	0.94	0.0000038
IL13	922	LIFR	ENSG00000113594.9	1.04	0.00096	0.17	0.9	1.26	0.0000087
IL13	922	IL18RAP	ENSG00000115607.9	0.95	2	0.19	2	1.48	2
IL13	922	RNF19B	ENSG00000116514.16	0.95	4E-15	0.72	3.6E-08	1.85	4.7E-60
IL13	922	COL7A1	ENSG00000114270.17	0.9	2.7E-08	0.26	0.49	0.69	0.000035
IL13	922	SLIT2	ENSG00000145147.19	0.9	0.0016	0.47	0.33	1.37	0.00000002
IL13	922	PTPRE	ENSG00000132334.16	0.88	0.000082	0.4	0.29	1.14	0.000000022
IL13	922	GBP4	ENSG00000162654.8	0.86	0.024	1.11	0.0013	2.29	2.3E-16
IL13	922	PTGER4	ENSG00000171522.5	0.86	3.5E-06	0.7	0.00074	1.96	1.9E-34
IL13	922	SERPINB2	ENSG00000197632.8	0.82	6.9E-07	0.24	0.54	1.49	6E-24
IL13	922	TNFRSF9	ENSG00000049249.8	0.75	0.062	0.42	0.57	1.59	0.000000012
IL13	922	ADAP1	ENSG00000105963.13	0.75	0.018	-0.09	0.95	0.72	0.015
IL13	922	NDP	ENSG00000124479.8	0.74	0.34	-0.45	0.75	0.39	0.64
IL13	922	XAF1	ENSG00000132530.16	0.74	0.041	1.06	0.00046	1.39	0.00000016
IL13	922	RGMB	ENSG00000174136.11	0.72	6.9E-07	0.8	5.5E-08	1.31	3.2E-23
IL13	922	IL18R1	ENSG00000115604.10	0.71	0.026	0.65	0.06	2.13	4.5E-22
IL13	922	AMD1	ENSG00000123505.15	0.71	0.00002	0.3	0.29	0.76	0.0000013
IL13	922	STAR	ENSG00000147465.11	0.71	0.035	-0.08	0.96	1.67	1.4E-12
IL13	922	ARL4A	ENSG00000122644.12	0.7	0.00054	0.09	0.93	1.15	2E-11
IL13	922	TSLP	ENSG00000145777.14	0.7	0.096	0.7	0.099	1.42	0.00000041
IL13	922	C3orf36	ENSG00000221972.3	0.7	0.55	0.67	2	1.6	0.028
IL13	922	SPDL1	ENSG00000040275.16	0.69	0.015	0.62	0.05	0.82	0.00084
IL13	922	COL4A4	ENSG00000081052.11	0.69	0.44	0.41	0.79	2.82	5.2E-13
IL13	922	LYPD6	ENSG00000187123.14	0.69	0.51	0.43	0.79	0.81	0.21
IL13	922	LRRC8D	ENSG00000171492.14	0.68	0.000008	0.16	0.74	0.87	6.5E-10
IL13	922	SSTR2	ENSG00000180616.8	0.67	0.49	0.94	0.22	2.05	0.00079
IL13	922	PCSK9	ENSG00000169174.10	0.66	0.6	0.65	2	0.86	0.25
IL13	922	FAM111B	ENSG00000189057.10	0.65	0.46	1.45	0.0018	1.4	0.0011
IL13	922	KRT18	ENSG00000111057.10	0.63	0.14	-0.75	0.049	0.21	0.75
IL13	922	SLC39A8	ENSG00000138821.12	0.63	0.000044	0.2	0.59	1	4.1E-13
IL13	922	SIPA1L3	ENSG00000105738.10	0.62	0.13	0.42	0.51	0.79	0.012
IL13	922	GATA6	ENSG00000141448.8	0.62	0.00041	0.17	0.75	1.03	6.2E-12
IL13	922	ADCY1	ENSG00000164742.14	0.62	0.5	0.91	0.13	1.69	0.000024
IL13	922	NIPA1	ENSG00000170113.15	0.62	0.026	0.16	0.88	0.7	0.0035
IL13	922	GJD3	ENSG00000183153.6	0.6	0.38	0.3	0.83	0.81	0.055

IL13	922	EDNRB	ENSG00000136160.14	0.59	0.48	0.26	0.88	0.58	0.33
IL13	922	KCNQ5	ENSG00000185760.15	0.59	0.4	0.64	0.31	0.75	0.096
IL13	922	AL355607.2	ENSG00000260454.1	0.59	0.33	1.9	0.0011	1.9	0.00041
IL13	922	SAMD10	ENSG00000130590.13	0.58	0.0038	0.5	0.027	1.26	1.1E-15
IL13	922	CLDN16	ENSG00000113946.3	0.57	0.67	1.65	0.0012	1.21	0.02
IL13	922	INTS6L	ENSG00000165359.15	0.57	0.03	0.17	0.84	0.49	0.055
IL13	922	MIR222HG	ENSG00000270069.1	0.57	0.058	1.03	9.1E-06	1.05	0.0000016
IL13	922	VEPH1	ENSG00000197415.11	0.56	0.027	0.34	0.42	0.84	0.000035
IL13	922	SFMBT1	ENSG00000163935.13	0.55	0.39	0.04	0.99	0.85	0.02
IL13	922	RNF207	ENSG00000158286.12	0.54	0.27	0.61	0.17	1.15	0.000043
IL13	922	FRMD4A	ENSG00000151474.21	0.53	0.015	0.1	0.91	0.49	0.015
IL13	922	HOPX	ENSG00000171476.21	0.53	0.67	0.1	0.97	1.12	0.022
IL13	922	CDC7	ENSG00000097046.12	0.52	0.49	0.43	0.63	0.64	0.16
IL13	922	NLRP10	ENSG00000182261.3	0.52	0.87	-0.24	2	0.27	0.88
IL13	922	PSME2P2	ENSG00000225131.2	0.52	0.51	0.47	0.57	0.72	0.1
IL13	922	PDCD1LG2	ENSG00000197646.7	0.51	0.029	0.7	0.00071	1.04	4.5E-09
IL13	922	CCDC18	ENSG00000122483.17	0.5	0.38	0.44	0.49	0.58	0.13
IL13	922	SLCO5A1	ENSG00000137571.10	0.5	0.75	1.1	0.1	1.49	0.005
IL13	922	CXCL5	ENSG00000163735.6	0.5	0.2	1.47	4.7E-10	1.62	5.1E-13
IL13	922	NKX3-1	ENSG00000167034.9	0.5	0.039	0.38	0.21	0.74	0.000087
IL13	922	EZH2	ENSG00000106462.10	0.49	0.065	0.34	0.37	0.58	0.007
IL13	922	CXCL3	ENSG00000163734.4	0.49	0.17	1.21	1E-07	1.57	7.5E-14
IL13	922	CYP7B1	ENSG00000172817.3	0.49	0.8	1.1	0.15	1.65	0.0031
IL13	922	TNFRSF8	ENSG00000120949.14	0.48	0.78	0.7	0.48	1.38	0.005
IL13	922	EFHD2	ENSG00000142634.12	0.48	0.2	0.29	0.64	0.78	0.0014
IL13	922	FANCM	ENSG00000187790.10	0.48	0.15	0.29	0.63	0.6	0.019
IL13	922	SLC22A4	ENSG00000197208.5	0.48	0.0054	0.61	0.00011	0.85	6.9E-10
IL13	922	COL11A2	ENSG00000204248.10	0.48	0.42	-0.29	0.79	0.24	0.72
IL13	922	AC005831.1	ENSG00000280202.1	0.48	0.72	-0.06	0.98	0.46	0.54
IL13	922	FAS	ENSG00000026103.21	0.47	0.031	0.33	0.27	1.1	3.9E-12
IL13	922	BRICD5	ENSG00000182685.7	0.47	0.38	0.26	0.8	0.04	0.97
IL13	922	AC006058.1	ENSG00000261786.1	0.47	0.83	0.76	0.47	0.45	0.66
IL13	922	ATG16L2	ENSG00000168010.10	0.46	0.014	0.21	0.59	0.46	0.0062
IL13	922	ZCCHC6	ENSG00000083223.17	0.45	0.55	0.62	0.24	0.77	0.037
IL13	922	ZNF726	ENSG00000213967.10	0.45	0.85	0.44	2	0.74	0.38
IL13	922	HELLS	ENSG00000119969.14	0.44	0.51	0.51	0.37	0.71	0.042
IL13	922	IDO1	ENSG00000131203.12	0.44	0.72	0.73	2	0.49	0.48
IL13	922	MIR3142HG	ENSG00000253522.5	0.44	0.41	0.4	0.5	0.55	0.11
IL13	922	KCNK2	ENSG00000082482.13	0.43	0.63	0.07	0.97	0.87	0.02
IL13	922	CLSPN	ENSG00000092853.13	0.43	0.64	1.28	0.00023	0.72	0.081
IL13	922	PIM2	ENSG00000102096.9	0.43	0.012	0.07	0.92	0.46	0.0023
IL13	922	NR6A1	ENSG00000148200.16	0.43	0.64	-0.12	0.94	0.46	0.39
IL13	922	OLR1	ENSG00000173391.8	0.43	0.63	0.34	0.76	1	0.004
IL13	922	HSP90AA2P	ENSG00000224411.3	0.43	0.82	0.72	0.41	0.41	0.65
IL13	922	BRCA1	ENSG00000012048.20	0.42	0.61	0.6	0.26	0.7	0.073
IL13	922	ELMO3	ENSG00000102890.14	0.42	0.59	0.36	0.67	0.62	0.11
IL13	922	PLOD2	ENSG00000152952.11	0.42	0.34	0.64	0.036	0.89	0.00019
IL13	922	CKAP2L	ENSG00000169607.12	0.42	0.61	0.44	0.55	0.27	0.69
IL13	922	ZDHHC23	ENSG00000184307.14	0.42	0.68	0.63	0.28	0.96	0.0082
IL13	922	CSF1	ENSG00000184371.13	0.42	0.0057	0.56	0.000073	0.87	9.9E-13
IL13	922	LINC00664	ENSG00000268658.5	0.42	0.91	0.52	2	0.05	0.98
IL13	922	AC015912.3	ENSG00000274213.1	0.42	0.91	0.33	0.91	0.34	0.81
IL13	922	SLC4A7	ENSG00000033867.16	0.41	0.35	0.88	0.00077	0.75	0.0027
IL13	922	SRSF7	ENSG00000115875.18	0.41	0.072	0.35	0.2	0.61	0.00042
IL13	922	DEPDC1B	ENSG00000035499.12	0.4	0.79	0.14	0.95	0.53	0.4
IL13	922	EIF4A1	ENSG00000161960.14	0.4	0.29	0.19	0.83	0.51	0.051
IL13	922	SLFN11	ENSG00000172716.16	0.4	0.11	0.88	4.2E-07	1.24	3.9E-15
IL13	922	EXO1	ENSG00000174371.16	0.4	0.7	1.13	0.0016	0.58	0.22
IL13	922	KDM4A-AS1	ENSG00000236200.5	0.4	0.79	0.95	0.069	1.24	0.002
IL13	922	OSBPL6	ENSG00000079156.16	0.39	0.16	0.1	0.92	0.27	0.35
IL13	922	LYAR	ENSG00000145220.13	0.39	0.47	0.25	0.77	0.52	0.098
IL13	922	MFHAS1	ENSG00000147324.10	0.39	0.26	0.3	0.54	0.84	0.000034
IL13	922	ELF3	ENSG00000163435.15	0.39	0.73	0.12	0.95	0.78	0.063
IL13	922	USP49	ENSG00000164663.14	0.39	0.53	0.46	0.36	0.66	0.036
IL13	922	FLRT2	ENSG00000185070.10	0.39	0.37	0.07	0.95	0.69	0.0042

IL13	922	ORC6	ENSG00000091651.8	0.37	0.33	0.39	0.27	0.31	0.32
IL13	922	CGNL1	ENSG00000128849.10	0.37	0.68	-0.37	0.65	0.21	0.76
IL13	922	SNRPA1	ENSG00000131876.16	0.37	0.12	0.52	0.008	0.59	0.00054
IL13	922	SULF1	ENSG00000137573.13	0.37	0.59	0.32	0.67	0.88	0.0019
IL13	922	ZGRF1	ENSG00000138658.15	0.37	0.79	0.1	0.96	0.31	0.67
IL13	922	EAF2	ENSG00000145088.8	0.37	0.9	-0.22	0.92	-0.13	0.93
IL13	922	TRIM36	ENSG00000152503.9	0.37	0.86	0.65	0.46	0.15	0.92
IL13	922	KCNA3	ENSG00000177272.8	0.37	0.89	0.33	0.88	0.52	0.55
IL13	922	CDC6	ENSG00000094804.9	0.36	0.46	0.7	0.0084	0.67	0.0066
IL13	922	UCHL3	ENSG00000118939.17	0.36	0.81	0.12	0.95	0.5	0.41
IL13	922	SELENOI	ENSG00000138018.17	0.36	0.52	0.44	0.33	0.39	0.28
IL13	922	TAP2	ENSG00000204267.13	0.36	0.053	0.7	5.9E-07	0.74	0.00000002
IL13	922	LINC01204	ENSG00000229563.6	0.36	0.89	0.38	0.83	0.8	0.19
IL13	922	C17orf49	ENSG00000258315.5	0.36	0.71	0.87	0.017	1	0.0012
IL13	922	MIR4479	ENSG00000266507.1	0.36	0.92	0.95	0.34	1.09	0.12
IL13	922	GABRQ	ENSG00000268089.2	0.36	0.92	0.5	0.78	0.02	1
IL13	922	GALNT1	ENSG00000141429.13	0.35	0.69	0.44	0.49	0.64	0.072
IL13	922	CXCL8	ENSG00000169429.10	0.35	0.6	1.42	1.8E-08	1.33	0.000000055
IL13	922	ZNF788	ENSG00000214189.8	0.35	0.49	0.54	0.086	0.42	0.18
IL13	922	AC018690.1	ENSG00000273306.1	0.35	0.94	0.29	0.91	0.67	0.47
IL13	922	NOP58	ENSG00000055044.10	0.34	0.56	0.31	0.63	0.47	0.14
IL13	922	C1GALT1	ENSG00000106392.10	0.34	0.23	0.24	0.56	0.54	0.0039
IL13	922	SMC4	ENSG00000113810.15	0.34	0.54	0.21	0.81	0.41	0.2
IL13	922	PLS1	ENSG00000120756.12	0.34	0.81	0.37	0.74	0.31	0.67
IL13	922	HEATR3	ENSG00000155393.12	0.34	0.033	0.07	0.92	0.44	0.00075
IL13	922	SIX2	ENSG00000170577.7	0.34	0.79	-0.62	0.37	-0.16	0.88
IL13	922	PRSS36	ENSG00000178226.10	0.34	0.91	1.21	0.041	1.23	0.017
IL13	922	SELENOT	ENSG00000198843.12	0.34	0.13	0.27	0.33	0.72	0.00000099
IL13	922	DPF1	ENSG00000011332.19	0.33	0.88	-0.58	0.53	0.1	0.94
IL13	922	TARBP1	ENSG00000059588.9	0.33	0.28	0.27	0.49	0.39	0.073
IL13	922	ST8SIA4	ENSG00000113532.12	0.33	0.96	-0.14	2	0.39	0.79
IL13	922	DONSON	ENSG00000159147.17	0.33	0.46	0.23	0.71	0.32	0.31
IL13	922	NET1	ENSG00000173848.18	0.33	0.32	0.34	0.29	0.62	0.0012
IL13	922	GEN1	ENSG00000178295.14	0.33	0.63	0.26	0.75	0.25	0.61
IL13	922	LINC00595	ENSG00000230417.11	0.33	0.95	-0.05	2	0.76	0.4
IL13	922	MMP19	ENSG00000123342.15	0.32	0.2	0.72	4.5E-06	1.23	2.1E-19
IL13	922	CHAC2	ENSG00000143942.4	0.32	0.67	0.16	0.9	0.47	0.17
IL13	922	C5orf34	ENSG00000172244.8	0.32	0.79	0.68	0.13	0.62	0.12
IL13	922	ATR	ENSG00000175054.14	0.32	0.76	0.46	0.48	0.54	0.18
IL13	922	COL27A1	ENSG00000196739.14	0.32	0.087	0.73	4.7E-08	0.81	6.7E-11
IL13	922	AC025449.1	ENSG00000270558.1	0.32	0.95	-0.09	0.98	0.38	0.76
IL13	922	CEP152	ENSG00000103995.13	0.31	0.85	0.45	0.62	0.34	0.61
IL13	922	CD58	ENSG00000116815.15	0.31	0.094	0.22	0.42	0.41	0.0047
IL13	922	KIAA1524	ENSG00000163507.13	0.31	0.7	0.49	0.28	0.61	0.046
IL13	922	TP73	ENSG00000078900.14	0.3	2	0.29	2	0.2	2
IL13	922	SLITRK2	ENSG00000185985.8	0.3	2	0.4	2	-0.02	2
IL13	922	ARHGAP11B	ENSG00000187951.10	0.3	0.78	0.52	0.31	0.41	0.37
IL13	922	PRR22	ENSG00000212123.3	0.3	0.94	0.32	0.88	0.07	0.97
IL13	922	PSME2	ENSG00000100911.15	0.29	0.64	0.47	0.18	0.87	0.00015
IL13	922	NEFM	ENSG00000104722.13	0.29	0.94	-0.87	0.3	-0.61	0.44
IL13	922	LRP8	ENSG00000157193.15	0.29	0.77	0.29	0.74	0.54	0.13
IL13	922	POGLUT1	ENSG00000163389.10	0.29	0.42	0.11	0.89	0.47	0.022
IL13	922	KIF15	ENSG00000163808.16	0.29	0.82	0.42	0.56	0.31	0.59
IL13	922	CDC25A	ENSG00000164045.11	0.29	0.59	0.04	0.98	0.28	0.41
IL13	922	ABTB2	ENSG00000166016.5	0.29	0.47	0.3	0.44	0.34	0.2
IL13	922	NRROS	ENSG00000174004.5	0.29	0.91	0.37	0.78	0.41	0.57
IL13	922	BORA	ENSG00000136122.15	0.28	0.79	0.04	0.98	0.49	0.19
IL13	922	PARP1	ENSG00000143799.12	0.28	0.034	-0.01	0.99	0.42	0.000082
IL13	922	FICD	ENSG00000198855.6	0.28	0.1	0.37	0.012	0.59	0.00000028
IL13	922	CCNE1	ENSG00000105173.13	0.27	0.29	-0.03	0.97	0.2	0.4
IL13	922	NAA25	ENSG00000111300.9	0.27	0.72	0.37	0.45	0.38	0.26
IL13	922	SMC2	ENSG00000136824.18	0.27	0.83	0.34	0.68	0.38	0.42
IL13	922	ADAMTS3	ENSG00000156140.9	0.27	0.7	-0.14	0.89	-0.01	0.99
IL13	922	KBTBD8	ENSG00000163376.11	0.27	0.91	0.76	0.16	0.19	0.85
IL13	922	TMEM200A	ENSG00000164484.11	0.27	0.22	0.13	0.78	0.58	0.000014

IL13	922	FABP4	ENSG00000170323.8	0.27	0.98	-0.78	2	-0.73	0.55
IL13	922	RPS27AP16	ENSG00000224631.4	0.27	0.96	-0.35	0.85	0.47	0.6
IL13	922	HSP90B2P	ENSG00000259706.1	0.27	0.96	0.17	0.94	0.57	0.49
IL13	922	POLE2	ENSG00000100479.12	0.26	0.91	0.14	0.94	0.3	0.7
IL13	922	CCP110	ENSG00000103540.16	0.26	0.77	0.14	0.91	0.16	0.79
IL13	922	FBXO5	ENSG00000112029.9	0.26	0.63	0.12	0.9	0.31	0.3
IL13	922	ECE2	ENSG00000145194.17	0.26	0.81	0.07	0.96	0.18	0.79
IL13	922	SUV39H2	ENSG00000152455.15	0.26	0.71	0.18	0.83	0.28	0.46
IL13	922	MLKL	ENSG00000168404.12	0.26	0.69	0.41	0.28	0.55	0.031
IL13	922	KRT15	ENSG00000171346.15	0.26	0.78	0.06	0.97	0.14	0.83
IL13	922	ZWILCH	ENSG00000174442.11	0.26	0.79	0.36	0.55	0.65	0.026
IL13	922	AC016397.2	ENSG00000279822.1	0.26	0.91	0.77	0.1	0.4	0.5
IL13	922	CEP128	ENSG00000100629.16	0.25	0.92	0.32	0.81	0.08	0.94
IL13	922	BIRC2	ENSG00000110330.8	0.25	0.76	0.53	0.11	0.54	0.047
IL13	922	SLF1	ENSG00000133302.12	0.25	0.9	0.3	0.78	0.32	0.6
IL13	922	PIF1	ENSG00000140451.12	0.25	0.42	0.4	0.04	0.42	0.012
IL13	922	ANKLE1	ENSG00000160117.14	0.25	0.86	0.02	0.99	0.39	0.4
IL13	922	KPNA2	ENSG00000182481.8	0.25	0.016	0.34	0.00024	0.74	7.5E-22
IL13	922	BRI3BP	ENSG00000184992.10	0.25	0.73	-0.01	1	0.14	0.81
IL13	922	ZNF195	ENSG00000005801.16	0.24	0.68	0.19	0.78	0.22	0.54
IL13	922	CYLD	ENSG00000083799.17	0.24	0.7	0.49	0.096	0.5	0.037
IL13	922	DMC1	ENSG00000100206.9	0.24	2	0.35	2	0.15	2
IL13	922	SLC17A9	ENSG00000101194.17	0.24	0.74	0.18	0.84	0.31	0.38
IL13	922	CDCA7	ENSG00000144354.13	0.24	0.88	0.12	0.94	0.34	0.5
IL13	922	DSN1	ENSG00000149636.15	0.24	0.78	0.06	0.97	0.14	0.82
IL13	922	TDO2	ENSG00000151790.8	0.24	0.97	0.46	0.79	2.62	0.000000042
IL13	922	MAT2A	ENSG00000168906.12	0.24	0.0064	-0.13	0.41	0.25	0.0023
IL13	922	APLN	ENSG00000171388.11	0.24	0.87	-0.72	0.079	0.26	0.65
IL13	922	AFF1	ENSG00000172493.20	0.24	0.86	0.32	0.7	0.38	0.39
IL13	922	CEP83	ENSG00000173588.14	0.24	0.74	0.37	0.37	0.41	0.16
IL13	922	NEIL3	ENSG00000109674.3	0.23	0.97	0.51	0.69	-0.07	0.96
IL13	922	GPR68	ENSG00000119714.10	0.23	0.86	1.57	1.1E-10	1.52	1.5E-10
IL13	922	PKMYT1	ENSG00000127564.16	0.23	0.9	0.53	0.32	0.09	0.93
IL13	922	ZNF714	ENSG00000160352.15	0.23	0.56	-0.27	0.41	-0.07	0.89
IL13	922	BNC1	ENSG00000169594.13	0.23	0.74	0.2	0.79	0.64	0.0047
IL13	922	EMC3-AS1	ENSG00000180385.8	0.23	0.87	-0.17	0.89	0.2	0.76
IL13	922	LIN9	ENSG00000183814.15	0.23	0.82	0.03	0.99	0.33	0.36
IL13	922	KDM4D	ENSG00000186280.6	0.23	0.91	-0.12	0.94	-0.07	0.95
IL13	922	SNORD46	ENSG00000200913.1	0.23	0.97	-0.01	1	0.39	0.74
IL13	922	REXO5	ENSG00000005189.19	0.22	0.8	-0.13	0.9	0.38	0.21
IL13	922	DBF4	ENSG00000006634.7	0.22	0.85	0.36	0.52	0.57	0.056
IL13	922	BID	ENSG00000015475.18	0.22	0.8	0.09	0.94	0.36	0.25
IL13	922	RAD18	ENSG00000070950.9	0.22	0.71	0.21	0.71	0.27	0.39
IL13	922	PSMA3	ENSG00000100567.12	0.22	0.89	0.18	0.88	0.45	0.25
IL13	922	TLE4	ENSG00000106829.18	0.22	0.58	-0.1	0.89	0.23	0.37
IL13	922	DNMT1	ENSG00000130816.14	0.22	0.34	0.22	0.34	0.48	0.00014
IL13	922	NASP	ENSG00000132780.16	0.22	0.61	0.25	0.48	0.28	0.21
IL13	922	CKAP2	ENSG00000136108.14	0.22	0.85	0.22	0.82	0.16	0.79
IL13	922	GAS2L3	ENSG00000139354.10	0.22	0.89	0.47	0.33	0.44	0.24
IL13	922	MANF	ENSG00000145050.15	0.22	0.84	0.06	0.96	0.45	0.14
IL13	922	CENPJ	ENSG00000151849.14	0.22	0.93	0.43	0.59	0.42	0.43
IL13	922	FAM111A	ENSG00000166801.15	0.22	0.87	0.7	0.04	0.49	0.17
IL13	922	CSPG4	ENSG00000173546.7	0.22	0.7	0.16	0.83	0.3	0.29
IL13	922	PSMB10	ENSG00000205220.11	0.22	0.72	0.66	0.0025	0.74	0.00013
IL13	922	SNHG15	ENSG00000232956.8	0.22	0.92	0.06	0.97	0.19	0.82
IL13	922	MSH5-SAPCD1	ENSG00000255152.8	0.22	0.98	0.07	2	0.09	0.96
IL13	922	AP005233.2	ENSG00000260877.2	0.22	0.98	-0.35	2	0.06	0.98
IL13	922	CARD8-AS1	ENSG00000268001.1	0.22	0.91	0.56	0.26	0.74	0.027
IL13	922	PALB2	ENSG00000083093.9	0.21	0.83	0.23	0.73	0.21	0.63
IL13	922	RBBP8	ENSG00000101773.18	0.21	0.88	0.51	0.2	0.67	0.016
IL13	922	TNFSF13B	ENSG00000102524.11	0.21	0.96	0.28	0.86	0.34	0.67
IL13	922	AUNIP	ENSG00000127423.10	0.21	0.97	0.22	0.92	0.29	0.8
IL13	922	SASS6	ENSG00000156876.9	0.21	0.88	0.28	0.72	0.08	0.93
IL13	922	PCLAF	ENSG00000166803.11	0.21	0.94	0.51	0.47	0.19	0.84
IL13	922	ATAD5	ENSG00000176208.8	0.21	0.94	0.27	0.84	0.38	0.52

IL13	922	KLHL23	ENSG00000213160.9	0.21	0.91	-0.26	0.8	-0.03	0.98
IL13	922	PDCD5	ENSG00000105185.11	0.2	0.8	0.22	0.73	0.39	0.15
IL13	922	ECT2	ENSG00000114346.13	0.2	0.83	0.56	0.04	0.64	0.0047
IL13	922	DTL	ENSG00000143476.17	0.2	0.91	0.98	0.00066	0.46	0.24
IL13	922	NUP35	ENSG00000163002.12	0.2	0.87	0.36	0.48	0.13	0.84
IL13	922	ZNF367	ENSG00000165244.6	0.2	0.94	0.8	0.055	0.65	0.11
IL13	922	KNTC1	ENSG00000184445.11	0.2	0.9	0.28	0.74	0.08	0.93
IL13	922	RAD54B	ENSG00000197275.13	0.2	0.98	0.25	0.91	0.18	0.89
IL13	922	SCML1	ENSG00000047634.14	0.19	0.76	0.2	0.71	0.29	0.27
IL13	922	NOP16	ENSG00000048162.20	0.19	0.9	0.06	0.97	0.25	0.58
IL13	922	MCM4	ENSG00000104738.16	0.19	0.53	0.21	0.45	0.17	0.43
IL13	922	MASTL	ENSG00000120539.14	0.19	0.61	0.35	0.055	0.4	0.0081
IL13	922	DNAJB9	ENSG00000128590.4	0.19	0.67	0.29	0.27	0.53	0.00065
IL13	922	IL17REL	ENSG00000188263.10	0.19	0.98	-0.43	0.83	0.25	0.85
IL13	922	MSTO2P	ENSG00000203761.5	0.19	0.96	0.31	0.83	0.62	0.23
IL13	922	HLA-F	ENSG00000204642.13	0.19	0.8	0.8	0.000048	0.91	0.00000041
IL13	922	CNTF	ENSG00000242689.2	0.19	0.98	-0.08	0.98	-0.26	0.85
IL13	922	CD24	ENSG00000272398.5	0.19	0.98	1.49	0.028	0.38	0.75
IL13	922	AL391988.1	ENSG00000277879.1	0.19	0.96	0.04	0.99	0.49	0.3
IL13	922	SEC23B	ENSG00000101310.14	0.18	0.18	0.14	0.47	0.34	0.0001
IL13	922	TIMELESS	ENSG00000111602.11	0.18	0.59	0.29	0.14	0.11	0.73
IL13	922	TRIM6	ENSG00000121236.20	0.18	0.88	-0.33	0.49	-0.21	0.62
IL13	922	MCM8	ENSG00000125885.13	0.18	0.91	0.12	0.92	0.19	0.72
IL13	922	PPAT	ENSG00000128059.8	0.18	0.89	0.29	0.61	0.25	0.55
IL13	922	CEP85	ENSG00000130695.14	0.18	0.81	-0.04	0.97	0.12	0.8
IL13	922	TOE1	ENSG00000132773.11	0.18	0.86	-0.1	0.92	0.3	0.35
IL13	922	KIF20B	ENSG00000138182.14	0.18	0.92	0.38	0.51	0.35	0.4
IL13	922	FANCI	ENSG00000140525.17	0.18	0.94	0.33	0.7	0.13	0.88
IL13	922	NCAPD3	ENSG00000151503.12	0.18	0.89	-0.01	1	0.24	0.54
IL13	922	SLC2A6	ENSG00000160326.13	0.18	0.51	0.66	1.1E-08	0.69	2.8E-10
IL13	922	C16orf59	ENSG00000162062.14	0.18	0.94	0.22	0.85	0.26	0.63
IL13	922	FABP5	ENSG00000164687.10	0.18	0.96	0.1	0.96	0.13	0.9
IL13	922	PGM2	ENSG00000169299.13	0.18	0.83	0.25	0.6	0.41	0.092
IL13	922	CHRNA5	ENSG00000169684.13	0.18	0.98	0.41	0.83	0.6	0.46
IL13	922	ANXA2	ENSG00000182718.16	0.18	0.86	0.12	0.9	0.58	0.0074
IL13	922	ZNF100	ENSG00000197020.10	0.18	0.94	0.48	0.41	0.28	0.64
IL13	922	C4orf46	ENSG00000205208.4	0.18	0.79	0.16	0.8	0.35	0.13
IL13	922	PICALM	ENSG00000073921.17	0.17	0.89	0.31	0.55	0.45	0.098
IL13	922	WDR76	ENSG00000092470.11	0.17	0.9	0.47	0.15	0.2	0.67
IL13	922	CNTNAP1	ENSG00000108797.11	0.17	0.96	0.06	0.97	0.18	0.81
IL13	922	SRSF2	ENSG00000161547.16	0.17	0.56	0.3	0.064	0.4	0.0015
IL13	922	ZC3H12A	ENSG00000163874.10	0.17	0.83	0.36	0.21	0.55	0.004
IL13	922	UTP15	ENSG00000164338.9	0.17	0.89	0.55	0.041	0.43	0.1
IL13	922	MFSD2A	ENSG00000168389.17	0.17	0.91	0.43	0.22	0.65	0.0048
IL13	922	NOC3L	ENSG00000173145.11	0.17	0.9	0.34	0.47	0.43	0.14
IL13	922	SCFD2	ENSG00000184178.15	0.17	0.64	0.02	0.98	0.23	0.2
IL13	922	SNHG12	ENSG00000197989.13	0.17	0.77	0.27	0.4	0.32	0.14
IL13	922	IKBKE	ENSG00000263528.7	0.17	0.69	-0.06	0.93	0.35	0.039
IL13	922	MCM6	ENSG00000076003.4	0.16	0.75	0.1	0.87	0.31	0.094
IL13	922	PUS7	ENSG00000091127.13	0.16	0.86	0.26	0.54	0.38	0.1
IL13	922	RFC5	ENSG00000111445.13	0.16	0.86	-0.18	0.77	0.07	0.9
IL13	922	DKC1	ENSG00000130826.17	0.16	0.64	0.15	0.66	0.28	0.068
IL13	922	TUBB2B	ENSG00000137285.9	0.16	0.93	-0.07	0.95	0.11	0.87
IL13	922	RABGGTB	ENSG00000137955.15	0.16	0.91	0.24	0.69	0.21	0.59
IL13	922	PNPT1	ENSG00000138035.14	0.16	0.9	0.31	0.52	0.26	0.46
IL13	922	DNA2	ENSG00000138346.14	0.16	0.97	0.11	0.95	0.24	0.78
IL13	922	ATP5G1	ENSG00000159199.13	0.16	0.94	0.03	0.98	0.17	0.77
IL13	922	HSPA4L	ENSG00000164070.11	0.16	0.95	0.14	0.92	0.12	0.88
IL13	922	NOLC1	ENSG00000166197.16	0.16	0.67	0.27	0.17	0.32	0.029
IL13	922	SNCAIP	ENSG00000064692.18	0.15	0.96	-0.22	0.83	0.02	0.98
IL13	922	ENO1	ENSG00000074800.13	0.15	0.77	0.05	0.95	0.42	0.0037
IL13	922	TIGAR	ENSG00000078237.6	0.15	0.73	0.09	0.87	0.15	0.53
IL13	922	MYEF2	ENSG00000104177.17	0.15	0.94	0.49	0.18	0.41	0.21
IL13	922	FARSB	ENSG00000116120.9	0.15	0.51	0.09	0.79	0.27	0.013
IL13	922	GADD45A	ENSG00000116717.11	0.15	0.9	0.15	0.85	0.61	0.0019

IL13	922	TUBG1	ENSG00000131462.7	0.15	0.83	0.02	0.99	0.25	0.3
IL13	922	LTV1	ENSG00000135521.8	0.15	0.93	0.29	0.62	0.36	0.27
IL13	922	TPMT	ENSG00000137364.4	0.15	0.85	0.34	0.16	0.52	0.002
IL13	922	ANP32E	ENSG00000143401.14	0.15	0.91	0.18	0.8	0.25	0.46
IL13	922	MIS18A	ENSG00000159055.3	0.15	0.91	0.08	0.94	0.17	0.71
IL13	922	ZG16B	ENSG00000162078.11	0.15	0.97	0.23	0.85	0.31	0.58
IL13	922	TNFAIP2	ENSG00000185215.8	0.15	0.94	0.24	0.75	0.44	0.16
IL13	922	PARPBP	ENSG00000185480.11	0.15	0.95	-0.02	0.99	0.05	0.95
IL13	922	AC078785.1	ENSG00000240057.5	0.15	0.98	0.42	0.67	0.11	0.93
IL13	922	AC087623.4	ENSG00000272159.1	0.15	0.99	0.13	2	0.21	0.9
IL13	922	TIPIN	ENSG00000075131.9	0.14	0.96	0.12	0.93	0.3	0.52
IL13	922	UBE2T	ENSG00000077152.9	0.14	0.97	0.07	0.97	0.43	0.37
IL13	922	VRK1	ENSG00000100749.7	0.14	0.89	0.08	0.93	0.15	0.66
IL13	922	SPAG1	ENSG00000104450.12	0.14	0.96	0.34	0.56	0.55	0.067
IL13	922	XRCC3	ENSG00000126215.13	0.14	0.89	-0.08	0.92	0.16	0.66
IL13	922	PARP2	ENSG00000129484.13	0.14	0.89	0.26	0.49	0.17	0.62
IL13	922	KIF1A	ENSG00000130294.15	0.14	2	0.53	2	0.29	2
IL13	922	URB2	ENSG00000135763.9	0.14	0.96	0.12	0.92	0.17	0.77
IL13	922	GPATCH4	ENSG00000160818.16	0.14	0.94	0.14	0.89	0.35	0.32
IL13	922	NAA15	ENSG00000164134.12	0.14	0.94	0.33	0.46	0.33	0.3
IL13	922	NAV2	ENSG00000166833.19	0.14	0.97	0.46	0.52	0.21	0.79
IL13	922	NANP	ENSG00000170191.4	0.14	0.91	0	1	0.27	0.39
IL13	922	SERPINB9	ENSG00000170542.5	0.14	0.91	-0.21	0.7	0.11	0.81
IL13	922	POLR1C	ENSG00000171453.17	0.14	0.92	0.02	0.99	0.14	0.76
IL13	922	TUBB4B	ENSG00000188229.5	0.14	0.88	0.09	0.92	0.44	0.023
IL13	922	AC097461.1	ENSG00000261428.2	0.14	0.99	-0.23	0.92	0.17	0.9
IL13	922	NME1-NME2	ENSG00000011052.21	0.13	0.98	-0.01	1	0.29	0.71
IL13	922	CXCL2	ENSG00000081041.8	0.13	0.96	1.15	4.8E-07	1.13	0.00000025
IL13	922	AURKA	ENSG00000087586.17	0.13	0.91	0.17	0.79	0.24	0.39
IL13	922	NUP107	ENSG00000111581.9	0.13	0.93	0.28	0.56	0.3	0.32
IL13	922	PSMD14	ENSG00000115233.11	0.13	0.84	0.24	0.38	0.3	0.1
IL13	922	IL1RL2	ENSG00000115598.9	0.13	0.98	-0.07	0.97	-0.17	0.85
IL13	922	PASK	ENSG00000115687.13	0.13	0.91	0.22	0.63	-0.08	0.87
IL13	922	PSMC3IP	ENSG00000131470.14	0.13	0.96	0.36	0.38	-0.06	0.93
IL13	922	GNL2	ENSG00000134697.12	0.13	0.96	0.31	0.57	0.21	0.64
IL13	922	WDR12	ENSG00000138442.9	0.13	0.73	0.15	0.59	0.19	0.25
IL13	922	BRCA2	ENSG00000139618.14	0.13	0.98	0.64	0.39	0.25	0.79
IL13	922	OTUD6B	ENSG00000155100.10	0.13	0.95	0.28	0.58	0.35	0.23
IL13	922	TOPBP1	ENSG00000163781.12	0.13	0.97	0.24	0.83	0.19	0.79
IL13	922	ABCE1	ENSG00000164163.10	0.13	0.96	0.34	0.5	0.36	0.28
IL13	922	DDX21	ENSG00000165732.12	0.13	0.94	0.46	0.11	0.34	0.24
IL13	922	FEN1	ENSG00000168496.3	0.13	0.97	0.33	0.61	0.3	0.5
IL13	922	SPIN4	ENSG00000186767.6	0.13	0.96	-0.09	0.94	0.3	0.43
IL13	922	MAK16	ENSG00000198042.10	0.13	0.94	0.37	0.28	0.39	0.13
IL13	922	AL513165.1	ENSG00000234160.1	0.13	0.98	-0.07	0.98	0.27	0.79
IL13	922	NME1	ENSG00000239672.7	0.13	0.98	-0.01	1	0.17	0.84
IL13	922	APBA3	ENSG00000011132.11	0.12	0.79	0.25	0.2	0.33	0.015
IL13	922	NOP56	ENSG00000101361.16	0.12	0.66	0.04	0.94	0.16	0.24
IL13	922	POP1	ENSG00000104356.10	0.12	0.85	0.07	0.92	0.29	0.079
IL13	922	NOP2	ENSG00000111641.11	0.12	0.91	0.1	0.89	0.16	0.61
IL13	922	PAK1IP1	ENSG00000111845.4	0.12	0.93	0.16	0.8	0.22	0.46
IL13	922	HSPH1	ENSG00000120694.19	0.12	0.96	0.21	0.76	0.26	0.46
IL13	922	CSE1L	ENSG00000124207.16	0.12	0.97	0.27	0.71	0.23	0.63
IL13	922	AEN	ENSG00000181026.14	0.12	0.85	0.06	0.93	0.24	0.22
IL13	922	C3orf58	ENSG00000181744.8	0.12	0.89	0.44	0.019	0.6	0.000061
IL13	922	ARL9	ENSG00000196503.3	0.12	1	-0.03	0.99	0.12	0.95
IL13	922	MZT1	ENSG00000204899.5	0.12	0.9	0.13	0.84	0.16	0.62
IL13	922	ZNF587B	ENSG00000269343.6	0.12	0.94	0.06	0.95	0.23	0.45
IL13	922	USP13	ENSG00000058056.8	0.11	0.92	-0.16	0.75	0.07	0.87
IL13	922	GTPBP4	ENSG00000107937.18	0.11	0.92	0.26	0.38	0.25	0.26
IL13	922	NUDT15	ENSG00000136159.3	0.11	0.94	0.14	0.84	0.17	0.62
IL13	922	CENPU	ENSG00000151725.11	0.11	0.97	0.52	0.17	0.26	0.59
IL13	922	PDIA4	ENSG00000155660.10	0.11	0.6	0.1	0.64	0.33	0.000044
IL13	922	ATAD3B	ENSG00000160072.19	0.11	0.95	-0.01	0.99	0.12	0.79
IL13	922	ERAP1	ENSG00000164307.12	0.11	0.96	0.73	0.0012	0.72	0.00058

IL13	922	ERAP2	ENSG00000164308.16	0.11	0.97	0.71	0.01	0.57	0.035
IL13	922	PUS1	ENSG00000177192.13	0.11	0.95	0.1	0.9	0.16	0.64
IL13	922	HES7	ENSG00000179111.8	0.11	0.98	-0.3	0.83	0.32	0.64
IL13	922	FJX1	ENSG00000179431.6	0.11	0.91	0.11	0.87	0.36	0.039
IL13	922	AC073130.1	ENSG00000237870.6	0.11	2	0	2	0.32	2
IL13	922	AC024060.1	ENSG00000271870.1	0.11	0.99	-0.02	1	0.43	0.65
IL13	922	LAP3	ENSG00000002549.12	0.1	0.94	0.58	0.0026	0.85	0.000000096
IL13	922	PRPS2	ENSG00000101911.12	0.1	0.87	0.16	0.54	0.17	0.34
IL13	922	TJP2	ENSG00000119139.17	0.1	0.97	0.35	0.36	0.63	0.0038
IL13	922	SLC5A6	ENSG00000138074.14	0.1	0.93	0.4	0.04	0.43	0.0083
IL13	922	CNOT9	ENSG00000144580.13	0.1	0.8	0.24	0.099	0.38	0.00038
IL13	922	WDR43	ENSG00000163811.11	0.1	0.96	0.27	0.49	0.26	0.36
IL13	922	CYCS	ENSG00000172115.8	0.1	0.96	0.2	0.72	0.26	0.37
IL13	922	UCP3	ENSG00000175564.12	0.1	1	-0.02	1	0.05	0.98
IL13	922	WDHD1	ENSG00000198554.11	0.1	0.98	0.48	0.35	0.12	0.88
IL13	922	VN1R81P	ENSG00000268357.1	0.1	1	-0.19	0.94	0.03	0.99
IL13	922	POLD3	ENSG00000077514.8	0.09	0.94	0.09	0.89	0.19	0.41
IL13	922	ACOT7	ENSG00000097021.19	0.09	0.97	0.08	0.93	0.46	0.034
IL13	922	CENPI	ENSG00000102384.13	0.09	0.99	0.45	0.57	0.36	0.55
IL13	922	MGME1	ENSG00000125871.13	0.09	0.96	-0.05	0.96	0.05	0.91
IL13	922	FIGNL1	ENSG00000132436.11	0.09	0.99	0.1	0.95	0.23	0.73
IL13	922	DOCK10	ENSG00000135905.18	0.09	0.99	0.65	0.12	0.74	0.024
IL13	922	SLC31A2	ENSG00000136867.10	0.09	0.94	0.41	0.0088	0.49	0.00029
IL13	922	UTP4	ENSG00000141076.17	0.09	0.94	0.03	0.97	0.16	0.51
IL13	922	HSPD1	ENSG00000144381.16	0.09	0.97	0.13	0.88	0.1	0.85
IL13	922	TIFA	ENSG00000145365.10	0.09	0.93	0.3	0.082	0.31	0.033
IL13	922	EME1	ENSG00000154920.14	0.09	0.98	0.18	0.88	0.17	0.79
IL13	922	AGPAT5	ENSG00000155189.11	0.09	0.97	0.37	0.31	0.35	0.22
IL13	922	NUP205	ENSG00000155561.14	0.09	0.97	0.12	0.9	0.2	0.6
IL13	922	SGO2	ENSG00000163535.17	0.09	0.99	-0.14	0.93	0.14	0.88
IL13	922	SEC11C	ENSG00000166562.8	0.09	0.99	0.13	0.93	0.42	0.38
IL13	922	NETO2	ENSG00000171208.9	0.09	0.96	0.12	0.87	0.04	0.94
IL13	922	POLE	ENSG00000177084.16	0.09	0.97	0.02	0.99	0.08	0.88
IL13	922	GINS3	ENSG00000181938.13	0.09	0.98	0.17	0.88	-0.01	0.99
IL13	922	BAZ1A	ENSG00000198604.10	0.09	0.98	0.52	0.14	0.38	0.28
IL13	922	ANXA2P2	ENSG00000231991.4	0.09	0.91	0.07	0.91	0.42	0.0016
IL13	922	HSP90AA1	ENSG00000080824.18	0.08	0.99	0.23	0.85	0.18	0.82
IL13	922	NDC80	ENSG00000080986.12	0.08	0.99	0.49	0.33	0.23	0.7
IL13	922	B4GALNT1	ENSG00000135454.13	0.08	2	0.14	2	0.27	2
IL13	922	CCDC150	ENSG00000144395.17	0.08	1	0.29	0.87	0.23	0.83
IL13	922	HYOU1	ENSG00000149428.18	0.08	0.96	0.26	0.37	0.39	0.029
IL13	922	TMEM51	ENSG00000171729.13	0.08	0.96	0.6	0.000019	0.59	0.0000088
IL13	922	BEND3	ENSG00000178409.13	0.08	0.97	0.25	0.52	0.14	0.73
IL13	922	ZNF823	ENSG00000197933.12	0.08	0.99	0.15	0.93	0.09	0.93
IL13	922	GGCT	ENSG00000006625.17	0.07	0.99	0.12	0.93	0.01	1
IL13	922	TDP1	ENSG00000042088.13	0.07	0.95	0.11	0.75	0.12	0.54
IL13	922	PRDM1	ENSG00000057657.15	0.07	0.99	0.07	0.97	0.11	0.9
IL13	922	REEP1	ENSG00000068615.17	0.07	2	-0.32	2	-0.45	2
IL13	922	ATP2B1	ENSG00000070961.15	0.07	0.99	0.45	0.26	0.16	0.77
IL13	922	ORC1	ENSG00000085840.12	0.07	1	0.52	0.39	-0.29	0.65
IL13	922	SRPK1	ENSG00000096063.15	0.07	0.99	0.5	0.093	0.36	0.21
IL13	922	POLA1	ENSG00000101868.10	0.07	0.99	0.21	0.82	0.18	0.73
IL13	922	PNO1	ENSG00000115946.7	0.07	0.91	0.15	0.48	0.16	0.25
IL13	922	ACYP1	ENSG00000119640.8	0.07	0.98	0	1	-0.02	0.98
IL13	922	CISD1	ENSG00000122873.11	0.07	0.99	0.1	0.94	0.24	0.7
IL13	922	ITPR2	ENSG00000123104.11	0.07	1	0.24	0.9	0.28	0.76
IL13	922	HAUS6	ENSG00000147874.10	0.07	0.99	0.42	0.46	0.2	0.74
IL13	922	RPL22L1	ENSG00000163584.17	0.07	0.98	-0.01	1	0.26	0.53
IL13	922	STXBP6	ENSG00000168952.15	0.07	1	-0.72	0.62	-0.5	0.68
IL13	922	RCC2	ENSG00000179051.13	0.07	0.86	0.28	0.0022	0.37	0.0000016
IL13	922	ACTL10	ENSG00000182584.4	0.07	1	-0.27	0.82	-0.18	0.83
IL13	922	ORAI1	ENSG00000276045.2	0.07	0.96	0.41	0.018	0.54	0.00013
IL13	922	ITGA6	ENSG00000091409.14	0.06	0.99	0.22	0.74	0.11	0.84
IL13	922	KIF14	ENSG00000118193.11	0.06	1	0.65	0.23	0.25	0.74
IL13	922	SPATA5	ENSG00000145375.7	0.06	0.98	0.1	0.91	0.05	0.94

IL13	922	ARHGAP45	ENSG00000180448.10	0.06	0.99	-0.08	0.95	0.08	0.91
IL13	922	ZFP91	ENSG00000186660.14	0.06	0.97	0.22	0.36	0.24	0.16
IL13	922	ATAD3A	ENSG00000197785.13	0.06	0.99	0.03	0.98	0.19	0.69
IL13	922	NUP62	ENSG00000213024.11	0.06	0.96	0.21	0.2	0.41	0.000062
IL13	922	NOP56P1	ENSG00000235559.1	0.06	1	0.16	2	0.22	0.9
IL13	922	AC004801.2	ENSG00000240399.1	0.06	2	-0.15	2	0.11	2
IL13	922	TNIP3	ENSG00000050730.15	0.05	1	1.6	2.9E-10	1.73	7.1E-13
IL13	922	NUP50	ENSG00000093000.18	0.05	0.99	0.15	0.85	0.09	0.87
IL13	922	WDR77	ENSG00000116455.13	0.05	0.98	-0.05	0.94	-0.03	0.94
IL13	922	SRM	ENSG00000116649.9	0.05	0.99	-0.01	0.99	0.1	0.86
IL13	922	ZMIZ2	ENSG00000122515.14	0.05	0.99	0.62	0.0087	0.66	0.0014
IL13	922	HAT1	ENSG00000128708.12	0.05	0.98	0.05	0.95	0.17	0.52
IL13	922	EXOSC2	ENSG00000130713.15	0.05	0.96	-0.1	0.7	0.01	0.97
IL13	922	FKBP11	ENSG00000134285.10	0.05	0.99	0.21	0.72	0.25	0.41
IL13	922	STAMBPL1	ENSG00000138134.11	0.05	0.99	-0.27	0.41	-0.03	0.96
IL13	922	PTTG1	ENSG00000164611.12	0.05	1	-0.11	0.96	-0.06	0.96
IL13	922	CENPN	ENSG00000166451.13	0.05	0.99	0.14	0.83	0.05	0.92
IL13	922	LPAR3	ENSG00000171517.5	0.05	1	-0.95	2	-1.01	0.28
IL13	922	SFXN4	ENSG00000183605.16	0.05	1	0.03	0.98	0.07	0.91
IL13	922	IPO4	ENSG00000196497.16	0.05	1	-0.07	0.97	-0.16	0.84
IL13	922	RNU6-26P	ENSG00000206712.1	0.05	1	1.03	0.15	0.87	0.18
IL13	922	DNAJC9	ENSG00000213551.4	0.05	0.98	-0.1	0.84	0.01	0.99
IL13	922	POLA2	ENSG00000014138.8	0.04	0.99	-0.09	0.89	0.12	0.69
IL13	922	NAMPT	ENSG00000105835.11	0.04	1	0.92	0.0003	0.8	0.0013
IL13	922	PPIF	ENSG00000108179.13	0.04	0.99	0.27	0.41	0.32	0.14
IL13	922	UAP1	ENSG00000117143.13	0.04	0.99	0.31	0.31	0.27	0.28
IL13	922	DDX39A	ENSG00000123136.14	0.04	1	0.11	0.9	0.1	0.83
IL13	922	RPP40	ENSG00000124787.13	0.04	1	0.04	0.98	0.28	0.5
IL13	922	PRMT1	ENSG00000126457.21	0.04	0.99	0.14	0.81	0.22	0.36
IL13	922	EIF5A	ENSG00000132507.17	0.04	1	0.1	0.93	0.26	0.47
IL13	922	PCNA	ENSG00000132646.10	0.04	1	-0.13	0.89	-0.03	0.97
IL13	922	IRAK2	ENSG00000134070.4	0.04	0.99	0.48	0.00016	0.25	0.1
IL13	922	PPRC1	ENSG00000148840.10	0.04	1	0.12	0.91	0.08	0.9
IL13	922	IL15	ENSG00000164136.16	0.04	1	0.71	0.00019	0.64	0.00059
IL13	922	SNRPD1	ENSG00000167088.10	0.04	1	0.08	0.96	0.25	0.63
IL13	922	RRS1	ENSG00000179041.3	0.04	0.99	0.29	0.056	0.24	0.093
IL13	922	CA13	ENSG00000185015.7	0.04	1	0	1	0.12	0.82
IL13	922	COLCA2	ENSG00000214290.8	0.04	2	0.02	2	0.05	2
IL13	922	FANCG	ENSG00000221829.9	0.04	1	-0.11	0.9	-0.07	0.9
IL13	922	RANP1	ENSG00000236603.2	0.04	1	0.07	0.97	0.27	0.63
IL13	922	ICAM1	ENSG00000090339.8	0.03	1	0.62	0.0015	0.57	0.0019
IL13	922	E2F1	ENSG00000101412.12	0.03	1	0.14	0.92	0.17	0.82
IL13	922	SH2B3	ENSG00000111252.10	0.03	1	0.22	0.83	0.21	0.72
IL13	922	XPO5	ENSG00000124571.17	0.03	1	0.15	0.79	0.15	0.64
IL13	922	PAICS	ENSG00000128050.8	0.03	0.98	0.12	0.64	0.12	0.49
IL13	922	WDR89	ENSG00000140006.11	0.03	1	0.39	0.29	0.41	0.14
IL13	922	POLE3	ENSG00000148229.12	0.03	0.99	-0.01	0.99	0.1	0.77
IL13	922	INO80C	ENSG00000153391.15	0.03	1	0.15	0.87	0.39	0.18
IL13	922	ZDHHC14	ENSG00000175048.16	0.03	1	0.05	0.94	0.3	0.07
IL13	922	ZNF267	ENSG00000185947.14	0.03	1	1.05	1.4E-08	0.87	0.0000023
IL13	922	NCR3LG1	ENSG00000188211.8	0.03	1	0.19	0.85	0.12	0.86
IL13	922	SLC12A8	ENSG00000221955.10	0.03	1	-0.5	0.0042	0.01	0.99
IL13	922	YBX1P2	ENSG00000231167.3	0.03	1	0.34	0.8	0.1	0.94
IL13	922	AL357060.2	ENSG00000237499.6	0.03	1	0.15	0.93	0.29	0.7
IL13	922	RBM14	ENSG00000239306.4	0.03	0.99	-0.03	0.97	0	1
IL13	922	SBF2-AS1	ENSG00000246273.6	0.03	1	0.07	0.94	-0.05	0.95
IL13	922	AC025419.1	ENSG00000250748.6	0.03	1	0.86	0.11	0.3	0.74
IL13	922	AC005180.1	ENSG00000267405.1	0.03	2	0.31	2	-0.09	2
IL13	922	HMMR	ENSG00000072571.19	0.02	1	0.42	0.61	0.21	0.8
IL13	922	COL5A3	ENSG00000080573.6	0.02	1	0.28	0.82	0.55	0.23
IL13	922	HSPE1	ENSG00000115541.10	0.02	1	-0.09	0.95	-0.01	0.99
IL13	922	TUBA1B	ENSG00000123416.15	0.02	1	0.02	0.99	0.28	0.28
IL13	922	IFNAR2	ENSG00000159110.19	0.02	1	0.25	0.24	0.32	0.031
IL13	922	TLCD1	ENSG00000160606.10	0.02	1	0.11	0.93	0.29	0.48
IL13	922	DTYMK	ENSG00000168393.12	0.02	1	-0.06	0.97	0.06	0.95

IL13	922	PA2G4	ENSG00000170515.13	0.02	1	0.02	0.97	0.05	0.87
IL13	922	GMPPB	ENSG00000173540.12	0.02	1	-0.11	0.87	0.12	0.71
IL13	922	ACTA2-AS1	ENSG00000180139.11	0.02	1	-0.71	2	-0.65	0.53
IL13	922	PPTC7	ENSG00000196850.5	0.02	1	0.44	0.057	0.3	0.22
IL13	922	ZNF257	ENSG00000197134.11	0.02	2	0.12	2	0.04	2
IL13	922	HNRNPAB	ENSG00000197451.11	0.02	0.99	0.11	0.58	0.12	0.36
IL13	922	ZNF551	ENSG00000204519.10	0.02	1	-0.07	0.97	0.02	0.99
IL13	922	RFC2	ENSG00000049541.10	0.01	1	0.01	0.99	0.05	0.93
IL13	922	RAD51	ENSG00000051180.16	0.01	1	0.14	0.88	0.08	0.9
IL13	922	MRTO4	ENSG00000053372.4	0.01	1	0.02	0.99	0.11	0.83
IL13	922	TRAF4	ENSG00000076604.14	0.01	1	0.03	0.98	0.07	0.9
IL13	922	ENO3	ENSG00000108515.17	0.01	1	-0.07	0.95	0.06	0.92
IL13	922	POLR3G	ENSG00000113356.11	0.01	1	0.32	0.54	0	1
IL13	922	SDF2L1	ENSG00000128228.4	0.01	1	0.16	0.92	0.32	0.58
IL13	922	DUS3L	ENSG00000141994.15	0.01	1	0.07	0.94	0.03	0.96
IL13	922	CCT5	ENSG00000150753.11	0.01	1	0.16	0.75	0.14	0.65
IL13	922	BUB3	ENSG00000154473.17	0.01	1	0.25	0.29	0.09	0.78
IL13	922	RFC4	ENSG00000163918.10	0.01	1	-0.04	0.98	-0.1	0.89
IL13	922	LRR1	ENSG00000165501.16	0.01	1	-0.02	0.99	-0.04	0.96
IL13	922	ERN1	ENSG00000178607.15	0.01	1	0.26	0.42	0.22	0.38
IL13	922	CDCA2	ENSG00000184661.13	0.01	1	0.64	0.046	0.03	0.97
IL13	922	PRIM1	ENSG00000198056.13	0.01	1	0.13	0.93	0.2	0.78
IL13	922	PIGW	ENSG00000277161.1	0.01	1	0.16	0.79	0.29	0.24
IL13	922	METTL1	ENSG00000037897.16	0	1	0.23	0.72	0.33	0.3
IL13	922	MSH2	ENSG00000095002.13	0	1	0.13	0.92	0.03	0.97
IL13	922	NFKBIB	ENSG00000104825.16	0	1	0.12	0.93	0.26	0.62
IL13	922	PLXNA1	ENSG00000114554.11	0	1	0.77	0.0026	0.71	0.0027
IL13	922	CENPL	ENSG00000120334.15	0	1	-0.03	0.98	0.2	0.59
IL13	922	HIVEP3	ENSG00000127124.14	0	1	0.47	0.27	0.56	0.063
IL13	922	EGLN3	ENSG00000129521.13	0	1	0.69	0.29	1.25	0.0011
IL13	922	MYBBP1A	ENSG00000132382.14	0	1	0.14	0.87	0.07	0.9
IL13	922	CLGN	ENSG00000153132.12	0	1	0.21	0.83	0.27	0.57
IL13	922	PKN3	ENSG00000160447.6	0	1	-0.25	0.78	-0.02	0.99
IL13	922	MARCH3	ENSG00000173926.5	0	1	-0.27	0.011	-0.27	0.005
IL13	922	TLR10	ENSG00000174123.10	0	2	0.39	2	0.42	2
IL13	922	AC022613.1	ENSG00000256802.2	0	1	0.63	0.03	0.93	0.000033
IL13	922	VPS9D1-AS1	ENSG00000261373.1	0	1	-0.08	0.92	-0.34	0.11
IL13	922	SNHG25	ENSG00000266402.3	0	1	0.41	0.73	-0.02	0.99
IL13	922	MGLL	ENSG00000074416.13	-0.01	1	0.31	0.33	0.15	0.68
IL13	922	KIF22	ENSG00000079616.12	-0.01	1	-0.05	0.97	-0.14	0.82
IL13	922	SEH1L	ENSG00000085415.15	-0.01	1	0.18	0.47	0.17	0.34
IL13	922	KIAA0391	ENSG00000100890.15	-0.01	1	0.12	0.88	0.18	0.56
IL13	922	DOT1L	ENSG00000104885.17	-0.01	1	0.17	0.79	0.18	0.61
IL13	922	RRP9	ENSG00000114767.6	-0.01	1	-0.03	0.99	-0.14	0.83
IL13	922	PHF19	ENSG00000119403.13	-0.01	1	0.1	0.84	-0.01	0.98
IL13	922	DSCC1	ENSG00000136982.5	-0.01	1	0.37	0.57	0.17	0.8
IL13	922	SLC37A4	ENSG00000137700.17	-0.01	1	-0.15	0.78	-0.08	0.84
IL13	922	CEP55	ENSG00000138180.15	-0.01	1	0.47	0.36	0.31	0.54
IL13	922	MMS22L	ENSG00000146263.11	-0.01	1	-0.03	0.98	-0.31	0.41
IL13	922	POLR3K	ENSG00000161980.5	-0.01	1	-0.08	0.97	0.11	0.91
IL13	922	MTMR10	ENSG00000166912.16	-0.01	1	0.35	0.057	0.47	0.0011
IL13	922	IFRD2	ENSG00000214706.10	-0.01	1	0.06	0.96	0.08	0.9
IL13	922	AL161891.1	ENSG00000276672.1	-0.01	1	0.23	2	0.26	0.87
IL13	922	NUP188	ENSG00000095319.14	-0.02	1	0.05	0.97	0.14	0.82
IL13	922	DHODH	ENSG00000102967.11	-0.02	1	0.09	0.94	0.11	0.88
IL13	922	UBE2S	ENSG00000108106.13	-0.02	1	-0.05	0.98	0	1
IL13	922	E2F3	ENSG00000112242.14	-0.02	1	0.15	0.47	0.12	0.48
IL13	922	CEP72	ENSG00000112877.7	-0.02	1	-0.06	0.96	-0.03	0.96
IL13	922	AGMAT	ENSG00000116771.5	-0.02	1	0.16	0.89	-0.11	0.88
IL13	922	DCLRE1B	ENSG00000118655.4	-0.02	1	0.17	0.68	-0.06	0.89
IL13	922	NUDCD1	ENSG00000120526.10	-0.02	1	0.31	0.3	0.33	0.15
IL13	922	NUP153	ENSG00000124789.11	-0.02	1	0.2	0.79	0.14	0.78
IL13	922	THEM6	ENSG00000130193.7	-0.02	1	-0.38	0.31	-0.18	0.7
IL13	922	CCDC15	ENSG00000149548.14	-0.02	1	0.77	0.12	0.41	0.49
IL13	922	AFAP1L1	ENSG00000157510.13	-0.02	1	-0.09	0.92	0.02	0.97

IL13	922	ARHGAP42	ENSG00000165895.17	-0.02	1	0.64	0.15	0.56	0.14
IL13	922	RRM1	ENSG00000167325.14	-0.02	1	0.13	0.87	0.08	0.89
IL13	922	CHAF1A	ENSG00000167670.15	-0.02	1	0.14	0.79	0.03	0.96
IL13	922	STIP1	ENSG00000168439.16	-0.02	1	0.01	0.99	0.1	0.63
IL13	922	THOP1	ENSG00000172009.14	-0.02	1	-0.09	0.93	0.11	0.82
IL13	922	ZNF124	ENSG00000196418.12	-0.02	1	-0.1	0.94	-0.02	0.99
IL13	922	UHRF1	ENSG00000276043.4	-0.02	1	0.43	0.034	0.25	0.3
IL13	922	TMEM38B	ENSG00000095209.11	-0.03	1	0.18	0.74	0.06	0.91
IL13	922	TNFRSF10A	ENSG00000104689.9	-0.03	1	0.32	0.44	0.22	0.54
IL13	922	MCM3	ENSG00000112118.18	-0.03	0.99	0.08	0.87	0.07	0.82
IL13	922	KNSTRN	ENSG00000128944.13	-0.03	1	0.11	0.84	-0.08	0.82
IL13	922	TOMM40	ENSG00000130204.12	-0.03	1	0.01	0.99	0.08	0.86
IL13	922	PPAN	ENSG00000130810.19	-0.03	1	-0.09	0.95	-0.12	0.88
IL13	922	ITGAV	ENSG00000138448.11	-0.03	1	0.68	0.033	0.56	0.07
IL13	922	CLSTN3	ENSG00000139182.13	-0.03	1	0.45	0.1	0.97	0.000000093
IL13	922	C2CD2	ENSG00000157617.16	-0.03	1	0.58	0.012	0.48	0.029
IL13	922	LMNB2	ENSG00000176619.12	-0.03	1	0.16	0.74	0.14	0.65
IL13	922	PFAS	ENSG00000178921.13	-0.03	1	0.11	0.93	-0.01	1
IL13	922	PGP	ENSG00000184207.8	-0.03	1	0.13	0.92	0.09	0.91
IL13	922	PIM3	ENSG00000198355.4	-0.03	1	0.32	0.37	0.36	0.14
IL13	922	TAPBP	ENSG00000231925.11	-0.03	1	0.47	0.031	0.39	0.053
IL13	922	AC091182.2	ENSG00000253746.1	-0.03	1	0.34	0.61	0.44	0.25
IL13	922	AC093788.1	ENSG00000273449.1	-0.03	1	-0.37	0.87	-0.35	0.79
IL13	922	ZNF670	ENSG00000277462.1	-0.03	1	0.48	0.23	0.16	0.8
IL13	922	RBL1	ENSG00000080839.11	-0.04	1	0.02	0.99	-0.09	0.93
IL13	922	MTHFD1	ENSG00000100714.15	-0.04	0.99	0.11	0.85	0.1	0.74
IL13	922	USP31	ENSG00000103404.14	-0.04	1	0.63	0.0058	0.3	0.3
IL13	922	GRWD1	ENSG00000105447.12	-0.04	0.99	-0.03	0.97	0.04	0.91
IL13	922	TUBA1C	ENSG00000167553.15	-0.04	1	0.16	0.88	0.22	0.66
IL13	922	SPSB1	ENSG00000171621.13	-0.04	0.99	0.14	0.78	-0.03	0.94
IL13	922	KLF17	ENSG00000171872.4	-0.04	2	-0.64	2	0.53	2
IL13	922	FGD6	ENSG00000180263.13	-0.04	1	0.8	0.037	0.72	0.039
IL13	922	PSMC1P1	ENSG00000241506.1	-0.04	1	0.21	0.87	0.21	0.76
IL13	922	GPR75	ENSG00000119737.5	-0.05	1	-0.24	0.9	-0.1	0.94
IL13	922	DAB2IP	ENSG00000136848.16	-0.05	0.99	0.38	0.057	0.63	0.000016
IL13	922	CCNA2	ENSG00000145386.9	-0.05	0.99	0.32	0.37	-0.1	0.85
IL13	922	RFWD3	ENSG00000168411.13	-0.05	0.99	0	1	0.05	0.93
IL13	922	AC003092.1	ENSG00000236453.5	-0.05	1	0.7	0.037	0.6	0.059
IL13	922	AC011503.2	ENSG00000269397.1	-0.05	2	0.13	2	0.16	2
IL13	922	MYO19	ENSG00000278259.4	-0.05	0.98	0	1	-0.08	0.82
IL13	922	GINS1	ENSG00000101003.9	-0.06	0.99	0.43	0.2	0.15	0.79
IL13	922	BCL7A	ENSG00000110987.8	-0.06	0.99	0.34	0.32	0.29	0.3
IL13	922	TOP2A	ENSG00000131747.14	-0.06	0.99	0.67	0.025	0.31	0.42
IL13	922	INCENP	ENSG00000149503.12	-0.06	0.99	0.12	0.89	0.03	0.97
IL13	922	CHEK1	ENSG00000149554.12	-0.06	0.99	-0.06	0.95	-0.06	0.92
IL13	922	POC5	ENSG00000152359.14	-0.06	0.99	0.05	0.97	0.1	0.87
IL13	922	LGI2	ENSG00000153012.11	-0.06	0.98	1.86	2	1.39	0.056
IL13	922	TONSL	ENSG00000160949.16	-0.06	0.99	-0.02	0.99	-0.03	0.96
IL13	922	MCM7	ENSG00000166508.17	-0.06	0.96	0.15	0.57	0.04	0.9
IL13	922	CTPS1	ENSG00000171793.13	-0.06	0.96	0.19	0.3	0	1
IL13	922	VARS	ENSG00000204394.12	-0.06	0.98	-0.04	0.97	-0.04	0.92
IL13	922	AL365361.1	ENSG00000259834.1	-0.06	1	-0.03	0.99	0.29	0.83
IL13	922	AC092718.4	ENSG00000261061.1	-0.06	0.99	0.11	0.94	0.03	0.98
IL13	922	SPIB	ENSG00000269404.6	-0.06	0.98	0.68	2	0.66	0.42
IL13	922	MATR3	ENSG00000015479.18	-0.06	1	0.06	0.98	0.11	0.9
IL13	922	SLC38A5	ENSG00000017483.14	-0.07	0.98	-0.08	0.92	-0.07	0.89
IL13	922	TRMT6	ENSG00000089195.14	-0.07	0.98	0.19	0.76	0.13	0.79
IL13	922	SPX	ENSG00000134548.9	-0.07	1	-0.05	0.99	-0.08	0.96
IL13	922	STX11	ENSG00000135604.9	-0.07	0.99	-0.05	0.97	0.3	0.38
IL13	922	UCN2	ENSG00000145040.3	-0.07	1	0.28	0.88	0.16	0.9
IL13	922	NCAPG2	ENSG00000146918.19	-0.07	0.99	0.08	0.94	-0.08	0.9
IL13	922	RACGAP1	ENSG00000161800.12	-0.07	0.96	-0.05	0.92	-0.06	0.84
IL13	922	POC1A	ENSG00000164087.7	-0.07	0.98	-0.03	0.98	-0.01	0.99
IL13	922	DDIAS	ENSG00000165490.12	-0.07	1	0.17	0.92	-0.01	0.99
IL13	922	QSOX2	ENSG00000165661.16	-0.07	0.97	0.23	0.51	0.11	0.77

IL13	922	HEG1	ENSG00000173706.12	-0.07	0.99	-0.37	0.31	-0.09	0.88
IL13	922	SLC25A10	ENSG00000183048.11	-0.07	1	-0.27	0.83	-0.12	0.9
IL13	922	ALPK2	ENSG00000198796.6	-0.07	0.99	0.14	0.91	0.19	0.74
IL13	922	JPT2	ENSG00000206053.12	-0.07	0.91	0.21	0.11	0.16	0.23
IL13	922	DHFR	ENSG00000228716.6	-0.07	0.96	0.13	0.79	0.07	0.87
IL13	922	SLC25A13	ENSG00000004864.13	-0.08	0.96	0.17	0.65	-0.08	0.84
IL13	922	DDX11	ENSG00000013573.16	-0.08	0.96	0.14	0.74	0	1
IL13	922	GALNT18	ENSG00000110328.5	-0.08	0.98	0.34	0.46	0.89	0.00002
IL13	922	TNFRSF10B	ENSG00000120889.12	-0.08	0.95	0.37	0.043	0.19	0.39
IL13	922	GNAZ	ENSG00000128266.8	-0.08	1	-0.31	0.85	-0.92	0.063
IL13	922	PDLIM4	ENSG00000131435.12	-0.08	0.98	0.52	0.025	0.54	0.0064
IL13	922	CLUH	ENSG00000132361.16	-0.08	0.96	-0.01	0.99	-0.09	0.76
IL13	922	GGH	ENSG00000137563.11	-0.08	0.98	0.19	0.82	0.14	0.8
IL13	922	KNL1	ENSG00000137812.19	-0.08	0.99	0.43	0.59	0.06	0.96
IL13	922	SEMA7A	ENSG00000138623.9	-0.08	0.98	0.97	0.000012	0.46	0.092
IL13	922	SLC27A2	ENSG00000140284.10	-0.08	2	-0.23	2	0.08	2
IL13	922	C9orf72	ENSG00000147894.14	-0.08	0.98	0.63	0.014	0.11	0.86
IL13	922	WDR4	ENSG00000160193.11	-0.08	0.96	-0.03	0.97	0.15	0.66
IL13	922	CD320	ENSG00000167775.10	-0.08	0.99	-0.15	0.92	-0.09	0.92
IL13	922	CDK5R1	ENSG00000176749.8	-0.08	0.98	-0.01	0.99	-0.11	0.85
IL13	922	PCGF5	ENSG00000180628.14	-0.08	0.98	0.25	0.66	0.05	0.93
IL13	922	FUT4	ENSG00000196371.3	-0.08	0.95	0.43	0.0029	0.56	0.0000067
IL13	922	BOP1	ENSG00000261236.7	-0.08	0.98	-0.07	0.95	-0.07	0.92
IL13	922	FKBP4	ENSG00000004478.7	-0.09	0.91	-0.1	0.85	-0.15	0.5
IL13	922	NDC1	ENSG00000058804.11	-0.09	0.97	0.09	0.93	-0.05	0.93
IL13	922	NUP88	ENSG00000108559.11	-0.09	0.9	0.02	0.98	-0.01	0.98
IL13	922	NSD2	ENSG00000109685.17	-0.09	0.95	0.23	0.54	0.07	0.87
IL13	922	ULBP2	ENSG00000131015.4	-0.09	0.96	0.19	0.69	0.06	0.9
IL13	922	TMEM132B	ENSG00000139364.10	-0.09	0.98	0.95	0.00083	0.4	0.29
IL13	922	DUSP10	ENSG00000143507.17	-0.09	0.87	0.45	0.000063	0.07	0.78
IL13	922	TNFRSF4	ENSG00000186827.10	-0.09	1	0.65	0.64	0.11	0.95
IL13	922	H2AFX	ENSG00000188486.3	-0.09	0.97	0	1	0.02	0.98
IL13	922	MARS2	ENSG00000247626.4	-0.09	0.97	0.22	0.66	0.29	0.26
IL13	922	AC093535.2	ENSG00000279118.1	-0.09	0.99	0.32	0.72	0.27	0.66
IL13	922	POLQ	ENSG00000051341.13	-0.1	0.99	0.8	0.14	0.27	0.76
IL13	922	COBLL1	ENSG00000082438.15	-0.1	0.98	0.1	0.94	-0.11	0.86
IL13	922	HSPA8	ENSG00000109971.13	-0.1	0.65	-0.09	0.69	-0.13	0.26
IL13	922	BTN2A2	ENSG00000124508.16	-0.1	0.9	0.62	1.7E-07	0.53	0.0000062
IL13	922	DLGAP5	ENSG00000126787.12	-0.1	0.98	0.7	0.044	0.27	0.6
IL13	922	KIF23	ENSG00000137807.13	-0.1	0.98	0.4	0.43	0.23	0.67
IL13	922	NOC4L	ENSG00000184967.6	-0.1	0.96	-0.1	0.91	-0.02	0.98
IL13	922	CENPW	ENSG00000203760.8	-0.1	0.99	0.01	1	-0.2	0.84
IL13	922	MTFP1	ENSG00000242114.5	-0.1	0.98	0.02	0.99	0.37	0.41
IL13	922	AC023906.5	ENSG00000259712.1	-0.1	0.99	0.3	0.83	-0.01	1
IL13	922	MATR3	ENSG00000280987.4	-0.1	0.98	0.26	0.79	0.11	0.89
IL13	922	NCAPG	ENSG00000109805.9	-0.11	0.98	0.46	0.47	0.15	0.86
IL13	922	NUP155	ENSG00000113569.15	-0.11	0.96	0.09	0.93	0	1
IL13	922	UTP20	ENSG00000120800.4	-0.11	0.98	0.23	0.81	-0.11	0.88
IL13	922	KIF18A	ENSG00000121621.6	-0.11	0.98	0.36	0.63	-0.06	0.95
IL13	922	NXT1	ENSG00000132661.3	-0.11	0.97	0.09	0.94	0.2	0.71
IL13	922	TCF19	ENSG00000137310.11	-0.11	0.95	0.13	0.88	-0.31	0.28
IL13	922	KIF11	ENSG00000138160.5	-0.11	0.98	0.52	0.26	0.17	0.81
IL13	922	TEX30	ENSG00000151287.16	-0.11	0.98	0.11	0.93	0.26	0.58
IL13	922	SGK494	ENSG00000167524.14	-0.11	0.98	0.23	0.82	0.06	0.95
IL13	922	SAPCD2	ENSG00000186193.8	-0.11	0.98	0.17	0.9	-0.04	0.96
IL13	922	ZNF850	ENSG00000267041.5	-0.11	0.98	-0.04	0.98	-0.44	0.3
IL13	922	SMC1A	ENSG00000072501.17	-0.12	0.98	0.17	0.89	0.06	0.95
IL13	922	TTLL12	ENSG00000100304.12	-0.12	0.83	-0.03	0.97	-0.05	0.91
IL13	922	OIP5	ENSG00000104147.8	-0.12	0.98	0.15	0.94	-0.09	0.94
IL13	922	TIMM10	ENSG00000134809.8	-0.12	0.98	-0.05	0.98	-0.08	0.95
IL13	922	NUSAP1	ENSG00000137804.12	-0.12	0.98	0.24	0.85	0.02	0.99
IL13	922	DHX37	ENSG00000150990.7	-0.12	0.8	0.01	0.99	0.05	0.9
IL13	922	ABHD3	ENSG00000158201.9	-0.12	0.98	0.29	0.79	0.13	0.89
IL13	922	CLPB	ENSG00000162129.12	-0.12	0.94	-0.12	0.9	-0.07	0.9
IL13	922	NEMP1	ENSG00000166881.9	-0.12	0.96	0.31	0.54	0.07	0.92

IL13	922	CDK1	ENSG00000170312.15	-0.12	0.96	0.53	0.079	0.03	0.98
IL13	922	CHD7	ENSG00000171316.11	-0.12	0.95	0.1	0.92	-0.03	0.97
IL13	922	DPY19L1	ENSG00000173852.14	-0.12	0.91	0.53	0.0036	0.23	0.37
IL13	922	RN7SL749P	ENSG00000242853.3	-0.12	2	0	2	0.02	2
IL13	922	AC124798.1	ENSG00000260196.1	-0.12	0.97	0.3	0.73	0.09	0.92
IL13	922	RRP12	ENSG00000052749.13	-0.13	0.79	0.06	0.92	-0.13	0.59
IL13	922	P3H2	ENSG00000090530.9	-0.13	0.78	0.09	0.86	0.21	0.2
IL13	922	GALE	ENSG00000117308.14	-0.13	0.85	-0.13	0.81	-0.13	0.67
IL13	922	NUP85	ENSG00000125450.10	-0.13	0.85	0	1	-0.1	0.77
IL13	922	BUB1B	ENSG00000156970.12	-0.13	0.98	0.53	0.53	0.14	0.9
IL13	922	CKS1B	ENSG00000173207.12	-0.13	0.98	-0.23	0.87	-0.25	0.72
IL13	922	NPB	ENSG00000183979.7	-0.13	0.99	0.03	0.99	0.03	0.99
IL13	922	DHFRP1	ENSG00000188985.6	-0.13	0.98	0.26	0.88	-0.15	0.89
IL13	922	ARHGAP11A	ENSG00000198826.10	-0.13	0.94	0.37	0.33	-0.08	0.9
IL13	922	CYP26B1	ENSG00000003137.8	-0.14	0.95	0.02	0.99	-0.03	0.97
IL13	922	SYNGR2	ENSG00000108639.7	-0.14	0.94	0.31	0.5	0.33	0.3
IL13	922	SLC43A3	ENSG00000134802.17	-0.14	0.87	0.44	0.032	0.31	0.15
IL13	922	CHAF1B	ENSG00000159259.7	-0.14	0.89	-0.07	0.94	-0.04	0.94
IL13	922	DBF4B	ENSG00000161692.17	-0.14	0.94	-0.08	0.94	-0.12	0.84
IL13	922	TMEM133	ENSG00000170647.3	-0.14	0.97	0.41	0.61	0.38	0.48
IL13	922	SP6	ENSG00000189120.4	-0.14	0.95	1.9	3.2E-19	1.76	3.2E-17
IL13	922	HMBS	ENSG00000256269.7	-0.14	0.95	-0.11	0.93	-0.07	0.93
IL13	922	RELT	ENSG00000054967.12	-0.15	0.81	0.27	0.31	0.37	0.029
IL13	922	SUV39H1	ENSG00000101945.16	-0.15	0.77	-0.1	0.88	-0.1	0.76
IL13	922	HS2ST1	ENSG00000153936.16	-0.15	0.94	0.12	0.92	-0.05	0.94
IL13	922	CENPX	ENSG00000169689.14	-0.15	0.96	-0.03	0.99	-0.11	0.89
IL13	922	SLC19A1	ENSG00000173638.18	-0.15	0.82	0.02	0.98	-0.24	0.29
IL13	922	GPAT2	ENSG00000186281.12	-0.15	0.99	0.27	0.91	-0.01	1
IL13	922	SLC6A7	ENSG00000011083.8	-0.16	2	-0.07	2	0.18	2
IL13	922	RNASEH2A	ENSG00000104889.5	-0.16	0.96	-0.1	0.95	-0.22	0.74
IL13	922	DEPDC7	ENSG00000121690.10	-0.16	0.91	0.35	0.41	0.06	0.93
IL13	922	ADGRE5	ENSG00000123146.19	-0.16	0.42	0.25	0.058	0.11	0.55
IL13	922	DCTPP1	ENSG00000179958.8	-0.16	0.91	-0.17	0.84	-0.24	0.55
IL13	922	XRCC2	ENSG00000196584.2	-0.16	0.96	0.42	0.54	0.1	0.92
IL13	922	EIF5AL1	ENSG00000253626.3	-0.16	0.96	0.1	0.95	0.34	0.48
IL13	922	EXOSC5	ENSG00000077348.8	-0.17	0.9	-0.1	0.93	-0.17	0.74
IL13	922	EZR	ENSG00000092820.17	-0.17	0.39	-0.32	0.0097	-0.29	0.012
IL13	922	SLC29A1	ENSG00000112759.16	-0.17	0.58	-0.03	0.97	-0.24	0.15
IL13	922	STIL	ENSG00000123473.15	-0.17	0.91	0.08	0.94	0.03	0.98
IL13	922	CLN6	ENSG00000128973.12	-0.17	0.72	-0.04	0.96	-0.22	0.3
IL13	922	GINS2	ENSG00000131153.8	-0.17	0.94	0.4	0.48	0.03	0.98
IL13	922	ADPRH	ENSG00000144843.11	-0.17	0.91	0.45	0.19	0.27	0.5
IL13	922	ITGA2	ENSG00000164171.10	-0.17	0.96	0.76	0.067	0.15	0.86
IL13	922	ZNF724	ENSG00000196081.9	-0.17	0.98	0	1	-0.18	0.89
IL13	922	AC073585.1	ENSG00000255624.2	-0.17	0.98	0.09	2	0.08	0.97
IL13	922	MIR4292	ENSG00000265806.1	-0.17	0.98	-0.29	0.9	-0.37	0.74
IL13	922	PRR11	ENSG00000068489.12	-0.18	0.75	0.14	0.81	-0.15	0.66
IL13	922	XYLB	ENSG00000093217.9	-0.18	0.81	0.05	0.96	-0.22	0.5
IL13	922	SOD2	ENSG00000112096.16	-0.18	0.77	1.13	4.7E-12	0.75	0.0000091
IL13	922	TMEM255A	ENSG00000125355.15	-0.18	0.98	0.74	2	0.54	0.65
IL13	922	PRSS23	ENSG00000150687.11	-0.18	0.31	-0.38	0.00059	-0.52	0.000000054
IL13	922	MELK	ENSG00000165304.7	-0.18	0.85	0.51	0.041	0.22	0.52
IL13	922	VPS37C	ENSG00000167987.10	-0.18	0.56	0.27	0.16	0.09	0.76
IL13	922	C14orf80	ENSG00000185347.17	-0.18	0.95	0.07	0.97	0.03	0.98
IL13	922	COTL1	ENSG00000103187.7	-0.19	0.7	0.43	0.044	0.22	0.42
IL13	922	JAK3	ENSG00000105639.18	-0.19	0.71	0.82	2.1E-07	0.73	0.0000029
IL13	922	ADTRP	ENSG00000111863.12	-0.19	0.97	1.13	0.013	0.2	0.86
IL13	922	ETS1	ENSG00000134954.14	-0.19	0.84	0.32	0.46	0.11	0.85
IL13	922	DRAM1	ENSG00000136048.13	-0.19	0.48	0.48	0.00029	0.16	0.42
IL13	922	VEGFC	ENSG00000150630.3	-0.19	0.22	1.05	5.6E-37	0.86	5.1E-25
IL13	922	FLI1	ENSG00000151702.16	-0.19	0.23	0.26	0.027	0.1	0.61
IL13	922	RASGRP3	ENSG00000152689.17	-0.19	0.94	2.01	1.3E-14	1.47	0.000000034
IL13	922	GPR176	ENSG00000166073.10	-0.19	0.78	0.13	0.87	0.07	0.89
IL13	922	GPHN	ENSG00000171723.15	-0.19	0.6	-0.22	0.46	-0.29	0.11
IL13	922	CDKN3	ENSG00000100526.19	-0.2	0.94	0.1	0.95	0.18	0.82

IL13	922	UCK2	ENSG00000143179.14	-0.2	0.36	0.27	0.1	-0.01	0.98
IL13	922	PHLDA2	ENSG00000181649.5	-0.2	0.9	0.35	0.57	0.22	0.69
IL13	922	PRC1	ENSG00000198901.13	-0.2	0.74	0.21	0.67	-0.1	0.83
IL13	922	HAUS8	ENSG00000131351.14	-0.21	0.77	-0.18	0.81	0.07	0.9
IL13	922	MAD2L1	ENSG00000164109.13	-0.21	0.8	0.18	0.83	-0.14	0.79
IL13	922	RHEBL1	ENSG00000167550.10	-0.21	0.94	0.99	0.13	0.91	0.12
IL13	922	JUNB	ENSG00000171223.5	-0.21	0.82	0.51	0.087	0.4	0.17
IL13	922	LAMB3	ENSG00000196878.14	-0.21	0.5	0.16	0.67	-0.21	0.32
IL13	922	FMN1	ENSG00000248905.8	-0.21	0.93	0.18	0.9	-0.26	0.68
IL13	922	AC005674.2	ENSG00000261490.1	-0.21	0.95	-0.23	0.88	0.04	0.98
IL13	922	MYBPC2	ENSG00000086967.9	-0.22	2	0.72	2	0.21	2
IL13	922	GMNN	ENSG00000112312.9	-0.22	0.91	-0.13	0.93	0.06	0.95
IL13	922	KYNU	ENSG00000115919.14	-0.22	0.83	0.74	0.0053	0.41	0.2
IL13	922	PLAU	ENSG00000122861.15	-0.22	0.11	0.41	0.000043	-0.12	0.46
IL13	922	TMEM164	ENSG00000157600.11	-0.22	0.55	0.43	0.026	0.39	0.028
IL13	922	CDT1	ENSG00000167513.8	-0.22	0.71	0.12	0.89	-0.34	0.19
IL13	922	COL8A2	ENSG00000171812.12	-0.22	0.88	-0.09	0.94	-0.21	0.71
IL13	922	MYOZ2	ENSG00000172399.5	-0.22	0.62	0.13	0.85	0.07	0.88
IL13	922	TRAIP	ENSG00000183763.8	-0.22	0.84	0.03	0.98	-0.25	0.59
IL13	922	HMSD	ENSG00000221887.5	-0.22	0.92	0.52	0.36	0.48	0.29
IL13	922	CFTR	ENSG00000001626.14	-0.23	2	-0.43	2	-0.2	2
IL13	922	PAG1	ENSG00000076641.4	-0.23	0.71	0.89	3.7E-06	0.71	0.00029
IL13	922	BDKRB1	ENSG00000100739.10	-0.23	0.21	-0.14	0.63	-0.62	4.8E-09
IL13	922	VNN1	ENSG00000112299.7	-0.23	0.77	0.79	0.00082	0.65	0.0057
IL13	922	CCNB2	ENSG00000157456.7	-0.23	0.75	-0.03	0.98	0	1
IL13	922	AC091057.3	ENSG00000269974.1	-0.23	2	0.24	2	0.14	2
IL13	922	NAV3	ENSG00000067798.14	-0.24	0.89	0.73	0.064	0.33	0.53
IL13	922	RCL1	ENSG00000120158.11	-0.24	0.16	-0.01	0.99	-0.05	0.86
IL13	922	PODXL	ENSG00000128567.16	-0.24	0.87	1	0.001	-0.27	0.62
IL13	922	EPSTI1	ENSG00000133106.14	-0.24	0.89	0.1	0.95	0	1
IL13	922	FBXO43	ENSG00000156509.13	-0.24	2	0.33	2	-0.3	2
IL13	922	CCDC138	ENSG00000163006.11	-0.24	0.91	0.01	1	-0.35	0.58
IL13	922	ADORA2B	ENSG00000170425.3	-0.24	0.3	-0.23	0.32	-0.46	0.0007
IL13	922	ARHGEF28	ENSG00000214944.9	-0.24	0.78	0.26	0.72	-0.22	0.64
IL13	922	LINC02051	ENSG00000226859.1	-0.24	0.97	0.66	0.55	0.43	0.65
IL13	922	DIAPH3	ENSG00000139734.17	-0.25	0.43	-0.18	0.68	-0.54	0.00085
IL13	922	ATAD2	ENSG00000156802.12	-0.25	0.85	0.27	0.77	-0.22	0.71
IL13	922	H2AFZ	ENSG00000164032.11	-0.25	0.73	0.11	0.93	-0.22	0.62
IL13	922	SERPINB8	ENSG00000166401.14	-0.25	0.44	0.17	0.72	0.04	0.95
IL13	922	HR	ENSG00000168453.14	-0.25	0.96	-0.78	0.44	-0.52	0.57
IL13	922	AC010809.1	ENSG00000259287.2	-0.25	0.97	0.08	0.98	-0.62	0.45
IL13	922	TNFRSF12A	ENSG00000006327.13	-0.26	0.5	0.3	0.35	-0.07	0.9
IL13	922	CSPG5	ENSG00000114646.9	-0.26	0.94	-0.73	0.39	-0.76	0.2
IL13	922	CENPK	ENSG00000123219.12	-0.26	0.86	0.56	0.28	0.06	0.96
IL13	922	ARHGAP18	ENSG00000146376.10	-0.26	0.68	0.28	0.57	-0.08	0.89
IL13	922	SKA1	ENSG00000154839.9	-0.26	0.79	0.01	1	-0.15	0.81
IL13	922	PAQR4	ENSG00000162073.13	-0.26	0.59	0.57	0.0083	0.26	0.39
IL13	922	C1orf112	ENSG00000000460.16	-0.27	0.68	0.04	0.98	-0.09	0.89
IL13	922	PRSS22	ENSG00000005001.9	-0.27	2	-0.27	2	0.06	2
IL13	922	TTLL4	ENSG00000135912.10	-0.27	0.64	0.4	0.29	0.15	0.78
IL13	922	ABL2	ENSG00000143322.19	-0.27	0.52	0.23	0.64	-0.22	0.49
IL13	922	RPL39L	ENSG00000163923.9	-0.27	0.83	-0.21	0.87	-0.25	0.69
IL13	922	CDCA4	ENSG00000170779.10	-0.27	0.45	0.26	0.49	-0.12	0.78
IL13	922	CHST11	ENSG00000171310.10	-0.27	0.78	0.59	0.1	0.08	0.93
IL13	922	MCM5	ENSG00000100297.15	-0.28	0.22	0.26	0.28	-0.11	0.72
IL13	922	RFTN1	ENSG00000131378.13	-0.28	0.06	0.24	0.16	0.1	0.68
IL13	922	ALDH1B1	ENSG00000137124.7	-0.28	0.094	0.05	0.94	-0.21	0.2
IL13	922	AP1S3	ENSG00000152056.16	-0.28	0.61	-0.5	0.1	-0.81	0.00021
IL13	922	SLC35E4	ENSG00000100036.12	-0.29	0.16	-0.08	0.9	-0.3	0.073
IL13	922	DOPEY2	ENSG00000142197.12	-0.29	0.79	0.29	0.76	-0.19	0.77
IL13	922	MB21D1	ENSG00000164430.15	-0.29	0.72	0.2	0.84	0.27	0.54
IL13	922	DEPDC1	ENSG00000024526.16	-0.3	0.79	0.55	0.23	0.01	1
IL13	922	DARS2	ENSG00000117593.9	-0.3	0.092	0	1	-0.2	0.28
IL13	922	SCLY	ENSG00000132330.16	-0.3	0.59	0.31	0.49	0.27	0.43
IL13	922	ACTBL2	ENSG00000169067.3	-0.3	0.56	-0.7	0.0047	-0.72	0.0015

IL13	922	CD3EAP	ENSG00000117877.10	-0.31	0.085	-0.02	0.98	-0.37	0.011
IL13	922	SDC1	ENSG00000115884.10	-0.32	0.51	0.14	0.89	-0.31	0.35
IL13	922	CDK2	ENSG00000123374.10	-0.32	0.013	-0.14	0.61	-0.26	0.042
IL13	922	CSF2	ENSG00000164400.5	-0.32	0.78	2.11	4.1E-12	1.56	0.00000045
IL13	922	NRARP	ENSG00000198435.3	-0.32	0.51	0.3	0.52	-0.36	0.23
IL13	922	PANX1	ENSG00000110218.8	-0.33	0.35	0.51	0.034	0.14	0.73
IL13	922	AC096537.1	ENSG00000233384.2	-0.33	0.73	0.05	0.98	-0.24	0.71
IL13	922	ARHGDIB	ENSG00000111348.8	-0.34	0.0018	0.05	0.92	-0.3	0.0044
IL13	922	SLC20A2	ENSG00000168575.9	-0.34	0.009	0.11	0.77	-0.34	0.0048
IL13	922	SEMA5A	ENSG00000112902.11	-0.35	0.57	-0.08	0.95	-0.2	0.73
IL13	922	GLDC	ENSG00000178445.9	-0.35	0.91	0	2	0.03	0.99
IL13	922	MCM2	ENSG00000073111.13	-0.36	0.17	0.05	0.96	-0.52	0.0055
IL13	922	PTPN3	ENSG00000070159.13	-0.37	0.74	-0.28	0.83	-0.22	0.78
IL13	922	HOXB9	ENSG00000170689.9	-0.38	0.81	0.46	0.67	-0.09	0.94
IL13	922	TMCC3	ENSG00000057704.12	-0.4	0.22	0.1	0.92	-0.2	0.6
IL13	922	ESM1	ENSG00000164283.12	-0.4	0.72	1.05	0.057	-0.33	0.59
IL13	922	NEK2	ENSG00000117650.12	-0.41	0.62	0.33	0.71	-0.08	0.94
IL13	922	APBB2	ENSG00000163697.16	-0.41	0.38	0.19	0.86	-0.36	0.33
IL13	922	NR2C2AP	ENSG00000184162.14	-0.41	0.34	-0.09	0.94	-0.37	0.28
IL13	922	PLAUR	ENSG00000011422.11	-0.42	0.099	0.5	0.031	-0.09	0.86
IL13	922	GRM2	ENSG00000164082.14	-0.42	2	-0.21	2	-0.17	2
IL13	922	CHST2	ENSG00000175040.5	-0.42	0.0067	0.38	0.028	-0.45	0.0014
IL13	922	SLC9A9	ENSG00000181804.14	-0.43	0.56	0.45	0.49	0.49	0.24
IL13	922	CITED4	ENSG00000179862.6	-0.44	0.52	0.52	0.36	0.2	0.78
IL13	922	PEX5L	ENSG00000114757.18	-0.45	0.62	-0.57	0.38	-1.08	0.0036
IL13	922	G0S2	ENSG00000123689.5	-0.45	0.41	0.25	0.8	-0.42	0.31
IL13	922	BARD1	ENSG00000138376.10	-0.45	0.25	0.28	0.66	-0.26	0.53
IL13	922	PMAIP1	ENSG00000141682.11	-0.45	0.0024	0.68	1E-07	0.09	0.77
IL13	922	ZNF215	ENSG00000149054.15	-0.45	0.49	0.36	0.63	0	1
IL13	922	PLCL1	ENSG00000115896.15	-0.46	0.22	-0.94	0.00023	-1.07	0.0000052
IL13	922	ERRFI1	ENSG00000116285.12	-0.46	0.015	-0.04	0.97	-0.22	0.39
IL13	922	PLLP	ENSG00000102934.9	-0.48	0.55	0.12	0.94	-0.46	0.4
IL13	922	ZNF93	ENSG00000184635.15	-0.48	0.076	0.16	0.84	-0.81	0.000054
IL13	922	LINC01121	ENSG00000205054.6	-0.48	2	-0.5	2	-0.62	2
IL13	922	ITGA4	ENSG00000115232.13	-0.49	0.43	-0.27	0.81	-0.7	0.055
IL13	922	GJA1	ENSG00000152661.7	-0.49	0.095	0.11	0.92	-0.58	0.015
IL13	922	BMPER	ENSG00000164619.8	-0.49	0.049	0.16	0.83	-0.4	0.098
IL13	922	AC008543.1	ENSG00000197332.8	-0.49	2	0.03	2	-0.16	2
IL13	922	GABBR2	ENSG00000136928.6	-0.5	0.0015	-0.43	0.014	-0.71	0.00000019
IL13	922	EML5	ENSG00000165521.15	-0.5	0.79	0.4	0.87	-0.1	0.95
IL13	922	AC020571.1	ENSG00000229056.2	-0.5	0.32	1.23	0.12	0.05	0.99
IL13	922	BRIP1	ENSG00000136492.8	-0.51	0.47	0.52	0.43	-0.31	0.64
IL13	922	SERPINE1	ENSG00000106366.8	-0.52	0.0015	0.54	0.0012	-0.3	0.12
IL13	922	TLR1	ENSG00000174125.7	-0.52	0.37	1.43	4.3E-07	1	0.00083
IL13	922	LIF	ENSG00000128342.4	-0.53	0.022	0.96	3.1E-07	-0.21	0.55
IL13	922	UBASH3B	ENSG00000154127.9	-0.53	0.011	0.18	0.75	-0.6	0.0013
IL13	922	HBD	ENSG00000223609.7	-0.53	2	-0.31	2	-0.77	2
IL13	922	C12orf60	ENSG00000182993.3	-0.54	0.29	0.25	0.8	-0.28	0.61
IL13	922	GPRC5A	ENSG00000013588.7	-0.55	0.065	-0.61	0.035	-1.07	0.00000054
IL13	922	KIF21B	ENSG00000116852.14	-0.55	0.27	0.17	0.9	-0.43	0.33
IL13	922	SLC7A7	ENSG00000155465.18	-0.55	0.000096	0.73	2.7E-09	0.18	0.39
IL13	922	ETV4	ENSG00000175832.12	-0.55	0.004	0.61	0.00094	-0.28	0.24
IL13	922	EPHB1	ENSG00000154928.16	-0.56	0.66	0.19	0.94	0.03	0.99
IL13	922	FILIP1L	ENSG00000168386.18	-0.57	0.36	0.24	0.87	-0.47	0.37
IL13	922	PLCXD2	ENSG00000240891.7	-0.58	0.64	0.27	0.9	0.03	0.99
IL13	922	HEPACAM	ENSG00000165478.6	-0.59	0.68	-0.08	0.98	0.02	1
IL13	922	ADGRE2	ENSG00000127507.17	-0.61	0.11	0.13	0.92	-0.1	0.9
IL13	922	TLR6	ENSG00000174130.12	-0.62	0.17	0.59	0.2	0	1
IL13	922	NOG	ENSG00000183691.4	-0.62	0.058	0.01	0.99	-0.55	0.07
IL13	922	HES4	ENSG00000188290.10	-0.62	0.43	-0.05	0.99	-0.78	0.11
IL13	922	CST4	ENSG00000101441.4	-0.63	0.68	0.38	2	-0.84	0.26
IL13	922	AC011447.7	ENSG00000280079.1	-0.63	2	-0.08	2	-0.19	2
IL13	922	DIO2	ENSG00000211448.11	-0.64	0.26	0.7	0.15	-0.13	0.89
IL13	922	ADAMTS6	ENSG00000049192.14	-0.69	0.013	0.24	0.75	-0.89	0.00016
IL13	922	CD274	ENSG00000120217.13	-0.7	0.0056	0.43	0.25	-0.32	0.34

IL13	922	NPAS2	ENSG00000170485.16	-0.7	0.0012	1.04	4.9E-08	0.08	0.88
IL13	922	TFPI2	ENSG00000105825.11	-0.71	1.6E-06	0.34	0.12	-0.46	0.0047
IL13	922	ID3	ENSG00000117318.8	-0.71	0.026	0.52	0.21	-0.45	0.2
IL13	922	RTL3	ENSG00000179300.3	-0.71	0.6	-0.14	2	-0.99	0.17
IL13	922	MARCH4	ENSG00000144583.4	-0.73	0.0006	0.24	0.65	-0.64	0.0024
IL13	922	CORO1A	ENSG00000102879.15	-0.75	0.23	-0.69	0.31	-0.17	0.88
IL13	922	EVI2A	ENSG00000126860.11	-0.8	0.00026	-0.26	0.62	-0.91	0.0000096
IL13	922	MGP	ENSG00000111341.9	-0.83	0.016	-0.33	0.69	-0.99	0.00081
IL13	922	AL021578.1	ENSG00000275894.1	-0.83	0.54	-0.72	2	-0.51	0.68
IL13	922	FLT1	ENSG00000102755.11	-0.87	0.049	0.66	0.21	0.61	0.16
IL13	922	CCDC81	ENSG00000149201.9	-0.96	0.00016	0.03	0.99	-1.21	0.0000005
IL13	922	LINC00704	ENSG00000231298.6	-0.97	0.097	0.35	0.81	-0.72	0.21
IL13	922	CREG2	ENSG00000175874.9	-1	0.26	-0.37	0.87	-0.62	0.5
IL13	922	RND1	ENSG00000172602.9	-1.03	5.5E-11	0.57	0.0004	-0.16	0.58
IL13	922	COL19A1	ENSG00000082293.12	-1.21	0.034	0.66	0.4	-0.56	0.42
IL13	922	C8orf4	ENSG00000176907.4	-1.46	1.5E-13	0.59	0.0084	-1.15	4.3E-09
IL13	922	MCTP1	ENSG00000175471.19	-1.77	1.3E-12	0.23	0.8	-2.1	4.1E-17
IL13	922	HES1	ENSG00000114315.3	-2.04	8E-14	-0.02	0.99	-2.35	6.5E-18
IL13	9	ANOS1	ENSG00000078401.6	-1.79	8.8E-15	-0.39	0.44	-2.27	7.2E-23
IL13	9	PTGIS	ENSG00000124212.5	2.13	2.6E-16	0.04	0.99	2.54	9.1E-24
IL13	9	SPTBN5	ENSG00000137877.9	0.23	0.92	0.09	0.96	0.6	0.17
IL13	9	MSTN	ENSG00000138379.4	0.61	0.83	0.15	2	0	1
IL13	9	CILP	ENSG00000138615.5	-0.11	0.99	-0.53	0.7	-0.66	0.36
IL13	9	TINAGL1	ENSG00000142910.15	-0.48	0.45	-0.08	0.96	-0.21	0.77
IL13	9	CLEC3B	ENSG00000163815.5	-0.53	2	-1.07	2	-0.72	2
IL13	9	SPINT2	ENSG00000167642.12	3.46	8.6E-31	-0.25	0.86	3.24	2.7E-27
IL13	9	ACTN3	ENSG00000248746.5	0.32	0.87	-1.39	0.0061	-0.06	0.98
IL13	7	ANOS1	ENSG00000128510.10	1.29	1.1E-15	-0.87	1.5E-06	0.64	0.00044
IL13	7	TRPC4	ENSG00000133107.14	-0.32	0.68	-0.42	0.43	-1.02	0.00015
IL13	7	IL6	ENSG00000136244.11	2.65	3.6E-36	0.17	0.88	3.05	2.6E-48
IL13	7	GDF6	ENSG00000156466.9	0.44	0.82	-0.28	0.88	-0.11	0.92
IL13	7	RASGRP1	ENSG00000172575.11	-1.19	0.0093	0.36	0.74	-0.67	0.18
IL13	7	CCNE2	ENSG00000175305.17	0.34	0.85	0.82	0.13	0.66	0.2
IL13	7	AP001476.1	ENSG00000224413.1	1.64	0.00019	0.71	0.4	2.15	0.000000069
IL13	116	ANOS1	ENSG00000002933.7	0	2	-0.02	2	-0.01	2
IL13	116	DBNDD1	ENSG00000003249.13	-0.06	0.98	0.24	0.56	-0.01	0.99
IL13	116	THSD7A	ENSG00000005108.15	0.3	0.97	-0.03	1	-0.45	0.74
IL13	116	SYT7	ENSG00000011347.9	0.32	0.9	0.57	0.56	0.48	0.52
IL13	116	SLC7A14	ENSG00000013293.5	1.35	0.000021	0.3	0.79	1.61	0.000000059
IL13	116	PTPRN	ENSG00000054356.13	-0.11	0.94	0.01	1	-0.22	0.48
IL13	116	LZTS1	ENSG00000061337.15	0.79	0.074	-0.1	0.96	0.4	0.49
IL13	116	NGEF	ENSG00000066248.14	-0.38	0.81	0.28	0.88	-0.13	0.9
IL13	116	CRMP1	ENSG00000072832.14	0.33	0.96	-0.33	2	0.23	0.89
IL13	116	SNCB	ENSG00000074317.10	-0.23	2	0.36	2	-0.09	2
IL13	116	LXN	ENSG00000079257.7	0.8	0.000008	-0.07	0.94	0.85	0.00000065
IL13	116	PTPRH	ENSG00000080031.9	-0.02	1	-0.83	0.12	-0.36	0.61
IL13	116	TCF7	ENSG00000081059.19	0.25	0.72	0.49	0.11	0.18	0.72
IL13	116	ABCB1	ENSG00000085563.14	-0.13	2	0.05	2	-0.09	2
IL13	116	STX1B	ENSG00000099365.10	0.65	0.099	0.33	0.68	0.63	0.065
IL13	116	TIMP1	ENSG00000102265.11	0.4	0.4	-0.03	0.99	0.42	0.21
IL13	116	RASL12	ENSG00000103710.10	0.38	0.76	-0.37	0.77	0.73	0.097
IL13	116	CTSH	ENSG00000103811.15	0.06	1	0.2	0.91	0.36	0.61
IL13	116	TRPA1	ENSG00000104321.10	-1.18	5.4E-07	-0.13	0.91	-1.02	0.000017
IL13	116	TMEM176B	ENSG00000106565.17	0	2	-0.02	2	-0.01	2
IL13	116	ABI3	ENSG00000108798.8	-0.21	0.97	-0.7	0.51	-0.73	0.32
IL13	116	EFNB3	ENSG00000108947.4	0.44	0.34	-0.14	0.92	0.05	0.95
IL13	116	GLI1	ENSG00000111087.9	1.49	0.0037	-0.41	0.82	1.6	0.00069
IL13	116	FAM46A	ENSG00000112773.15	0.42	0.057	-0.27	0.44	0.26	0.32
IL13	116	PPL	ENSG00000118898.15	0.07	1	-1.06	0.0036	-0.79	0.032
IL13	116	DUSP4	ENSG00000120875.8	0.9	8.1E-10	-0.28	0.36	0.81	0.000000041
IL13	116	LRAT	ENSG00000121207.11	-0.65	2	-0.52	2	-0.48	2
IL13	116	TWIST1	ENSG00000122691.12	0.62	9.3E-07	-0.04	0.96	0.86	3.2E-13
IL13	116	LYPD3	ENSG00000124466.8	0.39	0.6	-0.02	0.99	0.48	0.24
IL13	116	ID1	ENSG00000125968.8	-0.72	0.17	-0.37	0.72	-1.11	0.0022
IL13	116	VGF	ENSG00000128564.6	-0.13	0.96	0.11	0.93	-0.23	0.63

IL13	116	FCHO1	ENSG00000130475.14	-0.6	0.0027	-0.35	0.23	-0.6	0.0015
IL13	116	LSP1	ENSG00000130592.15	-0.11	0.98	-0.95	0.0015	-0.54	0.11
IL13	116	MPP1	ENSG00000130830.14	1.05	4E-33	-0.34	0.0037	1.03	5.3E-32
IL13	116	RAI2	ENSG00000131831.17	-0.5	0.83	-0.3	2	-0.39	0.76
IL13	116	EPHB2	ENSG00000133216.16	-0.52	0.00055	0.32	0.11	-0.19	0.39
IL13	116	RERG	ENSG00000134533.6	-1.24	0.086	-0.04	0.99	-0.05	0.98
IL13	116	SPOCD1	ENSG00000134668.12	-0.46	0.000085	0.32	0.025	-0.49	0.000011
IL13	116	ITGA7	ENSG00000135424.15	-0.1	0.97	-0.15	0.9	-0.26	0.56
IL13	116	TMOD1	ENSG00000136842.13	0.68	0.12	-0.04	0.99	1.35	0.0000024
IL13	116	TACC2	ENSG00000138162.18	0.48	0.0075	0.05	0.95	0.45	0.0076
IL13	116	SDSL	ENSG00000139410.14	-0.12	0.98	-0.32	0.81	-0.22	0.8
IL13	116	PCSK6	ENSG00000140479.16	0.83	0.21	0.82	2	0.85	0.11
IL13	116	ECM1	ENSG00000143369.14	-0.28	0.038	-0.26	0.076	-0.44	0.000049
IL13	116	CTSK	ENSG00000143387.12	0.15	0.76	0.1	0.88	0.17	0.49
IL13	116	SYT14	ENSG00000143469.18	-0.45	0.55	0.67	0.16	-0.14	0.88
IL13	116	SDK1	ENSG00000146555.18	0.43	0.47	-0.06	0.97	0.5	0.18
IL13	116	LYPD1	ENSG00000150551.10	1.51	1.4E-17	-0.11	0.91	1.48	3.4E-17
IL13	116	NR4A2	ENSG00000153234.13	-0.7	0.0027	-0.48	0.11	-1.24	4.7E-10
IL13	116	TIAM1	ENSG00000156299.13	0.56	0.75	0.01	1	0.78	0.32
IL13	116	SUSD3	ENSG00000157303.10	0.11	0.97	0.05	0.97	-0.06	0.93
IL13	116	DUSP2	ENSG00000158050.4	-0.18	0.97	-0.49	0.63	-0.58	0.33
IL13	116	CLSTN2	ENSG00000158258.16	-0.74	0.13	-0.32	0.79	-1.09	0.0023
IL13	116	TPPP3	ENSG00000159713.10	-0.01	1	0.03	0.99	-0.5	0.34
IL13	116	DRAXIN	ENSG00000162490.6	0.09	0.99	0.55	0.44	0.05	0.98
IL13	116	KIAA1522	ENSG00000162522.10	-0.28	0.34	-0.08	0.93	-0.43	0.017
IL13	116	KCNF1	ENSG00000162975.4	-0.5	0.74	-0.19	0.93	-0.87	0.15
IL13	116	TLR3	ENSG00000164342.12	0.22	0.96	0.61	0.45	0.5	0.43
IL13	116	BAALC	ENSG00000164929.16	-0.88	0.00016	-0.47	0.17	-1.1	0.00000034
IL13	116	PKD1L2	ENSG00000166473.17	-0.1	2	0.14	2	0.28	2
IL13	116	KRT80	ENSG00000167767.13	-0.41	0.35	-0.12	0.92	-0.57	0.04
IL13	116	VWCE	ENSG00000167992.12	0.38	0.012	0	1	0.42	0.0026
IL13	116	KCTD19	ENSG00000168676.10	-0.19	0.98	0.12	0.96	-0.32	0.78
IL13	116	TMEM266	ENSG00000169758.12	-0.34	0.92	0.39	0.85	0.68	0.38
IL13	116	REPS2	ENSG00000169891.17	0.02	1	-0.2	0.69	-0.27	0.31
IL13	116	LONRF2	ENSG00000170500.12	0.8	0.52	-0.49	2	0.17	0.91
IL13	116	HTRA3	ENSG00000170801.9	-0.63	0.000031	-0.05	0.95	-0.76	0.000000076
IL13	116	NPTX1	ENSG00000171246.5	-0.66	0.0013	0.01	1	-0.95	0.000000098
IL13	116	SYT12	ENSG00000173227.13	-0.84	0.014	-0.63	0.12	-1.29	0.000016
IL13	116	SNCG	ENSG00000173267.13	0.12	0.98	-0.27	0.82	-0.05	0.97
IL13	116	REP15	ENSG00000174236.3	-0.16	0.99	0.43	0.84	0.27	0.85
IL13	116	CAMK1D	ENSG00000183049.12	0.35	0.16	0.24	0.52	0.61	0.00029
IL13	116	PTGDR2	ENSG00000183134.4	2.93	6.3E-31	-0.91	0.021	2.72	8.3E-27
IL13	116	C2CD4C	ENSG00000183186.7	0.51	0.78	0.33	0.88	-0.4	0.64
IL13	116	CHST6	ENSG00000183196.8	-0.19	0.97	0.08	0.97	-0.5	0.44
IL13	116	FAM46C	ENSG00000183508.4	-1.14	0.017	0.69	0.29	-1.36	0.0014
IL13	116	OSBP2	ENSG00000184792.15	-0.15	0.93	0.02	0.99	-0.16	0.77
IL13	116	TCN2	ENSG00000185339.8	-0.1	0.96	-0.39	0.15	-0.17	0.64
IL13	116	AHNAK2	ENSG00000185567.6	-0.29	0.86	0.01	1	-0.44	0.41
IL13	116	HYAL3	ENSG00000186792.16	0.06	0.99	-0.2	0.83	-0.06	0.94
IL13	116	TNFRSF18	ENSG00000186891.13	0.42	0.83	0.31	0.88	0.21	0.88
IL13	116	TRPV2	ENSG00000187688.14	0.05	0.98	0.03	0.98	0.16	0.56
IL13	116	PALM3	ENSG00000187867.8	1.09	0.21	-0.62	0.66	1.04	0.15
IL13	116	SPRED3	ENSG00000188766.12	-0.08	0.98	0.39	0.24	0.19	0.63
IL13	116	MMP1	ENSG00000196611.4	0.2	0.68	0.47	0.019	0.42	0.025
IL13	116	ADA	ENSG00000196839.12	-0.23	0.59	-0.16	0.79	-0.2	0.49
IL13	116	HIST1H4C	ENSG00000197061.4	-0.18	2	-0.28	2	-0.26	2
IL13	116	SVIL	ENSG00000197321.14	-0.45	0.41	0.45	0.43	-0.1	0.9
IL13	116	PTMAP2	ENSG00000197744.5	0.18	0.97	0.62	0.43	0.6	0.3
IL13	116	CARD11	ENSG00000198286.9	-0.49	0.2	0.02	0.99	-0.46	0.15
IL13	116	SMOC1	ENSG00000198732.10	-1.16	1.4E-16	0.24	0.44	-1.26	9.4E-20
IL13	116	SNORA73B	ENSG00000200087.1	0.24	0.97	-0.1	0.97	0.34	0.76
IL13	116	NHSL2	ENSG00000204131.9	0.35	0.91	0.51	0.72	0.55	0.5
IL13	116	HLA-A	ENSG00000206503.12	-0.11	0.93	0.12	0.87	0.07	0.89
IL13	116	MT-TT	ENSG00000210195.2	0.54	2	0.12	2	0.83	2
IL13	116	MT-RNR1	ENSG00000211459.2	-0.09	0.83	-0.14	0.47	-0.15	0.24

IL13	116	MIAT	ENSG00000225783.6	0.04	1	0.66	0.096	0.36	0.45
IL13	116	AP000692.1	ENSG00000228107.1	-0.64	0.6	-0.18	0.94	-0.69	0.36
IL13	116	GAS6-AS1	ENSG00000233695.2	-0.2	0.9	-0.57	0.12	-0.54	0.088
IL13	116	CTBP2P8	ENSG00000234383.1	-0.47	0.85	-0.27	0.92	-0.16	0.93
IL13	116	AC073046.1	ENSG00000235499.1	0.07	1	0.12	0.97	0.31	0.76
IL13	116	CFB	ENSG00000243649.8	-0.01	1	0.39	0.3	0.41	0.16
IL13	116	AC105285.1	ENSG00000245213.6	0.39	0.66	-0.43	0.59	0.55	0.2
IL13	116	AC022075.1	ENSG00000245648.1	0.97	0.036	-0.22	0.9	0.89	0.038
IL13	116	TMEM158	ENSG00000249992.1	0.84	0.000049	0.39	0.25	1.03	0.000000069
IL13	116	AC069360.1	ENSG00000250041.2	0.09	1	0.33	2	0.58	0.64
IL13	116	SCARNA13	ENSG00000252481.1	0.43	0.85	0.55	0.7	0.34	0.76
IL13	116	AC144548.1	ENSG00000258011.2	-0.01	1	0.08	0.97	-0.36	0.63
IL13	116	SNORD3A	ENSG00000263934.4	0.37	0.93	0.32	0.91	0.31	0.83
IL13	116	RASSF5	ENSG00000266094.7	0.65	0.032	0.18	0.87	1.4	3.2E-10
IL13	116	AC011498.2	ENSG00000267030.1	0.61	0.75	0.28	2	0.03	1
IL13	116	AC112220.4	ENSG00000271643.1	-0.11	0.99	0.04	0.99	-0.09	0.93
IL13	116	HIST1H2BH	ENSG00000275713.2	-0.21	0.97	-0.15	0.95	0.06	0.97
IL13	116	DACH1	ENSG00000276644.4	2.63	2.7E-14	-1.15	0.045	1.82	0.00000061
IL13	116	AC013553.3	ENSG00000277351.1	-0.04	2	-0.02	2	-0.03	2
IL13	116	AC027682.7	ENSG00000280214.1	0.72	0.71	0.28	2	0.95	0.31
IL13	360	ANOS1	ENSG00000003987.13	0.2	0.98	0.58	2	0.63	0.53
IL13	360	SLC7A2	ENSG00000003989.17	-0.1	0.98	0.98	0.0018	0.69	0.034
IL13	360	MCUB	ENSG00000005059.15	-0.08	0.96	0.02	0.99	0.04	0.93
IL13	360	MAP3K14	ENSG00000006062.14	0.41	0.15	0.93	8.7E-07	1.2	4.5E-12
IL13	360	TMEM132A	ENSG00000006118.14	0.12	0.88	0.97	4.7E-14	0.91	4.3E-13
IL13	360	CX3CL1	ENSG00000006210.6	0.78	0.038	1	0.0026	1.77	4.2E-11
IL13	360	CACNA1G	ENSG00000006283.17	-0.53	0.63	0.3	0.86	0.28	0.77
IL13	360	MAP3K9	ENSG00000006432.15	0.26	0.97	-0.16	0.95	-0.04	0.98
IL13	360	E2F2	ENSG00000007968.6	-0.05	1	0.32	0.88	0.37	0.73
IL13	360	PLEKHG6	ENSG00000008323.15	-0.27	0.97	-0.57	0.74	-0.22	0.9
IL13	360	IL32	ENSG00000008517.16	-0.04	1	0.86	0.0018	0.86	0.00067
IL13	360	ETV7	ENSG00000010030.13	0.8	0.12	1.93	1.9E-08	2.31	5.6E-13
IL13	360	ANLN	ENSG00000011426.10	-0.27	0.8	0.38	0.54	-0.03	0.98
IL13	360	TACC3	ENSG00000013810.18	-0.03	1	0.02	0.99	0.01	0.99
IL13	360	CCDC88C	ENSG00000015133.18	0.26	0.97	-0.31	0.89	0.24	0.87
IL13	360	CD74	ENSG00000019582.14	-0.04	1	0.71	0.0097	0.94	0.000029
IL13	360	NRXN3	ENSG00000021645.18	-0.59	0.57	-0.45	0.73	-1.38	0.0038
IL13	360	BIRC3	ENSG00000023445.13	1.14	3.5E-07	2.93	4.6E-48	3.58	6.6E-74
IL13	360	TYMP	ENSG00000025708.13	-0.1	0.98	0.59	0.27	0.7	0.068
IL13	360	TOMM34	ENSG00000025772.7	-0.16	0.5	-0.05	0.92	-0.15	0.37
IL13	360	TNFRSF1B	ENSG00000028137.18	0.23	0.64	1.07	1.1E-09	1.31	1.1E-15
IL13	360	POU2F2	ENSG00000028277.21	-0.43	0.074	0.73	0.00011	-0.35	0.14
IL13	360	ARNTL2	ENSG00000029153.14	0.99	1.5E-09	1.49	2.5E-21	2.36	1.1E-56
IL13	360	RIPOR3	ENSG00000042062.11	0.38	0.38	1.07	1.9E-06	1.68	5.2E-17
IL13	360	EPHA3	ENSG00000044524.10	0.17	2	0.09	2	0.02	2
IL13	360	HDAC9	ENSG00000048052.21	-0.2	0.87	0.79	0.0038	0.27	0.53
IL13	360	NFE2L3	ENSG00000050344.8	0.21	0.34	0.76	4.7E-12	0.93	4.1E-19
IL13	360	NRIP2	ENSG00000053702.14	1.65	0.041	0.18	2	1.92	0.0072
IL13	360	CYFIP2	ENSG00000055163.19	0.09	0.97	-0.03	0.98	0.41	0.066
IL13	360	TRAF1	ENSG00000056558.10	-0.2	0.72	1.9	3.2E-38	1.54	6.2E-26
IL13	360	DCBLD2	ENSG00000057019.15	0.38	0.22	0.93	1.3E-06	1.14	1.6E-10
IL13	360	LAMC2	ENSG00000058085.14	-0.47	0.36	0.93	0.0022	0.17	0.81
IL13	360	DGAT2	ENSG00000062282.14	-0.34	0.071	0.84	1.4E-11	1	1E-17
IL13	360	POLD1	ENSG00000062822.12	-0.04	0.99	0.18	0.6	0.01	0.99
IL13	360	CDON	ENSG00000064309.14	0.42	0.31	0.39	0.38	0.83	0.00043
IL13	360	CHI3L2	ENSG00000064886.13	1.26	2.9E-20	0.13	0.83	2.3	4.9E-69
IL13	360	MCM10	ENSG00000065328.16	0.16	0.98	0.96	0.035	0.37	0.6
IL13	360	ASPM	ENSG00000066279.17	-0.04	1	0.27	0.88	-0.02	0.99
IL13	360	STAG3	ENSG00000066923.17	0.28	0.91	-0.01	1	-0.1	0.93
IL13	360	PKM	ENSG00000067225.17	-0.11	0.86	0.21	0.4	0.24	0.13
IL13	360	GAL	ENSG00000069482.6	-0.23	0.91	0.45	0.51	0.15	0.87
IL13	360	TRIP13	ENSG00000071539.13	-0.13	0.87	0.18	0.65	-0.12	0.69
IL13	360	TP63	ENSG00000073282.12	0	1	0.51	2	0.85	0.076
IL13	360	GTSE1	ENSG00000075218.18	-0.31	0.5	0.41	0.19	-0.23	0.54
IL13	360	WDR62	ENSG00000075702.16	0.05	1	0.33	0.67	0.53	0.15

IL13	360	SPAG5	ENSG00000076382.16	-0.13	0.95	0.37	0.31	0.02	0.98
IL13	360	NFKB2	ENSG00000077150.18	0.02	1	1.08	9.4E-24	1.11	5.4E-26
IL13	360	SYNJ2	ENSG00000078269.14	0.1	0.96	0.56	0.018	0.48	0.029
IL13	360	CD82	ENSG00000085117.11	-0.17	0.88	0.63	0.011	0.38	0.18
IL13	360	RAD54L	ENSG00000085999.11	-0.23	0.91	0.27	0.83	-0.09	0.92
IL13	360	ACHE	ENSG00000087085.13	-0.43	0.64	0.34	0.76	0.48	0.37
IL13	360	TPX2	ENSG00000088325.15	-0.05	0.98	0.15	0.68	0.01	0.98
IL13	360	BIRC5	ENSG00000089685.14	-0.23	0.89	0.15	0.92	-0.1	0.9
IL13	360	RGS1	ENSG00000090104.11	0.09	2	0	2	0.12	2
IL13	360	KIF4A	ENSG00000090889.11	-0.41	0.28	-0.03	0.98	-0.27	0.47
IL13	360	CDC45	ENSG00000093009.9	3.67	6.4E-65	0.4	0.49	3.93	8.7E-75
IL13	360	PTGS1	ENSG00000095303.14	-0.68	5.5E-06	-0.42	0.026	-0.94	1.3E-11
IL13	360	DERL3	ENSG00000099958.14	0.5	0.72	-0.39	0.8	0.55	0.45
IL13	360	GGT5	ENSG00000099998.17	0.18	0.84	-0.08	0.94	0.34	0.21
IL13	360	CYTH4	ENSG00000100055.20	-0.36	0.72	0.28	0.79	-0.17	0.85
IL13	360	PLA2G3	ENSG00000100078.3	0.43	0.89	0.49	2	0.48	0.68
IL13	360	CENPM	ENSG00000100162.14	-0.08	0.99	0.12	0.95	-0.53	0.29
IL13	360	PAPLN	ENSG00000100767.15	0.28	0.72	0.43	0.33	0.86	0.00063
IL13	360	NFKBIA	ENSG00000100906.10	-0.01	1	0.6	0.0014	0.62	0.00031
IL13	360	MMP9	ENSG00000100985.7	-0.24	0.91	2.05	5.3E-11	2.12	1.7E-12
IL13	360	MYBL2	ENSG00000101057.15	-0.45	0.44	0.51	0.28	-0.13	0.87
IL13	360	SLCO4A1	ENSG00000101187.15	0.51	0.75	1.19	0.11	1.25	0.044
IL13	360	NTSR1	ENSG00000101188.4	0.18	2	0.83	2	-0.34	2
IL13	360	FAM83D	ENSG00000101447.14	-0.03	1	-0.28	0.54	-0.29	0.34
IL13	360	LIPG	ENSG00000101670.11	-1.6	1.2E-17	0.47	0.097	-1.51	4.6E-16
IL13	360	KLHL4	ENSG00000102271.13	0.4	0.62	-0.51	0.41	0.09	0.92
IL13	360	HTR2A	ENSG00000102468.10	-0.4	0.94	-1.13	2	-0.46	0.74
IL13	360	IGDCC4	ENSG00000103742.11	0.42	0.49	0.91	0.0041	1.19	0.0000082
IL13	360	WISP1	ENSG00000104415.13	0.11	0.98	-0.13	0.94	0.48	0.32
IL13	360	KCNN4	ENSG00000104783.11	0.08	0.99	0.34	0.75	0.34	0.58
IL13	360	RELB	ENSG00000104856.13	0	1	0.57	0.0027	0.41	0.035
IL13	360	IL4I1	ENSG00000104951.15	0.74	0.00033	1.36	4.2E-14	2.45	1.7E-49
IL13	360	IL27RA	ENSG00000104998.3	0.02	1	0.87	3.5E-14	0.9	5.5E-16
IL13	360	ASF1B	ENSG00000105011.8	-0.3	0.63	0.16	0.87	-0.35	0.31
IL13	360	EBI3	ENSG00000105246.5	-0.14	0.96	0.72	0.016	0.74	0.005
IL13	360	LIG1	ENSG00000105486.13	-0.12	0.7	0.06	0.91	-0.21	0.079
IL13	360	PLA2G4C	ENSG00000105499.13	-0.34	0.016	1.09	7.3E-28	0.6	0.000000012
IL13	360	CADM4	ENSG00000105767.2	-0.19	0.77	-0.04	0.97	0.06	0.91
IL13	360	DNAH11	ENSG00000105877.17	-0.61	0.54	0.37	0.81	-0.03	0.98
IL13	360	DKK1	ENSG00000107984.9	-1.26	1.3E-15	0.06	0.95	-1.48	1.3E-21
IL13	360	RASD1	ENSG00000108551.4	-1.11	1.1E-08	-0.06	0.96	-1.42	3.3E-14
IL13	360	CCL2	ENSG00000108691.9	2.35	1.4E-21	1.4	3.2E-07	3.59	6.6E-51
IL13	360	ABCC3	ENSG00000108846.15	-0.23	0.44	0.19	0.57	-0.17	0.48
IL13	360	GALNT7	ENSG00000109586.11	0.94	0.0001	0.31	0.6	1.22	0.000000035
IL13	360	NECTIN1	ENSG00000110400.10	0.3	0.14	0.24	0.36	0.36	0.026
IL13	360	KIAA1549L	ENSG00000110427.14	-0.11	0.98	0.41	0.61	-0.08	0.94
IL13	360	SLC35F2	ENSG00000110660.14	-0.33	0.15	0.17	0.72	-0.14	0.64
IL13	360	FOXM1	ENSG00000111206.12	-0.32	0.46	0.01	1	-0.22	0.55
IL13	360	RAD51AP1	ENSG00000111247.14	-0.19	0.94	0.28	0.79	0.12	0.9
IL13	360	OAS3	ENSG00000111331.12	0.04	1	0.65	0.45	0.58	0.38
IL13	360	CDCA3	ENSG00000111665.11	-0.46	0.51	0	1	-0.05	0.96
IL13	360	CRYBG1	ENSG00000112297.14	2.6	2.3E-24	1.51	9.1E-08	3.71	2.1E-50
IL13	360	TTK	ENSG00000112742.9	-0.03	1	0.4	0.47	0.22	0.69
IL13	360	KIF20A	ENSG00000112984.11	-0.55	0.2	0.19	0.87	-0.46	0.23
IL13	360	HBEGF	ENSG00000113070.7	-0.67	0.00034	1.08	2.9E-11	0.08	0.88
IL13	360	LMNB1	ENSG00000113368.11	-0.21	0.56	0.42	0.018	0.05	0.91
IL13	360	WWC1	ENSG00000113645.14	-0.01	1	0.88	4.3E-07	1.02	3.5E-10
IL13	360	DPYSL3	ENSG00000113657.12	-0.43	0.032	-0.02	0.98	-0.11	0.77
IL13	360	CDX1	ENSG00000113722.16	0.55	0.48	0.25	0.87	0.63	0.19
IL13	360	C3orf52	ENSG00000114529.12	-0.14	0.91	0.62	0.0022	-0.04	0.95
IL13	360	CENPA	ENSG00000115163.14	-0.3	0.89	0.2	0.91	-0.51	0.4
IL13	360	IFIH1	ENSG00000115267.5	0.51	0.38	1.28	0.00004	1.47	0.00000026
IL13	360	ST6GALNAC5	ENSG00000117069.14	-0.99	1.3E-11	0.32	0.18	-0.77	0.00000028
IL13	360	CDC20	ENSG00000117399.13	-0.47	0.27	-0.06	0.97	-0.52	0.1
IL13	360	PIK3R3	ENSG00000117461.14	0.55	0.023	0.37	0.29	1.12	8.6E-10

IL13	360	TNFSF4	ENSG00000117586.10	-0.02	1	0.95	0.000035	0.76	0.00093
IL13	360	RPS6KA1	ENSG00000117676.13	-0.21	0.84	0.16	0.86	-0.17	0.73
IL13	360	CENPF	ENSG00000117724.12	-0.15	0.97	0.62	0.23	0.11	0.91
IL13	360	TNFAIP3	ENSG00000118503.14	-0.27	0.61	0.18	0.81	-0.06	0.92
IL13	360	MOB3B	ENSG00000120162.9	0.23	0.89	2.56	4.6E-30	2.48	3.7E-29
IL13	360	TNFSF18	ENSG00000120337.8	0.95	0.18	-0.04	0.99	1.63	0.00058
IL13	360	KIAA1217	ENSG00000120549.17	-0.13	0.96	0.41	0.41	0.17	0.77
IL13	360	TNFSF11	ENSG00000120659.14	-0.43	0.85	0.36	0.87	0.05	0.98
IL13	360	SOCS2	ENSG00000120833.13	-0.05	0.99	0.4	0.049	0.34	0.08
IL13	360	NCAPH	ENSG00000121152.9	-0.32	0.75	0.57	0.21	0.24	0.7
IL13	360	MND1	ENSG00000121211.7	-0.57	0.48	0.16	0.93	0.02	0.99
IL13	360	EGR2	ENSG00000122877.15	0.79	0.31	0.19	0.94	1.28	0.0079
IL13	360	ZWINT	ENSG00000122952.16	0.29	0.72	0.31	0.66	0.32	0.46
IL13	360	CIT	ENSG00000122966.15	0.24	0.78	0.26	0.71	0.63	0.015
IL13	360	BHLHE41	ENSG00000123095.5	-1.41	1.3E-30	0.07	0.92	-1.73	5.7E-45
IL13	360	HJURP	ENSG00000123485.11	0.02	1	0.56	0.02	0.56	0.0085
IL13	360	CKS2	ENSG00000123975.4	-0.2	0.49	-0.03	0.97	-0.04	0.93
IL13	360	CXCL6	ENSG00000124875.9	1.63	1.1E-10	1.9	4.8E-14	3.31	3.8E-45
IL13	360	C17orf53	ENSG00000125319.14	-0.26	0.8	0.23	0.79	-0.25	0.61
IL13	360	IRF1	ENSG00000125347.13	0.28	0.2	1.14	4.7E-20	1.04	1.9E-17
IL13	360	C3	ENSG00000125730.16	0.28	0.68	0.1	0.93	0.45	0.12
IL13	360	BMP2	ENSG00000125845.6	-0.87	3.1E-09	0.85	1.1E-08	-0.55	0.00059
IL13	360	FAM110A	ENSG00000125898.12	-0.21	0.87	0.17	0.88	-0.29	0.52
IL13	360	GDF5	ENSG00000125965.8	-0.13	0.97	-0.15	0.9	0.18	0.75
IL13	360	TRAF2	ENSG00000127191.17	-0.03	1	0.54	0.0096	0.6	0.00083
IL13	360	CHTF18	ENSG00000127586.16	0.14	0.88	0.22	0.58	0.29	0.21
IL13	360	POM121L9P	ENSG00000128262.8	0.68	0.031	0.36	0.55	1.4	7.4E-10
IL13	360	RIBC2	ENSG00000128408.8	0.15	0.99	0.38	0.84	-0.38	0.67
IL13	360	STRIP2	ENSG00000128578.9	0.15	0.93	0.54	0.079	0.83	0.00022
IL13	360	ARHGAP22	ENSG00000128805.14	-0.06	0.97	0.31	0.043	-0.05	0.88
IL13	360	DLL4	ENSG00000128917.6	-0.32	0.82	-0.04	0.99	-0.71	0.1
IL13	360	E2F8	ENSG00000129173.12	0.6	0.68	0.74	0.5	1.16	0.065
IL13	360	PIMREG	ENSG00000129195.15	-0.15	0.97	0.4	0.59	0.1	0.93
IL13	360	RHBDF2	ENSG00000129667.12	-0.17	0.81	0.64	0.001	0.31	0.21
IL13	360	SGO1	ENSG00000129810.14	-0.4	0.75	0.28	0.85	0.07	0.96
IL13	360	CRACR2A	ENSG00000130038.9	0.1	0.97	-0.13	0.91	0.21	0.63
IL13	360	EPS8L1	ENSG00000131037.14	-0.28	0.81	0.13	0.93	-0.02	0.99
IL13	360	TRAF3	ENSG00000131323.14	-0.06	0.98	0.27	0.44	0.21	0.47
IL13	360	ZSWIM4	ENSG00000132003.9	-0.46	0.3	0.64	0.056	0.19	0.73
IL13	360	TRIM47	ENSG00000132481.6	0.21	0.7	1.47	1.2E-20	1.51	7E-23
IL13	360	TESMIN	ENSG00000132749.10	-0.02	1	-0.36	0.67	0.01	1
IL13	360	CHI3L1	ENSG00000133048.12	-0.2	0.7	0.52	0.0096	0.13	0.73
IL13	360	DCLK1	ENSG00000133083.14	2.02	9.9E-29	1.16	1.3E-08	3.26	1.4E-76
IL13	360	RFC3	ENSG00000133119.12	0.17	0.96	0.26	0.83	0.28	0.65
IL13	360	MICALCL	ENSG00000133808.4	-0.31	0.7	-0.14	0.91	-0.57	0.093
IL13	360	MICAL2	ENSG00000133816.13	-0.43	0.58	-0.09	0.96	-0.69	0.068
IL13	360	TTC9	ENSG00000133985.2	0.59	0.44	0.77	0.18	1.65	0.0000017
IL13	360	CCNB1	ENSG00000134057.14	-0.08	0.97	0.1	0.88	-0.22	0.4
IL13	360	BHLHE40	ENSG00000134107.4	-0.46	0.026	0.56	0.0035	-0.05	0.93
IL13	360	IL15RA	ENSG00000134470.20	0.34	0.18	1.21	1.1E-16	1.44	7E-25
IL13	360	DOCK2	ENSG00000134516.15	0.1	0.98	1.11	8.4E-07	0.88	0.00015
IL13	360	CDCA8	ENSG00000134690.10	-0.26	0.81	0.36	0.55	0.08	0.92
IL13	360	SLC37A2	ENSG00000134955.11	-0.25	0.51	-0.06	0.94	-0.49	0.0088
IL13	360	NT5E	ENSG00000135318.11	0.11	0.95	0.46	0.05	0.31	0.21
IL13	360	TROAP	ENSG00000135451.12	0.02	1	0.61	0.17	-0.43	0.37
IL13	360	ESPL1	ENSG00000135476.11	-0.1	0.98	0.48	0.19	0.62	0.016
IL13	360	PKIB	ENSG00000135549.14	0.42	0.31	-0.21	0.81	0.58	0.03
IL13	360	CPM	ENSG00000135678.11	-0.63	4.7E-14	0.89	7.3E-32	-0.1	0.55
IL13	360	TNS3	ENSG00000136205.16	-0.18	0.9	0.28	0.65	0.06	0.93
IL13	360	C7orf69	ENSG00000136275.10	0.07	2	0.53	2	0.1	2
IL13	360	ZFHX2	ENSG00000136367.13	0.25	0.89	0.83	0.037	1.02	0.0017
IL13	360	LIMD2	ENSG00000136490.8	-0.03	1	0.38	0.33	0.19	0.68
IL13	360	IL33	ENSG00000137033.11	-1.11	6E-08	0.83	0.00023	-0.48	0.062
IL13	360	ARHGEF39	ENSG00000137135.17	0.03	1	0.01	1	-0.06	0.96
IL13	360	HMGA1	ENSG00000137309.19	-0.23	0.22	0.17	0.52	-0.11	0.65

IL13	360	TLR2	ENSG00000137462.6	-0.02	1	1.08	6.9E-08	0.62	0.0048
IL13	360	CENPO	ENSG00000138092.10	0.08	0.97	-0.06	0.94	0.05	0.93
IL13	360	LOXL4	ENSG00000138131.3	-0.21	0.78	-0.12	0.9	-0.11	0.84
IL13	360	MYPN	ENSG00000138347.15	0.26	0.85	0.42	0.49	0.09	0.93
IL13	360	CENPE	ENSG00000138778.11	0.21	0.95	0.53	0.49	0.2	0.84
IL13	360	FRMD6	ENSG00000139926.15	-0.16	0.88	0.32	0.44	0.04	0.95
IL13	360	TICRR	ENSG00000140534.13	-0.72	0.061	0.26	0.78	-0.2	0.76
IL13	360	NTRK3	ENSG00000140538.16	2.56	0.00054	1.04	0.26	3.23	0.0000068
IL13	360	TAF4B	ENSG00000141384.12	0.34	0.48	0.72	0.0044	0.65	0.006
IL13	360	SLC14A1	ENSG00000141469.16	-0.03	1	0.08	0.94	-0.32	0.25
IL13	360	EPHA2	ENSG00000142627.12	0	1	0.15	0.72	0.06	0.87
IL13	360	PLK4	ENSG00000142731.10	-0.22	0.93	0.54	0.41	0.21	0.8
IL13	360	KIF2C	ENSG00000142945.12	-0.41	0.31	0.42	0.27	-0.09	0.89
IL13	360	NUF2	ENSG00000143228.12	-0.57	0.27	0.44	0.48	-0.09	0.93
IL13	360	VASH2	ENSG00000143494.15	0.63	0.26	0.66	0.23	1.76	0.00000001
IL13	360	CNIH3	ENSG00000143786.7	-0.65	9.1E-09	0.28	0.079	-0.49	0.000025
IL13	360	FANCD2	ENSG00000144554.10	0.31	0.55	0.36	0.4	0.2	0.67
IL13	360	LRIG1	ENSG00000144749.13	-0.15	0.9	0.98	4.1E-07	0.84	0.00001
IL13	360	NCEH1	ENSG00000144959.9	0.16	0.84	1.27	1.1E-16	1.26	4.4E-17
IL13	360	ANK2	ENSG00000145362.17	1.02	0.0067	0.34	0.74	2.16	3.4E-13
IL13	360	PLK2	ENSG00000145632.14	0.56	0.00017	-0.34	0.1	0.49	0.0012
IL13	360	CRHBP							
IL13	360	NFKBIE	ENSG00000146232.15	0.11	0.97	0.87	0.00077	0.89	0.00016
IL13	360	MTFR2	ENSG00000146410.11	0.34	0.87	0.22	0.91	-0.27	0.77
IL13	360	CDCA5	ENSG00000146670.9	-0.12	0.96	0.24	0.68	0	1
IL13	360	CHRNB3	ENSG00000147432.6	-0.24	2	0.04	2	0.08	2
IL13	360	CHRNA6	ENSG00000147434.8	-0.58	0.78	0.04	2	-0.3	0.85
IL13	360	GINS4	ENSG00000147536.11	0.2	0.8	0.01	1	-0.03	0.97
IL13	360	ANKRD1	ENSG00000148677.6	-0.94	5.3E-09	-0.25	0.51	-0.97	7.3E-10
IL13	360	MKI67	ENSG00000148773.13	-0.24	0.91	0.58	0.3	0.29	0.66
IL13	360	INA	ENSG00000148798.10	-0.09	0.99	-0.12	0.94	0.56	0.19
IL13	360	NOCT	ENSG00000151014.5	0.52	0.12	0.74	0.0062	1.44	6.3E-12
IL13	360	CCDC3	ENSG00000151468.10	0.84	0.45	-0.58	0.71	0.13	0.94
IL13	360	ADAM8	ENSG00000151651.15	1.13	2.4E-14	1.32	3.8E-19	2.13	2.4E-52
IL13	360	SPC25	ENSG00000152253.8	-0.6	0.45	0.28	0.86	-0.5	0.42
IL13	360	THY1	ENSG00000154096.13	0.5	0.0013	0.55	0.00032	1.64	8.2E-41
IL13	360	ROBO4	ENSG00000154133.14	-0.62	0.097	0.87	0.0012	0.13	0.87
IL13	360	NRGN	ENSG00000154146.12	1.03	0.0094	0.47	0.57	0.89	0.023
IL13	360	CXADR	ENSG00000154639.18	-0.26	0.64	0.41	0.18	-0.01	0.99
IL13	360	KLF10	ENSG00000155090.14	-0.28	0.049	-0.12	0.71	-0.48	0.0000038
IL13	360	PPARGC1B	ENSG00000155846.16	0.39	0.72	-0.06	0.98	0.52	0.31
IL13	360	SH3RF2	ENSG00000156463.17	0.81	6.6E-07	-0.43	0.064	0.65	0.000097
IL13	360	NRG1	ENSG00000157168.18	0.21	0.9	0.42	0.48	0.89	0.0017
IL13	360	IL34	ENSG00000157368.10	0.06	0.99	1.19	1E-07	1.49	3.7E-13
IL13	360	GALNT14	ENSG00000158089.14	-0.27	0.96	-0.36	0.88	-0.52	0.62
IL13	360	XDH	ENSG00000158125.9	0.09	0.98	0.26	0.75	0.04	0.96
IL13	360	RHBDL2	ENSG00000158315.10	0.14	0.98	0.84	0.14	1.28	0.0011
IL13	360	CDC25C	ENSG00000158402.18	-0.2	0.96	0.48	0.57	0.42	0.5
IL13	360	ADAMTS4	ENSG00000158859.9	0.31	0.68	0.93	0.0006	0.85	0.0011
IL13	360	STC1	ENSG00000159167.11	-1.32	1.2E-14	0.27	0.52	-1.34	3.3E-15
IL13	360	ACTC1	ENSG00000159251.6	-0.12	0.97	0.53	0.55	-0.08	0.89
IL13	360	PTGIR	ENSG00000160013.8	-0.02	1	0.3	0.2	0.39	0.017
IL13	360	CCDC28B	ENSG00000160050.14	0.21	0.92	0.29	0.77	0.68	0.054
IL13	360	CILP2	ENSG00000160161.9	0.55	0.071	0.04	0.98	0.43	0.16
IL13	360	C21orf58	ENSG00000160298.17	0.22	0.81	0.01	1	-0.04	0.96
IL13	360	RECQL4	ENSG00000160957.12	0.07	0.98	0.06	0.96	0.19	0.62
IL13	360	PLXDC1	ENSG00000161381.13	0.22	0.97	0.34	0.87	0.81	0.23
IL13	360	SPC24	ENSG00000161888.11	0.15	0.97	-0.04	0.99	-0.12	0.89
IL13	360	IP6K3	ENSG00000161896.11	-0.17	0.81	0	1	0.39	0.04
IL13	360	CCNF	ENSG00000162063.12	-0.12	0.9	0.12	0.86	0.19	0.51
IL13	360	VANGL2	ENSG00000162738.5	-0.15	0.95	0.05	0.98	-0.01	0.99
IL13	360	CTSS	ENSG00000163131.10	0.25	0.75	1.21	1.9E-08	1.36	1.5E-11
IL13	360	CLDN1	ENSG00000163347.5	0.26	0.39	0.78	7E-07	1	4.5E-12
IL13	360	NUAK2	ENSG00000163545.8	-0.49	0.013	0.56	0.0019	-0.13	0.71
IL13	360	CADPS	ENSG00000163618.17	0.02	1	-1.22	2	-0.64	0.58

IL13	360	CXCL1	ENSG00000163739.4	0.85	0.0055	1.38	1.6E-07	2.14	3.3E-19
IL13	360	CDCP1	ENSG00000163814.7	0.38	0.51	0.54	0.17	0.62	0.037
IL13	360	HPGD	ENSG00000164120.13	0.12	2	0	2	0	2
IL13	360	HHIP	ENSG00000164161.9	-1.67	9.8E-34	0.22	0.54	-1.94	1.3E-45
IL13	360	EGFLAM	ENSG00000164318.17	1	0.014	-0.07	0.98	1.25	0.00027
IL13	360	MICALL2	ENSG00000164877.18	0.28	0.2	0.68	5E-07	0.98	1.2E-15
IL13	360	MAMDC2	ENSG00000165072.9	0.2	0.67	0.13	0.84	0.72	0.0000024
IL13	360	SKA3	ENSG00000165480.15	-0.33	0.86	0.3	0.84	0.33	0.67
IL13	360	ZNF219	ENSG00000165804.15	0.08	0.97	0.19	0.72	0.38	0.069
IL13	360	HSPA12A	ENSG00000165868.13	-0.11	0.95	0.25	0.58	0	1
IL13	360	E2F7	ENSG00000165891.15	-0.22	0.84	0.6	0.076	-0.13	0.85
IL13	360	DCHS1	ENSG00000166341.7	0.36	0.72	0.11	0.95	0.58	0.17
IL13	360	RRAD	ENSG00000166592.11	-0.14	0.92	0.82	0.0001	0.61	0.0048
IL13	360	PLK1	ENSG00000166851.14	-0.14	0.91	0.16	0.81	-0.07	0.9
IL13	360	TAC3	ENSG00000166863.11	0.06	1	1.14	2	2.2	0.0027
IL13	360	GPRC5B	ENSG00000167191.11	0.28	0.59	0.25	0.66	0.66	0.0023
IL13	360	CYP2S1	ENSG00000167600.13	-0.24	0.79	0.42	0.32	0.07	0.93
IL13	360	TK1	ENSG00000167900.11	-0.38	0.64	0.19	0.89	-0.16	0.84
IL13	360	PBK	ENSG00000168078.9	-0.17	0.96	0.48	0.47	0.19	0.82
IL13	360	TAP1	ENSG00000168394.10	-0.14	0.78	0.48	0.0021	0.4	0.0088
IL13	360	GDNF	ENSG00000168621.14	-1.16	5.2E-29	0.16	0.51	-1.55	1.8E-49
IL13	360	C8orf46	ENSG00000169085.11	-0.12	0.99	0.68	0.56	0.79	0.28
IL13	360	GPRIN1	ENSG00000169258.6	-0.44	0.0055	-0.03	0.97	-0.46	0.0018
IL13	360	COL22A1	ENSG00000169436.16	-0.26	0.91	0.42	0.82	0.19	0.92
IL13	360	BUB1	ENSG00000169679.14	-0.19	0.88	0.64	0.03	0.1	0.89
IL13	360	ROBO1	ENSG00000169855.19	0.21	0.89	1.03	0.00016	1.15	0.0000038
IL13	360	WNT10B	ENSG00000169884.13	0.26	0.95	0.04	0.99	0.06	0.97
IL13	360	TM4SF1	ENSG00000169908.11	0.25	0.42	-0.06	0.94	0.31	0.12
IL13	360	CST1	ENSG00000170373.8	-1.01	0.00029	0.66	0.056	-0.5	0.15
IL13	360	MTSS1	ENSG00000170873.18	-0.74	0.00044	0.38	0.25	-0.27	0.39
IL13	360	SHCBP1	ENSG00000171241.8	-0.24	0.87	0.62	0.12	0.13	0.87
IL13	360	ESCO2	ENSG00000171320.14	0.12	0.99	0.96	0.11	0.93	0.068
IL13	360	ENC1	ENSG00000171617.13	-0.06	0.97	-0.1	0.81	-0.22	0.15
IL13	360	RRM2	ENSG00000171848.14	-0.44	0.42	0.26	0.77	0.08	0.93
IL13	360	FRMD5	ENSG00000171877.20	-0.17	0.85	-0.03	0.98	-0.29	0.3
IL13	360	GAP43	ENSG00000172020.12	0.24	0.98	0.48	2	1.3	0.12
IL13	360	MYEOV	ENSG00000172927.7	-0.06	1	0.3	0.72	-0.4	0.38
IL13	360	SLCO2A1	ENSG00000174640.12	1.11	1.7E-08	1.05	2.2E-07	2.28	9.9E-38
IL13	360	UBE2C	ENSG00000175063.16	-0.48	0.28	0.3	0.66	-0.36	0.38
IL13	360	FOSL1	ENSG00000175592.8	0.42	0.051	0.77	4.6E-06	0.89	0.000000012
IL13	360	SPHK1	ENSG00000176170.13	-0.1	0.98	0.51	0.28	0.27	0.61
IL13	360	TYMS	ENSG00000176890.15	0.17	0.59	0.14	0.68	0.35	0.01
IL13	360	SHMT1	ENSG00000176974.19	0.24	0.8	-0.04	0.98	-0.33	0.39
IL13	360	HASPIN	ENSG00000177602.5	0.18	0.96	0.23	0.87	0.06	0.96
IL13	360	ADAMTSL1	ENSG00000178031.16	0.04	1	0.32	0.51	0.39	0.2
IL13	360	STAP2	ENSG00000178078.11	-0.16	0.96	0.57	0.27	0.48	0.29
IL13	360	AURKB	ENSG00000178999.12	-0.23	0.89	-0.06	0.97	-0.16	0.82
IL13	360	TMTC2	ENSG00000179104.8	-0.31	0.47	0.07	0.95	-0.14	0.78
IL13	360	APOBEC3B	ENSG00000179750.15	-0.42	0.75	0.56	0.51	0.54	0.38
IL13	360	AKAP5	ENSG00000179841.8	-0.31	0.7	-0.23	0.81	-0.67	0.031
IL13	360	FDCSP	ENSG00000181617.5	0.08	1	1.12	0.18	1.26	0.055
IL13	360	GPR3	ENSG00000181773.6	-0.08	0.99	0.19	0.9	0.38	0.46
IL13	360	CHST15	ENSG00000182022.17	-0.02	1	0.33	0.59	0.51	0.11
IL13	360	CAPN12	ENSG00000182472.8	0.7	0.13	1.17	0.00053	1.55	0.00000011
IL13	360	GJC1	ENSG00000182963.9	-0.03	0.99	0.57	9.5E-11	0.34	0.00028
IL13	360	OPCML	ENSG00000183715.13	1.87	0.000094	0.16	0.95	1.68	0.00039
IL13	360	GPR39	ENSG00000183840.6	0.04	1	0.46	0.16	0.61	0.0089
IL13	360	IQGAP3	ENSG00000183856.10	-0.26	0.88	0.32	0.74	-0.17	0.83
IL13	360	ANO9	ENSG00000185101.12	0.2	0.97	1.01	0.04	1.94	0.00000006
IL13	360	GP1BA	ENSG00000185245.7	-0.04	0.99	1.31	0.026	2.49	0.00000037
IL13	360	TNFAIP8L1	ENSG00000185361.8	-0.31	0.49	0.2	0.77	0.09	0.88
IL13	360	MYBL1	ENSG00000185697.16	0.51	0.42	1.91	8.3E-11	1.78	5.5E-10
IL13	360	KIF18B	ENSG00000186185.13	-0.55	0.34	0.33	0.71	-0.16	0.85
IL13	360	KIF24	ENSG00000186638.16	-0.01	1	0.47	0.42	0.26	0.68
IL13	360	ERCC6L	ENSG00000186871.6	0.24	0.96	1.08	0.062	0.39	0.66

IL13	360	EMID1	ENSG00000186998.15	-0.3	0.56	0.29	0.58	0.05	0.94
IL13	360	FANCA	ENSG00000187741.14	0.18	0.92	0.33	0.57	0.37	0.32
IL13	360	S100A3	ENSG00000188015.9	0.16	0.98	0.32	0.88	0.89	0.15
IL13	360	WNT7B	ENSG00000188064.9	-0.87	0.026	0.78	0.045	-0.29	0.64
IL13	360	CLDN4	ENSG00000189143.9	-0.06	1	0.39	0.75	0.17	0.88
IL13	360	SH2D5	ENSG00000189410.11	-0.9	1.5E-08	0.29	0.28	-0.51	0.0029
IL13	360	RFX8	ENSG00000196460.12	0.86	5.4E-07	0.41	0.1	0.91	0.000000048
IL13	360	SIGLEC15	ENSG00000197046.11	-0.04	1	1	2.5E-07	0.47	0.053
IL13	360	ACSL5	ENSG00000197142.10	-0.01	1	0.75	0.00019	0.58	0.0051
IL13	360	C6orf141	ENSG00000197261.11	1.12	5.6E-11	-0.16	0.84	0.57	0.0044
IL13	360	BLM	ENSG00000197299.10	0.06	1	0.78	0.095	0.13	0.9
IL13	360	MAP3K5	ENSG00000197442.9	-0.16	0.91	0.79	0.0014	0.69	0.0038
IL13	360	STMN3	ENSG00000197457.9	0.12	0.96	-0.31	0.58	0.07	0.92
IL13	360	SLC28A3	ENSG00000197506.7	-0.26	0.86	1.4	2.3E-07	1.1	0.000081
IL13	360	ELOVL2	ENSG00000197977.3	1.52	0.048	0.66	2	2.42	0.0002
IL13	360	PNP	ENSG00000198805.11	0.1	0.94	0.31	0.2	0.26	0.23
IL13	360	L1CAM	ENSG00000198910.12	0	1	0.85	5.1E-06	0.91	0.0000002
IL13	360	SLC44A4	ENSG00000204385.10	-0.06	2	0.34	2	0.19	2
IL13	360	FAM196B	ENSG00000204767.3	0.66	0.37	-0.19	0.92	0.23	0.82
IL13	360	ADGRG1	ENSG00000205336.11	-0.96	0.000048	0.24	0.72	-0.95	0.000033
IL13	360	EXOC3L4	ENSG00000205436.7	-0.08	0.98	0.05	0.99	0.23	0.82
IL13	360	HLA-H	ENSG00000206341.7	-0.02	1	0.19	0.62	0.22	0.34
IL13	360	TMEM200C	ENSG00000206432.4	0.09	2	0.16	2	0.3	2
IL13	360	TRBC2	ENSG00000211772.11	-0.51	0.83	-0.26	2	-0.25	0.87
IL13	360	ZNF90	ENSG00000213988.10	0.26	0.94	0.01	1	-0.35	0.69
IL13	360	AC098934.1	ENSG00000214796.8	-0.19	0.97	-0.17	0.94	-0.11	0.94
IL13	360	DDX12P	ENSG00000214826.5	-0.18	0.98	-0.13	0.96	0.18	0.89
IL13	360	AL390719.1	ENSG00000217801.9	-0.05	1	1	8.8E-06	0.74	0.0014
IL13	360	AL354740.1	ENSG00000225339.3	-0.11	0.97	0.39	0.27	0.23	0.55
IL13	360	LINC00511	ENSG00000227036.6	-0.27	0.96	0.33	0.87	0.17	0.9
IL13	360	AL139220.2	ENSG00000230615.6	0.44	0.62	-0.13	0.94	0.32	0.65
IL13	360	HLA-B	ENSG00000234745.10	-0.01	1	0.31	0.41	0.35	0.16
IL13	360	NFAM1	ENSG00000235568.6	-0.25	0.91	0.85	0.038	1.19	0.00014
IL13	360	HMGA1P1	ENSG00000236683.3	-0.05	1	0.19	0.92	-0.52	0.4
IL13	360	KIFC1	ENSG00000237649.7	-0.33	0.48	0.4	0.27	0	1
IL13	360	AC116347.1	ENSG00000238000.1	0.04	1	0.24	0.86	0.71	0.067
IL13	360	PSMB9	ENSG00000240065.7	0.24	0.85	0.8	0.013	1.01	0.00018
IL13	360	LINC00973	ENSG00000240476.1	-0.39	0.31	1.04	3.2E-07	0.27	0.46
IL13	360	RPLP0P2	ENSG00000243742.5	0.3	0.86	1.01	0.0084	1.29	0.000057
IL13	360	AC097451.1	ENSG00000250657.1	-0.38	0.86	0.85	0.23	-0.37	0.69
IL13	360	LINC01605	ENSG00000253161.5	-0.31	0.11	0.13	0.78	0.09	0.8
IL13	360	AC107959.3	ENSG00000253616.5	-0.62	0.65	0.15	0.96	0.28	0.83
IL13	360	AC090197.1	ENSG00000253837.1	0.28	0.88	1.06	0.0039	0.52	0.3
IL13	360	AC112777.1	ENSG00000256663.1	0.18	0.98	1.01	0.13	1.03	0.063
IL13	360	TMPO-AS1	ENSG00000257167.2	-0.6	0.28	0.15	0.93	-0.1	0.91
IL13	360	LINC02407	ENSG00000257219.5	0.22	0.7	0.25	0.58	0.44	0.042
IL13	360	AC004264.1	ENSG00000268812.3	-0.43	0.85	0.33	0.88	-0.41	0.68
IL13	360	AC009549.1	ENSG00000270607.1	-0.24	0.68	1.06	1E-09	0.45	0.041
IL13	360	AC012073.1	ENSG00000271936.1	0.33	0.91	0	1	0.23	0.85
IL13	360	AL138724.1	ENSG00000272269.1	-0.06	0.99	0.74	0.002	0.54	0.034
IL13	360	EPOP	ENSG00000273604.1	0.12	0.97	0.17	0.88	0.46	0.16
IL13	360	AL035461.2	ENSG00000275632.1	0.03	1	0.33	2	0.14	0.94
IL13	360	AC020763.4	ENSG00000279569.1	0.18	0.98	0.88	0.22	0.4	0.67
IL13	360	HELLPAR	ENSG00000281344.1	1.17	0.13	0.19	0.94	0.79	0.32
IL13	360	SNHG4	ENSG00000281398.2	0.1	0.97	0.05	0.97	0.26	0.42
IL13	3	ANOS1	ENSG00000111728.10	4.21	4.1E-126	0.63	0.032	4.39	4.3E-137
IL13	3	AC009533.1	ENSG00000111788.10	0.18	0.92	-0.14	0.92	0.04	0.96
IL13	3	RGS4	ENSG00000117152.13	-0.63	0.0022	0.12	0.89	-0.55	0.0062
IL17	1	ENSG0000012573	C3	0.91	2.3	3.95	3.00E-10	3.10E-68	2.40E-209
IL17	355	ENSG0000000293	TMEM176A	0.73	0.64	0.76	0.013	2	0.0055
IL17	355	ENSG0000000398	MTMR7	0.8	-0.15	1.27	0.0095	2	1.30E-05
IL17	355	ENSG0000000398	SLC7A2	0.6	-0.02	2.18	1.00E-18	0.85	2.10E-250
IL17	355	ENSG0000000606	MAP3K14	0.81	0.21	1.15	6.20E-30	0.033	1.80E-63
IL17	355	ENSG0000000611	TMEM132A	0.93	0.13	1.72	2.90E-44	0.17	2.20E-155
IL17	355	ENSG0000000621	CX3CL1	1.47	0.19	1.43	2.70E-08	2	3.10E-08

IL17	355	ENSG0000000628	CACNA1G	0.9	0.19	1.58	3.00E-05	2	1.10E-15
IL17	355	ENSG0000000643	MAP3K9	1.08	0.19	1.03	1.10E-04	2	1.30E-04
IL17	355	ENSG0000000796	E2F2	0.96	0.41	1.43	3.40E-08	2	1.10E-18
IL17	355	ENSG0000000832	PLEKHG6	0.66	-0.19	0.63	0.036	2	0.034
IL17	355	ENSG0000000851	IL32	1.4	-0.41	2.29	3.70E-35	0.01	3.60E-98
IL17	355	ENSG0000001003	ETV7	1.14	0.27	2.01	1.20E-05	2	5.90E-13
IL17	355	ENSG0000001134	SYT7	1.13	0.1	0.68	5.50E-36	0.48	4.80E-13
IL17	355	ENSG0000001142	ANLN	0.63	0.11	1.2	1.00E-10	0.42	7.90E-39
IL17	355	ENSG0000001381	TACC3	0.63	0.23	0.73	8.40E-15	0.028	2.30E-20
IL17	355	ENSG0000001513	CCDC88C	1.16	0.28	1.74	9.60E-08	2	3.20E-17
IL17	355	ENSG0000001958	CD74	1.62	0.19	1.58	1.60E-65	0.19	4.70E-63
IL17	355	ENSG0000002164	NRXN3	0.76	0.14	1.07	1.30E-04	2	4.40E-09
IL17	355	ENSG0000002344	BIRC3	1.94	-0.18	4.51	5.40E-23	0.51	3.80E-118
IL17	355	ENSG0000002570	TYMP	0.77	0.11	2.23	7.60E-08	0.6	7.00E-69
IL17	355	ENSG0000002577	TOMM34	0.6	0.04	1.07	1.90E-15	0.76	1.50E-49
IL17	355	ENSG0000002813	TNFRSF1B	0.85	0.15	1.7	7.40E-36	0.11	3.20E-152
IL17	355	ENSG0000002827	POU2F2	1.18	0.6	1.9	1.30E-16	2	4.70E-45
IL17	355	ENSG0000002915	ARNTL2	0.92	-0.01	2.27	2.10E-35	0.97	4.70E-227
IL17	355	ENSG0000004206	RIPOR3	0.88	0.52	1.57	2.60E-31	5.80E-09	1.40E-106
IL17	355	ENSG0000004452	EPHA3	1.07	-0.02	1.19	1.20E-19	0.91	4.40E-25
IL17	355	ENSG0000004805	HDAC9	0.79	-0.34	1.07	3.20E-06	2	1.30E-11
IL17	355	ENSG0000005034	NFE2L3	1.48	0.05	2.02	8.70E-143	0.57	2.80E-277
IL17	355	ENSG0000005370	NRIP2	0.92	-0.25	1.14	6.90E-04	2	7.30E-06
IL17	355	ENSG0000005655	TRAF1	1.09	0.03	3.34	4.10E-20	0.87	5.50E-193
IL17	355	ENSG0000005701	DCBLD2	0.74	-0.05	1.99	4.30E-40	0.54	6.30E-291
IL17	355	ENSG0000005808	LAMC2	0.79	0.21	2.01	2.40E-07	2	1.90E-48
IL17	355	ENSG0000006133	LZTS1	0.73	-0.09	0.65	0.0041	2	0.0086
IL17	355	ENSG0000006228	DGAT2	0.88	-0.15	1.82	1.50E-04	2	9.70E-18
IL17	355	ENSG0000006282	POLD1	0.59	0.27	0.9	1.50E-11	0.017	2.80E-27
IL17	355	ENSG0000006430	CDON	0.63	0.05	0.82	8.60E-09	0.77	4.50E-15
IL17	355	ENSG0000006488	CHI3L2	0.63	0.57	1.83	0.033	2	5.30E-10
IL17	355	ENSG0000006532	MCM10	0.62	0.22	1.56	2.70E-09	0.11	1.80E-62
IL17	355	ENSG0000006627	ASPM	0.62	0.07	0.83	2.40E-06	0.73	2.30E-11
IL17	355	ENSG0000006722	PKM	0.69	0.11	0.98	1.40E-43	0.1	4.20E-88
IL17	355	ENSG0000006948	GAL	1.47	0.04	1.38	2.90E-47	0.86	1.20E-41
IL17	355	ENSG0000007153	TRIP13	0.63	0.26	1.1	1.00E-12	0.024	3.80E-40
IL17	355	ENSG0000007328	TP63	0.7	0.08	1.42	0.019	2	6.90E-05
IL17	355	ENSG0000007431	SNCB	0.97	0.23	0.67	0.001	2	0.022
IL17	355	ENSG0000007521	GTSE1	0.73	0.24	0.78	6.00E-15	0.045	7.00E-18
IL17	355	ENSG0000007570	WDR62	0.86	0.28	1.31	1.50E-18	0.031	1.60E-45
IL17	355	ENSG0000007638	SPAG5	0.76	0.34	0.96	4.90E-19	0.0023	1.90E-31
IL17	355	ENSG0000007715	NFKB2	1.06	0.03	2.29	1.70E-54	0.81	2.30E-263
IL17	355	ENSG0000007826	SYNJ2	0.75	0.07	1.04	1.60E-61	0.25	5.00E-121
IL17	355	ENSG0000008511	CD82	0.92	-0.08	1.39	2.80E-40	0.44	8.80E-94
IL17	355	ENSG0000008599	RAD54L	0.69	0.37	1.31	5.50E-08	0.019	2.10E-29
IL17	355	ENSG0000008708	ACHE	0.92	-0.12	2.27	2.20E-05	2	6.70E-31
IL17	355	ENSG0000008832	TPX2	0.64	0.25	0.66	2.60E-09	0.07	3.40E-10
IL17	355	ENSG0000008968	BIRC5	0.62	0.16	0.68	7.50E-13	0.17	5.20E-16
IL17	355	ENSG0000009010	RGS1	0.68	0.13	1.83	0.024	2	2.10E-07
IL17	355	ENSG0000009088	KIF4A	0.59	0.18	0.72	1.00E-09	0.14	9.40E-15
IL17	355	ENSG0000009300	CDC45	0.77	0.3	1.37	6.70E-12	0.04	1.80E-38
IL17	355	ENSG0000009530	PTGS1	0.92	0.16	1.16	1.10E-93	0.01	1.10E-153
IL17	355	ENSG0000009995	DERL3	0.82	0.39	0.94	0.0026	2	2.20E-04
IL17	355	ENSG0000009999	GGT5	0.88	0.08	1.31	1.20E-05	2	3.10E-12
IL17	355	ENSG0000010007	PLA2G3	0.73	0.08	1.26	0.0018	2	2.10E-09
IL17	355	ENSG0000010016	CENPM	0.67	0.38	0.94	2.10E-08	0.013	5.80E-17
IL17	355	ENSG0000010076	PAPLN	1.34	0.14	1.2	8.30E-118	0.081	2.70E-95
IL17	355	ENSG0000010090	NFKBIA	0.8	0.07	1.72	4.20E-30	0.52	4.50E-145
IL17	355	ENSG0000010098	MMP9	1.74	-0.17	2.27	2.70E-07	2	3.20E-10
IL17	355	ENSG0000010105	MYBL2	0.69	0.3	1.14	8.90E-17	0.006	1.40E-46
IL17	355	ENSG0000010118	SLCO4A1	0.63	0.27	1.56	0.0093	2	5.30E-14
IL17	355	ENSG0000010118	NTSR1	1.49	0.28	1.6	2.20E-46	0.059	4.30E-54
IL17	355	ENSG0000010144	FAM83D	0.72	0.42	0.69	5.80E-11	0.0029	9.70E-11
IL17	355	ENSG0000010167	LIPG	1.07	0.29	1.8	5.60E-12	0.16	1.70E-34
IL17	355	ENSG0000010226	TIMP1	0.73	0.03	0.61	2.60E-39	0.69	2.60E-28

IL17	355	ENSG0000010227	KLHL4	1.25	0.11	1.03	5.70E-05	2	5.10E-04
IL17	355	ENSG0000010246	HTR2A	1.19	0.11	1.64	5.90E-05	2	3.80E-08
IL17	355	ENSG0000010374	IGDCC4	0.7	0.07	0.86	3.00E-47	0.3	1.00E-72
IL17	355	ENSG0000010381	CTSH	0.67	0.35	0.64	0.0025	2	0.003
IL17	355	ENSG0000010441	WISP1	0.86	-0.11	1.37	3.90E-07	2	2.30E-18
IL17	355	ENSG0000010478	KCNN4	1.05	-0.15	1.18	2.50E-06	2	3.90E-08
IL17	355	ENSG0000010485	RELB	1	-0.02	1.87	3.20E-39	0.91	3.80E-144
IL17	355	ENSG0000010495	IL4I1	1.59	-0.02	2.56	7.70E-14	2	5.70E-35
IL17	355	ENSG0000010499	IL27RA	1.63	0.02	2.63	2.10E-43	0.94	5.00E-119
IL17	355	ENSG0000010501	ASF1B	0.67	0.27	1	4.20E-12	0.033	3.10E-27
IL17	355	ENSG0000010524	EBI3	0.72	-0.1	1.01	0.021	2	4.80E-04
IL17	355	ENSG0000010548	LIG1	0.68	0.28	0.97	2.00E-22	0.0023	2.40E-47
IL17	355	ENSG0000010549	PLA2G4C	1.84	0.04	2.55	1.10E-45	0.9	1.20E-91
IL17	355	ENSG0000010576	CADM4	0.89	0.04	0.69	5.80E-17	0.85	9.50E-11
IL17	355	ENSG0000010587	DNAH11	1.13	-0.31	0.99	8.30E-05	2	4.00E-04
IL17	355	ENSG0000010656	TMEM176B	0.84	0.53	0.69	8.70E-04	2	0.0049
IL17	355	ENSG0000010798	DKK1	0.76	0.05	1.01	5.90E-18	0.72	3.70E-32
IL17	355	ENSG0000010855	RASD1	1.01	0.1	0.7	3.50E-10	2	1.60E-05
IL17	355	ENSG0000010869	CCL2	0.63	0.09	1.68	0.016	2	1.40E-13
IL17	355	ENSG0000010884	ABCC3	0.76	0.04	1.32	1.60E-28	0.74	5.20E-91
IL17	355	ENSG0000011040	NECTIN1	0.7	0.23	0.88	4.80E-23	0.013	3.00E-38
IL17	355	ENSG0000011042	KIAA1549L	0.66	0.03	1.45	2.50E-04	2	7.70E-20
IL17	355	ENSG0000011066	SLC35F2	0.86	0.13	1.56	2.40E-22	0.29	4.30E-79
IL17	355	ENSG0000011120	FOXM1	0.62	0.21	0.63	3.20E-15	0.038	6.90E-16
IL17	355	ENSG0000011124	RAD51AP1	0.64	0.21	1.16	4.70E-08	0.17	6.00E-27
IL17	355	ENSG0000011133	OAS3	0.84	0.26	1.09	9.00E-23	0.023	5.70E-40
IL17	355	ENSG0000011166	CDCA3	0.65	0.23	0.64	1.60E-09	0.099	1.40E-09
IL17	355	ENSG0000011172	ST8SIA1	0.82	0.04	0.91	5.10E-06	2	1.30E-07
IL17	355	ENSG0000011178	AC009533.1	0.62	0.44	0.88	0.0095	2	4.10E-05
IL17	355	ENSG0000011229	CRYBG1	1.13	0.09	1.69	4.50E-39	0.49	4.60E-91
IL17	355	ENSG0000011274	TTK	0.67	0.2	0.93	2.50E-08	0.2	2.00E-16
IL17	355	ENSG0000011277	FAM46A	0.62	-0.16	0.67	2.10E-13	0.15	5.00E-16
IL17	355	ENSG0000011307	HBEGF	1.22	0.32	2.77	1.30E-20	0.077	2.40E-114
IL17	355	ENSG0000011336	LMNB1	0.63	0.18	0.93	7.30E-24	0.027	3.80E-54
IL17	355	ENSG0000011364	WWC1	0.79	0.41	2.3	8.20E-10	0.012	1.60E-90
IL17	355	ENSG0000011365	DPYSL3	0.78	0.07	0.81	2.40E-103	0.13	1.10E-113
IL17	355	ENSG0000011372	CDX1	0.87	0	2.45	1.10E-04	2	1.50E-33
IL17	355	ENSG0000011452	C3orf52	0.81	0.35	1.73	2.50E-06	2	4.80E-29
IL17	355	ENSG0000011526	IFIH1	0.65	-0.08	1.24	4.90E-06	0.69	5.30E-22
IL17	355	ENSG0000011706	ST6GALNAC5	1	-0.04	2.23	1.40E-08	2	3.50E-44
IL17	355	ENSG0000011715	RGS4	0.84	0.04	1.31	9.90E-13	0.85	9.80E-32
IL17	355	ENSG0000011739	CDC20	0.7	0.27	0.63	1.20E-13	0.027	1.40E-11
IL17	355	ENSG0000011746	PIK3R3	0.83	0.08	0.7	2.30E-20	0.55	9.20E-15
IL17	355	ENSG0000011758	TNFSF4	0.94	0.02	2	1.00E-39	0.87	1.20E-189
IL17	355	ENSG0000011767	RPS6KA1	0.9	0.29	1.2	2.90E-06	2	3.90E-11
IL17	355	ENSG0000011772	CENPF	0.6	-0.01	0.83	3.40E-06	0.97	1.80E-11
IL17	355	ENSG0000011850	TNFAIP3	0.78	0.01	1.74	1.60E-18	0.94	1.10E-97
IL17	355	ENSG0000012016	MOB3B	1.47	-0.13	3.61	2.40E-16	2	1.40E-98
IL17	355	ENSG0000012033	TNFSF18	0.74	-0.27	2.14	1.20E-04	2	1.80E-36
IL17	355	ENSG0000012054	KIAA1217	1	0.01	0.84	9.30E-21	0.97	4.90E-15
IL17	355	ENSG0000012065	TNFSF11	0.99	-0.02	1.23	0.0026	2	2.80E-04
IL17	355	ENSG0000012083	SOCS2	0.6	-0.13	0.99	2.00E-06	0.44	1.80E-17
IL17	355	ENSG0000012115	NCAPH	0.77	0.35	1.11	5.30E-11	0.021	3.50E-23
IL17	355	ENSG0000012121	MND1	0.63	-0.12	0.99	4.00E-04	2	8.30E-10
IL17	355	ENSG0000012287	EGR2	0.98	0.19	1.28	6.40E-07	2	5.70E-12
IL17	355	ENSG0000012295	ZWINT	0.69	0.29	1.26	6.10E-13	0.02	6.80E-45
IL17	355	ENSG0000012309	BHLHE41	1.21	0.02	1.76	1.00E-07	2	5.10E-16
IL17	355	ENSG0000012348	HJURP	0.72	0.27	0.95	5.80E-19	0.011	9.80E-34
IL17	355	ENSG0000012397	CKS2	0.67	0.17	0.67	2.60E-12	0.19	5.70E-13
IL17	355	ENSG0000012487	CXCL6	0.62	0.93	3.44	0.0015	2	7.40E-101
IL17	355	ENSG0000012531	C17orf53	0.7	0.21	1.09	1.50E-06	2	5.60E-16
IL17	355	ENSG0000012534	IRF1	0.64	-0.02	0.91	5.60E-18	0.87	1.30E-37
IL17	355	ENSG0000012584	BMP2	0.87	0.12	1.56	7.10E-08	2	1.50E-25
IL17	355	ENSG0000012596	GDF5	0.59	0.43	0.72	1.70E-25	6.10E-12	1.10E-38
IL17	355	ENSG0000012719	TRAF2	0.73	0.06	1.31	1.70E-14	0.67	1.90E-48

IL17	355	ENSG0000012758	CHTF18	0.67	0.35	0.98	1.50E-17	6.50E-04	3.30E-39
IL17	355	ENSG0000012826	POM121L9P	0.78	0.14	1.25	0.012	2	1.40E-05
IL17	355	ENSG0000012840	RIBC2	0.83	0.32	0.92	0.002	2	3.20E-04
IL17	355	ENSG0000012857	STRIP2	0.89	0.31	1.56	2.20E-07	2	5.30E-23
IL17	355	ENSG0000012880	ARHGAP22	0.73	0.14	1.18	5.90E-21	0.18	3.70E-56
IL17	355	ENSG0000012891	DLL4	0.71	0.35	3.5	0.0075	2	2.40E-48
IL17	355	ENSG0000012917	E2F8	0.76	0.24	1.11	2.50E-06	2	8.60E-14
IL17	355	ENSG0000012919	PIMREG	0.72	0.35	0.63	2.10E-10	0.015	1.30E-08
IL17	355	ENSG0000012966	RHBDF2	1.37	0.09	1.74	1.30E-53	0.51	1.70E-89
IL17	355	ENSG0000012981	SGO1	0.61	0.25	1.08	2.40E-05	2	2.20E-16
IL17	355	ENSG0000013003	CRACR2A	0.63	0.12	1.05	8.80E-04	2	4.80E-10
IL17	355	ENSG0000013103	EPS8L1	0.99	-0.04	1.02	1.30E-04	2	3.90E-05
IL17	355	ENSG0000013132	TRAF3	0.91	0.18	1.4	3.80E-61	0.019	4.30E-152
IL17	355	ENSG0000013200	ZSWIM4	0.77	0.23	1.44	4.50E-19	0.049	1.40E-70
IL17	355	ENSG0000013248	TRIM47	1.29	0.15	1.91	2.50E-57	0.21	9.40E-130
IL17	355	ENSG0000013274	TESMIN	1.05	0.43	0.68	5.00E-04	2	0.019
IL17	355	ENSG0000013304	CHI3L1	0.59	-0.03	1.74	3.00E-04	0.91	6.00E-34
IL17	355	ENSG0000013308	DCLK1	1.8	0.19	1.92	2.80E-14	2	1.80E-16
IL17	355	ENSG0000013311	RFC3	0.63	0.24	1.33	1.40E-09	0.075	7.80E-44
IL17	355	ENSG0000013321	EPHB2	0.6	0.18	0.59	3.60E-40	0.0025	1.30E-40
IL17	355	ENSG0000013380	MICALCL	0.69	0.19	1.19	0.028	2	2.10E-05
IL17	355	ENSG0000013381	MICAL2	0.86	0.13	1.13	3.70E-69	0.044	4.50E-122
IL17	355	ENSG0000013398	TTC9	1.79	-0.14	1.97	4.20E-55	0.43	5.00E-68
IL17	355	ENSG0000013405	CCNB1	0.61	0.15	0.61	6.90E-13	0.17	2.10E-13
IL17	355	ENSG0000013410	BHLHE40	0.62	0.03	1.02	4.40E-18	0.78	7.10E-50
IL17	355	ENSG0000013447	IL15RA	0.75	0.02	1.79	1.10E-10	0.94	2.20E-66
IL17	355	ENSG0000013451	DOCK2	0.73	-0.09	1.12	1.10E-05	2	4.10E-13
IL17	355	ENSG0000013453	RERG	0.69	-0.21	0.62	6.30E-06	2	3.90E-05
IL17	355	ENSG0000013469	CDCA8	0.71	0.23	0.69	5.60E-16	0.042	1.30E-15
IL17	355	ENSG0000013531	NT5E	0.81	0.05	0.95	5.00E-45	0.59	3.40E-62
IL17	355	ENSG0000013547	ESPL1	0.68	0.33	0.7	1.00E-09	0.021	9.00E-11
IL17	355	ENSG0000013554	PKIB	0.66	0.18	0.95	1.10E-22	0.046	1.20E-47
IL17	355	ENSG0000013567	CPM	1.35	0.04	1.26	2.60E-24	2	9.60E-22
IL17	355	ENSG0000013620	TNS3	0.8	-0.03	0.94	3.10E-70	0.68	1.40E-96
IL17	355	ENSG0000013627	C7orf69	1.07	0.03	1.03	0.0015	2	0.0013
IL17	355	ENSG0000013636	ZFHX2	1.05	0.16	1.84	5.70E-06	2	2.50E-17
IL17	355	ENSG0000013649	LIMD2	0.7	0.11	0.81	1.00E-16	0.36	6.40E-23
IL17	355	ENSG0000013703	IL33	1.48	0.32	3.13	1.30E-10	2	2.90E-41
IL17	355	ENSG0000013730	HMGA1	0.96	0.04	0.94	2.90E-76	0.64	4.10E-74
IL17	355	ENSG0000013746	TLR2	0.83	-0.22	1.24	7.90E-09	2	5.00E-20
IL17	355	ENSG0000013809	CENPO	0.65	0.32	0.74	4.30E-21	2.90E-04	1.80E-28
IL17	355	ENSG0000013813	LOXL4	0.84	0.25	1.18	9.20E-52	7.10E-04	1.10E-103
IL17	355	ENSG0000013834	MYPN	0.73	-0.05	0.84	0.0091	2	0.0012
IL17	355	ENSG0000013877	CENPE	0.61	0.07	0.77	2.40E-07	0.69	5.40E-12
IL17	355	ENSG0000013992	FRMD6	0.66	-0.07	1.34	5.70E-33	0.4	6.90E-136
IL17	355	ENSG0000014053	TICRR	0.65	0.38	0.96	1.90E-11	0.0021	4.90E-26
IL17	355	ENSG0000014053	NTRK3	3.26	0.13	2.26	4.30E-12	2	1.40E-08
IL17	355	ENSG0000014138	TAF4B	0.8	0.2	1.78	7.60E-05	2	6.40E-23
IL17	355	ENSG0000014146	SLC14A1	1.57	0.23	0.81	4.20E-56	0.11	1.40E-14
IL17	355	ENSG0000014262	EPHA2	0.87	0.22	1.25	1.30E-59	0.0032	2.30E-128
IL17	355	ENSG0000014273	PLK4	0.65	0.14	1.24	4.70E-07	0.41	7.80E-26
IL17	355	ENSG0000014294	KIF2C	0.63	0.24	0.67	1.60E-12	0.034	1.10E-14
IL17	355	ENSG0000014322	NUF2	0.66	0.07	1.13	5.10E-09	0.68	2.90E-27
IL17	355	ENSG0000014338	CTSK	0.9	0.14	0.6	9.30E-84	0.034	1.30E-37
IL17	355	ENSG0000014349	VASH2	1.08	-0.02	1.49	3.70E-33	0.88	1.10E-64
IL17	355	ENSG0000014378	CNIH3	0.86	0.26	0.96	4.40E-17	0.058	7.90E-22
IL17	355	ENSG0000014455	FANCD2	0.69	0.21	1.21	6.50E-15	0.074	6.00E-49
IL17	355	ENSG0000014474	LRIG1	0.73	0.13	1.06	2.90E-76	0.015	5.50E-165
IL17	355	ENSG0000014495	NCEH1	0.63	0.14	1.69	8.00E-08	0.4	6.20E-59
IL17	355	ENSG0000014536	ANK2	1	-0.04	1.69	2.80E-57	0.7	4.20E-172
IL17	355	ENSG0000014623	NFKBIE	1.35	0.01	2.2	6.30E-61	0.95	3.90E-173
IL17	355	ENSG0000014641	MTFR2	0.67	0.23	1.08	5.20E-04	2	6.00E-10
IL17	355	ENSG0000014667	CDCA5	0.73	0.33	1.13	6.20E-17	0.0038	2.40E-41
IL17	355	ENSG0000014743	CHRNA6	0.76	-0.16	1.24	0.013	2	1.60E-04
IL17	355	ENSG0000014753	GINS4	1.2	0.38	0.96	2.10E-51	4.60E-04	2.90E-33

IL17	355	ENSG0000014867	ANKRD1	0.83	0.03	3	2.40E-12	0.88	3.90E-161
IL17	355	ENSG0000014877	MKI67	0.74	0.12	0.96	3.00E-10	0.46	2.30E-17
IL17	355	ENSG0000014879	INA	1.4	0.06	2.01	2.50E-14	2	6.80E-30
IL17	355	ENSG0000015101	NOCT	0.71	0.17	1.56	3.70E-09	2	5.40E-47
IL17	355	ENSG0000015146	CCDC3	0.8	-0.03	1.14	1.40E-05	2	4.20E-11
IL17	355	ENSG0000015165	ADAM8	1.04	0.16	2.43	7.10E-10	2	4.10E-55
IL17	355	ENSG0000015225	SPC25	0.59	0.13	0.88	6.80E-07	0.44	1.50E-15
IL17	355	ENSG0000015409	THY1	0.87	0.1	1.14	8.80E-45	0.24	7.60E-79
IL17	355	ENSG0000015413	ROBO4	0.99	0.27	0.7	8.70E-05	2	0.0055
IL17	355	ENSG0000015463	CXADR	1.06	-0.2	1.42	4.00E-05	2	7.90E-09
IL17	355	ENSG0000015509	KLF10	0.64	0.12	0.82	1.80E-29	0.11	2.40E-50
IL17	355	ENSG0000015584	PPARGC1B	0.67	0.24	0.88	0.016	2	6.40E-04
IL17	355	ENSG0000015646	SH3RF2	0.8	0.38	0.75	0.0099	2	0.011
IL17	355	ENSG0000015716	NRG1	0.74	-0.17	2.08	2.00E-07	0.38	6.20E-61
IL17	355	ENSG0000015736	IL34	2.59	-0.06	2.89	1.10E-104	0.75	3.60E-133
IL17	355	ENSG0000015805	DUSP2	0.67	0.39	0.8	0.0076	2	5.60E-04
IL17	355	ENSG0000015808	GALNT14	0.9	0.48	0.84	0.0044	2	0.0051
IL17	355	ENSG0000015812	XDH	0.79	0	0.83	0.012	2	0.0051
IL17	355	ENSG0000015831	RHBDL2	0.83	-0.03	1.19	0.006	2	3.10E-05
IL17	355	ENSG0000015885	ADAMTS4	0.59	0.07	1.48	5.90E-05	2	8.40E-32
IL17	355	ENSG0000015916	STC1	0.89	-0.02	1.51	1.50E-16	0.91	2.60E-49
IL17	355	ENSG0000016001	PTGIR	0.6	0.29	1.45	6.70E-07	0.055	6.80E-41
IL17	355	ENSG0000016005	CCDC28B	0.64	-0.01	1.19	1.40E-11	0.96	1.40E-41
IL17	355	ENSG0000016016	CILP2	0.9	-0.01	1.2	1.00E-05	2	3.20E-10
IL17	355	ENSG0000016095	RECQL4	0.66	0.28	0.91	9.00E-15	0.01	1.60E-29
IL17	355	ENSG0000016188	SPC24	0.68	0.33	0.66	2.30E-07	0.044	2.40E-07
IL17	355	ENSG0000016189	IP6K3	1.1	0.15	1.27	1.80E-13	2	1.10E-18
IL17	355	ENSG0000016206	CCNF	0.67	0.27	0.66	1.10E-13	0.02	1.10E-13
IL17	355	ENSG0000016249	DRAXIN	1.13	0.02	0.64	3.40E-04	2	0.037
IL17	355	ENSG0000016273	VANGL2	0.85	0.24	1	4.50E-07	2	4.20E-10
IL17	355	ENSG0000016313	CTSS	1.9	0.25	2.64	3.70E-40	0.25	8.30E-80
IL17	355	ENSG0000016334	CLDN1	0.69	0.16	2.72	1.20E-11	0.24	1.10E-197
IL17	355	ENSG0000016354	NUAK2	0.79	0.18	1.62	8.50E-11	0.28	2.00E-47
IL17	355	ENSG0000016361	CADPS	1.21	0.49	0.7	6.70E-11	2	2.80E-04
IL17	355	ENSG0000016373	CXCL1	0.59	1.17	2.9	2.40E-04	2	4.90E-105
IL17	355	ENSG0000016381	CDCP1	0.88	0.39	1.34	1.20E-34	2.00E-05	1.60E-83
IL17	355	ENSG0000016416	HHIP	0.67	-0.32	0.93	1.90E-07	2	9.10E-15
IL17	355	ENSG0000016431	EGFLAM	0.71	0.25	1.33	1.50E-04	2	2.80E-15
IL17	355	ENSG0000016434	TLR3	0.92	-0.1	0.59	8.00E-04	2	0.032
IL17	355	ENSG0000016487	MICALL2	0.79	0.15	1.41	1.20E-38	0.072	5.10E-129
IL17	355	ENSG0000016507	MAMDC2	0.69	0.21	1.22	3.10E-08	0.21	7.90E-26
IL17	355	ENSG0000016548	SKA3	0.66	0.26	1.15	3.70E-09	0.072	7.80E-29
IL17	355	ENSG0000016580	ZNF219	0.67	0.08	0.8	4.00E-10	0.61	5.50E-15
IL17	355	ENSG0000016586	HSPA12A	0.61	0.01	0.81	7.60E-16	0.96	3.70E-29
IL17	355	ENSG0000016589	E2F7	0.85	0.18	1.25	9.70E-22	0.13	3.80E-49
IL17	355	ENSG0000016634	DCHS1	0.68	0.19	0.59	3.60E-34	0.009	2.60E-26
IL17	355	ENSG0000016659	RRAD	1.19	-0.2	2.26	7.40E-22	0.28	1.90E-83
IL17	355	ENSG0000016686	TAC3	4.27	-0.18	4.52	2.00E-139	0.4	8.30E-157
IL17	355	ENSG0000016719	GPRC5B	0.88	0.12	1.7	1.70E-23	0.35	1.60E-90
IL17	355	ENSG0000016760	CYP2S1	1.09	0.07	1.31	2.60E-10	2	3.30E-15
IL17	355	ENSG0000016790	TK1	0.62	0.25	0.67	3.70E-17	0.0093	2.60E-20
IL17	355	ENSG0000016807	PBK	0.66	0.15	1.11	3.90E-10	0.3	3.00E-29
IL17	355	ENSG0000016839	TAP1	0.74	0.14	1.4	6.20E-28	0.13	3.50E-105
IL17	355	ENSG0000016862	GDNF	0.89	0.28	1.28	0.0041	2	1.50E-05
IL17	355	ENSG0000016908	C8orf46	0.66	0.06	0.86	0.035	2	0.0024
IL17	355	ENSG0000016925	GPRIN1	1.01	0.18	0.93	2.90E-37	0.11	2.60E-32
IL17	355	ENSG0000016943	COL22A1	2.29	0.33	2.64	3.30E-25	2	2.20E-33
IL17	355	ENSG0000016967	BUB1	0.68	0.2	0.91	5.10E-10	0.16	5.20E-18
IL17	355	ENSG0000016985	ROBO1	0.74	-0.07	1.04	1.40E-37	0.37	2.40E-76
IL17	355	ENSG0000016988	WNT10B	1.15	0.19	1.18	1.00E-07	2	1.80E-08
IL17	355	ENSG0000016990	TM4SF1	1.14	-0.01	1.94	4.60E-49	0.97	4.50E-145
IL17	355	ENSG0000017037	CST1	3.12	0.23	3.14	1.20E-59	2	2.20E-61
IL17	355	ENSG0000017087	MTSS1	0.59	-0.31	1.34	8.30E-12	0.007	3.60E-62
IL17	355	ENSG0000017124	SHCBP1	0.64	0.09	1.2	2.20E-13	0.47	8.30E-48
IL17	355	ENSG0000017132	ESCO2	0.63	0.15	1.49	3.30E-06	2	7.70E-36

IL17	355	ENSG0000017161	ENC1	0.66	0.17	1.05	1.20E-16	0.13	7.60E-44
IL17	355	ENSG0000017184	RRM2	0.59	0.33	1.27	7.60E-12	0.0034	1.20E-54
IL17	355	ENSG0000017187	FRMD5	1.1	-0.12	0.87	5.20E-17	2	5.50E-11
IL17	355	ENSG0000017202	GAP43	1.26	0.06	1.05	8.10E-32	2	2.60E-22
IL17	355	ENSG0000017292	MYEOV	1.02	0.34	0.95	1.00E-04	2	2.00E-04
IL17	355	ENSG0000017464	SLCO2A1	1.2	0.12	3.44	1.70E-11	2	5.20E-97
IL17	355	ENSG0000017506	UBE2C	0.75	0.27	0.73	7.10E-17	0.02	1.50E-16
IL17	355	ENSG0000017559	FOSL1	0.92	0.2	1.65	3.90E-31	0.056	3.40E-104
IL17	355	ENSG0000017617	SPHK1	0.7	0.05	1.65	5.00E-11	0.76	6.70E-67
IL17	355	ENSG0000017689	TYMS	0.65	0.16	1.06	5.30E-23	0.063	2.00E-61
IL17	355	ENSG0000017697	SHMT1	0.68	0.33	0.89	4.30E-07	2	1.40E-12
IL17	355	ENSG0000017760	HASPIN	0.82	0.4	1.09	9.70E-08	2	5.90E-14
IL17	355	ENSG0000017803	ADAMTSL1	0.93	0.12	1.3	1.80E-18	0.44	8.90E-38
IL17	355	ENSG0000017807	STAP2	0.64	-0.16	0.95	1.40E-04	2	8.50E-10
IL17	355	ENSG0000017899	AURKB	0.71	0.24	0.93	3.10E-13	0.063	2.40E-23
IL17	355	ENSG0000017910	TMTC2	1.94	0.19	1.65	9.80E-88	0.21	6.70E-63
IL17	355	ENSG0000017975	APOBEC3B	1.04	0.39	1.27	2.00E-22	0.006	9.20E-35
IL17	355	ENSG0000017984	AKAP5	0.6	0.19	1.11	0.0083	2	3.00E-08
IL17	355	ENSG0000018161	FDCSP	0.66	0.14	1.91	0.0014	2	1.40E-07
IL17	355	ENSG0000018177	GPR3	0.71	0.44	1.39	0.0054	2	1.30E-09
IL17	355	ENSG0000018202	CHST15	0.72	0.02	1.54	0.01	2	4.10E-10
IL17	355	ENSG0000018247	CAPN12	0.64	0.22	1.1	0.033	2	4.00E-05
IL17	355	ENSG0000018296	GJC1	0.62	0	1.07	1.20E-32	0.96	3.00E-98
IL17	355	ENSG0000018318	C2CD4C	0.72	0.45	0.92	0.022	2	0.0023
IL17	355	ENSG0000018371	OPCML	0.61	0.42	0.72	0.011	2	0.0012
IL17	355	ENSG0000018384	GPR39	1.27	0.14	1.46	1.60E-21	2	3.50E-29
IL17	355	ENSG0000018510	ANO9	1.09	0.04	2.08	3.10E-04	2	5.40E-09
IL17	355	ENSG0000018524	GP1BA	0.62	0.07	1.74	0.012	2	1.10E-05
IL17	355	ENSG0000018569	MYBL1	1.35	-0.02	2.13	1.90E-52	0.88	6.50E-137
IL17	355	ENSG0000018618	KIF18B	0.8	0.18	0.9	1.30E-17	0.15	9.80E-23
IL17	355	ENSG0000018663	KIF24	0.75	0.24	0.88	3.00E-08	2	1.90E-11
IL17	355	ENSG0000018687	ERCC6L	0.63	-0.01	1.12	3.20E-06	2	1.60E-19
IL17	355	ENSG0000018699	EMID1	1.38	0.32	1.44	1.50E-14	2	1.60E-16
IL17	355	ENSG0000018774	FANCA	0.71	0.32	1.24	1.20E-18	0.0026	3.00E-59
IL17	355	ENSG0000018801	S100A3	0.65	0.1	0.82	0.027	2	0.0024
IL17	355	ENSG0000018806	WNT7B	1.17	-0.1	1.75	4.90E-06	2	1.40E-12
IL17	355	ENSG0000018876	SPRED3	0.67	0.22	0.63	4.80E-08	2	2.60E-07
IL17	355	ENSG0000018914	CLDN4	0.72	0.33	1.16	4.40E-06	2	8.30E-16
IL17	355	ENSG0000018941	SH2D5	0.83	0.07	1.56	5.60E-12	0.7	3.80E-44
IL17	355	ENSG0000019646	RFX8	1.19	0.08	1.7	2.70E-11	2	1.70E-23
IL17	355	ENSG0000019661	MMP1	0.81	-0.04	1.1	2.20E-22	0.79	1.60E-43
IL17	355	ENSG0000019704	SIGLEC15	0.87	0.28	1.36	7.70E-04	2	1.20E-08
IL17	355	ENSG0000019714	ACSL5	0.77	0.14	1.41	1.90E-04	2	3.80E-14
IL17	355	ENSG0000019726	C6orf141	0.61	0.43	0.65	0.034	2	0.015
IL17	355	ENSG0000019729	BLM	0.66	-0.07	1.29	2.90E-07	0.74	6.90E-28
IL17	355	ENSG0000019744	MAP3K5	0.67	0.05	1.7	1.20E-19	0.69	4.30E-133
IL17	355	ENSG0000019750	SLC28A3	1.29	0.08	2.07	1.00E-05	2	7.00E-12
IL17	355	ENSG0000019797	ELOVL2	1.27	0.28	2.9	8.90E-09	2	1.30E-42
IL17	355	ENSG0000019880	PNP	0.69	0.18	1.7	3.40E-08	0.28	1.10E-50
IL17	355	ENSG0000019891	L1CAM	2.57	0.08	2.47	3.40E-79	0.76	7.00E-74
IL17	355	ENSG0000020438	SLC44A4	1.12	0.18	0.73	4.50E-04	2	0.015
IL17	355	ENSG0000020476	FAM196B	0.73	0.17	0.66	1.80E-06	2	1.10E-05
IL17	355	ENSG0000020543	EXOC3L4	0.91	0.25	1.37	0.0017	2	4.70E-06
IL17	355	ENSG0000020643	TMEM200C	1	0.13	1.26	1.40E-30	0.29	7.00E-51
IL17	355	ENSG0000021019	MT-TT	0.7	0.76	0.82	0.012	2	0.0015
IL17	355	ENSG0000021177	TRBC2	0.61	-0.04	0.63	0.037	2	0.02
IL17	355	ENSG0000021398	ZNF90	0.69	0.24	0.78	0.0031	2	3.50E-04
IL17	355	ENSG0000021482	DDX12P	0.72	0.47	1.12	0.0018	2	6.70E-08
IL17	355	ENSG0000021780	AL390719.1	1.25	0.13	1.44	4.70E-21	2	1.30E-28
IL17	355	ENSG0000022533	AL354740.1	0.91	0.06	0.79	3.90E-05	2	2.90E-04
IL17	355	ENSG0000022703	LINC00511	1.12	0.03	0.67	4.50E-06	2	0.0073
IL17	355	ENSG0000022810	AP000692.1	0.67	0.02	0.68	0.047	2	0.033
IL17	355	ENSG0000023474	HLA-B	0.75	0	0.77	3.30E-56	0.96	2.10E-60
IL17	355	ENSG0000023556	NFAM1	0.63	-0.07	1.51	0.03	2	3.50E-05
IL17	355	ENSG0000023668	HMGA1P1	1.02	0.28	1.11	0.0012	2	2.40E-04

IL17	355	ENSG0000023764	KIFC1	0.75	0.35	0.97	9.50E-18	0.0021	5.90E-30
IL17	355	ENSG0000023800	AC116347.1	0.66	0.24	0.76	0.033	2	0.0088
IL17	355	ENSG0000024006	PSMB9	0.92	0.06	1.37	5.60E-14	0.79	1.60E-32
IL17	355	ENSG0000024047	LINC00973	1	0.22	1.73	6.30E-04	2	1.30E-08
IL17	355	ENSG0000024364	CFB	0.67	0.47	1.03	0.012	2	2.00E-05
IL17	355	ENSG0000024374	RPLP0P2	1.09	0.05	1.37	1.40E-14	2	8.30E-24
IL17	355	ENSG0000024999	TMEM158	0.71	0.27	0.59	9.40E-22	0.006	8.40E-16
IL17	355	ENSG0000025065	AC097451.1	1.66	0.19	1.86	9.50E-11	2	1.80E-13
IL17	355	ENSG0000025316	LINC01605	0.98	0.15	1.17	9.40E-07	2	1.00E-09
IL17	355	ENSG0000025361	AC107959.3	0.78	0.12	0.98	0.011	2	6.30E-04
IL17	355	ENSG0000025383	AC090197.1	0.69	-0.01	0.64	0.028	2	0.03
IL17	355	ENSG0000025666	AC112777.1	0.61	0.35	0.86	0.016	2	1.80E-04
IL17	355	ENSG0000025716	TMPO-AS1	0.61	0.27	0.64	2.00E-05	2	2.20E-06
IL17	355	ENSG0000025721	LINC02407	0.96	0.09	1.27	2.10E-06	2	2.30E-11
IL17	355	ENSG0000025801	AC144548.1	0.64	0.51	0.72	0.049	2	0.017
IL17	355	ENSG0000026609	RASSF5	0.64	-0.13	0.59	0.0012	2	0.0021
IL17	355	ENSG0000027060	AC009549.1	0.73	0.04	1.57	4.10E-04	2	7.00E-18
IL17	355	ENSG0000027193	AC012073.1	0.65	0.12	0.69	0.0089	2	0.0034
IL17	355	ENSG0000027226	AL138724.1	0.81	0.03	1.68	4.20E-05	2	6.80E-21
IL17	355	ENSG0000027360	EPOP	0.67	0.26	1.21	0.014	2	4.60E-07
IL17	355	ENSG0000027563	AL035461.2	0.65	0.21	1.02	0.047	2	8.50E-04
IL17	355	ENSG0000027956	AC020763.4	0.62	0.08	1.02	0.034	2	7.90E-05
IL17	355	ENSG0000028134	HELLPAR	0.59	0.4	0.8	0.043	2	0.0019
IL17	355	ENSG0000028139	SNHG4	0.62	0.18	1.1	0.0032	2	4.50E-09
IL17	123	ENSG0000000324	DBNDD1	0.72	0.39	0.21	1.00E-04	2	0.36
IL17	123	ENSG0000000505	MCUB	0.61	0.11	0.5	8.50E-09	0.45	1.60E-06
IL17	123	ENSG0000000510	THSD7A	0.67	-0.1	0.07	0.0047	2	0.84
IL17	123	ENSG0000001329	SLC7A14	0.62	0.17	0.07	8.10E-08	0.25	0.67
IL17	123	ENSG0000005435	PTPRN	1.01	0.01	-0.12	5.80E-36	0.95	0.28
IL17	123	ENSG0000005516	CYFIP2	0.68	0.02	0.4	1.90E-08	0.94	0.0017
IL17	123	ENSG0000006624	NGEF	0.67	0.08	-0.24	5.70E-05	2	0.23
IL17	123	ENSG0000006692	STAG3	0.72	0.3	0.17	0.017	2	0.65
IL17	123	ENSG0000007283	CRMP1	0.72	0.27	0.09	0.0057	2	0.81
IL17	123	ENSG0000007925	LXN	0.63	0.11	0.48	7.00E-12	0.42	1.80E-07
IL17	123	ENSG0000008003	PTPRH	0.82	0.07	0.3	9.00E-04	2	0.3
IL17	123	ENSG0000008105	TCF7	0.65	-0.05	0.23	4.10E-11	0.74	0.042
IL17	123	ENSG0000008556	ABCB1	0.62	-0.11	0.44	0.0095	2	0.072
IL17	123	ENSG0000009936	STX1B	0.62	0.27	0.16	2.90E-07	2	0.29
IL17	123	ENSG0000010005	CYTH4	0.78	-0.21	0.41	0.016	2	0.22
IL17	123	ENSG0000010371	RASL12	0.71	0.15	-0.31	1.30E-13	0.26	0.0031
IL17	123	ENSG0000010432	TRPA1	0.62	-0.02	0.48	4.00E-20	0.85	6.80E-13
IL17	123	ENSG0000010879	ABI3	0.64	0.13	0.01	0.0017	2	0.99
IL17	123	ENSG0000010894	EFNB3	0.66	-0.03	-0.43	4.50E-05	2	0.016
IL17	123	ENSG0000010958	GALNT7	0.63	-0.18	0.4	1.20E-15	0.078	4.70E-07
IL17	123	ENSG0000011108	GLI1	0.76	0.09	0.06	2.90E-06	2	0.81
IL17	123	ENSG0000011298	KIF20A	0.61	0.27	0.2	4.50E-07	0.077	0.13
IL17	123	ENSG0000011516	CENPA	0.68	0.11	0.43	6.90E-08	0.53	9.10E-04
IL17	123	ENSG0000011889	PPL	0.75	0.14	-1.07	1.40E-27	0.13	5.10E-49
IL17	123	ENSG0000012087	DUSP4	1.14	-0.14	0.15	2.50E-17	2	0.42
IL17	123	ENSG0000012120	LRAT	0.93	-0.14	0.42	0.0037	2	0.22
IL17	123	ENSG0000012269	TWIST1	0.69	-0.02	-0.24	1.80E-15	0.92	0.014
IL17	123	ENSG0000012296	CIT	0.6	0.24	0.47	3.70E-12	0.033	5.20E-08
IL17	123	ENSG0000012446	LYPD3	0.59	-0.16	0.32	0.05	2	0.31
IL17	123	ENSG0000012589	FAM110A	0.66	0.23	0.3	4.90E-06	2	0.057
IL17	123	ENSG0000012596	ID1	0.66	0.25	0.06	2.50E-04	2	0.82
IL17	123	ENSG0000012856	VGF	0.88	-0.47	-0.41	2.10E-07	2	0.04
IL17	123	ENSG0000013047	FCHO1	0.6	0.19	-0.01	0.028	2	0.97
IL17	123	ENSG0000013059	LSP1	0.73	0.01	-0.42	4.70E-06	2	0.018
IL17	123	ENSG0000013083	MPP1	0.61	-0.05	0.43	3.20E-11	0.74	5.00E-06
IL17	123	ENSG0000013183	RAI2	0.83	0	0.25	3.50E-09	2	0.14
IL17	123	ENSG0000013466	SPOCD1	0.69	0.19	0.41	1.20E-17	0.072	6.40E-07
IL17	123	ENSG0000013495	SLC37A2	0.6	0.32	0.53	2.30E-04	2	9.40E-04
IL17	123	ENSG0000013542	ITGA7	0.79	0.07	0.52	7.30E-26	0.52	1.30E-11
IL17	123	ENSG0000013545	TROAP	0.61	0.21	0.44	3.00E-08	0.14	7.10E-05
IL17	123	ENSG0000013684	TMOD1	0.95	-0.22	0.36	0.0011	2	0.27

IL17	123	ENSG0000013713	ARHGEF39	0.67	0.29	0.31	2.70E-04	2	0.13
IL17	123	ENSG0000013816	TACC2	0.62	0.11	0.34	4.00E-24	0.17	9.30E-08
IL17	123	ENSG0000013941	SDSL	0.61	0.26	-0.11	0.0055	2	0.69
IL17	123	ENSG0000014047	PCSK6	0.82	-0.09	0.33	0.011	2	0.35
IL17	123	ENSG0000014336	ECM1	0.65	0.07	-0.04	1.60E-37	0.36	0.58
IL17	123	ENSG0000014346	SYT14	0.86	0.15	0.48	2.70E-04	2	0.052
IL17	123	ENSG0000014563	PLK2	0.75	0.06	0.43	8.30E-63	0.4	6.40E-21
IL17	123	ENSG0000014570	CRHBP	0.81	-0.04	0.3	1.10E-04	2	0.22
IL17	123	ENSG0000014655	SDK1	0.69	0.15	0.35	9.00E-36	0.038	6.90E-10
IL17	123	ENSG0000014743	CHRNB3	0.85	-0.07	0.41	0.0016	2	0.16
IL17	123	ENSG0000015055	LYPD1	0.7	0.29	0.28	2.30E-13	0.018	0.007
IL17	123	ENSG0000015323	NR4A2	0.63	0.09	-0.51	9.30E-08	2	7.70E-05
IL17	123	ENSG0000015414	NRGN	0.64	0.1	0.28	1.40E-06	2	0.058
IL17	123	ENSG0000015629	TIAM1	0.89	0.26	0.47	8.60E-06	2	0.026
IL17	123	ENSG0000015730	SUSD3	0.7	0.12	0.12	0.03	2	0.76
IL17	123	ENSG0000015825	CLSTN2	0.64	-0.21	0.39	0.043	2	0.22
IL17	123	ENSG0000015840	CDC25C	0.68	0.1	0.35	6.20E-08	2	0.009
IL17	123	ENSG0000015925	ACTC1	0.97	0.05	0.32	2.50E-04	2	0.3
IL17	123	ENSG0000015971	TPPP3	0.8	0.12	0.18	1.10E-04	2	0.49
IL17	123	ENSG0000016029	C21orf58	0.66	0.21	0.55	1.00E-10	0.12	6.60E-08
IL17	123	ENSG0000016138	PLXDC1	0.82	-0.07	0.48	8.80E-06	2	0.014
IL17	123	ENSG0000016252	KIAA1522	0.59	0.13	0.04	4.50E-15	0.2	0.73
IL17	123	ENSG0000016297	KCNF1	0.69	0.08	-0.23	2.70E-04	2	0.31
IL17	123	ENSG0000016412	HPGD	0.76	-0.02	0.57	0.023	2	0.074
IL17	123	ENSG0000016492	BAALC	0.75	-0.05	0.44	2.30E-30	0.62	6.30E-11
IL17	123	ENSG0000016647	PKD1L2	0.62	-0.01	-0.07	0.013	2	0.83
IL17	123	ENSG0000016685	PLK1	0.62	0.24	0.54	8.40E-11	0.053	1.70E-08
IL17	123	ENSG0000016776	KRT80	0.6	0.11	0.13	1.00E-05	2	0.44
IL17	123	ENSG0000016799	VWCE	0.95	0.08	0.41	4.00E-12	2	0.0075
IL17	123	ENSG0000016867	KCTD19	0.7	0.27	0.16	0.031	2	0.69
IL17	123	ENSG0000016975	TMEM266	0.71	-0.09	0.51	0.034	2	0.11
IL17	123	ENSG0000016989	REPS2	0.88	0	0.23	4.70E-04	2	0.45
IL17	123	ENSG0000017050	LONRF2	0.71	0.18	0.43	0.01	2	0.13
IL17	123	ENSG0000017080	HTRA3	0.75	0.11	0.23	2.70E-13	0.47	0.054
IL17	123	ENSG0000017124	NPTX1	0.72	-0.1	-0.56	1.70E-08	0.6	1.30E-05
IL17	123	ENSG0000017322	SYT12	0.73	0.51	0.63	0.039	2	0.06
IL17	123	ENSG0000017326	SNCG	0.76	0.16	-0.29	6.70E-06	2	0.14
IL17	123	ENSG0000017423	REP15	0.65	0.06	0.49	0.046	2	0.12
IL17	123	ENSG0000018304	CAMK1D	0.75	0.11	0.34	1.30E-13	0.44	0.0021
IL17	123	ENSG0000018313	PTGDR2	0.6	-0.1	-0.93	0.0047	2	1.00E-05
IL17	123	ENSG0000018319	CHST6	0.67	0.06	0.47	0.027	2	0.12
IL17	123	ENSG0000018350	FAM46C	0.68	-0.07	0.37	4.50E-07	2	0.0085
IL17	123	ENSG0000018385	IQGAP3	0.71	0.4	0.48	3.70E-20	2.70E-05	5.70E-10
IL17	123	ENSG0000018479	OSBP2	0.67	0.09	0.4	7.70E-06	2	0.01
IL17	123	ENSG0000018533	TCN2	0.69	0.08	-0.07	2.50E-14	0.57	0.57
IL17	123	ENSG0000018536	TNFAIP8L1	0.63	0.27	0.42	1.30E-13	0.014	1.20E-06
IL17	123	ENSG0000018556	AHNAK2	0.78	0.12	-0.37	4.20E-22	0.27	1.40E-05
IL17	123	ENSG0000018679	HYAL3	0.59	0.07	0.28	0.0092	2	0.25
IL17	123	ENSG0000018689	TNFRSF18	0.92	-0.21	0.39	4.30E-04	2	0.17
IL17	123	ENSG0000018768	TRPV2	0.64	0.07	0.21	8.80E-15	0.57	0.027
IL17	123	ENSG0000018786	PALM3	0.86	0.15	0.15	9.70E-07	2	0.52
IL17	123	ENSG0000019683	ADA	0.69	0.18	0.04	6.20E-21	0.062	0.71
IL17	123	ENSG0000019706	HIST1H4C	0.72	0.26	0.37	0.036	2	0.27
IL17	123	ENSG0000019732	SVIL	0.59	-0.01	0.49	1.20E-29	0.92	2.30E-20
IL17	123	ENSG0000019745	STMN3	0.81	0.14	0.58	1.20E-32	0.13	4.10E-17
IL17	123	ENSG0000019774	PTMAP2	0.72	0.07	0.52	0.0056	2	0.045
IL17	123	ENSG0000019828	CARD11	0.88	-0.03	0.17	2.00E-05	2	0.52
IL17	123	ENSG0000019873	SMOC1	0.74	0.16	0.28	9.20E-11	0.32	0.028
IL17	123	ENSG0000020008	SNORA73B	1.37	0.11	0.25	1.30E-04	2	0.33
IL17	123	ENSG0000020413	NHSL2	0.62	-0.06	-0.03	7.60E-06	2	0.87
IL17	123	ENSG0000020533	ADGRG1	0.8	0.05	0.46	1.50E-19	0.69	2.80E-07
IL17	123	ENSG0000020634	HLA-H	0.69	0.02	0.56	2.30E-13	0.91	4.50E-09
IL17	123	ENSG0000020650	HLA-A	0.62	0.05	0.51	9.60E-35	0.48	1.20E-24
IL17	123	ENSG0000021145	MT-RNR1	0.98	-0.02	-0.29	0.0035	0.97	0.39
IL17	123	ENSG0000021479	AC098934.1	0.66	0.3	0.55	0.037	2	0.075

IL17	123	ENSG0000022578	MIAT	1.54	0.17	0.14	6.40E-32	0.37	0.47
IL17	123	ENSG0000023061	AL139220.2	0.75	0.57	0.37	0.0017	2	0.15
IL17	123	ENSG0000023369	GAS6-AS1	0.79	0.26	-0.09	5.00E-06	2	0.72
IL17	123	ENSG0000023438	CTBP2P8	0.77	-0.1	-0.11	0.0056	2	0.75
IL17	123	ENSG0000023549	AC073046.1	0.84	0.58	0.57	0.011	2	0.077
IL17	123	ENSG0000024521	AC105285.1	0.8	0.01	-0.04	1.10E-04	2	0.9
IL17	123	ENSG0000024564	AC022075.1	0.72	0.2	0.34	0.033	2	0.33
IL17	123	ENSG0000025004	AC069360.1	0.7	-0.22	0.07	0.02	2	0.87
IL17	123	ENSG0000025248	SCARNA13	1.09	-0.03	0.31	0.0018	2	0.39
IL17	123	ENSG0000026393	SNORD3A	1.11	0	0	5.00E-04	2	0.95
IL17	123	ENSG0000026703	AC011498.2	0.73	0.18	0.54	0.028	2	0.09
IL17	123	ENSG0000026881	AC004264.1	0.8	0.46	0.27	0.013	2	0.44
IL17	123	ENSG0000027164	AC112220.4	0.65	-0.08	0.18	0.036	2	0.62
IL17	123	ENSG0000027571	HIST1H2BH	0.71	-0.04	-0.26	0.036	2	0.45
IL17	123	ENSG0000027664	DACH1	0.62	-0.19	-0.25	7.00E-04	2	0.24
IL17	123	ENSG0000027735	AC013553.3	0.84	0.57	0.17	0.0066	2	0.67
IL17	123	ENSG0000028021	AC027682.7	0.8	0.25	0.55	0.013	2	0.081
IL17	1	ENSG0000014480	NFKBIZ	-0.11	0.84	0.91	0.44	2.50E-18	2.00E-24
IL17	1070	ENSG0000016502	ABCA1	-0.33	-0.06	0.59	1.40E-04	0.63	1.90E-14
IL17	1070	ENSG0000016416	ABCE1	0.08	-0.09	0.89	0.37	0.28	1.70E-54
IL17	1070	ENSG0000016017	ABCG1	0.09	0.44	0.95	0.87	2	0.0013
IL17	1070	ENSG0000015820	ABHD3	0.18	0.15	0.74	0.29	2	3.30E-10
IL17	1070	ENSG0000015417	ABI3BP	-0.2	0.14	0.96	0.11	0.28	4.50E-25
IL17	1070	ENSG0000014332	ABL2	0.35	-0.08	0.7	1.20E-06	0.39	3.00E-25
IL17	1070	ENSG0000016601	ABTB2	0.46	0.28	0.79	0.1	2	0.001
IL17	1070	ENSG0000023645	AC003092.1	0.18	0.3	0.91	2	2	0.0041
IL17	1070	ENSG0000026082	AC004656.1	0.54	-0.06	0.66	0.083	2	0.02
IL17	1070	ENSG0000024039	AC004801.2	0.4	0.15	0.66	2	2	0.029
IL17	1070	ENSG0000028020	AC005831.1	0.41	0.15	0.88	0.23	2	0.0026
IL17	1070	ENSG0000025643	AC005840.3	0.14	0.08	0.64	0.76	2	0.044
IL17	1070	ENSG0000027290	AC006033.2	0.41	-0.21	0.7	2	2	0.034
IL17	1070	ENSG0000026178	AC006058.1	0.35	0.02	1.42	0.018	2	8.70E-34
IL17	1070	ENSG0000026712	AC008105.3	0.48	0.33	0.78	0.16	2	0.0075
IL17	1070	ENSG0000023349	AC008163.1	0.43	0.08	0.67	2	2	0.037
IL17	1070	ENSG0000028007	AC011447.7	0.28	-0.11	0.77	0.47	2	0.0094
IL17	1070	ENSG0000028023	AC011498.7	0.5	0.22	0.61	0.077	2	0.016
IL17	1070	ENSG0000027982	AC016397.2	0.5	0.33	1.41	0.087	2	3.20E-09
IL17	1070	ENSG0000027330	AC018690.1	-0.24	0.23	0.75	0.47	2	0.021
IL17	1070	ENSG0000027271	AC019069.1	0.13	-0.02	0.68	0.66	2	5.00E-04
IL17	1070	ENSG0000022905	AC020571.1	0.27	0.07	0.93	2	2	0.0046
IL17	1070	ENSG0000026982	AC022150.4	0.46	-0.21	0.6	0.014	2	4.50E-04
IL17	1070	ENSG0000025680	AC022613.1	0.43	-0.16	0.64	0.14	2	0.011
IL17	1070	ENSG0000025971	AC023906.5	0.18	-0.1	0.78	2	2	0.015
IL17	1070	ENSG0000027187	AC024060.1	0.25	-0.17	0.76	0.51	2	0.0067
IL17	1070	ENSG0000025074	AC025419.1	0.14	0.17	0.69	0.77	2	0.03
IL17	1070	ENSG0000027055	AC025449.1	0.34	0.34	0.73	0.35	2	0.02
IL17	1070	ENSG0000027233	AC067838.1	0.29	-0.09	0.65	0.48	2	0.048
IL17	1070	ENSG0000024163	AC069499.1	0.03	-0.26	0.71	0.95	2	0.011
IL17	1070	ENSG0000023787	AC073130.1	0.31	0.07	0.62	0.4	2	0.046
IL17	1070	ENSG0000025562	AC073585.1	0.38	0.24	0.95	0.3	2	0.0022
IL17	1070	ENSG0000026788	AC074135.1	0.25	0.26	0.62	0.44	2	0.0086
IL17	1070	ENSG0000024005	AC078785.1	0.16	0.22	0.83	0.72	2	0.004
IL17	1070	ENSG0000024968	AC079921.2	0.08	-0.16	0.61	0.87	2	0.029
IL17	1070	ENSG0000026739	AC090229.1	0.36	0.07	0.68	2	2	0.036
IL17	1070	ENSG0000026997	AC091057.3	0.52	0.31	0.78	0.12	2	0.011
IL17	1070	ENSG0000025374	AC091182.2	0.58	0.17	0.7	0.085	2	0.025
IL17	1070	ENSG0000023203	AC092168.2	0.23	0.07	0.7	2	2	0.034
IL17	1070	ENSG0000026106	AC092718.4	0.52	0.04	0.72	0.0015	2	1.20E-06
IL17	1070	ENSG0000027911	AC093535.2	0.2	-0.06	0.68	0.53	2	0.0019
IL17	1070	ENSG0000023338	AC096537.1	0.53	0.39	0.67	0.12	2	0.03
IL17	1070	ENSG0000026142	AC097461.1	0.26	0.31	1.1	0.51	2	2.30E-04
IL17	1070	ENSG0000026087	AC104072.1	0.25	-0.02	0.78	0.53	2	0.0081
IL17	1070	ENSG0000022799	AC108463.1	0.45	0.03	0.59	0.053	2	0.0046
IL17	1070	ENSG0000026019	AC124798.1	0.48	-0.09	0.95	0.013	2	7.20E-09
IL17	1070	ENSG0000025089	AC125807.2	0.23	0	0.79	0.32	2	2.10E-06

IL17	1070	ENSG0000018013	ACTA2-AS1	0.11	0.22	0.68	0.82	2	0.026
IL17	1070	ENSG0000016906	ACTBL2	0.45	0.2	0.7	2	2	0.031
IL17	1070	ENSG0000011494	ADAM23	0.17	0.05	0.6	0.041	0.63	1.60E-19
IL17	1070	ENSG0000013402	ADAMDEC1	-0.07	0.18	0.67	0.63	2	0.012
IL17	1070	ENSG0000015614	ADAMTS3	0.07	-0.21	1.12	0.81	2	7.20E-13
IL17	1070	ENSG0000004919	ADAMTS6	0.28	-0.09	0.84	0.0036	0.48	5.50E-24
IL17	1070	ENSG0000016474	ADCY1	-0.02	0.23	0.69	0.96	2	0.017
IL17	1070	ENSG0000012750	ADGRE2	0.38	0.23	0.93	0.25	2	4.60E-04
IL17	1070	ENSG0000012314	ADGRE5	0.56	0.14	1	9.60E-17	0.13	1.90E-54
IL17	1070	ENSG0000017369	ADGRG2	-0.08	-0.24	0.86	2	2	0.015
IL17	1070	ENSG0000016261	ADGRL4	0.24	-0.29	0.75	0.063	0.044	2.60E-13
IL17	1070	ENSG0000017042	ADORA2B	0.43	0.31	0.9	0.015	2	1.80E-09
IL17	1070	ENSG0000014484	ADPRH	0.41	0.01	0.77	0.15	2	0.0011
IL17	1070	ENSG0000011186	ADTRP	0.46	-0.32	0.64	0.17	2	0.028
IL17	1070	ENSG0000018102	AEN	0.4	0.18	0.79	1.90E-09	0.034	3.40E-36
IL17	1070	ENSG0000017249	AFF1	0.34	-0.07	0.76	1.40E-05	0.54	4.70E-26
IL17	1070	ENSG0000011677	AGMAT	0.42	0.35	0.91	0.16	2	1.90E-04
IL17	1070	ENSG0000015518	AGPAT5	0.19	0.09	0.89	0.021	0.37	3.40E-42
IL17	1070	ENSG0000010654	AHR	0.18	0.04	0.7	0.0099	0.66	3.50E-33
IL17	1070	ENSG0000023792	AL078604.2	0.11	0.01	0.64	2	2	0.031
IL17	1070	ENSG0000025873	AL121603.2	0.29	0.12	0.66	0.11	2	3.20E-06
IL17	1070	ENSG0000027667	AL161891.1	0.43	0.35	1.09	0.18	2	2.40E-04
IL17	1070	ENSG0000026997	AL162424.1	0.37	-0.02	0.83	0.34	2	0.012
IL17	1070	ENSG0000026045	AL355607.2	0.53	-0.04	1.17	2	2	7.40E-04
IL17	1070	ENSG0000023749	AL357060.2	0.29	0.19	0.73	0.46	2	0.019
IL17	1070	ENSG0000026166	AL359752.1	0.22	0.03	0.72	0.56	2	0.0072
IL17	1070	ENSG0000027305	AL359921.2	0.18	0.27	0.7	0.68	2	0.028
IL17	1070	ENSG0000027303	AL365203.3	0.24	0	0.59	0.33	2	0.0011
IL17	1070	ENSG0000025983	AL365361.1	0.54	-0.26	1.29	0.029	2	4.80E-10
IL17	1070	ENSG0000023416	AL513165.1	0.26	0.18	0.68	0.3	2	2.40E-04
IL17	1070	ENSG0000027223	AL590438.1	0.28	0.19	0.8	2	2	0.01
IL17	1070	ENSG0000013712	ALDH1B1	0.46	0.15	0.85	2.90E-09	0.13	1.80E-31
IL17	1070	ENSG0000019879	ALPK2	-0.06	-0.04	0.98	0.47	0.64	1.70E-75
IL17	1070	ENSG0000012350	AMD1	-0.04	0.02	0.77	0.63	0.79	5.40E-46
IL17	1070	ENSG0000011619	ANGPTL1	0.21	-0.06	0.77	0.61	2	0.0077
IL17	1070	ENSG0000016011	ANKLE1	0.33	0.05	0.85	0.26	2	2.20E-04
IL17	1070	ENSG0000017691	ANKLE2	0.18	0.07	0.66	3.40E-04	0.25	6.50E-51
IL17	1070	ENSG0000014340	ANP32E	0.25	-0.19	0.68	0.0038	0.065	4.20E-19
IL17	1070	ENSG0000018271	ANXA2	0.26	0	0.6	3.60E-08	0.99	1.90E-42
IL17	1070	ENSG0000023199	ANXA2P2	0.37	0.18	0.65	1.50E-05	0.094	3.70E-17
IL17	1070	ENSG0000023047	AP000695.1	0.2	0.56	0.65	2	2	0.034
IL17	1070	ENSG0000026541	AP001094.2	0.58	0.09	0.68	2	2	0.036
IL17	1070	ENSG0000022441	AP001476.1	0.55	0.47	1.13	0.048	2	2.80E-06
IL17	1070	ENSG0000025366	AP003356.1	0.48	-0.03	0.59	0.13	2	0.035
IL17	1070	ENSG0000015205	AP1S3	0.15	-0.09	1	0.66	2	9.60E-07
IL17	1070	ENSG0000001113	APBA3	0.43	0.17	0.62	7.40E-08	0.1	2.40E-16
IL17	1070	ENSG0000016369	APBB2	0.39	-0.06	0.76	6.90E-12	0.49	1.50E-46
IL17	1070	ENSG0000012828	APOL3	0.54	0.1	0.71	2.90E-04	0.66	2.90E-07
IL17	1070	ENSG0000022196	APOL6	0.03	-0.06	0.62	0.78	0.54	4.10E-20
IL17	1070	ENSG0000010932	AREG	0.42	0.07	0.73	0.11	2	0.0011
IL17	1070	ENSG0000019882	ARHGAP11A	0.42	0.05	0.87	5.80E-06	0.72	1.30E-24
IL17	1070	ENSG0000014637	ARHGAP18	0.56	-0.12	0.77	7.90E-12	0.28	4.50E-23
IL17	1070	ENSG0000016589	ARHGAP42	0.22	-0.02	0.98	0.082	0.91	1.80E-24
IL17	1070	ENSG0000018044	ARHGAP45	0.37	0.05	0.88	0.33	2	0.0061
IL17	1070	ENSG0000011134	ARHGDIB	0.35	0.07	0.84	0.29	2	0.0013
IL17	1070	ENSG0000021494	ARHGEF28	0.34	0.02	0.76	2.50E-07	0.87	1.20E-35
IL17	1070	ENSG0000012264	ARL4A	0.49	-0.03	0.73	2.20E-05	0.85	1.40E-11
IL17	1070	ENSG0000018804	ARL4C	0.34	0.03	0.65	1.20E-10	0.75	6.20E-38
IL17	1070	ENSG0000019650	ARL9	0.61	0.54	0.68	0.052	2	0.022
IL17	1070	ENSG0000017237	ARNT2	0.33	-0.01	0.6	9.90E-06	0.96	2.70E-18
IL17	1070	ENSG0000015680	ATAD2	0.3	-0.01	1.11	6.70E-05	0.94	8.80E-64
IL17	1070	ENSG0000019778	ATAD3A	0.33	0.19	0.74	9.80E-06	0.038	6.50E-28
IL17	1070	ENSG0000016007	ATAD3B	0.29	0.34	0.64	0.0011	9.80E-04	4.00E-16
IL17	1070	ENSG0000017620	ATAD5	0.42	-0.02	1.09	0.0019	2	4.10E-21
IL17	1070	ENSG0000016801	ATG16L2	0.4	0.27	0.59	9.10E-04	0.057	7.60E-08

IL17	1070	ENSG0000013365	ATP13A3	0.06	-0.18	0.62	0.56	0.044	3.00E-21
IL17	1070	ENSG0000014315	ATP1B1	0.23	-0.07	0.59	0.058	0.65	2.30E-09
IL17	1070	ENSG0000007096	ATP2B1	-0.03	-0.26	0.92	0.77	0.0011	1.20E-56
IL17	1070	ENSG0000015919	ATP5G1	0.47	0.19	0.83	1.10E-07	0.09	3.20E-24
IL17	1070	ENSG0000020493	ATP6V0E2-AS1	0.32	0.04	0.61	0.17	2	0.0012
IL17	1070	ENSG0000014388	ATP6V1C2	0.27	0.07	0.62	2	2	0.048
IL17	1070	ENSG0000010404	ATP8B4	-0.5	0.13	0.62	0.076	2	0.013
IL17	1070	ENSG0000017505	ATR	0.02	-0.09	0.66	0.89	0.37	1.30E-20
IL17	1070	ENSG0000012742	AUNIP	0.48	0.11	1.2	0.053	2	4.60E-09
IL17	1070	ENSG0000017231	B3GALT1	-0.18	-0.21	0.59	0.57	2	0.0098
IL17	1070	ENSG0000013545	B4GALNT1	0.17	-0.11	0.72	0.7	2	0.016
IL17	1070	ENSG0000011827	B4GALT6	0.14	0.15	0.59	0.61	2	0.0012
IL17	1070	ENSG0000009573	BAMBI	0.29	0.07	0.76	0.096	2	4.50E-08
IL17	1070	ENSG0000013837	BARD1	0.23	0.1	0.82	0.063	0.52	1.30E-16
IL17	1070	ENSG0000012368	BATF3	0.01	0.34	0.74	0.99	2	2.50E-05
IL17	1070	ENSG0000019860	BAZ1A	0.33	-0.06	1.09	5.70E-06	0.56	2.00E-65
IL17	1070	ENSG0000017179	BCL2	0.28	0.02	0.74	0.083	2	1.60E-08
IL17	1070	ENSG0000006939	BCL3	0.35	0.38	0.7	2.90E-08	7.80E-08	8.00E-33
IL17	1070	ENSG0000011098	BCL7A	0.14	0.05	0.75	0.22	0.72	4.50E-19
IL17	1070	ENSG0000010073	BDKRB1	0.56	0.43	1.03	9.90E-05	2	1.90E-15
IL17	1070	ENSG0000017840	BEND3	0.09	0.03	0.78	0.67	2	6.80E-10
IL17	1070	ENSG0000001547	BID	0.4	0.17	0.8	3.80E-07	0.084	1.50E-28
IL17	1070	ENSG0000011033	BIRC2	0.07	-0.15	0.78	0.27	0.015	1.40E-66
IL17	1070	ENSG0000016321	BMP10	0.35	0	0.94	2	2	3.30E-04
IL17	1070	ENSG0000013875	BMP2K	-0.24	-0.15	0.69	1.20E-04	0.047	1.70E-36
IL17	1070	ENSG0000016461	BMPER	-0.35	0.2	1.34	0.0011	0.1	1.90E-54
IL17	1070	ENSG0000016959	BNC1	0.54	0.05	0.9	0.0011	2	1.10E-09
IL17	1070	ENSG0000026123	BOP1	0.43	0.22	0.89	3.30E-09	0.018	5.00E-40
IL17	1070	ENSG0000001204	BRCA1	0.47	0.03	1.04	3.20E-06	0.85	5.30E-30
IL17	1070	ENSG0000013961	BRCA2	0.37	-0.04	1.01	7.20E-04	0.79	3.60E-26
IL17	1070	ENSG0000018499	BRI3BP	0.35	0.13	1.02	0.0021	0.37	2.30E-26
IL17	1070	ENSG0000013649	BRIP1	0.33	-0.12	0.84	0.0011	0.34	2.60E-21
IL17	1070	ENSG0000015464	BTG3	0.04	0.06	0.59	0.68	0.53	1.90E-21
IL17	1070	ENSG0000012450	BTN2A2	0.44	0.02	1.49	7.80E-04	0.93	2.90E-41
IL17	1070	ENSG0000015697	BUB1B	0.52	-0.01	0.78	3.20E-07	0.93	6.60E-16
IL17	1070	ENSG0000015447	BUB3	0.25	0.02	0.59	3.90E-06	0.81	7.50E-33
IL17	1070	ENSG0000019840	BZW1P2	0.09	-0.1	0.63	0.83	2	0.0087
IL17	1070	ENSG0000016550	C10orf10	-0.3	0.07	0.7	0.0018	0.6	7.60E-17
IL17	1070	ENSG0000018299	C12orf60	0.11	0.06	0.77	0.82	2	0.0074
IL17	1070	ENSG0000018534	C14orf80	0.55	0.19	1.07	9.00E-06	0.24	1.70E-21
IL17	1070	ENSG0000016692	C15orf48	0.1	0.24	0.62	2	2	0.046
IL17	1070	ENSG0000016206	C16orf59	0.57	0.4	0.98	2.40E-05	2	5.00E-16
IL17	1070	ENSG0000010639	C1GALT1	0.17	-0.13	0.86	0.13	0.29	8.80E-25
IL17	1070	ENSG0000000046	C1orf112	0.52	0.2	0.83	1.40E-05	0.19	6.80E-14
IL17	1070	ENSG0000017391	C1QTNF1	0.36	0.03	0.72	2.20E-07	0.77	1.10E-29
IL17	1070	ENSG0000016627	C2	0.1	0.58	0.68	0.84	2	0.0042
IL17	1070	ENSG0000015761	C2CD2	0.49	0.05	0.96	2.10E-15	0.6	3.10E-60
IL17	1070	ENSG0000022197	C3orf36	0.43	0.07	1.17	2	2	4.50E-05
IL17	1070	ENSG0000018174	C3orf58	0.08	-0.1	1.11	0.41	0.32	4.70E-65
IL17	1070	ENSG0000020520	C4orf46	0.18	-0.03	0.63	0.082	0.82	5.20E-15
IL17	1070	ENSG0000017224	C5orf34	0.39	0.14	0.73	0.028	2	1.50E-06
IL17	1070	ENSG0000017690	C8orf4	-0.12	0.08	0.99	0.59	2	4.20E-14
IL17	1070	ENSG0000014789	C9orf72	-0.09	-0.03	0.83	0.68	2	8.50E-11
IL17	1070	ENSG0000016518	C9orf84	-0.08	0.35	0.78	2	2	0.028
IL17	1070	ENSG0000018501	CA13	0.44	0.06	0.88	0.16	2	7.50E-04
IL17	1070	ENSG0000015395	CACNA2D1	0.06	-0.12	0.64	0.64	0.25	7.60E-18
IL17	1070	ENSG0000018238	CACNB4	0.19	0.06	0.59	0.66	2	0.046
IL17	1070	ENSG0000008477	CAD	0.32	0.21	0.6	3.90E-09	0.0023	9.80E-31
IL17	1070	ENSG0000008180	CADPS2	0.23	0.1	0.85	0.047	0.46	8.30E-20
IL17	1070	ENSG0000017389	CBX2	0.46	0.15	0.59	2.20E-05	0.3	5.30E-09
IL17	1070	ENSG0000016300	CCDC138	0.27	0.04	0.81	0.27	2	1.00E-05
IL17	1070	ENSG0000014954	CCDC15	0.26	-0.25	0.66	0.23	2	6.80E-05
IL17	1070	ENSG0000014439	CCDC150	0.55	0.14	1.04	0.0093	2	2.30E-08
IL17	1070	ENSG0000012248	CCDC18	0.23	0.05	0.61	0.071	0.78	1.50E-09
IL17	1070	ENSG0000011747	CCDC181	0.34	0.05	0.68	0.23	2	0.0031

IL17	1070	ENSG0000017615	CCDC57	0.32	0.15	0.59	4.50E-07	0.058	1.50E-23
IL17	1070	ENSG0000011010	CCDC86	0.3	0.14	0.94	1.70E-04	0.16	7.80E-42
IL17	1070	ENSG0000014538	CCNA2	0.53	0.16	0.63	1.50E-07	0.23	5.70E-11
IL17	1070	ENSG0000010517	CCNE1	0.41	0.19	0.88	0.025	2	1.50E-08
IL17	1070	ENSG0000017530	CCNE2	0.35	0.24	1.57	0.02	0.16	2.10E-40
IL17	1070	ENSG0000010354	CCP110	0.06	-0.16	0.65	0.72	0.23	1.40E-12
IL17	1070	ENSG0000016622	CCT2	0.18	0.09	0.6	1.40E-04	0.12	4.60E-45
IL17	1070	ENSG0000015075	CCT5	0.23	0.07	0.77	1.30E-05	0.27	2.50E-58
IL17	1070	ENSG0000014673	CCT6A	0.15	-0.12	0.68	0.0048	0.041	9.80E-54
IL17	1070	ENSG0000012021	CD274	0.22	0.25	1.47	0.27	2	2.90E-26
IL17	1070	ENSG0000016777	CD320	0.45	0.19	0.61	6.00E-09	0.053	8.90E-17
IL17	1070	ENSG0000017405	CD34	0.34	0.31	1.01	2	2	0.0016
IL17	1070	ENSG0000011787	CD3EAP	0.31	0.12	1.08	0.014	0.45	3.60E-25
IL17	1070	ENSG0000019677	CD47	-0.02	-0.08	0.64	0.83	0.26	6.70E-35
IL17	1070	ENSG0000011681	CD58	0.19	-0.19	0.62	0.43	2	2.60E-04
IL17	1070	ENSG0000007933	CDC14A	0.15	0	0.78	0.47	2	2.50E-09
IL17	1070	ENSG0000016404	CDC25A	0.48	0.3	1.43	3.20E-05	0.037	4.00E-46
IL17	1070	ENSG0000009480	CDC6	0.45	0.16	1.29	2.40E-08	0.11	1.10E-70
IL17	1070	ENSG0000009704	CDC7	0.34	0.05	0.93	0.0091	0.79	8.80E-18
IL17	1070	ENSG0000018466	CDCA2	0.51	0.06	0.66	9.20E-06	0.74	1.70E-09
IL17	1070	ENSG0000017077	CDCA4	0.53	0.28	0.81	7.60E-09	0.014	1.70E-20
IL17	1070	ENSG0000014435	CDCA7	0.57	0.38	1.37	1.20E-09	0.0013	5.70E-59
IL17	1070	ENSG0000014094	CDH13	0.52	-0.04	0.64	1.20E-17	0.65	2.20E-27
IL17	1070	ENSG0000017031	CDK1	0.42	0.11	0.84	7.40E-06	0.39	5.10E-23
IL17	1070	ENSG0000012337	CDK2	0.36	0.18	0.65	1.80E-07	0.041	6.20E-24
IL17	1070	ENSG0000017674	CDK5R1	0.34	0.09	0.75	0.15	2	5.70E-05
IL17	1070	ENSG0000010581	CDK6	0.09	-0.22	0.69	0.28	0.008	2.30E-27
IL17	1070	ENSG0000016751	CDT1	0.52	0.19	0.75	1.10E-11	0.058	5.90E-25
IL17	1070	ENSG0000011581	CEBPZ	-0.08	-0.16	0.63	0.3	0.047	4.10E-28
IL17	1070	ENSG0000010238	CENPI	0.42	0.04	0.99	2.70E-04	0.82	3.80E-22
IL17	1070	ENSG0000015184	CENPJ	0.3	-0.05	0.85	0.0038	0.7	1.20E-21
IL17	1070	ENSG0000012321	CENPK	0.4	0.13	1.19	7.60E-04	0.38	2.20E-31
IL17	1070	ENSG0000012033	CENPL	0.22	0.02	0.72	0.1	0.94	2.00E-12
IL17	1070	ENSG0000016645	CENPN	0.32	0.02	0.84	5.10E-04	0.88	4.60E-26
IL17	1070	ENSG0000015172	CENPU	0.38	0.05	1.1	0.0012	0.75	2.90E-27
IL17	1070	ENSG0000020376	CENPW	0.47	0.03	0.66	3.10E-04	2	3.50E-08
IL17	1070	ENSG0000016968	CENPX	0.25	0.1	0.72	0.019	0.46	2.30E-15
IL17	1070	ENSG0000010062	CEP128	0.54	0.2	1.11	5.10E-04	2	8.10E-16
IL17	1070	ENSG0000010399	CEP152	0.35	0.03	0.72	0.0034	0.88	9.60E-12
IL17	1070	ENSG0000013818	CEP55	0.51	0.05	0.92	1.60E-07	0.75	4.70E-24
IL17	1070	ENSG0000013069	CEP85	0.53	0.23	0.82	3.90E-09	0.05	2.20E-22
IL17	1070	ENSG0000017282	CES3	0.44	0.36	0.61	2	2	0.046
IL17	1070	ENSG0000000097	CFH	0.34	0.07	0.76	1.70E-05	0.5	1.10E-25
IL17	1070	ENSG0000000162	CFTR	0.56	0.19	1.83	3.50E-05	2	1.00E-55
IL17	1070	ENSG0000012884	CGNL1	0.57	-0.15	1.39	0.0018	2	2.30E-18
IL17	1070	ENSG0000013813	CH25H	-0.1	0.31	0.72	0.77	2	0.023
IL17	1070	ENSG0000014394	CHAC2	-0.19	-0.07	0.72	0.54	2	4.70E-04
IL17	1070	ENSG0000016767	CHAF1A	0.49	0.21	1.08	8.70E-11	0.028	1.10E-52
IL17	1070	ENSG0000015925	CHAF1B	0.58	0.26	0.82	4.90E-10	0.032	8.40E-21
IL17	1070	ENSG0000017131	CHD7	0.13	-0.06	0.95	0.56	2	5.60E-11
IL17	1070	ENSG0000014955	CHEK1	0.23	-0.07	0.68	0.0061	0.53	1.70E-21
IL17	1070	ENSG0000011017	CHORDC1	0.07	-0.15	0.65	0.46	0.096	4.30E-25
IL17	1070	ENSG0000005493	CHRDL2	-0.26	0.41	0.83	0.32	2	5.20E-06
IL17	1070	ENSG0000016968	CHRNA5	0.52	-0.02	1.1	0.056	2	1.70E-06
IL17	1070	ENSG0000017131	CHST11	0.21	0.27	1.42	0.22	2	4.70E-34
IL17	1070	ENSG0000017504	CHST2	0.25	0.12	0.96	0.019	0.34	1.20E-28
IL17	1070	ENSG0000012287	CISD1	0.35	0.04	0.86	0.0038	0.81	3.00E-17
IL17	1070	ENSG0000017986	CITED4	0.58	0	0.7	3.60E-04	2	3.60E-06
IL17	1070	ENSG0000013610	CKAP2	0.41	0.02	0.65	3.40E-06	0.86	6.40E-15
IL17	1070	ENSG0000016960	CKAP2L	0.5	-0.02	0.94	1.50E-05	0.93	7.40E-19
IL17	1070	ENSG0000017320	CKS1B	0.32	0.14	0.61	0.0018	0.28	1.90E-11
IL17	1070	ENSG0000013797	CLCA2	-0.08	0.32	0.76	0.87	2	0.015
IL17	1070	ENSG0000011394	CLDN16	0.06	-0.03	0.62	2	2	0.043
IL17	1070	ENSG0000015313	CLGN	0.04	-0.03	0.92	0.82	0.84	1.30E-24
IL17	1070	ENSG0000012897	CLN6	0.47	0.24	0.84	1.20E-07	0.031	2.40E-24

IL17	1070	ENSG0000009285	CLSPN	0.57	0.06	1.46	3.30E-10	0.67	3.50E-69
IL17	1070	ENSG0000013918	CLSTN3	0.16	0.1	0.8	0.033	0.23	3.10E-37
IL17	1070	ENSG0000013236	CLUH	0.39	0.17	0.73	6.90E-12	0.016	3.00E-43
IL17	1070	ENSG0000014458	CNOT9	0.34	0.16	0.76	1.80E-10	0.019	3.60E-52
IL17	1070	ENSG0000024268	CNTF	0.16	0.27	0.84	0.71	2	0.0079
IL17	1070	ENSG0000010879	CNTNAP1	0.3	0.22	0.82	1.20E-07	0.0023	6.00E-54
IL17	1070	ENSG0000008243	COBLL1	-0.1	-0.15	0.82	0.75	2	3.80E-06
IL17	1070	ENSG0000020424	COL11A2	0.33	0.15	0.75	0.18	2	1.20E-04
IL17	1070	ENSG0000008229	COL19A1	0.59	0.09	1.58	0.059	2	1.70E-08
IL17	1070	ENSG0000019673	COL27A1	0.31	0.16	0.8	4.60E-06	0.062	7.50E-38
IL17	1070	ENSG0000008105	COL4A4	0.38	0.05	1.5	0.29	2	4.80E-06
IL17	1070	ENSG0000017181	COL8A2	0.22	-0.26	0.63	0.058	0.043	1.80E-11
IL17	1070	ENSG0000010287	CORO1A	0.31	0.23	0.68	0.26	2	0.0016
IL17	1070	ENSG0000010318	COTL1	0.44	0.07	0.9	3.40E-19	0.27	8.30E-79
IL17	1070	ENSG0000012851	CPA4	0.15	0.31	0.79	0.0057	2.40E-08	5.50E-64
IL17	1070	ENSG0000013911	CPNE8	-0.08	-0.08	0.8	2	2	0.018
IL17	1070	ENSG0000012420	CSE1L	0.24	-0.03	0.92	5.10E-04	0.74	3.80E-53
IL17	1070	ENSG0000018437	CSF1	0.56	0.11	1.37	5.90E-19	0.21	1.30E-117
IL17	1070	ENSG0000016440	CSF2	0.31	0.12	2.08	0.23	2	2.80E-07
IL17	1070	ENSG0000017179	CTPS1	0.42	0.25	1.05	1.20E-09	0.0039	3.20E-61
IL17	1070	ENSG0000010809	CUL2	0.09	-0.09	0.76	0.27	0.3	5.00E-37
IL17	1070	ENSG0000008104	CXCL2	0.39	0.24	2.41	0.25	2	9.60E-17
IL17	1070	ENSG0000016373	CXCL3	-0.16	-0.06	1.4	0.69	2	1.10E-06
IL17	1070	ENSG0000016373	CXCL5	0.32	0.22	3.36	0.11	2	2.30E-121
IL17	1070	ENSG0000016942	CXCL8	-0.61	0.48	1.64	2.90E-04	2	5.80E-32
IL17	1070	ENSG0000017211	CYCS	0.01	-0.05	0.81	0.91	0.59	1.10E-40
IL17	1070	ENSG0000008379	CYLD	0.37	-0.14	0.71	7.20E-10	0.078	2.20E-36
IL17	1070	ENSG0000000313	CYP26B1	-0.01	0.32	0.89	0.98	2	0.0054
IL17	1070	ENSG0000011101	CYP27B1	0.09	-0.06	0.7	0.87	2	0.034
IL17	1070	ENSG0000015220	CYSLTR2	0.24	0	0.68	0.56	2	0.031
IL17	1070	ENSG0000000663	DBF4	0.43	-0.03	0.88	3.40E-05	0.85	3.70E-21
IL17	1070	ENSG0000023548	DBF4P1	0.1	0.18	0.6	2	2	0.048
IL17	1070	ENSG0000016493	DCAF13	0.08	-0.09	0.62	0.32	0.26	1.40E-28
IL17	1070	ENSG0000011865	DCLRE1B	0.22	0.16	0.65	0.024	0.15	4.00E-16
IL17	1070	ENSG0000017995	DCTPP1	0.35	0.18	0.67	7.40E-05	0.089	9.80E-17
IL17	1070	ENSG0000016549	DDIAS	0.51	0.05	1.32	0.00093	2	4.80E-24
IL17	1070	ENSG0000001357	DDX11	0.51	0.21	0.77	2.00E-06	0.12	1.10E-14
IL17	1070	ENSG0000008820	DDX18	0.01	-0.1	0.71	0.91	0.21	3.30E-36
IL17	1070	ENSG0000016573	DDX21	0.1	-0.1	1.03	0.11	0.14	5.50E-104
IL17	1070	ENSG0000012313	DDX39A	0.34	0.17	0.71	5.00E-08	0.029	3.30E-35
IL17	1070	ENSG0000002452	DEPDC1	0.44	0.03	0.89	2.20E-04	0.86	1.20E-16
IL17	1070	ENSG0000003549	DEPDC1B	0.57	0.1	0.99	1.10E-04	2	4.20E-14
IL17	1070	ENSG0000012169	DEPDC7	0.38	-0.25	0.68	0.024	2	3.50E-06
IL17	1070	ENSG0000022871	DHFR	0.43	0.09	0.67	8.90E-08	0.44	1.90E-19
IL17	1070	ENSG0000010296	DHODH	0.29	0.24	0.73	0.094	2	1.80E-07
IL17	1070	ENSG0000015099	DHX37	0.26	0.15	0.73	2.90E-04	0.083	8.70E-31
IL17	1070	ENSG0000013973	DIAPH3	0.25	0.06	0.94	8.60E-05	0.47	3.70E-62
IL17	1070	ENSG0000021144	DIO2	0.13	0.13	1.7	0.73	2	1.40E-16
IL17	1070	ENSG0000019740	DIO3	0.12	0.11	0.83	2	2	0.0093
IL17	1070	ENSG0000013082	DKC1	0.32	0.09	0.89	5.70E-06	0.3	2.50E-45
IL17	1070	ENSG0000009114	DLD	0.1	-0.12	0.61	0.21	0.16	3.80E-24
IL17	1070	ENSG0000012678	DLGAP5	0.57	-0.01	0.72	5.80E-08	0.97	8.50E-13
IL17	1070	ENSG0000010020	DMC1	0.24	0.04	0.64	0.5	2	0.014
IL17	1070	ENSG0000013834	DNA2	0.18	-0.17	0.8	0.35	2	6.20E-10
IL17	1070	ENSG0000018584	DNAH14	0.16	-0.07	0.61	0.46	2	4.90E-05
IL17	1070	ENSG0000009052	DNAJB11	0.1	0.02	0.61	0.2	0.84	1.10E-28
IL17	1070	ENSG0000012859	DNAJB9	-0.01	-0.11	1.09	0.95	0.28	2.30E-59
IL17	1070	ENSG0000007723	DNAJC10	-0.06	-0.1	0.69	0.46	0.13	6.70E-44
IL17	1070	ENSG0000010582	DNAJC2	0.14	-0.01	0.67	0.13	0.95	1.00E-20
IL17	1070	ENSG0000010258	DNAJC3	-0.09	-0.13	0.61	0.15	0.043	1.20E-37
IL17	1070	ENSG0000021355	DNAJC9	0.3	0.1	0.65	4.50E-05	0.29	1.20E-22
IL17	1070	ENSG0000013081	DNMT1	0.42	0.19	0.79	1.10E-22	7.20E-04	1.90E-83
IL17	1070	ENSG0000013590	DOCK10	0.55	-0.18	1.03	1.00E-11	0.085	1.50E-41
IL17	1070	ENSG0000015914	DONSON	0.39	0.13	0.77	6.60E-06	0.23	1.20E-22
IL17	1070	ENSG0000010488	DOT1L	0.27	0.19	0.63	6.50E-06	0.01	3.40E-32

IL17	1070	ENSG0000001133	DPF1	0.49	0.24	0.71	0.0024	2	8.40E-07
IL17	1070	ENSG0000013276	DPH2	0.27	0.27	0.62	0.018	0.038	2.00E-10
IL17	1070	ENSG0000017385	DPY19L1	0.02	-0.09	0.96	0.81	0.27	8.90E-72
IL17	1070	ENSG0000013604	DRAM1	0.18	0.03	0.98	3.30E-04	0.68	1.80E-112
IL17	1070	ENSG0000013698	DSCC1	0.38	0.18	1.09	0.011	2	1.90E-19
IL17	1070	ENSG0000014963	DSN1	0.27	0.02	0.75	0.009	0.88	6.80E-18
IL17	1070	ENSG0000009669	DSP	0.14	-0.06	0.82	0.66	2	4.10E-05
IL17	1070	ENSG0000014347	DTL	0.49	0.03	1.22	1.40E-07	0.87	8.30E-47
IL17	1070	ENSG0000014199	DUS3L	0.22	0.25	0.66	0.028	0.023	1.40E-15
IL17	1070	ENSG0000014350	DUSP10	0.14	0.08	0.73	0.13	0.42	3.10E-26
IL17	1070	ENSG0000013816	DUSP5	0.33	0.13	0.69	6.60E-06	0.17	1.00E-25
IL17	1070	ENSG0000010141	E2F1	0.45	0.21	1.18	5.90E-07	0.059	8.20E-49
IL17	1070	ENSG0000011224	E2F3	0.21	-0.01	0.6	0.014	0.94	1.50E-16
IL17	1070	ENSG0000013374	E2F5	0.11	0	0.66	0.58	2	1.10E-07
IL17	1070	ENSG0000014508	EAF2	0.21	0.57	0.7	0.63	2	0.025
IL17	1070	ENSG0000011739	EBNA1BP2	0.11	0.07	0.64	0.13	0.41	2.90E-30
IL17	1070	ENSG0000014519	ECE2	0.35	0.22	0.84	0.19	2	5.30E-05
IL17	1070	ENSG0000011434	ECT2	0.44	-0.01	0.97	8.20E-07	0.96	2.00E-32
IL17	1070	ENSG0000013616	EDNRB	0.1	-0.16	1.43	0.5	0.24	1.10E-56
IL17	1070	ENSG0000012480	EEF1E1	0.05	-0.07	0.59	0.83	2	2.80E-06
IL17	1070	ENSG0000014263	EFHD2	0.4	0.07	0.76	1.50E-13	0.34	8.10E-50
IL17	1070	ENSG0000012952	EGLN3	0.55	-0.24	1.21	0.11	2	1.50E-04
IL17	1070	ENSG0000013537	EHF	0.47	0.32	1.74	0.0082	2	3.40E-07
IL17	1070	ENSG0000013250	EIF5A	0.29	0.06	0.76	1.10E-08	0.4	2.40E-59
IL17	1070	ENSG0000016343	ELF3	0.25	0.3	1.63	2	2	8.90E-06
IL17	1070	ENSG0000017052	ELOVL6	0.13	-0.1	0.6	0.07	0.2	2.80E-24
IL17	1070	ENSG0000015492	EME1	0.5	0.2	0.83	4.70E-04	2	1.60E-10
IL17	1070	ENSG0000016552	EML5	0.28	-0.26	1.04	0.49	2	7.30E-04
IL17	1070	ENSG0000007480	ENO1	0.35	0.17	0.73	2.10E-14	0.0032	9.00E-63
IL17	1070	ENSG0000015492	EPHB1	0.32	0.12	1.35	0.31	2	8.00E-09
IL17	1070	ENSG0000013310	EPSTI1	0.25	0.04	1.01	0.11	0.86	3.70E-17
IL17	1070	ENSG0000016430	ERAP1	0.39	-0.03	0.81	3.40E-13	0.75	1.00E-55
IL17	1070	ENSG0000016430	ERAP2	0.4	-0.15	0.59	0.089	2	0.0038
IL17	1070	ENSG0000012488	EREG	0.21	-0.06	0.8	0.051	0.67	2.60E-20
IL17	1070	ENSG0000017860	ERN1	0.24	0.09	1.11	0.018	0.49	2.40E-41
IL17	1070	ENSG0000011628	ERRFI1	0.12	0.13	0.87	0.068	0.079	5.30E-63
IL17	1070	ENSG0000008904	ESF1	-0.07	-0.09	0.62	0.57	0.44	1.40E-15
IL17	1070	ENSG0000016428	ESM1	-0.01	-0.4	1.41	0.96	2	3.50E-07
IL17	1070	ENSG0000013495	ETS1	0.2	-0.17	0.8	5.90E-04	0.019	2.00E-55
IL17	1070	ENSG0000017583	ETV4	0.5	0.23	0.71	7.80E-13	0.01	9.90E-27
IL17	1070	ENSG0000016697	EVA1C	0.16	0.06	0.64	0.67	2	0.012
IL17	1070	ENSG0000012686	EVI2A	0.55	-0.23	0.69	0.12	2	0.031
IL17	1070	ENSG0000018586	EVI2B	0.39	0.11	0.59	2	2	0.02
IL17	1070	ENSG0000017437	EXO1	0.39	0	1.14	4.90E-04	1	3.40E-32
IL17	1070	ENSG0000013819	EXOC6	0.05	-0.09	0.82	0.76	0.5	6.90E-23
IL17	1070	ENSG0000013071	EXOSC2	0.28	0.19	0.63	0.0012	0.057	4.10E-17
IL17	1070	ENSG0000007734	EXOSC5	0.47	0.37	0.98	0.0012	2	1.40E-14
IL17	1070	ENSG0000012069	EXOSC8	0.08	0.02	0.63	0.37	0.89	4.20E-25
IL17	1070	ENSG0000012373	EXOSC9	0.19	0.04	0.6	0.019	0.68	1.10E-19
IL17	1070	ENSG0000010646	EZH2	0.49	0.08	0.81	9.10E-10	0.49	1.90E-27
IL17	1070	ENSG0000016468	FABP5	0.56	-0.13	0.8	7.40E-05	2	1.30E-09
IL17	1070	ENSG0000016912	FAM110B	0.47	-0.03	0.69	1.10E-04	0.88	5.50E-10
IL17	1070	ENSG0000016680	FAM111A	0.29	-0.01	0.67	5.30E-05	0.96	3.40E-25
IL17	1070	ENSG0000018905	FAM111B	0.38	-0.04	1.81	0.0017	0.81	2.40E-76
IL17	1070	ENSG0000018891	FAM196A	0.22	0.17	0.65	0.45	2	0.0012
IL17	1070	ENSG0000013943	FAM222A	-0.03	-0.07	0.71	0.94	2	0.008
IL17	1070	ENSG0000018882	FAM26F	-0.05	-0.15	0.83	0.9	2	0.0051
IL17	1070	ENSG0000018861	FAM72B	0.29	0.17	0.61	0.41	2	0.022
IL17	1070	ENSG0000022182	FANCG	0.39	0.22	0.6	5.80E-07	0.026	2.30E-16
IL17	1070	ENSG0000014052	FANCI	0.48	0.05	1.03	6.10E-09	0.67	1.10E-41
IL17	1070	ENSG0000018779	FANCM	0.16	0.05	0.75	0.4	2	6.60E-09
IL17	1070	ENSG0000011612	FARSB	0.28	0.08	0.88	2.50E-05	0.35	1.50E-51
IL17	1070	ENSG0000002610	FAS	0.17	-0.12	0.78	0.1	0.29	1.20E-21
IL17	1070	ENSG0000012427	FASTKD3	-0.04	0	0.6	0.87	2	2.70E-06
IL17	1070	ENSG0000017897	FBXO34	0.53	0.03	0.65	1.00E-18	0.78	2.50E-28

IL17	1070	ENSG0000017401	FBXO45	0.16	-0.01	0.68	0.13	0.94	7.40E-18
IL17	1070	ENSG0000011202	FBXO5	0.38	0.14	0.89	5.40E-05	0.26	5.80E-26
IL17	1070	ENSG0000016849	FEN1	0.5	0.19	1.35	1.90E-06	0.15	5.00E-46
IL17	1070	ENSG0000010131	FERMT1	0.27	0.07	0.59	0.073	2	1.20E-06
IL17	1070	ENSG0000018026	FGD6	0.53	-0.18	2.02	9.70E-08	0.18	2.30E-118
IL17	1070	ENSG0000019885	FICD	0.13	0.11	1.09	0.36	0.46	1.10E-31
IL17	1070	ENSG0000013243	FIGNL1	0.36	-0.11	0.96	2.80E-04	0.38	2.30E-28
IL17	1070	ENSG0000016838	FILIP1L	0.4	-0.09	0.98	1.20E-10	0.27	8.80E-63
IL17	1070	ENSG0000017943	FJX1	0.26	0.24	0.81	0.0028	0.015	2.00E-28
IL17	1070	ENSG0000013428	FKBP11	0.26	0.14	0.85	3.80E-05	0.072	2.00E-54
IL17	1070	ENSG0000000447	FKBP4	0.19	0.1	0.66	0.0026	0.18	3.30E-36
IL17	1070	ENSG0000015170	FLI1	0.03	-0.1	0.72	0.95	2	9.90E-04
IL17	1070	ENSG0000018507	FLRT2	0.33	-0.05	1.46	1.90E-04	0.7	5.60E-79
IL17	1070	ENSG0000010275	FLT1	-0.5	0.05	1.1	0.005	2	4.60E-14
IL17	1070	ENSG0000024890	FMN1	-0.51	-0.18	0.69	6.90E-10	0.091	8.80E-19
IL17	1070	ENSG0000015147	FRMD4A	0.35	0	0.59	3.30E-13	0.99	5.20E-38
IL17	1070	ENSG0000019637	FUT4	0.09	0.01	0.86	0.47	0.93	2.00E-30
IL17	1070	ENSG0000012368	G0S2	-0.37	0.1	0.86	0.0025	0.5	2.60E-18
IL17	1070	ENSG0000014590	G3BP1	0.12	0.04	0.67	0.0047	0.5	4.00E-73
IL17	1070	ENSG0000015183	GABRA2	0.45	-0.08	0.76	1.30E-06	0.54	9.10E-19
IL17	1070	ENSG0000026808	GABRQ	0.33	0.35	1.34	0.33	2	2.70E-05
IL17	1070	ENSG0000011671	GADD45A	0.37	0.08	1.34	6.10E-06	0.46	5.10E-74
IL17	1070	ENSG0000014142	GALNT1	0.23	-0.13	0.83	1.00E-04	0.069	9.80E-55
IL17	1070	ENSG0000011032	GALNT18	0.51	0.12	1.06	0.0076	2	8.10E-11
IL17	1070	ENSG0000011533	GALNT3	0.23	-0.15	0.62	0.4	2	0.002
IL17	1070	ENSG0000014144	GATA6	0.23	0.1	0.81	0.0023	0.25	2.60E-37
IL17	1070	ENSG0000016265	GBP4	0.17	0.21	0.64	0.66	2	0.009
IL17	1070	ENSG0000017829	GEN1	0.49	0.02	0.91	5.40E-06	0.91	2.30E-20
IL17	1070	ENSG0000013145	GFPT2	0.22	0.18	0.84	0.0036	0.04	3.60E-40
IL17	1070	ENSG0000015189	GFRA1	-0.34	0.16	1.12	2.30E-04	0.15	1.10E-44
IL17	1070	ENSG0000000662	GGCT	0.19	0.1	0.61	0.12	0.46	5.40E-11
IL17	1070	ENSG0000013756	GGH	0.26	-0.18	0.7	0.0085	0.12	3.50E-16
IL17	1070	ENSG0000010100	GINS1	0.51	0.11	1.04	1.50E-08	0.4	1.10E-36
IL17	1070	ENSG0000013115	GINS2	0.51	0.13	1.05	4.40E-08	0.29	4.50E-35
IL17	1070	ENSG0000018193	GINS3	0.57	0.23	1.12	2.90E-04	2	7.00E-16
IL17	1070	ENSG0000015266	GJA1	0.47	-0.1	1.13	3.20E-20	0.16	4.30E-118
IL17	1070	ENSG0000018315	GJD3	0.47	0.08	0.74	0.046	2	2.70E-04
IL17	1070	ENSG0000017844	GLDC	0.31	0.38	0.71	0.39	2	0.02
IL17	1070	ENSG0000010973	GLRB	0	-0.02	0.73	0.99	0.93	1.50E-11
IL17	1070	ENSG0000011231	GMNN	0.32	0	0.95	6.70E-04	0.98	6.30E-32
IL17	1070	ENSG0000017354	GMPPB	0.39	0.2	0.79	3.20E-08	0.021	1.00E-33
IL17	1070	ENSG0000012826	GNAZ	0.56	0.13	0.67	0.035	2	0.0057
IL17	1070	ENSG0000013469	GNL2	0.06	-0.11	0.61	0.5	0.18	2.60E-23
IL17	1070	ENSG0000016393	GNL3	0.1	-0.04	0.75	0.077	0.55	8.10E-62
IL17	1070	ENSG0000011167	GNPTAB	-0.01	-0.12	0.69	0.96	0.14	4.60E-32
IL17	1070	ENSG0000018628	GPAT2	0.58	0.29	0.66	0.083	2	0.031
IL17	1070	ENSG0000016081	GPATCH4	0.22	-0.04	0.96	0.084	0.83	1.00E-21
IL17	1070	ENSG0000017172	GPHN	0.42	0.15	0.64	1.70E-04	0.3	6.90E-10
IL17	1070	ENSG0000018367	GPR1	0.18	-0.12	0.8	0.62	2	4.20E-04
IL17	1070	ENSG0000016607	GPR176	0.28	0.03	0.62	1.60E-08	0.68	1.00E-41
IL17	1070	ENSG0000011971	GPR68	0.39	-0.09	1.15	0.16	2	9.00E-08
IL17	1070	ENSG0000016460	GPR85	0.44	-0.13	0.66	0.061	2	0.0012
IL17	1070	ENSG0000001358	GPRC5A	0.27	0.44	0.63	0.014	1.50E-04	6.30E-12
IL17	1070	ENSG0000010951	GRPEL1	0.12	0.1	0.59	0.19	0.34	7.80E-18
IL17	1070	ENSG0000010544	GRWD1	0.29	0.21	0.77	3.60E-06	0.0065	6.00E-42
IL17	1070	ENSG0000010793	GTPBP4	0.02	-0.04	0.73	0.87	0.68	5.30E-34
IL17	1070	ENSG0000015123	GXYLT1	-0.07	-0.25	0.69	0.5	0.0096	9.50E-22
IL17	1070	ENSG0000017096	HAS2	-0.49	0.01	0.61	2.50E-05	0.96	4.80E-09
IL17	1070	ENSG0000012870	HAT1	0.04	-0.1	0.71	0.73	0.37	6.10E-22
IL17	1070	ENSG0000014787	HAUS6	0.12	-0.13	0.72	0.3	0.26	4.80E-19
IL17	1070	ENSG0000013135	HAUS8	0.54	0.15	0.83	4.90E-07	0.28	4.40E-17
IL17	1070	ENSG0000022360	HBD	0.17	0.38	1	0.7	2	4.90E-04
IL17	1070	ENSG0000016650	HDGFL3	0.08	-0.09	0.64	0.23	0.18	4.40E-42
IL17	1070	ENSG0000015539	HEATR3	0.14	0.07	0.61	0.21	0.59	2.40E-13
IL17	1070	ENSG0000017370	HEG1	0.07	0.09	0.84	0.33	0.21	2.60E-62

IL17	1070	ENSG0000011996	HELLS	0.47	-0.04	1.1	1.20E-05	0.83	1.10E-29
IL17	1070	ENSG0000016547	HEPACAM	0.46	-0.03	1.4	0.18	2	1.10E-05
IL17	1070	ENSG0000011431	HES1	0.45	-0.33	0.96	0.13	2	1.10E-04
IL17	1070	ENSG0000018829	HES4	0.33	0.24	0.95	0.16	2	1.80E-07
IL17	1070	ENSG0000017911	HES7	0.13	-0.15	0.79	0.79	2	0.014
IL17	1070	ENSG0000001999	HGF	0.46	0.06	0.95	8.60E-12	0.54	6.20E-50
IL17	1070	ENSG0000015280	HHEX	0.13	0.01	0.7	0.23	0.94	5.50E-19
IL17	1070	ENSG0000017737	HIC1	0.45	0.1	0.64	8.40E-16	0.16	7.40E-33
IL17	1070	ENSG0000012712	HIVEP3	-0.01	0.23	1.03	0.96	0.028	4.30E-42
IL17	1070	ENSG0000020463	HLA-G	0.43	0.31	0.6	0.098	2	0.0074
IL17	1070	ENSG0000007257	HMMR	0.46	0.01	0.87	9.40E-05	0.98	2.20E-16
IL17	1070	ENSG0000022188	HMSD	0.18	-0.07	0.96	0.62	2	4.60E-05
IL17	1070	ENSG0000019745	HNRNPAB	0.28	0.13	0.72	1.30E-05	0.11	2.50E-35
IL17	1070	ENSG0000018660	HPDL	-0.01	0.04	0.79	0.97	2	0.0011
IL17	1070	ENSG0000016845	HR	0.59	0.03	0.85	0.075	2	0.0043
IL17	1070	ENSG0000015393	HS2ST1	0.13	0	1.11	0.036	0.97	3.50E-107
IL17	1070	ENSG0000008082	HSP90AA1	0.15	-0.1	0.76	0.012	0.17	4.40E-49
IL17	1070	ENSG0000016659	HSP90B1	-0.02	-0.08	0.68	0.86	0.29	2.00E-40
IL17	1070	ENSG0000018752	HSPA14	0.11	-0.12	0.68	0.54	2	3.10E-09
IL17	1070	ENSG0000016407	HSPA4L	0.36	-0.06	1.03	0.019	2	2.40E-16
IL17	1070	ENSG0000010997	HSPA8	0.28	0.12	0.66	5.00E-14	0.0098	9.40E-78
IL17	1070	ENSG0000014438	HSPD1	0.16	-0.05	0.9	0.0067	0.49	7.40E-70
IL17	1070	ENSG0000011554	HSPE1	0.28	0.09	0.83	3.40E-04	0.39	4.90E-33
IL17	1070	ENSG0000012069	HSPH1	0.29	-0.11	0.88	3.90E-06	0.16	5.50E-54
IL17	1070	ENSG0000014942	HYOU1	0.2	0.16	0.93	0.0011	0.03	9.80E-67
IL17	1070	ENSG0000009033	ICAM1	0.11	0.04	2.23	0.36	0.73	6.70E-187
IL17	1070	ENSG0000011731	ID3	0.16	-0.03	1.04	0.54	2	4.70E-10
IL17	1070	ENSG0000016641	IDH3A	0.18	0.06	0.62	0.024	0.55	2.90E-21
IL17	1070	ENSG0000013796	IFI44	0.58	0.14	0.62	1.40E-04	2	2.20E-05
IL17	1070	ENSG0000015911	IFNAR2	0.23	0	0.93	0.018	1	1.90E-32
IL17	1070	ENSG0000021470	IFRD2	0.25	0.25	0.8	6.40E-04	0.0038	3.60E-37
IL17	1070	ENSG0000008555	IGSF9	0.5	-0.05	0.65	0.092	2	0.022
IL17	1070	ENSG0000026352	IKBKE	0.55	0.23	0.73	4.00E-09	0.055	2.10E-16
IL17	1070	ENSG0000016881	IL12A	0.26	0.4	0.72	0.33	2	2.20E-04
IL17	1070	ENSG0000016413	IL15	0.23	0.05	0.68	0.15	2	4.30E-08
IL17	1070	ENSG0000018826	IL17REL	0.3	0.02	1.37	2	2	1.80E-04
IL17	1070	ENSG0000015078	IL18	0.33	0.07	0.66	2	2	0.038
IL17	1070	ENSG0000011560	IL18R1	-0.01	-0.09	1.18	0.96	0.56	4.10E-33
IL17	1070	ENSG0000011560	IL18RAP	0.4	0.31	0.96	0.28	2	0.0026
IL17	1070	ENSG0000011500	IL1A	-0.06	-0.48	0.84	0.91	2	0.011
IL17	1070	ENSG0000011560	IL1RL1	0.28	0.05	1.17	0.43	2	3.80E-06
IL17	1070	ENSG0000007723	IL4R	0.32	0.1	0.65	2.20E-08	0.17	5.90E-34
IL17	1070	ENSG0000013624	IL6	-0.3	0.8	1.7	0.23	2	3.50E-23
IL17	1070	ENSG0000016868	IL7R	-0.08	0	0.61	0.3	0.97	1.40E-28
IL17	1070	ENSG0000013671	IMP4	0.19	0.11	0.72	0.0031	0.14	3.30E-41
IL17	1070	ENSG0000023213	IMPDH1P10	0.26	0.07	0.6	2	2	0.037
IL17	1070	ENSG0000014950	INCENP	0.58	0.19	0.71	1.20E-12	0.074	2.20E-19
IL17	1070	ENSG0000012264	INHBA	-0.41	-0.19	0.72	1.60E-09	0.028	6.50E-30
IL17	1070	ENSG0000015339	INO80C	0.52	0.04	0.87	2.70E-04	2	1.00E-11
IL17	1070	ENSG0000016535	INTS6L	-0.02	-0.04	0.71	0.91	0.83	8.00E-14
IL17	1070	ENSG0000019649	IPO4	0.4	0.22	0.69	0.17	2	0.004
IL17	1070	ENSG0000020533	IPO7	0.1	-0.11	0.66	0.17	0.14	2.90E-35
IL17	1070	ENSG0000017462	IQCK	0.23	-0.11	0.59	0.17	2	7.00E-06
IL17	1070	ENSG0000013407	IRAK2	0.57	-0.08	1.43	5.00E-05	2	5.60E-32
IL17	1070	ENSG0000016417	ITGA2	0.58	-0.12	1.69	1.60E-13	0.26	6.70E-117
IL17	1070	ENSG0000011523	ITGA4	0.42	0.06	0.88	1.20E-05	0.64	1.00E-24
IL17	1070	ENSG0000009140	ITGA6	0.24	0	0.81	9.50E-04	0.98	1.30E-36
IL17	1070	ENSG0000013844	ITGAV	0.51	-0.16	1.06	4.80E-23	0.017	2.40E-100
IL17	1070	ENSG0000012310	ITPR2	0.4	-0.16	1.1	1.50E-04	0.22	7.70E-31
IL17	1070	ENSG0000010563	JAK3	0.56	-0.02	1.71	1.60E-07	0.92	7.30E-76
IL17	1070	ENSG0000020605	JPT2	0.47	0.14	0.85	8.70E-13	0.11	5.10E-43
IL17	1070	ENSG0000017122	JUNB	0.34	0.09	0.87	1.10E-07	0.32	4.90E-48
IL17	1070	ENSG0000017659	KBTBD11	-0.03	0.34	0.61	0.95	2	0.0047
IL17	1070	ENSG0000016337	KBTBD8	-0.17	-0.39	0.59	0.57	2	0.0036
IL17	1070	ENSG0000017727	KCNA3	0.48	0.37	1.45	0.025	2	1.80E-16

IL17	1070	ENSG0000008248	KCNK2	0.48	0.3	0.89	4.60E-05	0.036	8.30E-17
IL17	1070	ENSG0000018576	KCNQ5	0.25	-0.25	1.68	0.48	2	1.80E-12
IL17	1070	ENSG0000017074	KCNS3	0.57	0.1	0.92	0.098	2	0.0029
IL17	1070	ENSG0000023620	KDM4A-AS1	0.35	-0.03	0.72	0.36	2	0.024
IL17	1070	ENSG0000012805	KDR	-0.42	0.15	0.85	7.50E-07	0.14	1.20E-29
IL17	1070	ENSG0000010089	KIAA0391	0.31	0.06	0.64	0.049	2	1.10E-06
IL17	1070	ENSG0000010926	KIAA1211	0.55	-0.07	0.72	9.00E-06	0.69	5.80E-10
IL17	1070	ENSG0000016350	KIAA1524	0.5	0.03	1.21	2.30E-07	0.84	3.00E-43
IL17	1070	ENSG0000013816	KIF11	0.39	-0.03	0.8	0.0037	0.9	1.40E-11
IL17	1070	ENSG0000011819	KIF14	0.56	0.1	0.98	5.30E-06	0.54	1.20E-17
IL17	1070	ENSG0000016380	KIF15	0.49	-0.06	0.9	2.10E-07	0.69	1.10E-24
IL17	1070	ENSG0000012162	KIF18A	0.41	0	0.6	0.0028	0.99	1.30E-06
IL17	1070	ENSG0000013029	KIF1A	-0.07	0.17	0.82	0.88	2	0.015
IL17	1070	ENSG0000013818	KIF20B	0.54	0.07	0.98	5.10E-10	0.56	9.00E-33
IL17	1070	ENSG0000013911	KIF21A	0.19	-0.12	0.71	0.54	2	9.00E-04
IL17	1070	ENSG0000011685	KIF21B	0.12	0.13	0.8	0.79	2	0.0017
IL17	1070	ENSG0000013780	KIF23	0.43	0.14	0.89	2.60E-08	0.16	4.00E-35
IL17	1070	ENSG0000004913	KITLG	0.06	-0.07	0.71	0.57	0.43	4.70E-29
IL17	1070	ENSG0000017187	KLF17	0.27	0.01	0.75	2	2	0.016
IL17	1070	ENSG0000021316	KLHL23	0.07	-0.04	0.77	0.74	2	7.20E-10
IL17	1070	ENSG0000013781	KNL1	0.54	-0.02	0.82	9.60E-05	0.94	1.00E-10
IL17	1070	ENSG0000018444	KNTC1	0.46	0.02	1.01	1.30E-08	0.88	2.10E-41
IL17	1070	ENSG0000018248	KPNA2	0.53	0.14	0.78	6.00E-13	0.15	6.90E-29
IL17	1070	ENSG0000017134	KRT15	0.64	0.25	0.9	2	2	0.0043
IL17	1070	ENSG0000011105	KRT18	0.56	0.02	0.95	1.60E-13	0.91	4.00E-39
IL17	1070	ENSG0000011591	KYNU	0.52	-0.01	1.41	0.08	2	6.30E-08
IL17	1070	ENSG0000019687	LAMB3	0.41	0.1	0.71	0.021	2	2.90E-06
IL17	1070	ENSG0000000254	LAP3	0.3	0.07	0.93	9.80E-05	0.47	3.00E-43
IL17	1070	ENSG0000015301	LGI2	0.53	0.2	1.81	0.062	2	1.50E-07
IL17	1070	ENSG0000014568	LHFPL2	0.18	0.1	0.68	0.034	0.33	6.60E-23
IL17	1070	ENSG0000012834	LIF	0.51	0.37	1	3.00E-10	1.70E-04	5.50E-40
IL17	1070	ENSG0000011359	LIFR	0.07	-0.15	0.87	0.43	0.084	4.80E-43
IL17	1070	ENSG0000018381	LIN9	0.24	-0.08	0.81	0.2	2	3.90E-09
IL17	1070	ENSG0000023041	LINC00595	0.09	0.15	0.63	0.87	2	0.044
IL17	1070	ENSG0000023129	LINC00704	0.35	0.12	1.11	0.25	2	4.50E-04
IL17	1070	ENSG0000020505	LINC01121	0.23	0.27	0.85	2	2	0.0076
IL17	1070	ENSG0000022956	LINC01204	0.26	-0.12	0.82	0.5	2	0.0082
IL17	1070	ENSG0000022989	LINC01315	0.62	0.22	0.63	2	2	0.029
IL17	1070	ENSG0000023083	LINC01614	0.42	0.47	0.61	2	2	0.046
IL17	1070	ENSG0000022685	LINC02051	0.52	0.01	0.6	2	2	0.016
IL17	1070	ENSG0000017661	LMNB2	0.41	0.19	0.61	6.20E-12	0.014	2.80E-26
IL17	1070	ENSG0000015719	LRP8	0.3	0.16	1.06	2.50E-04	0.1	8.20E-51
IL17	1070	ENSG0000016550	LRR1	0.37	0.03	0.98	2.90E-04	0.86	9.40E-29
IL17	1070	ENSG0000016968	LRRC45	0.36	0.31	0.61	5.10E-04	0.013	1.80E-10
IL17	1070	ENSG0000010882	LRRC59	0.24	0.07	0.59	1.60E-07	0.23	1.90E-42
IL17	1070	ENSG0000019714	LRRC8B	0.31	-0.13	1.08	0.017	0.41	6.50E-25
IL17	1070	ENSG0000017148	LRRC8C	0.2	-0.17	0.73	0.024	0.095	6.00E-23
IL17	1070	ENSG0000017149	LRRC8D	0.31	0.15	0.85	2.70E-05	0.11	4.60E-38
IL17	1070	ENSG0000013552	LTV1	0.02	-0.14	0.72	0.93	0.27	1.90E-17
IL17	1070	ENSG0000013932	LUM	0.05	-0.03	0.65	0.57	0.73	2.30E-33
IL17	1070	ENSG0000014522	LYAR	0.15	-0.03	0.91	0.28	0.88	7.70E-21
IL17	1070	ENSG0000025408	LYN	0.21	0.06	0.67	0.27	2	1.40E-06
IL17	1070	ENSG0000018712	LYPD6	0.39	0.33	0.92	0.11	2	2.60E-06
IL17	1070	ENSG0000014028	LYSMD2	-0.16	-0.09	0.59	0.48	2	1.10E-04
IL17	1070	ENSG0000016410	MAD2L1	0.41	0	1	4.10E-06	0.99	1.20E-34
IL17	1070	ENSG0000019804	MAK16	0.12	0.1	0.78	0.19	0.29	9.90E-34
IL17	1070	ENSG0000014505	MANF	0.25	0.14	0.76	1.40E-04	0.07	5.50E-39
IL17	1070	ENSG0000013552	MAP7	0.26	0.09	0.84	0.5	2	0.0031
IL17	1070	ENSG0000024762	MARS2	0.32	0.34	1.04	0.068	2	6.70E-14
IL17	1070	ENSG0000012053	MASTL	0.21	0.02	0.88	0.07	0.9	3.50E-22
IL17	1070	ENSG0000016890	MAT2A	0.3	0.13	0.65	4.50E-20	0.0031	3.30E-94
IL17	1070	ENSG0000016443	MB21D1	0.34	0.07	1.05	0.01	2	5.20E-22
IL17	1070	ENSG0000007311	MCM2	0.56	0.19	1.02	1.70E-19	0.022	2.70E-66
IL17	1070	ENSG0000011211	MCM3	0.49	0.19	1.05	5.10E-15	0.019	4.50E-71
IL17	1070	ENSG0000010473	MCM4	0.49	0.16	1.01	3.30E-17	0.039	3.80E-74

IL17	1070	ENSG0000010029	MCM5	0.53	0.16	0.99	5.70E-17	0.051	2.20E-61
IL17	1070	ENSG0000007600	MCM6	0.18	0.08	0.83	0.0081	0.32	2.40E-46
IL17	1070	ENSG0000016650	MCM7	0.51	0.22	1.04	2.90E-14	0.0097	6.60E-59
IL17	1070	ENSG0000012588	MCM8	0.35	-0.05	1.24	2.60E-04	0.74	8.80E-51
IL17	1070	ENSG0000017547	MCTP1	0.47	0	1.12	0.12	2	7.90E-06
IL17	1070	ENSG0000011215	MDN1	0.14	-0.01	0.68	0.11	0.96	8.00E-24
IL17	1070	ENSG0000008221	ME2	0.17	-0.03	0.61	0.013	0.79	4.90E-29
IL17	1070	ENSG0000016530	MELK	0.44	0.03	0.91	1.10E-08	0.81	1.30E-36
IL17	1070	ENSG0000003789	METTL1	0.36	0.32	1.02	0.0025	0.019	1.90E-24
IL17	1070	ENSG0000014732	MFHAS1	0.32	0.07	0.85	2.70E-05	0.53	1.10E-35
IL17	1070	ENSG0000016838	MFSD2A	0.23	0.26	0.98	0.35	2	1.70E-08
IL17	1070	ENSG0000007441	MGLL	0.4	0.11	0.81	2.10E-13	0.13	5.30E-55
IL17	1070	ENSG0000011134	MGP	0.27	-0.39	1.04	0.17	0.054	8.00E-12
IL17	1070	ENSG0000020787	MIR221	0.37	0.22	0.63	0.28	2	0.036
IL17	1070	ENSG0000027006	MIR222HG	0.58	-0.13	0.69	2.60E-05	2	1.40E-07
IL17	1070	ENSG0000025352	MIR3142HG	0.19	0.21	0.64	2	2	0.011
IL17	1070	ENSG0000026623	MIR3176	0.16	0.07	0.7	0.72	2	0.032
IL17	1070	ENSG0000026580	MIR4292	0.27	0.39	0.64	0.49	2	0.042
IL17	1070	ENSG0000016840	MLKL	0.48	0.11	0.66	0.0023	2	3.40E-06
IL17	1070	ENSG0000010896	MMD	0.18	0.05	0.62	0.092	0.71	4.30E-13
IL17	1070	ENSG0000016667	MMP10	0.39	0.12	0.62	2	2	0.036
IL17	1070	ENSG0000012334	MMP19	0.41	0.09	0.67	1.80E-09	0.36	8.50E-25
IL17	1070	ENSG0000011811	MMP8	0.21	0.07	0.71	2	2	0.025
IL17	1070	ENSG0000014626	MMS22L	0.2	0.01	0.89	0.066	0.95	1.30E-24
IL17	1070	ENSG0000015804	MRPL17	0.27	0.15	0.59	4.10E-04	0.11	2.30E-18
IL17	1070	ENSG0000005337	MRTO4	0.32	0.19	0.86	5.60E-05	0.048	1.70E-33
IL17	1070	ENSG0000017886	MSC	0.57	0.18	0.81	5.40E-08	0.2	2.00E-16
IL17	1070	ENSG0000009500	MSH2	0.17	-0.13	0.71	0.061	0.2	4.50E-22
IL17	1070	ENSG0000020376	MSTO2P	0.43	0.42	0.6	0.097	2	0.0085
IL17	1070	ENSG0000026054	MT1L	0.22	-0.02	0.61	0.24	2	1.90E-05
IL17	1070	ENSG0000017216	MTBP	-0.01	0	0.83	0.97	2	1.40E-08
IL17	1070	ENSG0000024211	MTFP1	0.2	-0.2	0.81	0.6	2	0.0016
IL17	1070	ENSG0000010071	MTHFD1	0.49	0.19	1.06	9.50E-19	0.0087	8.10E-91
IL17	1070	ENSG0000016691	MTMR10	0.19	-0.08	1.31	0.024	0.39	2.20E-89
IL17	1070	ENSG0000011851	MYB	0.06	0.21	1.38	0.91	2	4.90E-06
IL17	1070	ENSG0000013238	MYBBP1A	0.28	0.23	0.76	1.60E-06	8.60E-04	1.30E-47
IL17	1070	ENSG0000008696	MYBPC2	0.09	-0.05	1.81	0.85	2	3.00E-07
IL17	1070	ENSG0000010417	MYEF2	0.36	-0.1	0.94	0.0041	0.53	1.90E-18
IL17	1070	ENSG0000027825	MYO19	0.3	0.18	0.61	4.40E-06	0.027	1.00E-24
IL17	1070	ENSG0000017239	MYOZ2	0.06	-0.18	1.41	0.92	2	2.20E-05
IL17	1070	ENSG0000020489	MZT1	0.17	-0.05	0.64	0.21	0.75	2.00E-10
IL17	1070	ENSG0000016413	NAA15	0.11	-0.19	0.79	0.22	0.041	1.10E-31
IL17	1070	ENSG0000011130	NAA25	0.13	-0.13	0.71	0.29	0.29	2.60E-16
IL17	1070	ENSG0000010583	NAMPT	0.13	-0.07	1.21	0.13	0.49	4.70E-88
IL17	1070	ENSG0000022964	NAMPTP1	-0.09	-0.19	1.05	0.79	2	7.60E-08
IL17	1070	ENSG0000017019	NANP	0.21	-0.06	0.61	0.1	0.71	2.60E-09
IL17	1070	ENSG0000013278	NASP	0.27	-0.07	0.74	0.00093	0.53	5.20E-24
IL17	1070	ENSG0000016683	NAV2	0.48	0.05	0.82	1.30E-14	0.54	2.70E-43
IL17	1070	ENSG0000006779	NAV3	0	-0.05	1.1	0.99	0.67	9.10E-64
IL17	1070	ENSG0000010449	NCALD	-0.06	-0.07	0.64	2	2	0.041
IL17	1070	ENSG0000015150	NCAPD3	0.4	0.24	0.91	7.50E-10	0.0032	1.30E-52
IL17	1070	ENSG0000010980	NCAPG	0.5	0.03	1.14	5.10E-08	0.84	4.10E-40
IL17	1070	ENSG0000014691	NCAPG2	0.32	0.03	0.74	1.20E-04	0.84	7.90E-23
IL17	1070	ENSG0000011505	NCL	0.1	-0.09	0.62	0.4	0.43	8.40E-15
IL17	1070	ENSG0000012591	NCLN	0.26	0.19	0.63	1.10E-06	0.0033	3.20E-37
IL17	1070	ENSG0000011191	NCOA7	0.02	-0.2	0.76	0.88	0.046	8.70E-26
IL17	1070	ENSG0000018821	NCR3LG1	0.33	0.1	0.92	2.30E-04	0.41	9.60E-32
IL17	1070	ENSG0000005880	NDC1	0.41	0.09	0.97	6.10E-08	0.36	7.20E-46
IL17	1070	ENSG0000008098	NDC80	0.48	0.06	0.71	1.00E-05	0.72	2.60E-12
IL17	1070	ENSG0000011185	NEDD9	-0.07	-0.01	0.65	0.51	0.96	3.90E-22
IL17	1070	ENSG0000010472	NEFM	0.09	-0.28	0.9	0.88	2	0.0047
IL17	1070	ENSG0000010967	NEIL3	0.48	0.11	0.76	4.90E-04	2	9.40E-10
IL17	1070	ENSG0000011765	NEK2	0.49	-0.05	0.65	8.40E-04	0.83	1.50E-06
IL17	1070	ENSG0000016688	NEMP1	0.24	0.05	0.77	0.0016	0.61	2.70E-32
IL17	1070	ENSG0000017120	NETO2	0.54	-0.09	0.75	1.20E-15	0.35	6.00E-31

IL17	1070	ENSG0000008264	NFE2L1	0.08	0.15	0.75	0.27	0.034	5.90E-46
IL17	1070	ENSG0000014786	NFIB	0.11	-0.04	0.6	0.36	0.76	6.20E-14
IL17	1070	ENSG0000010932	NFKB1	0.13	0.01	0.72	0.047	0.88	1.10E-45
IL17	1070	ENSG0000010482	NFKBIB	0.25	0.07	0.96	0.02	0.58	4.80E-29
IL17	1070	ENSG0000010050	NIN	0.09	-0.16	0.59	0.36	0.081	4.90E-18
IL17	1070	ENSG0000013260	NIP7	0.06	0.03	0.65	0.62	0.81	3.30E-19
IL17	1070	ENSG0000017011	NIPA1	0.13	-0.15	0.64	0.31	0.24	2.00E-12
IL17	1070	ENSG0000016703	NKX3-1	0.34	0.27	0.97	0.31	2	1.50E-04
IL17	1070	ENSG0000018226	NLRP10	0.02	0.04	1.04	0.97	2	1.70E-05
IL17	1070	ENSG0000023967	NME1	0.32	0.18	1.02	2.50E-06	0.032	9.30E-65
IL17	1070	ENSG0000001105	NME1-NME2	0.35	0.32	0.7	0.35	2	0.027
IL17	1070	ENSG0000017314	NOC3L	0.09	-0.03	0.97	0.49	0.81	2.30E-33
IL17	1070	ENSG0000018369	NOG	0.3	0.23	0.65	0.05	2	3.70E-07
IL17	1070	ENSG0000016619	NOLC1	0.2	0.04	0.83	4.60E-04	0.6	1.30E-61
IL17	1070	ENSG0000008726	NOP14	0.23	0.09	0.65	6.00E-04	0.25	2.50E-28
IL17	1070	ENSG0000004816	NOP16	0.33	0.18	1.09	6.00E-04	0.13	5.20E-40
IL17	1070	ENSG0000011164	NOP2	0.26	0.23	0.77	0.0049	0.031	2.40E-22
IL17	1070	ENSG0000010136	NOP56	0.26	0.19	0.8	2.60E-05	0.01	1.10E-48
IL17	1070	ENSG0000005504	NOP58	0.13	-0.02	0.8	0.11	0.82	1.90E-35
IL17	1070	ENSG0000017048	NPAS2	0.57	0.14	1.7	5.00E-16	0.14	2.20E-151
IL17	1070	ENSG0000014930	NPAT	0.09	-0.04	0.62	0.48	0.8	1.20E-13
IL17	1070	ENSG0000018397	NPB	0.4	-0.2	0.66	0.055	2	2.00E-04
IL17	1070	ENSG0000018416	NR2C2AP	0.19	0.05	0.79	0.23	2	5.90E-12
IL17	1070	ENSG0000014820	NR6A1	0.25	-0.04	0.81	0.53	2	0.0065
IL17	1070	ENSG0000017400	NRROS	0.56	0.07	0.76	0.1	2	0.018
IL17	1070	ENSG0000010968	NSD2	0.39	0.15	0.66	4.20E-17	0.013	1.00E-50
IL17	1070	ENSG0000012052	NUDCD1	0.04	0.02	0.8	0.81	0.9	1.30E-19
IL17	1070	ENSG0000013615	NUDT15	0.22	0.02	0.75	0.026	0.9	4.80E-20
IL17	1070	ENSG0000011158	NUP107	0.14	-0.08	0.79	0.13	0.46	2.40E-30
IL17	1070	ENSG0000012478	NUP153	0.17	0	0.62	0.0022	0.99	3.40E-40
IL17	1070	ENSG0000011356	NUP155	0.22	0.05	0.68	0.0012	0.61	3.90E-30
IL17	1070	ENSG0000009531	NUP188	0.42	0.24	0.85	8.60E-15	3.30E-04	7.00E-63
IL17	1070	ENSG0000015556	NUP205	0.24	0.05	0.72	2.50E-05	0.51	4.20E-46
IL17	1070	ENSG0000016300	NUP35	0.2	0.12	0.89	0.26	2	5.40E-13
IL17	1070	ENSG0000009300	NUP50	0.26	0.04	0.73	1.80E-06	0.6	9.40E-52
IL17	1070	ENSG0000013949	NUP58	0	-0.09	0.6	0.99	0.3	1.40E-20
IL17	1070	ENSG0000021302	NUP62	0.42	0.16	0.7	3.80E-18	0.011	4.20E-51
IL17	1070	ENSG0000012545	NUP85	0.35	0.18	0.63	1.50E-05	0.069	5.20E-17
IL17	1070	ENSG0000010855	NUP88	0.21	0.13	0.67	0.0046	0.12	7.80E-26
IL17	1070	ENSG0000013780	NUSAP1	0.58	0.04	0.77	6.60E-11	0.8	1.90E-19
IL17	1070	ENSG0000013266	NXT1	0.24	0.24	0.75	0.045	0.059	2.70E-15
IL17	1070	ENSG0000011575	ODC1	0.14	0.14	0.61	0.037	0.054	7.00E-29
IL17	1070	ENSG0000017798	ODF3B	0	-0.22	0.59	0.98	2	0.038
IL17	1070	ENSG0000011990	OGFRL1	0.06	0.09	0.84	0.59	0.38	5.30E-31
IL17	1070	ENSG0000010414	OIP5	0.51	0.24	0.67	0.002	2	1.20E-05
IL17	1070	ENSG0000017339	OLR1	-0.1	-0.19	1.5	0.43	0.12	3.20E-70
IL17	1070	ENSG0000027604	ORAI1	0.29	0.14	0.84	8.10E-04	0.2	2.50E-28
IL17	1070	ENSG0000008584	ORC1	0.52	0	1.08	9.20E-05	2	7.20E-20
IL17	1070	ENSG0000009165	ORC6	0.56	0.22	1.01	1.80E-09	0.072	3.90E-32
IL17	1070	ENSG0000007915	OSBPL6	0.45	0.11	0.97	0.0099	2	3.50E-11
IL17	1070	ENSG0000016482	OSGIN2	0.05	-0.17	0.74	0.77	0.2	2.40E-15
IL17	1070	ENSG0000014562	OSMR	-0.03	0.06	0.62	0.72	0.4	1.20E-38
IL17	1070	ENSG0000016416	OTUD4	-0.04	-0.1	0.66	0.77	0.33	1.30E-18
IL17	1070	ENSG0000015510	OTUD6B	-0.05	-0.07	0.66	0.78	0.68	2.20E-11
IL17	1070	ENSG0000009053	P3H2	0.17	0.02	1.65	0.2	0.92	1.30E-65
IL17	1070	ENSG0000017051	PA2G4	0.31	0.11	0.72	1.20E-10	0.067	2.00E-58
IL17	1070	ENSG0000007664	PAG1	0.5	-0.18	1.24	3.30E-10	0.084	1.90E-63
IL17	1070	ENSG0000012805	PAICS	0.28	0.1	0.83	1.80E-07	0.15	5.90E-62
IL17	1070	ENSG0000011184	PAK1IP1	0.25	0.04	0.83	0.0035	0.74	2.30E-30
IL17	1070	ENSG0000008309	PALB2	0.04	-0.01	0.63	0.8	0.95	7.10E-12
IL17	1070	ENSG0000011021	PANX1	0.29	0	0.74	3.50E-09	0.98	2.80E-59
IL17	1070	ENSG0000016207	PAQR4	0.51	0.22	0.99	4.70E-07	0.096	2.90E-26
IL17	1070	ENSG0000014379	PARP1	0.35	0.07	0.65	6.70E-13	0.26	2.40E-45
IL17	1070	ENSG0000012948	PARP2	0.31	0.08	0.66	0.0021	0.53	5.40E-14
IL17	1070	ENSG0000018548	PARPBP	0.31	0.06	0.68	0.051	2	1.60E-07

IL17	1070	ENSG0000011568	PASK	0.37	0.17	0.79	7.50E-04	0.22	5.10E-16
IL17	1070	ENSG0000018062	PCGF5	-0.17	-0.21	0.77	0.041	0.021	6.20E-30
IL17	1070	ENSG0000016680	PCLAF	0.47	0.25	0.79	5.90E-04	2	1.20E-10
IL17	1070	ENSG0000013264	PCNA	0.46	0.05	0.97	1.40E-12	0.62	2.30E-56
IL17	1070	ENSG0000019764	PDCD1LG2	-0.09	0.09	1.05	0.56	0.52	1.40E-32
IL17	1070	ENSG0000010518	PDCD5	0.16	-0.07	0.64	0.013	0.35	2.50E-31
IL17	1070	ENSG0000015227	PDE3B	0.11	0.03	0.65	0.59	2	1.90E-07
IL17	1070	ENSG0000015566	PDIA4	0.31	0.16	0.7	6.20E-12	0.0058	9.40E-60
IL17	1070	ENSG0000013143	PDLIM4	0.46	0.07	1.01	1.20E-12	0.43	1.80E-62
IL17	1070	ENSG0000016311	PDLIM5	0.04	-0.03	0.68	0.63	0.71	5.50E-42
IL17	1070	ENSG0000016249	PDPN	-0.11	0.16	0.71	0.75	2	5.00E-04
IL17	1070	ENSG0000014845	PDSS1	0.14	-0.21	0.6	0.51	2	3.20E-05
IL17	1070	ENSG0000013340	PDZD2	0.49	-0.18	0.72	0.13	2	0.012
IL17	1070	ENSG0000011475	PEX5L	0.16	-0.09	0.91	0.7	2	2.70E-04
IL17	1070	ENSG0000017892	PFAS	0.45	0.28	0.96	7.10E-11	0.0019	3.60E-50
IL17	1070	ENSG0000024707	PGAM5	0.27	0.05	0.6	1.20E-04	0.64	1.30E-21
IL17	1070	ENSG0000016929	PGM2	0.19	-0.05	0.86	0.042	0.65	4.20E-33
IL17	1070	ENSG0000018164	PHLDA2	0.51	0.17	1.26	2.00E-11	0.093	2.70E-72
IL17	1070	ENSG0000007392	PICALM	0.11	-0.14	0.59	0.048	0.027	7.60E-36
IL17	1070	ENSG0000027716	PIGW	0.17	0.15	0.89	0.3	0.37	6.50E-15
IL17	1070	ENSG0000019835	PIM3	0.38	0.12	0.96	6.00E-07	0.21	5.80E-44
IL17	1070	ENSG0000025409	PINX1	0.05	0.19	0.7	0.86	2	2.80E-06
IL17	1070	ENSG0000012756	PKMYT1	0.56	0.16	1.08	4.50E-07	0.28	2.90E-26
IL17	1070	ENSG0000016044	PKN3	0.45	0.2	0.77	7.10E-05	0.17	1.00E-13
IL17	1070	ENSG0000011671	PLA2G4A	0.35	-0.1	0.68	0.3	2	0.011
IL17	1070	ENSG0000012286	PLAU	0.4	0.27	1.1	2.40E-11	2.30E-04	2.80E-86
IL17	1070	ENSG0000001142	PLAUR	0.26	0.17	1.18	0.0011	0.07	2.60E-67
IL17	1070	ENSG0000011589	PLCL1	0.37	-0.11	1.26	0.064	2	1.20E-15
IL17	1070	ENSG0000015295	PLOD2	-0.01	-0.13	0.86	0.95	0.087	2.80E-58
IL17	1070	ENSG0000012075	PLS1	0	-0.01	0.61	1	2	0.025
IL17	1070	ENSG0000011455	PLXNA1	0.16	0.13	0.91	0.0024	0.04	3.30E-86
IL17	1070	ENSG0000014168	PMAIP1	0.28	0.04	1.1	0.0013	0.73	1.90E-50
IL17	1070	ENSG0000011594	PNO1	0.28	0.25	0.88	0.0032	0.025	9.60E-27
IL17	1070	ENSG0000013803	PNPT1	0.06	-0.16	0.74	0.67	0.16	5.10E-21
IL17	1070	ENSG0000016408	POC1A	0.54	0.29	0.67	6.90E-08	0.021	1.30E-12
IL17	1070	ENSG0000012856	PODXL	0.43	0.09	0.92	0.013	2	3.70E-10
IL17	1070	ENSG0000016338	POGLUT1	0.2	0.01	0.91	0.044	0.92	8.80E-33
IL17	1070	ENSG0000010186	POLA1	0.2	-0.04	0.93	0.031	0.77	3.40E-36
IL17	1070	ENSG0000001413	POLA2	0.46	0.22	0.96	9.10E-08	0.045	1.40E-33
IL17	1070	ENSG0000007751	POLD3	0.16	0.01	0.62	0.071	0.94	6.60E-19
IL17	1070	ENSG0000017708	POLE	0.54	0.38	1	6.30E-15	7.90E-06	3.50E-52
IL17	1070	ENSG0000010047	POLE2	0.44	0.13	1.23	0.004	2	1.20E-21
IL17	1070	ENSG0000014822	POLE3	0.27	0.2	0.61	1.90E-04	0.02	5.80E-21
IL17	1070	ENSG0000005134	POLQ	0.58	-0.03	1.33	1.30E-05	0.88	1.50E-28
IL17	1070	ENSG0000006865	POLR1A	0.28	0.15	0.62	6.10E-07	0.029	1.30E-32
IL17	1070	ENSG0000011335	POLR3G	0.21	0.02	0.96	0.37	2	1.40E-09
IL17	1070	ENSG0000016198	POLR3K	0.04	-0.02	0.61	0.84	0.94	5.60E-09
IL17	1070	ENSG0000010435	POP1	0.44	0.14	0.89	3.90E-06	0.28	2.90E-24
IL17	1070	ENSG0000012805	PPAT	0.05	-0.1	0.92	0.78	0.44	1.00E-25
IL17	1070	ENSG0000016638	PPFIBP2	0.25	-0.14	0.94	0.16	2	8.00E-12
IL17	1070	ENSG0000010817	PPIF	0.39	0.19	1.25	1.50E-10	0.016	8.70E-113
IL17	1070	ENSG0000007715	PPP1R12B	0.15	0.05	0.59	0.068	0.66	3.60E-18
IL17	1070	ENSG0000014884	PPRC1	0.27	0.22	0.7	1.30E-07	4.10E-04	3.20E-50
IL17	1070	ENSG0000019685	PPTC7	0.16	0.1	0.62	0.11	0.35	3.70E-16
IL17	1070	ENSG0000019890	PRC1	0.5	0.21	0.63	1.90E-06	0.11	1.80E-10
IL17	1070	ENSG0000005765	PRDM1	0.17	0.21	0.77	0.24	0.14	1.70E-13
IL17	1070	ENSG0000019805	PRIM1	0.3	0.13	0.69	0.057	2	1.10E-07
IL17	1070	ENSG0000012645	PRMT1	0.25	0.16	0.78	5.00E-06	0.016	5.20E-55
IL17	1070	ENSG0000018523	PRMT3	0.07	0.04	0.61	0.62	0.77	1.40E-11
IL17	1070	ENSG0000010191	PRPS2	0.22	-0.09	0.73	0.026	0.47	1.00E-18
IL17	1070	ENSG0000021212	PRR22	0.52	0.32	0.83	0.082	2	0.0017
IL17	1070	ENSG0000000500	PRSS22	0.79	-0.03	1.04	2	2	0.0018
IL17	1070	ENSG0000017822	PRSS36	0.6	0.4	1.25	0.063	2	2.40E-05
IL17	1070	ENSG0000010056	PSMA3	0.09	-0.13	0.72	0.2	0.074	5.10E-41
IL17	1070	ENSG0000013147	PSMC3IP	0.35	0.11	0.97	0.0029	0.46	1.20E-21

IL17	1070	ENSG0000011523	PSMD14	0.19	-0.02	0.68	0.0061	0.86	6.70E-32
IL17	1070	ENSG0000010091	PSME2	0.54	0.07	1.29	3.20E-17	0.42	2.70E-102
IL17	1070	ENSG0000022513	PSME2P2	0.34	-0.06	1.36	0.35	2	1.30E-06
IL17	1070	ENSG0000017152	PTGER4	0.34	0.29	0.75	9.80E-06	0.0026	1.00E-26
IL17	1070	ENSG0000007015	PTPN3	0.5	0.04	0.8	0.0059	2	9.10E-07
IL17	1070	ENSG0000013233	PTPRE	0.42	0.17	1.18	0.12	2	5.60E-08
IL17	1070	ENSG0000014137	PTRH2	0.09	0.05	0.69	0.49	0.71	3.30E-16
IL17	1070	ENSG0000016461	PTTG1	0.47	0.12	0.6	2.30E-06	0.35	3.50E-10
IL17	1070	ENSG0000016366	PTX3	-0.21	0.26	0.64	2.10E-06	4.50E-08	5.20E-57
IL17	1070	ENSG0000008060	PUM3	-0.13	-0.11	0.66	0.062	0.14	3.30E-35
IL17	1070	ENSG0000017719	PUS1	0.2	0.13	0.63	0.088	0.3	3.40E-12
IL17	1070	ENSG0000009112	PUS7	0.12	-0.12	0.87	0.35	0.35	2.70E-25
IL17	1070	ENSG0000016566	QSOX2	0.16	0.24	0.79	0.047	0.0062	6.90E-34
IL17	1070	ENSG0000015157	QTRT2	0.06	-0.05	0.65	0.68	0.73	1.20E-15
IL17	1070	ENSG0000014354	RAB13	0.48	-0.09	0.64	2.70E-13	0.34	7.90E-24
IL17	1070	ENSG0000013795	RABGGTB	0	0.01	0.75	0.98	0.96	7.70E-33
IL17	1070	ENSG0000016180	RACGAP1	0.56	0.15	0.72	1.10E-11	0.15	1.00E-19
IL17	1070	ENSG0000007095	RAD18	0.36	0.08	0.89	4.80E-04	0.59	1.00E-22
IL17	1070	ENSG0000005118	RAD51	0.28	0.12	0.87	0.018	0.43	2.80E-19
IL17	1070	ENSG0000019727	RAD54B	0.23	-0.04	0.91	0.45	2	1.50E-05
IL17	1070	ENSG0000009990	RANBP1	0.22	0.06	0.6	3.50E-04	0.47	2.40E-27
IL17	1070	ENSG0000013704	RANBP6	0.03	-0.1	0.62	0.85	0.35	1.10E-17
IL17	1070	ENSG0000023660	RANP1	0.23	0.21	0.69	0.41	2	7.20E-04
IL17	1070	ENSG0000012731	RAP1B	-0.07	-0.21	0.59	0.42	0.0069	8.50E-25
IL17	1070	ENSG0000011884	RARRES1	-0.22	0.26	0.59	0.41	2	0.0031
IL17	1070	ENSG0000017257	RASGRP1	-0.34	0.06	0.93	0.075	2	2.00E-10
IL17	1070	ENSG0000015268	RASGRP3	0.42	-0.36	0.59	0.028	2	4.60E-04
IL17	1070	ENSG0000010177	RBBP8	0.2	-0.07	0.93	0.015	0.5	7.10E-44
IL17	1070	ENSG0000008083	RBL1	0.28	0.04	0.9	0.014	0.79	6.00E-21
IL17	1070	ENSG0000023930	RBM14	0.29	0.19	0.62	1.30E-05	0.021	4.50E-25
IL17	1070	ENSG0000016683	RBPMS2	0.31	0.21	0.75	0.21	2	1.00E-04
IL17	1070	ENSG0000017905	RCC2	0.35	0.1	0.65	2.90E-11	0.14	1.90E-38
IL17	1070	ENSG0000012015	RCL1	0.34	0.25	0.96	0.014	2	8.70E-18
IL17	1070	ENSG0000000470	RECQL	0.2	-0.09	0.62	0.0099	0.32	3.70E-21
IL17	1070	ENSG0000005496	RELT	0.46	0.22	1.15	0.01	2	3.00E-14
IL17	1070	ENSG0000004954	RFC2	0.33	0.07	0.81	2.60E-05	0.51	5.00E-32
IL17	1070	ENSG0000016391	RFC4	0.38	0.01	0.99	2.10E-05	0.95	1.40E-36
IL17	1070	ENSG0000011144	RFC5	0.25	-0.05	0.64	0.009	0.7	4.00E-15
IL17	1070	ENSG0000013137	RFTN1	0.39	-0.06	0.9	8.10E-11	0.45	5.50E-59
IL17	1070	ENSG0000016841	RFWD3	0.53	0.22	0.92	5.30E-14	0.015	2.30E-44
IL17	1070	ENSG0000017413	RGMB	0.32	0.17	1.12	7.70E-15	0.002	8.40E-182
IL17	1070	ENSG0000016962	RGPD8	0.21	0.23	0.74	0.6	2	0.0069
IL17	1070	ENSG0000018290	RGS7	0.45	-0.19	0.69	0.2	2	0.024
IL17	1070	ENSG0000012478	RIOK1	0.03	0.08	0.59	0.84	0.47	1.10E-16
IL17	1070	ENSG0000010488	RNASEH2A	0.41	0.21	0.72	8.70E-07	0.045	1.20E-20
IL17	1070	ENSG0000017260	RND1	0.19	-0.36	0.72	0.65	2	0.029
IL17	1070	ENSG0000014586	RNF145	0.05	-0.07	0.69	0.46	0.25	6.10E-63
IL17	1070	ENSG0000011651	RNF19B	0.19	0.03	0.83	0.087	0.82	1.90E-22
IL17	1070	ENSG0000015828	RNF207	0.56	0.13	0.9	0.0044	2	1.90E-07
IL17	1070	ENSG0000015219	RNF219	-0.1	-0.1	0.6	0.49	0.47	1.80E-10
IL17	1070	ENSG0000020671	RNU6-26P	0.34	0.39	1.35	0.35	2	6.00E-06
IL17	1070	ENSG0000016358	RPL22L1	0.34	-0.05	0.67	1.50E-05	0.68	1.10E-20
IL17	1070	ENSG0000012478	RPP40	0.26	-0.08	0.64	0.12	2	1.10E-06
IL17	1070	ENSG0000024174	RPSAP52	0.44	0.09	0.77	0.082	2	2.70E-04
IL17	1070	ENSG0000016732	RRM1	0.34	0.04	0.8	9.90E-08	0.66	6.50E-42
IL17	1070	ENSG0000005274	RRP12	0.25	0.23	0.71	0.0014	0.011	3.00E-26
IL17	1070	ENSG0000006753	RRP15	-0.02	-0.06	0.68	0.9	0.67	7.90E-14
IL17	1070	ENSG0000011476	RRP9	0.19	0.17	0.78	0.097	0.16	2.40E-17
IL17	1070	ENSG0000017904	RRS1	0.42	0.25	0.95	4.00E-06	0.025	2.10E-30
IL17	1070	ENSG0000014637	RSPO3	-0.27	-0.32	0.77	0.47	2	0.012
IL17	1070	ENSG0000017343	SAA1	0	0.06	0.8	2	2	8.70E-04
IL17	1070	ENSG0000013059	SAMD10	0.53	-0.01	1.1	0.0015	2	2.70E-14
IL17	1070	ENSG0000015687	SASS6	0.26	-0.01	0.84	0.18	2	1.50E-08
IL17	1070	ENSG0000024627	SBF2-AS1	0.48	-0.01	0.92	2.70E-05	0.96	1.10E-18
IL17	1070	ENSG0000007306	SCARB1	0.4	0.22	0.59	2.20E-10	0.0058	6.50E-23

IL17	1070	ENSG0000018417	SCFD2	0.37	0.25	0.73	2.80E-04	0.038	9.20E-16
IL17	1070	ENSG0000004763	SCML1	-0.06	-0.08	0.67	0.67	0.48	5.40E-17
IL17	1070	ENSG0000010209	SCML2	0.08	0.06	0.61	0.79	2	0.0011
IL17	1070	ENSG0000011588	SDC1	0.45	0.25	0.74	3.90E-11	0.0047	7.70E-30
IL17	1070	ENSG0000012822	SDF2L1	0.4	0.17	1	3.30E-05	0.16	5.90E-31
IL17	1070	ENSG0000016656	SEC11C	0.26	-0.01	0.83	0.12	2	1.30E-10
IL17	1070	ENSG0000010131	SEC23B	0.28	0.09	0.76	8.60E-07	0.22	6.50E-50
IL17	1070	ENSG0000008541	SEH1L	0.13	0.04	0.66	0.12	0.72	5.30E-27
IL17	1070	ENSG0000013801	SELENOI	0.09	-0.07	0.75	0.49	0.57	1.80E-19
IL17	1070	ENSG0000019884	SELENOT	0.07	-0.02	0.59	0.39	0.84	2.70E-25
IL17	1070	ENSG0000007521	SEMA3A	0.44	-0.07	0.65	2.40E-11	0.44	1.40E-25
IL17	1070	ENSG0000017038	SEMA3E	-0.19	-0.13	0.84	0.46	2	6.30E-07
IL17	1070	ENSG0000011290	SEMA5A	0.26	-0.05	0.88	1.60E-06	0.5	2.20E-68
IL17	1070	ENSG0000024844	SEMA6A-AS1	0.42	0.38	0.66	0.25	2	0.034
IL17	1070	ENSG0000013862	SEMA7A	0.36	0.03	1.05	8.80E-06	0.79	1.90E-48
IL17	1070	ENSG0000019763	SERPINB2	0.1	0.27	1.99	0.69	0.16	1.10E-47
IL17	1070	ENSG0000016640	SERPINB8	0.08	-0.02	0.68	0.58	0.89	4.80E-16
IL17	1070	ENSG0000017054	SERPINB9	0.12	0.15	1.84	0.51	0.38	3.90E-69
IL17	1070	ENSG0000010636	SERPINE1	0.12	0.24	1.19	0.077	8.40E-04	3.90E-104
IL17	1070	ENSG0000016393	SFMBT1	0.2	0.25	0.83	0.37	2	1.40E-07
IL17	1070	ENSG0000018360	SFXN4	0.26	0.32	0.76	0.025	0.013	5.20E-15
IL17	1070	ENSG0000016353	SGO2	0.27	-0.09	0.76	0.013	0.5	3.20E-16
IL17	1070	ENSG0000011125	SH2B3	0.35	0.15	0.66	1.30E-12	0.016	5.40E-46
IL17	1070	ENSG0000010573	SIPA1L3	0.5	0.18	0.83	4.20E-18	0.018	2.70E-52
IL17	1070	ENSG0000017057	SIX2	-0.08	-0.09	1.27	0.85	2	2.20E-04
IL17	1070	ENSG0000015483	SKA1	0.58	0.17	0.89	6.40E-07	0.26	2.30E-16
IL17	1070	ENSG0000011350	SLC12A7	0.45	0.19	0.59	1.80E-11	0.027	2.00E-20
IL17	1070	ENSG0000022195	SLC12A8	0.22	0.13	0.99	0.15	2	1.30E-18
IL17	1070	ENSG0000017363	SLC19A1	0.35	0.28	0.85	1.30E-04	0.0094	5.00E-26
IL17	1070	ENSG0000013591	SLC19A3	-0.59	-0.1	0.95	0.052	2	0.0042
IL17	1070	ENSG0000014413	SLC20A1	0.44	0.11	0.61	2.20E-23	0.063	3.00E-46
IL17	1070	ENSG0000013726	SLC22A23	0.51	0.28	0.67	0.14	2	0.034
IL17	1070	ENSG0000019720	SLC22A4	0.51	0.37	1.25	0.013	2	6.80E-13
IL17	1070	ENSG0000000486	SLC25A13	0.24	-0.04	0.59	0.0063	0.74	1.20E-14
IL17	1070	ENSG0000014028	SLC27A2	0.28	0.1	1.4	0.41	2	3.10E-05
IL17	1070	ENSG0000016032	SLC2A6	0.31	0.26	1.24	0.0055	0.046	6.20E-40
IL17	1070	ENSG0000013686	SLC31A2	0.14	0	0.69	0.54	2	4.90E-06
IL17	1070	ENSG0000016019	SLC37A1	0.52	0.11	0.81	0.11	2	0.005
IL17	1070	ENSG0000001748	SLC38A5	0.38	0.35	0.73	2.70E-05	0.0018	9.40E-19
IL17	1070	ENSG0000013882	SLC39A8	0.12	-0.07	0.96	0.72	2	5.30E-07
IL17	1070	ENSG0000013605	SLC41A2	0.18	-0.17	0.72	0.41	2	1.60E-06
IL17	1070	ENSG0000016770	SLC43A2	0.25	0.16	0.59	0.073	2	2.80E-07
IL17	1070	ENSG0000013480	SLC43A3	0.43	0.1	0.99	1.50E-05	0.46	2.30E-29
IL17	1070	ENSG0000003386	SLC4A7	0.14	-0.14	0.79	0.15	0.2	4.30E-25
IL17	1070	ENSG0000013807	SLC5A6	0.31	0.27	1.06	4.80E-05	0.0035	4.60E-57
IL17	1070	ENSG0000001108	SLC6A7	0.26	0.02	1.22	2	2	5.80E-04
IL17	1070	ENSG0000015546	SLC7A7	0.29	0.11	1.01	0.0052	0.38	3.00E-32
IL17	1070	ENSG0000010067	SLC8A3	0.21	0.11	0.77	0.62	2	0.015
IL17	1070	ENSG0000006592	SLC9A7	0.38	0.09	0.63	3.10E-05	0.45	1.10E-13
IL17	1070	ENSG0000018180	SLC9A9	0.27	-0.07	0.94	0.002	0.55	2.80E-36
IL17	1070	ENSG0000013757	SLCO5A1	0.19	-0.09	1.08	2	2	0.0033
IL17	1070	ENSG0000013330	SLF1	0.12	-0.1	0.74	0.51	0.58	6.10E-11
IL17	1070	ENSG0000017271	SLFN11	0.37	0.02	1.01	1.10E-11	0.78	1.20E-88
IL17	1070	ENSG0000014514	SLIT2	0.45	0.14	0.84	3.20E-11	0.1	1.20E-40
IL17	1070	ENSG0000018598	SLITRK2	0.2	0.08	0.64	0.45	2	3.70E-04
IL17	1070	ENSG0000007250	SMC1A	0.24	0.01	0.67	9.40E-04	0.91	4.90E-26
IL17	1070	ENSG0000013682	SMC2	0.32	-0.17	0.83	4.40E-05	0.077	2.90E-32
IL17	1070	ENSG0000011381	SMC4	0.29	-0.06	0.67	1.60E-05	0.5	3.80E-27
IL17	1070	ENSG0000016593	SMCO2	0.48	0.07	1.02	0.15	2	0.0012
IL17	1070	ENSG0000017925	SMCO3	0.56	0.13	0.88	2	2	0.0037
IL17	1070	ENSG0000022453	SMIM13	-0.04	-0.08	0.72	0.74	0.37	7.00E-30
IL17	1070	ENSG0000006469	SNCAIP	0.24	-0.18	0.82	0.15	2	9.20E-11
IL17	1070	ENSG0000025571	SNHG1	0.1	0.08	0.6	0.25	0.37	3.30E-21
IL17	1070	ENSG0000019798	SNHG12	0.13	0.18	0.61	0.44	0.24	4.60E-08
IL17	1070	ENSG0000023295	SNHG15	0.26	0.3	0.89	0.043	0.037	1.00E-17

IL17	1070	ENSG0000026640	SNHG25	0.62	0.68	0.85	0.06	2	0.0049
IL17	1070	ENSG0000022236	SNORD12B	0.35	0.17	0.66	2	2	0.037
IL17	1070	ENSG0000013187	SNRPA1	0.11	-0.03	0.79	0.31	0.8	2.20E-26
IL17	1070	ENSG0000016708	SNRPD1	0.12	-0.07	0.74	0.15	0.45	9.80E-34
IL17	1070	ENSG0000011209	SOD2	-0.01	-0.08	1.68	0.95	0.35	2.80E-166
IL17	1070	ENSG0000017124	SOSTDC1	0.59	-0.03	0.85	0.085	2	0.0072
IL17	1070	ENSG0000018912	SP6	0.47	-0.26	1.56	0.16	2	1.40E-08
IL17	1070	ENSG0000014537	SPATA5	0.27	0.17	0.74	0.13	2	5.90E-08
IL17	1070	ENSG0000004027	SPDL1	0.26	0.01	0.94	0.0054	0.92	2.40E-32
IL17	1070	ENSG0000026940	SPIB	0.14	-0.03	1.85	0.49	2	1.20E-05
IL17	1070	ENSG0000017162	SPSB1	0.46	0.14	0.65	3.70E-10	0.14	2.80E-20
IL17	1070	ENSG0000013454	SPX	0.26	-0.04	1.05	0.52	2	3.00E-04
IL17	1070	ENSG0000013776	SQOR	0.27	0.1	0.75	0.023	0.5	4.50E-14
IL17	1070	ENSG0000015130	SRFBP1	-0.14	-0.24	0.68	0.42	0.14	2.20E-08
IL17	1070	ENSG0000011664	SRM	0.26	0.21	0.64	3.40E-07	6.20E-04	7.70E-42
IL17	1070	ENSG0000009606	SRPK1	0.19	-0.06	0.65	0.0049	0.48	4.60E-29
IL17	1070	ENSG0000014486	SRPRB	0.16	0.15	0.64	0.018	0.042	2.70E-31
IL17	1070	ENSG0000016154	SRSF2	0.28	0.09	0.59	5.60E-12	0.096	9.10E-54
IL17	1070	ENSG0000011587	SRSF7	0.23	0.06	0.69	5.10E-05	0.45	3.80E-43
IL17	1070	ENSG0000012478	SSR1	0.04	-0.09	0.61	0.62	0.14	1.50E-43
IL17	1070	ENSG0000011485	SSR3	0	-0.07	0.64	0.98	0.26	4.50E-45
IL17	1070	ENSG0000018061	SSTR2	0.45	0.04	2.44	0.082	2	5.90E-09
IL17	1070	ENSG0000011353	ST8SIA4	0.58	-0.27	1.14	3.70E-05	2	3.00E-19
IL17	1070	ENSG0000013813	STAMBPL1	0.33	-0.17	0.87	1.30E-05	0.07	2.00E-39
IL17	1070	ENSG0000014746	STAR	0	0.25	0.9	1	2	2.50E-13
IL17	1070	ENSG0000016464	STEAP1	-0.24	0.02	0.85	0.29	2	1.60E-07
IL17	1070	ENSG0000012347	STIL	0.47	0.18	0.94	3.80E-06	0.17	1.70E-24
IL17	1070	ENSG0000016843	STIP1	0.28	0.11	0.62	3.10E-10	0.045	1.20E-50
IL17	1070	ENSG0000013560	STX11	0.09	0.37	0.92	0.82	2	5.70E-06
IL17	1070	ENSG0000016895	STXBP6	0.53	0.08	0.82	0.056	2	7.40E-04
IL17	1070	ENSG0000013757	SULF1	0.49	0.34	2.33	1.20E-06	0.0063	4.50E-158
IL17	1070	ENSG0000009220	SUPT16H	0.08	-0.15	0.61	0.3	0.047	9.30E-26
IL17	1070	ENSG0000010194	SUV39H1	0.46	0.25	0.81	4.50E-05	0.072	3.90E-15
IL17	1070	ENSG0000015245	SUV39H2	0.21	-0.13	0.87	0.1	0.36	1.30E-19
IL17	1070	ENSG0000010863	SYNGR2	0.43	-0.02	0.87	1.10E-06	0.89	1.00E-27
IL17	1070	ENSG0000017074	SYT9	0.2	0.06	0.62	2	2	0.014
IL17	1070	ENSG0000020426	TAP2	0.4	0.17	0.98	1.40E-05	0.15	4.90E-33
IL17	1070	ENSG0000023192	TAPBP	0.53	0.04	0.89	2.90E-27	0.57	6.50E-79
IL17	1070	ENSG0000005958	TARBP1	0.25	-0.04	0.62	0.0031	0.74	1.00E-17
IL17	1070	ENSG0000013731	TCF19	0.5	0.29	1.02	4.80E-12	0.0017	2.20E-50
IL17	1070	ENSG0000015179	TDO2	0.25	-0.25	1.9	0.53	2	4.50E-11
IL17	1070	ENSG0000004208	TDP1	0.39	0.11	0.73	9.30E-06	0.35	5.00E-20
IL17	1070	ENSG0000019790	TEAD4	0.24	0.23	0.6	0.036	0.065	3.20E-10
IL17	1070	ENSG0000022667	TEX41	0.29	0.04	0.61	2	2	0.037
IL17	1070	ENSG0000010806	TFAM	0.02	-0.01	0.67	0.85	0.9	3.40E-24
IL17	1070	ENSG0000010582	TFPI2	0.25	-0.05	1.31	0.011	0.68	7.20E-63
IL17	1070	ENSG0000007227	TFRC	0.21	-0.02	0.79	0.036	0.91	1.10E-21
IL17	1070	ENSG0000013019	THEM6	0.39	0.29	0.67	0.033	2	1.60E-05
IL17	1070	ENSG0000017200	THOP1	0.36	0.27	0.61	3.80E-07	0.002	3.80E-20
IL17	1070	ENSG0000014536	TIFA	0.21	0	0.82	0.19	0.99	8.80E-12
IL17	1070	ENSG0000007823	TIGAR	0.25	-0.15	0.66	0.035	0.27	3.10E-11
IL17	1070	ENSG0000011160	TIMELESS	0.48	0.15	0.85	2.70E-13	0.083	3.10E-42
IL17	1070	ENSG0000007513	TIPIN	0.25	0.06	0.87	0.12	2	1.50E-12
IL17	1070	ENSG0000011913	TJP2	0.35	-0.01	0.8	3.80E-07	0.94	9.70E-37
IL17	1070	ENSG0000016060	TLCD1	0.08	0.05	1.25	0.71	2	9.80E-26
IL17	1070	ENSG0000010682	TLE4	0.47	0.04	0.63	2.20E-15	0.66	9.80E-28
IL17	1070	ENSG0000017412	TLR1	0.55	-0.28	2.2	0.0045	2	9.60E-44
IL17	1070	ENSG0000017412	TLR10	0.34	0.12	0.79	2	2	0.0073
IL17	1070	ENSG0000017413	TLR6	0.54	-0.2	1.48	1.90E-04	2	8.80E-32
IL17	1070	ENSG0000005770	TMCC3	0.51	0.03	0.91	0.0057	2	1.60E-08
IL17	1070	ENSG0000013936	TMEM132B	0.22	0.03	1.29	0.54	2	5.60E-08
IL17	1070	ENSG0000017064	TMEM133	0.31	0.08	1.07	0.19	2	2.00E-09
IL17	1070	ENSG0000013721	TMEM14B	0.22	0.05	0.62	0.0049	0.64	2.50E-19
IL17	1070	ENSG0000017000	TMEM154	0.15	-0.03	0.65	0.57	2	2.30E-04
IL17	1070	ENSG0000012189	TMEM156	-0.1	-0.03	0.72	0.82	2	0.021

IL17	1070	ENSG0000015760	TMEM164	0.38	0.06	0.73	1.50E-05	0.65	1.70E-19
IL17	1070	ENSG0000013504	TMEM2	0.03	-0.14	0.6	0.83	0.12	8.50E-20
IL17	1070	ENSG0000016448	TMEM200A	0.28	0	0.79	9.40E-04	0.97	4.50E-28
IL17	1070	ENSG0000018880	TMEM201	0.1	0.21	0.6	0.41	0.05	1.40E-13
IL17	1070	ENSG0000017273	TMEM217	0.46	0.19	0.6	0.074	2	0.0078
IL17	1070	ENSG0000012535	TMEM255A	0.36	-0.18	0.68	0.2	2	0.0028
IL17	1070	ENSG0000010913	TMEM33	0.07	-0.1	0.65	0.43	0.26	7.60E-27
IL17	1070	ENSG0000009520	TMEM38B	0.28	0.15	0.94	0.13	2	2.60E-11
IL17	1070	ENSG0000017172	TMEM51	0.52	0.1	0.79	5.70E-06	0.56	9.40E-14
IL17	1070	ENSG0000015207	TMEM56	0.17	0.08	0.63	0.59	2	0.0038
IL17	1070	ENSG0000018521	TNFAIP2	0.54	0.18	0.83	6.00E-19	0.027	3.60E-46
IL17	1070	ENSG0000010468	TNFRSF10A	0.49	0.22	1.22	0.0012	2	1.20E-20
IL17	1070	ENSG0000012088	TNFRSF10B	0.33	0.07	0.74	5.50E-16	0.19	9.40E-82
IL17	1070	ENSG0000014607	TNFRSF21	-0.11	0.15	0.62	0.26	0.1	2.20E-19
IL17	1070	ENSG0000018682	TNFRSF4	0.15	-0.09	0.66	0.75	2	0.043
IL17	1070	ENSG0000012094	TNFRSF8	0.48	0.13	0.83	0.19	2	0.0082
IL17	1070	ENSG0000004924	TNFRSF9	0.37	0.08	0.99	0.18	2	0.0019
IL17	1070	ENSG0000012185	TNFSF10	0.26	-0.02	0.66	0.21	2	0.0069
IL17	1070	ENSG0000010252	TNFSF13B	0.43	-0.31	0.96	0.079	2	1.50E-06
IL17	1070	ENSG0000018163	TNFSF15	0.41	0.4	1.47	0.0032	0.012	4.00E-38
IL17	1070	ENSG0000013277	TOE1	0.24	0.29	0.7	0.065	0.035	1.90E-11
IL17	1070	ENSG0000013020	TOMM40	0.28	0.15	0.74	2.00E-06	0.043	2.80E-43
IL17	1070	ENSG0000016094	TONSL	0.48	0.29	0.99	1.30E-09	0.0037	9.20E-42
IL17	1070	ENSG0000013174	TOP2A	0.49	-0.03	0.77	4.60E-05	0.87	3.50E-12
IL17	1070	ENSG0000016378	TOPBP1	0.19	-0.13	0.64	0.01	0.11	8.70E-26
IL17	1070	ENSG0000013736	TPMT	0.33	-0.1	1.17	3.10E-04	0.43	4.80E-51
IL17	1070	ENSG0000007660	TRAF4	0.4	0.19	0.8	1.40E-09	0.027	5.80E-38
IL17	1070	ENSG0000012277	TRIM24	0.17	-0.1	0.59	0.048	0.29	5.30E-19
IL17	1070	ENSG0000008919	TRMT6	0.04	-0.04	0.68	0.81	0.81	8.10E-13
IL17	1070	ENSG0000013310	TRPC4	-0.09	0.21	1.36	0.78	2	4.00E-14
IL17	1070	ENSG0000014577	TSLP	0.57	-0.05	0.89	9.60E-06	2	6.80E-14
IL17	1070	ENSG0000009928	TSPAN15	0.17	0.01	0.6	2	2	0.05
IL17	1070	ENSG0000016772	TSR1	0.16	0.06	0.64	0.0047	0.44	1.20E-41
IL17	1070	ENSG0000001869	TTC27	0.21	0.11	0.61	0.0043	0.2	1.10E-22
IL17	1070	ENSG0000010030	TTLL12	0.36	0.17	0.72	9.20E-09	0.032	4.90E-36
IL17	1070	ENSG0000013591	TTLL4	0.34	0.05	1.07	1.80E-05	0.64	1.10E-55
IL17	1070	ENSG0000016755	TUBA1C	0.45	0.19	0.86	8.60E-14	0.015	2.10E-50
IL17	1070	ENSG0000010781	TWNK	0.2	0.07	0.63	0.12	0.64	3.30E-10
IL17	1070	ENSG0000011714	UAP1	0.13	-0.02	0.78	0.078	0.86	6.40E-41
IL17	1070	ENSG0000015412	UBASH3B	0.3	0.05	0.86	3.20E-07	0.57	1.50E-58
IL17	1070	ENSG0000007715	UBE2T	0.56	0.09	1	1.00E-07	0.54	1.00E-24
IL17	1070	ENSG0000011893	UCHL3	0.15	0.1	0.63	0.5	2	3.20E-05
IL17	1070	ENSG0000014317	UCK2	0.39	0.14	0.87	2.50E-12	0.047	8.80E-63
IL17	1070	ENSG0000017556	UCP3	0.55	0.09	0.61	0.057	2	0.022
IL17	1070	ENSG0000027604	UHRF1	0.52	0.25	1.04	1.00E-13	0.0063	1.00E-55
IL17	1070	ENSG0000013101	ULBP2	0.02	0.09	0.69	0.98	2	0.026
IL17	1070	ENSG0000007624	UNG	0.31	0.16	0.6	6.60E-05	0.096	2.10E-17
IL17	1070	ENSG0000018369	UPP1	0.21	0	0.66	0.094	0.98	9.70E-12
IL17	1070	ENSG0000013576	URB2	0.4	0.22	0.9	1.20E-06	0.028	6.00E-35
IL17	1070	ENSG0000016260	USP1	0.07	-0.05	0.67	0.36	0.58	2.70E-38
IL17	1070	ENSG0000005805	USP13	0.18	0.01	0.61	0.02	0.91	2.70E-22
IL17	1070	ENSG0000010340	USP31	0.53	-0.03	1.27	4.60E-08	0.86	2.10E-47
IL17	1070	ENSG0000013591	USP37	-0.03	-0.06	0.69	0.88	0.68	5.90E-14
IL17	1070	ENSG0000016466	USP49	0.29	-0.11	0.77	0.0039	0.41	2.70E-19
IL17	1070	ENSG0000016433	UTP15	0.06	-0.08	1.01	0.74	0.57	1.70E-28
IL17	1070	ENSG0000012080	UTP20	0.19	-0.03	0.78	0.016	0.75	1.20E-34
IL17	1070	ENSG0000014107	UTP4	0.2	0.18	0.69	0.0049	0.032	5.80E-30
IL17	1070	ENSG0000020439	VARS	0.26	0.15	0.64	3.80E-09	0.0077	5.90E-54
IL17	1070	ENSG0000016269	VCAM1	0.26	0.13	2.37	0.0013	0.19	2.10E-259
IL17	1070	ENSG0000015063	VEGFC	0.11	0.13	0.94	0.1	0.055	2.40E-81
IL17	1070	ENSG0000019741	VEPH1	0.1	0.18	0.67	0.57	0.23	7.90E-10
IL17	1070	ENSG0000026835	VN1R81P	0.49	0.23	0.87	0.14	2	0.0027
IL17	1070	ENSG0000011229	VNN1	0.25	0.02	1.16	2	2	6.10E-04
IL17	1070	ENSG0000016798	VPS37C	0.37	0.1	0.67	1.80E-08	0.25	3.80E-27
IL17	1070	ENSG0000026137	VPS9D1-AS1	0.31	0.24	1.01	0.1	2	7.80E-12

IL17	1070	ENSG0000010074	VRK1	0.35	0.02	0.98	0.0042	0.92	2.50E-21
IL17	1070	ENSG0000008446	WBP11	0.23	0.07	0.63	1.30E-06	0.28	1.40E-49
IL17	1070	ENSG0000019855	WDHD1	0.4	0	1.04	1.20E-05	0.98	3.30E-38
IL17	1070	ENSG0000013844	WDR12	0.23	0.05	0.71	0.0043	0.65	9.30E-26
IL17	1070	ENSG0000006518	WDR3	-0.02	-0.17	0.62	0.87	0.055	9.10E-23
IL17	1070	ENSG0000016019	WDR4	0.33	0.22	1.02	0.005	0.11	9.00E-26
IL17	1070	ENSG0000016381	WDR43	0.12	-0.03	0.74	0.1	0.78	3.00E-37
IL17	1070	ENSG0000009247	WDR76	0.37	0.1	0.97	1.10E-04	0.42	8.70E-30
IL17	1070	ENSG0000011645	WDR77	0.19	0.26	0.66	0.035	0.0079	1.30E-19
IL17	1070	ENSG0000014000	WDR89	0.19	-0.13	1	0.2	0.43	3.40E-21
IL17	1070	ENSG0000014645	WTAP	0.08	-0.07	0.73	0.24	0.34	1.00E-51
IL17	1070	ENSG0000013253	XAF1	0.46	-0.09	0.84	3.60E-04	0.61	4.60E-13
IL17	1070	ENSG0000016309	XIRP2	0.33	-0.11	1.07	0.38	2	8.70E-04
IL17	1070	ENSG0000012457	XPO5	0.28	0.14	0.84	1.30E-06	0.051	2.40E-58
IL17	1070	ENSG0000019658	XRCC2	0.52	0.03	1.18	1.20E-04	0.88	3.90E-23
IL17	1070	ENSG0000012621	XRCC3	0.51	0.18	0.89	5.50E-08	0.13	4.30E-24
IL17	1070	ENSG0000009321	XYLB	0.49	0.26	0.6	0.0054	2	2.10E-04
IL17	1070	ENSG0000019644	YRDC	0.12	0.09	0.59	0.38	0.52	4.50E-10
IL17	1070	ENSG0000028193	Z98044.1	0.19	0.08	1	2	2	0.0025
IL17	1070	ENSG0000016387	ZC3H12A	0.3	0.59	1.25	0.047	2	8.50E-26
IL17	1070	ENSG0000008322	ZCCHC6	0.07	-0.22	0.76	0.57	0.027	3.80E-25
IL17	1070	ENSG0000017504	ZDHHC14	0.23	0.16	0.7	0.18	2	5.40E-08
IL17	1070	ENSG0000018430	ZDHHC23	0.31	-0.6	0.91	0.42	2	0.0019
IL17	1070	ENSG0000018666	ZFP91	0.37	0.15	0.69	6.80E-15	0.017	2.20E-54
IL17	1070	ENSG0000013865	ZGRF1	0.34	-0.13	0.9	0.024	2	1.30E-12
IL17	1070	ENSG0000012251	ZMIZ2	0.56	0.07	1.24	1.00E-21	0.38	7.40E-108
IL17	1070	ENSG0000019702	ZNF100	0.17	0.13	0.7	0.42	2	1.50E-06
IL17	1070	ENSG0000019796	ZNF121	0.21	-0.16	0.66	0.054	0.19	1.10E-13
IL17	1070	ENSG0000019641	ZNF124	0.27	0.11	0.79	0.28	2	1.60E-05
IL17	1070	ENSG0000000580	ZNF195	0.22	-0.05	0.62	0.094	0.74	1.20E-09
IL17	1070	ENSG0000019713	ZNF257	0.28	0.23	0.78	0.41	2	0.0014
IL17	1070	ENSG0000018594	ZNF267	0.39	-0.19	1.27	3.60E-04	0.17	2.20E-42
IL17	1070	ENSG0000016524	ZNF367	0.31	-0.07	1.33	0.014	0.68	1.20E-39
IL17	1070	ENSG0000014252	ZNF473	0.13	0.18	0.63	0.33	0.16	3.20E-11
IL17	1070	ENSG0000019829	ZNF485	0.28	-0.1	0.83	0.34	2	1.30E-04
IL17	1070	ENSG0000022967	ZNF492	0.26	0.27	0.68	2	2	0.032
IL17	1070	ENSG0000020451	ZNF551	0.41	0.09	0.79	0.01	2	1.20E-08
IL17	1070	ENSG0000027746	ZNF670	0.21	-0.29	0.68	0.46	2	5.80E-04
IL17	1070	ENSG0000017304	ZNF680	0.16	0.06	0.72	0.44	2	2.40E-07
IL17	1070	ENSG0000019617	ZNF681	0.41	0.19	0.68	0.071	2	3.80E-04
IL17	1070	ENSG0000019608	ZNF724	0.31	0	0.66	0.36	2	0.012
IL17	1070	ENSG0000021396	ZNF726	-0.03	-0.12	0.85	0.95	2	4.10E-04
IL17	1070	ENSG0000021418	ZNF788	0.33	-0.09	1.04	0.011	2	7.80E-22
IL17	1070	ENSG0000019793	ZNF823	0.39	0.07	0.73	0.093	2	1.60E-04
IL17	1070	ENSG0000026704	ZNF850	0.16	0.08	0.62	0.6	2	0.0018
IL17	1070	ENSG0000017444	ZWILCH	0.24	-0.04	0.69	0.015	0.75	1.70E-16
IL17	1070	ENSG0000017392	3-Mar	0.37	0.22	1.37	0.011	2	2.10E-32
IL17	1070	ENSG0000014458	4-Mar	0.39	0.03	0.69	4.80E-05	0.84	3.50E-15

Table S10. Pathway analysis for 1292 and 1427 upregulated transcripts induced after stimulation with LIGHT in IL-13 or IL-17 pre-exposed human pulmonary fibroblasts, associated with Fig. 5.

Group _Pre-exposed	Category	Term	Count	%	PValue	Genes	List Total	Pop Hits	Pop Total	Fold Enrichment	Bonferroni	Benjamini	FDR
IL13	UP_KW_BIOLOGICAL_PROCESS	KW-0131~Cell cycle	156	12.97836938	3.1734474459	ERCC6L, ZWILCH, DSCC1, GMNN, CCNF, HJURP, BUB1B, MKI67, SMC4, SMC2, CDC20, PTTG1, RPS6KA1, CHEK1, NUSAP1, KNTC1, OIP5, PIM3, NEK2, PIM2, FBXO5, TP63, TIPIN, BORA, LIG1, VRK1, ESCO2, KNL1, ATAD3B, HASPIN, PIMREG, CDC25C, KNSTRN, CDC25A, SGO1, SGO2, MELK, CCNE2, FANCD2, CCNE1, TIMELESS, KIF20B, SEH1L, CDCA2, CDCA3, HEPACAM, CDCA5, CDCA8, NCAPG, PKMYT1, SKA3, NCAPH, SKA1, CCNB2, DSN1, CCNB1, RACGAP1, CLSPN, ECT2, FAM83D, FANCI, CDT1, UBE2C, GADD45A, DDIAS, RCC2, PLK1, CDC7, CDC6, ZWINT, NDC80, CYLD, ANLN, TPX2, KIF18B, NASP, STAG3, UBE2S, CDK2, SASS6, CDK1, TP73, SUV39H2, MCM7, MCM8, SUV39H1, DDX12P, NCAPG2, BRCA1, KIF11, FOXM1, BRCA2, CHTF18, CKS1B, CHAF1B, CHAF1A, NUF2, SPDL1, DLGAP5, CEP55, HELLS, CKAP2, HAUS6, KIF23, SMC1A, MASTL, CIT, CCNA2, ASPM, RBL1, DBF4, SAPCD2, POC5, INCENP, KIFC1, DMC1, MCM3, CKS2, BIRC5, MCM4, MCM5, KIF2C, MCM6,	764	690	11523	3.4099476439790575	4.3158885264673035E-43	4.3476230009266216E-43	3.903340358496164E-43
IL13	UP_KW_BIOLOGICAL_PROCESS	KW-0498~Mitosis	86	7.1547420965	4.9957156003	ERCC6L, ZWILCH, CCNF, NCAPG2, BUB1B, KIF11, SMC4, SMC2, CDC20, PTTG1, NUF2, NUSAP1, KNTC1, NEK2, OIP5, FBXO5, SPDL1, CEP55, HELLS, TIPIN, BORA, HAUS6, VRK1, KIF23, KNL1, SMC1A, MASTL, PIMREG, CDC25C, KNSTRN, CDC25A, CIT, SGO1, CCNA2, ASPM, MELK, SAPCD2, INCENP, KIFC1, TIMELESS, BIRC5, KIF2C, KIF20B, CDCA2, SEH1L, CDCA3, CDCA5, NCAPG, CDCA8, SKA3, CENPA, NCAPH, SKA1, AURKB, AURKA, CCNB2, DSN1, CCNB1, HAUS8, RBBP8, BUB3, FAM83D, BUB1, CDT1, CENPW, CENPX, SPAG5, UBE2C, RCC2, PLK1, CDC6, NDC80, ZWINT, CENPE, ANLN, TPX2, CENPF, MIS18A, KIF18B, CDK2, CDK1, TACC3, NCAPD3, SPC24, SPC25, MAD2L1	764	294	11523	4.411876269	6.794173216472927E-31	3.422065186238202E-31	3.0723650942138604E-31
IL13	GOTERM_BP_DIRECT	GO:0051301~cell division	92	7.6539101497	5.2134073117	ERCC6L, ZWILCH, CCNF, NCAPG2, KIF14, BUB1B, KIF11, SMC4, CKS1B, SMC2, CDC20, TUBA1C, TUBA1B, PTTG1, NUF2, KNTC1, NEK2, OIP5, FBXO5, SPDL1, HELLS, TIPIN, BORA, LIG1, HAUS6, VRK1, KNL1, ATAD3B, SMC1A, MASTL, PIMREG, CDC25C, KNSTRN, CDC25A, SGO1, CCNA2, ASPM, SGO2, CCNE2, CCNE1, KIFC1, CKS2, TIMELESS, BIRC5, MCM5, KIF2C, KIF20B, CDCA2, SEH1L, CDCA3, CDCA5, NCAPG, CDCA8, SKA3, NCAPH, SKA1, AURKB, AURKA, CCNB2, DSN1, CCNB1, HAUS8, RBBP8, BUB3, FAM83D, BUB1, CDT1, CENPW, CENPX, SPAG5, UBE2C, RCC2, PLK1, CDC7, CDC6, NDC80, ZWINT, CENPE, TPX2, CENPF, MIS18A, KIF18B, STAG3, UBE2S, PRC1, CDK2, CDK1, TACC3, NCAPD3, SPC24, SPC25, MAD2L1	1119	397	19478	4.033774308205194	2.2339450330853132E-27	2.2339450330853132E-27	2.19171643386013E-27

IL13	UP_KW_BIOLOGICAL_PROCESS	KW-0132~Cell division	100	8.3194675540	2.8751038020	ERCC6L, ZWILCH, CCNF, BUB1B, SMC4, SMC2, CDC20, PTTG1, NUSAP1, KNTC1, OIP5, NEK2, FBXO5, TIPIN, BORA, LIG1, VRK1, KNL1, ATAD3B, PIMREG, CDC25C, KNSTRN, CDC25A, SGO1, SGO2, MELK, CCNE2, CCNE1, TIMELESS, KIF20B, SEH1L, CDCA2, CDCA3, CDCA5, CDCA8, NCAPG, SKA3, NCAPH, SKA1, CCNB2, DSN1, CCNB1, RACGAP1, ECT2, FAM83D, CDT1, UBE2C, RCC2, PLK1, CDC7, CDC6, ZWINT, NDC80, ANLN, TPX2, KIF18B, STAG3, UBE2S, CDK2, CDK1, NCAPG2, KIF11, CKS1B, NUF2, SPDL1, CEP55, HELLS, HAUS6, KIF23, SMC1A, MASTL, CIT, CCNA2, ASPM, SAPCD2, INCENP, KIFC1, CKS2, BIRC5, MCM5, KIF2C, CENPA, AURKB, AURKA, HAUS8, RBBP8, BUB3, BUB1, CENPW, CENPX, SPAG5, CENPE, CENPF, MIS18A, PRC1, TACC3, NCAPD3, SPC24, SPC25, MAD2L1	764	421	11523	3.5825322406138467	3.910141170814272E-28	1.3129640696116551E-28	1.1787925588484203E-28
IL13	UP_KW_BIOLOGICAL_PROCESS	KW-0235~DNA replication	43	3.5773710482	5.6581525221	BLM, FEN1, PCNA, MCM7, MCM8, DSCC1, PRIM1, MCM10, CHTF18, POLD3, CHAF1B, ORC6, CHAF1A, CDC45, ORC1, POLD1, POLE, RFC5, GINS1, GINS2, CDT1, RFC3, RFC4, LIG1, RFC2, GINS3, DDX11, GINS4, CDC6, FAM111A, POLA1, POLA2, DBF4, NASP, GRWD1, POLE2, MCM3, MCM4, MCM5, MCM6, DNA2, DTL, MCM2	764	95	11523	6.826798015982364	7.695087430094931E-23	1.9379172388290543E-23	1.7398819005545525E-23
IL13	GOTERM_BP_DIRECT	GO:0006260~DNA replication	45	3.7437603993	5.7324610964	PIF1, BLM, FEN1, RNASEH2A, PCNA, MCM7, DSCC1, CHTF18, POLD3, RECQL4, CHAF1B, ORC6, CHAF1A, ORC1, POLD1, CHEK1, PCLAF, POLE, RFC5, GINS1, RFC3, RFC4, LIG1, RFC2, GINS3, DONSON, TICRR, FAM111B, FAM111A, POLA1, POLA2, DBF4, NASP, GRWD1, POLE2, POLE3, CDK2, CDK1, MCM4, MCM5, MCM6, DNA2, DTL, MCM2, ATR	1119	117	19478	6.694851172062968	2.456359579815055E-21	1.2281797899075274E-21	1.2049633224670352E-21
IL13	GOTERM_BP_DIRECT	GO:0007059~chromosome segregation	41	3.4109816971	8.1269661093	TOP2A, CDCA2, HJURP, TTK, BRCA1, MKI67, SKA3, SKA1, DSN1, NUF2, OIP5, NEK2, BUB1, DLGAP5, CENPU, CENPW, CENPX, SPAG5, ESCO2, KNSTRN, NDC80, SRPK1, SGO1, SGO2, CENPE, CENPF, DIAPH3, MIS18A, STAG3, INCENP, CENPI, CENPK, CENPL, BIRC5, CENPM, CENPN, KIF2C, CENPO, CEP85, SPC24, SPC25	1119	101	19478	7.066050841009035	3.4824049778751314E-20	1.1608016592917104E-20	1.1388588507963479E-20
IL13	KEGG_PATHWAY	hsa04110:Cell cycle	52	4.3261231281	2.3223750852	MCM7, BUB1B, CDC20, PTTG1, CHEK1, FBXO5, DDX11, ESCO2, KNL1, SMC1A, CDC25C, CDC25A, SGO1, CCNA2, RBL1, DBF4, CCNE2, ESPL1, CCNE1, MCM3, MCM4, MCM5, MCM6, MCM2, PCNA, CDCA5, TTK, DBF4B, PKMYT1, AURKB, CCNB2, CCNB1, ORC6, CDC45, ORC1, E2F1, E2F2, E2F3, BUB3, BUB1, CDT1, GADD45A, PLK1, CDC7, CDC6, NDC80, TICRR, CDK2, CDK1, TRIP13, ATR, MAD2L1	607	158	8534	4.627114049173149	7.663837781179573E-19	7.663837781179573E-19	7.060020259026031E-19
IL13	GOTERM_BP_DIRECT	GO:0006281~DNA repair	64	5.3244592346	1.7355258800	DCLRE1B, ERCC6L, FEN1, BRCA1, FOXM1, BRCA2, CHAF1B, CHAF1A, EME1, PTTG1, EXO1, CHEK1, PCLAF, TOPBP1, POLE, RFC5, WDHD1, RFC3, RFC4, LIG1, PARP1, PARP2, RFC2, DDX11, KIF22, SMC1A, MSH2, FANCD2, DMC1, TIMELESS, DNA2, INO80C, PIF1, BLM, PARPBP, DOT1L, RAD51AP1, RECQL4, BRIP1, POLD1, RBBP8, RAD54L, CLSPN, FANCI, BARD1, POLQ, RRM1, CENPX, FANCM, GADD45A, XRCC2, XRCC3, FANCA, FANCG, TICRR, POLA1, RAD51, UBE2T, POLE2, TDP1, CDK2, CDK1, RAD18, ATR	1119	303	19478	3.6766443400372206	7.43672839601822E-16	1.859182099004555E-16	1.8240376999335237E-16

IL13	GOTERM_BP_DIRECT	GO:0000070~mitotic sister chromatid segregation	22	1.8302828618	7.4467239774	SPAG5, PLK1, NCAPG2, CDCA8, KNL1, SMC1A, TUBG1, SKA3, SMC4, KNSTRN, NDC80, SKA1, ZWINT, KIF18A, KIF18B, ESPL1, KIFC1, CENPI, CENPK, NUSAP1, KNTC1, MAD2L1	1119	35	19478	10.94129962977148	3.190921224351793E-14	6.381842448703587E-15	6.261205520268349E-15
IL13	UP_KW_BIOLOGICAL_PROCESS	KW-0227~DNA damage	82	6.8219633943	1.2363228904	DCLRE1B, FEN1, ANKLE1, MCM8, WDR4, TONSL, MCM10, BRCA1, FOXM1, BRCA2, CHAF1B, CHAF1A, EME1, PTTG1, EXO1, ZC3H12A, CHEK1, PCLAF, TOPBP1, IKBKE, POLE, GEN1, MSH5-SAPCD1, TIPIN, LIG1, PARP1, ZGRF1, PARP2, DDX11, AEN, SMC1A, TRAIP, MMS22L, NEIL3, SLFN11, MSH2, FANCD2, TIMELESS, DNA2, DTL, INO80C, PIF1, BLM, PARPBP, PCNA, PSMD14, UHRF1, NPAS2, RAD54B, RAD51AP1, POLD3, BRIP1, POLD1, RBBP8, RAD54L, CLSPN, E2F7, FANCI, BARD1, POLQ, MGME1, EGLN3, SLF1, CENPX, ATAD5, FANCM, GADD45A, XRCC2, XRCC3, FANCA, PALB2, FANCG, TICRR, FAM111A, RAD51, UBE2T, TDP1, RFWD3, CDK2, AUNIP, RAD18, ATR	764	443	11523	2.791787314006122	1.6813991309939878E-15	3.3875247197967108E-16	3.0413543104744195E-16
IL13	KEGG_PATHWAY	hsa03030:DNA replication	23	1.9134775374	5.2496477216	RFC5, FEN1, RFC3, RFC4, RNASEH2A, PCNA, LIG1, MCM7, RFC2, PRIM1, POLD3, POLA1, POLA2, POLD1, POLE2, POLE3, MCM3, MCM4, MCM5, DNA2, MCM6, POLE, MCM2	607	36	8534	8.982335712978216	1.7323837481551966E-14	8.661918740775983E-15	7.979464536957268E-15
IL13	GOTERM_BP_DIRECT	GO:0000278~mitotic cell cycle	38	3.1613976705	6.2157366747	CDCA5, NOLC1, CDCA8, KIF11, PKMYT1, SKA3, SKA1, AURKB, AURKA, KIF15, TUBA1C, TUBA1B, MYB, PBK, NEK2, MYBL2, NDP, NUDT15, POLE, MYBL1, CDT1, WDHD1, CENPW, XRCC2, PLK1, KIF22, TUBB4B, TUBG1, HASPIN, NDC80, CIT, CENPE, TPX2, TUBB2B, CENPF, KIF18B, INCENP, BIRC5	1119	135	19478	4.899639228146825	2.8543833963112775E-12	4.43907194188408E-13	4.3551594967749527E-13
IL13	GOTERM_BP_DIRECT	GO:0007094~mitotic spindle assembly checkpoint signaling	19	1.5806988352	1.8387363070	ZWILCH, PLK1, BUB1B, TTK, HASPIN, NDC80, ZWINT, CDC20, CENPF, NUF2, BIRC5, KNTC1, BUB3, TRIP13, SPDL1, BUB1, SPC24, MAD2L1, SPC25	1119	30	19478	11.024188263330354	8.087419622881953E-12	1.1255692965480746E-12	1.1042924907090096E-12
IL13	GOTERM_BP_DIRECT	GO:0006270~DNA replication initiation	18	1.4975041597	3.4557751609	MCM7, PRIM1, MCM10, NOC3L, CDC6, POLA1, POLA2, ORC6, CDC45, ORC1, CCNE2, CCNE1, MCM3, MCM4, MCM5, TOPBP1, MCM6, MCM2	1119	27	19478	11.604408698242478	1.4747647547608267E-11	1.8509995705764835E-12	1.816009847072004E-12
IL13	UP_KW_BIOLOGICAL_PROCESS	KW-0234~DNA repair	69	5.7404326123	3.5083191405	DCLRE1B, FEN1, ANKLE1, MCM8, TONSL, BRCA1, FOXM1, BRCA2, CHAF1B, CHAF1A, EME1, PTTG1, EXO1, CHEK1, PCLAF, TOPBP1, POLE, GEN1, MSH5-SAPCD1, LIG1, PARP1, ZGRF1, PARP2, DDX11, SMC1A, TRAIP, MMS22L, NEIL3, MSH2, FANCD2, TIMELESS, DNA2, INO80C, PIF1, BLM, PARPBP, PCNA, PSMD14, UHRF1, RAD54B, RAD51AP1, POLD3, BRIP1, POLD1, RBBP8, RAD54L, CLSPN, FANCI, BARD1, POLQ, MGME1, SLF1, CENPX, FANCM, XRCC2, XRCC3, FANCA, PALB2, FANCG, TICRR, FAM111A, RAD51, UBE2T, TDP1, RFWD3, CDK2, AUNIP, RAD18, ATR	764	366	11523	2.8434147283494977	4.831690603168681E-13	8.010662037593138E-14	7.192054238131066E-14
IL13	KEGG_PATHWAY	hsa04668:TNF signaling pathway	35	2.9118136439	7.7440641017	CXCL6, CSF2, CSF1, TNFAIP3, PIK3R3, CXCL1, CXCL3, CXCL2, CXCL5, CX3CL1, ICAM1, CCL2, JUNB, MAP3K5, VCAM1, MLKL, IL15, RHBDF2, DAB2IP, LIF, VEGFC, TRAF2, TRAF1, TNFRSF1B, MMP9, NFKBIA, CYLD, IL6, TRAF3, IRF1, FAS, MAP3K14, BIRC2, IL18R1, BIRC3	607	119	8534	4.135090609555189	2.555455846930954E-10	8.518470511887439E-11	7.847318289738731E-11

IL13	GOTERM_BP_DIRECT	GO:0006954~inflammatory response	65	5.40765391	2.5999648938	CXCL6, NRROS, CXCL8, CSF1, GPR68, TNFAIP3, CXCL1, CXCL3, CXCL2, CXCL5, CX3CL1, ADGRE2, CRHBP, CYP26B1, IL18RAP, ADGRE5, ZC3H12A, NAMPT, OLR1, BDKRB1, TNFRSF4, PTGIR, SPHK1, NFAM1, PLA2G4C, TNFRSF1B, TLR1, ELF3, COL6A1, CHI3L1, TLR10, ADAM8, TLR6, MGLL, CHST2, IDO1, EPHA2, TLR2, PTGER4, SEMA7A, HDAC9, FUT4, RELB, C3, IL1RL1, IL1RL2, IRAK2, CCL2, NDP, GGT5, VCAM1, KDM4D, IL34, DAB2IP, NFKB2, FOSL1, MFHAS1, BMP2, IL6, VNN1, NLRP10, TNIP3, TNFSF4, IL18R1, NFKBIB	1119	432	19478	2.619050574256115	1.1140658284780613E-8	1.2378721744423463E-9	1.2144724903980453E-9
IL13	GOTERM_BP_DIRECT	GO:0031297~replication fork processing	18	1.4975041597	6.4591977948	GEN1, POLQ, BLM, TIPIN, PCNA, FANCM, CENPX, DDX11, TONSL, DONSON, TRAIP, MMS22L, FAM111A, RAD51, EME1, RFWD3, TIMELESS, ATR	1119	38	19478	8.245237759	2.7677528269265395E-8	2.7677662551128496E-9	2.715446752974193E-9
IL13	GOTERM_BP_DIRECT	GO:0006974~DNA damage response	51	4.2429284525	4.3484307402	TOP2A, ANKLE1, MCM7, MCM8, SUV39H1, WDR4, MCM10, BRCA1, ZC3H12A, CHEK1, PCLAF, FBXO5, TOPBP1, TP63, NKX3-1, TIPIN, PARP1, PARP2, PRMT1, DDX11, VRK1, MASTL, ATAD3A, WDR76, TRAIP, SLFN11, TIMELESS, DTL, BLM, UHRF1, NPAS2, RAD51AP1, BRIP1, PMAIP1, BARD1, POLQ, EGLN3, TIGAR, SLF1, GADD45A, XRCC3, FANCG, FAM111A, RAD51, UBE2T, RFWD3, POLE3, CDK1, RAD18, ATR, TP73	1119	313	19478	2.836221295257347	1.863303249471926E-7	1.693911429265731E-8	1.661891166542155E-8
IL13	GOTERM_BP_DIRECT	GO:0000727~double-strand break repair via break-induced replication	10	0.8319467554	1.2323978553	GINS2, CDC45, MCM7, GINS4, MCM3, MCM4, CDC7, MCM5, MCM6, MCM2	1119	12	19478	14.505510872803097	5.280810848828921E-6	4.400687341877602E-7	4.3175004866402423E-7
IL13	GOTERM_BP_DIRECT	GO:0007052~mitotic spindle organization	19	1.5806988352	1.4642068731	STIL, PLK2, PLK1, TTK, WDR62, KIF11, TUBG1, NDC80, AURKB, AURKA, FAM110A, CENPE, CCNB1, KIF4A, NUF2, TACC3, BIRC5, DLGAP5, SPC25	1119	57	19478	5.802204349121239	6.274106746806396E-6	4.826251116592475E-7	4.735019765263655E-7
IL13	GOTERM_BP_DIRECT	GO:0000724~double-strand break repair via homologous recombination	24	1.9966722129	2.1696219994	GEN1, BLM, SLF1, FEN1, ANKLE1, PSMD14, KDM4D, MCM8, UHRF1, XRCC2, XRCC3, TONSL, BRCA1, BRCA2, PALB2, RAD54B, RAD51AP1, RECQL4, MMS22L, RAD51, RFWD3, RBBP8, AUNIP, TOPBP1	1119	105	19478	3.9786544108259925	9.29639824729378E-5	6.640593048464226E-6	6.5150649184932575E-6
IL13	KEGG_PATHWAY	hsa03440:Homologous recombination	16	1.3311148086	5.5464071943	BARD1, BLM, XRCC2, XRCC3, BRCA1, BRCA2, PALB2, RAD54B, POLD3, BRIP1, RAD51, EME1, POLD1, RBBP8, RAD54L, TOPBP1	607	41	8534	5.486559247800056	1.8302976730000253E-5	4.575785935311962E-6	4.215269467681322E-6
IL13	KEGG_PATHWAY	hsa05222:Small cell lung cancer	24	1.9966722129	8.3146914764	LAMB3, GADD45A, ITGA2, PIK3R3, LAMC2, TRAF2, TRAF1, CKS1B, NFKBIA, CCNE2, TRAF4, CCNE1, TRAF3, COL4A4, CDK2, CKS2, E2F1, E2F2, CYCS, ITGAV, ITGA6, E2F3, BIRC2, BIRC3	607	93	8534	3.628208534835521	2.7438106592225786E-5	5.487696374474375E-6	5.055332417697605E-6
IL13	GOTERM_BP_DIRECT	GO:0000281~mitotic cytokinesis	18	1.4975041597	9.5892257685	PLK1, CKAP2, CDCA8, KIF23, CENPA, AURKB, CIT, ANLN, ESPL1, RACGAP1, INCENP, KIF4A, NUSAP1, TRIM36, BIRC5, KIF20A, ECT2, CEP55	1119	65	19478	4.820292843885337	4.1081393673902955E-4	2.7393221612020476E-5	2.6875403420521367E-5
IL13	BIOCARTA	h_mcmPathway:CDK Regulation of DNA Replication	12	0.9983361064	1.1460661901	CDT1, ORC6, MCM7, ORC1, CCNE1, CDK2, MCM3, MCM4, MCM5, CDC6, MCM6, MCM2	162	18	1622	6.674897119341564	2.2577250361477397E-5	2.246289732661496E-5	2.2119077469574934E-5
IL13	GOTERM_BP_DIRECT	GO:0000082~G1/S transition of mitotic cell cycle	20	1.6638935108	1.4034380904	BTN2A2, PLK2, CCNF, CDC7, IQGAP3, CDC25A, CCNA2, CCNB2, CCNB1, DBF4, CCNE2, CCNE1, CDK2, RBBP8, CDK1, E2F3, PIM2, POLE, EZH2, CDKN3	1119	82	19478	4.245515377405784	6.011924751853082E-4	3.7585826358581626E-5	3.6875335825315556E-5
IL13	KEGG_PATHWAY	hsa03410:Base excision repair	16	1.3311148086	1.6448076055	RFC5, FEN1, RFC3, RFC4, PCNA, LIG1, PARP1, PARP2, RFC2, POLD3, NEIL3, POLD1, POLE2, TDP1, POLE3, POLE	607	44	8534	5.1124756627227805	5.427718239148227E-5	9.046441830639745E-6	8.333691868225705E-6

IL13	GOTERM_BP_DIRECT	GO:0034080~CENP-A containing chromatin assembly	8	0.6655574043	2.0753126966	MIS18A, CENPW, NASP, CENPI, HJURP, OIP5, CENPN, CENPA	1119	10	19478	13.925290437890975	8.888762982526277E-4	5.231008767851846E-5	5.13212622171509E-5
IL13	UP_KW_BIOLOGICAL_PROCESS	KW-0159~Chromosome partition	16	1.3311148086	2.5045871694	SEH1L, KNL1, SRPK1, SGO1, SGO2, DSN1, ESPL1, STAG3, PTTG1, INCENP, BIRC5, NEK2, KIF2C, BUB3, CEP85, BUB1	764	48	11523	5.027486910994765	3.4061809659369224E-5	4.901834888733449E-6	4.4009174548482794E-6
IL13	GOTERM_BP_DIRECT	GO:0030174~regulation of DNA-templated DNA replication initiation	9	0.7487520798	2.5302468652	CDT1, MCM7, GMNN, MCM3, MCM4, MCM5, MCM6, MCM2, TICRR	1119	14	19478	11.189965530448104	0.001083623	6.0233932320431155E-5	5.909532123106011E-5
IL13	GOTERM_BP_DIRECT	GO:0007080~mitotic metaphase chromosome alignment	15	1.2479201331	2.9804413538	SEH1L, CDCA5, KIF14, CDCA8, KIF22, SKA3, SKA1, CENPE, KIF18A, CCNB1, KIFC1, NUP62, RRS1, KIF2C, SPDL1	1119	48	19478	5.439566577301162	0.001276304	6.721679579692762E-5	6.594618658816424E-5
IL13	GOTERM_BP_DIRECT	GO:0007051~spindle organization	10	0.8319467554	3.5843680973	ASPM, SPAG5, TRIM36, CEP72, TTK, AUNIP, KIF11, KNSTRN, AURKB, AURKA	1119	19	19478	9.161375288086166	0.001534723	7.679508648541022E-5	7.53434174059894E-5
IL13	GOTERM_BP_DIRECT	GO:0033209~tumor necrosis factor-mediated signaling pathway	16	1.3311148086	3.8680865413	EIF5A, TNFSF18, EIF5AL1, TRAF2, TRAF1, TNFRSF1B, TNFSF13B, NFKBIA, KRT18, TRAF3, TIFA, FAS, TNFSF11, TNFRSF4, BIRC2, BIRC3	1119	56	19478	4.973318013532491	0.001656103	7.892738490343696E-5	7.743540866605578E-5
IL13	GOTERM_BP_DIRECT	GO:0051256~mitotic spindle midzone assembly	8	0.6655574043	5.4228529537	RACGAP1, INCENP, PRC1, KIF4A, CDCA8, BIRC5, KIF23, AURKB	1119	11	19478	12.659354943537249	0.002320995	1.0562238594039771E-4	1.0362579008014748E-4
IL13	KEGG_PATHWAY	hsa04115:p53 signaling pathway	20	1.6638935108	7.4888567105	RRM2, CD82, GADD45A, SERPINE1, TNFRSF10B, TNFRSF10A, CCNB2, CCNB1, CCNE2, CCNE1, CHEK1, CDK2, CDK1, FAS, PMAIP1, CYCS, BID, GTSE1, ATR, TP73	607	75	8534	3.7491488193300384	2.471018292901084E-4	3.220291297046833E-5	2.9665713766734463E-5
IL13	KEGG_PATHWAY	hsa05169:Epstein-Barr virus infection	36	2.9950083194	7.8067667807	PSMD14, TNFAIP3, PIK3R3, RELB, ICAM1, E2F1, E2F2, E2F3, HES1, CD58, BID, JAK3, IKBKE, IFNAR2, GADD45A, HLA-B, TAP2, TAP1, TRAF2, HLA-F, TAPBP, NFKB2, NFKBIA, CCNA2, IL6, CCNE2, CCNE1, TRAF3, OAS3, CDK2, FAS, CYCS, NFKBIE, MAP3K14, NFKBIB, TLR2	607	204	8534	2.481054365733114	2.575902222672344E-4	3.220291297046833E-5	2.9665713766734463E-5
IL13	GOTERM_BP_DIRECT	GO:0036297~interstrand cross-link repair	13	1.0815307820	8.1120492771	FANCI, DCLRE1B, FANCM, CENPX, XRCC3, FANCA, FANCG, RAD51AP1, NEIL3, RAD51, FANCD2, RFWD3, ATR	1119	38	19478	5.954893937256008	0.00346998	1.4738335165473247E-4	1.445973419735111E-4
IL13	GOTERM_BP_DIRECT	GO:0000086~G2/M transition of mitotic cell cycle	15	1.2479201331	8.8099713676	WNT10B, PLK1, PKMYT1, FOXM1, MASTL, CDC25C, CDC25A, AURKA, CCNA2, CCNB1, MELK, CHEK1, CDK2, CDK1, BIRC5	1119	52	19478	5.021138379047226	0.003767958	1.4738335165473247E-4	1.445973419735111E-4
IL13	GOTERM_BP_DIRECT	GO:0006261~DNA-templated DNA replication	14	1.1647254575	8.9298353867	RFC5, POLQ, WDHD1, GINS2, RFC3, RFC4, RFC2, GINS4, BAZ1A, POLD3, POLD1, POLE2, POLE3, POLE	1119	45	19478	5.415390725846491	0.003819125	1.4738335165473247E-4	1.445973419735111E-4
IL13	GOTERM_BP_DIRECT	GO:0006271~DNA strand elongation involved in DNA replication	7	0.5823627287	8.9427471249	POLD3, POLA1, RFC3, RFC4, MCM7, MCM3, MCM4	1119	8	19478	15.230786416443255	0.003824636	1.4738335165473247E-4	1.445973419735111E-4
IL13	GOTERM_BP_DIRECT	GO:0051310~metaphase chromosome alignment	9	0.7487520798	9.7770298878	CENPE, CENPF, INCENP, KIF2C, KIF22, FAM83D, SKA3, NDC80, SKA1	1119	16	19478	9.791219839	0.004180696	1.5516508544183408E-4	1.5223197647548902E-4
IL13	GOTERM_BP_DIRECT	GO:0090267~positive regulation of mitotic cell cycle spindle assembly checkpoint	8	0.6655574043	1.2367137525	GEN1, INCENP, XRCC3, CDCA8, BIRC5, NDC80, AURKB, MAD2L1	1119	12	19478	11.604408698242478	0.005285305	1.892613724822357E-4	1.8568373626961935E-4

IL13	GOTERM_BP_DIRECT	GO:0090307~mitotic spindle assembly	14	1.1647254575	1.5455861186	UHRF1, CDCA8, KIF11, SMC1A, MZT1, AURKB, KIF15, CDC20, TPX2, INCENP, KIFC1, BIRC5, NEK2, MYBL2	1119	47	19478	5.1849485672998314	0.006600959	2.28373673048348E-4	2.240566911307479E-4
IL13	GOTERM_BP_DIRECT	GO:0010971~positive regulation of G2/M transition of mitotic cell cycle	11	0.9151414309	1.8911589931	WNT10B, RRM1, CCNB1, RCC2, CDK1, CDC7, DBF4B, FBXO5, CDC25C, DTL, CDC25A	1119	28	19478	6.838312268607175	0.008070878	2.701205428577233E-4	2.6501441357616533E-4
IL13	GOTERM_BP_DIRECT	GO:0000076~DNA replication checkpoint signaling	8	0.6655574043	2.5463032242	CDT1, TIPIN, CDC45, TIMELESS, TOPBP1, DNA2, CDC6, CLSPN	1119	13	19478	10.71176187530075	0.010851614983126368	3.4169832262232006E-4	3.3523914779562045E-4
IL13	GOTERM_BP_DIRECT	GO:1900264~positive regulation of DNA-directed DNA polymerase activity	7	0.5823627287	2.5517727710	RFC5, RFC3, RFC4, PCNA, RFC2, DSCC1, CHTF18	1119	9	19478	13.538476814616226	0.010874797449270335	3.4169832262232006E-4	3.3523914779562045E-4
IL13	KEGG_PATHWAY	hsa03460:Fanconi anemia pathway	16	1.3311148086	4.0841721076	FANCI, BLM, FANCM, CENPX, FANCA, BRCA1, BRCA2, PALB2, FANCG, BRIP1, RAD51, EME1, FANCD2, UBE2T, HES1, ATR	607	55	8534	4.089980530178224	0.001346872	1.497529772788965E-4	1.3795425785692285E-4
IL13	GOTERM_BP_DIRECT	GO:0051984~positive regulation of chromosome segregation	8	0.6655574043	4.8395817620	NCAPG2, NCAPG, NCAPD3, CDC6, SMC4, RAD18, NCAPH, SMC2	1119	14	19478	9.946636027064981	0.020524111499833686	6.284123591001929E-4	6.165333856842966E-4
IL13	GOTERM_BP_DIRECT	GO:0051315~attachment of mitotic spindle microtubules to kinetochore	8	0.6655574043	8.6237446656	CDT1, CENPE, SEH1L, NUF2, KIF2C, SKA3, NDC80, SKA1	1119	15	19478	9.283526958593981	0.036278479	0.001070841	0.001050598
IL13	KEGG_PATHWAY	hsa04210:Apoptosis	26	2.1630615640	8.6317376489	PIK3R3, ITPR2, LMNB2, CTSS, LMNB1, TUBA1C, TUBA1B, PMAIP1, BID, MAP3K5, PARP1, GADD45A, PARP2, DAB2IP, TNFRSF10B, TRAF2, TNFRSF10A, TRAF1, ERN1, NFKBIA, BIRC5, FAS, CYCS, MAP3K14, BIRC2, BIRC3	607	136	8534	2.6878088962108735	0.002844433	2.848473424157809E-4	2.6240482452847695E-4
IL13	GOTERM_BP_DIRECT	GO:0007018~microtubule-based movement	17	1.4143094841	8.7466570506	DNAH11, KIF14, KIF24, KIF23, KIF11, KIF22, KIF15, CENPE, KIF18A, KIF18B, KIFC1, KIF4A, KIF2C, KIF20A, KIF1A, KIF21B, KIF20B	1119	79	19478	3.7457268582934575	0.036785923	0.001070841	0.001050598
IL13	KEGG_PATHWAY	hsa04064:NF-kappa B signaling pathway	22	1.8302828618	1.1830415627	VCAM1, CXCL8, PARP1, GADD45A, TNFAIP3, CXCL1, TRAF2, TRAF1, CXCL3, CXCL2, TNFSF13B, RELB, NFKB2, ICAM1, NFKBIA, CYLD, PLAU, TRAF3, TNFSF11, MAP3K14, BIRC2, BIRC3	607	105	8534	2.945759786616459	0.003896449	3.468931213101025E-4	3.195621481159732E-4
IL13	KEGG_PATHWAY	hsa04060:Cytokine-cytokine receptor interaction	43	3.5773710482	1.2614295320	CXCL6, CNTF, CSF2, CXCL8, CSF1, EBI3, CXCL1, CXCL3, CXCL2, CXCL5, CX3CL1, TNFSF13B, IL27RA, IL1RL1, IL1RL2, IL18RAP, CCL2, TNFSF11, TNFRSF8, RELT, TNFRSF4, IL32, TNFSF18, IFNAR2, IL15RA, IL33, TNFRSF12A, TSLP, IL15, IL34, TNFRSF9, LIF, TNFRSF10B, LIFR, TNFRSF10A, TNFRSF1B, GDF6, GDF5, BMP2, IL6, TNFSF4, FAS, IL18R1	607	298	8534	2.028692104419358	0.004154092	3.468931213101025E-4	3.195621481159732E-4
IL13	GOTERM_BP_DIRECT	GO:0034501~protein localization to kinetochore	7	0.5823627287	1.2699154696	ZWILCH, CDK1, TTK, BUB3, KNL1, SPDL1, AURKB	1119	11	19478	11.076935575595094	0.052962155	0.001470699	0.001442899
IL13	GOTERM_BP_DIRECT	GO:0051988~regulation of attachment of spindle microtubules to kinetochore	7	0.5823627287	1.2699154696	SPAG5, RACGAP1, ZWILCH, KNTC1, NEK2, ECT2, KNSTRN	1119	11	19478	11.076935575595094	0.052962155	0.001470699	0.001442899

IL13	KEGG_PATHWAY	hsa03430:Mismatch repair	10	0.8319467554	1.4318581658	RFC5, POLD3, RFC3, RFC4, PCNA, MSH2, LIG1, EXO1, RFC2, POLD1	607	23	8534	6.112742640212019	0.00471402	3.6347168824795226E-4	3.3483452493144694E-4
IL13	GOTERM_BP_DIRECT	GO:0006302~double-strand break repair	17	1.4143094841	1.4392241582	POLQ, FEN1, PARP1, ZGRF1, PARP2, CDCA5, PLK1, BRCA1, ESCO2, BRCA2, BRIP1, EME1, MSH2, TDP1, NSD2, TRIP13, ATR	1119	82	19478	3.6086880707949165	0.059808028	0.001622915	0.001592236
IL13	GOTERM_BP_DIRECT	GO:0007346~regulation of mitotic cell cycle	15	1.2479201331	1.5093150364	PLK1, GMNN, MASTL, CKS1B, CDC20, CYLD, CDK2, CKS2, BIRC5, PIM3, PIM2, TTLL12, FBXO5, DLGAP5, TP73	1119	65	19478	4.016910703237781	0.0626276	0.001658312	0.001626964
IL13	GOTERM_BP_DIRECT	GO:0000079~regulation of cyclin-dependent protein serine/threonine kinase activity	10	0.8319467554	1.6749282909	BLM, CCNE2, GADD45A, CDC6, PKMYT1, CDC25C, CDC25A, GTPBP4, CDK5R1, CDKN3	1119	28	19478	6.2166475169156135	0.069256247	0.001794267	0.00176035
IL13	GOTERM_BP_DIRECT	GO:0007088~regulation of mitotic nuclear division	9	0.7487520798	1.7819768121	CDCA2, BORA, NEK2, FBXO5, MKI67, PKMYT1, KIF20B, CDC25C, FBXO43	1119	22	19478	7.120887155739703	0.073515893	0.001862383	0.001827178
IL13	GOTERM_BP_DIRECT	GO:0008608~attachment of spindle microtubules to kinetochore	8	0.6655574043	2.3542566317	SGO1, DSN1, NUF2, BUB3, KNL1, NDC80, SPC24, SPC25	1119	17	19478	8.191347316406455	0.095959469	0.002396443	0.002351143
IL13	GOTERM_BP_DIRECT	GO:0006139~nucleobase-containing compound metabolic process	11	0.9151414309	2.4048318088	PRPS2, BRIP1, PNP, TPMT, OAS3, DDX11, DDX12P, CTPS1, TK1, SLC29A1, ATR	1119	36	19478	5.318687320027803	0.097916582	0.002396443	0.002351143
IL13	GOTERM_BP_DIRECT	GO:0051382~kinetochore assembly	7	0.5823627287	4.2688989139	CENPE, CENPF, CENPW, CENPX, CENPK, CENPA, DLGAP5	1119	13	19478	9.372791640888156	0.16717040282394413	0.004157325	0.004078739
IL13	GOTERM_BP_DIRECT	GO:1902975~mitotic DNA replication initiation	5	0.4159733777	5.1524806812	POLA1, GINS3, MCM3, MCM4, MCM2	1119	5	19478	17.406613047363717	0.1981145288036149	0.004906307	0.004813562
IL13	GOTERM_BP_DIRECT	GO:0007076~mitotic chromosome condensation	8	0.6655574043	5.5108171742	CDCA5, PLK1, NUSAP1, NCAPG, NCAPD3, SMC4, NCAPH, SMC2	1119	19	19478	7.329100230468934	0.21033385391813963	0.005133446	0.005036408
IL13	UP_KW_BIOLOGICAL_PROCESS	KW-0395~Inflammatory response	29	2.4126455906	6.3271253499	SEMA7A, CXCL8, CSF1, TNFAIP3, CXCL1, CXCL3, CXCL2, FUT4, CX3CL1, C3, ADGRE2, IL1RL2, IL18RAP, ZC3H12A, CCL2, OLR1, IL34, DAB2IP, TLR1, MFHAS1, ELF3, NLRP10, TNIP3, CHI3L1, TLR10, TLR6, CHST2, IL18R1, TLR2	764	192	11523	2.2780800065445024	0.008568244	0.00108352	9.727955225485982E-4
IL13	GOTERM_BP_DIRECT	GO:1905821~positive regulation of chromosome condensation	6	0.4991680532	6.4061257665	NCAPG2, NCAPG, NCAPD3, SMC4, NCAPH, SMC2	1119	9	19478	11.60440869824248	0.2400565694517509	0.005840478	0.005730075
IL13	GOTERM_BP_DIRECT	GO:0006913~nucleocytoplasmic transport	12	0.9983361064	6.7200998965	NDC1, NUP205, NUP107, SEH1L, NUP188, NUP155, NUP85, NUP50, NUP62, NUP35, NUP153, NUP88	1119	48	19478	4.351653261840929	0.25021288279867837	0.005999089	0.005885687
IL13	KEGG_PATHWAY	hsa05166:Human T-cell leukemia virus 1 infection	33	2.7454242928	1.0547702443	CSF2, PIK3R3, BUB1B, ADCY1, ETS1, RELB, ICAM1, CDC20, CCNB2, PTTG1, CHEK1, E2F1, E2F2, E2F3, BUB3, JAK3, IL15RA, EGR2, IL15, HLA-B, HLA-F, NFKB2, NFKBIA, CCNA2, FOSL1, IL6, ESPL1, CCNE2, CCNE1, CDK2, MAP3K14, ATR, MAD2L1	607	224	8534	2.0712373499646977	0.034210381	0.002320866	0.00213801
IL13	KEGG_PATHWAY	hsa04218:Cellular senescence	26	2.1630615640	1.0549392841	CXCL8, SERPINE1, PIK3R3, ITPR2, LIN9, FOXM1, ETS1, CCNB2, CCNB1, CHEK1, E2F1, E2F2, E2F3, MYBL2, GADD45A, HLA-B, HLA-F, CDC25A, CCNA2, IL6, RBL1, CCNE2, CCNE1, CDK2, CDK1, ATR	607	157	8534	2.3282930565903106	0.034215768722432816	0.002320866	0.00213801

IL13	GOTERM_BP_DIRECT	GO:0035825~homologous recombination	6	0.4991680532	1.2205438030	BARD1, BRIP1, UHRF1, RBBP8, BRCA1, TOPBP1	1119	10	19478	10.443967828418232	0.407281051	0.010673531012420792	0.010471767649058814
IL13	GOTERM_BP_DIRECT	GO:0071479~cellular response to ionizing radiation	11	0.9151414309	1.2570221654	NET1, BARD1, RAD51AP1, BLM, RAD51, KDM4D, GADD45A, FIGNL1, BRCA1, BRCA2, ECT2	1119	43	19478	4.452854500488393	0.4164749248352778	0.010772679957584828	0.010569042366788007
IL13	GOTERM_BP_DIRECT	GO:0051726~regulation of cell cycle	30	2.4958402662	1.3316067618	INO80C, HEPACAM, CCNF, DOT1L, BRCA1, LIN9, FOXM1, FIGNL1, PCLAF, E2F2, JUNB, BARD1, PNPT1, DDIAS, GADD45A, PLK1, DAB2IP, MASTL, WDR12, BOP1, RBL1, IRF1, TRIM36, KIF20B, PRR11, DTL, BIRC2, CDKN3, BIRC3, TP73	1119	241	19478	2.1667983046510852	0.43483157286027674	0.011188107793061222	0.010976617307358081
IL13	UP_KW_BIOLOGICAL_PROCESS	KW-0690~Ribosome biogenesis	19	1.5806988352	1.4694304825	NOP56, UTP15, LTV1, NOP58, UTP4, PAK1IP1, NOP2, GNL2, WDR12, GTPBP4, WDR43, IPO4, RCL1, BOP1, MYBBP1A, DKC1, MRTO4, RRS1, HEATR3	764	104	11523	2.755449556987515	0.019787332	0.0022368	0.002008222
IL13	GOTERM_BP_DIRECT	GO:0001525~angiogenesis	32	2.6622296173	1.5407608848	ROBO4, FLT1, CXCL8, SERPINE1, TNFAIP2, NRXN3, THY1, TYMP, ARHGAP22, DLL4, ADGRG1, ESM1, ZC3H12A, CCL2, ITGAV, NDP, EPHB1, COL27A1, TNFRSF12A, ANXA2, C1GALT1, ERAP1, DAB2IP, VEGFC, PLXDC1, APLN, COL8A2, MMP19, ADAM8, CSPG4, NAA15, EPHA2	1119	267	19478	2.086185833391906	0.4832865979003671	0.012482067	0.012246116986043745
IL13	GOTERM_BP_DIRECT	GO:0042254~ribosome biogenesis	11	0.9151414309	1.5438729787	NOP56, LTV1, RCL1, BOP1, MYBBP1A, GRWD1, DHX37, URB2, NOC4L, GNL2, GTPBP4	1119	44	19478	4.351653262	0.4839752987827106	0.012482067	0.012246116986043745
IL13	GOTERM_BP_DIRECT	GO:0043254~regulation of protein-containing complex assembly	8	0.6655574043	1.6022891599	TNFSF18, HSPA8, HSP90AA1, DAB2IP, HJURP, HES1, TRAF2, IKBKE	1119	22	19478	6.329677471768624	0.496733746	0.012714461204733156	0.012474118
IL13	GOTERM_BP_DIRECT	GO:0008284~positive regulation of cell population proliferation	51	4.2429284525	1.7332449146	CNTF, FLT1, CSF2, CSF1, BNC1, KIF14, LAMC2, HTR2A, FOXM1, CXCL5, CX3CL1, IL27RA, ESM1, EDNRB, BCL7A, NAMPT, ITGAV, FBXO5, NKX3-1, TIPIN, IL15, PRMT1, SPHK1, LIFR, NRG1, WDR77, SAPCD2, BIRC5, KIF20B, HBEGF, EBI3, NOP2, TTK, DBF4B, HAS2, E2F3, HES1, TSLP, IL34, NTRK3, LIF, VEGFC, CDC7, FOSL1, BMP2, IL6, GDNF, PRC1, TNFSF4, CDK2, EZH2	1119	512	19478	1.7338618465147455	0.5242010162633424	0.013503553562299856	0.013248293856688121
IL13	KEGG_PATHWAY	hsa05168:Herpes simplex virus 1 infection	28	2.3294509151	1.9255110067	PIK3R3, IFIH1, C3, CCL2, BID, IKBKE, IFNAR2, CD74, HLA-B, TAP2, TAP1, TRAF2, POU2F2, HLA-F, SRPK1, TAPBP, NFKBIA, IL6, TRAF3, OAS3, SRSF2, FAS, CYCS, SRSF7, BIRC2, BIRC3, TLR2, NECTIN1	607	182	8534	2.162970472690407	0.061570909049791345	0.003971366	0.003658471
IL13	GOTERM_BP_DIRECT	GO:1905820~positive regulation of chromosome separation	6	0.4991680532	2.1318373498	NCAPG2, NCAPG, NCAPD3, SMC4, NCAPH, SMC2	1119	11	19478	9.494516207652937	0.5989181224456546	0.015638475999801546	0.015342859533994327
IL13	GOTERM_BP_DIRECT	GO:0033314~mitotic DNA replication checkpoint signaling	6	0.4991680532	2.1318373498	ORC1, DONSON, TOPBP1, CDC6, CLSPN, TICRR	1119	11	19478	9.494516207652937	0.5989181224456546	0.015638475999801546	0.015342859533994327
IL13	GOTERM_BP_DIRECT	GO:0051321~meiotic cell cycle	15	1.2479201331	2.1893642414	XRCC2, PKMYT1, FBXO43, SMC1A, PSMC3IP, RAD51AP1, SGO2, RAD51, EXO1, DMC1, CDK2, RBBP8, RAD54L, NEK2, BUB3	1119	82	19478	3.1841365330543385	0.6086861240469776	0.015638475999801546	0.015342859533994327
IL13	GOTERM_BP_DIRECT	GO:0007099~centriole replication	8	0.6655574043	2.1897515985	PLK4, CEP152, CDK2, SASS6, CEP72, CCP110, WDR62, CEP85	1119	23	19478	6.054474103430858	0.6087510841498205	0.015638475999801546	0.015342859533994327
IL13	GOTERM_BP_DIRECT	GO:0071168~protein localization to chromatin	8	0.6655574043	2.1897515985	MMS22L, MSH2, PARP1, MCM8, PLK1, TONSL, ESCO2, EZH2	1119	23	19478	6.054474103430858	0.6087510841498205	0.015638475999801546	0.015342859533994327

IL13	GOTERM_BP_DIRECT	GO:0007095~mitotic G2 DNA damage checkpoint signaling	10	0.8319467554	2.3519681289	BLM, PLK1, CHEK1, CDK1, DONSON, BRCA1, TOPBP1, CLSPN, DTL, TICRR	1119	38	19478	4.580687644043083	0.6350288171676924	0.016521612184309854	0.016209301662272725
IL13	GOTERM_BP_DIRECT	GO:1900745~positive regulation of p38MAPK cascade	9	0.7487520798	2.7690585173	MFHAS1, BMP2, PRMT1, GADD45A, SPHK1, ZC3H12A, GDF6, XDH, MAP3K5	1119	31	19478	5.05353282	0.6947749437595815	0.019137767	0.018776003236967674
IL13	KEGG_PATHWAY	hsa04114:Oocyte meiosis	23	1.9134775374	2.9500047271	PLK1, ITPR2, ADCY1, PKMYT1, CDC25C, FBXO43, SMC1A, AURKA, SGO1, CDC20, CCNB2, CCNB1, ESPL1, STAG3, CCNE2, PTTG1, CCNE1, RPS6KA1, CDK2, CDK1, FBXO5, BUB1, MAD2L1	607	139	8534	2.3263603285411207	0.092774754	0.00572648	0.005275303
IL13	GOTERM_BP_DIRECT	GO:0006310~DNA recombination	16	1.3311148086	3.2158955125	INO80C, GEN1, PIF1, BLM, FEN1, LIG1, MCM8, XRCC3, BRCA1, BRCA2, PSMC3IP, RECQL4, RAD51, EME1, EXO1, RAD54L	1119	95	19478	2.9316400921875734	0.7479764772705132	0.021873194081592006	0.021459721801403223
IL13	KEGG_PATHWAY	hsa04657:IL-17 signaling pathway	18	1.4975041597	3.2797014024	CXCL6, HSP90AA1, CXCL8, CSF2, TNFAIP3, CXCL1, TRAF2, CXCL3, CXCL2, MMP9, CXCL5, NFKBIA, FOSL1, IL6, TRAF4, TRAF3, CCL2, IKBKE	607	95	8534	2.663868897945027	0.102594896	0.006012786	0.005539051
IL13	GOTERM_BP_DIRECT	GO:0070301~cellular response to hydrogen peroxide	13	1.0815307820	3.3309612813	PCNA, SPHK1, TNFAIP3, ERN1, NET1, IL6, IL18RAP, MYB, CDK1, PPIF, ECT2, EZH2, MAP3K5	1119	66	19478	3.428575297208005	0.7601051744854809	0.022301827	0.021880252
IL13	GOTERM_BP_DIRECT	GO:0006606~protein import into nucleus	17	1.4143094841	3.4884328157	IL33, NUP107, NUP188, NUP155, CSE1L, NUP153, IPO4, NFKBIA, POLA2, NUP85, NUP50, SIX2, NUP62, HEATR3, E2F3, NUP88, KPNA2	1119	106	19478	2.7916266208036147	0.7757635596496971	0.022775061346928888	0.022344540934069786
IL13	GOTERM_BP_DIRECT	GO:0071897~DNA biosynthetic process	9	0.7487520798	3.5079441047	POLD3, CENPF, LIG1, POLD1, SPHK1, POLE3, TK1, LIN9, TYMS	1119	32	19478	4.895609919571045	0.7776311433790783	0.022775061346928888	0.022344540934069786
IL13	GOTERM_BP_DIRECT	GO:0051298~centrosome duplication	7	0.5823627287	3.6021811514	STIL, CEP152, CDK2, SASS6, CCP110, BRCA2, NDC80	1119	18	19478	6.769238407308113	0.7864347120605055	0.023037830200042145	0.022602343
IL13	GOTERM_BP_DIRECT	GO:0098609~cell-cell adhesion	25	2.0798668885	3.7668893670	CYFIP2, CLSTN3, THY1, COL19A1, CX3CL1, ICAM1, ITGAV, CD58, STXBP6, CDON, KIRREL3, DSP, VCAM1, ITGA4, ANXA2, ITGA2, KRT18, VNN1, IGDCC4, COL8A2, ITGA6, ADAM8, CD24, TJP2, NECTIN1	1119	196	19478	2.2202312560412905	0.8009931791541363	0.023736943	0.02328824
IL13	BIOCARTA	h_g2Pathway:Cell Cycle: G2/M Checkpoint	10	0.8319467554	3.7878266182	CCNB1, GADD45A, RPS6KA1, PLK1, CHEK1, CDK1, BRCA1, CDC25C, CDC25A, ATR	162	25	1622	4.004938271604938	0.071917195	0.037120701	0.036552527
IL13	GOTERM_BP_DIRECT	GO:0006364~rRNA processing	20	1.6638935108	4.1403786213	NOP56, UTP15, NOP58, SUV39H1, NOP2, CHD7, PPAN, DDX21, NOLC1, NOC4L, PA2G4, IPO4, RRP9, BOP1, EXOSC5, DKC1, MRTO4, UTP20, EXOSC2, LYAR	1119	140	19478	2.4866590067662453	0.8304351151185396	0.025712351293332398	0.025226306846480605
IL13	GOTERM_BP_DIRECT	GO:0006915~apoptotic process	60	4.9916805324	4.2507484295	CYFIP2, ERRFI1, PDCD5, BUB1B, TNFAIP3, PCSK9, DPF1, ZC3H12A, CHEK1, PIM3, MAP3K9, PHLDA2, TP63, NKX3-1, HELLS, RBM14, TNFRSF12A, PARP1, CKAP2, TRAF1, MMP9, TRAIP, MELK, TRAF4, ESPL1, TRAF3, IRF1, BIRC5, CHI3L1, ELMO3, BIRC2, TLR2, BIRC3, MCM2, MTFP1, CDCA7, AURKA, DRAM1, NUAK2, CHST11, PMAIP1, BID, BUB1, RELT, EGLN3, DDIAS, GADD45A, TNFRSF9, EAF2, TNFRSF10B, TNFRSF10A, SULF1, GDF6, PALB2, TPX2, CDK1, CYCS, FAS, XAF1, TP73	1119	656	19478	1.5920682665271693	0.8382709600799452	0.026020652886692197	0.025528781
IL13	GOTERM_BP_DIRECT	GO:0099171~presynaptic modulation of chemical synaptic transmission	9	0.7487520798	4.3997318164	GRM2, CHRNB3, CHRNA5, ADORA2B, NOG, CHRNA6, NRXN3, ADCY1, HTR2A	1119	33	19478	4.747258103826469	0.8482771749962766	0.026553311033198048	0.026051369797798037

IL13	GOTERM_BP_DIRECT	GO:0030198~extracellular matrix organization	22	1.8302828618	4.6991664787	OLFML2B, COL27A1, COL22A1, ITGA2, PAPLN, MMP8, MMP9, COL19A1, NFKB2, ADAMTS4, ADAMTSL1, ADAMTS3, EGFLAM, ELF3, COL5A3, COL7A1, COL8A2, MMP19, APBB2, COL6A6, SLC39A8, ADAMTS6	1119	164	19478	2.3350334575731817	0.8665550298605663	0.027966567168769746	0.027437910939908522
IL13	GOTERM_BP_DIRECT	GO:0006955~immune response	51	4.2429284525	5.3458439610	CXCL6, CSF2, NRROS, CXCL8, CXCL1, CXCL3, ETS1, CXCL2, CXCL5, CX3CL1, CTSS, TNFSF13B, IL27RA, IL18RAP, PNP, ADGRE5, RGS1, TNFSF11, IKBKE, TNFRSF4, HLA-H, IL15, HLA-B, SERPINB9, PDCD1LG2, HLA-F, TNFRSF1B, TLR1, TLR10, TLR6, TLR2, PTGER4, CD274, SEMA7A, HEPACAM, PIK3R3, C3, IL1RL1, CCL2, CD58, IL32, CD74, LIF, APLN, IL6, TNFSF4, FAS, ULBP2, MAP3K14, IL18R1, NECTIN1	1119	537	19478	1.6531420212580068	0.898865771	0.031379372	0.030786202756755084
IL13	UP_KW_BIOLOGICAL_PROCESS	KW-0698~rRNA processing	18	1.4975041597	5.6241654310	UTP15, UTP4, SUV39H1, NOP2, RPP40, CHD7, DDX21, PA2G4, WDR12, WDR43, IPO4, RRP9, BOP1, EXOSC5, DKC1, UTP20, EXOSC2, LYAR	764	106	11523	2.561172577	0.073656504	0.007705107	0.006917723
IL13	KEGG_PATHWAY	hsa05200:Pathways in cancer	59	4.9084858569	6.1108803980	CXCL8, HHIP, LAMC2, BRCA2, ETS1, CKS1B, EDNRB, BDKRB1, ITGAV, PIM2, JAK3, NKX3-1, IFNAR2, IL15RA, HSP90AA1, IL15, ITGA2, TRAF2, TRAF1, MMP9, CCNA2, TRAF4, CCNE2, MSH2, CCNE1, TRAF3, COL4A4, CKS2, BIRC5, ITGA6, BIRC2, BIRC3, PTGER4, PIK3R3, LPAR3, ADCY1, RASGRP1, RASGRP3, DLL4, E2F1, E2F2, PMAIP1, E2F3, HES1, BID, WNT10B, EGLN3, LAMB3, GADD45A, WNT7B, VEGFC, NFKB2, NFKBIA, BMP2, IL6, RAD51, CDK2, CYCS, FAS	607	533	8534	1.5562836327894392	0.1826768206412367	0.010518166263046802	0.009689462
IL13	KEGG_PATHWAY	hsa00240:Pyrimidine metabolism	13	1.0815307820	6.3746462200	RRM1, DTYMK, RRM2, CTPS1, TYMS, TYMP, DHODH, NME1, NME1-NME2, NT5E, UCK2, TK1, DCTPP1	607	58	8534	3.1512242231437826	0.18976455714272422	0.010518166263046802	0.009689462
IL13	GOTERM_BP_DIRECT	GO:0007057~spindle assembly involved in female meiosis I	4	0.3327787021	7.2209159151	CCNB2, FBXO5, NDC80, AURKA	1119	4	19478	17.406613047363717	0.9547376691980027	0.040712664	0.039943066
IL13	GOTERM_BP_DIRECT	GO:0006272~leading strand elongation	4	0.3327787021	7.2209159151	POLA1, PCNA, POLE3, POLE	1119	4	19478	17.406613047363717	0.9547376691980027	0.040712664	0.039943066
IL13	GOTERM_BP_DIRECT	GO:1901970~positive regulation of mitotic sister chromatid separation	4	0.3327787021	7.2209159151	INCENP, CDCA8, BIRC5, AURKB	1119	4	19478	17.406613047363717	0.9547376691980027	0.040712664	0.039943066
IL13	GOTERM_BP_DIRECT	GO:0042098~T cell proliferation	10	0.8319467554	7.5029205688	GJA1, EBI3, HES1, CTPS1, TNFRSF4, RASGRP1, CORO1A, PSMB10, TNFSF13B, IDO1	1119	44	19478	3.95604842	0.9598931510458304	0.041217967483832184	0.040438818
IL13	GOTERM_BP_DIRECT	GO:0006275~regulation of DNA replication	10	0.8319467554	7.5029205688	INO80C, CCNA2, PCNA, DSCC1, POLE3, GMNN, ID3, ESCO2, BAZ1A, FBXO5	1119	44	19478	3.95604842	0.9598931510458304	0.041217967483832184	0.040438818
IL13	GOTERM_BP_DIRECT	GO:0030225~macrophage differentiation	9	0.7487520798	8.2278789649	IL33, IL1RL1, DIAPH3, CSF2, CSF1, PARP1, IL15, LIF, MMP9	1119	36	19478	4.351653262	0.9706098990007909	0.044628432	0.043784814
IL13	GOTERM_BP_DIRECT	GO:0038061~non-canonical NF-kappaB signal transduction	7	0.5823627287	9.0775469775	NFKBIA, TRAF2, MAP3K14, BIRC2, RELB, NFKB2, BIRC3	1119	21	19478	5.802204349	0.979585205	0.048621611	0.047702509
IL13	GOTERM_BP_DIRECT	GO:0002237~response to molecule of bacterial origin	5	0.4159733777	0.001077587	CXCL8, TAP2, TNFAIP3, CD24, CXCL2	1119	9	19478	9.670340581868732	0.990146727	0.056310511	0.055246065

IL13	GOTERM_BP_DIRECT	GO:0061518~microglial cell proliferation	5	0.4159733777	0.001077587	IL33, CSF1, IL34, NDP, CX3CL1	1119	9	19478	9.670340581868732	0.990146727	0.056310511	0.055246065
IL13	GOTERM_BP_DIRECT	GO:0001833~inner cell mass cell proliferation	6	0.4991680532	0.001142416	GINS1, CHEK1, GINS4, NCAPG2, BRCA2, PALB2	1119	15	19478	6.962645218945487	0.9925388999552056	0.058276834237428216	0.057175218
IL13	GOTERM_BP_DIRECT	GO:2000001~regulation of DNA damage checkpoint	6	0.4991680532	0.001142416	BARD1, RAD51, RFWD3, BRCA1, BRCA2, WDR76	1119	15	19478	6.962645218945487	0.9925388999552056	0.058276834237428216	0.057175218
IL13	GOTERM_BP_DIRECT	GO:0043065~positive regulation of apoptotic process	34	2.8286189683	0.001269601	TOP2A, HPGD, PDCD5, E2F1, ANKRD1, TNFRSF8, PMAIP1, APBB2, MAP3K9, SLIT2, PHLDA2, ECT2, BID, NTSR1, MAP3K5, BARD1, WNT10B, TNFRSF12A, GADD45A, DAB2IP, TNFRSF10B, TNFRSF10A, MMP9, NET1, FOSL1, BMP2, IL6, GAL, MELK, ID3, FAS, ITGA6, IDO1, TP73	1119	328	19478	1.8043440353974582	0.9956765237556222	0.062240031	0.061063499
IL13	GOTERM_BP_DIRECT	GO:0071356~cellular response to tumor necrosis factor	16	1.3311148086	0.001274048	VCAM1, CXCL8, DAB2IP, BRCA1, CLDN1, CRHBP, FABP4, ZC3H12A, ANKRD1, CCL2, CHI3L1, HAS2, HES1, CD58, NKX3-1, MAP3K5	1119	108	19478	2.5787574884983284	0.995758228	0.062240031	0.061063499
IL13	GOTERM_BP_DIRECT	GO:0007129~homologous chromosome pairing at meiosis	8	0.6655574043	0.001278208	PSMC3IP, NDC1, STAG3, CCNE2, FANCD2, CCNE1, DMC1, MND1	1119	30	19478	4.641763479296991	0.9958332803371076	0.062240031	0.061063499
IL13	GOTERM_BP_DIRECT	GO:0006298~mismatch repair	8	0.6655574043	0.001278208	POLD3, MSH5-SAPCD1, RNASEH2A, PCNA, MSH2, LIG1, EXO1, TP73	1119	30	19478	4.641763479296991	0.9958332803371076	0.062240031	0.061063499
IL13	KEGG_PATHWAY	hsa05417:Lipid and atherosclerosis	29	2.4126455906	0.001347906	CXCL8, PIK3R3, CXCL1, CXCL3, CXCL2, HSPD1, ICAM1, CCL2, OLR1, BID, IKBKE, MAP3K5, HSPA8, HSP90AA1, VCAM1, TNFRSF10B, TRAF2, TNFRSF10A, POU2F2, SOD2, MMP9, ERN1, NFKBIA, IL6, TRAF3, FAS, CYCS, TLR6, TLR2	607	216	8534	1.8875922875099151	0.3592456532089673	0.021181384748207556	0.019512548374106355
IL13	GOTERM_BP_DIRECT	GO:0071222~cellular response to lipopolysaccharide	23	1.9134775374	0.00156268	CXCL6, CD274, CXCL8, CSF2, SERPINE1, DAB2IP, TNFAIP3, CXCL1, PDCD1LG2, CXCL3, MMP8, TNFRSF1B, CXCL2, MMP9, CXCL5, IL6, EDNRB, TNIP3, TNFSF4, ZC3H12A, ANKRD1, CCL2, NFKBIB	1119	192	19478	2.085167187965445	0.998770714	0.074761581	0.073348351
IL13	GOTERM_BP_DIRECT	GO:0097421~liver regeneration	8	0.6655574043	0.001570255	IL6, PCNA, PNPT1, TYMS, CLDN1, EZH2, AURKA, PTPN3	1119	31	19478	4.492029173513218	0.9988100340441964	0.074761581	0.073348351
IL13	KEGG_PATHWAY	hsa05134:Legionellosis	12	0.9983361064	0.001663964	NFKBIA, C3, HSPA8, IL6, CXCL8, CYCS, CXCL1, CXCL3, CXCL2, HSPD1, NFKB2, TLR2	607	56	8534	3.0127088726759235	0.4227993431353603	0.024959452674614264	0.022992950342674956
IL13	GOTERM_BP_DIRECT	GO:0019885~antigen processing and presentation of endogenous peptide antigen via MHC class I	5	0.4159733777	0.001714609	ERAP2, ERAP1, TAP2, TAP1, TAPBP	1119	10	19478	8.703306523681858	0.9993595933124292	0.077939268	0.07646597
IL13	GOTERM_BP_DIRECT	GO:0007250~activation of NF-kappaB-inducing kinase activity	5	0.4159733777	0.001714609	TNFRSF10B, ZFP91, CHI3L1, TNFRSF10A, TLR6	1119	10	19478	8.703306523681858	0.9993595933124292	0.077939268	0.07646597
IL13	GOTERM_BP_DIRECT	GO:0045132~meiotic chromosome segregation	4	0.3327787021	0.001727942	SGO1, NUF2, SMC4, SMC2	1119	5	19478	13.925290437890975	0.999395213	0.077939268	0.07646597
IL13	GOTERM_BP_DIRECT	GO:0046602~regulation of mitotic centrosome separation	4	0.3327787021	0.001727942	CHEK1, NEK2, KIF11, CEP85	1119	5	19478	13.925290437890975	0.999395213	0.077939268	0.07646597

IL13	GOTERM_BP_DIRECT	GO:0046601~positive regulation of centriole replication	4	0.3327787021	0.001727942	PLK4, STIL, NUP62, SASS6	1119	5	19478	13.925290437890975	0.999395213	0.077939268	0.07646597
IL13	BIOCARTA	h_hivnefPathway:HIV-I Nef: negative effector of Fas and TNF	15	1.2479201331	0.001801019	PARP1, TRAF2, TRAF1, TNFRSF1B, LMNB2, LMNB1, NFKBIA, ARHGDIB, FAS, CYCS, BID, MAP3K14, BIRC2, BIRC3, MAP3K5	162	61	1622	2.462052216150577	0.29891116094580206	0.11766658774652956	0.11586556854632758
IL13	GOTERM_BP_DIRECT	GO:0050729~positive regulation of inflammatory response	16	1.3311148086	0.001845595	TNFSF18, PTGER4, IL33, TSLP, IL15, SERPINE1, HTR2A, ETS1, CX3CL1, NFKBIA, IL1RL1, GPRC5B, FABP4, NLRP10, TNFSF4, TLR2	1119	112	19478	2.4866590067662453	0.9996350251892014	0.08237891	0.080821689
IL13	GOTERM_BP_DIRECT	GO:0045740~positive regulation of DNA replication	8	0.6655574043	0.001911704	CDT1, PCNA, ATAD5, CDK2, CDK1, DNA2, BAZ1A, CDC25A	1119	32	19478	4.351653261840929	0.9997252065915524	0.08445001	0.082853639
IL13	GOTERM_BP_DIRECT	GO:0016477~cell migration	29	2.4126455906	0.001936374	ERRFI1, FLT1, WWC1, TNFAIP3, CORO1A, RND1, ADGRE2, ADGRG1, STRIP2, PODXL, SIX2, BDKRB1, HES1, ITGAV, SPDL1, FAM83D, ZG16B, L1CAM, MMP9, KNSTRN, DEPDC1B, CDK1, SDC1, DCHS1, CEP85, CD24, DOCK2, EPHA3, EPHA2	1119	271	19478	1.8627002892012836	0.9997528231056756	0.084666965	0.083066492
IL13	GOTERM_BP_DIRECT	GO:0043066~negative regulation of apoptotic process	47	3.9101497504	0.001979005	HHIP, GATA6, KIF14, CX3CL1, HSPD1, AURKA, SOCS2, EDNRB, NUAK2, CHST11, FIGNL1, NUP62, RPS6KA1, APBB2, PIM3, PIM2, NTSR1, BARD1, TNFSF18, CD74, STIL, SERPINB2, TSLP, PLK2, SPHK1, PLK1, PLAUR, SERPINB9, PA2G4, SOD2, MMP9, DKK1, ATAD3A, PALB2, SH3RF2, IL6, ACTC1, KRT18, GDNF, CDK1, PPIF, BIRC5, FAS, NAA15, BIRC2, BIRC3, MAD2L1	1119	515	19478	1.588564685875912	0.999794166	0.085656943	0.084037757
IL13	GOTERM_BP_DIRECT	GO:0010212~response to ionizing radiation	9	0.7487520798	0.002014595	RRM1, VCAM1, RFWD3, RAD54L, AEN, BRCA1, TOPBP1, TICRR, RAD54B	1119	41	19478	3.820963839665206	0.9998233333786442	0.086325389	0.084693567
IL13	UP_KW_BIOLOGICAL_PROCESS	KW-0226~DNA condensation	6	0.4991680532	0.002157375	NCAPG2, NCAPG, NCAPD3, SMC4, NCAPH, SMC2	764	15	11523	6.032984293193717	0.25451477515642407	0.026869126646552298	0.024123376478291478
IL13	GOTERM_BP_DIRECT	GO:0060707~trophoblast giant cell differentiation	5	0.4159733777	0.002572567	PLK4, LIF, PRDM1, E2F7, E2F8	1119	11	19478	7.91209684	0.9999839154732347	0.10914306112102007	0.10707991340788059
IL13	KEGG_PATHWAY	hsa05161:Hepatitis B	23	1.9134775374	0.002573742	EGR2, PCNA, CXCL8, PIK3R3, MMP9, IFIH1, NFKBIA, CCNA2, IL6, CCNE2, CCNE1, TRAF3, CDK2, E2F1, FAS, BIRC5, E2F2, CYCS, E2F3, BID, JAK3, IKBKE, TLR2	607	163	8534	1.983828746424637	0.5727687565537111	0.036805653	0.033905814
IL13	KEGG_PATHWAY	hsa05323:Rheumatoid arthritis	16	1.3311148086	0.002676775	CXCL6, FLT1, CXCL8, CSF2, CSF1, IL15, CXCL1, CXCL3, CXCL2, CXCL5, TNFSF13B, ICAM1, IL6, CCL2, TNFSF11, TLR2	607	95	8534	2.367883464840024	0.5870876892441287	0.036805653	0.033905814
IL13	GOTERM_BP_DIRECT	GO:0002830~positive regulation of type 2 immune response	4	0.3327787021	0.003308369	IL33, CD74, TNFSF4, IDO1	1119	6	19478	11.604408698242478	0.999999319	0.13501296998620754	0.13246079949171913
IL13	GOTERM_BP_DIRECT	GO:0006335~DNA replication-dependent chromatin assembly	4	0.3327787021	0.003308369	CHAF1B, CHAF1A, NASP, ASF1B	1119	6	19478	11.604408698242478	0.999999319	0.13501296998620754	0.13246079949171913
IL13	GOTERM_BP_DIRECT	GO:0051383~kinetochore organization	4	0.3327787021	0.003308369	NUF2, SMC4, NDC80, SMC2	1119	6	19478	11.604408698242478	0.999999319	0.13501296998620754	0.13246079949171913
IL13	GOTERM_BP_DIRECT	GO:1903490~positive regulation of mitotic cytokinesis	4	0.3327787021	0.003308369	INCENP, CDCA8, BIRC5, AURKB	1119	6	19478	11.604408698242478	0.999999319	0.13501296998620754	0.13246079949171913

IL13	GOTERM_BP_DIRECT	GO:0002224~toll-like receptor signaling pathway	7	0.5823627287	0.003686448	TLR1, FOSL1, TRAF3, TLR10, TLR6, CTSS, TLR2	1119	27	19478	4.512825604872075	0.9999998660255135	0.14824755835787604	0.14544521244726039
IL13	GOTERM_BP_DIRECT	GO:0010628~positive regulation of gene expression	44	3.6605657237	0.003701864	CNTF, DNMT1, CSF2, CXCL8, CSF1, PDCD5, PIK3R3, BRCA1, PRDM1, ETS1, IQGAP3, ROBO1, DLL4, GJA1, CYP26B1, C1QTNF1, ZC3H12A, NAMPT, E2F1, CCL2, TNFSF11, HES1, IKBKE, NTSR1, NKX3-1, IL32, CD74, IL33, IL34, NTRK3, NOG, LIF, ANK2, DKK1, BMP2, IL6, ACTC1, RNF207, CDK1, ID3, TLR6, ADTRP, EZR, TLR2	1119	489	19478	1.5662392108057335	0.9999998746206376	0.14824755835787604	0.14544521244726039
IL13	KEGG_PATHWAY	hsa03013:Nucleocytoplasmic transport	17	1.4143094841	0.003793024	NDC1, NUP205, NUP107, SEH1L, NXT1, NUP188, NUP155, CSE1L, NUP153, IPO4, NUP85, NUP50, XPO5, NUP62, NUP35, NUP88, KPNA2	607	108	8534	2.213039233632314	0.7146610874604842	0.05006791	0.046123166
IL13	GOTERM_BP_DIRECT	GO:0042273~ribosomal large subunit biogenesis	8	0.6655574043	0.003882231	NOP16, PAK1IP1, MRTO4, NOP2, RRS1, HEATR3, WDR12, GTPBP4	1119	36	19478	3.868136232747493	0.9999999422841908	0.1540311086738294	0.1511194354410219
IL13	BIOCARTA	h_atrbrcaPathway:Role of BRCA1, BRCA2 and ATR in Cancer Susceptibility	8	0.6655574043	0.003927726	RAD51, FANCD2, CHEK1, FANCA, BRCA1, BRCA2, FANCG, ATR	162	22	1622	3.6408529741863074	0.5394277718546637	0.18667399676539606	0.1838167417128645
IL13	KEGG_PATHWAY	hsa01523:Antifolate resistance	8	0.6655574043	0.004242933	DHFR, ABCC3, IL6, SHMT1, GGH, TYMS, SLC19A1, ABCG2	607	30	8534	3.7491488193300384	0.7541777637507807	0.05207277	0.047970067
IL13	KEGG_PATHWAY	hsa05162:Measles	20	1.6638935108	0.004260499	IFNAR2, HSPA8, PIK3R3, TNFAIP3, IFIH1, NFKBIA, IL6, CCNE2, CCNE1, TRAF3, OAS3, CDK2, FAS, CYCS, BID, JAK3, IKBKE, NFKBIB, TLR2, TP73	607	139	8534	2.022922024818366	0.7556047234570227	0.05207277	0.047970067
IL13	UP_KW_BIOLOGICAL_PROCESS	KW-0090~Biological rhythms	20	1.6638935108	0.00440946	TOP2A, KLF10, SUV39H2, SUV39H1, BHLHE41, ADCY1, NPAS2, RELB, NFKB2, GPR176, MYBBP1A, NOCT, BHLHE40, NAMPT, TIMELESS, CDK1, ID3, DTL, EZH2, CDK5R1	764	149	11523	2.024491373554939	0.45174378629132705	0.050341331	0.045196961
IL13	KEGG_PATHWAY	hsa04061:Viral protein interaction with cytokine and cytokine receptor	16	1.3311148086	0.004424152	CXCL6, CXCL8, CSF1, IL34, TNFRSF10B, CXCL1, TNFRSF10A, CXCL3, TNFRSF1B, CXCL2, CXCL5, CX3CL1, IL6, IL18RAP, CCL2, IL18R1	607	100	8534	2.249489291598023	0.7685078453104872	0.052141792	0.048033650711501175
IL13	GOTERM_BP_DIRECT	GO:0006405~RNA export from nucleus	6	0.4991680532	0.00464145	DDX39A, NUP188, NUP155, NUP62, XPO5, NUP153	1119	20	19478	5.221983914209116	0.9999999978002267	0.1791766868883938	0.17578968300555603
IL13	GOTERM_BP_DIRECT	GO:0036342~post-anal tail morphogenesis	6	0.4991680532	0.00464145	HES7, CHST11, DCHS1, PALB2, TP63, EPHA2	1119	20	19478	5.221983914209116	0.9999999978002267	0.1791766868883938	0.17578968300555603
IL13	GOTERM_BP_DIRECT	GO:0007062~sister chromatid cohesion	6	0.4991680532	0.00464145	STAG3, DDX11, PLK1, ESCO2, KIF22, SMC1A	1119	20	19478	5.221983914209116	0.9999999978002267	0.1791766868883938	0.17578968300555603
IL13	BIOCARTA	h_bard1Pathway:BRCA1-dependent Ub-ligase activity	5	0.4159733777	0.004762092	BARD1, FANCD2, FANCA, BRCA1, FANCG	162	8	1622	6.257716049382716	0.6095178135462838	0.18667399676539606	0.1838167417128645
IL13	GOTERM_BP_DIRECT	GO:0042274~ribosomal small subunit biogenesis	12	0.9983361064	0.004812803	NOP56, LTV1, UTP15, RCL1, NOP58, UTP4, PNO1, DHX37, UTP20, WDR43, IPO4, RRP9	1119	78	19478	2.6779404688251875	0.9999999989480809	0.18413270423896788	0.18065201601414727
IL13	KEGG_PATHWAY	hsa04914:Progesterone-mediated oocyte maturation	17	1.4143094841	0.004989181	HSP90AA1, PLK1, PIK3R3, ADCY1, KIF22, PKMYT1, CDC25C, CDC25A, AURKA, CCNA2, CCNB2, CCNB1, RPS6KA1, CDK2, CDK1, BUB1, MAD2L1	607	111	8534	2.1532273624530625	0.8080558376926954	0.056773444	0.052300385206028775

IL13	GOTERM_BP_DIRECT	GO:0051983~regulation of chromosome segregation	5	0.4159733777	0.005082798	CDCA2, KIF2C, MKI67, BUB1, AURKB	1119	13	19478	6.694851172062968	0.9999999996711204	0.1927415032422129	0.18909808159399366
IL13	GOTERM_BP_DIRECT	GO:0007249~canonical NF-kappaB signal transduction	8	0.6655574043	0.005312747	NFKBIA, IRAK2, ADAM8, MAP3K14, BIRC2, RELB, NFKB2, BIRC3	1119	38	19478	3.664550115234467	0.9999999998778548	0.19960406033504768	0.1958309147371156
IL13	UP_KW_BIOLOGICAL_PROCESS	KW-0037~Angiogenesis	19	1.5806988352	0.005431653	ROBO4, FLT1, TNFRSF12A, C1GALT1, DAB2IP, TNFAIP2, NRXN3, VEGFC, TYMP, APLN, DLL4, ARHGAP22, ESM1, ZC3H12A, COL8A2, MMP19, CSPG4, NAA15, EPHA2	764	141	11523	2.032388325721288	0.5232286596572397	0.057241264	0.051391792
IL13	GOTERM_BP_DIRECT	GO:0032755~positive regulation of interleukin-6 production	14	1.1647254575	0.0055404	CD74, IL33, TSLP, POU2F2, HSPD1, IFIH1, TLR1, IL1RL2, IL6, NLRP10, ADORA2B, TNFSF4, TLR6, TLR2	1119	102	19478	2.3891429672852165	0.9999999999541948	0.19960406033504768	0.1958309147371156
IL13	GOTERM_BP_DIRECT	GO:0006312~mitotic recombination	4	0.3327787021	0.005543263	RAD51, MSH2, DMC1, RAD54B	1119	7	19478	9.946636027064981	0.9999999999547565	0.19960406033504768	0.1958309147371156
IL13	GOTERM_BP_DIRECT	GO:0002043~blood vessel endothelial cell proliferation involved in sprouting angiogenesis	4	0.3327787021	0.005543263	SEMA5A, BMPER, NRARP, EPHA2	1119	7	19478	9.946636027064981	0.9999999999547565	0.19960406033504768	0.1958309147371156
IL13	GOTERM_BP_DIRECT	GO:0010032~meiotic chromosome condensation	4	0.3327787021	0.005543263	NCAPD3, SMC4, NCAPH, SMC2	1119	7	19478	9.946636027064981	0.9999999999547565	0.19960406033504768	0.1958309147371156
IL13	GOTERM_BP_DIRECT	GO:0044772~mitotic cell cycle phase transition	4	0.3327787021	0.005543263	CCNB2, CCNB1, CKS2, CKS1B	1119	7	19478	9.946636027064981	0.9999999999547565	0.19960406033504768	0.1958309147371156
IL13	GOTERM_BP_DIRECT	GO:0007155~cell adhesion	48	3.9933444259	0.005675734	SEMA5A, ACHE, CNTNAP1, HEPACAM, CLSTN3, TROAP, NRXN3, LAMC2, THY1, CLDN1, COL19A1, CX3CL1, ROBO1, ICAM1, ADGRE2, ADGRG1, ADGRE5, FLRT2, PODXL, CCL2, ABL2, OLR1, HES1, ITGAV, OPCML, CDON, IL32, VCAM1, MYBPC2, TNFRSF12A, LAMB3, ITGA4, ITGA2, GP1BA, RGMB, L1CAM, MTSS1, CLDN4, COL7A1, COL6A1, ANOS1, ITGA6, COL6A6, CLDN16, CD24, EPHA3, EPHA2, NECTIN1	1119	559	19478	1.4946644477163837	0.9999999999744351	0.20267098992579283	0.19883986969615705
IL13	GOTERM_BP_DIRECT	GO:0060236~regulation of mitotic spindle organization	6	0.4991680532	0.005808229	TPX2, STIL, BORA, NUP62, SASS6, TACC3	1119	21	19478	4.9733180135324915	0.9999999999855572	0.20526095627005664	0.20138087751676034
IL13	GOTERM_BP_DIRECT	GO:0071260~cellular response to mechanical stimulus	12	0.9983361064	0.005844069	PTGER4, IL33, GADD45A, ITGA2, IRF1, CHEK1, ANKRD1, FAS, TNFRSF10B, TNFRSF8, TNFRSF10A, MAP3K14	1119	80	19478	2.610991957104558	0.9999999999876245	0.20526095627005664	0.20138087751676034
IL13	BIOCARTA	h_tnfr2Pathway:TNFR2 Signaling Pathway	7	0.5823627287	0.005875518	NFKBIA, TRAF3, TNFAIP3, TRAF2, TRAF1, TNFRSF1B, MAP3K14	162	18	1622	3.893689986282579	0.6867929595685183	0.19193359181576236	0.18899583275735785
IL13	GOTERM_BP_DIRECT	GO:0050767~regulation of neurogenesis	8	0.6655574043	0.006160803	DLL4, HES7, ANXA2, CHD7, BHLHE40, BHLHE41, HES1, CX3CL1	1119	39	19478	3.570587291766916	0.9999999999968407	0.21462635645527126	0.21056924213254619
IL13	GOTERM_BP_DIRECT	GO:0009410~response to xenobiotic stimulus	26	2.1630615640	0.006318599	ERRFI1, GATA6, HTR2A, ADCY1, TYMS, PFAS, LRP8, NPAS2, RAD54B, PNP, RAD54L, SLC19A1, TIGAR, HSP90AA1, ITGA2, VEGFC, CTPS1, SOD2, FOSL1, CENPF, RAD51, GAL, COL6A1, CDK1, ATR, TP73	1119	255	19478	1.7747919185547318	0.9999999999984002	0.21717031540261206	0.2130651122409758

IL13	GOTERM_BP_DIRECT	GO:0007098~centrosome cycle	9	0.7487520798	0.006344795	HAUS8, GADD45A, XRCC2, PLK1, NUP62, CDK1, PCLAF, HAUS6, BRCA1	1119	49	19478	3.1971330086994585	1	0.21717031540261206	0.2130651122409758
IL13	GOTERM_BP_DIRECT	GO:0000731~DNA synthesis involved in DNA repair	7	0.5823627287	0.006385872	POLD3, POLQ, POLA1, RRM1, RFC3, POLD1, POLE	1119	30	19478	4.061543044384868	1	0.21717031540261206	0.2130651122409758
IL13	KEGG_PATHWAY	hsa01232:Nucleotide metabolism	14	1.1647254575	0.006603511	RRM1, DTYMK, RRM2, CTPS1, TYMS, TYMP, NME1, NME1-NME2, NT5E, UCK2, PNP, TK1, DCTPP1, XDH	607	85	8534	2.315650741350906	0.8876780053080805	0.072638623	0.06691558
IL13	GOTERM_BP_DIRECT	GO:0032465~regulation of cytokinesis	8	0.6655574043	0.007105769	PRC1, PLK1, CCP110, KIF20A, BRCA2, MYO19, AURKB, AURKA	1119	40	19478	3.481322609472744	0.9999999999999464	0.23974977626824606	0.23521775015909133
IL13	UP_KW_BIOLOGICAL_PROCESS	KW-0346~Stress response	16	1.3311148086	0.007463844	HSP90AA2P, HSPA8, HSP90AA1, HSPA4L, DAB2IP, HSPE1, HSPA12A, HSPD1, MANF, HSP90B2P, HSPH1, RPS6KA1, ZC3H12A, HYOU1, MAP3K9, MAP3K5	764	113	11523	2.135569661307511	0.6390045743014279	0.073039048	0.065575204
IL13	GOTERM_BP_DIRECT	GO:0007131~reciprocal meiotic recombination	7	0.5823627287	0.00754162	PSMC3IP, RAD51, DMC1, RAD54L, MND1, TRIP13, RAD54B	1119	31	19478	3.9305255268240655	0.9999999999999918	0.2524674994481424	0.2476950683033817
IL13	GOTERM_BP_DIRECT	GO:0000723~telomere maintenance	10	0.8319467554	0.007684257	INO80C, RECQL4, DCLRE1B, PIF1, BLM, PARP1, CCNE2, CCNE1, DNA2, ATR	1119	61	19478	2.853543122518642	0.9999999999999956	0.255248367	0.2504233683854707
IL13	GOTERM_BP_DIRECT	GO:0008283~cell population proliferation	18	1.4975041597	0.007797499	USP13, CSF2, ATAD5, MCM7, SPHK1, PAK1IP1, NRG1, MCM10, MKI67, CDC25C, CDC25A, BOP1, IL18RAP, MELK, SIX2, OTUD6B, POLR3G, FAM83D	1119	155	19478	2.0214131280809475	0.9999999999999973	0.25701757098668526	0.2521591291547316
IL13	GOTERM_BP_DIRECT	GO:0032467~positive regulation of cytokinesis	8	0.6655574043	0.008153952	RACGAP1, KIF14, KIF23, CDC6, KIF20B, ECT2, AURKB, CIT	1119	41	19478	3.3964123019246277	0.9999999999999994	0.264694588	0.25969102680313133
IL13	GOTERM_BP_DIRECT	GO:0000077~DNA damage checkpoint signaling	8	0.6655574043	0.008153952	BRIP1, CHEK1, DOT1L, DONSON, E2F1, TOPBP1, CLSPN, ATR	1119	41	19478	3.3964123019246277	0.9999999999999994	0.264694588	0.25969102680313133
IL13	GOTERM_BP_DIRECT	GO:1902425~positive regulation of attachment of mitotic spindle microtubules to kinetochore	4	0.3327787021	0.008492942	INCENP, CDCA8, BIRC5, AURKB	1119	8	19478	8.703306523681858	0.9999999999999999	0.26759013561562184	0.2625318390030511
IL13	GOTERM_BP_DIRECT	GO:0110025~DNA strand resection involved in replication fork processing	4	0.3327787021	0.008492942	BARD1, EXO1, RBBP8, BRCA1	1119	8	19478	8.703306523681858	0.9999999999999999	0.26759013561562184	0.2625318390030511
IL13	GOTERM_BP_DIRECT	GO:0071459~protein localization to chromosome, centromeric region	4	0.3327787021	0.008492942	MIS18A, BUB1B, HASPIN, CENPA	1119	8	19478	8.703306523681858	0.9999999999999999	0.26759013561562184	0.2625318390030511
IL13	GOTERM_BP_DIRECT	GO:0006999~nuclear pore organization	4	0.3327787021	0.008492942	NDC1, NUP205, SEH1L, NUP35	1119	8	19478	8.703306523681858	0.9999999999999999	0.26759013561562184	0.2625318390030511
IL13	GOTERM_BP_DIRECT	GO:0006287~base-excision repair, gap-filling	5	0.4159733777	0.008852182	FEN1, PCNA, LIG1, POLD1, POLE	1119	15	19478	5.802204349121238	1	0.2748666812147204	0.2696708349653873
IL13	GOTERM_BP_DIRECT	GO:0007064~mitotic sister chromatid cohesion	5	0.4159733777	0.008852182	CDC20, CDCA5, ESCO2, SMC1A, HASPIN	1119	15	19478	5.802204349121238	1	0.2748666812147204	0.2696708349653873

IL13	GOTERM_BP_DIRECT	GO:0045892~negative regulation of DNA-templated transcription	48	3.9933444259	0.009000842	SUV39H2, CSF2, SUV39H1, GMNN, GATA6, BHLHE41, BRCA1, ENO1, FOXM1, HDAC9, RELB, TRIM6, RASD1, SCML1, BCL7A, MYB, E2F1, RBBP8, ANKRD1, HES1, TNFRSF4, PPARGC1B, TP63, NKX3-1, KLF10, HSPA8, PARP1, SFMBT1, DAB2IP, HMGA1, NRG1, PA2G4, BMP2, CENPF, FABP4, ELF3, MYBBP1A, IRF1, TNFSF4, ZNF219, BHLHE40, TIMELESS, DEPDC1, SRSF2, ID3, BIRC5, CDK5R1, EZH2	1119	573	19478	1.458145595590678	1	0.2774719910187975	0.27222689620607343
IL13	GOTERM_BP_DIRECT	GO:0042102~positive regulation of T cell proliferation	10	0.8319467554	0.009473615	CD274, IL6, VCAM1, PNP, IL15, TNFSF4, HES1, PDCD1LG2, CORO1A, TNFSF13B	1119	63	19478	2.7629544519624947	1	0.2899602995133931	0.28447913632539196
IL13	GOTERM_BP_DIRECT	GO:1901796~regulation of signal transduction by p53 class mediator	8	0.6655574043	0.0105850703	BOP1, PAK1IP1, NOP2, CHEK1, RRS1, URB2, AURKB, AURKA	1119	43	19478	3.238439636718831	1	0.32168103939305576	0.3156002542843422
IL13	GOTERM_BP_DIRECT	GO:0007019~microtubule depolymerization	5	0.4159733777	0.0112750104	KIF18A, KIF18B, KIF24, STMN3, KIF2C	1119	16	19478	5.439566577301162	1	0.3331959973425105	0.32689754325038833
IL13	GOTERM_BP_DIRECT	GO:0008156~negative regulation of DNA replication	5	0.4159733777	0.0112750104	SLFN11, GMNN, CDC6, GTPBP4, ATR	1119	16	19478	5.439566577301162	1	0.3331959973425105	0.32689754325038833
IL13	GOTERM_BP_DIRECT	GO:0000463~maturation of LSU-rRNA from tricistronic rRNA transcript (SSU-rRNA, 5.8S rRNA, LSU-rRNA)	5	0.4159733777	0.0112750104	BOP1, PAK1IP1, PPAN, WDR12, GTPBP4	1119	16	19478	5.439566577301162	1	0.3331959973425105	0.32689754325038833
IL13	GOTERM_BP_DIRECT	GO:0006164~purine nucleotide biosynthetic process	5	0.4159733777	0.0112750104	PRPS2, SLC4A7, MTHFD1, PPAT, PFAS	1119	16	19478	5.439566577301162	1	0.3331959973425105	0.32689754325038833
IL13	GOTERM_BP_DIRECT	GO:0035987~endodermal cell differentiation	7	0.5823627287	0.0119057335	LAMB3, ITGA4, COL7A1, COL6A1, ITGAV, MMP8, MMP9	1119	34	19478	3.5837144509278245	1	0.3485310446313021	0.34194270983197056
IL13	GOTERM_BP_DIRECT	GO:0035563~positive regulation of chromatin binding	4	0.3327787021	0.0122006199	CDT1, KDM4D, DDX11, GMNN	1119	9	19478	7.736272465494986	1	0.3485310446313021	0.34194270983197056
IL13	GOTERM_BP_DIRECT	GO:0150105~protein localization to cell-cell junction	4	0.3327787021	0.0122006199	DSP, HEPACAM, CGNL1, TJP2	1119	9	19478	7.736272465494986	1	0.3485310446313021	0.34194270983197056
IL13	GOTERM_BP_DIRECT	GO:0000447~endonucleolytic cleavage in ITS1 to separate SSU-rRNA from 5.8S rRNA and LSU-rRNA from tricistronic rRNA transcript (SSU-rRNA, 5.8S rRNA, LSU-rRNA)	4	0.3327787021	0.0122006199	RCL1, RPP40, RRS1, UTP20	1119	9	19478	7.736272465494986	1	0.3485310446313021	0.34194270983197056
IL13	GOTERM_BP_DIRECT	GO:0086014~atrial cardiac muscle cell action potential	4	0.3327787021	0.0122006199	GJC1, GJA1, NUP155, ANK2	1119	9	19478	7.736272465494986	1	0.3485310446313021	0.34194270983197056

IL13	KEGG_PATHWAY	hsa04814:Motor proteins	24	1.9966722129	0.0122920549	DNAH11, KIF14, KIF24, KIF23, KIF11, KIF22, TUBB4B, TUBG1, MYO19, KIF15, CENPE, TUBA1C, TUBA1B, KIF18A, TUBB2B, ACTC1, KIF18B, KIFC1, KIF4A, KIF2C, KIF20A, KIF1A, KIF21B, KIF20B	607	197	8534	1.7128091052776826	0.9831181290613465	0.13014767529671728	0.11989361603091532
IL13	GOTERM_BP_DIRECT	GO:0046697~decidualization	6	0.4991680532	0.0125417254	PARP1, PARP2, LIF, STC1, NDP, JUNB	1119	25	19478	4.177587131367293	1	0.35590260648095556	0.34917492593837507
IL13	BIOCARTA	h_deathPathway:Induction of apoptosis through DR3 and DR4/5 Death Receptors	9	0.7487520798	0.0126167781	NFKBIA, TNFRSF10B, CYCS, TRAF2, TNFRSF10A, BID, MAP3K14, BIRC2, BIRC3	162	33	1622	2.730639731	0.9180231152320318	0.3270714155441558	0.32206522040827584
IL13	KEGG_PATHWAY	hsa03420:Nucleotide excision repair	11	0.9151414309	0.0126203806	RFC5, POLD3, RFC3, RFC4, PCNA, LIG1, RFC2, POLD1, POLE2, POLE3, POLE	607	63	8534	2.454799822180382	0.9848723191696394	0.13014767529671728	0.11989361603091532
IL13	UP_KW_BIOLOGICAL_PROCESS	KW-0053~Apoptosis	54	4.4925124792	0.0132992182	CYFIP2, PDCD5, BUB1B, TNFAIP3, PCSK9, ZC3H12A, PIM3, PIM2, MAP3K9, TP63, MAP3K5, TNFRSF12A, PARP1, CKAP2, AEN, TRAF2, TRAF1, TNFRSF1B, ERN1, MELK, TRAF4, TRAF3, PPIF, BIRC5, CHI3L1, ELMO3, BIRC2, EPHA2, BIRC3, MTFP1, CDCA7, DRAM1, NUAK2, E2F1, PMAIP1, BID, BUB1, RELT, EGLN3, TIGAR, DDIAS, DAB2IP, EAF2, G0S2, TNFRSF10B, TNFRSF10A, SULF1, GDF6, TPX2, CDK1, CYCS, FAS, XAF1, TP73	764	589	11523	1.3827722913092564	0.8381084312097342	0.12146619329472413	0.10905358960037276
IL13	BIOCARTA	h_atmPathway:ATM Signaling Pathway	7	0.5823627287	0.0133498536	NFKBIA, RAD51, GADD45A, CHEK1, RBBP8, BRCA1, TP73	162	21	1622	3.337448559670782	0.9291813945746851	0.3270714155441558	0.32206522040827584
IL13	GOTERM_BP_DIRECT	GO:0043406~positive regulation of MAP kinase activity	8	0.6655574043	0.0135036879	FLT1, IL34, NTRK3, TNFSF11, CD24, RASGRP1, EZH2, ROBO1	1119	45	19478	3.0945089861979946	1	0.37819152205866025	0.37104251078987344
IL13	GOTERM_BP_DIRECT	GO:0032722~positive regulation of chemokine production	8	0.6655574043	0.0135036879	IL33, IL1RL1, CD74, IL6, TSLP, ADORA2B, TNFSF4, TLR2	1119	45	19478	3.0945089861979946	1	0.37819152205866025	0.37104251078987344
IL13	GOTERM_BP_DIRECT	GO:0007165~signal transduction	95	7.9034941763	0.0136347614	ARHGAP11A, CXCL6, ARHGAP11B, CNTNAP1, CNTF, OLFML2B, CXCL8, HHIP, CXCL1, CXCL3, CXCL2, CXCL5, RND1, NRGN, TNFSF13B, CRHBP, ARHGAP42, GJA1, DUSP10, PLAU, RGS1, NAMPT, RPS6KA1, TNFRSF8, SPX, MAP3K9, GPR39, CHRNB3, IL15, NFAM1, PLAUR, TRAF2, VRK1, ANK2, RGMB, MOB3B, TRAIP, ARHGAP45, TLR1, GPRC5A, TRAF4, TRAF3, TLR6, HBEGF, TLR2, CD274, CHRNA5, PKN3, CHRNA6, NRXN3, ARHGAP18, STC1, ITPR2, AKAP5, RASGRP1, PLCXD2, LRP8, TYMP, ARHGAP22, DLL4, C3, IL1RL1, IL1RL2, RASD1, RACGAP1, IRAK2, NCR3LG1, ABL2, CCL2, FAM83D, SH2B3, PAG1, IL32, TNFSF18, GABRQ, HSPA8, VEGFC, TNFRSF10B, TNFRSF10A, CDC7, APLN, NFKB2, NET1, PTPRE, GDNF, TNFSF4, CDK2, CSPG5, FAS, CCDC3, RHEBL1, ULBP2, IL18R1, NFKBIB, CD320	1119	1308	19478	1.2642417733177012	1	0.3784304558081639	0.37127692793874467
IL13	GOTERM_BP_DIRECT	GO:0098656~monoatomic anion transmembrane transport	7	0.5823627287	0.0136888496	PANX1, SLC4A7, ABCC3, LRRC8C, LRRC8D, LRRC8B, SLC19A1	1119	35	19478	3.4813226094727434	1	0.3784304558081639	0.37127692793874467
IL13	GOTERM_BP_DIRECT	GO:0000712~resolution of meiotic recombination intermediates	5	0.4159733777	0.0140862668	TOP2A, ANKLE1, FANCM, CENPX, EME1	1119	17	19478	5.119592072754034	1	0.3869208552713513	0.3796068321028613

IL13	GOTERM_BP_DIRECT	GO:0043123~positive regulation of canonical NF-kappaB signal transduction	23	1.9134775374	0.0144970788	CD74, PARP1, PLK2, DAB2IP, DDX21, TNFRSF10B, TRAF2, CX3CL1, GJA1, GPRC5B, TRIM6, EDNRB, IRAK2, TIFA, NUP62, NAMPT, TNFSF11, PIM2, TLR6, ECT2, IKBKE, BIRC2, BIRC3	1119	232	19478	1.725655603833472	1	0.39566868158745877	0.38818929694134807
IL13	GOTERM_BP_DIRECT	GO:0032496~response to lipopolysaccharide	16	1.3311148086	0.0146134124	PTGER4, PTGIR, VCAM1, HPGD, DIO2, SOD2, CLDN1, NFKB2, TRIM6, DUSP10, NOCT, COL6A1, E2F1, BDKRB1, IDO1, TLR2	1119	140	19478	1.9893272054129962	1	0.39631944482744474	0.3888277587058524
IL13	GOTERM_BP_DIRECT	GO:0045943~positive regulation of transcription by RNA polymerase I	6	0.4991680532	0.0148108737	UTP15, MYBBP1A, BNC1, DDX21, LYAR, WDR43	1119	26	19478	4.016910703237781	1	0.39914839033228966	0.39160322822799204
IL13	GOTERM_BP_DIRECT	GO:0030335~positive regulation of cell migration	27	2.2462562396	0.0150941186	SEMA5A, CD274, SEMA7A, FLT1, CSF2, CSF1, PIK3R3, LAMC2, CLDN1, CX3CL1, PLAU, PODXL, CCL2, HAS2, ITGAV, HSPA8, ARHGEF39, SPHK1, NTRK3, SOD2, SH3RF2, BMP2, CLDN4, ITGA6, EZH2, HBEGF, EPHA2	1119	288	19478	1.6318699731903485	1	0.40423936461516746	0.3965979670576812
IL13	UP_KW_BIOLOGICAL_PROCESS	KW-0811~Translocation	13	1.0815307820	0.0152402288	NDC1, NUP205, NUP107, SEH1L, NUP188, NUP155, NUP153, TIMM10, NUP85, NUP50, NUP62, NUP35, NUP88	764	90	11523	2.1785776614310643	0.8761429803684887	0.1304944598523282	0.11715925957544794
IL13	KEGG_PATHWAY	hsa01524:Platinum drug resistance	12	0.9983361064	0.0161567068	TOP2A, MSH2, FAS, PMAIP1, PIK3R3, BIRC5, CYCS, BRCA1, BID, BIRC2, MAP3K5, BIRC3	607	75	8534	2.249489291598023	0.9953702590313074	0.16156706880271265	0.14883754216977166
IL13	GOTERM_BP_DIRECT	GO:0010888~negative regulation of lipid storage	4	0.3327787021	0.0166946245	NFKBIA, IL6, CLSTN3, ITGAV	1119	10	19478	6.962645218945488	1	0.43355434050110725	0.42535879754180983
IL13	GOTERM_BP_DIRECT	GO:0002819~regulation of adaptive immune response	4	0.3327787021	0.0166946245	IL4I1, DUSP10, IRF1, TNFSF4	1119	10	19478	6.962645218945488	1	0.43355434050110725	0.42535879754180983
IL13	GOTERM_BP_DIRECT	GO:0051292~nuclear pore complex assembly	4	0.3327787021	0.0166946245	NDC1, NUP205, NUP107, NUP153	1119	10	19478	6.962645218945488	1	0.43355434050110725	0.42535879754180983
IL13	GOTERM_BP_DIRECT	GO:1904668~positive regulation of ubiquitin protein ligase activity	4	0.3327787021	0.0166946245	CDC20, UBE2C, UBE2S, PLK1	1119	10	19478	6.962645218945488	1	0.43355434050110725	0.42535879754180983
IL13	GOTERM_BP_DIRECT	GO:0150077~regulation of neuroinflammatory response	4	0.3327787021	0.0166946245	IL6, SPHK1, TNFRSF1B, MMP9	1119	10	19478	6.962645218945488	1	0.43355434050110725	0.42535879754180983
IL13	GOTERM_BP_DIRECT	GO:0007259~cell surface receptor signaling pathway via JAK-STAT	9	0.7487520798	0.0171700075	SOCS2, IFNAR2, IL15RA, IL6, CNTF, CSF2, TSLP, CCL2, JAK3	1119	58	19478	2.701026162521956	1	0.44321374827093846	0.43483561207258464
IL13	GOTERM_BP_DIRECT	GO:0010165~response to X-ray	5	0.4159733777	0.0173045277	BLM, RAD51, MSH2, XRCC2, BRCA2	1119	18	19478	4.835170290934366	1	0.44342588000201205	0.4350437338456147
IL13	GOTERM_BP_DIRECT	GO:0051028~mRNA transport	12	0.9983361064	0.0173851920	NDC1, NUP205, NUP107, SEH1L, NUP188, NUP85, NUP50, NUP62, NUP35, NUP153, NUP88, SRSF7	1119	93	19478	2.2460145867566084	1	0.44342588000201205	0.4350437338456147
IL13	GOTERM_BP_DIRECT	GO:0006284~base-excision repair	7	0.5823627287	0.0177975667	POLQ, FEN1, NEIL3, LIG1, PARP2, HMGA1, DNA2	1119	37	19478	3.2931430089607034	1	0.44745186747191046	0.4389936174683574
IL13	GOTERM_BP_DIRECT	GO:0072719~cellular response to cisplatin	3	0.2495840266	0.0182739969	RAD51, DDX11, TIMELESS	1119	4	19478	13.054959785522788	1	0.44745186747191046	0.4389936174683574
IL13	GOTERM_BP_DIRECT	GO:0046105~thymidine biosynthetic process	3	0.2495840266	0.0182739969	DHFR, DTYMK, TK1	1119	4	19478	13.054959785522788	1	0.44745186747191046	0.4389936174683574

IL13	GOTERM_BP_DIRECT	GO:0043137~DNA replication, removal of RNA primer	3	0.2495840266	0.0182739969	FEN1, RNASEH2A, DNA2	1119	4	19478	13.054959785522788	1	0.44745186747191046	0.4389936174683574
IL13	GOTERM_BP_DIRECT	GO:0043111~replication fork arrest	3	0.2495840266	0.0182739969	TIPIN, SLFN11, TIMELESS	1119	4	19478	13.054959785522788	1	0.44745186747191046	0.4389936174683574
IL13	GOTERM_BP_DIRECT	GO:0042148~DNA strand invasion	3	0.2495840266	0.0182739969	RAD51, XRCC2, DMC1	1119	4	19478	13.054959785522788	1	0.44745186747191046	0.4389936174683574
IL13	GOTERM_BP_DIRECT	GO:0045003~double-strand break repair via synthesis-dependent strand annealing	3	0.2495840266	0.0182739969	FANCM, XRCC3, RAD54L	1119	4	19478	13.054959785522788	1	0.44745186747191046	0.4389936174683574
IL13	GOTERM_BP_DIRECT	GO:0030593~neutrophil chemotaxis	9	0.7487520798	0.0188963193	CXCL6, CXADR, CXCL8, CXCL1, CXCL3, SLC37A4, CXCL2, CXCL5, CX3CL1	1119	59	19478	2.6552460580724317	1	0.4600609574502489	0.4513643559208509
IL13	UP_KW_BIOLOGICAL_PROCESS	KW-0469~Meiosis	15	1.2479201331	0.019490175	MND1, FBXO43, SMC1A, PSMC3IP, RAD51AP1, SGO2, STAG3, EXO1, DMC1, CDK2, RBBP8, TESMIN, NEK2, BUB3, TRIP13	764	115	11523	1.9672774869109948	0.9312195206811128	0.150352656	0.13498815128358602
IL13	UP_KW_BIOLOGICAL_PROCESS	KW-0945~Host-virus interaction	61	5.0748752079	0.0197543636	EIF4A1, SUV39H1, JPT2, HTR2A, CLDN1, CX3CL1, ICAM1, IFIH1, TRIM6, ZC3H12A, XPO5, RPS6KA1, NUP62, ITGAV, KPNA2, IKBKE, TNFRSF4, MAP3K5, HSP90AA1, CXADR, ANXA2, ITGA2, DDX11, PLA2G4C, HLA-B, TAP2, TAP1, VRK1, CDC25C, PDIA4, CCNA2, PSMA3, RBL1, TRAF3, BIRC5, CCDC86, EPHA2, PCNA, SLC20A2, CENPA, LRP8, PSMB10, HSPD1, SYNGR2, E2F1, BUB1, CENPU, HSPA8, NUP153, APLN, PSMB9, SRPK1, NFKBIA, FAM111A, POLA1, KRT18, CDK1, ABCE1, ULBP2, NECTIN1, TP73	764	695	11523	1.3237843233266788	0.933694614	0.150352656	0.13498815128358602
IL13	GOTERM_BP_DIRECT	GO:0007623~circadian rhythm	10	0.8319467554	0.019885378	KLF10, SUV39H2, CLDN4, SUV39H1, BHLHE40, COL6A1, TIMELESS, ID3, ADCY1, TYMS	1119	71	19478	2.451635640473763	1	0.47940747551432533	0.4703451638418258
IL13	KEGG_PATHWAY	hsa04981:Folate transport and metabolism	7	0.5823627287	0.0200009656	DHFR, ABCC3, MTHFD1, SHMT1, TYMS, SLC19A1, ABCG2	607	31	8534	3.1746824679810812	0.9987280683791327	0.1941270192527201	0.1788321631903846
IL13	GOTERM_BP_DIRECT	GO:0010332~response to gamma radiation	6	0.4991680532	0.0201373018	TIGAR, PARP1, FANCD2, XRCC2, BRCA2, SOD2	1119	28	19478	3.729988510149368	1	0.47940747551432533	0.4703451638418258
IL13	GOTERM_BP_DIRECT	GO:0000132~establishment of mitotic spindle orientation	7	0.5823627287	0.02013847	GJA1, SAPCD2, PLK1, SPDL1, CENPA, NDC80, MAD2L1	1119	38	19478	3.206481350830159	1	0.47940747551432533	0.4703451638418258
IL13	GOTERM_BP_DIRECT	GO:2001238~positive regulation of extrinsic apoptotic signaling pathway	7	0.5823627287	0.02013847	CYLD, TNFRSF12A, G0S2, TNFSF11, TRAF2, TLR6, BID	1119	38	19478	3.206481350830159	1	0.47940747551432533	0.4703451638418258
IL13	GOTERM_BP_DIRECT	GO:0060348~bone development	9	0.7487520798	0.0207430590	PTGER4, TTC9, GJA1, BMP2, COL6A1, NSD2, STC1, SULF1, TMEM38B	1119	60	19478	2.6109919571045577	1	0.4910718679560794	0.48178906251746967
IL13	BIOCARTA	h_LairPathway:Cells and Molecules involved in local acute inflammatory response	6	0.4991680532	0.020867161	C3, IL6, VCAM1, CXCL8, ITGA4, ICAM1	162	17	1622	3.5337690631808276	0.9843036578199779	0.4544403959511234	0.44748467560493266
IL13	GOTERM_BP_DIRECT	GO:0042730~fibrinolysis	5	0.4159733777	0.0209453632	SERPINB2, ANXA2, PLAU, SERPINE1, GP1BA	1119	19	19478	4.580687644043083	1	0.49205083231237856	0.48274952136318305

IL13	GOTERM_BP_DIRECT	GO:1990830~cellular response to leukemia inhibitory factor	13	1.0815307820	0.0210140728	TLE4, HELLS, GLDC, SHMT1, VEGFC, SRM, ICAM1, MAT2A, NUP35, TNFSF11, MYBL2, SRSF7, INA	1119	108	19478	2.095240459404892	1	0.49205083231237856	0.48274952136318305
IL13	KEGG_PATHWAY	hsa05167:Kaposi sarcoma-associated herpesvirus infection	23	1.9134775374	0.021423274	IFNAR2, CXCL8, CSF2, HLA-B, PIK3R3, ITPR2, CXCL1, TRAF2, CXCL3, HLA-F, CXCL2, ICAM1, C3, NFKBIA, IL6, TRAF3, E2F1, FAS, E2F2, CYCS, E2F3, BID, IKBKE	607	196	8534	1.649816763608244	0.9992123900646949	0.20199087155050302	0.18607643924652398
IL13	GOTERM_BP_DIRECT	GO:1990918~double-strand break repair involved in meiotic recombination	4	0.3327787021	0.0219903961	BRIP1, RAD51, FANCD2, DMC1	1119	11	19478	6.329677471768624	1	0.5038975805675054	0.4943723287528105
IL13	GOTERM_BP_DIRECT	GO:0001574~ganglioside biosynthetic process	4	0.3327787021	0.0219903961	ST8SIA4, ST8SIA6, B4GALNT1, ST6GALNAC5	1119	11	19478	6.329677471768624	1	0.5038975805675054	0.4943723287528105
IL13	GOTERM_BP_DIRECT	GO:0072711~cellular response to hydroxyurea	4	0.3327787021	0.0219903961	BLM, RAD51, DDX11, TIMELESS	1119	11	19478	6.329677471768624	1	0.5038975805675054	0.4943723287528105
IL13	GOTERM_BP_DIRECT	GO:0001780~neutrophil homeostasis	4	0.3327787021	0.0219903961	MTHFD1, CSF1, SLC37A4, SH2B3	1119	11	19478	6.329677471768624	1	0.5038975805675054	0.4943723287528105
IL13	GOTERM_BP_DIRECT	GO:0006338~chromatin remodeling	53	4.4093178036	0.0253720671	SUV39H2, CHD7, BUB1B, HR, BRCA1, BRCA2, DUSP10, BCL7A, UBASH3B, DPF1, RPS6KA1, CHEK1, PIM3, NEK2, PIM2, TP63, USP49, PPTC7, PRMT1, VRK1, BAZ1A, PASK, HASPIN, MASTL, CDC25C, CDC25A, CIT, ERN1, PKM, SLFN11, MELK, PGP, ALPK2, INO80C, DDX21, PKMYT1, AURKA, NUAK2, NSD2, BUB1, BARD1, KDM4D, CDC7, DCLK1, SRPK1, PTPRE, MYBBP1A, POLE3, CDK2, CDK1, CDKN3, PTPN3, ATR	1119	686	19478	1.3448257893735818	1	0.5782941905843968	0.5673626084519963
IL13	BIOCARTA	h_caspasePathway:Caspase Cascade in Apoptosis	7	0.5823627287	0.0257778363	PARP1, ARHGDIB, CYCS, LMNB2, BIRC2, LMNB1, BIRC3	162	24	1622	2.920267489711934	0.9941705565008103	0.505245593	0.4975122424234291
IL13	UP_KW_BIOLOGICAL_PROCESS	KW-0233~DNA recombination	13	1.0815307820	0.0261180694	INO80C, PIF1, LIG1, XRCC2, XRCC3, MND1, BRCA1, BRCA2, PALB2, PSMC3IP, RAD51AP1, RAD51, EME1	764	97	11523	2.0213607167917096	0.9726562619626125	0.1883250272958852	0.16908013399557578
IL13	GOTERM_BP_DIRECT	GO:0000027~ribosomal large subunit assembly	4	0.3327787021	0.028092278	BOP1, MRTO4, NOP2, RRS1	1119	12	19478	5.802204349121239	1	0.6095662185809234	0.5980434965960798
IL13	GOTERM_BP_DIRECT	GO:0043589~skin morphogenesis	4	0.3327787021	0.028092278	ERRFI1, ITGA2, ITGA6, TP63	1119	12	19478	5.802204349121239	1	0.6095662185809234	0.5980434965960798
IL13	GOTERM_BP_DIRECT	GO:0007194~negative regulation of adenylate cyclase activity	4	0.3327787021	0.028092278	GRM2, GABBR2, EDNRB, AKAP5	1119	12	19478	5.802204349121239	1	0.6095662185809234	0.5980434965960798
IL13	GOTERM_BP_DIRECT	GO:0045842~positive regulation of mitotic metaphase/anaphase transition	4	0.3327787021	0.028092278	CDC20, ESPL1, UBE2C, DLGAP5	1119	12	19478	5.802204349121239	1	0.6095662185809234	0.5980434965960798
IL13	GOTERM_BP_DIRECT	GO:0090656~t-circle formation	4	0.3327787021	0.028092278	BLM, EXO1, XRCC3, DNA2	1119	12	19478	5.802204349121239	1	0.6095662185809234	0.5980434965960798
IL13	GOTERM_BP_DIRECT	GO:0015810~aspartate transmembrane transport	4	0.3327787021	0.028092278	LRRC8C, LRRC8D, LRRC8B, SLC25A13	1119	12	19478	5.802204349121239	1	0.6095662185809234	0.5980434965960798

IL13	GOTERM_BP_DIRECT	GO:1990542~mitochondrial transmembrane transport	4	0.3327787021	0.028092278	UCP3, COL6A1, SFXN4, SLC25A13	1119	12	19478	5.802204349121239	1	0.6095662185809234	0.5980434965960798
IL13	GOTERM_BP_DIRECT	GO:0010389~regulation of G2/M transition of mitotic cell cycle	4	0.3327787021	0.028092278	CENPF, CDK2, KIF14, AURKA	1119	12	19478	5.802204349121239	1	0.6095662185809234	0.5980434965960798
IL13	GOTERM_BP_DIRECT	GO:0019441~L-tryptophan catabolic process to kynurenine	3	0.2495840266	0.0293047003	TDO2, KYNU, IDO1	1119	5	19478	10.443967828418232	1	0.6095662185809234	0.5980434965960798
IL13	GOTERM_BP_DIRECT	GO:0006297~nucleotide-excision repair, DNA gap filling	3	0.2495840266	0.0293047003	POLD3, POLD1, POLE	1119	5	19478	10.443967828418232	1	0.6095662185809234	0.5980434965960798
IL13	GOTERM_BP_DIRECT	GO:0045835~negative regulation of meiotic nuclear division	3	0.2495840266	0.0293047003	LIF, FBXO5, FBXO43	1119	5	19478	10.443967828418232	1	0.6095662185809234	0.5980434965960798
IL13	GOTERM_BP_DIRECT	GO:0060676~ureteric bud formation	3	0.2495840266	0.0293047003	GDNF, NOG, HS2ST1	1119	5	19478	10.443967828418232	1	0.6095662185809234	0.5980434965960798
IL13	GOTERM_BP_DIRECT	GO:0032201~telomere maintenance via semi-conservative replication	3	0.2495840266	0.0293047003	BLM, FEN1, DNA2	1119	5	19478	10.443967828418232	1	0.6095662185809234	0.5980434965960798
IL13	GOTERM_BP_DIRECT	GO:0002158~osteoclast proliferation	3	0.2495840266	0.0293047003	CSF1, TNFSF11, JUNB	1119	5	19478	10.443967828418232	1	0.6095662185809234	0.5980434965960798
IL13	GOTERM_BP_DIRECT	GO:0043009~chordate embryonic development	3	0.2495840266	0.0293047003	CENPU, CHD7, BRCA1	1119	5	19478	10.443967828418232	1	0.6095662185809234	0.5980434965960798
IL13	GOTERM_BP_DIRECT	GO:0009113~purine nucleobase biosynthetic process	3	0.2495840266	0.0293047003	SHMT1, PPAT, PAICS	1119	5	19478	10.443967828418232	1	0.6095662185809234	0.5980434965960798
IL13	GOTERM_BP_DIRECT	GO:0061041~regulation of wound healing	3	0.2495840266	0.0293047003	CADM4, TNFRSF12A, PLAU	1119	5	19478	10.443967828418232	1	0.6095662185809234	0.5980434965960798
IL13	GOTERM_BP_DIRECT	GO:0051664~nuclear pore localization	3	0.2495840266	0.0293047003	NDC1, LMNB2, LMNB1	1119	5	19478	10.443967828418232	1	0.6095662185809234	0.5980434965960798
IL13	GOTERM_BP_DIRECT	GO:0044818~mitotic G2/M transition checkpoint	5	0.4159733777	0.0295420739	BARD1, CHEK1, RBBP8, BRCA1, ATR	1119	21	19478	4.144431677943742	1	0.6115352020573748	0.5999752600814944
IL13	GOTERM_BP_DIRECT	GO:0051276~chromosome organization	6	0.4991680532	0.0302422149	CENPW, PTTG1, HAT1, RAD54L, CDCA8, TOPBP1	1119	31	19478	3.3690218801349134	1	0.6230187068017993	0.6112416904071795
IL13	KEGG_PATHWAY	hsa04514:Cell adhesion molecules	19	1.5806988352	0.0309128350	CD274, CNTNAP1, VCAM1, ITGA4, HLA-B, NRXN3, PDCD1LG2, L1CAM, HLA-F, CLDN1, ICAM1, CLDN4, SLITRK2, SDC1, ITGAV, ITGA6, CD58, CLDN16, NECTIN1	607	158	8534	1.690676287197881	0.9999683963146394	0.28336765466646835	0.26104171823820116
IL13	BIOCARTA	h_cellcyclePathway:Cyclins and Cell Cycle Regulation	7	0.5823627287	0.031213132	CCNB1, RBL1, CCNE1, CDK2, CDK1, E2F1, CDC25A	162	25	1622	2.8034567901234566	0.9980637386999974	0.5347027799343902	0.5265185537109046
IL13	GOTERM_BP_DIRECT	GO:0006986~response to unfolded protein	9	0.7487520798	0.031916867	HSPA8, HSP90AA1, HSPH1, FICD, HSPA4L, DNAJB9, HSPE1, HSPD1, MANF	1119	65	19478	2.4101464219426685	1	0.6541707273991253	0.6418048396699936

IL13	GOTERM_BP_DIRECT	GO:0071456~cellular response to hypoxia	15	1.2479201331	0.032059709	SUV39H2, EGLN3, TIGAR, SUV39H1, GATA6, STC1, CCNA2, BRIP1, PMAIP1, ADAM8, HYOU1, NDP, SLC29A1, KCNK2, NKX3-1	1119	141	19478	1.8517673454642254	1	0.6541707273991253	0.6418048396699936
IL13	BIOCARTA	h_rbPathway:RB Tumor Suppressor/Checkpoint Signaling in response to DNA damage	5	0.4159733777	0.032736905	CHEK1, CDK2, CDK1, CDC25C, CDC25A	162	13	1622	3.8509021842355176	0.9985799953877281	0.5347027799343902	0.5265185537109046
IL13	GOTERM_BP_DIRECT	GO:0006935~chemotaxis	14	1.1647254575	0.0341903978	CXCL6, CXCL8, PLAUR, CXCL1, L1CAM, CXCL2, CXCL5, CX3CL1, TYMP, FOSL1, PLAU, ANOS1, CCL2, DOCK2	1119	129	19478	1.889089788085985	1	0.6910327086787454	0.6779700133688322
IL13	GOTERM_BP_DIRECT	GO:0007159~leukocyte cell-cell adhesion	6	0.4991680532	0.0342054342	NT5E, VCAM1, ITGA4, OLR1, EZR, ICAM1	1119	32	19478	3.263739946380697	1	0.6910327086787454	0.6779700133688322
IL13	GOTERM_BP_DIRECT	GO:0031589~cell-substrate adhesion	5	0.4159733777	0.034514291	ITGA2, ITGAV, ITGA6, CORO1A, EPHB1	1119	22	19478	3.95604842	1	0.6910327086787454	0.6779700133688322
IL13	GOTERM_BP_DIRECT	GO:0035435~phosphate ion transmembrane transport	4	0.3327787021	0.0349951220	SLC37A2, SLC20A2, SLC25A10, SLC37A4	1119	13	19478	5.355880937650375	1	0.6910327086787454	0.6779700133688322
IL13	GOTERM_BP_DIRECT	GO:1903238~positive regulation of leukocyte tethering or rolling	4	0.3327787021	0.0349951220	ITGA4, GP1BA, CHST2, FUT4	1119	13	19478	5.355880937650375	1	0.6910327086787454	0.6779700133688322
IL13	GOTERM_BP_DIRECT	GO:0002474~antigen processing and presentation of peptide antigen via MHC class I	4	0.3327787021	0.0349951220	ERAP2, ERAP1, HLA-B, HLA-F	1119	13	19478	5.355880937650375	1	0.6910327086787454	0.6779700133688322
IL13	GOTERM_BP_DIRECT	GO:0046425~regulation of receptor signaling pathway via JAK-STAT	4	0.3327787021	0.0349951220	IFNAR2, DOT1L, HES1, JAK3	1119	13	19478	5.355880937650375	1	0.6910327086787454	0.6779700133688322
IL13	GOTERM_BP_DIRECT	GO:0006468~protein phosphorylation	25	2.0798668885	0.035298364	PKN3, AURKA, NUAK2, IRAK2, CHEK1, RPS6KA1, CCL2, PIM3, NEK2, MAP3K9, PIM2, IP6K3, MAP3K5, PLK4, PLK2, PLK1, VRK1, PASK, HASPIN, DCLK1, SRPK1, ERN1, CCNE1, CDK2, BIRC5	1119	282	19478	1.543139454553521	1	0.6938233510174171	0.6807079037753143
IL13	GOTERM_BP_DIRECT	GO:0042127~regulation of cell population proliferation	16	1.3311148086	0.036518971	EGLN3, CADM4, FLT1, TNFRSF9, CDCA7, PRDM1, FOXM1, PTGS1, NFKBIA, CHST11, PLAU, CHEK1, JUNB, BIRC2, BRICD5, EZH2	1119	157	19478	1.7739223487759204	1	0.7145378608318039	0.7010308440926263
IL13	GOTERM_BP_DIRECT	GO:0071347~cellular response to interleukin-1	8	0.6655574043	0.036974243	CXCL8, ZC3H12A, DAB2IP, ANKRD1, CCL2, HAS2, HES1, NKX3-1	1119	55	19478	2.5318709887074498	1	0.7168987814193646	0.7033471358429425
IL13	GOTERM_BP_DIRECT	GO:0097191~extrinsic apoptotic signaling pathway	8	0.6655574043	0.036974243	IL33, KRT18, PARP2, FAS, G0S2, TNFRSF10A, BID, TNFRSF1B	1119	55	19478	2.5318709887074498	1	0.7168987814193646	0.7033471358429425
IL13	KEGG_PATHWAY	hsa05164:Influenza A	20	1.6638935108	0.037627071	IFNAR2, IL33, NXT1, CXCL8, PIK3R3, TNFRSF10B, TNFRSF10A, ICAM1, IFIH1, NFKBIA, IL6, TRAF3, OAS3, FAS, CCL2, CYCS, BID, KPNA2, IKBKE, NFKBIB	607	173	8534	1.6253535343916352	0.999996813	0.3355927947162733	0.30915215028408205
IL13	UP_KW_BIOLOGICAL_PROCESS	KW-0617~Plasminogen activation	3	0.2495840266	0.0382872387	SERPINB2, PLAU, ENO1	764	5	11523	9.049476439790576	0.9950549404857514	0.24594452289794158	0.2208115059594658
IL13	UP_KW_BIOLOGICAL_PROCESS	KW-0228~DNA excision	3	0.2495840266	0.0382872387	POLD3, EXO1, POLD1	764	5	11523	9.049476439790576	0.9950549404857514	0.24594452289794158	0.2208115059594658

IL13	GOTERM_BP_DIRECT	GO:0035924~cellular response to vascular endothelial growth factor stimulus	6	0.4991680532	0.038475586	ERN1, DLL4, FLT1, VCAM1, SPHK1, DAB2IP	1119	33	19478	3.164838735884312	1	0.7426481334254008	0.7286097439720852
IL13	UP_KW_BIOLOGICAL_PROCESS	KW-0554~One-carbon metabolism	5	0.4159733777	0.039494741	DHFR, DHFRP1, MAT2A, MTHFD1, SHMT1	764	20	11523	3.7706151832460733	0.9958316362754875	0.24594452289794158	0.2208115059594658
IL13	GOTERM_BP_DIRECT	GO:0009311~oligosaccharide metabolic process	5	0.4159733777	0.039941946	ST8SIA1, ST8SIA4, ST8SIA6, ST6GALNAC5, FUT4	1119	23	19478	3.784046314644286	1	0.7602382877013243	0.7458673889139713
IL13	GOTERM_BP_DIRECT	GO:0006259~DNA metabolic process	5	0.4159733777	0.039941946	NT5E, AEN, TOPBP1, KPNA2, NME1	1119	23	19478	3.784046314644286	1	0.7602382877013243	0.7458673889139713
IL13	GOTERM_BP_DIRECT	GO:0009636~response to toxic substance	10	0.8319467554	0.042171666	BRIP1, RAD51, COL6A1, GATA6, CDK1, SDC1, TYMS, CLDN1, SLC19A1, TLR2	1119	81	19478	2.148964573748607	1	0.7602382877013243	0.7458673889139713
IL13	GOTERM_BP_DIRECT	GO:0006569~L-tryptophan catabolic process	3	0.2495840266	0.042302866	IL4I1, KYNU, IDO1	1119	6	19478	8.703306523681858	1	0.7602382877013243	0.7458673889139713
IL13	GOTERM_BP_DIRECT	GO:0022614~membrane to membrane docking	3	0.2495840266	0.042302866	VCAM1, EZR, ICAM1	1119	6	19478	8.703306523681858	1	0.7602382877013243	0.7458673889139713
IL13	GOTERM_BP_DIRECT	GO:0045143~homologous chromosome segregation	3	0.2495840266	0.042302866	PTTG1, ESPL1, PLK1	1119	6	19478	8.703306523681858	1	0.7602382877013243	0.7458673889139713
IL13	GOTERM_BP_DIRECT	GO:0010571~positive regulation of nuclear cell cycle DNA replication	3	0.2495840266	0.042302866	DBF4, CDC7, DBF4B	1119	6	19478	8.703306523681858	1	0.7602382877013243	0.7458673889139713
IL13	GOTERM_BP_DIRECT	GO:0044770~cell cycle phase transition	3	0.2495840266	0.042302866	TIPIN, TIMELESS, CDC7	1119	6	19478	8.703306523681858	1	0.7602382877013243	0.7458673889139713
IL13	GOTERM_BP_DIRECT	GO:0060770~negative regulation of epithelial cell proliferation involved in prostate gland development	3	0.2495840266	0.042302866	EAF2, NKX3-1, WDR77	1119	6	19478	8.703306523681858	1	0.7602382877013243	0.7458673889139713
IL13	GOTERM_BP_DIRECT	GO:1902751~positive regulation of cell cycle G2/M phase transition	3	0.2495840266	0.042302866	ATAD5, CDC25C, CDC25A	1119	6	19478	8.703306523681858	1	0.7602382877013243	0.7458673889139713
IL13	GOTERM_BP_DIRECT	GO:0007100~mitotic centrosome separation	3	0.2495840266	0.042302866	NUP62, KIF11, AURKA	1119	6	19478	8.703306523681858	1	0.7602382877013243	0.7458673889139713
IL13	GOTERM_BP_DIRECT	GO:0006189~'de novo' IMP biosynthetic process	3	0.2495840266	0.042302866	PPAT, PAICS, PFAS	1119	6	19478	8.703306523681858	1	0.7602382877013243	0.7458673889139713
IL13	GOTERM_BP_DIRECT	GO:0031536~positive regulation of exit from mitosis	3	0.2495840266	0.042302866	UBE2C, CDCA5, BIRC5	1119	6	19478	8.703306523681858	1	0.7602382877013243	0.7458673889139713

IL13	GOTERM_BP_DIRECT	GO:0002693~positive regulation of cellular extravasation	3	0.2495840266	0.042302866	ADAM8, THY1, ICAM1	1119	6	19478	8.703306523681858	1	0.7602382877013243	0.7458673889139713
IL13	GOTERM_BP_DIRECT	GO:0031503~protein-containing complex localization	4	0.3327787021	0.042685725	SEH1L, BIRC5, FKBP4, EZR	1119	14	19478	4.973318013532491	1	0.7602382877013243	0.7458673889139713
IL13	GOTERM_BP_DIRECT	GO:0003222~ventricular trabecula myocardium morphogenesis	4	0.3327787021	0.042685725	DLL4, HEG1, CHD7, NRG1	1119	14	19478	4.973318013532491	1	0.7602382877013243	0.7458673889139713
IL13	GOTERM_BP_DIRECT	GO:0051058~negative regulation of small GTPase mediated signal transduction	4	0.3327787021	0.042685725	ARHGAP22, SLIT2, CGNL1, ARHGAP45	1119	14	19478	4.973318013532491	1	0.7602382877013243	0.7458673889139713
IL13	GOTERM_BP_DIRECT	GO:0030278~regulation of ossification	4	0.3327787021	0.042685725	PTGER4, EGR2, CSF1, SIX2	1119	14	19478	4.973318013532491	1	0.7602382877013243	0.7458673889139713
IL13	GOTERM_BP_DIRECT	GO:0042771~intrinsic apoptotic signaling pathway in response to DNA damage by p53 class mediator	6	0.4991680532	0.043056595	MSH2, ATAD5, AEN, BRCA2, TP63, TP73	1119	34	19478	3.071755243652421	1	0.7602382877013243	0.7458673889139713
IL13	GOTERM_BP_DIRECT	GO:0030071~regulation of mitotic metaphase/anaphase transition	6	0.4991680532	0.043056595	CENPE, BCL7A, UBE2C, DPF1, PLK1, CDC6	1119	34	19478	3.071755243652421	1	0.7602382877013243	0.7458673889139713
IL13	GOTERM_BP_DIRECT	GO:0046718~symbiont entry into host cell	12	0.9983361064	0.0432049265	CXADR, SLC20A2, ITGA2, CDK1, NUP153, ITGAV, HTR2A, CLDN1, TNFRSF4, EPHA2, ICAM1, NECTIN1	1119	107	19478	1.9521435193305106	1	0.7602382877013243	0.7458673889139713
IL13	GOTERM_BP_DIRECT	GO:0000226~microtubule cytoskeleton organization	15	1.2479201331	0.043290115	GAS2L3, HAUS6, TUBB4B, TUBG1, KNSTRN, TUBA1C, TUBA1B, TUBB2B, TTLL4, PRC1, FIGNL1, CDK1, TACC3, BIRC5, CDK5R1	1119	147	19478	1.7761850048330323	1	0.7602382877013243	0.7458673889139713
IL13	GOTERM_BP_DIRECT	GO:0045786~negative regulation of cell cycle	8	0.6655574043	0.0437036334	BARD1, CDT1, BMP2, HPGD, SUV39H1, GMNN, BRCA1, ETS1	1119	57	19478	2.4430334101563114	1	0.7643676299047626	0.7499186735401686
IL13	GOTERM_BP_DIRECT	GO:2001243~negative regulation of intrinsic apoptotic signaling pathway	5	0.4159733777	0.045826342	HELLS, FIGNL1, PLAUR, PPIF, MMP9	1119	24	19478	3.626377718200774	1	0.798235273	0.7831461112882443
IL13	GOTERM_BP_DIRECT	GO:0006457~protein folding	17	1.4143094841	0.046400736	HSP90AA2P, HSPA8, HSP90AA1, HSPA4L, HSPE1, HSPD1, PDIA4, TAPBP, CLGN, HSP90B2P, HSPH1, ANP32E, PPIF, HYOU1, QSOX2, FKBP4, CCT5	1119	176	19478	1.6813205784385408	1	0.804968235	0.7897517991978825
IL13	GOTERM_BP_DIRECT	GO:0070098~chemokine-mediated signaling pathway	9	0.7487520798	0.046626216	CXCL6, CXCL8, GPR75, CCL2, CXCL1, CXCL3, CXCL2, CXCL5, CX3CL1	1119	70	19478	2.237993106089621	1	0.805618292	0.7903895679049873
IL13	KEGG_PATHWAY	hsa04621:NOD-like receptor signaling pathway	21	1.7470881863	0.046750124	IFNAR2, HSP90AA1, CXCL8, TNFAIP3, ITPR2, CXCL1, TRAF2, CXCL3, CXCL2, PANX1, NFKBIA, IL6, TRAF3, OAS3, NAMPT, CCL2, IKBKE, GBP4, BIRC2, BIRC3, NFKBIB	607	189	8534	1.562145341387516	0.9999998625230206	0.40598791767870973	0.37400099083129623
IL13	GOTERM_BP_DIRECT	GO:0045931~positive regulation of mitotic cell cycle	6	0.4991680532	0.047951219	CCNB1, SPHK1, BIRC5, BRCA2, AURKA, NKX3-1	1119	35	19478	2.9839908081194944	1	0.8251846347767443	0.8095860454145702

IL13	GOTERM_BP_DIRECT	GO:0001649~osteoblast differentiation	14	1.1647254575	0.048979413	WNT10B, SEMA7A, COL11A2, NOG, DDX21, HSPE1, GTPBP4, GJA1, BMP2, MYBBP1A, FIGNL1, COL6A1, JUNB, EPHA2	1119	136	19478	1.7918572254639122	1	0.8395071419637489	0.8236378120923221
IL13	KEGG_PATHWAY	hsa04512:ECM-receptor interaction	12	0.9983361064	0.049611676	LAMB3, ITGA4, COL4A4, ITGA2, COL6A1, SDC1, COL6A6, ITGAV, ITGA6, GP1BA, LAMC2, HMMR	607	89	8534	1.895637043	0.9999999490246343	0.4197911078270824	0.3867166569073729
IL13	GOTERM_BP_DIRECT	GO:0030183~B cell differentiation	9	0.7487520798	0.0500218782	VCAM1, MSH2, ITGA4, NFAM1, PIK3R3, DNAJB9, HDAC9, JAK3, EZH2	1119	71	19478	2.2064720764263868	1	0.8539591559858516	0.8378166375179742
IL13	GOTERM_BP_DIRECT	GO:0031103~axon regeneration	4	0.3327787021	0.05114411	DHFR, DHFRP1, GAP43, CSPG5	1119	15	19478	4.641763479296991	1	0.8594216207132087	0.8431758444523522
IL13	GOTERM_BP_DIRECT	GO:0033147~negative regulation of intracellular estrogen receptor signaling pathway	4	0.3327787021	0.05114411	BRCA1, CYP7B1, CNOT9, TP63	1119	15	19478	4.641763479296991	1	0.8594216207132087	0.8431758444523522
IL13	GOTERM_BP_DIRECT	GO:0070266~necroptotic process	4	0.3327787021	0.05114411	CYLD, MLKL, PPIF, BIRC2	1119	15	19478	4.641763479296991	1	0.8594216207132087	0.8431758444523522
IL13	GOTERM_BP_DIRECT	GO:0046655~folic acid metabolic process	4	0.3327787021	0.05114411	DHFR, DHFRP1, SHMT1, SLC19A1	1119	15	19478	4.641763479296991	1	0.8594216207132087	0.8431758444523522
IL13	BIOCARTA	H_cdc25Pathway:cdc25 and chk1 Regulatory Pathway in response to DNA damage	4	0.3327787021	0.051491648	CHEK1, CDK1, CDC25C, CDC25A	162	9	1622	4.449931412894376	0.9999700008911434	0.7763356233545304	0.7644529352419611
IL13	GOTERM_BP_DIRECT	GO:0043122~regulation of canonical NF-kappaB signal transduction	6	0.4991680532	0.053161065	TRAF4, TRAF3, TNIP3, DAB2IP, TRAF1, NFKBIB	1119	36	19478	2.901102175	1	0.8898248616385072	0.8730043683379893
IL13	KEGG_PATHWAY	hsa05203:Viral carcinogenesis	22	1.8302828618	0.0564897235	EGR2, HLA-B, PIK3R3, TRAF2, TRAF1, HLA-F, HDAC9, NFKB2, C3, NFKBIA, CCNA2, CDC20, PKM, RBL1, CCNE2, CCNE1, TRAF3, CHEK1, CDK2, CDK1, PMAIP1, JAK3	607	205	8534	1.5088037931450153	0.999999995	0.46604021969740494	0.4293218993576094
IL13	GOTERM_BP_DIRECT	GO:0040036~regulation of fibroblast growth factor receptor signaling pathway	3	0.2495840266	0.057006707	HHIP, NOG, SULF1	1119	7	19478	7.459977020298736	1	0.9080808163410383	0.8909152279807993
IL13	GOTERM_BP_DIRECT	GO:0006269~DNA replication, synthesis of primer	3	0.2495840266	0.057006707	POLA1, POLA2, PRIM1	1119	7	19478	7.459977020298736	1	0.9080808163410383	0.8909152279807993
IL13	GOTERM_BP_DIRECT	GO:0035330~regulation of hippo signaling	3	0.2495840266	0.057006707	NUAK2, WWC1, MOB3B	1119	7	19478	7.459977020298736	1	0.9080808163410383	0.8909152279807993
IL13	GOTERM_BP_DIRECT	GO:0010994~free ubiquitin chain polymerization	3	0.2495840266	0.057006707	TRIM6, UBE2C, UBE2S	1119	7	19478	7.459977020298736	1	0.9080808163410383	0.8909152279807993
IL13	GOTERM_BP_DIRECT	GO:0000212~meiotic spindle organization	3	0.2495840266	0.057006707	ESPL1, TUBG1, AURKA	1119	7	19478	7.459977020298736	1	0.9080808163410383	0.8909152279807993
IL13	GOTERM_BP_DIRECT	GO:1903438~positive regulation of mitotic cytokinetic process	3	0.2495840266	0.057006707	NUP62, KIF20B, ECT2	1119	7	19478	7.459977020298736	1	0.9080808163410383	0.8909152279807993

IL13	GOTERM_BP_DIRECT	GO:0034136~negative regulation of toll-like receptor 2 signaling pathway	3	0.2495840266	0.057006707	MFHAS1, TNFAIP3, TLR6	1119	7	19478	7.459977020298736	1	0.9080808163410383	0.8909152279807993
IL13	GOTERM_BP_DIRECT	GO:0007091~metaphase/anaphase transition of mitotic cell cycle	3	0.2495840266	0.057006707	PLK1, TACC3, BUB1B	1119	7	19478	7.459977020298736	1	0.9080808163410383	0.8909152279807993
IL13	GOTERM_BP_DIRECT	GO:0006929~substrate-dependent cell migration	3	0.2495840266	0.057006707	ITGA2, VEGFC, CSPG4	1119	7	19478	7.459977020298736	1	0.9080808163410383	0.8909152279807993
IL13	GOTERM_BP_DIRECT	GO:0140361~cyclic-GMP-AMP transmembrane import across plasma membrane	3	0.2495840266	0.057006707	LRRC8C, LRRC8B, SLC19A1	1119	7	19478	7.459977020298736	1	0.9080808163410383	0.8909152279807993
IL13	GOTERM_BP_DIRECT	GO:0097294~'de novo' XMP biosynthetic process	3	0.2495840266	0.057006707	PPAT, PAICS, PFAS	1119	7	19478	7.459977020298736	1	0.9080808163410383	0.8909152279807993
IL13	GOTERM_BP_DIRECT	GO:0098534~centriole assembly	3	0.2495840266	0.057006707	RBM14, NUP62, CEP85	1119	7	19478	7.459977020298736	1	0.9080808163410383	0.8909152279807993
IL13	GOTERM_BP_DIRECT	GO:0032466~negative regulation of cytokinesis	3	0.2495840266	0.057006707	E2F7, AURKB, E2F8	1119	7	19478	7.459977020298736	1	0.9080808163410383	0.8909152279807993
IL13	GOTERM_BP_DIRECT	GO:0046330~positive regulation of JNK cascade	11	0.9151414309	0.057673936	MFHAS1, SH3RF2, CRACR2A, TRAF4, GADD45A, WNT7B, CCDC88C, DAB2IP, TNFSF11, DKK1, MAP3K5	1119	99	19478	1.9340681163737465	1	0.9153067258668482	0.8980045450511622
IL13	GOTERM_BP_DIRECT	GO:0045088~regulation of innate immune response	5	0.4159733777	0.0589581595	IRF1, ERAP1, LRP8, BIRC2, BIRC3	1119	26	19478	3.347425586031484	1	0.9254055443566708	0.9079124640549462
IL13	GOTERM_BP_DIRECT	GO:0032212~positive regulation of telomere maintenance via telomerase	5	0.4159733777	0.0589581595	PKIB, HSP90AA1, DKC1, CCT5, ATR	1119	26	19478	3.347425586031484	1	0.9254055443566708	0.9079124640549462
IL13	GOTERM_BP_DIRECT	GO:0043507~positive regulation of JUN kinase activity	5	0.4159733777	0.0589581595	ERN1, DAB2IP, TRAF2, TLR6, MAP3K5	1119	26	19478	3.347425586031484	1	0.9254055443566708	0.9079124640549462
IL13	GOTERM_BP_DIRECT	GO:0008630~intrinsic apoptotic signaling pathway in response to DNA damage	7	0.5823627287	0.0602707628	E2F1, BRCA1, SOD2, TNFRSF1B, IKBKE, EPHA2, TP73	1119	49	19478	2.4866590067662457	1	0.9334906841434802	0.9158447692273491
IL13	GOTERM_BP_DIRECT	GO:0044027~negative regulation of gene expression via chromosomal CpG island methylation	4	0.3327787021	0.060344672	HELLS, DNMT1, UHRF1, BRCA1	1119	16	19478	4.351653261840929	1	0.9334906841434802	0.9158447692273491
IL13	GOTERM_BP_DIRECT	GO:0046007~negative regulation of activated T cell proliferation	4	0.3327787021	0.060344672	CD274, BTN2A2, PDCD1LG2, IDO1	1119	16	19478	4.351653261840929	1	0.9334906841434802	0.9158447692273491
IL13	GOTERM_BP_DIRECT	GO:0022407~regulation of cell-cell adhesion	4	0.3327787021	0.060344672	PODXL, ADAM8, CD24, JAK3	1119	16	19478	4.351653261840929	1	0.9334906841434802	0.9158447692273491

IL13	KEGG_PATHWAY	hsa05146:Amoebiasis	13	1.0815307820	0.06048187	CXCL8, CSF2, LAMB3, PIK3R3, CXCL1, LAMC2, SERPINB9, ADCY1, CXCL3, CXCL2, IL6, COL4A4, TLR2	607	103	8534	1.7744757761392174	0.9999999988552664	0.486805298	0.44845094128233803
IL13	KEGG_PATHWAY	hsa04820:Cytoskeleton in muscle cells	24	1.9966722129	0.064217259	DSP, COL27A1, MYBPC2, ITGA4, ITGA2, COL11A2, ANK2, ENO1, ENO3, LMNB2, MYPN, LMNB1, ACTC1, DIAPH3, COL4A4, COL5A3, COL6A1, ANKRD1, SDC1, COL6A6, ITGAV, ITGA6, MYOZ2, PDLIM4	607	232	8534	1.4544111799125148	1	0.5045641754464301	0.46481063435065073
IL13	GOTERM_BP_DIRECT	GO:0019221~cytokine-mediated signaling pathway	14	1.1647254575	0.064754382	TSLP, EBI3, LIFR, LRP8, CX3CL1, IL27RA, SOCS2, FOSL1, IL6, IL17REL, CCL2, TNFSF11, CNOT9, JAK3	1119	142	19478	1.7161449483316342	1	0.9981026205240665	0.9792353364488158
IL13	GOTERM_BP_DIRECT	GO:0070536~protein K63-linked deubiquitination	5	0.4159733777	0.066196248	USP13, CYLD, PSMD14, STAMBPL1, TNFAIP3	1119	27	19478	3.2234468606229107	1	1	0.9813258636788048
IL13	KEGG_PATHWAY	hsa05219:Bladder cancer	7	0.5823627287	0.067456979	CXCL8, E2F1, E2F2, E2F3, MMP9, TYMP, HBEGF	607	41	8534	2.4003696709125246	0.9999999999021121	0.5176930964914962	0.47690515555580254
IL13	BIOCARTA	h_g1Pathway:Cell Cycle: G1/S Check Point	7	0.5823627287	0.069263301	DHFR, CCNE1, CDK2, CDK1, E2F1, CDC25A, ATR	162	30	1622	2.3362139917695472	0.9999992774179983	0.9009008713808473	0.8871115723290995
IL13	GOTERM_BP_DIRECT	GO:0008637~apoptotic mitochondrial changes	4	0.3327787021	0.070257187	PPIF, PIM2, BID, HSPD1	1119	17	19478	4.095673658203228	1	1	0.9813258636788048
IL13	GOTERM_BP_DIRECT	GO:0085020~protein K6-linked ubiquitination	4	0.3327787021	0.070257187	BARD1, UBE2S, UBE2T, BRCA1	1119	17	19478	4.095673658203228	1	1	0.9813258636788048
IL13	GOTERM_BP_DIRECT	GO:0048246~macrophage chemotaxis	4	0.3327787021	0.070257187	EDNRB, NUP85, CCL2, CX3CL1	1119	17	19478	4.095673658203228	1	1	0.9813258636788048
IL13	GOTERM_BP_DIRECT	GO:0042770~signal transduction in response to DNA damage	4	0.3327787021	0.070257187	ATAD5, GADD45A, CHEK1, BID	1119	17	19478	4.095673658203228	1	1	0.9813258636788048
IL13	GOTERM_BP_DIRECT	GO:0031573~mitotic intra-S DNA damage checkpoint signaling	4	0.3327787021	0.070257187	TIPIN, MSH2, EME1, FANCD2	1119	17	19478	4.095673658203228	1	1	0.9813258636788048
IL13	GOTERM_BP_DIRECT	GO:0051290~protein heterotetramerization	4	0.3327787021	0.070257187	RRM1, RRM2, GPRIN1, FARSB	1119	17	19478	4.095673658203228	1	1	0.9813258636788048
IL13	GOTERM_BP_DIRECT	GO:0021819~layer formation in cerebral cortex	4	0.3327787021	0.070257187	ADGRG1, DAB2IP, LRP8, CDK5R1	1119	17	19478	4.095673658203228	1	1	0.9813258636788048
IL13	GOTERM_BP_DIRECT	GO:0051279~regulation of release of sequestered calcium ion into cytosol	4	0.3327787021	0.070257187	UBASH3B, ANK2, TMEM38B, CORO1A	1119	17	19478	4.095673658203228	1	1	0.9813258636788048
IL13	GOTERM_BP_DIRECT	GO:0042119~neutrophil activation	4	0.3327787021	0.070257187	CXCL6, IL18RAP, CXCL8, IL15	1119	17	19478	4.095673658203228	1	1	0.9813258636788048
IL13	GOTERM_BP_DIRECT	GO:0002486~antigen processing and presentation of endogenous peptide antigen via MHC class I via ER pathway, TAP-independent	4	0.3327787021	0.070257187	HLA-H, HLA-B, ULBP2, HLA-F	1119	17	19478	4.095673658203228	1	1	0.9813258636788048

IL13	GOTERM_BP_DIRECT	GO:0043388~positive regulation of DNA binding	4	0.3327787021	0.070257187	PLAUR, HES1, MMP9, NME1	1119	17	19478	4.095673658203228	1	1	0.9813258636788048
IL13	GOTERM_BP_DIRECT	GO:0002476~antigen processing and presentation of endogenous peptide antigen via MHC class Ib	4	0.3327787021	0.070257187	HLA-H, HLA-B, ULBP2, HLA-F	1119	17	19478	4.095673658203228	1	1	0.9813258636788048
IL13	GOTERM_BP_DIRECT	GO:0006855~xenobiotic transmembrane transport	4	0.3327787021	0.070257187	ABCC3, SLC29A1, SLC19A1, SLC28A3	1119	17	19478	4.095673658203228	1	1	0.9813258636788048
IL13	GOTERM_BP_DIRECT	GO:0051591~response to cAMP	6	0.4991680532	0.070681265	FOSL1, PNPT1, GATA6, MMP19, SDC1, BIRC2	1119	39	19478	2.6779404688251875	1	1	0.9813258636788048
IL13	KEGG_PATHWAY	hsa05165:Human papillomavirus infection	32	2.6622296173	0.071836908	PTGER4, PIK3R3, LAMC2, E2F1, HES1, ITGAV, IKBKE, HES4, HES7, IFNAR2, WNT10B, LAMB3, ITGA4, ITGA2, WNT7B, HLA-B, TUBG1, HLA-F, CCNA2, PKM, RBL1, CCNE2, CCNE1, TRAF3, COL4A4, IRF1, COL6A1, CDK2, FAS, ITGA6, COL6A6, ATR	607	333	8534	1.3510446195783923	0.9999999999792978	0.5339824567474918	0.4919111116704166
IL13	KEGG_PATHWAY	hsa04215:Apoptosis - multiple species	6	0.4991680532	0.07281579	PMAIP1, BIRC5, CYCS, BID, BIRC2, BIRC3	607	32	8534	2.636120263591433	0.9999999999853854	0.5339824567474918	0.4919111116704166
IL13	GOTERM_BP_DIRECT	GO:1905832~positive regulation of spindle assembly	3	0.2495840266	0.073177962	STIL, SPAG5, SASS6	1119	8	19478	6.527479892761394	1	1	0.9813258636788048
IL13	GOTERM_BP_DIRECT	GO:0090435~protein localization to nuclear envelope	3	0.2495840266	0.073177962	PLK1, LMNB2, LMNB1	1119	8	19478	6.527479892761394	1	1	0.9813258636788048
IL13	GOTERM_BP_DIRECT	GO:0016264~gap junction assembly	3	0.2495840266	0.073177962	GJC1, GJA1, GJD3	1119	8	19478	6.527479892761394	1	1	0.9813258636788048
IL13	GOTERM_BP_DIRECT	GO:0003129~heart induction	3	0.2495840266	0.073177962	BMP2, DKK1, ROBO1	1119	8	19478	6.527479892761394	1	1	0.9813258636788048
IL13	GOTERM_BP_DIRECT	GO:0044208~'de novo' AMP biosynthetic process	3	0.2495840266	0.073177962	PPAT, PAICS, PFAS	1119	8	19478	6.527479892761394	1	1	0.9813258636788048
IL13	GOTERM_BP_DIRECT	GO:0060767~epithelial cell proliferation involved in prostate gland development	3	0.2495840266	0.073177962	EAF2, NKX3-1, WDR77	1119	8	19478	6.527479892761394	1	1	0.9813258636788048
IL13	GOTERM_BP_DIRECT	GO:0036035~osteoclast development	3	0.2495840266	0.073177962	ANXA2, GPR68, TNFSF11	1119	8	19478	6.527479892761394	1	1	0.9813258636788048
IL13	GOTERM_BP_DIRECT	GO:0007077~mitotic nuclear membrane disassembly	3	0.2495840266	0.073177962	PLK1, CDK1, VRK1	1119	8	19478	6.527479892761394	1	1	0.9813258636788048
IL13	GOTERM_BP_DIRECT	GO:0045216~cell-cell junction organization	5	0.4159733777	0.073872739	GJA1, CXADR, HEG1, CLDN1, TJP2	1119	28	19478	3.1083237584578067	1	1	0.9813258636788048
IL13	UP_KW_BIOLOGICAL_PROCESS	KW-0145~Chemotaxis	12	0.9983361064	0.07404083	CXCL6, FLT1, CXCL8, ANOS1, CCL2, SLIT2, CXCL3, CXCL2, CXCL5, CX3CL1, TYMP, ROBO1	764	102	11523	1.7744071450569756	0.9999713918470443	0.44102581428185494	0.395957483

IL13	GOTERM_BP_DIRECT	GO:0007267~cell-cell signaling	20	1.6638935108	0.075799647	TNFSF18, SEMA5A, KLF10, CXCL6, PTGIR, IL15, ECE2, THY1, GDF5, CXCL5, CX3CL1, GJC1, PANX1, GJA1, ADGRG1, BMP2, GJD3, ADGRE5, NAMPT, FJX1	1119	232	19478	1.5005700902899755	1	1	0.9813258636788048
IL13	GOTERM_BP_DIRECT	GO:0048146~positive regulation of fibroblast proliferation	7	0.5823627287	0.076138806	CCNA2, CD74, CCNB1, SPHK1, LIF, E2F1, CDC6	1119	52	19478	2.343197910222039	1	1	0.9813258636788048
IL13	GOTERM_BP_DIRECT	GO:0050919~negative chemotaxis	6	0.4991680532	0.077145984	SEMA5A, SEMA7A, FLRT2, ITGAV, NRG1, SLIT2	1119	40	19478	2.610991957104558	1	1	0.9813258636788048
IL13	KEGG_PATHWAY	hsa05170:Human immunodeficiency virus 1 infection	22	1.8302828618	0.077922069	HLA-B, TAP2, TAP1, PIK3R3, ITPR2, TRAF2, CDC25C, TNFRSF1B, HLA-F, TAPBP, NFKBIA, CCNB2, CCNB1, CHEK1, CDK1, FAS, CYCS, AP1S3, BID, ATR, APOBEC3B, TLR2	607	213	8534	1.452135106078536	0.9999999999976378	0.559006145	0.5149632366268523
IL13	GOTERM_BP_DIRECT	GO:0032757~positive regulation of interleukin-8 production	8	0.6655574043	0.078172628	TLR1, CD74, IL6, NLRP10, SERPINE1, CHI3L1, CD58, TLR2	1119	65	19478	2.142352375	1	1	0.9813258636788048
IL13	GOTERM_BP_DIRECT	GO:0045766~positive regulation of angiogenesis	15	1.2479201331	0.079608728	SEMA5A, FLT1, CXCL8, SPHK1, ERAP1, SERPINE1, GATA6, VASH2, VEGFC, BRCA1, ETS1, CX3CL1, C3, ZC3H12A, CHI3L1	1119	161	19478	1.6217341348475514	1	1	0.9813258636788048
IL13	BIOCARTA	h_p53Pathway:p53 Signaling Pathway	5	0.4159733777	0.079812744	PCNA, CCNE1, GADD45A, CDK2, E2F1	162	17	1622	2.9448075526506896	0.9999999235083149	0.9009008713808473	0.8871115723290995
IL13	GOTERM_BP_DIRECT	GO:0010569~regulation of double-strand break repair via homologous recombination	4	0.3327787021	0.080847719	RAD51AP1, RAD51, FIGNL1, CHEK1	1119	18	19478	3.868136232747493	1	1	0.9813258636788048
IL13	GOTERM_BP_DIRECT	GO:0060384~innervation	4	0.3327787021	0.080847719	VCAM1, CHD7, LRIG1, SULF1	1119	18	19478	3.868136232747493	1	1	0.9813258636788048
IL13	GOTERM_BP_DIRECT	GO:0007140~male meiotic nuclear division	5	0.4159733777	0.081977977	KIF18A, CYP26B1, FIGNL1, FANCA, TESMIN	1119	29	19478	3.001140180579951	1	1	0.9813258636788048
IL13	GOTERM_BP_DIRECT	GO:0048714~positive regulation of oligodendrocyte differentiation	5	0.4159733777	0.081977977	IL33, IL34, TNFRSF1B, TLR2, TP73	1119	29	19478	3.001140180579951	1	1	0.9813258636788048
IL13	GOTERM_BP_DIRECT	GO:0051216~cartilage development	8	0.6655574043	0.083346525	ERRFI1, BMP2, COL11A2, NOG, COL6A1, CHI3L1, TYMS, SULF1	1119	66	19478	2.1098924905895413	1	1	0.9813258636788048
IL13	GOTERM_BP_DIRECT	GO:0030199~collagen fibril organization	8	0.6655574043	0.083346525	ADAMTS3, COL27A1, ANXA2, COL5A3, COL11A2, COL6A1, LOXL4, PLOD2	1119	66	19478	2.1098924905895413	1	1	0.9813258636788048
IL13	GOTERM_BP_DIRECT	GO:0051056~regulation of small GTPase mediated signal transduction	12	0.9983361064	0.083886485	ARHGAP11A, NET1, ARHGAP22, ARHGAP11B, RACGAP1, ARHGEF28, PLEKHG6, ARHGAP18, DOCK2, ECT2, SIPA1L3, ARHGAP45	1119	120	19478	1.7406613047363717	1	1	0.9813258636788048
IL13	GOTERM_BP_DIRECT	GO:0048144~fibroblast proliferation	6	0.4991680532	0.083917712	WNT7B, CDK1, LIF, CKS2, E2F8, CKS1B	1119	41	19478	2.547309226443471	1	1	0.9813258636788048
IL13	GOTERM_BP_DIRECT	GO:0008285~negative regulation of cell population proliferation	33	2.7454242928	0.086398085	CXCL8, SPIN4, WWC1, CXCL1, ETS1, DLL4, ADGRG1, BRIP1, NUP62, TNFRSF8, UTP20, PIM2, SH2B3, E2F7, NKX3-1, KLF10, TNFRSF9, DAB2IP, LIF, HMGA1, P3H2, CDC6, SSTR2, SOD2, GTPBP4, NME1, FOSL1, BMP2, IL6, IRF1, CDKN3, TLR2, TP73	1119	433	19478	1.3266009943718307	1	1	0.9813258636788048
IL13	KEGG_PATHWAY	hsa05215:Prostate cancer	12	0.9983361064	0.086598706	NFKBIA, HSP90AA1, CCNE2, PLAU, CCNE1, CDK2, E2F1, PIK3R3, E2F2, E2F3, MMP9, NKX3-1	607	98	8534	1.721547927243385	0.9999999999998956	0.6080334672923837	0.5601278001723777

IL13	BIOCARTA	h_btg2Pathway:BTG family proteins and cell cycle regulation	4	0.3327787021	0.087332227	CHAF1B, HOXB9, CHAF1A, PRMT1	162	11	1622	3.6408529741863074	0.9999999848083506	0.9009008713808473	0.8871115723290995
IL13	BIOCARTA	h_ranMSPathway:Role of Ran in mitotic spindle regulation	4	0.3327787021	0.087332227	TPX2, KPNA2, KIF15, AURKA	162	11	1622	3.6408529741863074	0.9999999848083506	0.9009008713808473	0.8871115723290995
IL13	BIOCARTA	h_mhcPathway:Antigen Processing and Presentation	4	0.3327787021	0.087332227	CD74, TAP2, TAP1, PSMB9	162	11	1622	3.6408529741863074	0.9999999848083506	0.9009008713808473	0.8871115723290995
IL13	BIOCARTA	h_ptc1Pathway:Sonic Hedgehog (SHH) Receptor Ptc1 Regulates cell cycle	4	0.3327787021	0.087332227	CCNB1, CDK1, CDC25C, CDC25A	162	11	1622	3.6408529741863074	0.9999999848083506	0.9009008713808473	0.8871115723290995
IL13	GOTERM_BP_DIRECT	GO:0001822~kidney development	12	0.9983361064	0.087753583	HELLS, CENPF, CYP26B1, HPGD, SIX2, C1GALT1, PCSK9, HAS2, PRDM1, SULF1, ADAMTS6, TP73	1119	121	19478	1.726275674118716	1	1	0.9813258636788048
IL13	GOTERM_BP_DIRECT	GO:0042752~regulation of circadian rhythm	8	0.6655574043	0.088712084	TOP2A, KLF10, NOCT, BHLHE40, TIMELESS, CDK1, ADCY1, EZH2	1119	67	19478	2.0784015578941752	1	1	0.9813258636788048
IL13	GOTERM_BP_DIRECT	GO:0010467~gene expression	17	1.4143094841	0.089401649	IL33, ERRFI1, EGR2, DOT1L, GATA6, LIF, FMN1, LPAR3, PRDM1, IQGAP3, HS2ST1, FOSL1, GRM2, EDNRB, DIAPH3, COL6A1, IKBKE	1119	193	19478	1.533224983446545	1	1	0.9813258636788048
IL13	GOTERM_BP_DIRECT	GO:0032880~regulation of protein localization	10	0.8319467554	0.090227446	HSP90AA1, TRIM6, BORA, BMPER, PARP1, CCNE2, CCNE1, C9ORF72, NKX3-1, PICALM	1119	94	19478	1.8517673454642252	1	1	0.9813258636788048
IL13	GOTERM_BP_DIRECT	GO:0002639~positive regulation of immunoglobulin production	5	0.4159733777	0.090500615	IL33, IL6, TNFSF4, DNAJB9, TNFRSF4	1119	30	19478	2.901102174560619	1	1	0.9813258636788048
IL13	GOTERM_BP_DIRECT	GO:1903753~negative regulation of p38MAPK cascade	3	0.2495840266	0.09060006	CYLD, DUSP10, EZR	1119	9	19478	5.802204349	1	1	0.9813258636788048
IL13	GOTERM_BP_DIRECT	GO:0060426~lung vasculature development	3	0.2495840266	0.09060006	ERRFI1, BMP2, LIF	1119	9	19478	5.802204349	1	1	0.9813258636788048
IL13	GOTERM_BP_DIRECT	GO:0046653~tetrahydrofolate metabolic process	3	0.2495840266	0.09060006	DHFR, DHFRP1, SHMT1	1119	9	19478	5.802204349	1	1	0.9813258636788048
IL13	GOTERM_BP_DIRECT	GO:0072006~nephron development	3	0.2495840266	0.09060006	NUP107, NUP85, SIX2	1119	9	19478	5.802204349	1	1	0.9813258636788048
IL13	GOTERM_BP_DIRECT	GO:0032825~positive regulation of natural killer cell differentiation	3	0.2495840266	0.09060006	IL15RA, IL15, RASGRP1	1119	9	19478	5.802204349	1	1	0.9813258636788048
IL13	GOTERM_BP_DIRECT	GO:0106300~protein-DNA covalent cross-linking repair	3	0.2495840266	0.09060006	FAM111A, BRIP1, TRAIP	1119	9	19478	5.802204349	1	1	0.9813258636788048
IL13	GOTERM_BP_DIRECT	GO:0034214~protein hexamerization	3	0.2495840266	0.09060006	MAT2A, LRRC8C, LRRC8D	1119	9	19478	5.802204349	1	1	0.9813258636788048

IL13	GOTERM_BP_DIRECT	GO:0070100~negative regulation of chemokine-mediated signaling pathway	3	0.2495840266	0.09060006	SLIT2, SH2B3, ROBO1	1119	9	19478	5.802204349	1	1	0.9813258636788048
IL13	GOTERM_BP_DIRECT	GO:0043504~mitochondrial DNA repair	3	0.2495840266	0.09060006	MGME1, PARP1, DNA2	1119	9	19478	5.802204349	1	1	0.9813258636788048
IL13	GOTERM_BP_DIRECT	GO:0060544~regulation of necroptotic process	3	0.2495840266	0.09060006	CYLD, BIRC2, BIRC3	1119	9	19478	5.802204349	1	1	0.9813258636788048
IL13	GOTERM_BP_DIRECT	GO:1904894~positive regulation of receptor signaling pathway via STAT	3	0.2495840266	0.09060006	IL6, TSLP, LIF	1119	9	19478	5.802204349	1	1	0.9813258636788048
IL13	GOTERM_BP_DIRECT	GO:0032873~negative regulation of stress-activated MAPK cascade	3	0.2495840266	0.09060006	DUSP10, PBK, FOXM1	1119	9	19478	5.802204349	1	1	0.9813258636788048
IL13	GOTERM_BP_DIRECT	GO:0032733~positive regulation of interleukin-10 production	6	0.4991680532	0.090991837	CD274, IL6, TSLP, TNFSF4, HSPD1, TLR2	1119	42	19478	2.4866590067662457	1	1	0.9813258636788048
IL13	GOTERM_BP_DIRECT	GO:0050678~regulation of epithelial cell proliferation	4	0.3327787021	0.092079411	EDNRB, UHRF1, HES1, BID	1119	19	19478	3.664550115234467	1	1	0.9813258636788048
IL13	GOTERM_BP_DIRECT	GO:0032147~activation of protein kinase activity	4	0.3327787021	0.092079411	TPX2, KIF14, CLSPN, ECT2	1119	19	19478	3.664550115234467	1	1	0.9813258636788048
IL13	GOTERM_BP_DIRECT	GO:0051930~regulation of sensory perception of pain	4	0.3327787021	0.092079411	ZFHX2, FABP5, SPX, MGLL	1119	19	19478	3.664550115234467	1	1	0.9813258636788048
IL13	UP_KW_BIOLOGICAL_PROCESS	KW-0545~Nucleotide biosynthesis	3	0.2495840266	0.093955662	PRPS2, DTYMK, TYMS	764	8	11523	5.655922775	0.9999985129509712	0.5363302384153809	0.48152276879629086
IL13	GOTERM_BP_DIRECT	GO:0002931~response to ischemia	8	0.6655574043	0.094268096	PANX1, TIGAR, CSF1, MYB, PPIF, HYOU1, CX3CL1, MAP3K5	1119	68	19478	2.047836829101614	1	1	0.9813258636788048
IL13	GOTERM_BP_DIRECT	GO:0009887~animal organ morphogenesis	10	0.8319467554	0.094882993	ETV7, BMP2, CDX1, ITGA2, GMNN, VEGFC, CCL2, PHLDA2, PALB2, FLI1	1119	95	19478	1.8322750576172333	1	1	0.9813258636788048
IL13	GOTERM_BP_DIRECT	GO:0060391~positive regulation of SMAD protein signal transduction	6	0.4991680532	0.098362865	BMP2, BMPER, PARP1, TTK, GDF6, GDF5	1119	43	19478	2.428829727539123	1	1	0.9813258636788048
IL13	GOTERM_BP_DIRECT	GO:0032728~positive regulation of interferon-beta production	6	0.4991680532	0.098362865	IFIH1, HSP90AA1, IRF1, OAS3, POLR3G, TLR2	1119	43	19478	2.428829727539123	1	1	0.9813258636788048
IL13	KEGG_PATHWAY	hsa05160:Hepatitis C	17	1.4143094841	0.098806293	IFNAR2, PIK3R3, TRAF2, CLDN1, NFKBIA, CLDN4, TRAF3, OAS3, CDK2, E2F1, FAS, E2F2, CYCS, E2F3, CLDN16, BID, IKBKE	607	159	8534	1.503196461	0.9999999999999988	0.6792932666991224	0.6257731911410096
IL13	GOTERM_BP_DIRECT	GO:0034113~heterotypic cell-cell adhesion	5	0.4159733777	0.099427785	VCAM1, ITGA4, ITGAV, CD58, THY1	1119	31	19478	2.807518233445761	1	1	0.9813258636788048

IL13	UP_KW_BIOLOGICAL_PROCESS	KW-0509~mRNA transport	13	1.0815307820	0.099497175	NDC1, NUP205, NUP107, SEH1L, NUP188, NUP155, NUP153, NUP85, NUP50, NUP62, NUP35, NUP88, SRSF7	764	120	11523	1.6339332460732987	0.9999993543891749	0.5452445208130295	0.48952610262775637
IL13	GOTERM_BP_DIRECT	GO:0098586~cellular response to virus	10	0.8319467554	0.099675776	EIF5A, IFIH1, EIF5AL1, IFNAR2, IL6, HSP90AA1, TRIM6, ZC3H12A, POU2F2, IKBKE	1119	96	19478	1.813188859100387	1	1	0.9813258636788048
IL17A	UP_KW_BIOLOGICAL_PROCESS	KW-0131~Cell cycle	148	11.229135053	7.1867717456	ERCC6L, ANKLE2, ZWILCH, DSCC1, GMNN, CCNF, HJURP, BUB1B, AHR, MKI67, SMC4, CDC14A, SMC2, CDC20, MTBP, PTTG1, RPS6KA1, CHEK1, NUSAP1, KNTC1, OIP5, PIM3, NEK2, FBXO5, LZTS1, TP63, TIPIN, LIG1, VRK1, ESCO2, KNL1, ATAD3B, HASPIN, PIMREG, CDC25A, SGO1, SGO2, MELK, CCNE2, FANCD2, CCNE1, TIMELESS, KIF20B, SEH1L, CDCA2, CDCA3, HEPACAM, CDCA5, CDCA8, NCAPG, PKMYT1, SKA3, NCAPH, SKA1, NPAT, DSN1, CCNB1, RACGAP1, CLSPN, ECT2, FAM83D, FANCI, CDT1, UBE2C, GADD45A, DDIAS, RCC2, CDC7, CDC6, ZWINT, NDC80, CYLD, ANLN, TPX2, KIF18B, CDK6, NASP, CDK2, SASS6, CDK1, SUV39H2, USP37, MCM7, MCM8, SUV39H1, DDX12P, NCAPG2, BRCA1, KIF11, FOXM1, BRCA2, CHTF18, CKS1B, CHAF1B, CHAF1A, NUF2, SPDL1, DLGAP5, CEP55, HELLS, CKAP2, HAUS6, KIF23, SMC1A, MASTL, CCNA2, ASPM, RBL1, DBF4, INCENP, KIFC1, DMC1, MCM3, CKS2, BIRC5, MCM4, MCM5, KIF2C, MCM6, MCM2, UHRF1, NEDD9,	824	690	11523	2.9995145631067963	1.006148044397278E-33	1.0133348161429728E-33	9.342803269403296E-34
IL17A	GOTERM_BP_DIRECT	GO:0006260~DNA replication	49	3.7177541729	1.6668157885	FEN1, MCM7, DSCC1, CHTF18, CHAF1B, CHAF1A, CHEK1, PCLAF, POLE, RFC5, SUPT16H, RFC3, RFC4, LIG1, RFC2, TWNK, RECQL, DNAJC2, DBF4, GRWD1, MCM4, MCM5, MCM6, DNA2, DTL, MCM2, BLM, RNASEH2A, PCNA, RECQL4, POLD3, ORC6, ORC1, POLD1, GINS1, GINS3, DONSON, TICRR, FAM111B, FAM111A, POLA1, POLA2, NASP, NFIB, POLE2, POLE3, CDK2, CDK1, ATR	1218	117	19478	6.697416249140387	7.732358442932183E-24	7.732358442932183E-24	7.609014074581897E-24
IL17A	UP_KW_BIOLOGICAL_PROCESS	KW-0235~DNA replication	46	3.4901365705	7.1369792232	BLM, FEN1, PCNA, MCM7, MCM8, DSCC1, PRIM1, MCM10, CHTF18, POLD3, CHAF1B, ORC6, CHAF1A, CDC45, ORC1, POLD1, POLE, RFC5, GINS1, GINS2, CDT1, RFC3, SUPT16H, RFC4, LIG1, RFC2, GINS3, DDX11, GINS4, TWNK, CDC6, FAM111A, POLA1, POLA2, DBF4, NASP, NFIB, GRWD1, POLE2, MCM3, MCM4, MCM5, MCM6, DNA2, DTL, MCM2	824	95	11523	6.771308124680634	9.991770912526525E-25	5.031570352379429E-25	4.6390364951016015E-25
IL17A	UP_KW_BIOLOGICAL_PROCESS	KW-0498~Mitosis	79	5.9939301972	1.9469929226	ERCC6L, USP37, ANKLE2, ZWILCH, CCNF, NCAPG2, BUB1B, KIF11, SMC4, SMC2, CDC20, PTTG1, NUF2, NUSAP1, KNTC1, NEK2, OIP5, FBXO5, SPDL1, CEP55, HELLS, TIPIN, HAUS6, VRK1, KIF23, KNL1, SMC1A, MASTL, PIMREG, CDC25A, SGO1, CCNA2, ASPM, MELK, INCENP, KIFC1, TIMELESS, BIRC5, KIF2C, KIF20B, CDCA2, SEH1L, CDCA3, CDCA5, NEDD9, NCAPG, CDCA8, SKA3, NCAPH, SKA1, AURKB, DSN1, CCNB1, HAUS8, RBBP8, BUB3, FAM83D, BUB1, CDT1, CENPW, CENPX, SPAG5, UBE2C, RCC2, CDC6, NDC80, ZWINT, CENPE, ANLN, TPX2, CENPF, KIF18B, CDK2, CDK1, TACC3, NCAPD3, SPC24, SPC25, MAD2L1	824	294	11523	3.7576654448187043	2.72579009166582E-23	9.15086673630668E-24	8.436969331346585E-24

IL17A	GOTERM_BP_DIRECT	GO:0051301~cell division	87	6.6009104704	6.1153947564	ERCC6L, USP37, ANKLE2, ZWILCH, CCNF, NCAPG2, KIF14, BUB1B, KIF11, SMC4, CDC14A, CKS1B, SMC2, CDC20, TUBA1C, PTTG1, NUF2, KNTC1, NEK2, OIP5, FBXO5, SPDL1, HELLS, TIPIN, LIG1, HAUS6, VRK1, KNL1, ATAD3B, SMC1A, MASTL, PIMREG, CDC25A, SGO1, CCNA2, ASPM, SGO2, CCNE2, CCNE1, KIFC1, CKS2, TIMELESS, BIRC5, MCM5, KIF2C, KIF20B, CDCA2, SEH1L, CDCA3, CDCA5, NEDD9, NCAPG, CDCA8, SKA3, NCAPH, SKA1, AURKB, DSN1, CCNB1, HAUS8, RBBP8, BUB3, FAM83D, BUB1, CDT1, CENPW, CENPX, SPAG5, UBE2C, RCC2, CDC7, CDC6, NDC80, ZWINT, CENPE, TPX2, CENPF, KIF18B, CDK6, PRC1, CDK2, CDK1, TACC3, NCAPD3, SPC24, SPC25, MAD2L1	1218	397	19478	3.5044980208708165	2.836931627508573E-21	1.4184658137542865E-21	1.39583885315549E-21
IL17A	UP_KW_BIOLOGICAL_PROCESS	KW-0132~Cell division	93	7.0561456752	3.1542925455	ERCC6L, USP37, ANKLE2, ZWILCH, CCNF, NCAPG2, BUB1B, KIF11, SMC4, CDC14A, CKS1B, SMC2, CDC20, PTTG1, NUF2, NUSAP1, KNTC1, NEK2, OIP5, FBXO5, SPDL1, CEP55, HELLS, TIPIN, LIG1, HAUS6, VRK1, KIF23, KNL1, ATAD3B, SMC1A, MASTL, PIMREG, CDC25A, SGO1, CCNA2, ASPM, SGO2, MELK, CCNE2, CCNE1, INCENP, KIFC1, CKS2, TIMELESS, BIRC5, MCM5, KIF2C, KIF20B, CDCA2, SEH1L, CDCA3, CDCA5, NEDD9, NCAPG, CDCA8, SKA3, NCAPH, SKA1, AURKB, DSN1, CCNB1, HAUS8, RACGAP1, RBBP8, BUB3, FAM83D, ECT2, BUB1, CDT1, CENPW, CENPX, SPAG5, UBE2C, RCC2, CDC7, CDC6, NDC80, ZWINT, CENPE, ANLN, TPX2, CENPF, KIF18B, CDK6, PRC1, CDK2, CDK1, TACC3, NCAPD3, SPC24, SPC25, MAD2L1	824	421	11523	3.089151465535134	4.416009563719335E-21	1.1118881222936182E-21	1.0251450772919883E-21
IL17A	GOTERM_BP_DIRECT	GO:0007059~chromosome segregation	38	2.8831562974	1.6077771597	TOP2A, CDCA2, HJURP, TTK, BRCA1, MKI67, SKA3, SKA1, DSN1, NUF2, OIP5, NEK2, BUB1, DLGAP5, CENPU, CENPW, CENPX, SPAG5, ESCO2, NDC80, SRPK1, SGO1, SGO2, CENPE, CENPF, DIAPH3, INCENP, CENPI, CENPK, CENPL, BIRC5, CENPM, CENPN, KIF2C, CENPO, CEP85, SPC24, SPC25	1218	101	19478	6.016713001349396	7.45847824388306E-16	2.4861594146276863E-16	2.446500911354902E-16
IL17A	KEGG_PATHWAY	hsa04110:Cell cycle	52	3.9453717754	2.0073878551	MCM7, BUB1B, CDC14A, CDC20, MTBP, PTTG1, CHEK1, FBXO5, DDX11, ESCO2, KNL1, SMC1A, CDC25A, SGO1, CCNA2, RBL1, DBF4, CCNE2, ESPL1, CCNE1, MCM3, MCM4, MCM5, MCM6, MCM2, PCNA, CDCA5, TTK, PKMYT1, AURKB, CCNB1, ORC6, CDC45, ORC1, E2F1, E2F2, E2F3, BUB3, E2F5, BUB1, CDT1, GADD45A, CDC7, CDC6, NDC80, TICRR, CDK6, CDK2, CDK1, TRIP13, ATR, MAD2L1	671	158	8534	4.185779773246052	6.784970950565702E-17	6.784970950565702E-17	6.283123986766464E-17
IL17A	GOTERM_BP_DIRECT	GO:0006281~DNA repair	65	4.9317147192	2.7421605822	DCLRE1B, ERCC6L, FEN1, BRCA1, FOXM1, BRCA2, CHAF1B, CHAF1A, EME1, PTTG1, EXO1, CHEK1, PCLAF, TOPBP1, POLE, RFC5, WDHD1, SUPT16H, RFC3, RFC4, LIG1, PARP1, PARP2, RFC2, DDX11, RECQL, SMC1A, MSH2, FANCD2, DMC1, TIMELESS, DNA2, INO80C, BLM, PARPBP, DOT1L, RAD51AP1, RECQL4, BRIP1, POLD1, RBBP8, USP1, RAD54L, CLSPN, FANCI, BARD1, POLQ, RRM1, CENPX, FANCM, GADD45A, XRCC2, XRCC3, FANCA, FANCG, TICRR, POLA1, RAD51, UBE2T, POLE2, TDP1, CDK2, CDK1, RAD18, ATR	1218	303	19478	3.430581974453603	1.2720882940913528E-14	3.180220735228382E-15	3.1294907644573325E-15

IL17A	KEGG_PATHWAY	hsa03030:DNA replication	23	1.7450682852	4.4282744806	RFC5, FEN1, RFC3, RFC4, RNASEH2A, PCNA, LIG1, MCM7, RFC2, PRIM1, POLD3, POLA1, POLA2, POLD1, POLE2, POLE3, MCM3, MCM4, MCM5, DNA2, MCM6, POLE, MCM2	671	36	8534	8.125600264944527	1.5010215292932116E-13	7.483783872330087E-14	6.9302495622465E-14
IL17A	UP_KW_BIOLOGICAL_PROCESS	KW-0227~DNA damage	81	6.1456752655	4.5847419399	DCLRE1B, FEN1, ANKLE1, MCM8, WDR4, TONSL, MCM10, BRCA1, FOXM1, BRCA2, CHAF1B, CHAF1A, EME1, PTTG1, EXO1, ZC3H12A, CHEK1, PCLAF, TOPBP1, IKBKE, POLE, GEN1, TIPIN, SUPT16H, LIG1, PARP1, ZGRF1, PARP2, DDX11, AEN, SMC1A, MMS22L, NEIL3, SLFN11, MSH2, FANCD2, TIMELESS, DNA2, DTL, INO80C, BLM, PARPBP, PCNA, PSMD14, UHRF1, NPAS2, RAD54B, UNG, RAD51AP1, POLD3, BRIP1, POLD1, RBBP8, USP1, RAD54L, CLSPN, E2F7, FANCI, BARD1, POLQ, EGLN3, SLF1, CENPX, ATAD5, FANCM, GADD45A, XRCC2, XRCC3, FANCA, PALB2, FANCG, TICRR, FAM111A, RAD51, UBE2T, TDP1, RFWD3, CDK2, AUNIP, RAD18, ATR	824	443	11523	2.556934734489031	6.372680161348399E-13	1.2928972270688986E-13	1.1920329043897647E-13
IL17A	GOTERM_BP_DIRECT	GO:0006270~DNA replication initiation	18	1.3657056145	1.4095927197	MCM7, PRIM1, MCM10, NOC3L, CDC6, POLA1, POLA2, ORC6, CDC45, ORC1, CCNE2, CCNE1, MCM3, MCM4, MCM5, TOPBP1, MCM6, MCM2	1218	27	19478	10.66119321291735	6.540912256269849E-11	1.3078201253864495E-11	1.2869581531341113E-11
IL17A	GOTERM_BP_DIRECT	GO:0007094~mitotic spindle assembly checkpoint signaling	18	1.3657056145	1.6667677638	ZWILCH, BUB1B, TTK, HASPIN, NDC80, ZWINT, CDC20, CENPF, NUF2, BIRC5, KNTC1, BUB3, TRIP13, SPDL1, BUB1, SPC24, MAD2L1, SPC25	1218	30	19478	9.595073891625615	7.730637241465388E-10	1.2886892760786797E-10	1.268132473657938E-10
IL17A	GOTERM_BP_DIRECT	GO:0000070~mitotic sister chromatid segregation	19	1.4415781487	3.0567712447	SPAG5, NCAPG2, CDCA8, KNL1, SMC1A, SKA3, SMC4, NDC80, SKA1, ZWINT, KIF18A, KIF18B, ESPL1, KIFC1, CENPI, CENPK, NUSAP1, KNTC1, MAD2L1	1218	35	19478	8.681257330518413	1.4178843654732987E-9	2.02576597204342E-10	1.9934515331705567E-10
IL17A	UP_KW_BIOLOGICAL_PROCESS	KW-0234~DNA repair	68	5.1593323216	4.6259978222	DCLRE1B, FEN1, ANKLE1, MCM8, TONSL, BRCA1, FOXM1, BRCA2, CHAF1B, CHAF1A, EME1, PTTG1, EXO1, CHEK1, PCLAF, TOPBP1, POLE, GEN1, SUPT16H, LIG1, PARP1, ZGRF1, PARP2, DDX11, SMC1A, MMS22L, NEIL3, MSH2, FANCD2, TIMELESS, DNA2, INO80C, BLM, PARPBP, PCNA, PSMD14, UHRF1, RAD54B, UNG, RAD51AP1, POLD3, BRIP1, POLD1, RBBP8, USP1, RAD54L, CLSPN, FANCI, BARD1, POLQ, SLF1, CENPX, FANCM, XRCC2, XRCC3, FANCA, PALB2, FANCG, TICRR, FAM111A, RAD51, UBE2T, TDP1, RFWD3, CDK2, AUNIP, RAD18, ATR	824	366	11523	2.598161706	6.476819081058238E-11	1.0871094882403808E-11	1.0022995281648901E-11
IL17A	KEGG_PATHWAY	hsa04668:TNF signaling pathway	37	2.8072837632	4.9081795246	CXCL6, CSF2, CSF1, TNFAIP3, PIK3R3, CXCL1, CXCL3, CXCL2, CXCL5, CX3CL1, ICAM1, CCL2, JUNB, MAP3K5, VCAM1, MLKL, IL15, RHBDF2, LIF, VEGFC, TRAF2, TRAF1, TNFRSF1B, MMP9, NFKB1, NFKBIA, CYLD, IL6, TRAF3, IRF1, BCL3, FAS, PGAM5, MAP3K14, BIRC2, IL18R1, BIRC3	671	119	8534	3.9544390036193318	1.6590040452513222E-10	5.5298822644074895E-11	5.12086730402232E-11
IL17A	GOTERM_BP_DIRECT	GO:0031297~replication fork processing	18	1.3657056145	2.4814454126	GEN1, POLQ, BLM, TIPIN, PCNA, FANCM, CENPX, DDX11, RECQL, TONSL, DONSON, MMS22L, FAM111A, RAD51, EME1, RFWD3, TIMELESS, ATR	1218	38	19478	7.575058335493907	1.1511438369193883E-7	1.4389281586702416E-8	1.4159747886030724E-8

IL17A	GOTERM_BP_DIRECT	GO:0006974~DNA damage response	53	4.0212443095	8.8317506148	TOP2A, ANKLE1, MCM7, MCM8, SUV39H1, WDR4, MCM10, BRCA1, ZC3H12A, CHEK1, PCLAF, FBXO5, TOPBP1, TP63, NKX3-1, TIPIN, PARP1, PARP2, PRMT1, DDX11, VRK1, MASTL, ATAD3A, WDR76, SLFN11, TIMELESS, DTL, BLM, UHRF1, FBXO45, NPAS2, RAD51AP1, BRIP1, PMAIP1, BARD1, LYN, POLQ, EGLN3, TIGAR, SLF1, GADD45A, XRCC3, FANCG, FAM111A, RAD51, UBE2T, RFWD3, BCL3, POLE3, BCL2, CDK1, RAD18, ATR	1218	313	19478	2.7078749534406694	4.097046336637433E-7	4.465332494145748E-8	4.394102788483583E-8
IL17A	GOTERM_BP_DIRECT	GO:0006954~inflammatory response	65	4.9317147192	9.6256359002	CXCL6, NRROS, CXCL8, CSF1, GPR68, TNFAIP3, CXCL1, CXCL3, CXCL2, CXCL5, CX3CL1, ADGRE2, CYP26B1, IL18RAP, ADGRE5, ZC3H12A, NAMPT, OLR1, BDKRB1, TNFRSF4, PTGIR, SPHK1, NFAM1, PLA2G4C, IL18, TNFRSF1B, TLR1, IL1A, ELF3, CHI3L1, TLR10, ADAM8, CD47, TLR6, TLR3, MGLL, CHST2, EPHA2, TLR2, PTGER4, SEMA7A, HDAC9, FUT4, RELB, C3, IL1RL1, IRAK2, CCL2, APOL3, GGT5, VCAM1, IL34, NFKB1, NFKB2, FOSL1, MFHAS1, BMP2, IL6, VNN1, NLRP10, MIR221, TNFSF4, PTX3, IL18R1, NFKBIB	1218	432	19478	2.4061720793042634	4.4653304409614236E-7	4.465332494145748E-8	4.394102788483583E-8
IL17A	GOTERM_BP_DIRECT	GO:0000727~double-strand break repair via break-induced replication	10	0.7587253414	2.6136074344	GINS2, CDC45, MCM7, GINS4, MCM3, MCM4, CDC7, MCM5, MCM6, MCM2	1218	12	19478	13.326491516146689	1.212445143561336E-5	1.1022295353143323E-6	1.0846470853006956E-6
IL17A	GOTERM_BP_DIRECT	GO:0000278~mitotic cell cycle	30	2.2761760242	3.6868094899	CDCA5, NOLC1, CDCA8, KIF11, PKMYT1, SKA3, SKA1, AURKB, KIF15, TUBA1C, MYB, PBK, NEK2, MYBL2, NUDT15, POLE, MYBL1, CDT1, WDHD1, CENPW, HGF, XRCC2, HASPIN, NDC80, CENPE, TPX2, CENPF, KIF18B, INCENP, BIRC5	1218	135	19478	3.5537310709724506	1.7102963157600293E-5	1.4252591019717125E-6	1.4025237767839766E-6
IL17A	KEGG_PATHWAY	hsa05222:Small cell lung cancer	27	2.048558422	5.8261159847	PIK3R3, LAMC2, CKS1B, E2F1, E2F2, E2F3, ITGAV, LAMB3, GADD45A, ITGA2, TRAF2, TRAF1, NFKB1, NFKBIA, CDK6, TRAF4, CCNE2, CCNE1, TRAF3, COL4A4, CDK2, CKS2, BCL2, CYCS, ITGA6, BIRC2, BIRC3	671	93	8534	3.692418633719533	1.969225286280185E-6	4.923068007104267E-7	4.5589357580580927E-7
IL17A	BIOCARTA	h_mcmPathway:CDK Regulation of DNA Replication	13	0.9863429438	3.4790442693	CDT1, MCM7, CDC6, KITLG, ORC6, ORC1, CCNE1, CDK2, MCM3, MCM4, MCM5, MCM6, MCM2	186	18	1622	6.298088410991636	7.236386034836784E-6	7.201621637638587E-6	7.132040752250774E-6
IL17A	UP_KW_BIOLOGICAL_PROCESS	KW-0690~Ribosome biogenesis	26	1.9726858877	5.0342920589	LTV1, PAK1IP1, NIP7, NOP2, IPO4, WDR43, RRS1, HEATR3, RIOK1, DCAF13, NOP56, UTP15, NOP58, NOP14, UTP4, IMP4, GNL2, WDR12, GTPBP4, RCL1, BOP1, EBNA1BP2, MYBBP1A, DKC1, MRTO4, TSR1	824	104	11523	3.4960558252427183	7.0479842256832015E-6	1.0140502575921114E-6	9.349399538083297E-7
IL17A	KEGG_PATHWAY	hsa03410:Base excision repair	17	1.2898330804	9.0475394063	RFC5, FEN1, RFC3, RFC4, PCNA, LIG1, PARP1, PARP2, RFC2, UNG, POLD3, NEIL3, POLD1, POLE2, TDP1, POLE3, POLE	671	44	8534	4.9139005554802875	3.058021699320346E-5	6.116136638661072E-6	5.663759668345905E-6
IL17A	GOTERM_BP_DIRECT	GO:0000724~double-strand break repair via homologous recombination	24	1.8209408194	1.0222844050	GEN1, BLM, SLF1, FEN1, ANKLE1, PSMD14, MCM8, UHRF1, XRCC2, XRCC3, RECQL, TONSL, BRCA1, BRCA2, PALB2, RAD54B, RAD51AP1, RECQL4, MMS22L, RAD51, RFWD3, RBBP8, AUNIP, TOPBP1	1218	105	19478	3.655266244428806	4.7412532672375107E-4	3.647982580749049E-5	3.589791006923778E-5
IL17A	KEGG_PATHWAY	hsa05169:Epstein-Barr virus infection	40	3.0349013657	1.3102639466	PSMD14, TNFAIP3, PIK3R3, RELB, ICAM1, E2F1, E2F2, E2F3, HES1, CD58, BID, JAK3, IKBKE, LYN, IFNAR2, GADD45A, HLA-B, TAP2, TAP1, TRAF2, HLA-G, NFKB1, TAPBP, NFKB2, NFKBIA, CCNA2, IL6, CDK6, CCNE2, CCNE1, TRAF3, OAS3, CDK2, BCL2, FAS, CYCS, NFKBIE, MAP3K14, NFKBIB, TLR2	671	204	8534	2.4937903626428217	4.428594363259375E-5	7.381153565885405E-6	6.835210254799206E-6

IL17A	KEGG_PATHWAY	hsa04060:Cytokine-cytokine receptor interaction	51	3.8694992412	1.7608523565	CXCL6, BMP10, CNTF, CSF2, CXCL8, CSF1, CXCL1, CXCL3, CXCL2, CXCL5, CX3CL1, TNFSF13B, IL27RA, IL18RAP, TNFSF10, TNFSF11, TNFRSF8, IL12A, TNFRSF4, IFNAR2, IL15RA, IL4R, IL15, IL18, LIFR, OSMR, TNFRSF1B, IL1A, TNFRSF21, EBI3, IL1RL1, CCL2, RELT, IL32, TNFSF18, IL33, TSLP, TNFSF15, IL34, TNFRSF9, LIF, TNFRSF10B, TNFRSF10A, INHBA, GDF5, BMP2, IL6, TNFSF4, FAS, IL7R, IL18R1	671	298	8534	2.1766270916892547	5.951504378731709E-5	8.502401378956142E-6	7.873525537317375E-6
IL17A	KEGG_PATHWAY	hsa03440:Homologous recombination	16	1.2139605462	2.0920219693	BARD1, BLM, XRCC2, XRCC3, BRCA1, BRCA2, PALB2, RAD54B, POLD3, BRIP1, RAD51, EME1, POLD1, RBBP8, RAD54L, TOPBP1	671	41	8534	4.963251063211079	7.07078500303826E-5	8.838792820358849E-6	8.185035954947692E-6
IL17A	GOTERM_BP_DIRECT	GO:0006261~DNA-templated DNA replication	15	1.1380880121	3.4304770218	RFC5, POLQ, WDHD1, GINS2, RFC3, RFC4, RFC2, GINS4, TWNK, BAZ1A, POLD3, POLD1, POLE2, POLE3, POLE	1218	45	19478	5.330596606458676	0.001590133	1.1367130646124634E-4	1.1185805432110144E-4
IL17A	GOTERM_BP_DIRECT	GO:0008284~positive regulation of cell population proliferation	63	4.7799696509	4.2675989982	CNTF, FLT1, CSF2, CSF1, BNC1, KIF14, LAMC2, HTR2A, FOXM1, AREG, CXCL5, CX3CL1, IL27RA, ESM1, EDNRB, BCL7A, NAMPT, KDR, CTSH, ITGAV, FBXO5, TIMP1, NKX3-1, TIPIN, IL15, PRMT1, SPHK1, LIFR, NRG1, OSMR, EREG, WDR77, BIRC5, CD47, KIF20B, HBEGF, EBI3, ODC1, NOP2, TTK, SSR1, HAS2, E2F3, HES1, LYN, TSLP, IL34, NTRK3, LIF, VEGFC, CDC7, FOSL1, BMP2, KITLG, IL6, GDNF, BAMBI, PRC1, TNFSF4, CDK2, BCL2, IL7R, EZH2	1218	512	19478	1.9677397629310345	0.001977781	1.3198261168489487E-4	1.298772628457739E-4
IL17A	GOTERM_BP_DIRECT	GO:0030174~regulation of DNA-templated DNA replication initiation	9	0.6828528072	4.8579284055	CDT1, MCM7, GMNN, MCM3, MCM4, MCM5, MCM6, MCM2, TICRR	1218	14	19478	10.280436312456017	0.002251056	1.3632429948471774E-4	1.3414969328470283E-4
IL17A	GOTERM_BP_DIRECT	GO:0006364~rRNA processing	27	2.048558422	4.9957169459	SUV39H1, CHD7, NOP2, NOLC1, DDX21, IPO4, RRP9, EXOSC5, EXOSC9, EXOSC8, UTP20, LYAR, EXOSC2, RRP15, DCAF13, NOP56, UTP15, NOP58, NOP14, IMP4, PA2G4, WBP11, BOP1, EBNA1BP2, DKC1, MRTO4, ESF1	1218	140	19478	3.084130893736805	0.00231483	1.3632429948471774E-4	1.3414969328470283E-4
IL17A	KEGG_PATHWAY	hsa05417:Lipid and atherosclerosis	40	3.0349013657	6.1778483214	CXCL8, PIK3R3, CXCL1, CXCL3, CXCL2, HSPD1, HSP90B1, ICAM1, RAP1B, TNFSF10, CCL2, OLR1, IL12A, BID, IKBKE, MAP3K5, ABCA1, LYN, HSPA8, HSP90AA1, VCAM1, MMP1, IL18, TNFRSF10B, TRAF2, TNFRSF10A, POU2F2, SOD2, MMP9, NFKB1, ERN1, NFKBIA, IL6, TRAF3, BCL2, FAS, CYCS, TLR6, ABCG1, TLR2	671	216	8534	2.3552464536071094	2.087895382070215E-4	2.3201252584834084E-5	2.148518360666588E-5
IL17A	GOTERM_BP_DIRECT	GO:0007080~mitotic metaphase chromosome alignment	15	1.1380880121	8.3090694949	SEH1L, CDCA5, PINX1, KIF14, CDCA8, SKA3, SKA1, CENPE, KIF18A, CCNB1, KIFC1, NUP62, RRS1, KIF2C, SPDL1	1218	48	19478	4.997434318555008	0.00384716	2.141431854824524E-4	2.1072723469010458E-4
IL17A	GOTERM_BP_DIRECT	GO:0034501~protein localization to kinetochore	8	0.6069802731	9.6539197285	MTBP, ZWILCH, CDK1, TTK, BUB3, KNL1, SPDL1, AURKB	1218	11	19478	11.630392595909838	0.004468442	2.239226681037237E-4	2.2035071780416012E-4
IL17A	GOTERM_BP_DIRECT	GO:0051256~mitotic spindle midzone assembly	8	0.6069802731	9.6539197285	RACGAP1, INCENP, PRC1, KIF4A, CDCA8, BIRC5, KIF23, AURKB	1218	11	19478	11.630392595909838	0.004468442	2.239226681037237E-4	2.2035071780416012E-4
IL17A	UP_KW_BIOLOGICAL_PROCESS	KW-0698~rRNA processing	24	1.8209408194	1.2117311111	UTP15, NOP14, UTP4, SUV39H1, NOP2, RPP40, CHD7, DDX21, IMP4, PA2G4, WDR12, WBP11, WDR43, IPO4, RRP9, BOP1, EXOSC5, DKC1, EXOSC9, EXOSC8, UTP20, EXOSC2, LYAR, DCAF13	824	106	11523	3.166239238	1.6962806986875467E-4	2.1356760833560914E-5	1.969063055576538E-5

IL17A	KEGG_PATHWAY	hsa04064:NF-kappa B signaling pathway	25	1.8968133535	1.3538400767	CXCL8, TNFAIP3, CXCL1, CXCL3, CXCL2, RELB, TNFSF13B, ICAM1, PLAU, TNFSF11, LYN, VCAM1, PARP1, GADD45A, TRAF2, TRAF1, NFKB1, NFKB2, NFKBIA, CYLD, TRAF3, BCL2, MAP3K14, BIRC2, BIRC3	671	105	8534	3.0281740117805693	4.5749357360991283E-4	4.575979459488515E-5	4.2375194402955776E-5
IL17A	GOTERM_BP_DIRECT	GO:0006271~DNA strand elongation involved in DNA replication	7	0.5311077389	1.4758918186	POLD3, POLA1, RFC3, RFC4, MCM7, MCM3, MCM4	1218	8	19478	13.992816091954023	0.006823282	3.2603153080530017E-4	3.208307691584814E-4
IL17A	KEGG_PATHWAY	hsa04210:Apoptosis	29	2.2003034901	1.7264929801	PIK3R3, ITPR2, LMNB2, CTSS, LMNB1, TUBA1C, CTSK, TNFSF10, CTSH, PMAIP1, BID, MAP3K5, PARP1, GADD45A, PARP2, TNFRSF10B, TRAF2, TNFRSF10A, TRAF1, NFKB1, ERN1, NFKBIA, BCL2, BIRC5, FAS, CYCS, MAP3K14, BIRC2, BIRC3	671	136	8534	2.711997019374069	5.833848958581855E-4	5.3050420661264674E-5	4.912657297921847E-5
IL17A	GOTERM_BP_DIRECT	GO:0036297~interstrand cross-link repair	13	0.9863429438	1.9854406751	FANCI, DCLRE1B, FANCM, CENPX, XRCC3, FANCA, FANCG, RAD51AP1, NEIL3, RAD51, FANCD2, RFWD3, ATR	1218	38	19478	5.470875464523378	0.009168182	4.1865724054118713E-4	4.11978940088493E-4
IL17A	GOTERM_BP_DIRECT	GO:0090267~positive regulation of mitotic cell cycle spindle assembly checkpoint	8	0.6069802731	2.1913994689	GEN1, INCENP, XRCC3, CDCA8, BIRC5, NDC80, AURKB, MAD2L1	1218	12	19478	10.66119321291735	0.010114415035729074	4.419957450733344E-4	4.3494515547742434E-4
IL17A	GOTERM_BP_DIRECT	GO:0000082~G1/S transition of mitotic cell cycle	19	1.4415781487	2.3743169087	USP37, BTN2A2, CCNF, CUL2, CDC7, CDC25A, CCNA2, CCNB1, DBF4, CDK6, CCNE2, CCNE1, CDK2, RBBP8, BCL2, CDK1, E2F3, POLE, EZH2	1218	82	19478	3.7054147142456646	0.010954032047523321	4.5893567249500987E-4	4.516148620262385E-4
IL17A	KEGG_PATHWAY	hsa03460:Fanconi anemia pathway	17	1.2898330804	2.8464596117	FANCI, BLM, FANCM, CENPX, FANCA, BRCA1, BRCA2, PALB2, FANCG, BRIP1, RAD51, EME1, FANCD2, UBE2T, USP1, HES1, ATR	671	55	8534	3.931120444	9.616420436936446E-4	8.017527906293994E-5	7.424515487189407E-5
IL17A	KEGG_PATHWAY	hsa04115:p53 signaling pathway	20	1.5174506828	3.3894980459	RRM2, CD82, GADD45A, SERPINE1, TNFRSF10B, TNFRSF10A, CCNB1, CDK6, CCNE2, CCNE1, CHEK1, CDK2, BCL2, CDK1, FAS, PMAIP1, CYCS, BID, GTSE1, ATR	671	75	8534	3.391554893194238	0.001144996	8.812694919373953E-5	8.160868372082981E-5
IL17A	UP_KW_BIOLOGICAL_PROCESS	KW-0159~Chromosome partition	15	1.1380880121	3.9323084052	SEH1L, KNL1, SRPK1, SGO1, SGO2, DSN1, ESPL1, PTTG1, INCENP, BIRC5, NEK2, KIF2C, BUB3, CEP85, BUB1	824	48	11523	4.370069781553397	5.503727484887921E-4	6.160616501633293E-5	5.6800010298746676E-5
IL17A	GOTERM_BP_DIRECT	GO:0090307~mitotic spindle assembly	14	1.0622154779	3.9592271297	UHRF1, CDCA8, KIF11, SMC1A, MZT1, AURKB, KIF15, CDC20, TPX2, INCENP, KIFC1, BIRC5, NEK2, MYBL2	1218	47	19478	4.763511861090731	0.018199247603552626	7.346741861980102E-4	7.229548738939246E-4
IL17A	GOTERM_BP_DIRECT	GO:1900264~positive regulation of DNA-directed DNA polymerase activity	7	0.5311077389	4.1921836475	RFC5, RFC3, RFC4, PCNA, RFC2, DSCC1, CHTF18	1218	9	19478	12.438058748403577	0.019259696	7.440621388413738E-4	7.321930726041974E-4
IL17A	GOTERM_BP_DIRECT	GO:0006955~immune response	62	4.7040971168	4.4096844617	CXCL6, ADAMDEC1, CSF2, NRROS, CXCL8, CXCL1, CXCL3, ETS1, CXCL2, CXCL5, CX3CL1, CTSS, TNFSF13B, IL27RA, CYSLTR2, IL18RAP, PNP, ADGRE5, RGS1, CTSK, TNFSF10, TNFSF11, CTSH, IL12A, IKBKE, TNFRSF4, IL4R, IL15, IL18, HLA-B, SERPINB9, PDCD1LG2, TNFRSF1B, HLA-G, TLR1, IL1A, TLR10, TLR6, TLR2, PTGER4, CD274, SEMA7A, HEPACAM, PIK3R3, C3, IL1RL1, EXOSC9, CCL2, CD58, IL32, CD74, TNFSF15, LIF, IFI44, IL6, TNFSF4, FAS, ULBP2, IL7R, MAP3K14, IL18R1, NECTIN1	1218	537	19478	1.8463518972091502	0.020248755	7.440621388413738E-4	7.321930726041974E-4
IL17A	GOTERM_BP_DIRECT	GO:0000076~DNA replication checkpoint signaling	8	0.6069802731	4.4909980356	CDT1, TIPIN, CDC45, TIMELESS, TOPBP1, DNA2, CDC6, CLSPN	1218	13	19478	9.841101427308324	0.020618262658308684	7.440621388413738E-4	7.321930726041974E-4

IL17A	GOTERM_BP_DIRECT	GO:0006913~nucleocytoplasmic transport	14	1.0622154779	5.1211746254	NDC1, RANBP1, NUP205, NUP107, SEH1L, NUP188, NUP155, NUP153, NUP85, NUP50, NUP62, NUP35, NUP88, NUP58	1218	48	19478	4.6642720306513406	0.023477209449164538	8.192113478412965E-4	8.061435229350116E-4
IL17A	GOTERM_BP_DIRECT	GO:0033209~tumor necrosis factor-mediated signaling pathway	15	1.1380880121	6.2469518293	EIF5A, TNFSF18, TRAF2, TRAF1, TNFRSF1B, TNFSF13B, NFKBIA, KRT18, TRAF3, TIFA, FAS, TNFSF11, TNFRSF4, BIRC2, BIRC3	1218	56	19478	4.283515130190008	0.028563815631453715	9.659869845427025E-4	9.505778366970116E-4
IL17A	GOTERM_BP_DIRECT	GO:0051984~positive regulation of chromosome segregation	8	0.6069802731	8.4962032310	NCAPG2, NCAPG, NCAPD3, CDC6, SMC4, RAD18, NCAPH, SMC2	1218	14	19478	9.138165611072015	0.038647425366928334	0.001271416	0.001251134
IL17A	GOTERM_BP_DIRECT	GO:0007250~activation of NF-kappaB-inducing kinase activity	7	0.5311077389	9.9236863682	TNFSF15, TNFRSF10B, ZFP91, CHI3L1, TNFRSF10A, TLR6, TLR3	1218	10	19478	11.194252873563219	0.044992619	0.001438624	0.001415676
IL17A	KEGG_PATHWAY	hsa04218:Cellular senescence	30	2.2761760242	1.0900238966	CXCL8, SERPINE1, PIK3R3, ITPR2, LIN9, FOXM1, ETS1, CCNB1, RASSF5, CHEK1, E2F1, E2F2, E2F3, MYBL2, E2F5, GADD45A, HLA-B, HLA-G, CDC25A, NFKB1, CCNA2, IL1A, IL6, RBL1, CDK6, CCNE2, CCNE1, CDK2, CDK1, ATR	671	157	8534	2.430254302	0.003677522	2.631629121847157E-4	2.436981997450178E-4
IL17A	GOTERM_BP_DIRECT	GO:0051315~attachment of mitotic spindle microtubules to kinetochore	8	0.6069802731	1.5069583695	CDT1, CENPE, SEH1L, NUF2, KIF2C, SKA3, NDC80, SKA1	1218	15	19478	8.52895457	0.067520699	0.002118418	0.002084626
IL17A	KEGG_PATHWAY	hsa05200:Pathways in cancer	70	5.3110773899	1.7869701326	CXCL8, HHIP, LAMC2, BRCA2, ETS1, CKS1B, EDNRB, RASSF5, BDKRB1, IL12A, ITGAV, JAK3, NKX3-1, IFNAR2, IL15RA, HSP90AA1, IL4R, IL15, MMP1, HGF, ITGA2, TRAF2, TRAF1, MMP9, CCNA2, TRAF4, CCNE2, MSH2, CCNE1, TRAF3, COL4A4, CKS2, BIRC5, ITGA6, BIRC2, BIRC3, PTGER4, CUL2, PIK3R3, ADCY1, RASGRP1, HSP90B1, RASGRP3, DLL4, E2F1, E2F2, PMAIP1, E2F3, HES1, BID, WNT10B, ARNT2, EGLN3, LAMB3, GADD45A, WNT7B, VEGFC, NFKB1, NFKB2, NFKBIA, BMP2, KITLG, IL6, RAD51, CDK6, CDK2, BCL2, CYCS, FAS, IL7R	671	533	8534	1.6703248770421901	0.006021809	4.0266393656106827E-4	3.7288110101661053E-4
IL17A	GOTERM_BP_DIRECT	GO:0007018~microtubule-based movement	17	1.2898330804	2.5036694430	DNAH11, DNAH14, KIF14, KIF24, KIF23, KIF11, KIF15, CENPE, KIF18A, KIF18B, KIFC1, KIF4A, KIF21A, KIF2C, KIF1A, KIF21B, KIF20B	1218	79	19478	3.4412712269543344	0.10965538152628729	0.003359181	0.003305597
IL17A	GOTERM_BP_DIRECT	GO:0051310~metaphase chromosome alignment	8	0.6069802731	2.5344114310	CENPE, CENPF, INCENP, KIF2C, FAM83D, SKA3, NDC80, SKA1	1218	16	19478	7.995894909688013	0.11092424749533314	0.003359181	0.003305597
IL17A	GOTERM_BP_DIRECT	GO:0006606~protein import into nucleus	20	1.5174506828	2.8095870256	IL33, NUP107, NUP188, NUP155, CSE1L, NUP153, RANBP6, IPO7, IPO4, NFKBIA, POLA2, NUP85, NUP50, SIX2, NUP62, BCL3, HEATR3, E2F3, NUP88, KPNA2	1218	106	19478	3.0173188338445334	0.12220181826292109	0.003620465	0.003562712
IL17A	KEGG_PATHWAY	hsa05168:Herpes simplex virus 1 infection	32	2.4279210925	2.9400028838	PIK3R3, IFIH1, C3, CCL2, IL12A, BID, IKBKE, IFNAR2, CD74, HLA-B, TAP2, TAP1, TRAF2, POU2F2, HLA-G, NFKB1, SRPK1, TAPBP, NFKBIA, IL6, TRAF3, OAS3, BCL2, SRSF2, FAS, CYCS, SRSF7, BIRC2, TLR3, BIRC3, TLR2, NECTIN1	671	182	8534	2.236190039468728	0.009888143	6.210756092048226E-4	5.751380641452942E-4
IL17A	KEGG_PATHWAY	hsa03430:Mismatch repair	10	0.7587253414	3.2154440641	RFC5, POLD3, RFC3, RFC4, PCNA, MSH2, LIG1, EXO1, RFC2, POLD1	671	23	8534	5.529709064990605	0.010809528260973145	6.393059374569127E-4	5.920199953373185E-4
IL17A	GOTERM_BP_DIRECT	GO:1990830~cellular response to leukemia inhibitory factor	20	1.5174506828	3.6809271742	TLE4, HELLS, TFRC, GLDC, SHMT1, GFPT2, VEGFC, SRM, ICAM1, CACNB4, MAT2A, NCL, ADAM23, NUP35, TNFSF11, MYBL2, EEF1E1, MSC, SRSF7, INA	1218	108	19478	2.9614425591437086	0.15697726689627733	0.004615087	0.004541468

IL17A	KEGG_PATHWAY	hsa04657:IL-17 signaling pathway	21	1.5933232169	3.6983657025	CXCL6, HSP90AA1, CXCL8, CSF2, MMP1, TNFAIP3, CXCL1, TRAF2, CXCL3, CXCL2, MMP9, CXCL5, NFKB1, HSP90B1, NFKBIA, FOSL1, IL6, TRAF4, TRAF3, CCL2, IKBKE	671	95	8534	2.8114205035689075	0.012422898	6.579197934048776E-4	6.092570867920906E-4
IL17A	KEGG_PATHWAY	hsa05323:Rheumatoid arthritis	21	1.5933232169	3.6983657025	CXCL6, FLT1, CXCL8, CSF2, CSF1, IL15, MMP1, IL18, CXCL1, CXCL3, CXCL2, CXCL5, TNFSF13B, ICAM1, IL1A, IL6, CTSK, CCL2, TNFSF11, ATP6V1C2, TLR2	671	95	8534	2.8114205035689075	0.012422898	6.579197934048776E-4	6.092570867920906E-4
IL17A	UP_KW_BIOLOGICAL_PROCESS	KW-0395~Inflammatory response	31	2.3520485584	3.7985661140	SEMA7A, CXCL8, CSF1, TNFAIP3, CXCL1, CXCL3, CXCL2, FUT4, CX3CL1, C3, ADGRE2, IL18RAP, ZC3H12A, CCL2, OLR1, LYN, IL34, IL18, TLR1, IL1A, MFHAS1, ELF3, NLRP10, CHI3L1, TLR10, CD47, TLR6, CHST2, IL18R1, TLR3, TLR2	824	192	11523	2.257869387135922	0.005303978	5.355978220809075E-4	4.938135948263687E-4
IL17A	GOTERM_BP_DIRECT	GO:0007052~mitotic spindle organization	14	1.0622154779	3.8418769910	STIL, TTK, WDR62, KIF11, NDC80, AURKB, CENPE, CCNB1, KIF4A, NUF2, TACC3, BIRC5, DLGAP5, SPC25	1218	57	19478	3.9278080258116557	0.16324845903827678	0.004690123	0.004615307
IL17A	GOTERM_BP_DIRECT	GO:0008608~attachment of spindle microtubules to kinetochore	8	0.6069802731	4.0761295096	SGO1, DSN1, NUF2, BUB3, KNL1, NDC80, SPC24, SPC25	1218	17	19478	7.5255481502946004	0.17229254468931454	0.004848504	0.004771162
IL17A	GOTERM_BP_DIRECT	GO:0006139~nucleobase-containing compound metabolic process	11	0.834597876	4.9722230568	PRPS2, BRIP1, PNP, TPMT, OAS3, DDX11, DDX12P, CTPS1, UPP1, TK1, ATR	1218	36	19478	4.886380222587119	0.2059963035731015	0.005766536	0.00567455
IL17A	GOTERM_BP_DIRECT	GO:0042254~ribosome biogenesis	12	0.9104704097	6.1275916883	NOP56, LTV1, RCL1, BOP1, EBNA1BP2, MYBBP1A, GRWD1, DHX37, TSR1, URB2, GNL2, GTPBP4	1218	44	19478	4.361397223466189	0.2474348348434401	0.006933146	0.00682255
IL17A	GOTERM_BP_DIRECT	GO:0051298~centrosome duplication	8	0.6069802731	6.3106416253	STIL, CEP152, CDK2, CHORDC1, SASS6, CCP110, BRCA2, NDC80	1218	18	19478	7.107462141944901	0.25379871983069757	0.006970254	0.006859066
IL17A	GOTERM_BP_DIRECT	GO:0000086~G2/M transition of mitotic cell cycle	13	0.9863429438	6.7072755791	WNT10B, CCDC57, PKMYT1, FOXM1, MASTL, CDC25A, CCNA2, CCNB1, MELK, CHEK1, CDK2, CDK1, BIRC5	1218	52	19478	3.9979474548440073	0.2674040445338338	0.007236058	0.007120631
IL17A	GOTERM_BP_DIRECT	GO:0043066~negative regulation of apoptotic process	56	4.248861912	7.1307230699	ANKLE2, TFRC, HHIP, KIF14, CX3CL1, EDNRB, RPS6KA1, NUP62, CTSH, PIM3, TIMP1, SERPINB2, HGF, SPHK1, PLAUR, SERPINB9, DKK1, ATAD3A, MMP9, DNAJC3, PPIF, BIRC5, BIRC2, BIRC3, GATA6, HSPD1, HSP90B1, UNG, SOCS2, NUAK2, CHST11, FIGNL1, PDPN, APBB2, NTSR1, TNFSF18, BARD1, CD74, STIL, TSLP, PA2G4, SOD2, PALB2, NFKB1, SH3RF2, IL6, KRT18, MIR221, GDNF, BCL3, BCL2, CDK1, FAS, NAA15, EVI2B, MAD2L1	1218	515	19478	1.7389130677379756	0.281655513	0.00742948	0.007310967
IL17A	GOTERM_BP_DIRECT	GO:1902975~mitotic DNA replication initiation	5	0.3793626707	7.2068673768	POLA1, GINS3, MCM3, MCM4, MCM2	1218	5	19478	15.991789819376029	0.2841886507405399	0.00742948	0.007310967
IL17A	KEGG_PATHWAY	hsa05134:Legionellosis	15	1.1380880121	7.6884899070	HSPA8, CXCL8, IL18, CXCL1, CXCL3, CXCL2, NFKB1, HSPD1, NFKB2, C3, NFKBIA, IL6, CYCS, IL12A, TLR2	671	56	8534	3.4066957632531403	0.025653311	0.001299355	0.001203249
IL17A	GOTERM_BP_DIRECT	GO:0042274~ribosomal small subunit biogenesis	16	1.2139605462	8.3430564025	NOP56, UTP15, LTV1, NOP58, NOP14, UTP4, PNO1, WDR3, IMP4, WDR43, IPO4, RRP9, RCL1, DHX37, UTP20, RIOK1	1218	78	19478	3.280367142436108	0.32094322048738977	0.008413791	0.008279577
IL17A	GOTERM_BP_DIRECT	GO:1905821~positive regulation of chromosome condensation	6	0.4552352048	9.6260357306	NCAPG2, NCAPG, NCAPD3, SMC4, NCAPH, SMC2	1218	9	19478	10.661193212917352	0.36018313469814545	0.009501102	0.009349543

IL17A	GOTERM_BP_DIRECT	GO:0030335~positive regulation of cell migration	36	2.7314112291	1.2622395692	SEMA5A, CD274, SEMA7A, FLT1, CSF2, CSF1, SEMA3A, NEDD9, PIK3R3, LAMC2, SEMA3E, CLDN1, CX3CL1, PLAU, PODXL, PDPN, KDR, CCL2, HAS2, CTSH, ITGAV, EPHB2, LYN, HSPA8, HGF, SPHK1, NTRK3, SOD2, SH3RF2, BMP2, CLDN4, CDH13, ITGA6, EZH2, HBEGF, EPHA2	1218	288	19478	1.9989737274220034	0.4432226750724879	0.012199019503870503	0.012004424236940904
IL17A	GOTERM_BP_DIRECT	GO:0006302~double-strand break repair	16	1.2139605462	1.5057973610	POLQ, FEN1, PARP1, ZGRF1, PARP2, CDCA5, BRCA1, ESCO2, BRCA2, BRIP1, EME1, MSH2, TDP1, NSD2, TRIP13, ATR	1218	82	19478	3.1203492330489806	0.5027150086534768	0.014255906036287378	0.014028499904214677
IL17A	GOTERM_BP_DIRECT	GO:0000281~mitotic cytokinesis	14	1.0622154779	1.6174863294	CKAP2, CDCA8, KIF23, AURKB, ANLN, ESPL1, RACGAP1, INCENP, KIF4A, NUSAP1, BIRC5, EXOC6, ECT2, CEP55	1218	65	19478	3.4443854995579133	0.5278283050035566	0.014845392013933199	0.014608582570296413
IL17A	GOTERM_BP_DIRECT	GO:0071222~cellular response to lipopolysaccharide	27	2.048558422	1.6320650845	CD274, CXCL6, CSF2, CXCL8, SERPINE1, TNFAIP3, CXCL1, CXCL3, CXCL2, CXCL5, EDNRB, NFKBIZ, ZC3H12A, ANKRD1, CCL2, EPHB2, ABCA1, IL18, PDCD1LG2, MMP8, TNFRSF1B, MMP9, NFKB1, IL1A, IL6, TNFSF4, NFKBIB	1218	192	19478	2.2488454433497536	0.5310113834040406	0.014845392013933199	0.014608582570296413
IL17A	GOTERM_BP_DIRECT	GO:0002224~toll-like receptor signaling pathway	9	0.6828528072	1.7281727636	TLR1, FOSL1, TRAF3, NFKBIZ, TLR10, TLR6, CTSS, TLR3, TLR2	1218	27	19478	5.330596606458675	0.5514650287881078	0.015417295	0.015171362819702178
IL17A	GOTERM_BP_DIRECT	GO:0034080~CENP-A containing chromatin assembly	6	0.4552352048	1.8259702823	CENPW, NASP, CENPI, HJURP, OIP5, CENPN	1218	10	19478	9.595073891625617	0.571363037	0.015686437	0.015436211739167647
IL17A	GOTERM_BP_DIRECT	GO:0035825~homologous recombination	6	0.4552352048	1.8259702823	BARD1, BRIP1, UHRF1, RBBP8, BRCA1, TOPBP1	1218	10	19478	9.595073891625617	0.571363037	0.015686437	0.015436211739167647
IL17A	GOTERM_BP_DIRECT	GO:0050729~positive regulation of inflammatory response	19	1.4415781487	1.9423241853	TNFSF18, PTGER4, IL33, TSLP, IL15, SERPINE1, IL18, HTR2A, ETS1, CX3CL1, NFKBIA, IL1RL1, GPRC5B, NLRP10, TNFSF4, NFKBIZ, CD47, TLR3, TLR2	1218	112	19478	2.7128929157870045	0.5938901976932006	0.016182384732303306	0.015924248
IL17A	GOTERM_BP_DIRECT	GO:0038061~non-canonical NF-kappaB signal transduction	8	0.6069802731	1.9534674391	NFKBIA, TRAF2, MAP3K14, NFKB1, BIRC2, RELB, NFKB2, BIRC3	1218	21	19478	6.092110407381344	0.5959845137568685	0.016182384732303306	0.015924248
IL17A	GOTERM_BP_DIRECT	GO:0019221~cytokine-mediated signaling pathway	22	1.6691957511	1.9898977475	IL4R, TSLP, EBI3, IL18, LIFR, INHBA, OSMR, LRP8, CX3CL1, EREG, IL27RA, SOCS2, FOSL1, IL1A, IL6, IL17REL, NFKBIZ, CCL2, TNFSF11, IL7R, CNOT9, JAK3	1218	142	19478	2.4776012396216376	0.6027563356030983	0.016194975	0.015936637223674843
IL17A	GOTERM_BP_DIRECT	GO:0010971~positive regulation of G2/M transition of mitotic cell cycle	9	0.6828528072	2.2878021338	WNT10B, RRM1, CCNB1, RCC2, CDK1, CDC7, FBXO5, DTL, CDC25A	1218	28	19478	5.140218156228008	0.6540402471441124	0.018207632837849236	0.01791719
IL17A	GOTERM_BP_DIRECT	GO:0010628~positive regulation of gene expression	52	3.9453717754	2.3156937646	BMP10, CNTF, CSF2, CXCL8, CSF1, TFRC, PDCD5, BRCA1, PRDM1, ETS1, ROBO1, GJA1, CYP26B1, C1QTNF1, ZC3H12A, NAMPT, TRIM24, TNFSF11, CTSH, EPHB2, IKBKE, CD34, NKX3-1, NOG, ANK2, DKK1, IL1A, RNF207, TLR6, ADTRP, TLR3, TLR2, DNMT1, PIK3R3, DLL4, E2F1, CCL2, HES1, NTSR1, IL32, CD74, IL33, IL34, NTRK3, LIF, INHBA, BMP2, IL6, CDK6, CDK1, ID3, IL7R	1218	489	19478	1.7005584266003138	0.6584887775818535	0.018207632837849236	0.01791719
IL17A	GOTERM_BP_DIRECT	GO:0043254~regulation of protein-containing complex assembly	8	0.6069802731	2.7113213034	TNFSF18, HSPA8, HSP90AA1, NCLN, HJURP, HES1, TRAF2, IKBKE	1218	22	19478	5.815196297954919	0.7157645957764673	0.020963033	0.020628636250415503
IL17A	GOTERM_BP_DIRECT	GO:0042273~ribosomal large subunit biogenesis	10	0.7587253414	2.8511731478	EBNA1BP2, NOP16, PAK1IP1, NIP7, MRTO4, NOP2, RRS1, HEATR3, WDR12, GTPBP4	1218	36	19478	4.4421638387155635	0.7336243679219928	0.021682938087003015	0.021337058

IL17A	KEGG_PATHWAY	hsa05166:Human T-cell leukemia virus 1 infection	34	2.5796661608	3.0146256890	CSF2, PIK3R3, BUB1B, ADCY1, ETS1, RELB, ICAM1, CDC20, PTTG1, CHEK1, E2F1, E2F2, E2F3, BUB3, JAK3, RANBP1, IL15RA, EGR2, IL15, HLA-B, HLA-G, NFKB1, NFKB2, NFKBIA, CCNA2, FOSL1, IL6, ESPL1, CCNE2, CCNE1, CDK2, MAP3K14, ATR, MAD2L1	671	224	8534	1.9304609325101127	0.09688891	0.004852112	0.004493228
IL17A	GOTERM_BP_DIRECT	GO:0006275~regulation of DNA replication	11	0.834597876	3.0737962682	INO80C, CCNA2, USP37, PCNA, DSCC1, POLE3, GMNN, ID3, ESCO2, BAZ1A, FBXO5	1218	44	19478	3.9979474548440064	0.7597687517860717	0.022318734	0.021962711991199604
IL17A	GOTERM_BP_DIRECT	GO:0001525~angiogenesis	33	2.5037936267	3.1011797527	ROBO4, FLT1, CXCL8, PDE3B, SERPINE1, TNFAIP2, NRXN3, THY1, TYMP, ARHGAP22, DLL4, ESM1, TMEM201, ZC3H12A, KDR, CCL2, ITGAV, EPHB2, EPHB1, COL27A1, ANXA2, C1GALT1, ERAP1, IL18, VEGFC, EREG, NCL, COL8A2, MMP19, ADAM8, CD47, NAA15, EPHA2	1218	267	19478	1.9765133484622057	0.7628020862141914	0.022318734	0.021962711991199604
IL17A	GOTERM_BP_DIRECT	GO:1905820~positive regulation of chromosome separation	6	0.4552352048	3.1753318541	NCAPG2, NCAPG, NCAPD3, SMC4, NCAPH, SMC2	1218	11	19478	8.722794446932378	0.7708252269982978	0.022318734	0.021962711991199604
IL17A	GOTERM_BP_DIRECT	GO:0033314~mitotic DNA replication checkpoint signaling	6	0.4552352048	3.1753318541	ORC1, DONSON, TOPBP1, CDC6, CLSPN, TICRR	1218	11	19478	8.722794446932378	0.7708252269982978	0.022318734	0.021962711991199604
IL17A	GOTERM_BP_DIRECT	GO:0051988~regulation of attachment of spindle microtubules to kinetochore	6	0.4552352048	3.1753318541	SPAG5, RACGAP1, ZWILCH, KNTC1, NEK2, ECT2	1218	11	19478	8.722794446932378	0.7708252269982978	0.022318734	0.021962711991199604
IL17A	GOTERM_BP_DIRECT	GO:0007099~centriole replication	8	0.6069802731	3.6885636563	PLK4, CCDC57, CEP152, CDK2, SASS6, CCP110, WDR62, CEP85	1218	23	19478	5.562361676304705	0.8193945276296742	0.025468646	0.025062377
IL17A	GOTERM_BP_DIRECT	GO:0032722~positive regulation of chemokine production	11	0.834597876	3.7332785189	IL33, IL1RL1, CD74, IL6, IL4R, TSLP, ADORA2B, TNFSF4, IL18, TLR3, TLR2	1218	45	19478	3.9091041780696956	0.8231036414251192	0.025468646	0.025062377
IL17A	GOTERM_BP_DIRECT	GO:0016477~cell migration	33	2.5037936267	4.0398821829	ERRFI1, FLT1, WWC1, NEDD9, TNFAIP3, CORO1A, RND1, ADGRE2, STRIP2, PODXL, SIX2, PDPN, KDR, BDKRB1, IL12A, HES1, ITGAV, SPDL1, FAM83D, GFRA1, L1CAM, MMP9, DEPDC1B, BAMBI, CDK1, SDC1, CDH13, DCHS1, CD47, CEP85, DOCK2, EPHA3, EPHA2	1218	271	19478	1.9473397197026159	0.8465652684633479	0.027160889053286806	0.026727626
IL17A	KEGG_PATHWAY	hsa05164:Influenza A	28	2.124430956	4.1728381622	NXT1, CXCL8, PIK3R3, ICAM1, IFIH1, TNFSF10, CCL2, IL12A, BID, KPNA2, IKBKE, IFNAR2, IL33, IL18, TNFRSF10B, TNFRSF10A, NFKB1, DNAJC3, NFKBIA, IL1A, IL6, CDK6, TRAF3, OAS3, FAS, CYCS, TLR3, NFKBIB	671	173	8534	2.0584581721699133	0.13157266611617646	0.006410997	0.005936811
IL17A	GOTERM_BP_DIRECT	GO:0007249~canonical NF-kappaB signal transduction	10	0.7587253414	4.4099025626	NFKBIA, IRAK2, BCL3, ADAM8, MAP3K14, NFKB1, BIRC2, RELB, NFKB2, BIRC3	1218	38	19478	4.208365741941059	0.8707756205761678	0.029225054268561618	0.028758864569084667
IL17A	KEGG_PATHWAY	hsa00240:Pyrimidine metabolism	14	1.0622154779	4.4241544726	RRM1, RRM2, CAD, CTPS1, TYMS, TYMP, DHODH, NME1, NME1-NME2, NT5E, UCK2, TK1, UPP1, DCTPP1	671	58	8534	3.069941929184439	0.13892141724360563	0.006501584	0.006020697
IL17A	KEGG_PATHWAY	hsa05162:Measles	24	1.8209408194	4.7272789784	IFNAR2, HSPA8, PIK3R3, TNFAIP3, NFKB1, IFIH1, NFKBIA, IL1A, IL6, CDK6, CCNE2, CCNE1, TRAF3, OAS3, CDK2, BCL2, FAS, CYCS, IL12A, BID, JAK3, IKBKE, NFKBIB, TLR2	671	139	8534	2.1959707941545425	0.1477026477811404	0.006657585	0.00616516
IL17A	GOTERM_BP_DIRECT	GO:0051321~meiotic cell cycle	15	1.1380880121	5.1750107115	XRCC2, OSGIN2, PKMYT1, SMC1A, PSMC3IP, RAD51AP1, SGO2, RAD51, EXO1, DMC1, CDK2, RBBP8, RAD54L, NEK2, BUB3	1218	82	19478	2.9253274059834196	0.9094007063790408	0.0338125	0.033273132250948655

IL17A	GOTERM_BP_DIRECT	GO:0030198~extracellular matrix organization	23	1.7450682852	5.7363727889	COL27A1, MMP1, COL22A1, ITGA2, PAPLN, MMP8, MMP9, COL19A1, MMP10, NFKB2, ADAMTS4, ADAMTSL1, ADAMTS3, EGFLAM, ELF3, ABI3BP, BCL3, COL8A2, MMP19, APBB2, PTX3, SLC39A8, ADAMTS6	1218	164	19478	2.242751011253955	0.9301822415741681	0.036959769	0.036370196919090526
IL17A	GOTERM_BP_DIRECT	GO:0045740~positive regulation of DNA replication	9	0.6828528072	6.1928924864	CDT1, PCNA, ATAD5, CDK2, CDK1, DNA2, BAZ1A, CDC25A, EREG	1218	32	19478	4.497690886699507	0.9435145821134949	0.038822741	0.038203452
IL17A	GOTERM_BP_DIRECT	GO:0071897~DNA biosynthetic process	9	0.6828528072	6.1928924864	POLD3, CENPF, LIG1, POLD1, SPHK1, POLE3, TK1, LIN9, TYMS	1218	32	19478	4.497690886699507	0.9435145821134949	0.038822741	0.038203452
IL17A	BIOCARTA	h_deathPathway:Induction of apoptosis through DR3 and DR4/5 Death Receptors	12	0.9104704097	6.3877559159	NFKBIA, BCL2, TNFSF10, TNFRSF10B, CYCS, TRAF2, TNFRSF10A, BID, MAP3K14, NFKB1, BIRC2, BIRC3	186	33	1622	3.1710654936461387	0.12445416588298297	0.057322434	0.056768594
IL17A	KEGG_PATHWAY	hsa03013:Nucleocytoplasmic transport	20	1.5174506828	6.6579952291	NDC1, NUP205, NUP107, SEH1L, NXT1, NUP188, NUP155, CSE1L, NUP153, IPO7, IPO4, TMEM33, NUP85, NUP50, XPO5, NUP62, NUP35, NUP88, KPNA2, NUP58	671	108	8534	2.3552464536071094	0.2015757553138775	0.00900161	0.00833581
IL17A	GOTERM_BP_DIRECT	GO:0006915~apoptotic process	63	4.7799696509	7.5163454708	ERRFI1, PDCD5, BUB1B, TNFAIP3, AHR, RASSF5, DPF1, ZC3H12A, CHEK1, TNFSF10, CTSH, PIM3, MAP3K9, PHLDA2, TP63, NKX3-1, HELLS, RBM14, PARP1, CKAP2, TRAF1, MMP9, IL1A, MELK, TRAF4, ESPL1, TRAF3, PTRH2, IRF1, BIRC5, CHI3L1, CD47, BIRC2, TNFRSF21, TLR2, BIRC3, MCM2, MTFP1, CDCA7, DRAM1, NUAK2, CHST11, PMAIP1, BID, BUB1, RELT, EGLN3, GALNT3, DDIAS, GADD45A, TNFRSF9, EAF2, TNFRSF10B, TNFRSF10A, SULF1, PALB2, NFKB1, TPX2, BCL2, CDK1, CYCS, FAS, XAF1	1218	656	19478	1.5357968881412951	0.9694424574814966	0.046491102	0.045749489
IL17A	GOTERM_BP_DIRECT	GO:0007051~spindle organization	7	0.5311077389	7.8684774031	ASPM, RANBP1, SPAG5, TTK, AUNIP, KIF11, AURKB	1218	19	19478	5.8917120387174835	0.9740510333677416	0.047345458	0.046590217
IL17A	GOTERM_BP_DIRECT	GO:0007076~mitotic chromosome condensation	7	0.5311077389	7.8684774031	CDCA5, NUSAP1, NCAPG, NCAPD3, SMC4, NCAPH, SMC2	1218	19	19478	5.8917120387174835	0.9740510333677416	0.047345458	0.046590217
IL17A	GOTERM_BP_DIRECT	GO:0051382~kinetochore assembly	6	0.4552352048	7.9606504443	CENPE, CENPF, CENPW, CENPX, CENPK, DLGAP5	1218	13	19478	7.380826070481243	0.9751380419814522	0.047345458	0.046590217
IL17A	BIOCARTA	h_hivnefPathway:HIV-I Nef: negative effector of Fas and TNF	17	1.2898330804	8.3075991246	PARP1, TRAF2, TRAF1, TNFRSF1B, NFKB1, LMNB2, LMNB1, NFKBIA, ARHGDIB, BCL2, FAS, CYCS, BID, MAP3K14, BIRC2, BIRC3, MAP3K5	186	61	1622	2.4302838004583114	0.15875292788067152	0.057322434	0.056768594
IL17A	KEGG_PATHWAY	hsa05161:Hepatitis B	26	1.9726858877	8.7479898895	PCNA, CXCL8, PIK3R3, IFIH1, E2F1, E2F2, E2F3, BID, JAK3, IKBKE, EGR2, MMP9, NFKB1, NFKBIA, CCNA2, IL6, CCNE2, CCNE1, TRAF3, CDK2, BCL2, BIRC5, FAS, CYCS, TLR3, TLR2	671	163	8534	2.028690810346246	0.2560723287411262	0.011372386856404884	0.010531234
IL17A	GOTERM_BP_DIRECT	GO:0006272~leading strand elongation	4	0.3034901365	9.2789753558	POLA1, PCNA, POLE3, POLE	1218	4	19478	15.991789819376026	0.9865195525310598	0.053806458	0.052948153
IL17A	GOTERM_BP_DIRECT	GO:1901970~positive regulation of mitotic sister chromatid separation	4	0.3034901365	9.2789753558	INCENP, CDCA8, BIRC5, AURKB	1218	4	19478	15.991789819376026	0.9865195525310598	0.053806458	0.052948153
IL17A	GOTERM_BP_DIRECT	GO:0098609~cell-cell adhesion	25	1.8968133535	0.001233529	CLSTN3, THY1, COL19A1, CX3CL1, ICAM1, ITGAV, CD58, STXBP6, PDLIM5, CD34, CDON, DSP, VCAM1, ITGA4, ANXA2, ITGA2, KRT18, VNN1, IGDCC4, COL8A2, BCL2, ITGA6, ADAM8, TJP2, NECTIN1	1218	196	19478	2.039769109614289	0.9967394894547067	0.070646164	0.069519236

IL17A	GOTERM_BP_DIRECT	GO:0071260~cellular response to mechanical stimulus	14	1.0622154779	0.001302121	PTGER4, IL33, GADD45A, ITGA2, TNFRSF10B, TNFRSF10A, NFKB1, IRF1, CHEK1, ANKRD1, FAS, TNFRSF8, MAP3K14, TLR3	1218	80	19478	2.7985632183908047	0.9976290724153142	0.073665096	0.072490011
IL17A	GOTERM_BP_DIRECT	GO:0000079~regulation of cyclin-dependent protein serine/threonine kinase activity	8	0.6069802731	0.001353651	BLM, CCNE2, GADD45A, CDC6, PKMYT1, CDC25A, GTPBP4, CDK5R1	1218	28	19478	4.5690828055360075	0.9981337784500799	0.074679063	0.073487804
IL17A	GOTERM_BP_DIRECT	GO:0010332~response to gamma radiation	8	0.6069802731	0.001353651	IL1A, TIGAR, PARP1, FANCD2, XRCC2, BCL2, BRCA2, SOD2	1218	28	19478	4.5690828055360075	0.9981337784500799	0.074679063	0.073487804
IL17A	GOTERM_BP_DIRECT	GO:0030177~positive regulation of Wnt signaling pathway	10	0.7587253414	0.001368338	BMP2, DEPDC1B, HHEX, TMEM132A, RSPO3, TNFAIP3, SULF1, HSP90B1, ATP6V1C2, TLR2	1218	44	19478	3.6344976862218243	0.9982568600305453	0.074679063	0.073487804
IL17A	GOTERM_BP_DIRECT	GO:0030225~macrophage differentiation	9	0.6828528072	0.001426254	IL33, IL1RL1, DIAPH3, CSF2, CSF1, PARP1, IL15, LIF, MMP9	1218	36	19478	3.997947454844007	0.9986680527233708	0.076934773	0.075707531
IL17A	GOTERM_BP_DIRECT	GO:0000447~endonucleolytic cleavage in ITS1 to separate SSU-rRNA from 5.8S rRNA and LSU-rRNA from tricistronic rRNA transcript (SSU-rRNA, 5.8S rRNA, LSU-rRNA)	5	0.3793626707	0.001481989	RCL1, NOP14, RPP40, RRS1, UTP20	1218	9	19478	8.884327677431127	0.9989719027885463	0.07902237	0.077761828
IL17A	GOTERM_BP_DIRECT	GO:0008283~cell population proliferation	21	1.5933232169	0.00161769	USP13, CSF2, ATAD5, MCM7, SLC20A1, SPHK1, PAK1IP1, ODC1, IL18, NRG1, MCM10, MKI67, CDC25A, RAP1B, BOP1, IL18RAP, MELK, SIX2, OTUD6B, POLR3G, FAM83D	1218	155	19478	2.1666295884315905	0.9994527158532165	0.084321978	0.082976898
IL17A	GOTERM_BP_DIRECT	GO:0032755~positive regulation of interleukin-6 production	16	1.2139605462	0.001639506	CD74, IL33, TSLP, POU2F2, HSPD1, EREG, IFIH1, TLR1, IL1A, IL6, NLRP10, ADORA2B, TNFSF4, TLR6, TLR3, TLR2	1218	102	19478	2.50851605	0.9995054728541128	0.084321978	0.082976898
IL17A	GOTERM_BP_DIRECT	GO:0042102~positive regulation of T cell proliferation	12	0.9104704097	0.001655854	CD274, IL6, KITLG, VCAM1, PNP, TFRC, IL15, TNFSF4, HES1, PDCD1LG2, CORO1A, TNFSF13B	1218	63	19478	3.046055203690672	0.9995416475569986	0.084321978	0.082976898
IL17A	GOTERM_BP_DIRECT	GO:2000001~regulation of DNA damage checkpoint	6	0.4552352048	0.001672262	BARD1, RAD51, RFWD3, BRCA1, BRCA2, WDR76	1218	15	19478	6.396715928	0.9995752949568868	0.084321978	0.082976898
IL17A	GOTERM_BP_DIRECT	GO:0001833~inner cell mass cell proliferation	6	0.4552352048	0.001672262	GINS1, CHEK1, GINS4, NCAPG2, BRCA2, PALB2	1218	15	19478	6.396715928	0.9995752949568868	0.084321978	0.082976898
IL17A	GOTERM_BP_DIRECT	GO:0008285~negative regulation of cell population proliferation	44	3.3383915022	0.001810983	BTG3, CXCL8, WWC1, PINX1, CXCL1, ETS1, RERG, DLL4, MTBP, CYP27B1, BRIP1, ABI3BP, PDPN, NUP62, TNFRSF8, UTP20, SH2B3, E2F7, NKX3-1, KLF10, LYN, TNFRSF9, RARRES1, LIF, HMGA1, P3H2, CDC6, INHBA, SSTR2, SOD2, GTPBP4, EREG, NME1, FOSL1, IL1A, BMP2, IL6, CACNB4, CDK6, MIR221, IRF1, CDH13, EEF1E1, TLR2	1218	433	19478	1.6250317599365938	0.9997770950888334	0.090334935	0.088893938
IL17A	GOTERM_BP_DIRECT	GO:0030490~maturation of SSU-rRNA	7	0.5311077389	0.001841176	NOP14, UTP4, WDR3, SRFBP1, IMP4, RIOK1, IPO4	1218	22	19478	5.088296760710554	0.9998062780192033	0.090863978	0.089414542

IL17A	KEGG_PATHWAY	hsa04061:Viral protein interaction with cytokine and cytokine receptor	18	1.3657056145	0.001851637	CXCL6, CXCL8, CSF1, IL34, IL18, TNFRSF10B, CXCL1, TNFRSF10A, CXCL3, TNFRSF1B, CXCL2, CXCL5, CX3CL1, IL6, IL18RAP, TNFSF10, CCL2, IL18R1	671	100	8534	2.2892995529061104	0.46550522089827007	0.023179747177960452	0.021465269
IL17A	GOTERM_BP_DIRECT	GO:0097191~extrinsic apoptotic signaling pathway	11	0.834597876	0.001940876	IL33, KRT18, PARP2, FAS, G0S2, IL12A, TNFRSF10A, INHBA, BID, TNFRSF1B, TLR3	1218	55	19478	3.1983579638752047	0.9998781201349947	0.094776061	0.093264221
IL17A	GOTERM_BP_DIRECT	GO:0007095~mitotic G2 DNA damage checkpoint signaling	9	0.6828528072	0.002062937	BLM, CHEK1, CDK1, DONSON, BRCA1, TOPBP1, CLSPN, DTL, TICRR	1218	38	19478	3.7875291677469534	0.9999308925293285	0.099687115	0.098096934
IL17A	GOTERM_BP_DIRECT	GO:0007346~regulation of mitotic cell cycle	12	0.9104704097	0.002147693	CDC20, CYLD, CDK2, GMNN, CKS2, BIRC5, PIM3, TTLL12, FBXO5, MASTL, DLGAP5, CKS1B	1218	65	19478	2.952330428192497	0.999953398	0.1025980180280815	0.10096140381508774
IL17A	GOTERM_BP_DIRECT	GO:0046601~positive regulation of centriole replication	4	0.3034901365	0.002211641	PLK4, STIL, NUP62, SASS6	1218	5	19478	12.793431855500822	0.9999653829442635	0.1025980180280815	0.10096140381508774
IL17A	GOTERM_BP_DIRECT	GO:0046602~regulation of mitotic centrosome separation	4	0.3034901365	0.002211641	CHEK1, NEK2, KIF11, CEP85	1218	5	19478	12.793431855500822	0.9999653829442635	0.1025980180280815	0.10096140381508774
IL17A	GOTERM_BP_DIRECT	GO:0045132~meiotic chromosome segregation	4	0.3034901365	0.002211641	SGO1, NUF2, SMC4, SMC2	1218	5	19478	12.793431855500822	0.9999653829442635	0.1025980180280815	0.10096140381508774
IL17A	GOTERM_BP_DIRECT	GO:0006310~DNA recombination	15	1.1380880121	0.002263308	INO80C, GEN1, BLM, FEN1, LIG1, MCM8, XRCC3, BRCA1, BRCA2, PSMC3IP, RECQL4, RAD51, EME1, EXO1, RAD54L	1218	95	19478	2.5250194451646357	0.9999727752729851	0.10395529875193123	0.10229703358537745
IL17A	GOTERM_BP_DIRECT	GO:0019885~antigen processing and presentation of endogenous peptide antigen via MHC class I	5	0.3793626707	0.00234825	ERAP2, ERAP1, TAP2, TAP1, TAPBP	1218	10	19478	7.9958949096880145	0.9999816584646651	0.10530609271732033	0.10362628007212056
IL17A	GOTERM_BP_DIRECT	GO:0002819~regulation of adaptive immune response	5	0.3793626707	0.00234825	IL4I1, DUSP10, IRF1, TNFSF4, AHR	1218	10	19478	7.9958949096880145	0.9999816584646651	0.10530609271732033	0.10362628007212056
IL17A	GOTERM_BP_DIRECT	GO:0071168~protein localization to chromatin	7	0.5311077389	0.002360818	MMS22L, MSH2, PARP1, MCM8, TONSL, ESCO2, EZH2	1218	23	19478	4.867066466766617	0.9999826996126576	0.10530609271732033	0.10362628007212056
IL17A	BIOCARTA	h_tnfr2Pathway:TNFR2 Signaling Pathway	8	0.6069802731	0.002382811	NFKBIA, TRAF3, TNFAIP3, TRAF2, TRAF1, TNFRSF1B, MAP3K14, NFKB1	186	18	1622	3.8757467144563926	0.3911699311025626	0.1233104606973999	0.12211905528003372
IL17A	GOTERM_BP_DIRECT	GO:0070301~cellular response to hydrogen peroxide	12	0.9104704097	0.002434672	ERN1, IL6, IL18RAP, PCNA, SPHK1, MYB, CDK1, PPIF, TNFAIP3, ECT2, EZH2, MAP3K5	1218	66	19478	2.9075981489774594	0.9999877282863273	0.10756611579629702	0.10585025190991504
IL17A	GOTERM_BP_DIRECT	GO:1900745~positive regulation of p38MAPK cascade	8	0.6069802731	0.002551408	MFHAS1, BMP2, PRMT1, GADD45A, SPHK1, ZC3H12A, XDH, MAP3K5	1218	31	19478	4.1269135017744585	0.9999928693253299	0.11166018648652488	0.10987901515649626
IL17A	GOTERM_BP_DIRECT	GO:0001933~negative regulation of protein phosphorylation	10	0.7587253414	0.002586986	LYN, CADM4, NTRK3, ZC3H12A, CHORDC1, DNAJC10, SLIT2, EPHB2, XDH, C9ORF72	1218	48	19478	3.331622879036672	0.9999939567981074	0.1121591262216027	0.11036999594775088

IL17A	GOTERM_BP_DIRECT	GO:0007155~cell adhesion	53	4.0212443095	0.002693555	SEMA5A, ACHE, BMP10, CNTNAP1, CLSTN3, LAMC2, CLDN1, CX3CL1, ICAM1, ROBO1, ADGRE2, ADGRE5, OLR1, ITGAV, CD34, CDON, MYBPC2, ITGA4, ITGA2, GP1BA, RGMB, ATP1B1, MTSS1, CLDN4, CDH13, ITGA6, CLDN16, CD47, TLR3, EPHA3, EPHA2, HEPACAM, NRXN3, NEDD9, THY1, COL19A1, PDZD2, FLRT2, PODXL, PDPN, ADAM23, CLCA2, ABL2, CCL2, HES1, OPCML, IL32, VCAM1, LAMB3, L1CAM, FERMT1, KITLG, NECTIN1	1218	559	19478	1.5162162082771546	0.999996319	0.11569818197513651	0.11385259769702483
IL17A	GOTERM_BP_DIRECT	GO:0071356~cellular response to tumor necrosis factor	16	1.2139605462	0.002904847	VCAM1, CXCL8, BRCA1, CLDN1, NFKB1, CTSK, ZC3H12A, ANKRD1, CCL2, CHI3L1, HAS2, HES1, CD58, TNFRSF21, NKX3-1, MAP3K5	1218	108	19478	2.369154047314967	0.9999986224411003	0.12362924119336127	0.12165714292901364
IL17A	UP_KW_BIOLOGICAL_PROCESS	KW-0226~DNA condensation	6	0.4552352048	0.003013455	NCAPG2, NCAPG, NCAPD3, SMC4, NCAPH, SMC2	824	15	11523	5.59368932	0.344607309	0.038627019	0.035613563
IL17A	GOTERM_BP_DIRECT	GO:0000077~DNA damage checkpoint signaling	9	0.6828528072	0.003414113	LYN, BRIP1, CHEK1, DOT1L, DONSON, E2F1, TOPBP1, CLSPN, ATR	1218	41	19478	3.510392887180103	0.9999998712212049	0.14268532874671308	0.1404092532288737
IL17A	GOTERM_BP_DIRECT	GO:0010212~response to ionizing radiation	9	0.6828528072	0.003414113	RRM1, VCAM1, RFWD3, RAD54L, AEN, BRCA1, TOPBP1, TICRR, RAD54B	1218	41	19478	3.510392887180103	0.9999998712212049	0.14268532874671308	0.1404092532288737
IL17A	GOTERM_BP_DIRECT	GO:0060707~trophoblast giant cell differentiation	5	0.3793626707	0.00350866	PLK4, LIF, PRDM1, E2F7, E2F8	1218	11	19478	7.268995372443649	0.9999999170720653	0.14404134727295573	0.14174364093577127
IL17A	GOTERM_BP_DIRECT	GO:0045063~T-helper 1 cell differentiation	5	0.3793626707	0.00350866	CRACR2A, IL4R, NFKBIZ, IL18R1, RELB	1218	11	19478	7.268995372443649	0.9999999170720653	0.14404134727295573	0.14174364093577127
IL17A	GOTERM_BP_DIRECT	GO:0046697~decidualization	7	0.5311077389	0.003721028	CYP27B1, PARP1, PARP2, LIF, STC1, JUNB, DCAF13	1218	25	19478	4.477701149425288	0.9999999691473453	0.1514197126453757	0.14900430873596468
IL17A	GOTERM_BP_DIRECT	GO:1903490~positive regulation of mitotic cytokinesis	4	0.3034901365	0.004217826	INCENP, CDCA8, BIRC5, AURKB	1218	6	19478	10.66119321291735	0.9999999969492435	0.16723500680238826	0.16456732184800657
IL17A	GOTERM_BP_DIRECT	GO:0006335~DNA replication-dependent chromatin assembly	4	0.3034901365	0.004217826	CHAF1B, CHAF1A, NASP, ASF1B	1218	6	19478	10.66119321291735	0.9999999969492435	0.16723500680238826	0.16456732184800657
IL17A	GOTERM_BP_DIRECT	GO:0051383~kinetochore organization	4	0.3034901365	0.004217826	NUF2, SMC4, NDC80, SMC2	1218	6	19478	10.66119321291735	0.9999999969492435	0.16723500680238826	0.16456732184800657
IL17A	UP_KW_BIOLOGICAL_PROCESS	KW-0090~Biological rhythms	21	1.5933232169	0.00458515	TOP2A, KLF10, SUV39H2, SUV39H1, BHLHE41, AHR, ADCY1, NPAS2, RELB, NFKB2, GPR176, MYBBP1A, NOCT, BHLHE40, NAMPT, TIMELESS, CDK1, ID3, DTL, EZH2, CDK5R1	824	149	11523	1.9709308008079753	0.4744958524062284	0.053875515004168546	0.049672461
IL17A	GOTERM_BP_DIRECT	GO:0032212~positive regulation of telomere maintenance via telomerase	7	0.5311077389	0.004585576	CCT6A, CCT2, PKIB, HSP90AA1, DKC1, CCT5, ATR	1218	26	19478	4.305481874447392	0.9999999994501693	0.17889191612662683	0.1760382834917119

IL17A	GOTERM_BP_DIRECT	GO:0007165~signal transduction	106	8.0424886191	0.00458895	CNTF, HHIP, ARHGAP42, GJA1, RGS1, PLAU, RASSF5, RPS6KA1, NAMPT, TNFSF10, TNFRSF8, MAP3K9, IL4R, VRK1, ANK2, MOB3B, ARHGAP45, HDGFL3, CD274, CHRNA5, CHRNA6, GPR85, STC1, ITPR2, ARHGAP18, RASGRP1, RAP1B, ARHGAP22, C3, RACGAP1, IRAK2, ABL2, FAM83D, PAG1, TNFSF18, LYN, HSPA8, TNFSF15, TNFRSF10B, TNFRSF10A, CDC7, OSGIN2, NFKB1, NFKB2, PTPRE, CDK6, GDNF, TNFSF4, CDK2, ULBP2, IL7R, CD320, NFKBIB, ARHGAP11A, CXCL6, CNTNAP1, CXCL8, PDE3B, CXCL1, CXCL3, CXCL2, CXCL5, RND1, TNFSF13B, DUSP10, SPX, TIMP1, CD34, GPR39, DUSP5, DUSP2, IL15, NFAM1, PLAUR, TRAF2, RGMB, TLR1, GPRC5A, TRAF4, TRAF3, TLR6, PPP1R12B, TNFRSF21, TLR3, HBEGF, TLR2, PKN3, NRXN3, NEDD9, AKAP5, LRP8, TYMP, DLL4, IL1RL1, RASD1, NCR3LG1, PDPN, CCL2, SH2B3, IL32, GABRQ, RANBP1, VEGFC, FAS, CCDC3, IL18R1	1218	1308	19478	1.2959707345977514	0.9999999994587463	0.17889191612662683	0.1760382834917119
IL17A	GOTERM_BP_DIRECT	GO:0071479~cellular response to ionizing radiation	9	0.6828528072	0.004639886	BARD1, RAD51AP1, BLM, RAD51, GADD45A, FIGNL1, BRCA1, BRCA2, ECT2	1218	43	19478	3.347118799404284	0.9999999995731212	0.17937025582575322	0.17650899285289146
IL17A	GOTERM_BP_DIRECT	GO:0000027~ribosomal large subunit assembly	5	0.3793626707	0.005004802	BOP1, MRTO4, NOP2, RRS1, MDN1	1218	12	19478	6.663245758073344	0.9999999999220982	0.19187833550070302	0.18881754722153682
IL17A	GOTERM_BP_DIRECT	GO:0009410~response to xenobiotic stimulus	28	2.124430956	0.005174507	ERRFI1, GATA6, AHR, HTR2A, ADCY1, TYMS, PFAS, LRP8, NPAS2, RAD54B, PNP, NFKBIZ, RAD54L, SLC19A1, LYN, TIGAR, HSP90AA1, ITGA2, VEGFC, CTPS1, SOD2, FOSL1, CENPF, RAD51, GAL, BCL2, CDK1, ATR	1218	255	19478	1.7559612350687401	0.9999999999646901	0.19675849881366506	0.19361986356636798
IL17A	GOTERM_BP_DIRECT	GO:0002040~sprouting angiogenesis	8	0.6069802731	0.005247436	ESM1, FLT1, VEGFC, CDH13, RSPO3, SEMA3E, E2F7, E2F8	1218	35	19478	3.6552662444288058	0.9999999999748698	0.19790939401965246	0.1947524000214946
IL17A	GOTERM_BP_DIRECT	GO:0042098~T cell proliferation	9	0.6828528072	0.005366897	GJA1, KITLG, EBI3, HES1, CTPS1, TNFRSF4, RASGRP1, CORO1A, TNFSF13B	1218	44	19478	3.2710479175996414	0.9999999999856042	0.2007825242192861	0.1975796988706706
IL17A	UP_KW_BIOLOGICAL_PROCESS	KW-0037~Angiogenesis	20	1.5174506828	0.005406323	ROBO4, FLT1, C1GALT1, PDE3B, TNFAIP2, NRXN3, VEGFC, SEMA3E, TYMP, EREG, DLL4, ARHGAP22, ESM1, ZC3H12A, KDR, COL8A2, MMP19, CD47, NAA15, EPHA2	824	141	11523	1.983577773187358	0.5318369640925809	0.058637816	0.054063235
IL17A	KEGG_PATHWAY	hsa05167:Kaposi sarcoma-associated herpesvirus infection	27	2.048558422	0.00556026	CSF2, CXCL8, PIK3R3, ITPR2, CXCL1, CXCL3, CXCL2, ICAM1, C3, E2F1, E2F2, E2F3, BID, IKBKE, LYN, IFNAR2, HLA-B, TRAF2, HLA-G, NFKB1, NFKBIA, IL6, CDK6, TRAF3, FAS, CYCS, TLR3	671	196	8534	1.7520149639587577	0.848112048	0.06712028	0.062155762699746535
IL17A	GOTERM_BP_DIRECT	GO:0043123~positive regulation of canonical NF-kappaB signal transduction	26	1.9726858877	0.005578403	TFRC, SLC20A1, DDX21, CX3CL1, GJA1, EDNRB, IRAK2, TIFA, NUP62, NAMPT, TNFSF10, TNFSF11, ECT2, IKBKE, APOL3, CD74, PARP1, TNFSF15, TNFRSF10B, TRAF2, IL1A, GPRC5B, TLR6, BIRC2, TLR3, BIRC3	1218	232	19478	1.7921833418266238	0.9999999999946325	0.20702568645849406	0.20372327197306003
IL17A	GOTERM_BP_DIRECT	GO:0045766~positive regulation of angiogenesis	20	1.5174506828	0.005662454	SEMA5A, FLT1, CXCL8, SPHK1, ERAP1, SERPINE1, GATA6, VASH2, VEGFC, BRCA1, ETS1, CX3CL1, C3, IL1A, ZC3H12A, KDR, CTSH, CHI3L1, CD34, TLR3	1218	161	19478	1.9865577415373945	0.9999999999963736	0.20847716700161623	0.2051515989140716
IL17A	UP_KW_BIOLOGICAL_PROCESS	KW-0346~Stress response	17	1.2898330804	0.00636937	HSPA8, HSP90AA1, HSPA4L, HSPE1, HSPA14, HSPA12A, HSPD1, MANF, HSP90B1, DNAJC3, HSPH1, RPS6KA1, ZC3H12A, CHORDC1, HYOU1, MAP3K9, MAP3K5	824	113	11523	2.1038212045708398	0.5912141044953123	0.064148655	0.05914415
IL17A	GOTERM_BP_DIRECT	GO:0043032~positive regulation of macrophage activation	6	0.4552352048	0.006650363	IL33, IL1RL1, IL4R, PLA2G4A, TLR6, HSPD1	1218	20	19478	4.7975369458128085	0.9999999999999639	0.2391553197941958	0.2353403825954955

IL17A	GOTERM_BP_DIRECT	GO:0036342~post-anal tail morphogenesis	6	0.4552352048	0.006650363	HES7, CHST11, DCHS1, PALB2, TP63, EPHA2	1218	20	19478	4.7975369458128085	0.9999999999999639	0.2391553197941958	0.2353403825954955
IL17A	GOTERM_BP_DIRECT	GO:0006405~RNA export from nucleus	6	0.4552352048	0.006650363	DDX39A, NUP188, NUP155, NUP62, XPO5, NUP153	1218	20	19478	4.7975369458128085	0.9999999999999639	0.2391553197941958	0.2353403825954955
IL17A	GOTERM_BP_DIRECT	GO:0051983~regulation of chromosome segregation	5	0.3793626707	0.006875352	CDCA2, KIF2C, MKI67, BUB1, AURKB	1218	13	19478	6.150688392067703	0.9999999999999873	0.24189766675235375	0.23803898442002477
IL17A	GOTERM_BP_DIRECT	GO:0046425~regulation of receptor signaling pathway via JAK-STAT	5	0.3793626707	0.006875352	IFNAR2, DOT1L, HES1, EPHB2, JAK3	1218	13	19478	6.150688392067703	0.9999999999999873	0.24189766675235375	0.23803898442002477
IL17A	GOTERM_BP_DIRECT	GO:0045892~negative regulation of DNA-templated transcription	52	3.9453717754	0.006981351	SUV39H2, CSF2, SUV39H1, GMNN, BHLHE41, BRCA1, AHR, ENO1, FOXM1, HHEX, SCML2, SCML1, BCL7A, MYB, ANKRD1, TRIM24, TNFRSF4, TP63, NKX3-1, KLF10, PARP1, SFMBT1, NRG1, EREG, ELF3, IRF1, SRSF2, TIMELESS, DEPDC1, BIRC5, GATA6, HDAC9, RELB, NPAT, RASD1, RBBP8, E2F1, HES1, PPARGC1B, HSPA8, HMGA1, PA2G4, BMP2, CENPF, MYBBP1A, TNFSF4, ZNF219, BCL3, BHLHE40, ID3, CDK5R1, EZH2	1218	573	19478	1.4512619033290637	0.9999999999999923	0.24189766675235375	0.23803898442002477
IL17A	GOTERM_BP_DIRECT	GO:0010032~meiotic chromosome condensation	4	0.3034901365	0.007039488	NCAPD3, SMC4, NCAPH, SMC2	1218	7	19478	9.138165611072015	0.9999999999999941	0.24189766675235375	0.23803898442002477
IL17A	GOTERM_BP_DIRECT	GO:0006312~mitotic recombination	4	0.3034901365	0.007039488	RAD51, MSH2, DMC1, RAD54B	1218	7	19478	9.138165611072015	0.9999999999999941	0.24189766675235375	0.23803898442002477
IL17A	GOTERM_BP_DIRECT	GO:0034136~negative regulation of toll-like receptor 2 signaling pathway	4	0.3034901365	0.007039488	LYN, MFHAS1, TNFAIP3, TLR6	1218	7	19478	9.138165611072015	0.9999999999999941	0.24189766675235375	0.23803898442002477
IL17A	GOTERM_BP_DIRECT	GO:0006284~base-excision repair	8	0.6069802731	0.007207731	POLQ, FEN1, NEIL3, LIG1, PARP2, HMGA1, DNA2, UNG	1218	37	19478	3.457684285270492	0.9999999999999973	0.24585781606153245	0.2419359625610898
IL17A	KEGG_PATHWAY	hsa01523:Antifolate resistance	8	0.6069802731	0.007347899	DHFR, ABCC3, IL6, SHMT1, GGH, TYMS, NFKB1, SLC19A1	671	30	8534	3.3915548931942374	0.9173184903510746	0.085641026	0.079306631
IL17A	BIOCARTA	h_bard1Pathway:BRCA1-dependent Ub-ligase activity	5	0.3793626707	0.007926527	BARD1, FANCD2, FANCA, BRCA1, FANCG	186	8	1622	5.450268817204301	0.808962719	0.2535408634954674	0.2510911933167672
IL17A	GOTERM_BP_DIRECT	GO:0034097~response to cytokine	9	0.6828528072	0.008073976	CD274, BCL2, LIFR, TIMP1, TYMS, OSMR, NFKB1, RELB, NFKB2	1218	47	19478	3.0622576249868985	1	0.2714143042458483	0.26708478096190935
IL17A	GOTERM_BP_DIRECT	GO:0022617~extracellular matrix disassembly	9	0.6828528072	0.008073976	ADAMTS4, MMP1, CTSK, MMP19, ADAM8, MMP8, MMP9, CTSS, MMP10	1218	47	19478	3.0622576249868985	1	0.2714143042458483	0.26708478096190935
IL17A	GOTERM_BP_DIRECT	GO:2001238~positive regulation of extrinsic apoptotic signaling pathway	8	0.6069802731	0.008369458	CYLD, TNFSF15, TNFSF10, G0S2, TNFSF11, TRAF2, TLR6, BID	1218	38	19478	3.366692593552848	1	0.2780905337245859	0.2736545131391969

IL17A	GOTERM_BP_DIRECT	GO:0043065~positive regulation of apoptotic process	33	2.5037936267	0.008392471	TOP2A, PDCD5, E2F1, TNFSF10, ANKRD1, TNFRSF8, PMAIP1, APBB2, MAP3K9, SLIT2, PHLDA2, ECT2, BID, NTSR1, MAP3K5, BARD1, WNT10B, GADD45A, TNFRSF10B, TNFRSF10A, MMP9, FOSL1, BMP2, IL6, GAL, MELK, MIR221, BCL2, ID3, FAS, ITGA6, EEF1E1, TLR3	1218	328	19478	1.6089300732908807	1	0.2780905337245859	0.2736545131391969
IL17A	BIOCARTA	h_LairPathway:Cells and Molecules involved in local acute inflammatory response	7	0.5311077389	0.008544836	C3, IL1A, IL6, VCAM1, CXCL8, ITGA4, ICAM1	186	17	1622	3.590765338393422	0.8321967011762748	0.2535408634954674	0.2510911933167672
IL17A	BIOCARTA	h_atrbrcaPathway:Role of BRCA1, BRCA2 and ATR in Cancer Susceptibility	8	0.6069802731	0.008573846	RAD51, FANCD2, CHEK1, FANCA, BRCA1, BRCA2, FANCG, ATR	186	22	1622	3.171065493646139	0.8332148793854868	0.2535408634954674	0.2510911933167672
IL17A	GOTERM_BP_DIRECT	GO:0051726~regulation of cell cycle	26	1.9726858877	0.008932118	INO80C, HEPACAM, CCNF, DOT1L, BRCA1, LIN9, FOXM1, FIGNL1, PCLAF, E2F2, JUNB, BARD1, PNPT1, DDIAS, GADD45A, MASTL, WDR12, BOP1, RBL1, CDK6, IRF1, EVI2B, KIF20B, DTL, BIRC2, BIRC3	1218	241	19478	1.7252553332106917	1	0.2938730077075843	0.2891852296152452
IL17A	GOTERM_BP_DIRECT	GO:0000731~DNA synthesis involved in DNA repair	7	0.5311077389	0.009557543	POLD3, POLQ, POLA1, RRM1, RFC3, POLD1, POLE	1218	30	19478	3.7314176245210726	1	0.30789890835641015	0.30298739311209577
IL17A	GOTERM_BP_DIRECT	GO:0002639~positive regulation of immunoglobulin production	7	0.5311077389	0.009557543	IL33, IL6, IL4R, TNFSF4, DNAJB9, EPHB2, TNFRSF4	1218	30	19478	3.7314176245210726	1	0.30789890835641015	0.30298739311209577
IL17A	GOTERM_BP_DIRECT	GO:0007129~homologous chromosome pairing at meiosis	7	0.5311077389	0.009557543	PSMC3IP, NDC1, CCNE2, FANCD2, CCNE1, DMC1, MND1	1218	30	19478	3.7314176245210726	1	0.30789890835641015	0.30298739311209577
IL17A	GOTERM_BP_DIRECT	GO:0050767~regulation of neurogenesis	8	0.6069802731	0.009663143	DLL4, HES7, ANXA2, CHD7, BHLHE40, BHLHE41, HES1, CX3CL1	1218	39	19478	3.280367142436108	1	0.30915393239680306	0.3042223973682703
IL17A	GOTERM_BP_DIRECT	GO:0007229~integrin-mediated signaling pathway	15	1.1380880121	0.00999397	SEMA7A, ADAMDEC1, ITGA4, RCC2, ITGA2, NEDD9, THY1, FERMT1, ADAMTS3, DIAPH3, ADAM23, ADAM8, ITGAV, ITGA6, CD47	1218	112	19478	2.141757565095004	1	0.3175481364895705	0.3124826995203469
IL17A	UP_KW_BIOLOGICAL_PROCESS	KW-0945~Host-virus interaction	67	5.0834597875	0.0100327839	SCARB1, TFRC, SUV39H1, JPT2, HTR2A, IPO7, CLDN1, CX3CL1, ICAM1, IFIH1, ZC3H12A, XPO5, RPS6KA1, NUP62, KDR, IL12A, ITGAV, KPNA2, IKBKE, TNFRSF4, MAP3K5, SUPT16H, HSP90AA1, CXADR, ANXA2, MMP1, ITGA2, DDX11, PLA2G4C, HLA-B, TAP2, TAP1, VRK1, PDIA4, CCNA2, PSMA3, RBL1, TRAF3, BIRC5, CCDC86, TNFRSF21, EPHA2, PCNA, CFH, SLC20A1, CUL2, LRP8, HSPD1, UNG, SYNGR2, G3BP1, E2F1, BUB1, LYN, CENPU, HSPA8, NUP153, PSMB9, SRPK1, NFKBIA, FAM111A, POLA1, KRT18, CDK1, ABCE1, ULBP2, NECTIN1	824	695	11523	1.3481193685828037	0.7562673173448637	0.09430817	0.086950795
IL17A	GOTERM_BP_DIRECT	GO:0007088~regulation of mitotic nuclear division	6	0.4552352048	0.010182889	CDCA2, NEK2, FBXO5, MKI67, PKMYT1, KIF20B	1218	22	19478	4.361397223466189	1	0.32134981654595357	0.3162237362647721
IL17A	GOTERM_BP_DIRECT	GO:0034475~U4 snRNA 3'-end processing	4	0.3034901365	0.010743515	EXOSC5, EXOSC9, EXOSC8, EXOSC2	1218	8	19478	7.995894909688013	1	0.3300607072837816	0.32479567336720483

IL17A	GOTERM_BP_DIRECT	GO:0006999~nuclear pore organization	4	0.3034901365	0.010743515	NDC1, NUP205, SEH1L, NUP35	1218	8	19478	7.995894909688013	1	0.3300607072837816	0.32479567336720483
IL17A	GOTERM_BP_DIRECT	GO:1902425~positive regulation of attachment of mitotic spindle microtubules to kinetochore	4	0.3034901365	0.010743515	INCENP, CDCA8, BIRC5, AURKB	1218	8	19478	7.995894909688013	1	0.3300607072837816	0.32479567336720483
IL17A	GOTERM_BP_DIRECT	GO:0110025~DNA strand resection involved in replication fork processing	4	0.3034901365	0.010743515	BARD1, EXO1, RBBP8, BRCA1	1218	8	19478	7.995894909688013	1	0.3300607072837816	0.32479567336720483
IL17A	GOTERM_BP_DIRECT	GO:0050919~negative chemotaxis	8	0.6069802731	0.0110968749	SEMA5A, SEMA7A, FLRT2, SEMA3A, ITGAV, SEMA3E, NRG1, SLIT2	1218	40	19478	3.1983579638752055	1	0.33637286406232636	0.33100714042779045
IL17A	GOTERM_BP_DIRECT	GO:0070092~regulation of glucagon secretion	3	0.2276176024	0.0112158057	IL6, PASK, SYT7	1218	3	19478	15.991789819376026	1	0.33637286406232636	0.33100714042779045
IL17A	GOTERM_BP_DIRECT	GO:0097421~liver regeneration	7	0.5311077389	0.0112390157	IL6, PCNA, PNPT1, TYMS, CLDN1, EZH2, PTPN3	1218	31	19478	3.6110493140526514	1	0.33637286406232636	0.33100714042779045
IL17A	GOTERM_BP_DIRECT	GO:0007131~reciprocal meiotic recombination	7	0.5311077389	0.0112390157	PSMC3IP, RAD51, DMC1, RAD54L, MND1, TRIP13, RAD54B	1218	31	19478	3.6110493140526514	1	0.33637286406232636	0.33100714042779045
IL17A	GOTERM_BP_DIRECT	GO:0009636~response to toxic substance	12	0.9104704097	0.0117586690	LYN, BRIP1, RAD51, GATA6, BCL2, CDK1, SDC1, AHR, TYMS, CLDN1, SLC19A1, TLR2	1218	81	19478	2.369154047314967	1	0.34651634273193244	0.34098881322941826
IL17A	GOTERM_BP_DIRECT	GO:0070266~necroptotic process	5	0.3793626707	0.0118767187	CYLD, MLKL, PPIF, PGAM5, BIRC2	1218	15	19478	5.330596606458675	1	0.34651634273193244	0.34098881322941826
IL17A	GOTERM_BP_DIRECT	GO:0007064~mitotic sister chromatid cohesion	5	0.3793626707	0.0118767187	CDC20, CDCA5, ESCO2, SMC1A, HASPIN	1218	15	19478	5.330596606458675	1	0.34651634273193244	0.34098881322941826
IL17A	GOTERM_BP_DIRECT	GO:0006287~base-excision repair, gap-filling	5	0.3793626707	0.0118767187	FEN1, PCNA, LIG1, POLD1, POLE	1218	15	19478	5.330596606458675	1	0.34651634273193244	0.34098881322941826
IL17A	KEGG_PATHWAY	hsa04217:Necroptosis	22	1.6691957511	0.0125376002	IFNAR2, IL33, HSP90AA1, MLKL, PARP1, PLA2G4C, PLA2G4A, TNFAIP3, TNFRSF10B, TRAF2, TNFRSF10A, IL1A, CYLD, TNFSF10, BCL2, FAS, PGAM5, BID, JAK3, BIRC2, TLR3, BIRC3	671	159	8534	1.7597690483555006	0.9859410823667328	0.141256962	0.13080896230819442
IL17A	GOTERM_BP_DIRECT	GO:0032467~positive regulation of cytokinesis	8	0.6069802731	0.0126786199	RACGAP1, KIF14, KIF23, CDC6, KIF20B, ECT2, CDC14A, AURKB	1218	41	19478	3.1203492330489806	1	0.365317503	0.3594900629594005
IL17A	GOTERM_BP_DIRECT	GO:0030574~collagen catabolic process	8	0.6069802731	0.0126786199	ADAMTS3, MMP1, CTSK, MMP19, MMP8, MMP9, CTSS, MMP10	1218	41	19478	3.1203492330489806	1	0.365317503	0.3594900629594005
IL17A	GOTERM_BP_DIRECT	GO:0051028~mRNA transport	13	0.9863429438	0.0129080391	NDC1, NUP205, NUP107, SEH1L, NUP188, NUP153, NUP85, NUP50, NUP62, NUP35, NUP88, SRSF7, NUP58	1218	93	19478	2.2354114801278318	1	0.36963205925292275	0.3637357944577694
IL17A	GOTERM_BP_DIRECT	GO:0042100~B cell proliferation	7	0.5311077389	0.0131190038	NFKBIZ, BCL2, CTPS1, IL7R, RASGRP1, TNFSF13B, HSPD1	1218	32	19478	3.4982040229885056	1	0.37336845785124473	0.36741259109526453
IL17A	KEGG_PATHWAY	hsa01524:Platinum drug resistance	13	0.9863429438	0.0132828884	TOP2A, PIK3R3, BRCA1, MSH2, BCL2, FAS, BIRC5, PMAIP1, CYCS, BID, BIRC2, BIRC3, MAP3K5	671	75	8534	2.2045106805762544	0.9891077148645921	0.14482633191154312	0.13411432511335208
IL17A	GOTERM_BP_DIRECT	GO:0006457~protein folding	20	1.5174506828	0.0141319880	CCT2, HSPA8, HSP90AA1, HSPA4L, HSPE1, HSPD1, PDIA4, TAPBP, HSP90B1, CLGN, CCT6A, HSPH1, GRPEL1, DNAJB11, ANP32E, PPIF, HYOU1, QSOX2, FKBP4, CCT5	1218	176	19478	1.8172488431109122	1	0.39622931717658616	0.3899087805369941

IL17A	GOTERM_BP_DIRECT	GO:0032733~positive regulation of interleukin-10 production	8	0.6069802731	0.0144161737	CD274, IL6, TSLP, HGF, TNFSF4, BCL3, HSPD1, TLR2	1218	42	19478	3.046055203690672	1	0.39622931717658616	0.3899087805369941
IL17A	KEGG_PATHWAY	hsa05144:Malaria	10	0.7587253414	0.0146014154	IL6, VCAM1, CXCL8, HGF, IL18, CCL2, SDC1, IL12A, ICAM1, TLR2	671	50	8534	2.543666169895678	0.9930683961338406	0.15024106116423014	0.13912855664024862
IL17A	KEGG_PATHWAY	hsa01232:Nucleotide metabolism	14	1.0622154779	0.0146685059	RRM1, RRM2, CTPS1, TYMS, TYMP, NME1, NME1-NME2, NT5E, UCK2, PNP, TK1, UPP1, DCTPP1, XDH	671	85	8534	2.0947839046199705	0.9932260944399205	0.15024106116423014	0.13912855664024862
IL17A	GOTERM_BP_DIRECT	GO:0048146~positive regulation of fibroblast proliferation	9	0.6828528072	0.0146940389	CCNA2, CD74, CCNB1, CDK6, SPHK1, LIF, E2F1, CDC6, EREG	1218	52	19478	2.767809776430466	1	0.39622931717658616	0.3899087805369941
IL17A	GOTERM_BP_DIRECT	GO:0042327~positive regulation of phosphorylation	6	0.4552352048	0.014822059	LYN, TNFSF11, ITGA6, AREG, PPARGC1B, EREG	1218	24	19478	3.9979474548440064	1	0.39622931717658616	0.3899087805369941
IL17A	GOTERM_BP_DIRECT	GO:2001243~negative regulation of intrinsic apoptotic signaling pathway	6	0.4552352048	0.014822059	HELLS, FIGNL1, PLAUR, BCL2, PPIF, MMP9	1218	24	19478	3.9979474548440064	1	0.39622931717658616	0.3899087805369941
IL17A	GOTERM_BP_DIRECT	GO:0006164~purine nucleotide biosynthetic process	5	0.3793626707	0.0150662123	PRPS2, SLC4A7, MTHFD1, PPAT, PFAS	1218	16	19478	4.997434318555008	1	0.39622931717658616	0.3899087805369941
IL17A	GOTERM_BP_DIRECT	GO:0000463~maturation of LSU-rRNA from tricistronic rRNA transcript (SSU-rRNA, 5.8S rRNA, LSU-rRNA)	5	0.3793626707	0.0150662123	DDX18, BOP1, PAK1IP1, WDR12, GTPBP4	1218	16	19478	4.997434318555008	1	0.39622931717658616	0.3899087805369941
IL17A	GOTERM_BP_DIRECT	GO:0008156~negative regulation of DNA replication	5	0.3793626707	0.0150662123	SLFN11, GMNN, CDC6, GTPBP4, ATR	1218	16	19478	4.997434318555008	1	0.39622931717658616	0.3899087805369941
IL17A	GOTERM_BP_DIRECT	GO:0009887~animal organ morphogenesis	13	0.9863429438	0.0151241920	CDX1, ITGA2, TMEM176B, GMNN, VEGFC, PALB2, FLI1, EREG, ETV7, BMP2, CCL2, E2F5, PHLDA2	1218	95	19478	2.1883501858093513	1	0.39622931717658616	0.3899087805369941
IL17A	GOTERM_BP_DIRECT	GO:0099171~presynaptic modulation of chemical synaptic transmission	7	0.5311077389	0.0152083807	CHRNA5, ADORA2B, NOG, CHRNA6, NRXN3, ADCY1, HTR2A	1218	33	19478	3.3921978404737025	1	0.39622931717658616	0.3899087805369941
IL17A	GOTERM_BP_DIRECT	GO:0002237~response to molecule of bacterial origin	4	0.3034901365	0.0153742783	CXCL8, TAP2, TNFAIP3, CXCL2	1218	9	19478	7.107462141944901	1	0.39622931717658616	0.3899087805369941
IL17A	GOTERM_BP_DIRECT	GO:1904894~positive regulation of receptor signaling pathway via STAT	4	0.3034901365	0.0153742783	IL6, TSLP, LIF, IL7R	1218	9	19478	7.107462141944901	1	0.39622931717658616	0.3899087805369941
IL17A	GOTERM_BP_DIRECT	GO:1904874~positive regulation of telomerase RNA localization to Cajal body	4	0.3034901365	0.0153742783	CCT6A, CCT2, DKC1, CCT5	1218	9	19478	7.107462141944901	1	0.39622931717658616	0.3899087805369941
IL17A	GOTERM_BP_DIRECT	GO:0034214~protein hexamerization	4	0.3034901365	0.0153742783	MAT2A, TWNK, LRRC8C, LRRC8D	1218	9	19478	7.107462141944901	1	0.39622931717658616	0.3899087805369941

IL17A	GOTERM_BP_DIRECT	GO:0150105~protein localization to cell-cell junction	4	0.3034901365	0.0153742783	DSP, HEPACAM, CGNL1, TJP2	1218	9	19478	7.107462141944901	1	0.39622931717658616	0.3899087805369941
IL17A	GOTERM_BP_DIRECT	GO:0061518~microglial cell proliferation	4	0.3034901365	0.0153742783	IL33, CSF1, IL34, CX3CL1	1218	9	19478	7.107462141944901	1	0.39622931717658616	0.3899087805369941
IL17A	GOTERM_BP_DIRECT	GO:0086014~atrial cardiac muscle cell action potential	4	0.3034901365	0.0153742783	GJC1, GJA1, NUP155, ANK2	1218	9	19478	7.107462141944901	1	0.39622931717658616	0.3899087805369941
IL17A	BIOCARTA	h_lymPathway:Adhesion and Diapedesis of Lymphocytes	6	0.4552352048	0.0154757173	IL1A, VCAM1, CXCL8, ITGA4, CD34, ICAM1	186	14	1622	3.7373271889400925	0.9609970106279843	0.3717599039986862	0.3681680208682641
IL17A	GOTERM_BP_DIRECT	GO:0001666~response to hypoxia	20	1.5174506828	0.015776023	ARNT2, EGLN3, VCAM1, TFRC, ITGA2, LIF, VEGFC, DIO3, ITPR2, PLOD2, SOD2, HSP90B1, IL1A, BMP2, PLAU, UCP3, MYB, TFAM, BIRC2, TLR2	1218	178	19478	1.796830316783823	1	0.4043368478586328	0.39788698221053215
IL17A	GOTERM_BP_DIRECT	GO:1901796~regulation of signal transduction by p53 class mediator	8	0.6069802731	0.016317119	BOP1, PAK1IP1, NOP2, CHEK1, TRIM24, RRS1, URB2, AURKB	1218	43	19478	2.975216710581586	1	0.41590721514115403	0.40927278230639536
IL17A	GOTERM_BP_DIRECT	GO:0002244~hematopoietic progenitor cell differentiation	11	0.834597876	0.0164113872	TOP2A, SLC8A3, LYN, AGPAT5, ANLN, KITLG, RRS1, GPATCH4, ESCO2, INHBA, SIPA1L3	1218	74	19478	2.377157946123463	1	0.4160241824928119	0.4093878838283437
IL17A	GOTERM_BP_DIRECT	GO:0060907~positive regulation of macrophage cytokine production	6	0.4552352048	0.0175973162	PANX1, CD74, SEMA7A, GPRC5B, HLA-G, TLR3	1218	25	19478	3.8380295566502465	1	0.4436627733618817	0.4365855918079306
IL17A	KEGG_PATHWAY	hsa05203:Viral carcinogenesis	26	1.9726858877	0.0178635322	PIK3R3, HDAC9, C3, CDC20, CHEK1, PMAIP1, JAK3, LYN, RANBP1, EGR2, HLA-B, TRAF2, TRAF1, HLA-G, NFKB1, NFKB2, NFKBIA, CCNA2, PKM, RBL1, CDK6, CCNE2, CCNE1, TRAF3, CDK2, CDK1	671	205	8534	1.6130565955436005	0.9977401430057301	0.17758452639877548	0.16444957622135126
IL17A	BIOCARTA	h_cellcyclePathway:Cyclins and Cell Cycle Regulation	8	0.6069802731	0.0179594156	CCNB1, CDK6, RBL1, CCNE1, CDK2, CDK1, E2F1, CDC25A	186	25	1622	2.7905376344086026	0.9769366336097914	0.3717599039986862	0.3681680208682641
IL17A	BIOCARTA	h_g2Pathway:Cell Cycle: G2/M Checkpoint	8	0.6069802731	0.0179594156	CCNB1, GADD45A, RPS6KA1, CHEK1, CDK1, BRCA1, CDC25A, ATR	186	25	1622	2.7905376344086026	0.9769366336097914	0.3717599039986862	0.3681680208682641
IL17A	GOTERM_BP_DIRECT	GO:0048661~positive regulation of smooth muscle cell proliferation	9	0.6828528072	0.018216292	IL6, ITGA2, MYB, IL18, RBPMS2, CDH13, CX3CL1, EREG, HBEGF	1218	54	19478	2.6652983032293376	1	0.456785819	0.4494993022589218
IL17A	GOTERM_BP_DIRECT	GO:0008584~male gonad development	14	1.0622154779	0.0183593727	RRM1, GATA6, FANCA, GFRA1, INHBA, ASPM, GJA1, KITLG, NASP, MSH2, TNFSF10, BCL2, TLR3, NKX3-1	1218	109	19478	2.0539913529473792	1	0.4577247114605713	0.4504232178955611
IL17A	GOTERM_BP_DIRECT	GO:0051279~regulation of release of sequestered calcium ion into cytosol	5	0.3793626707	0.0187470780	LYN, UBASH3B, ANK2, TMEM38B, CORO1A	1218	17	19478	4.703467593934126	1	0.4577247114605713	0.4504232178955611
IL17A	GOTERM_BP_DIRECT	GO:0042119~neutrophil activation	5	0.3793626707	0.0187470780	CXCL6, IL18RAP, CXCL8, IL15, IL18	1218	17	19478	4.703467593934126	1	0.4577247114605713	0.4504232178955611
IL17A	GOTERM_BP_DIRECT	GO:0048246~macrophage chemotaxis	5	0.3793626707	0.0187470780	EDNRB, NUP85, SAA1, CCL2, CX3CL1	1218	17	19478	4.703467593934126	1	0.4577247114605713	0.4504232178955611

IL17A	GOTERM_BP_DIRECT	GO:0000712~resolution of meiotic recombination intermediates	5	0.3793626707	0.0187470780	TOP2A, ANKLE1, FANCM, CENPX, EME1	1218	17	19478	4.703467593934126	1	0.4577247114605713	0.4504232178955611
IL17A	GOTERM_BP_DIRECT	GO:0034976~response to endoplasmic reticulum stress	13	0.9863429438	0.0189736653	CXCL8, FICD, TNFRSF10B, TRAF2, PDIA4, HSP90B1, ERN1, TMEM33, DNAJC10, DNAJB9, HYOU1, CFTR, MAP3K5	1218	98	19478	2.121359873998861	1	0.45941641068652145	0.45208793161973926
IL17A	GOTERM_BP_DIRECT	GO:0042127~regulation of cell population proliferation	18	1.3657056145	0.0190144321	EGLN3, CADM4, FLT1, TNFRSF9, CDCA7, PLA2G4A, PRDM1, FOXM1, PTGS1, GNL3, NFKBIA, CHST11, PLAU, CHEK1, KDR, JUNB, BIRC2, EZH2	1218	157	19478	1.8334536098647674	1	0.45941641068652145	0.45208793161973926
IL17A	GOTERM_BP_DIRECT	GO:0006986~response to unfolded protein	10	0.7587253414	0.0191854478	DNAJC3, HSPA8, HSP90AA1, HSPH1, FICD, HSPA4L, DNAJB9, HSPE1, HSPD1, MANF	1218	65	19478	2.460275356827081	1	0.46114659440363936	0.45379051594149894
IL17A	GOTERM_BP_DIRECT	GO:0007267~cell-cell signaling	24	1.8209408194	0.0193084310	TNFSF18, SEMA5A, KLF10, CXCL6, PTGIR, IL15, IL18, INHBA, ECE2, THY1, GDF5, AREG, CXCL5, CX3CL1, EREG, GJC1, PANX1, GJA1, BMP2, GJD3, ADGRE5, NAMPT, TNFSF10, FJX1	1218	232	19478	1.6543230847630372	1	0.46171036866480886	0.4543452970370452
IL17A	GOTERM_BP_DIRECT	GO:0098656~monoatomic anion transmembrane transport	7	0.5311077389	0.0200561361	PANX1, SLC4A7, ABCC3, LRRC8C, LRRC8D, LRRC8B, SLC19A1	1218	35	19478	3.198357963875205	1	0.47277596579375786	0.46523437892832603
IL17A	GOTERM_BP_DIRECT	GO:0055074~calcium ion homeostasis	7	0.5311077389	0.0200561361	CYP27B1, NT5E, GPRIN1, TRIM24, KDR, TNFSF11, TMTC2	1218	35	19478	3.198357963875205	1	0.47277596579375786	0.46523437892832603
IL17A	GOTERM_BP_DIRECT	GO:0071347~cellular response to interleukin-1	9	0.6828528072	0.0201871565	CXCL8, ZC3H12A, ANKRD1, CCL2, HAS2, HES1, CD47, NFKB1, NKX3-1	1218	55	19478	2.6168383340797132	1	0.47277596579375786	0.46523437892832603
IL17A	KEGG_PATHWAY	hsa05215:Prostate cancer	15	1.1380880121	0.0203027913	HSP90AA1, PIK3R3, MMP9, NFKB1, HSP90B1, NFKBIA, CCNE2, CCNE1, PLAU, CDK2, E2F1, BCL2, E2F2, E2F3, NKX3-1	671	98	8534	1.9466832932875089	0.9990248929894667	0.1960669567467791	0.18156496290456173
IL17A	GOTERM_BP_DIRECT	GO:0046330~positive regulation of JNK cascade	13	0.9863429438	0.020406608	GADD45A, SEMA3A, WNT7B, DKK1, IL1A, MFHAS1, SH3RF2, CRACR2A, TRAF4, CCDC88C, TNFSF11, TLR3, MAP3K5	1218	99	19478	2.0999319964837206	1	0.47277596579375786	0.46523437892832603
IL17A	GOTERM_BP_DIRECT	GO:0045943~positive regulation of transcription by RNA polymerase I	6	0.4552352048	0.0206953496	UTP15, MYBBP1A, BNC1, DDX21, LYAR, WDR43	1218	26	19478	3.6904130352406215	1	0.47277596579375786	0.46523437892832603
IL17A	GOTERM_BP_DIRECT	GO:0000462~maturation of SSU-rRNA from tricistronic rRNA transcript (SSU-rRNA, 5.8S rRNA, LSU-rRNA)	6	0.4552352048	0.0206953496	NOP14, UTP4, DHX37, TSR1, DCAF13, WDR43	1218	26	19478	3.6904130352406215	1	0.47277596579375786	0.46523437892832603
IL17A	GOTERM_BP_DIRECT	GO:0051292~nuclear pore complex assembly	4	0.3034901365	0.0209568618	NDC1, NUP205, NUP107, NUP153	1218	10	19478	6.396715927750411	1	0.47277596579375786	0.46523437892832603
IL17A	GOTERM_BP_DIRECT	GO:0050764~regulation of phagocytosis	4	0.3034901365	0.0209568618	SCARB1, SPHK1, ITGAV, SYT7	1218	10	19478	6.396715927750411	1	0.47277596579375786	0.46523437892832603
IL17A	GOTERM_BP_DIRECT	GO:0150077~regulation of neuroinflammatory response	4	0.3034901365	0.0209568618	IL6, SPHK1, TNFRSF1B, MMP9	1218	10	19478	6.396715927750411	1	0.47277596579375786	0.46523437892832603
IL17A	GOTERM_BP_DIRECT	GO:0010888~negative regulation of lipid storage	4	0.3034901365	0.0209568618	NFKBIA, IL6, CLSTN3, ITGAV	1218	10	19478	6.396715927750411	1	0.47277596579375786	0.46523437892832603

IL17A	GOTERM_BP_DIRECT	GO:2000279~negative regulation of DNA biosynthetic process	4	0.3034901365	0.0209568618	DNAJC2, CHEK1, ANKRD1, KCNK2	1218	10	19478	6.396715927750411	1	0.47277596579375786	0.46523437892832603
IL17A	GOTERM_BP_DIRECT	GO:0072719~cellular response to cisplatin	3	0.2276176024	0.021503714	RAD51, DDX11, TIMELESS	1218	4	19478	11.99384236453202	1	0.47277596579375786	0.46523437892832603
IL17A	GOTERM_BP_DIRECT	GO:0042148~DNA strand invasion	3	0.2276176024	0.021503714	RAD51, XRCC2, DMC1	1218	4	19478	11.99384236453202	1	0.47277596579375786	0.46523437892832603
IL17A	GOTERM_BP_DIRECT	GO:0043137~DNA replication, removal of RNA primer	3	0.2276176024	0.021503714	FEN1, RNASEH2A, DNA2	1218	4	19478	11.99384236453202	1	0.47277596579375786	0.46523437892832603
IL17A	GOTERM_BP_DIRECT	GO:0043111~replication fork arrest	3	0.2276176024	0.021503714	TIPIN, SLFN11, TIMELESS	1218	4	19478	11.99384236453202	1	0.47277596579375786	0.46523437892832603
IL17A	GOTERM_BP_DIRECT	GO:0045626~negative regulation of T-helper 1 cell differentiation	3	0.2276176024	0.021503714	IL4R, TNFSF4, JAK3	1218	4	19478	11.99384236453202	1	0.47277596579375786	0.46523437892832603
IL17A	GOTERM_BP_DIRECT	GO:0045003~double-strand break repair via synthesis-dependent strand annealing	3	0.2276176024	0.021503714	FANCM, XRCC3, RAD54L	1218	4	19478	11.99384236453202	1	0.47277596579375786	0.46523437892832603
IL17A	GOTERM_BP_DIRECT	GO:0009615~response to virus	14	1.0622154779	0.0225042779	IFNAR2, BATF3, ODC1, IFI44, ENO1, DCLK1, IFIH1, FOSL1, CDK6, OAS3, TNFSF4, BCL3, IL12A, CCT5	1218	112	19478	1.9989737274220032	1	0.49244030826067264	0.48458504143349235
IL17A	GOTERM_BP_DIRECT	GO:0010165~response to X-ray	5	0.3793626707	0.02293811	BLM, RAD51, MSH2, XRCC2, BRCA2	1218	18	19478	4.4421638387155635	1	0.49724248835203994	0.48931061852275537
IL17A	GOTERM_BP_DIRECT	GO:0043517~positive regulation of DNA damage response, signal transduction by p53 class mediator	5	0.3793626707	0.02293811	ANKRD1, PMAIP1, EEF1E1, HIC1, ATR	1218	18	19478	4.4421638387155635	1	0.49724248835203994	0.48931061852275537
IL17A	KEGG_PATHWAY	hsa04621:NOD-like receptor signaling pathway	24	1.8209408194	0.022978851	IFNAR2, HSP90AA1, CXCL8, IL18, TNFAIP3, ITPR2, CXCL1, TRAF2, CXCL3, CXCL2, NFKB1, PANX1, NFKBIA, IL6, TRAF3, OAS3, NAMPT, BCL2, CCL2, IKBKE, GBP4, BIRC2, BIRC3, NFKBIB	671	189	8534	1.6150261396163037	0.9996131521993963	0.21274442661573623	0.19700889210273795
IL17A	KEGG_PATHWAY	hsa05160:Hepatitis C	21	1.5933232169	0.0237306678	IFNAR2, SCARB1, PIK3R3, TRAF2, CLDN1, NFKB1, NFKBIA, CLDN4, CDK6, TRAF3, OAS3, CDK2, E2F1, FAS, E2F2, CYCS, E2F3, CLDN16, BID, IKBKE, TLR3	671	159	8534	1.6797795461575233	0.9997017773316823	0.21274442661573623	0.19700889210273795
IL17A	KEGG_PATHWAY	hsa03420:Nucleotide excision repair	11	0.834597876	0.0239180124	RFC5, POLD3, RFC3, RFC4, PCNA, LIG1, RFC2, POLD1, POLE2, POLE3, POLE	671	63	8534	2.2206609419724175	0.9997205083260249	0.21274442661573623	0.19700889210273795
IL17A	GOTERM_BP_DIRECT	GO:0070536~protein K63-linked deubiquitination	6	0.4552352048	0.0241285356	OTUD4, USP13, CYLD, PSMD14, STAMBPL1, TNFAIP3	1218	27	19478	3.5537310709724506	1	0.5206152415049254	0.5123105362082313
IL17A	GOTERM_BP_DIRECT	GO:0045786~negative regulation of cell cycle	9	0.6828528072	0.0245744576	BARD1, CDT1, BMP2, CDK6, SUV39H1, GMNN, BRCA1, ETS1, IPO7	1218	57	19478	2.5250194451646357	1	0.5277819860036272	0.5193629588503037
IL17A	BIOCARTA	h_atmPathway:ATM Signaling Pathway	7	0.5311077389	0.025396424	NFKBIA, RAD51, GADD45A, CHEK1, RBBP8, BRCA1, NFKB1	186	21	1622	2.906810035842294	0.9952551324088772	0.47791452796228934	0.4732969962911562
IL17A	GOTERM_BP_DIRECT	GO:1904646~cellular response to amyloid-beta	8	0.6069802731	0.025696737	GJA1, VCAM1, ITGA4, PARP1, CACNA2D1, TLR6, EPHB2, ICAM1	1218	47	19478	2.7220067777661323	1	0.5452454848949347	0.5365478850065482

IL17A	GOTERM_BP_DIRECT	GO:0030890~positive regulation of B cell proliferation	8	0.6069802731	0.025696737	CD74, ATAD5, TFRC, BCL2, EPHB2, TNFRSF4, TNFSF13B, CD320	1218	47	19478	2.7220067777661323	1	0.5452454848949347	0.5365478850065482
IL17A	GOTERM_BP_DIRECT	GO:0001934~positive regulation of protein phosphorylation	15	1.1380880121	0.0257688651	LYN, CD74, TFRC, IL34, NTRK3, PLAUR, PIK3R3, RASGRP1, MMP9, C3, BMP2, KDR, FAS, ADTRP, NKX3-1	1218	126	19478	1.9037845023066697	1	0.5452454848949347	0.5365478850065482
IL17A	GOTERM_BP_DIRECT	GO:2001235~positive regulation of apoptotic signaling pathway	7	0.5311077389	0.0258577293	CTSK, FAS, CTSH, EEF1E1, CTSS, TP63, NKX3-1	1218	37	19478	3.0254737496116806	1	0.5452454848949347	0.5365478850065482
IL17A	UP_KW_BIOLOGICAL_PROCESS	KW-0811~Translocation	13	0.9863429438	0.025933846	NDC1, NUP205, NUP107, SEH1L, NUP188, NUP155, NUP153, NUP85, NUP50, NUP62, NUP35, NUP88, NUP58	824	90	11523	2.019943365695793	0.9747429016922354	0.22854201540537258	0.21071249647303855
IL17A	GOTERM_BP_DIRECT	GO:0045944~positive regulation of transcription by RNA polymerase II	93	7.0561456752	0.0259852319	TOP2A, EHF, CHD7, BRCA1, AHR, FOXM1, ETS1, CX3CL1, SPIB, IL4I1, HHEX, ZMIZ2, SIX2, ZC3H12A, NAMPT, RPS6KA1, NFKBIZ, MYB, TNFSF11, SPX, MYBL2, JUNB, MYBL1, TP63, NKX3-1, CDON, KLF10, EIF5A, ZNF485, TEAD4, RBM14, PARP1, ANXA2, HGF, NOG, IL18, CKAP2, PPRC1, ETV4, IL1A, GAL, PKM, ELF3, IRF1, NCL, GPRIN1, CDH13, ITGA6, NCOA7, TLR3, TLR2, UHRF1, DOT1L, GATA6, DDX21, RELB, NPAT, EXOSC9, E2F1, E2F2, APBB2, E2F3, HES1, PPARGC1B, E2F7, E2F8, ARNT2, IL33, EGR2, CDX1, LUM, PCGF5, LIF, EAF2, HMGA1, MICAL2, INHBA, POU2F2, FLI1, NFKB1, NFKB2, NFKBIA, FOSL1, BMP2, IL6, NR6A1, MYBBP1A, GDNF, NFIB, ZNF219, BCL3, CEBPZ, NFE2L1	1218	1205	19478	1.234221122989187	1	0.54545471	0.5367537724775469
IL17A	GOTERM_BP_DIRECT	GO:2000134~negative regulation of G1/S transition of mitotic cell cycle	9	0.6828528072	0.0270007198	CACNB4, SLFN11, RBL1, BTN2A2, BCL2, CCL2, INHBA, E2F7, EZH2	1218	58	19478	2.4814846271445554	1	0.5595272406621756	0.5506018222942083
IL17A	GOTERM_BP_DIRECT	GO:0007259~cell surface receptor signaling pathway via JAK-STAT	9	0.6828528072	0.0270007198	SOCS2, IFNAR2, IL15RA, IL6, CNTF, CSF2, TSLP, CCL2, JAK3	1218	58	19478	2.4814846271445554	1	0.5595272406621756	0.5506018222942083
IL17A	GOTERM_BP_DIRECT	GO:1990918~double-strand break repair involved in meiotic recombination	4	0.3034901365	0.027499938	BRIP1, RAD51, FANCD2, DMC1	1218	11	19478	5.815196297954919	1	0.5595272406621756	0.5506018222942083
IL17A	GOTERM_BP_DIRECT	GO:0032392~DNA geometric change	4	0.3034901365	0.027499938	RECQL4, BLM, RECQL, DNA2	1218	11	19478	5.815196297954919	1	0.5595272406621756	0.5506018222942083
IL17A	GOTERM_BP_DIRECT	GO:0150078~positive regulation of neuroinflammatory response	4	0.3034901365	0.027499938	IL33, IL6, IL18, MMP8	1218	11	19478	5.815196297954919	1	0.5595272406621756	0.5506018222942083
IL17A	GOTERM_BP_DIRECT	GO:0072711~cellular response to hydroxyurea	4	0.3034901365	0.027499938	BLM, RAD51, DDX11, TIMELESS	1218	11	19478	5.815196297954919	1	0.5595272406621756	0.5506018222942083
IL17A	GOTERM_BP_DIRECT	GO:0033089~positive regulation of T cell differentiation in thymus	4	0.3034901365	0.027499938	VNN1, ADAM8, IL7R, RASGRP1	1218	11	19478	5.815196297954919	1	0.5595272406621756	0.5506018222942083

IL17A	GOTERM_BP_DIRECT	GO:0042730~fibrinolysis	5	0.3793626707	0.027653861	SERPINB2, ANXA2, PLAU, SERPINE1, GP1BA	1218	19	19478	4.208365741941059	1	0.5602020127928455	0.5512658306530157
IL17A	GOTERM_BP_DIRECT	GO:0045216~cell-cell junction organization	6	0.4552352048	0.0279076701	GJA1, CXADR, HEG1, XIRP2, CLDN1, TJP2	1218	28	19478	3.4268121041520057	1	0.5628855734470447	0.5539065839158783
IL17A	GOTERM_BP_DIRECT	GO:0050714~positive regulation of protein secretion	8	0.6069802731	0.0285181407	IL1A, ACHE, ANKRD1, VEGFC, ADAM8, FRMD4A, KCNN4, ABCG1	1218	48	19478	2.6652983032293376	1	0.5713792131864945	0.5622647355456667
IL17A	GOTERM_BP_DIRECT	GO:0051607~defense response to virus	24	1.8209408194	0.0285751190	IFNAR2, IL33, CXADR, MLKL, DDX21, IFIH1, DNAJC3, IL6, EXOSC5, SLFN11, TRAF3, IRF1, OAS3, G3BP1, ZC3H12A, BCL2, PMAIP1, POLR3G, IKBKE, POLR3K, MAP3K14, TLR3, APOBEC3B, TLR2	1218	241	19478	1.5925433845021768	1	0.5713792131864945	0.5622647355456667
IL17A	GOTERM_BP_DIRECT	GO:0030593~neutrophil chemotaxis	9	0.6828528072	0.0295882184	CXCL6, CXADR, CXCL8, SAA1, CXCL1, CXCL3, CXCL2, CXCL5, CX3CL1	1218	59	19478	2.439425565667529	1	0.5890976187688824	0.5797005021944273
IL17A	KEGG_PATHWAY	hsa05146:Amoebiasis	15	1.1380880121	0.0299087362	CXCL8, CSF2, LAMB3, PIK3R3, CXCL1, LAMC2, SERPINB9, ADCY1, CXCL3, CXCL2, NFKB1, IL6, COL4A4, IL12A, TLR2	671	103	8534	1.8521841042929694	0.999965114	0.2592090478986016	0.24003678104219617
IL17A	GOTERM_BP_DIRECT	GO:0071456~cellular response to hypoxia	16	1.2139605462	0.0303940894	SUV39H2, EGLN3, TIGAR, SUV39H1, GATA6, STC1, INHBA, SLC8A3, CCNA2, BRIP1, BCL2, PMAIP1, ADAM8, HYOU1, KCNK2, NKX3-1	1218	141	19478	1.8146711851774215	1	0.6025563295327607	0.5929445234570064
IL17A	GOTERM_BP_DIRECT	GO:0007098~centrosome cycle	8	0.6069802731	0.031541799	HAUS8, GADD45A, XRCC2, NUP62, CDK1, PCLAF, HAUS6, BRCA1	1218	49	19478	2.61090446	1	0.6200101948384653	0.6101199696998478
IL17A	GOTERM_BP_DIRECT	GO:0008630~intrinsic apoptotic signaling pathway in response to DNA damage	8	0.6069802731	0.031541799	BCL2, E2F1, BRCA1, SOD2, TNFRSF1B, IKBKE, HIC1, EPHA2	1218	49	19478	2.61090446	1	0.6200101948384653	0.6101199696998478
IL17A	GOTERM_BP_DIRECT	GO:0030183~B cell differentiation	10	0.7587253414	0.0321505571	HHEX, VCAM1, MSH2, ITGA4, NFAM1, PIK3R3, DNAJB9, HDAC9, JAK3, EZH2	1218	71	19478	2.252364763292398	1	0.626861731	0.6168622119703662
IL17A	GOTERM_BP_DIRECT	GO:0016579~protein deubiquitination	15	1.1380880121	0.032570751	OTUD4, USP13, USP37, USP49, PSMD14, USP31, SHMT1, TNFAIP3, UCHL3, CYLD, STAMBPL1, OTUD6B, ZC3H12A, USP1, CDK1	1218	130	19478	1.8452065176203107	1	0.626861731	0.6168622119703662
IL17A	GOTERM_BP_DIRECT	GO:0051591~response to cAMP	7	0.5311077389	0.032677442	FOSL1, PNPT1, GATA6, MMP19, SDC1, AREG, BIRC2	1218	39	19478	2.870321249631594	1	0.626861731	0.6168622119703662
IL17A	GOTERM_BP_DIRECT	GO:0045190~isotype switching	5	0.3793626707	0.0329047353	MSH2, ATAD5, EXO1, NFKBIZ, UNG	1218	20	19478	3.9979474548440073	1	0.626861731	0.6168622119703662
IL17A	GOTERM_BP_DIRECT	GO:0045893~positive regulation of DNA-templated transcription	58	4.4006069802	0.033710218	BMP10, WWC1, CITED4, BRCA1, AHR, FOXM1, BRCA2, ETS1, HHEX, RPS6KA1, NUP62, MYB, ANKRD1, TRIM24, MYBL1, TP63, NKX3-1, MAP3K5, RGMB, ETV4, WDR77, CCNA2, DNAJC2, IRF1, TFAM, BIRC2, INO80C, CD274, BLM, CDCA4, DOT1L, GATA6, NPAS2, PSMC3IP, NPAT, NUP85, E2F1, HIVEP3, E2F5, ARNT2, CD74, EGR2, ATAD2, HMGA1, INHBA, HNRNPAB, FLI1, NFKB1, BMP2, IL6, NFIB, BAMBI, BCL3, CDK2, NAA15, CNOT9, NFKBIB, PICALM	1218	714	19478	1.2990529545151392	1	0.626861731	0.6168622119703662
IL17A	KEGG_PATHWAY	hsa04934:Cushing syndrome	20	1.5174506828	0.034162479	SCARB1, WNT10B, WNT7B, ITPR2, AHR, ADCY1, CACNA1G, RAP1B, RASD1, NCEH1, CDK6, CCNE2, STAR, CCNE1, CDK2, E2F1, ORAI1, E2F2, E2F3, KCNK2	671	155	8534	1.6410749483197924	0.9999921009180964	0.2886729493849564	0.2673213998742348
IL17A	GOTERM_BP_DIRECT	GO:0006508~proteolysis	51	3.8694992412	0.034330621	OTUD4, CPM, ADAMDEC1, USP37, USP31, TNFAIP3, ECE2, PRSS22, CTSS, ADAMTS4, ADAMTS3, PLAU, OTUD6B, CTSK, CTSH, OLR1, ADAMTS6, USP49, MMP1, HGF, VASH2, MMP8, MMP9, MMP10, ESPL1, MMP19, ADAM8, LAP3, THOP1, DLD, CFB, USP13, CFH, SHMT1, LRP8, C2, STAMBPL1, NRIP2, ADAM23, CLCA2, USP1, RHBDL2, CPA4, GGT5, ERAP2, ERAP1, PRSS36, FAM111B, CYLD, FAM111A, CAPN12	1218	615	19478	1.3261484240458168	1	0.626861731	0.6168622119703662

IL17A	GOTERM_BP_DIRECT	GO:0038135~ERBB2-ERBB4 signaling pathway	3	0.2276176024	0.034365096	NRG1, EREG, HBEGF	1218	5	19478	9.595073891625617	1	0.626861731	0.6168622119703662
IL17A	GOTERM_BP_DIRECT	GO:0002841~negative regulation of T cell mediated immune response to tumor cell	3	0.2276176024	0.034365096	CD274, IL4I1, AHR	1218	5	19478	9.595073891625617	1	0.626861731	0.6168622119703662
IL17A	GOTERM_BP_DIRECT	GO:0006595~polyamine metabolic process	3	0.2276176024	0.034365096	AMD1, ODC1, SRM	1218	5	19478	9.595073891625617	1	0.626861731	0.6168622119703662
IL17A	GOTERM_BP_DIRECT	GO:0033591~response to L-ascorbic acid	3	0.2276176024	0.034365096	IL1A, ITGA2, SOD2	1218	5	19478	9.595073891625617	1	0.626861731	0.6168622119703662
IL17A	GOTERM_BP_DIRECT	GO:0032201~telomere maintenance via semi-conservative replication	3	0.2276176024	0.034365096	BLM, FEN1, DNA2	1218	5	19478	9.595073891625617	1	0.626861731	0.6168622119703662
IL17A	GOTERM_BP_DIRECT	GO:0043009~chordate embryonic development	3	0.2276176024	0.034365096	CENPU, CHD7, BRCA1	1218	5	19478	9.595073891625617	1	0.626861731	0.6168622119703662
IL17A	GOTERM_BP_DIRECT	GO:0060676~ureteric bud formation	3	0.2276176024	0.034365096	GDNF, NOG, HS2ST1	1218	5	19478	9.595073891625617	1	0.626861731	0.6168622119703662
IL17A	GOTERM_BP_DIRECT	GO:0009113~purine nucleobase biosynthetic process	3	0.2276176024	0.034365096	SHMT1, PPAT, PAICS	1218	5	19478	9.595073891625617	1	0.626861731	0.6168622119703662
IL17A	GOTERM_BP_DIRECT	GO:0051664~nuclear pore localization	3	0.2276176024	0.034365096	NDC1, LMNB2, LMNB1	1218	5	19478	9.595073891625617	1	0.626861731	0.6168622119703662
IL17A	GOTERM_BP_DIRECT	GO:0002158~osteoclast proliferation	3	0.2276176024	0.034365096	CSF1, TNFSF11, JUNB	1218	5	19478	9.595073891625617	1	0.626861731	0.6168622119703662
IL17A	GOTERM_BP_DIRECT	GO:0006297~nucleotide-excision repair, DNA gap filling	3	0.2276176024	0.034365096	POLD3, POLD1, POLE	1218	5	19478	9.595073891625617	1	0.626861731	0.6168622119703662
IL17A	GOTERM_BP_DIRECT	GO:0046718~symbiont entry into host cell	13	0.9863429438	0.034901206	SCARB1, CXADR, TFRC, ITGA2, NUP153, HTR2A, CLDN1, ICAM1, CDK1, ITGAV, TNFRSF4, EPHA2, NECTIN1	1218	107	19478	1.9429277350643772	1	0.626861731	0.6168622119703662
IL17A	GOTERM_BP_DIRECT	GO:0043589~skin morphogenesis	4	0.3034901365	0.034998316	ERRFI1, ITGA2, ITGA6, TP63	1218	12	19478	5.330596606458675	1	0.626861731	0.6168622119703662
IL17A	GOTERM_BP_DIRECT	GO:0045842~positive regulation of mitotic metaphase/anaphase transition	4	0.3034901365	0.034998316	CDC20, ESPL1, UBE2C, DLGAP5	1218	12	19478	5.330596606458675	1	0.626861731	0.6168622119703662
IL17A	GOTERM_BP_DIRECT	GO:0090656~t-circle formation	4	0.3034901365	0.034998316	BLM, EXO1, XRCC3, DNA2	1218	12	19478	5.330596606458675	1	0.626861731	0.6168622119703662
IL17A	GOTERM_BP_DIRECT	GO:0015810~aspartate transmembrane transport	4	0.3034901365	0.034998316	LRRC8C, LRRC8D, LRRC8B, SLC25A13	1218	12	19478	5.330596606458675	1	0.626861731	0.6168622119703662
IL17A	GOTERM_BP_DIRECT	GO:0010389~regulation of G2/M transition of mitotic cell cycle	4	0.3034901365	0.034998316	CENPF, CDK6, CDK2, KIF14	1218	12	19478	5.330596606458675	1	0.626861731	0.6168622119703662
IL17A	GOTERM_BP_DIRECT	GO:0000723~telomere maintenance	9	0.6828528072	0.035263851	INO80C, RECQL4, DCLRE1B, BLM, PARP1, CCNE2, CCNE1, DNA2, ATR	1218	61	19478	2.3594443995800694	1	0.6291884748126908	0.6191518403793778

IL17A	UP_KW_BIOLOGICAL_PROCESS	KW-0177~Collagen degradation	5	0.3793626707	0.035333317	MMP1, MMP19, MMP8, MMP9, MMP10	824	18	11523	3.8845064724919096	0.99350141	0.2767776507812233	0.2551850680961633
IL17A	UP_KW_BIOLOGICAL_PROCESS	KW-0891~Chondrogenesis	5	0.3793626707	0.035333317	BMP2, MGP, NOG, CHRDL2, GDF5	824	18	11523	3.8845064724919096	0.99350141	0.2767776507812233	0.2551850680961633
IL17A	KEGG_PATHWAY	hsa04215:Apoptosis - multiple species	7	0.5311077389	0.035777561	BCL2, PMAIP1, BIRC5, CYCS, BID, BIRC2, BIRC3	671	32	8534	2.782134873323398	0.9999955135265696	0.29494672181328363	0.27313113588034843
IL17A	BIOCARTA	h_p53Pathway:p53 Signaling Pathway	6	0.4552352048	0.036017196	PCNA, CCNE1, GADD45A, CDK2, BCL2, E2F1	186	17	1622	3.0777988614800758	0.9995142513086334	0.6212966342877055	0.6152937682559403
IL17A	GOTERM_BP_DIRECT	GO:0006298~mismatch repair	6	0.4552352048	0.036538796	POLD3, RNASEH2A, PCNA, MSH2, LIG1, EXO1	1218	30	19478	3.198357963875205	1	0.6493068714489412	0.6389493141117517
IL17A	GOTERM_BP_DIRECT	GO:0043524~negative regulation of neuron apoptotic process	17	1.2898330804	0.036671352	CNTF, XRCC2, KIF14, PLA2G3, SEMA3E, SOD2, GDF5, CORO1A, IL27RA, GDNF, MSH2, KDR, BCL2, CCL2, BIRC5, ADAM8, SNCB	1218	157	19478	1.7315950759833916	1	0.6493068714489412	0.6389493141117517
IL17A	GOTERM_BP_DIRECT	GO:0007162~negative regulation of cell adhesion	8	0.6069802731	0.03821568	SEMA5A, ADAMDEC1, PODXL, PDE3B, PLXNA1, CDH13, EPHB2, RND1	1218	51	19478	2.50851605	1	0.6715247695786485	0.6608127986907806
IL17A	GOTERM_BP_DIRECT	GO:0038145~macrophage colony-stimulating factor signaling pathway	8	0.6069802731	0.03821568	FLT1, CSF1, NTRK3, KDR, EPHB2, EPHB1, EPHA3, EPHA2	1218	51	19478	2.50851605	1	0.6715247695786485	0.6608127986907806
IL17A	KEGG_PATHWAY	hsa05219:Bladder cancer	8	0.6069802731	0.0384765387	CXCL8, MMP1, E2F1, E2F2, E2F3, MMP9, TYMP, HBEGF	671	41	8534	2.4816255316055393	0.9999982604323945	0.30964452589934505	0.286741824
IL17A	GOTERM_BP_DIRECT	GO:0090200~positive regulation of release of cytochrome c from mitochondria	5	0.3793626707	0.038697133	PLAUR, TNFSF10, PMAIP1, BID, MMP9	1218	21	19478	3.807569005	1	0.6723445688460039	0.6616195207549058
IL17A	GOTERM_BP_DIRECT	GO:0044818~mitotic G2/M transition checkpoint	5	0.3793626707	0.038697133	BARD1, CHEK1, RBBP8, BRCA1, ATR	1218	21	19478	3.807569005	1	0.6723445688460039	0.6616195207549058
IL17A	GOTERM_BP_DIRECT	GO:0060236~regulation of mitotic spindle organization	5	0.3793626707	0.038697133	TPX2, STIL, NUP62, SASS6, TACC3	1218	21	19478	3.807569005	1	0.6723445688460039	0.6616195207549058
IL17A	GOTERM_BP_DIRECT	GO:0048013~ephrin receptor signaling pathway	11	0.834597876	0.0388578101	LYN, FLT1, NTRK3, ARHGEF28, KDR, EPHB2, MMP9, EPHB1, EPHA3, EPHA2, CDK5R1	1218	85	19478	2.069525741331015	1	0.6726170940225189	0.6618876986878204
IL17A	KEGG_PATHWAY	hsa04514:Cell adhesion molecules	20	1.5174506828	0.040437707	CD274, CNTNAP1, VCAM1, ITGA4, HLA-B, NRXN3, PDCD1LG2, L1CAM, CLDN1, HLA-G, ICAM1, CLDN4, SLITRK2, SDC1, ITGAV, ITGA6, CD58, CLDN16, CD34, NECTIN1	671	158	8534	1.6099152974023279	0.9999991275659248	0.31785918778766414	0.2943488928329553
IL17A	GOTERM_BP_DIRECT	GO:0051276~chromosome organization	6	0.4552352048	0.041404138	CENPW, PTTG1, HAT1, RAD54L, CDCA8, TOPBP1	1218	31	19478	3.095185126330844	1	0.7087593949886485	0.6974534680153439
IL17A	GOTERM_BP_DIRECT	GO:0034113~heterotypic cell-cell adhesion	6	0.4552352048	0.041404138	VCAM1, ITGA4, ITGAV, CD47, CD58, THY1	1218	31	19478	3.095185126330844	1	0.7087593949886485	0.6974534680153439
IL17A	GOTERM_BP_DIRECT	GO:0070372~regulation of ERK1 and ERK2 cascade	6	0.4552352048	0.041404138	LYN, IL1A, FAM83D, RASGRP1, EPHB1, EPHA2	1218	31	19478	3.095185126330844	1	0.7087593949886485	0.6974534680153439
IL17A	GOTERM_BP_DIRECT	GO:0050927~positive regulation of positive chemotaxis	4	0.3034901365	0.0434352051	NTRK3, ITGA2, KDR, CDH13	1218	13	19478	4.920550713654162	1	0.7327124247925078	0.7210244059447722

IL17A	GOTERM_BP_DIRECT	GO:1903238~positive regulation of leukocyte tethering or rolling	4	0.3034901365	0.0434352051	ITGA4, GP1BA, CHST2, FUT4	1218	13	19478	4.920550713654162	1	0.7327124247925078	0.7210244059447722
IL17A	GOTERM_BP_DIRECT	GO:0001706~endoderm formation	4	0.3034901365	0.0434352051	DUSP5, DUSP2, NOG, DKK1	1218	13	19478	4.920550713654162	1	0.7327124247925078	0.7210244059447722
IL17A	GOTERM_BP_DIRECT	GO:0016446~somatic hypermutation of immunoglobulin genes	4	0.3034901365	0.0434352051	POLQ, MSH2, EXO1, UNG	1218	13	19478	4.920550713654162	1	0.7327124247925078	0.7210244059447722
IL17A	UP_KW_BIOLOGICAL_PROCESS	KW-0228~DNA excision	3	0.2276176024	0.044072249	POLD3, EXO1, POLD1	824	5	11523	8.390533980582525	0.9981823520798924	0.31070935244974124	0.286469616
IL17A	UP_KW_BIOLOGICAL_PROCESS	KW-0617~Plasminogen activation	3	0.2276176024	0.044072249	SERPINB2, PLAU, ENO1	824	5	11523	8.390533980582525	0.9981823520798924	0.31070935244974124	0.286469616
IL17A	GOTERM_BP_DIRECT	GO:0001541~ovarian follicle development	7	0.5311077389	0.044925475	KITLG, DMC1, BCL2, KDR, MMP19, INHBA, FANCG	1218	42	19478	2.6652983032293376	1	0.7514784782899043	0.739491109
IL17A	GOTERM_BP_DIRECT	GO:0010744~positive regulation of macrophage derived foam cell differentiation	5	0.3793626707	0.0450336315	CSF2, CSF1, IL18, PLA2G3, NFKB1	1218	22	19478	3.6344976862218243	1	0.7514784782899043	0.739491109
IL17A	GOTERM_BP_DIRECT	GO:0031589~cell-substrate adhesion	5	0.3793626707	0.0450336315	ITGA2, ITGAV, ITGA6, CORO1A, EPHB1	1218	22	19478	3.6344976862218243	1	0.7514784782899043	0.739491109
IL17A	KEGG_PATHWAY	hsa04010:MAPK signaling pathway	33	2.5037936267	0.0461083189	FLT1, CSF1, RASGRP1, AREG, CACNA1G, RELB, RASGRP3, RAP1B, DUSP10, RPS6KA1, KDR, MAP3K9, MAP3K5, HSPA8, DUSP5, DUSP2, GADD45A, HGF, CACNA2D1, PLA2G4C, VEGFC, PLA2G4A, TRAF2, NFKB1, EREG, NFKB2, IL1A, KITLG, CACNB4, GDNF, FAS, MAP3K14, EPHA2	671	300	8534	1.399016393442623	0.9999998823254016	0.3541957224729257	0.3279978140059933
IL17A	BIOCARTA	h_g1Pathway:Cell Cycle: G1/S Check Point	8	0.6069802731	0.0465618591	DHFR, CDK6, CCNE1, CDK2, CDK1, E2F1, CDC25A, ATR	186	30	1622	2.325448028673835	0.9999507002838884	0.6976333909172518	0.6908929716813362
IL17A	GOTERM_BP_DIRECT	GO:0051085~chaperone cofactor-dependent protein refolding	6	0.4552352048	0.046642126	HSPA8, CD74, HSPH1, SDF2L1, HSPE1, HSPA14	1218	32	19478	2.998460591133005	1	0.7700100408877105	0.7577270611451602
IL17A	GOTERM_BP_DIRECT	GO:0007026~negative regulation of microtubule depolymerization	6	0.4552352048	0.046642126	TPX2, DIAPH3, NAV3, CCDC88C, CKAP2, HDGFL3	1218	32	19478	2.998460591133005	1	0.7700100408877105	0.7577270611451602
IL17A	GOTERM_BP_DIRECT	GO:0010575~positive regulation of vascular endothelial growth factor production	6	0.4552352048	0.046642126	C3, IL1A, IL6, ADORA2B, BRCA1, SULF1	1218	32	19478	2.998460591133005	1	0.7700100408877105	0.7577270611451602

IL17A	GOTERM_BP_DIRECT	GO:0006338~chromatin remodeling	55	4.172989378	0.047154988	SUV39H2, CHD7, BUB1B, HR, BRCA1, BRCA2, CDC14A, DUSP10, BCL7A, UBASH3B, DPF1, RPS6KA1, NFKBIZ, CHEK1, PIM3, NEK2, RIOK1, TP63, DUSP5, DUSP2, USP49, PPTC7, PRMT1, PRMT3, VRK1, BAZ1A, PASK, HASPIN, MASTL, CDC25A, ERN1, PKM, SLFN11, MELK, BMP2K, PGAM5, ALPK2, HDGFL3, INO80C, DDX21, PKMYT1, NUAK2, NSD2, BUB1, BARD1, CDC7, DCLK1, SRPK1, PTPRE, MYBBP1A, POLE3, CDK2, CDK1, PTPN3, ATR	1218	686	19478	1.2821405831861248	1	0.7757162668059349	0.7633422629810505
IL17A	BIOCARTA	h_caspasePathway:Caspase Cascade in Apoptosis	7	0.5311077389	0.047182935	PARP1, ARHGDIB, CYCS, LMNB2, BIRC2, LMNB1, BIRC3	186	24	1622	2.5434587813620073	0.9999569491626292	0.6976333909172518	0.6908929716813362
IL17A	GOTERM_BP_DIRECT	GO:0032757~positive regulation of interleukin-8 production	9	0.6828528072	0.0487197341	TLR1, CD74, IL6, NLRP10, SERPINE1, CHI3L1, CD58, TLR3, TLR2	1218	65	19478	2.214247821144373	1	0.7847457212937057	0.7722276821956815
IL17A	GOTERM_BP_DIRECT	GO:0042270~protection from natural killer cell mediated cytotoxicity	3	0.2276176024	0.0494387308	HLA-B, SERPINB9, HLA-G	1218	6	19478	7.995894909688013	1	0.7847457212937057	0.7722276821956815
IL17A	GOTERM_BP_DIRECT	GO:0031536~positive regulation of exit from mitosis	3	0.2276176024	0.0494387308	UBE2C, CDCA5, BIRC5	1218	6	19478	7.995894909688013	1	0.7847457212937057	0.7722276821956815
IL17A	GOTERM_BP_DIRECT	GO:0002830~positive regulation of type 2 immune response	3	0.2276176024	0.0494387308	IL33, CD74, TNFSF4	1218	6	19478	7.995894909688013	1	0.7847457212937057	0.7722276821956815
IL17A	GOTERM_BP_DIRECT	GO:0060770~negative regulation of epithelial cell proliferation involved in prostate gland development	3	0.2276176024	0.0494387308	EAF2, NKX3-1, WDR77	1218	6	19478	7.995894909688013	1	0.7847457212937057	0.7722276821956815
IL17A	GOTERM_BP_DIRECT	GO:0002693~positive regulation of cellular extravasation	3	0.2276176024	0.0494387308	ADAM8, THY1, ICAM1	1218	6	19478	7.995894909688013	1	0.7847457212937057	0.7722276821956815
IL17A	GOTERM_BP_DIRECT	GO:0006189~'de novo' IMP biosynthetic process	3	0.2276176024	0.0494387308	PPAT, PAICS, PFAS	1218	6	19478	7.995894909688013	1	0.7847457212937057	0.7722276821956815
IL17A	GOTERM_BP_DIRECT	GO:0044770~cell cycle phase transition	3	0.2276176024	0.0494387308	TIPIN, TIMELESS, CDC7	1218	6	19478	7.995894909688013	1	0.7847457212937057	0.7722276821956815
IL17A	GOTERM_BP_DIRECT	GO:0035655~interleukin-18-mediated signaling pathway	3	0.2276176024	0.0494387308	IL18RAP, IL18, IL18R1	1218	6	19478	7.995894909688013	1	0.7847457212937057	0.7722276821956815
IL17A	GOTERM_BP_DIRECT	GO:0032728~positive regulation of interferon-beta production	7	0.5311077389	0.049564668	IFIH1, HSP90AA1, IRF1, OAS3, POLR3G, TLR3, TLR2	1218	43	19478	2.6033146217588876	1	0.7847457212937057	0.7722276821956815
IL17A	GOTERM_BP_DIRECT	GO:0060391~positive regulation of SMAD protein signal transduction	7	0.5311077389	0.049564668	BMP10, BMP2, BMPER, PARP1, TTK, INHBA, GDF5	1218	43	19478	2.6033146217588876	1	0.7847457212937057	0.7722276821956815

IL17A	GOTERM_BP_DIRECT	GO:0006259~DNA metabolic process	5	0.3793626707	0.051913199	NT5E, AEN, TOPBP1, KPNA2, NME1	1218	23	19478	3.4764760476904404	1	0.8081384102014151	0.7952472176265272
IL17A	GOTERM_BP_DIRECT	GO:0051924~regulation of calcium ion transport	5	0.3793626707	0.051913199	RGS4, CACNA2D1, BCL2, ORAI1, ANK2	1218	23	19478	3.4764760476904404	1	0.8081384102014151	0.7952472176265272
IL17A	GOTERM_BP_DIRECT	GO:0035924~cellular response to vascular endothelial growth factor stimulus	6	0.4552352048	0.052255295	ERN1, DLL4, FLT1, VCAM1, SPHK1, KDR	1218	33	19478	2.9075981489774594	1	0.8081384102014151	0.7952472176265272
IL17A	GOTERM_BP_DIRECT	GO:0051216~cartilage development	9	0.6828528072	0.0525397278	ERRFI1, BMP2, COL11A2, NOG, CHI3L1, TIMP1, TYMS, CHRDL2, SULF1	1218	66	19478	2.1806986117330944	1	0.8081384102014151	0.7952472176265272
IL17A	GOTERM_BP_DIRECT	GO:0034383~low-density lipoprotein particle clearance	4	0.3034901365	0.052784207	SCARB1, NCEH1, DGAT2, CES3	1218	14	19478	4.5690828055360075	1	0.8081384102014151	0.7952472176265272
IL17A	GOTERM_BP_DIRECT	GO:0030278~regulation of ossification	4	0.3034901365	0.052784207	PTGER4, EGR2, CSF1, SIX2	1218	14	19478	4.5690828055360075	1	0.8081384102014151	0.7952472176265272
IL17A	GOTERM_BP_DIRECT	GO:0003222~ventricular trabecula myocardium morphogenesis	4	0.3034901365	0.052784207	DLL4, HEG1, CHD7, NRG1	1218	14	19478	4.5690828055360075	1	0.8081384102014151	0.7952472176265272
IL17A	GOTERM_BP_DIRECT	GO:0051058~negative regulation of small GTPase mediated signal transduction	4	0.3034901365	0.052784207	ARHGAP22, SLIT2, CGNL1, ARHGAP45	1218	14	19478	4.5690828055360075	1	0.8081384102014151	0.7952472176265272
IL17A	GOTERM_BP_DIRECT	GO:0010745~negative regulation of macrophage derived foam cell differentiation	4	0.3034901365	0.052784207	ABCA1, NFKBIA, ITGAV, ABCG1	1218	14	19478	4.5690828055360075	1	0.8081384102014151	0.7952472176265272
IL17A	GOTERM_BP_DIRECT	GO:0009314~response to radiation	4	0.3034901365	0.052784207	BCL2, SMC1A, CDC25A, FANCG	1218	14	19478	4.5690828055360075	1	0.8081384102014151	0.7952472176265272
IL17A	GOTERM_BP_DIRECT	GO:0032729~positive regulation of type II interferon production	10	0.7587253414	0.0537463052	TNFSF4, HMSD, EBI3, BCL3, IL18, IL12A, IL18R1, TLR3, HSPD1, IL27RA	1218	78	19478	2.0502294640225673	1	0.820161546	0.8070785637674265
IL17A	GOTERM_BP_DIRECT	GO:0061077~chaperone-mediated protein folding	7	0.5311077389	0.054487177	FKBP11, CCT6A, CCT2, CHORDC1, FKBP4, CCT5, PDIA4	1218	44	19478	2.544148380355277	1	0.8287410245509589	0.8155211849698486
IL17A	GOTERM_BP_DIRECT	GO:0032496~response to lipopolysaccharide	15	1.1380880121	0.0550980057	PTGER4, PTGIR, VCAM1, DIO2, SOD2, CLDN1, C2, NFKB2, CYP27B1, DUSP10, NOCT, E2F1, BDKRB1, IL12A, TLR2	1218	140	19478	1.7134060520760028	1	0.8352929691711902	0.821968615
IL17A	GOTERM_BP_DIRECT	GO:0007173~epidermal growth factor receptor signaling pathway	12	0.9104704097	0.05667761	ERRFI1, FLT1, NTRK3, KDR, ABL2, EPHB2, AREG, EPHB1, EPHA3, EPHA2, EREG, HBEGF	1218	103	19478	1.8631211440049738	1	0.8564411565107297	0.8427794523542749
IL17A	GOTERM_BP_DIRECT	GO:0031175~neuron projection development	14	1.0622154779	0.05692594	LYN, PRMT1, GFRA1, VRK1, L1CAM, AREG, MANF, IL6, GDNF, GPRIN1, RAB13, MICALL2, HDGFL3, CDK5R1	1218	128	19478	1.7491020114942528	1	0.8574007665589057	0.8437237549776686

IL17A	GOTERM_BP_DIRECT	GO:0006611~protein export from nucleus	6	0.4552352048	0.058244569	EGR2, NXT1, ANKLE1, CSE1L, XPO5, CHORDC1	1218	34	19478	2.822080556360475	1	0.8687992162755572	0.8549403798874583
IL17A	GOTERM_BP_DIRECT	GO:0035987~endodermal cell differentiation	6	0.4552352048	0.058244569	LAMB3, ITGA4, ITGAV, INHBA, MMP8, MMP9	1218	34	19478	2.822080556360475	1	0.8687992162755572	0.8549403798874583
IL17A	GOTERM_BP_DIRECT	GO:0042771~intrinsic apoptotic signaling pathway in response to DNA damage by p53 class mediator	6	0.4552352048	0.058244569	MSH2, ATAD5, BCL3, AEN, BRCA2, TP63	1218	34	19478	2.822080556360475	1	0.8687992162755572	0.8549403798874583
IL17A	GOTERM_BP_DIRECT	GO:0048012~hepatocyte growth factor receptor signaling pathway	8	0.6069802731	0.058734555	FLT1, NTRK3, HGF, KDR, EPHB2, EPHB1, EPHA3, EPHA2	1218	56	19478	2.2845414027680038	1	0.8733000005698949	0.8593693689591658
IL17A	GOTERM_BP_DIRECT	GO:0042531~positive regulation of tyrosine phosphorylation of STAT protein	5	0.3793626707	0.0593314269	TNFSF18, CNTF, IL18, HES1, IL12A	1218	24	19478	3.331622879036672	1	0.8791230724519843	0.8650995528655547
IL17A	GOTERM_BP_DIRECT	GO:0043406~positive regulation of MAP kinase activity	7	0.5311077389	0.059694712	FLT1, IL34, NTRK3, TNFSF11, RASGRP1, EZH2, ROBO1	1218	45	19478	2.4876117496807155	1	0.8791230724519843	0.8650995528655547
IL17A	GOTERM_BP_DIRECT	GO:0001755~neural crest cell migration	7	0.5311077389	0.059694712	SEMA5A, SEMA7A, KITLG, EDNRB, GDNF, SEMA3A, SEMA3E	1218	45	19478	2.4876117496807155	1	0.8791230724519843	0.8650995528655547
IL17A	GOTERM_BP_DIRECT	GO:0001819~positive regulation of cytokine production	9	0.6828528072	0.060742671	PTGER4, IL33, IL1A, IL15, NFAM1, TNFSF4, SAA1, IL18, EREG	1218	68	19478	2.1165604172703563	1	0.8889124591188727	0.874732782
IL17A	GOTERM_BP_DIRECT	GO:0002931~response to ischemia	9	0.6828528072	0.060742671	PANX1, TIGAR, CSF1, MYB, BCL2, PPIF, HYOU1, CX3CL1, MAP3K5	1218	68	19478	2.1165604172703563	1	0.8889124591188727	0.874732782
IL17A	GOTERM_BP_DIRECT	GO:0097028~dendritic cell differentiation	4	0.3034901365	0.06301108	LYN, BATF3, CSF2, TMEM176B	1218	15	19478	4.264477285	1	0.8954136835957378	0.8811303008438335
IL17A	GOTERM_BP_DIRECT	GO:0010884~positive regulation of lipid storage	4	0.3034901365	0.06301108	C3, ZC3H12A, IKBKE, NFKB1	1218	15	19478	4.264477285	1	0.8954136835957378	0.8811303008438335
IL17A	GOTERM_BP_DIRECT	GO:0032736~positive regulation of interleukin-13 production	4	0.3034901365	0.06301108	IL33, TSLP, TNFSF4, IL18	1218	15	19478	4.264477285	1	0.8954136835957378	0.8811303008438335
IL17A	GOTERM_BP_DIRECT	GO:0048731~system development	4	0.3034901365	0.06301108	GDNF, WNT7B, GATA6, EPHB2	1218	15	19478	4.264477285	1	0.8954136835957378	0.8811303008438335
IL17A	UP_KW_BIOLOGICAL_PROCESS	KW-0053~Apoptosis	53	4.0212443095	0.063481524	PDCD5, BUB1B, TNFAIP3, RASSF5, ZC3H12A, TNFSF10, PIM3, MAP3K9, TP63, MAP3K5, PARP1, CKAP2, AEN, TRAF2, TRAF1, TNFRSF1B, ERN1, MELK, TRAF4, TRAF3, PTRH2, PPIF, BIRC5, CHI3L1, CD47, BIRC2, TNFRSF21, EPHA2, BIRC3, MTFP1, CDCA7, DRAM1, NUAK2, E2F1, PMAIP1, BID, BUB1, RELT, EGLN3, TIGAR, DDIAS, EAF2, G0S2, TNFRSF10B, TNFRSF10A, SULF1, NFKB1, TPX2, BCL2, CDK1, CYCS, FAS, XAF1	824	589	11523	1.2583426739413521	0.9998971299549324	0.4244169957976482	0.39130645002620046

IL17A	GOTERM_BP_DIRECT	GO:0050680~negative regulation of epithelial cell proliferation	8	0.6069802731	0.063516969	WNT10B, DUSP10, CDK6, TRIM24, GDF5, MTSS1, EREG, NKX3-1	1218	57	19478	2.244461729035232	1	0.8954136835957378	0.8811303008438335
IL17A	GOTERM_BP_DIRECT	GO:0006401~RNA catabolic process	6	0.4552352048	0.064609308	EXOSC5, RNASEH2A, PNPT1, EXOSC9, EXOSC8, EXOSC2	1218	35	19478	2.7414496833216044	1	0.8954136835957378	0.8811303008438335
IL17A	GOTERM_BP_DIRECT	GO:0045931~positive regulation of mitotic cell cycle	6	0.4552352048	0.064609308	CCNB1, CCDC57, SPHK1, BIRC5, BRCA2, NKX3-1	1218	35	19478	2.7414496833216044	1	0.8954136835957378	0.8811303008438335
IL17A	GOTERM_BP_DIRECT	GO:0032689~negative regulation of type II interferon production	7	0.5311077389	0.065188108	IL33, IL1RL1, CD274, TNFSF4, ZC3H12A, INHBA, PDCD1LG2	1218	46	19478	2.4335332333833084	1	0.8954136835957378	0.8811303008438335
IL17A	KEGG_PATHWAY	hsa05143:African trypanosomiasis	7	0.5311077389	0.06609022	IL6, VCAM1, IL18, FAS, IL12A, THOP1, ICAM1	671	37	8534	2.4061707012526683	0.9999999999081531	0.4906857374493819	0.454392414
IL17A	UP_KW_BIOLOGICAL_PROCESS	KW-0469~Meiosis	14	1.0622154779	0.066221092	MND1, OSGIN2, SMC1A, PSMC3IP, RAD51AP1, SGO2, EXO1, DMC1, CDK2, RBBP8, TESMIN, NEK2, BUB3, TRIP13	824	115	11523	1.7024271844660193	0.9999317396637455	0.4244169957976482	0.39130645002620046
IL17A	GOTERM_BP_DIRECT	GO:0000480~endonucleolytic cleavage in 5'-ETS of tricistronic rRNA transcript (SSU-rRNA, 5.8S rRNA, LSU-rRNA)	3	0.2276176024	0.066398428	RCL1, NOP14, UTP20	1218	7	19478	6.853624208304011	1	0.8954136835957378	0.8811303008438335
IL17A	GOTERM_BP_DIRECT	GO:0097294~'de novo' XMP biosynthetic process	3	0.2276176024	0.066398428	PPAT, PAICS, PFAS	1218	7	19478	6.853624208304011	1	0.8954136835957378	0.8811303008438335
IL17A	GOTERM_BP_DIRECT	GO:1903438~positive regulation of mitotic cytokinetic process	3	0.2276176024	0.066398428	NUP62, KIF20B, ECT2	1218	7	19478	6.853624208304011	1	0.8954136835957378	0.8811303008438335
IL17A	GOTERM_BP_DIRECT	GO:0040036~regulation of fibroblast growth factor receptor signaling pathway	3	0.2276176024	0.066398428	HHIP, NOG, SULF1	1218	7	19478	6.853624208304011	1	0.8954136835957378	0.8811303008438335
IL17A	GOTERM_BP_DIRECT	GO:2001199~negative regulation of dendritic cell differentiation	3	0.2276176024	0.066398428	TMEM176B, TMEM176A, HLA-G	1218	7	19478	6.853624208304011	1	0.8954136835957378	0.8811303008438335
IL17A	GOTERM_BP_DIRECT	GO:0098534~centriole assembly	3	0.2276176024	0.066398428	RBM14, NUP62, CEP85	1218	7	19478	6.853624208304011	1	0.8954136835957378	0.8811303008438335
IL17A	GOTERM_BP_DIRECT	GO:0071035~nuclear polyadenylation-dependent rRNA catabolic process	3	0.2276176024	0.066398428	EXOSC9, EXOSC8, EXOSC2	1218	7	19478	6.853624208304011	1	0.8954136835957378	0.8811303008438335
IL17A	GOTERM_BP_DIRECT	GO:0140361~cyclic-GMP-AMP transmembrane import across plasma membrane	3	0.2276176024	0.066398428	LRRC8C, LRRC8B, SLC19A1	1218	7	19478	6.853624208304011	1	0.8954136835957378	0.8811303008438335

IL17A	GOTERM_BP_DIRECT	GO:0006269~DNA replication, synthesis of primer	3	0.2276176024	0.066398428	POLA1, POLA2, PRIM1	1218	7	19478	6.853624208304011	1	0.8954136835957378	0.8811303008438335
IL17A	GOTERM_BP_DIRECT	GO:0032466~negative regulation of cytokinesis	3	0.2276176024	0.066398428	E2F7, AURKB, E2F8	1218	7	19478	6.853624208304011	1	0.8954136835957378	0.8811303008438335
IL17A	GOTERM_BP_DIRECT	GO:0002043~blood vessel endothelial cell proliferation involved in sprouting angiogenesis	3	0.2276176024	0.066398428	SEMA5A, BMPER, EPHA2	1218	7	19478	6.853624208304011	1	0.8954136835957378	0.8811303008438335
IL17A	GOTERM_BP_DIRECT	GO:0035330~regulation of hippo signaling	3	0.2276176024	0.066398428	NUAK2, WWC1, MOB3B	1218	7	19478	6.853624208304011	1	0.8954136835957378	0.8811303008438335
IL17A	GOTERM_BP_DIRECT	GO:0071038~TRAMP-dependent tRNA surveillance pathway	3	0.2276176024	0.066398428	EXOSC9, EXOSC8, EXOSC2	1218	7	19478	6.853624208304011	1	0.8954136835957378	0.8811303008438335
IL17A	GOTERM_BP_DIRECT	GO:0021960~anterior commissure morphogenesis	3	0.2276176024	0.066398428	DRAXIN, NFIB, FBXO45	1218	7	19478	6.853624208304011	1	0.8954136835957378	0.8811303008438335
IL17A	GOTERM_BP_DIRECT	GO:0044772~mitotic cell cycle phase transition	3	0.2276176024	0.066398428	CCNB1, CKS2, CKS1B	1218	7	19478	6.853624208304011	1	0.8954136835957378	0.8811303008438335
IL17A	GOTERM_BP_DIRECT	GO:0045630~positive regulation of T-helper 2 cell differentiation	3	0.2276176024	0.066398428	IL4R, TNFSF4, IL18	1218	7	19478	6.853624208304011	1	0.8954136835957378	0.8811303008438335
IL17A	GOTERM_BP_DIRECT	GO:0044725~epigenetic programming in the zygotic pronuclei	3	0.2276176024	0.066398428	SUV39H2, SUV39H1, DCAF13	1218	7	19478	6.853624208304011	1	0.8954136835957378	0.8811303008438335
IL17A	GOTERM_BP_DIRECT	GO:0090594~inflammatory response to wounding	3	0.2276176024	0.066398428	IL6, NFKBIZ, TLR3	1218	7	19478	6.853624208304011	1	0.8954136835957378	0.8811303008438335
IL17A	GOTERM_BP_DIRECT	GO:1901331~positive regulation of odontoblast differentiation	3	0.2276176024	0.066398428	BMP2, SERPINE1, IPO7	1218	7	19478	6.853624208304011	1	0.8954136835957378	0.8811303008438335
IL17A	KEGG_PATHWAY	hsa04625:C-type lectin receptor signaling pathway	14	1.0622154779	0.066779716	EGR2, PIK3R3, ITPR2, NFKB1, RELB, NFKB2, NFKBIA, CYLD, IL6, IRF1, BCL3, IL12A, IKBKE, MAP3K14	671	105	8534	1.695777446597119	0.9999999999284435	0.4906857374493819	0.454392414
IL17A	GOTERM_BP_DIRECT	GO:0038063~collagen-activated tyrosine kinase receptor signaling pathway	8	0.6069802731	0.068529003	FLT1, UBASH3B, NTRK3, KDR, EPHB2, EPHB1, EPHA3, EPHA2	1218	58	19478	2.205764113017383	1	0.9214667921229438	0.9067678176420001
IL17A	GOTERM_BP_DIRECT	GO:0070059~intrinsic apoptotic signaling pathway in response to endoplasmic reticulum stress	6	0.4552352048	0.071347357	ERN1, DNAJC10, BCL2, TNFRSF10B, TRAF2, MAP3K5	1218	36	19478	2.665298303229338	1	0.9565907173206941	0.9413314560398724

IL17A	GOTERM_BP_DIRECT	GO:0030308~negative regulation of cell growth	13	0.9863429438	0.071908875	BMP10, EAF2, BRCA1, ENO1, INHBA, OSGIN2, RERG, DCBLD2, CYP27B1, GJA1, BCL2, BDKRB1, SLIT2	1218	120	19478	1.7324438970990697	1	0.9613408416492392	0.946005808
IL17A	BIOCARTA	h_ephA4Pathway:Eph Kinases and ephrins support platelet aggregation	4	0.3034901365	0.073005601	LYN, RAP1B, L1CAM, EPHB1	186	9	1622	3.8757467144563926	0.9999998580815239	0.9396390169260113	0.9305603790813156
IL17A	GOTERM_BP_DIRECT	GO:0007019~microtubule depolymerization	4	0.3034901365	0.07407528	KIF18A, KIF18B, KIF24, KIF2C	1218	16	19478	3.9979474548440064	1	0.9790177281949416	0.9634007176568028
IL17A	GOTERM_BP_DIRECT	GO:0044027~negative regulation of gene expression via chromosomal CpG island methylation	4	0.3034901365	0.07407528	HELLS, DNMT1, UHRF1, BRCA1	1218	16	19478	3.9979474548440064	1	0.9790177281949416	0.9634007176568028
IL17A	GOTERM_BP_DIRECT	GO:0002687~positive regulation of leukocyte migration	4	0.3034901365	0.07407528	TNFSF18, KITLG, ITGA2, BDKRB1	1218	16	19478	3.9979474548440064	1	0.9790177281949416	0.9634007176568028
IL17A	GOTERM_BP_DIRECT	GO:0090162~establishment of epithelial cell polarity	4	0.3034901365	0.07407528	FERMT1, HES1, FRMD4A, SIPA1L3	1218	16	19478	3.9979474548440064	1	0.9790177281949416	0.9634007176568028
IL17A	GOTERM_BP_DIRECT	GO:0007623~circadian rhythm	9	0.6828528072	0.074474783	KLF10, SUV39H2, CLDN4, SUV39H1, BHLHE40, TIMELESS, ID3, ADCY1, TYMS	1218	71	19478	2.0271282869631584	1	0.9815014738652144	0.9658448433271617
IL17A	GOTERM_BP_DIRECT	GO:0001894~tissue homeostasis	5	0.3793626707	0.07575086	BARD1, ERRFI1, NFIB, COL11A2, CD34	1218	26	19478	3.0753441960338517	1	0.9843368040557416	0.9686349451421558
IL17A	GOTERM_BP_DIRECT	GO:0045088~regulation of innate immune response	5	0.3793626707	0.07575086	IRF1, ERAP1, LRP8, BIRC2, BIRC3	1218	26	19478	3.0753441960338517	1	0.9843368040557416	0.9686349451421558
IL17A	GOTERM_BP_DIRECT	GO:0001954~positive regulation of cell-matrix adhesion	5	0.3793626707	0.07575086	FERMT1, CDK6, CSF1, CDH13, CX3CL1	1218	26	19478	3.0753441960338517	1	0.9843368040557416	0.9686349451421558
IL17A	GOTERM_BP_DIRECT	GO:0002042~cell migration involved in sprouting angiogenesis	5	0.3793626707	0.07575086	KDR, PIK3R3, SLIT2, ADTRP, ROBO1	1218	26	19478	3.0753441960338517	1	0.9843368040557416	0.9686349451421558
IL17A	GOTERM_BP_DIRECT	GO:0001836~release of cytochrome c from mitochondria	5	0.3793626707	0.07575086	GGCT, BCL2, PMAIP1, SOD2, BID	1218	26	19478	3.0753441960338517	1	0.9843368040557416	0.9686349451421558
IL17A	GOTERM_BP_DIRECT	GO:0098586~cellular response to virus	11	0.834597876	0.076639516	EIF5A, IFIH1, IFNAR2, IL6, HSP90AA1, ZC3H12A, IL12A, POU2F2, IKBKE, NFKB1, TLR3	1218	96	19478	1.8323925834701695	1	0.993102557	0.9772608690244162
IL17A	KEGG_PATHWAY	hsa04114:Oocyte meiosis	17	1.2898330804	0.077742959	ITPR2, ADCY1, PKMYT1, SMC1A, SGO1, CDC20, CCNB1, ESPL1, CCNE2, PTTG1, CCNE1, RPS6KA1, CDK2, CDK1, FBXO5, BUB1, MAD2L1	671	139	8534	1.5554793125261341	0.9999999999986818	0.5590876620301033	0.5177350243059833
IL17A	GOTERM_BP_DIRECT	GO:0045765~regulation of angiogenesis	6	0.4552352048	0.078455111	IL6, BMPER, PDE3B, VASH2, ETS1, EPHA2	1218	37	19478	2.593263213952869	1	1	0.9842604570935748
IL17A	GOTERM_BP_DIRECT	GO:0061436~establishment of skin barrier	6	0.4552352048	0.078455111	CLDN4, CYP26B1, NFKBIZ, IL18, CLDN1, TP63	1218	37	19478	2.593263213952869	1	1	0.9842604570935748

IL17A	GOTERM_BP_DIRECT	GO:2001240~negative regulation of extrinsic apoptotic signaling pathway in absence of ligand	6	0.4552352048	0.078455111	IL1A, CSF2, GDNF, BCL2, NRG1, CX3CL1	1218	37	19478	2.593263213952869	1	1	0.9842604570935748
IL17A	GOTERM_BP_DIRECT	GO:0060348~bone development	8	0.6069802731	0.079242889	PTGER4, TTC9, GJA1, BMP2, NSD2, STC1, SULF1, TMEM38B	1218	60	19478	2.132238643	1	1	0.9842604570935748
IL17A	GOTERM_BP_DIRECT	GO:0071466~cellular response to xenobiotic stimulus	9	0.6828528072	0.079435727	ABCA1, PCNA, MCM7, TFRC, ANKRD1, E2F1, CDH13, TLR3, NKX3-1	1218	72	19478	1.9989737274220034	1	1	0.9842604570935748
IL17A	KEGG_PATHWAY	hsa00670:One carbon pool by folate	7	0.5311077389	0.081417384	DHFR, MAT2A, MTHFD1, GLDC, SHMT1, TYMS, DLD	671	39	8534	2.282777332	1	0.5733140757547208	0.5309091884947562
IL17A	BIOCARTA	h_tall1Pathway:TACI and BCMA stimulation of B cell immune responses.	5	0.3793626707	0.081707741	TRAF3, TRAF2, MAP3K14, NFKB1, TNFSF13B	186	15	1622	2.9068100358422937	0.9999999800459024	0.9396390169260113	0.9305603790813156
IL17A	BIOCARTA	h_cd40Pathway:CD40L Signaling Pathway	5	0.3793626707	0.081707741	NFKBIA, TRAF3, TNFAIP3, MAP3K14, NFKB1	186	15	1622	2.9068100358422937	0.9999999800459024	0.9396390169260113	0.9305603790813156
IL17A	BIOCARTA	h_il17Pathway:IL 17 Signaling Pathway	5	0.3793626707	0.081707741	IL6, KITLG, CXCL8, CD58, CD34	186	15	1622	2.9068100358422937	0.9999999800459024	0.9396390169260113	0.9305603790813156
IL17A	GOTERM_BP_DIRECT	GO:0007169~cell surface receptor protein tyrosine kinase signaling pathway	12	0.9104704097	0.082386927	LYN, FLT1, CSF1, NTRK3, KDR, NEDD9, NRG1, SH2B3, ANGPTL1, MTSS1, SAMD10, EPHA2	1218	110	19478	1.7445588893864754	1	1	0.9842604570935748
IL17A	GOTERM_BP_DIRECT	GO:0007411~axon guidance	18	1.3657056145	0.083369512	SEMA5A, DRAXIN, SEMA7A, ROBO4, SEMA3A, NRXN3, SEMA3E, L1CAM, IGSF9, MYPN, FLRT2, APBB2, SLIT2, EPHB2, EPHB1, EPHA3, CDK5R1, NECTIN1	1218	189	19478	1.5230276018453357	1	1	0.9842604570935748
IL17A	GOTERM_BP_DIRECT	GO:0036323~vascular endothelial growth factor receptor-1 signaling pathway	7	0.5311077389	0.083378904	FLT1, NTRK3, KDR, EPHB2, EPHB1, EPHA3, EPHA2	1218	49	19478	2.2845414027680038	1	1	0.9842604570935748
IL17A	KEGG_PATHWAY	hsa04620:Toll-like receptor signaling pathway	14	1.0622154779	0.084386281	IFNAR2, CXCL8, PIK3R3, NFKB1, NFKBIA, TLR1, IL6, TRAF3, CTSK, IL12A, TLR6, IKBKE, TLR3, TLR2	671	109	8534	1.6335470815843807	0.9999999999998855	0.5820931237605123	0.5390388986302967
IL17A	GOTERM_BP_DIRECT	GO:0043616~keratinocyte proliferation	5	0.3793626707	0.084728655	FERMT1, NFKBIZ, CDH13, TP63, EREG	1218	27	19478	2.9614425591437086	1	1	0.9842604570935748
IL17A	GOTERM_BP_DIRECT	GO:0048168~regulation of neuronal synaptic plasticity	5	0.3793626707	0.084728655	EGR2, MCTP1, GPRIN1, NOG, EPHB2	1218	27	19478	2.9614425591437086	1	1	0.9842604570935748
IL17A	GOTERM_BP_DIRECT	GO:0032740~positive regulation of interleukin-17 production	5	0.3793626707	0.084728655	IL6, IL15, SPHK1, RFTN1, IL18	1218	27	19478	2.9614425591437086	1	1	0.9842604570935748
IL17A	UP_KW_BIOLOGICAL_PROCESS	KW-0233~DNA recombination	12	0.9104704097	0.084765851	INO80C, PSMC3IP, RAD51AP1, RAD51, LIG1, EME1, XRCC2, XRCC3, MND1, BRCA1, BRCA2, PALB2	824	97	11523	1.7300070063056752	0.9999958836691675	0.5067090465533083	0.46717855355978777

IL17A	GOTERM_BP_DIRECT	GO:0016264~gap junction assembly	3	0.2276176024	0.084950066	GJC1, GJA1, GJD3	1218	8	19478	5.996921182	1	1	0.9842604570935748
IL17A	GOTERM_BP_DIRECT	GO:0036035~osteoclast development	3	0.2276176024	0.084950066	ANXA2, GPR68, TNFSF11	1218	8	19478	5.996921182	1	1	0.9842604570935748
IL17A	GOTERM_BP_DIRECT	GO:0090435~protein localization to nuclear envelope	3	0.2276176024	0.084950066	TMEM201, LMNB2, LMNB1	1218	8	19478	5.996921182	1	1	0.9842604570935748
IL17A	GOTERM_BP_DIRECT	GO:0003129~heart induction	3	0.2276176024	0.084950066	BMP2, DKK1, ROBO1	1218	8	19478	5.996921182	1	1	0.9842604570935748
IL17A	GOTERM_BP_DIRECT	GO:1901164~negative regulation of trophoblast cell migration	3	0.2276176024	0.084950066	GJA1, ARHGDIB, TIMP1	1218	8	19478	5.996921182	1	1	0.9842604570935748
IL17A	GOTERM_BP_DIRECT	GO:1905832~positive regulation of spindle assembly	3	0.2276176024	0.084950066	STIL, SPAG5, SASS6	1218	8	19478	5.996921182	1	1	0.9842604570935748
IL17A	GOTERM_BP_DIRECT	GO:2000427~positive regulation of apoptotic cell clearance	3	0.2276176024	0.084950066	C3, CCL2, C2	1218	8	19478	5.996921182	1	1	0.9842604570935748
IL17A	GOTERM_BP_DIRECT	GO:0060767~epithelial cell proliferation involved in prostate gland development	3	0.2276176024	0.084950066	EAF2, NKX3-1, WDR77	1218	8	19478	5.996921182	1	1	0.9842604570935748
IL17A	GOTERM_BP_DIRECT	GO:0044208~'de novo' AMP biosynthetic process	3	0.2276176024	0.084950066	PPAT, PAICS, PFAS	1218	8	19478	5.996921182	1	1	0.9842604570935748
IL17A	GOTERM_BP_DIRECT	GO:0038111~interleukin-7-mediated signaling pathway	3	0.2276176024	0.084950066	TSLP, IL7R, JAK3	1218	8	19478	5.996921182	1	1	0.9842604570935748
IL17A	GOTERM_BP_DIRECT	GO:0110053~regulation of actin filament organization	3	0.2276176024	0.084950066	RGS4, XIRP2, C9ORF72	1218	8	19478	5.996921182	1	1	0.9842604570935748
IL17A	GOTERM_BP_DIRECT	GO:0051086~chaperone mediated protein folding independent of cofactor	3	0.2276176024	0.084950066	CCT6A, CCT2, CCT5	1218	8	19478	5.996921182	1	1	0.9842604570935748
IL17A	GOTERM_BP_DIRECT	GO:0038134~ERBB2-EGFR signaling pathway	3	0.2276176024	0.084950066	AREG, EREG, HBEGF	1218	8	19478	5.996921182	1	1	0.9842604570935748
IL17A	GOTERM_BP_DIRECT	GO:0097527~necroptotic signaling pathway	3	0.2276176024	0.084950066	MLKL, FAS, TLR3	1218	8	19478	5.996921182	1	1	0.9842604570935748
IL17A	GOTERM_BP_DIRECT	GO:1904385~cellular response to angiotensin	4	0.3034901365	0.085931323	BRIP1, INHBA, CDC6, NFKB1	1218	17	19478	3.7627740751473002	1	1	0.9842604570935748
IL17A	GOTERM_BP_DIRECT	GO:0043691~reverse cholesterol transport	4	0.3034901365	0.085931323	ABCA1, SCARB1, LIPG, ABCG1	1218	17	19478	3.7627740751473002	1	1	0.9842604570935748

IL17A	GOTERM_BP_DIRECT	GO:0042770~signal transduction in response to DNA damage	4	0.3034901365	0.085931323	ATAD5, GADD45A, CHEK1, BID	1218	17	19478	3.7627740751473002	1	1	0.9842604570935748
IL17A	GOTERM_BP_DIRECT	GO:0043388~positive regulation of DNA binding	4	0.3034901365	0.085931323	PLAUR, HES1, MMP9, NME1	1218	17	19478	3.7627740751473002	1	1	0.9842604570935748
IL17A	GOTERM_BP_DIRECT	GO:0055091~phospholipid homeostasis	4	0.3034901365	0.085931323	ABCA1, LIPG, TLCD1, ABCG1	1218	17	19478	3.7627740751473002	1	1	0.9842604570935748
IL17A	GOTERM_BP_DIRECT	GO:0031573~mitotic intra-S DNA damage checkpoint signaling	4	0.3034901365	0.085931323	TIPIN, MSH2, EME1, FANCD2	1218	17	19478	3.7627740751473002	1	1	0.9842604570935748
IL17A	GOTERM_BP_DIRECT	GO:0006998~nuclear envelope organization	4	0.3034901365	0.085931323	TMEM201, NUP155, LMNB2, LMNB1	1218	17	19478	3.7627740751473002	1	1	0.9842604570935748
IL17A	GOTERM_BP_DIRECT	GO:0006390~mitochondrial transcription	4	0.3034901365	0.085931323	POLR1A, TWNK, TFAM, PPARGC1B	1218	17	19478	3.7627740751473002	1	1	0.9842604570935748
IL17A	GOTERM_BP_DIRECT	GO:0051290~protein heterotetramerization	4	0.3034901365	0.085931323	RRM1, RRM2, GPRIN1, FARSB	1218	17	19478	3.7627740751473002	1	1	0.9842604570935748
IL17A	UP_KW_BIOLOGICAL_PROCESS	KW-0509~mRNA transport	14	1.0622154779	0.086248348	NDC1, NUP205, NUP107, SEH1L, NUP188, NUP155, NUP153, NUP85, NUP50, NUP62, NUP35, NUP88, SRSF7, NUP58	824	120	11523	1.631492718446602	0.9999967194663529	0.5067090465533083	0.46717855355978777
IL17A	GOTERM_BP_DIRECT	GO:0035790~platelet-derived growth factor receptor-alpha signaling pathway	7	0.5311077389	0.090007012	FLT1, NTRK3, KDR, EPHB2, EPHB1, EPHA3, EPHA2	1218	50	19478	2.238850574712644	1	1	0.9842604570935748
IL17A	GOTERM_BP_DIRECT	GO:0051384~response to glucocorticoid	7	0.5311077389	0.090007012	IL6, WNT7B, UCP3, BCL2, SDC1, TYMS, AREG	1218	50	19478	2.238850574712644	1	1	0.9842604570935748
IL17A	GOTERM_BP_DIRECT	GO:0038084~vascular endothelial growth factor signaling pathway	8	0.6069802731	0.090872765	FLT1, NTRK3, KDR, VEGFC, EPHB2, EPHB1, EPHA3, EPHA2	1218	62	19478	2.0634567508872292	1	1	0.9842604570935748
IL17A	KEGG_PATHWAY	hsa04640:Hematopoietic cell lineage	13	0.9863429438	0.091416553	IL4R, CSF2, CSF1, ITGA4, TFRC, ITGA2, GP1BA, IL1A, IL6, KITLG, ITGA6, IL7R, CD34	671	100	8534	1.6533830104321905	0.9999999999999916	0.6059235828198715	0.5611067497710645
IL17A	KEGG_PATHWAY	hsa04981:Folate transport and metabolism	6	0.4552352048	0.091426339	DHFR, ABCC3, MTHFD1, SHMT1, TYMS, SLC19A1	671	31	8534	2.4616124224796883	0.9999999999999916	0.6059235828198715	0.5611067497710645
IL17A	GOTERM_BP_DIRECT	GO:0032481~positive regulation of type I interferon production	6	0.4552352048	0.093758489	TRAF3, IRF1, G3BP1, UAP1, KPNA2, IKBKE	1218	39	19478	2.460275356827081	1	1	0.9842604570935748
IL17A	GOTERM_BP_DIRECT	GO:0001782~B cell homeostasis	5	0.3793626707	0.09419882	DOCK10, LYN, BCL2, IL7R, TNFSF13B	1218	28	19478	2.855676753460005	1	1	0.9842604570935748
IL17A	BIOCARTA	h_rnaPathway:Double Stranded RNA Induced Gene Expression	4	0.3034901365	0.095816804	NFKBIA, DNAJC3, MAP3K14, NFKB1	186	10	1622	3.488172043010753	0.9999999992032212	0.9951973537246378	0.9855819203553177
IL17A	BIOCARTA	h_cytokinePathway:Cytokine Network	6	0.4552352048	0.096154334	IL1A, IL6, CXCL8, IL15, IL18, IL12A	186	22	1622	2.378299120234604	0.9999999992627577	0.9951973537246378	0.9855819203553177

IL17A	KEGG_PATHWAY	hsa05202:Transcriptional misregulation in cancer	22	1.6691957511	0.096269201	ARNT2, FLT1, CXCL8, CSF2, GADD45A, DOT1L, TRAF1, ETV4, MMP9, FLI1, AFF1, NFKB1, ETV7, CCNA2, HHEX, IL6, PLAU, NFKBIZ, NSD2, BMP2K, BIRC2, BIRC3	671	198	8534	1.4131478721642656	0.9999999999999987	0.6257498089039935	0.5794665390146447
IL17A	GOTERM_BP_DIRECT	GO:0042149~cellular response to glucose starvation	7	0.5311077389	0.096912522	NUAK2, MYBBP1A, SUV39H1, ZC3H12A, BCL2, PMAIP1, UPP1	1218	51	19478	2.1949515438359253	1	1	0.9842604570935748
IL17A	GOTERM_BP_DIRECT	GO:0038109~Kit signaling pathway	7	0.5311077389	0.096912522	FLT1, NTRK3, KDR, EPHB2, EPHB1, EPHA3, EPHA2	1218	51	19478	2.1949515438359253	1	1	0.9842604570935748
IL17A	GOTERM_BP_DIRECT	GO:0045785~positive regulation of cell adhesion	8	0.6069802731	0.097027819	TNFSF18, FRMD5, ITGA2, SAA1, ADAM8, ITGAV, IL12A, NRG1	1218	63	19478	2.0307034691271144	1	1	0.9842604570935748
IL17A	GOTERM_BP_DIRECT	GO:0060384~innervation	4	0.3034901365	0.098529965	VCAM1, CHD7, LRIG1, SULF1	1218	18	19478	3.5537310709724506	1	1	0.9842604570935748
IL17A	GOTERM_BP_DIRECT	GO:0010951~negative regulation of endopeptidase activity	4	0.3034901365	0.098529965	SERPINE1, SERPINB9, TIMP1, SERPINB8	1218	18	19478	3.5537310709724506	1	1	0.9842604570935748
IL17A	GOTERM_BP_DIRECT	GO:0045591~positive regulation of regulatory T cell differentiation	4	0.3034901365	0.098529965	IL4I1, DUSP10, BTN2A2, HLA-G	1218	18	19478	3.5537310709724506	1	1	0.9842604570935748
IL17A	GOTERM_BP_DIRECT	GO:0007413~axonal fasciculation	4	0.3034901365	0.098529965	SEMA5A, EPHB2, TNFRSF21, CDK5R1	1218	18	19478	3.5537310709724506	1	1	0.9842604570935748
IL17A	GOTERM_BP_DIRECT	GO:0010569~regulation of double-strand break repair via homologous recombination	4	0.3034901365	0.098529965	RAD51AP1, RAD51, FIGNL1, CHEK1	1218	18	19478	3.5537310709724506	1	1	0.9842604570935748
IL17A	KEGG_PATHWAY	hsa05145:Toxoplasmosis	14	1.0622154779	0.099348873	HSPA8, LAMB3, LAMC2, NFKB1, NFKBIA, BCL2, PPIF, CYCS, ITGA6, IL12A, BIRC2, BIRC3, NFKBIB, TLR2	671	112	8534	1.589791356184799	0.9999999999999996	0.6335833786884618	0.586720703
